# Functionalization of α‐C(sp^3^)−H Bonds in Amides Using Radical Translocating Arylating Groups

**DOI:** 10.1002/anie.202013275

**Published:** 2020-12-30

**Authors:** Niklas Radhoff, Armido Studer

**Affiliations:** ^1^ Organisch-Chemisches Institut Westfälische Wilhelms-Universität Corrensstrasse 40 48149 Münster Germany

**Keywords:** aryl migration, hydrogen atom transfer, radical, visible light catalysis

## Abstract

α‐C−H arylation of N‐alkylamides using 2‐iodoarylsulfonyl radical translocating arylating (RTA) groups is reported. The method allows the construction of α‐quaternary carbon centers in amides. Various mono‐ and disubstituted RTA‐groups are applied to the arylation of primary, secondary, and tertiary α‐C(sp^3^)−H‐bonds. These radical transformations proceed in good to excellent yields and the cascades comprise a 1,6‐hydrogen atom transfer, followed by a 1,4‐aryl migration with subsequent SO_2_ extrusion.

## Graphical abstract

α‐C−H arylation of *N*‐alkylamides using 2‐iodoarylsulfonyl radical translocating arylating (RTA) groups is reported. The RTA group fulfills multiple roles: It acts as radical precursor, engages in remote C−H activation via hydrogen atom transfer, and serves as arylating moiety. The method allows the construction of α‐quaternary carbon centers in amides and shows broad substrate scope.

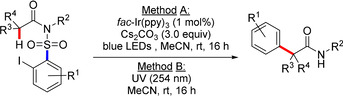

The functionalization of C−H bonds has attracted great attention in synthesis during the last 30 years.^[1]^ In this context, the principle of directed functionalization is a highly valuable strategy and many protocols for C(sp^2^)−H functionalization using transition metals (TM) as mediators or catalysts have been developed.[^1c^, ^1d^, ^2^] However, TM‐catalyzed processes for remote arylation of C(sp^3^)−H bonds are relatively scarce.[^1b^, ^3^] Complementary to TM‐mediated processes, remote radical C−H functionalization via selective hydrogen atom transfer (HAT) processes has been established.[^1c^, ^4^] HAT can be achieved to reactive N‐centered radicals, as documented early by the pioneering studies of Hofmann,^[5a]^ Löffler and Freytag.^[5b]^ Moreover, reactive C radicals can also be applied for HAT‐mediated remote C(sp^3^)−H functionalization. In the late 1980s, the group of Curran introduced the elegant concept of protecting radical translocatinrg groups by installing aryl radical precursors as protecting groups for alcohols and amines.^[6]^ The formation of a transient aryl radical then triggers a selective 1,5‐HAT to generate the corresponding translocated C radical that can be trapped, eventually leading to a remote C−H functionalization. In recent years, radical aryl migration reactions^[7]^ induced by 1,*n*‐HAT processes have emerged as a powerful tool for the regioselective arylation of C(sp^3^)−H bonds.^[8]^ Along these lines, our group developed a method for remote radical C(sp^3^)−H arylation of alcohols using 2‐iodoarylsulfonyl chlorides as radical translocating arylating groups (RTA, Scheme 1 a).^[9]^ Generation of an aryl radical in the corresponding sulfonates followed by selective 1,7‐HAT, 1,5‐aryl migration, and desulfonylation provided γ‐arylated alcohols in moderate to good yields.

**Scheme 1:**
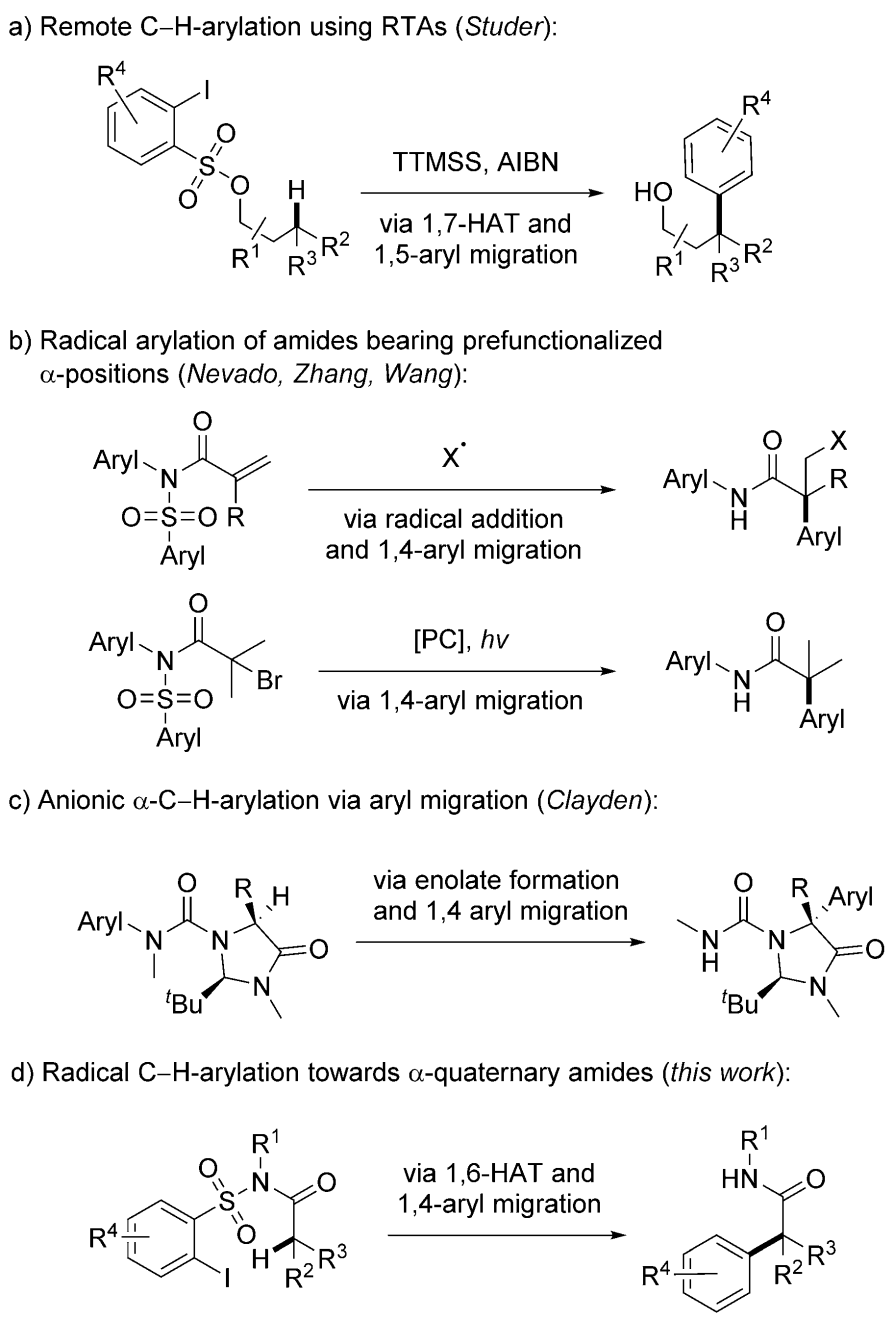
C‐arylation using radical and ionic chemistry. a) Remote C−H arylation of unactivated C(sp^3^)−H bonds using RTA groups.^[9]^ b) Radical α‐arylation of prefunctionalized amides.[^11a^, ^11b^, ^11c^, ^12^] c) Stereoselective anionic remote α‐C(sp^3^)−H arylation of amino acids.^[13]^ d) This work: remote radical C(sp^3^)−H arylation of amides under construction of an all‐carbon quaternary center.

All‐carbon α‐quaternary amides are valuable compounds in pharmacological chemistry since they can express biological activity, for example, as anti‐nausea (Netupitant*®*) and spasmolytic agents.^[10]^ Recently, three different strategies were introduced for the α‐arylation of amides proceeding via an aryl migration reaction either using radical or ionic chemistry. Importantly, these methods allow for the construction of quaternary C centers. The Nevado group used acrylamides as radical acceptors, where an initial conjugate radical addition is followed by a 1,4‐aryl migration reaction to an intermediately generated α‐amide radical to install an α‐aryl substituent (Scheme 1 b).[^11a^, ^11b^, ^11c^]

The groups of Zhang and Wang developed a similar protocol: Reduction of an α‐haloamide under photoredox conditions leads to the corresponding α‐amide radical that engages in an aryl migration reaction (Scheme 1 b).^[12]^ Of note, both strategies rely on α‐prefunctionalized amide derivatives. In 2018, the group of Clayden introduced a protocol for the stereoselective α‐C(sp^3^)−H arylation of amino acid derivatives using an anionic 1,4‐aryl migration strategy (Scheme 1 c).^[13]^

Herein, we introduce radical α‐C(sp^3^)−H arylation of amides using *N*‐alkyl‐*ortho*‐iodoarylsulfonamide‐based RTA‐chemistry. The RTA‐groups are easily incorporated into various carboxylic acid derivatives bearing an α‐C(sp^3^)−H bond via amidation of the corresponding acid chlorides with an aryl sulfonamide. The resulting sulfonamides can be converted to α‐arylated amides in a radical cascade comprising aryl radical generation, selective 1,6‐HAT followed by 1,4‐aryl migration, and reductive desulfonylation. Primary, secondary, and also tertiary C(sp^3^)−H bonds, which are highly challenging for C(sp^3^)−H arylation using transition metal‐mediated processes,[^8a^, ^9^] are efficiently functionalized by this cascade. In contrast to the previous radical‐based approaches where prefunctionalized amides were used as substrates (Scheme 1 b),[^11a^, ^11b^, ^11c^, ^12^] the introduced process operates on α‐unfunctionalized amides.

Optimization was conducted with *N*‐((2‐iodophenyl)sulfonyl)‐*N*‐isopropyl‐isobutyramide (**1 a**) as the model substrate (Table 1). The desired arylation product **2 a** was not detected by GC–MS analysis upon reacting **1 a** with azobis(isobutyronitrile) (AIBN, 0.3 equiv) and Bu_3_SnH (1.2 equiv, added over 2 h via a syringe pump) in refluxing benzene for 6 h (Table 1, entry 1). Instead, oxindole **3 a**, as a product of an homolytic aromatic substitution^[11a]^ of an intermediate amidyl radical, was isolated in 38 % yield. *fac*‐Ir(ppy)_3_ is known to reductively generate aryl radicals from aryl iodides at room temperature under irradiation with visible light.^[14]^ Pleasingly, irradiation of **1 a** in the presence of the Ir‐photocatalyst (1 mol %) and Cs_2_CO_3_ (3.0 equiv) as an additive with two blue LEDs (456 and 467 nm) for 16 h at room temperature in acetonitrile provided the desired α‐phenylated amide **2 a** in 73 % yield and oxindole **3 a** was not identified (Table 1, entry 2, Method A). Lowering the amount of Cs_2_CO_3_ afforded worse results (Table 1, entries 3–5). Notably, without added Cs_2_CO_3_, starting material could be recovered quantitatively (Table 1, entry 6). Blue light and also the Ir‐catalyst are both essential for a successful transformation (Table 1, entries 7 and 8). Switching the light source to a CFL lamp led to significantly lower yield (entry 9, Table 1). Moreover, the nature of the counterion of the carbonate was found to be crucial for the reaction outcome as the use of Na_2_CO_3_ and K_2_CO_3_ led to lower yields of amide **2 a** (Table 1, entries 10 and 11). However, catalyst‐free α‐arylation is feasible upon irradiation of a solution of **1 a** in acetonitrile (1 m) with UV light (254 nm) and the targeted product **2 a** was isolated in 82 % yield (Table 1, entry 9, Method B). With two different methods A and B in hand, the substrate scope was investigated.

**Table 1:** Reaction optimization. 
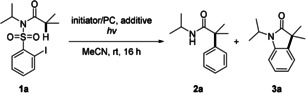

Entry	Initiator/PC	Additive	Light Source	Yield **2 a** [%]
1^[a,c]^	AIBN (30)	Bu_3_SnH (1.3)	None	n.i.
**2**	**Ir(ppy)_3_ (1)**	**Cs_2_CO_3_ (3.0)**	**blue LEDs**	**73^[b]^**
3	Ir(ppy)_3_ (1)	Cs_2_CO_3_ (2.0)	blue LEDs	67
4	Ir(ppy)_3_ (1)	Cs_2_CO_3_ (1.2)	blue LEDs	30
5	Ir(ppy)_3_ (1)	Cs_2_CO_3_ (0.5)	blue LEDs	19
6	Ir(ppy)_3_ (1)	/	blue LEDs	n.i.
7	None	Cs_2_CO_3_ (3.0)	blue LEDs	n.i.
8^[c]^	Ir(ppy)_3_ (1)	Cs_2_CO_3_ (3.0)	None	n.i.
9	Ir(ppy)_3_ (1)	Cs_2_CO_3_ (3.0)	CFL	37 %
10	Ir(ppy)_3_ (1)	Na_2_CO_3_ (3.0)	blue LEDs	22 %
11	Ir(ppy)_3_ (1)	K_2_CO_3_ (3.0)	blue LEDs	10
**12**	**None**	**/**	**254 nm**	**94 (82^[b]^)**

Reactions were conducted under an argon atmosphere on a 0.1 mmol scale. Blue LEDs: 456 and 467 nm. CFL=compact fluorescent lamp. Yields were determined by ^1^H NMR spectroscopy using 1,3,5‐trimethoxybenzene as internal standard. [a] In benzene at 95 °C for 6 h. Addition of tin hydride by syringe pump over 2 h. Yield of isolated oxindole **3 a**: 38 %. [b] Yield of isolated product. [c] In the dark. n.i.=not identified.

We first studied the influence of the N substituent R on the reaction outcome (Scheme 2). Sulfonamides **1 a**–**c** bearing a secondary *N*‐alkyl group provided the corresponding α‐phenylated amides **2 a**–**c** in good yields using the milder Ir‐mediated process (Method A, 73–85 %). Good yields for **2 a** and **2 b** were also achieved upon simple UV irradiation in the absence of the catalyst and additive (Method B, 82 and 63 %). The transformation of sulfonamide **1 a** to amide **2 a** was also feasible on a gram scale (59 % yield). The sterically less hindered *N*‐methyl amide **2 d** afforded the targeted **3 d** in 77 % using method A. However, the catalyst‐free variant did not provide a good result in this particular case (13 %). In contrast, the *N*‐benzyl‐substituted sulfonamide **1 e** was not a good substrate for the Ir‐catalyzed process (24 %), whereas method B afforded an acceptable yield (56 %). The *N*‐phenyl sulfonamide **1 f** delivered only 24 % product **2 f** using method A and with method B, **2 f** was not identified. Conformational effects exerted by the N substituents likely play a role in these transformations. Along these lines, the unsubstituted sulfonamide **1 f** did not provide the target **2 g** using either method A or B and the same result was noted for the Weinreb‐type substrate **1 h**.

**Scheme 2:**
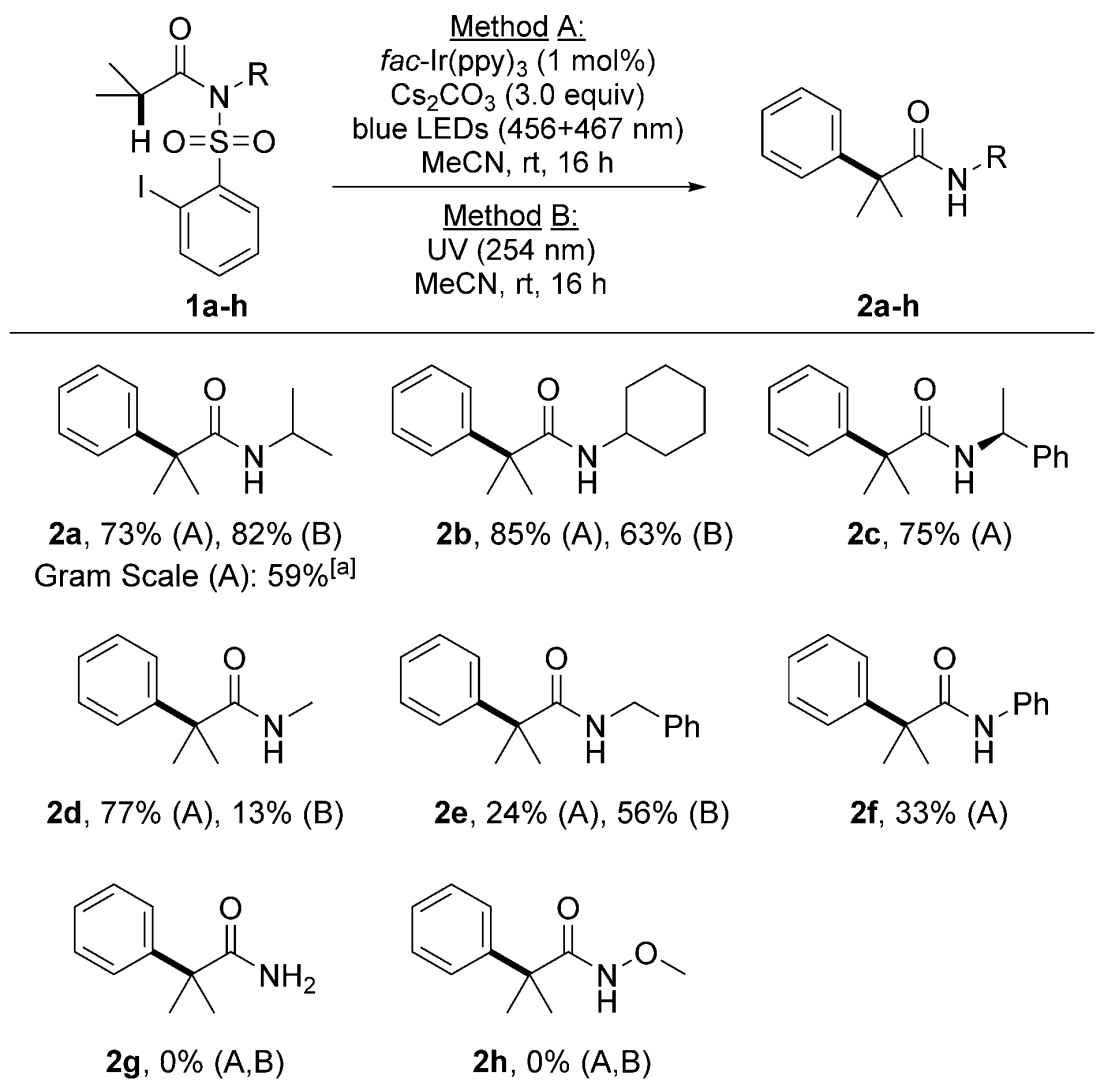
Substrate scope—variation of the N substituent R. Reaction scale between 0.1 and 0.25 m. Yields of isolated product. [a] Reaction time: 72 h.

To investigate the effect of the migrating aryl moiety on the reaction efficiency, various *N*‐isopropyl sulfonamides **1 i**–**s** were prepared (see SI). For this study, only method A was chosen (Scheme 3). We could show that *meta*‐ and *para*‐mono‐substituted phenyl groups bearing electron‐donating (methyl and methoxy) and ‐withdrawing (chloro and trifluoromethyl) substituents engaged in the transformation and the products **2 i**–**2 m** were isolated in 48–79 % yield. Arylsulfonamides **1 n**–**1 p** containing doubly substituted phenyl groups could be transformed to the corresponding α‐arylated amides **2 n**–**2 p** (62–72 %) and also the α‐naphthyl‐substituted amides **2 q** (73 %) and **2 r** (68 %) were accessible via this approach. The latter also shows the compatibility of amine functional groups with the applied conditions. The amide core of the anti‐nausea agent Netupitant*®* **2 s** (56 %) has also been prepared following our approach. However, the 2‐pyridyl derivative **2 t** and the thienyl amide **2 u** were formed in traces only. The targeted migration product was not observed for the *para*‐imide substrate **1 v**. Instead, N,S heterocycle **2′v** was formed in 76 % yield via 1,6‐HAT followed by a homolytic aromatic substitution reaction.

**Scheme 3:**
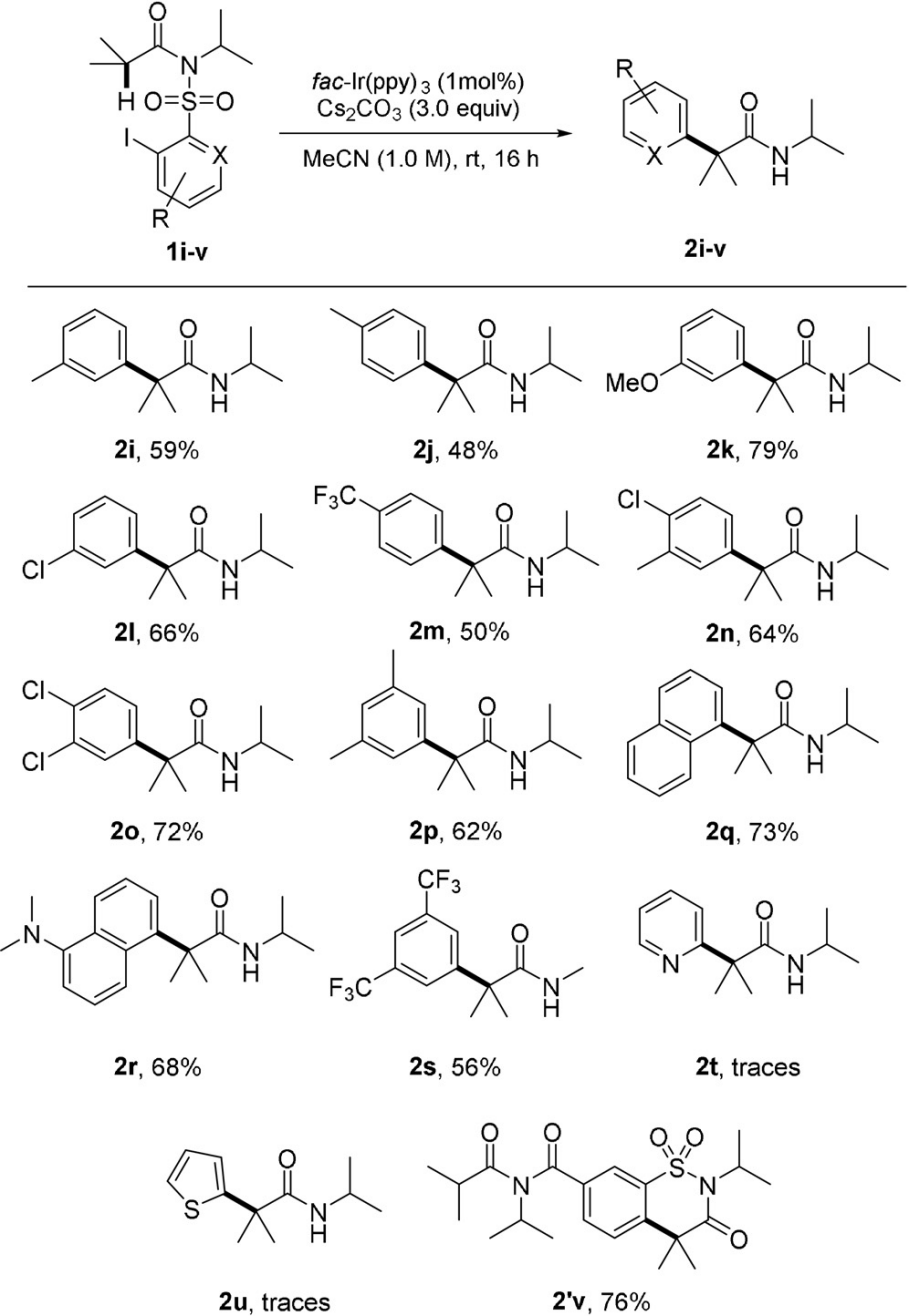
Substrate scope—variation of the radical translocating arylating group. Reaction scale: 0.1 ‐ 0.25 mmol. Yields of isolated product.

Next, we studied the effect of the α‐substituents in amides of type **1** on the novel cascade using method A (Scheme 4). Switching from the parent α,α‐dimethyl derivative **1 a** to the α‐methyl‐α‐propyl congener **1 w** did not affect the yield and **2 w** was isolated in 81 % yield. With the sterically even more hindered α‐isopropyl‐α‐methyl amide **1 x**, the yield remained good (**2 x**, 72 %) and a similar result was obtained for the cyclic amide **1 y**. We also addressed the synthesis of α,α‐diarylated amides using α‐aryl‐α‐methyl amides **1 z**–**1 ad** as substrates. For steric and also for electronic reasons (reactions proceed through more stabilized α‐amide radicals) these α‐arylations are even more challenging. We were pleased to find that all tested transformations proceeded, albeit with slightly lower efficiencies. Electronic effects of the α‐aryl substituent could be observed. Better yields were achieved with the more electron‐rich *para*‐alkyl‐phenyl amides and products **2 ac** and **2 ad** were isolated in 64 % yield. Notably, substrate **1 ad** is a readily prepared Ibuprofene*®*‐derived amide. Lower yields were noted for the *para*‐fluoro and *para*‐chloro derivatives (43–48 %), whereas the corresponding bromide **2 ab** was isolated in 62 % yield. Our method also tolerates an α‐methoxy substituent as documented by the successful preparation of **2 ae** (63 %).

**Scheme 4:**
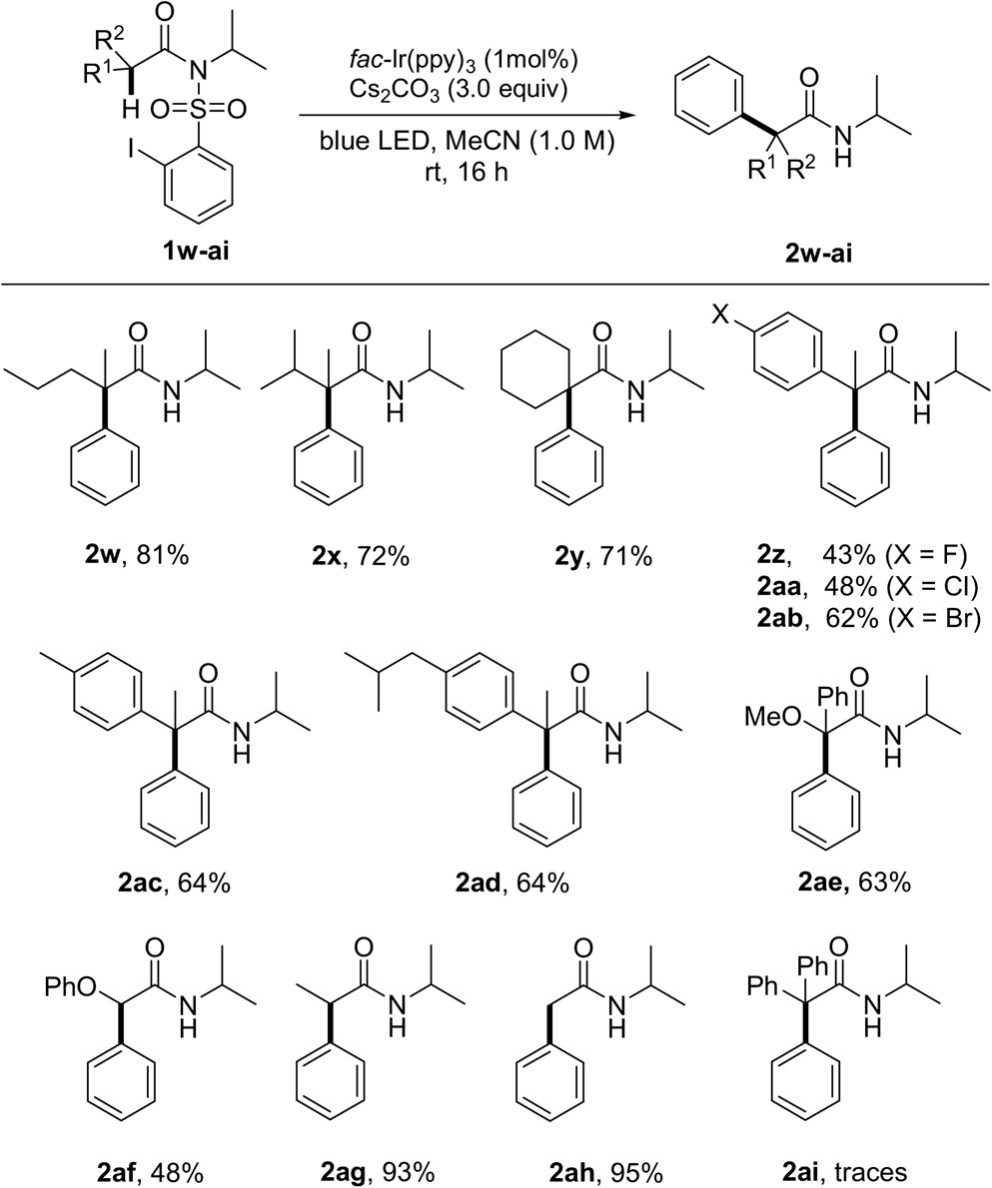
Substrate scope—primary, secondary, and tertiary α‐amide radicals. Reaction scale: 0.1–0.15 mmol. Yields of isolated product.

We continued the studies by addressing the α‐arylation of α‐monosubstituted amides. α‐Phenylation with amide **1 ag** occurred highly efficiently and targeted **2 ag** was isolated in excellent 93 % yield. A lower yield was achieved for the α‐phenoxy derivative **2 af**, likely due to the increased stability of the corresponding intermediate α‐amide radical. Not surprisingly, steric effects at the α‐position play a role and the highest yield was obtained for the α‐unsubstituted amide **1 ah** where the product **2 ah** was isolated in 95 % yield. This is in line with the fact that only traces of the sterically highly hindered product **2 ai** were observed by GC–MS and ESI‐MS. Moreover, the large stabilization of the corresponding intermediate bisbenzylic α‐amide radical also contributes to the suppression of the attempted α‐phenylation in **1 ai**.^[15]^

Plausible mechanisms for methods A and B are presented in Scheme 5. Considering method A, the aryl radical **R‐1** is reductively generated by oxidative quenching of the photoexcited Ir^III^‐catalyst.^[14]^ Stern–Volmer fluorescence quenching experiments with model substrate **1 a** and the photocatalyst support the oxidative quenching of the photocatalyst (see SI). Radical **R‐1** then undergoes a selective 1,6‐HAT to give the translocated α‐amide radical **R‐2**. *Ipso*‐attack of **R**‐**2** at the arene moiety of the RTA‐group leads to the cyclohexadienyl radical **R‐3**. Rearomatization completing the aryl migration and extrusion of SO_2_ lead to the amidyl radical **R‐4**.[^11a^, ^11b^, ^11c^, ^12^] Reduction of the amidyl radical by the excited [Ir^III^] catalyst and protonation by traces of water which is present in the reaction mixture eventually provide product **2 a**. To further support the suggested mechanism, the reaction was repeated in deuterated acetonitrile. As no deuterium incorporation into the N−H bond of the amide **2 a** was observed, hydrogen abstraction from the solvent is not likely. When deuterium oxide (20 equiv) was added to the reaction mixture, deuterium incorporation (>95 % ND) in **2 a** was obtained under standard conditions. The [Ir^III^] catalyst is regenerated upon oxidation of CO_3_^2−^ with the [Ir^IV^] complex with concomitant generation the carbonate radical anion.[^16^, ^17^] This also explains why more than one equivalent of Cs_2_CO_3_ is required. Moreover, cyclovoltammetry studies of cesium carbonate in acetonitrile support the feasibility of the reaction of Cs_2_CO_3_ with the [Ir^IV^] complex (see SI).

**Scheme 5:**
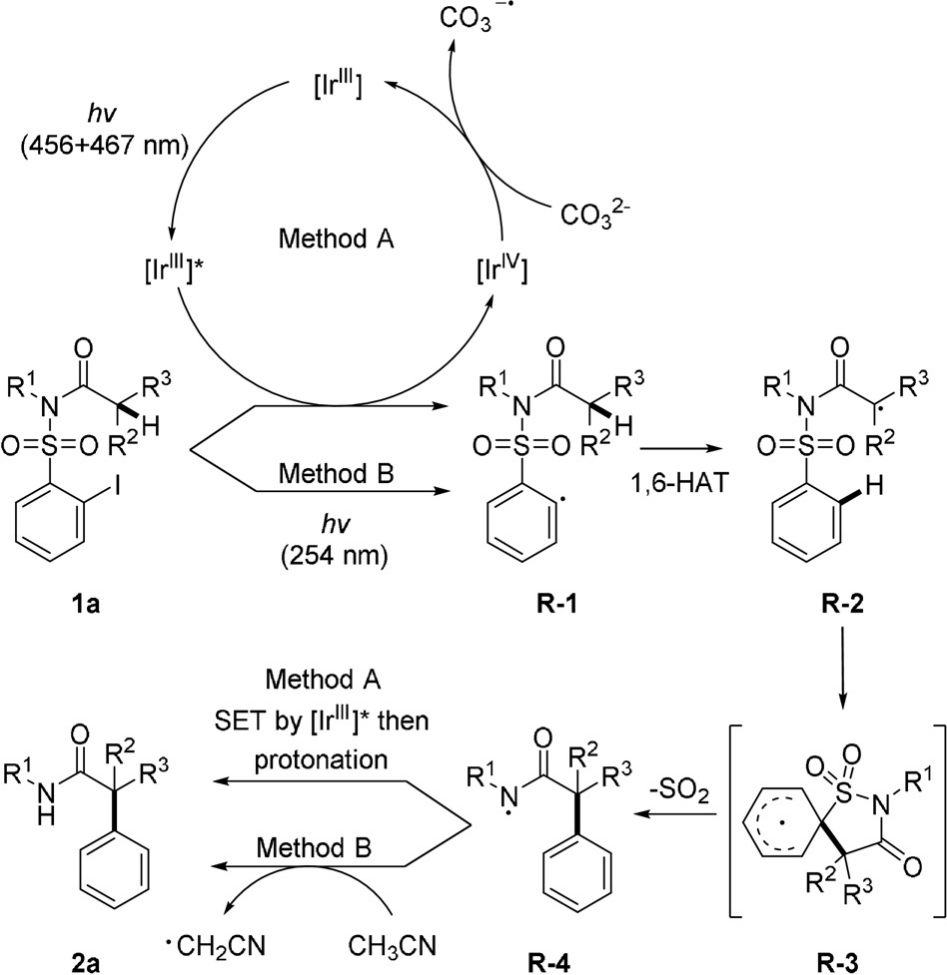
Suggested mechanism for the remote arylation of α‐C(sp^3^)−H amide bonds via a 1,6‐HAT/1,4‐aryl migration/desulfonylation cascade.

In method B, the aryl radical **R**‐**1** is generated by UV‐induced homolyis of the C−I bond in **1 a**. As for method A, 1,6‐HAT is followed by 1,4‐phenyl migration to give, after SO_2_ extrusion, the amidyl radical **R**‐**4**. Reduction by acetonitrile via intermolecular HAT finally provides product **2 a**.[^12^, ^18^] This reduction step could be further supported experimentally: UV‐light (254 nm) irradiation of **1 a** in deuterated acetonitrile provided amide **2 a‐D** with >95 % deuterium incorporation (see SI).

In summary, two protocols for arylation of α‐C(sp^3^)−H bonds in amides starting from easily accessible substrates are introduced. Reactions proceed in good to excellent yields via a 1,6‐HAT/1,4‐aryl migration/desulfonylation sequence. These radical translocation arylation cascades show broad substrate scope. Importantly, the introduced method allows for the construction of all‐carbon quaternary centers but secondary and primary α‐amide C(sp^3^)−H bonds can also be functionalized. Considering the Ir‐catalyzed process, inexpensive and environmentally benign cesium carbonate acts as the terminal reductant. Although less general and not as mild, a transition‐metal free variant for this transformation was also developed.

## Conflict of interest

The authors declare no conflict of interest.

## Supporting information

As a service to our authors and readers, this journal provides supporting information supplied by the authors. Such materials are peer reviewed and may be re‐organized for online delivery, but are not copy‐edited or typeset. Technical support issues arising from supporting information (other than missing files) should be addressed to the authors.

Supplementary
