# Supplementary material for: Functionalization of α‐C(sp3)−H Bonds in Amides Using Radical Translocating Arylating Groups
Source: Angew Chem Int Ed Engl. 2020 Dec 30;60(7):3561–5. doi: 10.1002/anie.202013275 (PMC7898318; doi:10.1002/anie.202013275)
Supplement: Supplementary file 1 — Supplementary [file ANIE-60-3561-s001.pdf]

Angewandte Chemie
Eine Zeitschrift der Gesellschaft Deutscher Chemiker

Supporting Information

Functionalization of α-C(sp^3)–H Bonds in Amides Using Radical Translocating Arylating Groups

*Niklas Radhoff and Armido Studer**

anie_202013275_sm_miscellaneous_information.pdf

Supporting Information

Table of Contents

1. General information 1

2. Cyclovoltammetry of cesium carbonate 2

 2.1 General 2

 2.2 Experimental 3

3. *Stern-Volmer* quenching analysis 3

4. Deuterium incorporation experiments 4

5. General procedures 6

6. Analytical data of compounds 9

 6.1 Synthesis 2-iodoarylsulfonic acids 9

 6.2 Synthesis of 2-iodoarylsulfonyl chlorides 12

 6.3 Synthesis of 2-iodoarylsulfonamides 15

 6.4 Synthesis of *N*-acylated 2-iodoarylsulfonamides 28

 6.5 Synthesis of α-arylated amides 51

7. NMR data of compounds 68

8. References 178

1. General information

All reactions involving air or moisture sensitive compounds have been carried out in oven-dried glassware under an argon atmosphere using standard Schlenk-technique. Prior to use, anhydrous Tetrahydrofuran (THF) used in the reactions was refluxed over sodium and freshly distilled from potassium afterwards. All other anhydrous solvents used were purchased in extra-dry grade from *Acros Organics* and stored over molecular sieves. Unless stated otherwise, all reagents used in the reactions are commercially available and have been used without further purification. They have been purchased from *Sigma Aldrich*, *Acros Organics*, *Alfa Aesar*, *ABCR*, *TCI*, *Fluorochem* and *BLDPharm*. Irradiation with blue LEDs was done with a *Kessil PR160-456 nm* lamp (456 nm, max. 40 W) and a *Kessil PR160-467 nm* lamp (467 nm, max. 40 W). UV-irradiation was conducted using *Pyrex*® quartz tubes in a *Rayonet RPPT-200* photoreactor (16 lamps, 254 nm, max. 35 W). Solvents used for chromatography and extractions were distilled prior to use. Flash column chromatography (FC) was conducted on *Merck* silica gel (40-63 µm) with an excess compressed air pressure up to 0.5 bar. For analytical as well as preparative thin layer chromatography (TLC) *Merck* silica gel 60 F254 plates have been used and compounds were detected using UV-light (254 nm) or $KMNO_4$-stain (1.5 g in 250 mL water, 5 g $NaHCO_3$). Medium pressure liquid chromatography (MPLC) was performed on an automatic flash-system by *Reveleris*® IES with commercially available 4 g, 12 g or 40 g *Reveleris*®-C_{18}-flash cartridges as the stationary phase. Detection was carried out by UV-absorption (λ = 210 nm, 230 nm) and electronic light scattering detection (ELSD) with isopropanol as carrier. Melting points were measured using a *Stuart SMP10* and are uncorrected. Infrared spectra were recorded using a *Digilab 3100 FT-IR Excalibur Series* spectrometer and the absorption bands are given in wave numbers ν (cm^{-1}). ^{1}H NMR (300 MHz and 600 MHz), ^{13}C NMR (75 MHz and 151 MHz) and ^{19}F NMR (282 MHz and 564 MHz) spectra were measured on a *Bruker DPX300* and *Agilent DD2 600* spectrometer. Chemical shifts (δ in ppm) were referenced to the solvent residual peak ($CDCl_3$: δ_H = 7.26 ppm and δ_c = 77.0 ppm; DMSO-d_6: δ_H = 2.50 ppm and δ_c = 39.5 ppm; D_2O: δ_H = 4.79 ppm). The multiplicities of all signals were described as s (singlet), d (doublet), t (triplet), q (quartet), hept (heptet), m (multiplet) and combination of the above. The ^{13}C NMR spectra of all fluorinated compounds were measured under ^{19}F-decoupling. HRMS (ESI) spectra were recorded on a *Bruker Daltonics Micro Tof* and *Thermo Fisher Scientific LTQ Orbitrap XL* spectrometer. The peaks are given in *m/z*.

2. Cyclovoltammetry of cesium carbonate

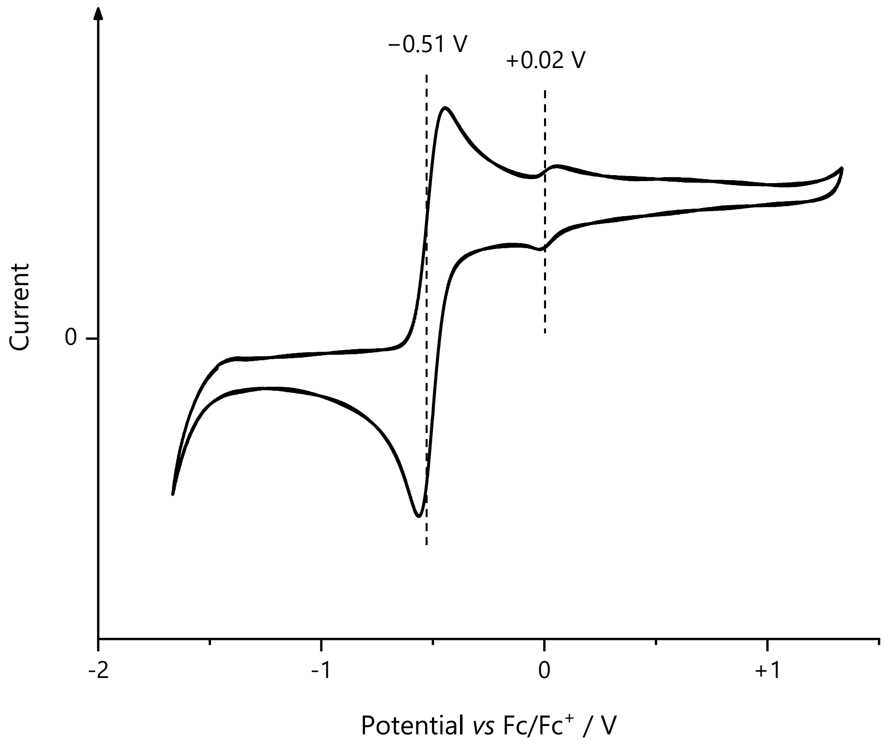

Figure 1: Cyclic voltammogram of cesium carbonate in acetonitrile with decamethylferrocene as an internal standard and $[Bu_4N^+][PF_6^-]$ (0.1 M) as the supporting electrolyte.

2.1 General

Cyclic voltammetry experiments were carried out in an air-tight three-electrode measuring cell (*rhd instruments, TSC 1600 closed*) with an approximate sample volume of 1.0 mL, with the end of a glass-isolated platinum wire (diameter 0.25 mm) as the working electrode and a platinum crucible as the counter electrode. A silver wire pseudo-reference electrode (*rhd instruments, Ag wire MicroPseudo reference*) was used. Measurements were performed using a *Metrohm Autolab* potentiostat (*Metrohm, PGSTAT204*) and data were collected and analysed using the *Autolab Nova 2.1* program.

Acetonitrile was obtained from *Acros Organics* in extra-dry grade. Tetrabutylammonium hexafluorophosphate (>99.0%, for electrochemical analysis) was purchased from *Sigma Aldrich*. Decamethylferrocene was purchased from *ABCR*. All chemicals were used without further purification.

2.2 Experimental

The substrate was poorly soluble in acetronitrile and thus measurements were carried out on a saturated solution of Cs_2CO_3 in acetonitrile with tetrabutylammonium hexafluorophosphate (0.1 M) as the supporting electrolyte. Initially ferrocene was added to the sample as an internal standard, but the ferrocene oxidation overlapped to a significant extent with oxidation of the substrate. Instead, the applied potential was referenced to the $Me_{10}Fc/Me_{10}Fc^+$ redox pair. The potential is however reported referenced to the Fc/Fc^+ redox pair for comparability, using −0.505 V for the redox potential (*vs* Fc/Fc^+) of the $Me_{10}Fc/Me_{10}Fc^+$ redox pair.[1] Due to the solubility issues, low currents were observed and AC interference can be seen in the voltammogram.

3. *Stern-Volmer* quenching analysis

Stern-Volmer fluorescence quenching analysis was carried out to identify, if sulfonamide **1a** quenches the excited photocatalyst (*fac*-Ir(ppy)$_3$). Therefore, the fluorescence of the excited photocatalyst is measured by varying concentrations of **1a**.

The quenching studies were carried out using a *JASCO-FP-8300* spectrofluorometer and *Starna*®-fluorescence quartz cuvettes (type 29 F, chamber volume = 1.400 mL, height = 48 mm, width = 12.5 mm, diameter = 10 mm, path length = 10 mm). The following data parameters were set: data interval = 0.5 nm, scan speed = 1000 nm/min, excitation wavelength λ = 420 nm, measured fluorescence wavelength λ = 540 nm.

All samples were prepared in an argon filled glovebox with degassed and dried acetonitrile, which was purchased in extra-dry grade from *Acros Organics*. The quenching studies were performed using a *fac*-Ir(ppy)$_3$-concentration of $1 \cdot 10^{-5}$ M in the respective solvent. The variable concentrations of the potential quencher were achieved by diluting stock-solutions in the cuvettes. The samples were sealed with PTFE stoppers and removed from the glovebox for the measurements.

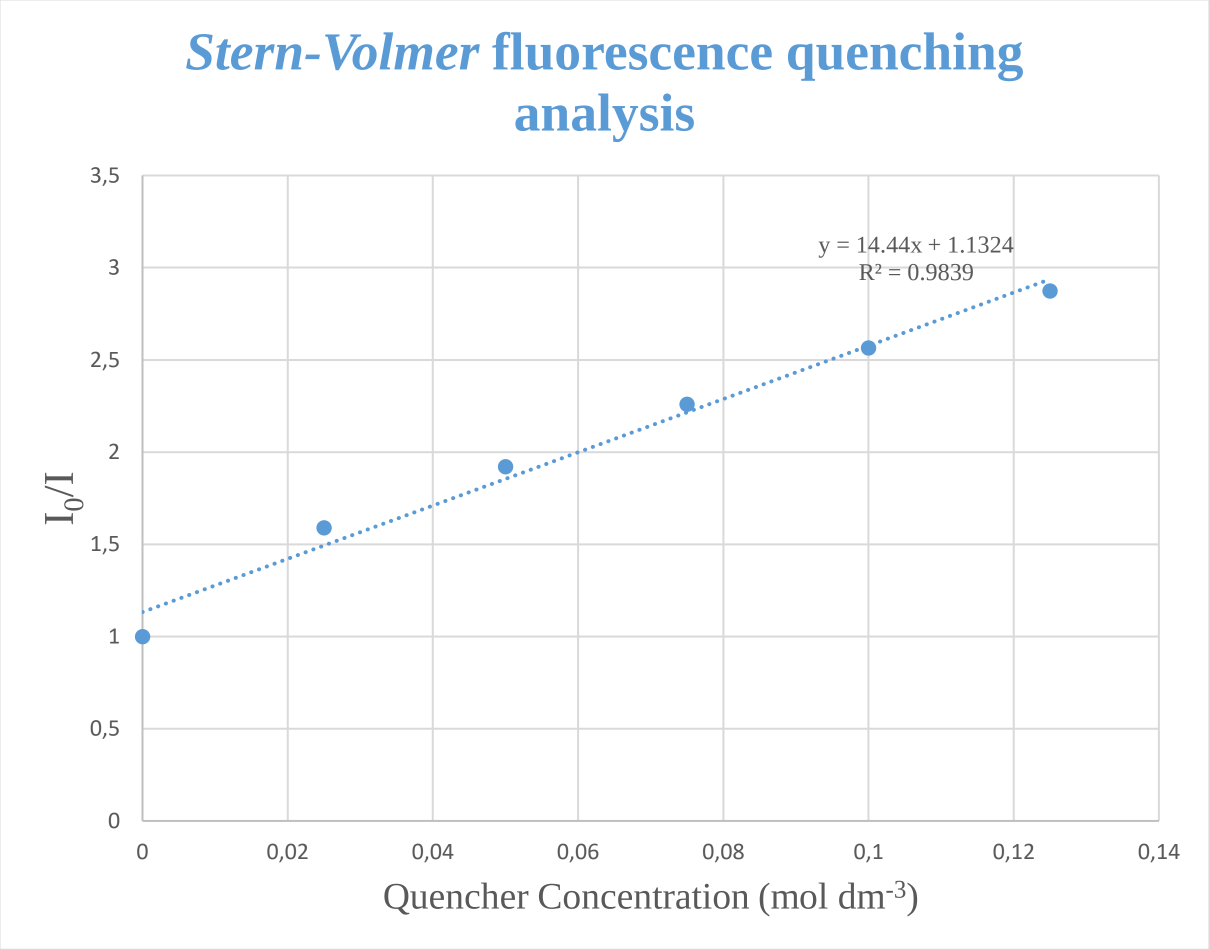

Figure 2: *Stern-Volmer* plot of the determined quenching rates against the concentration of sulfonamide **1a** in the cuvettes. Linear regression gave an R^2-value of R^2 = 0.9839. The *Stern-Volmer*-parameter has been determined to be K_{SV} = 14.44.

4. Deuterium incorporation experiments

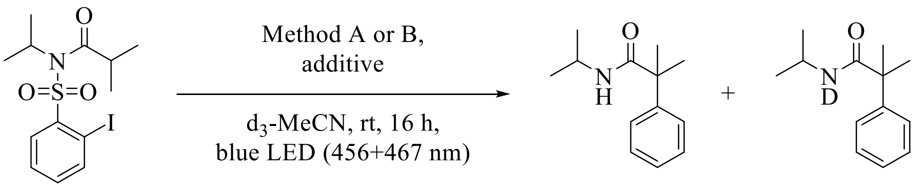

The deuterium incorporation studies of the radical arylation reaction of sulfonamide **1a** was conducted under conditions of each **Method A** and **Method B**. **Method A**: Irradiation of a one molar solution of sulfonamide **1a** in deuterated acetonitrile in the presence of *fac*-Ir(ppy)$_3$ (1 mol%) and Cs_2CO_3 (3.0 equiv.) with blue LEDs (456+467 nm). **Method B**: Irradiation of a one molar solution of sulfonamide **1a** in deuterated acetonitrile with UV-light (254 nm). Deuterium oxide (20 equiv.) was used as an additive in some cases. The reaction was conducted on a 0.1 mmol scale and analysed by ^{1}H NMR spectroscopy immediately after finishing irradiation.

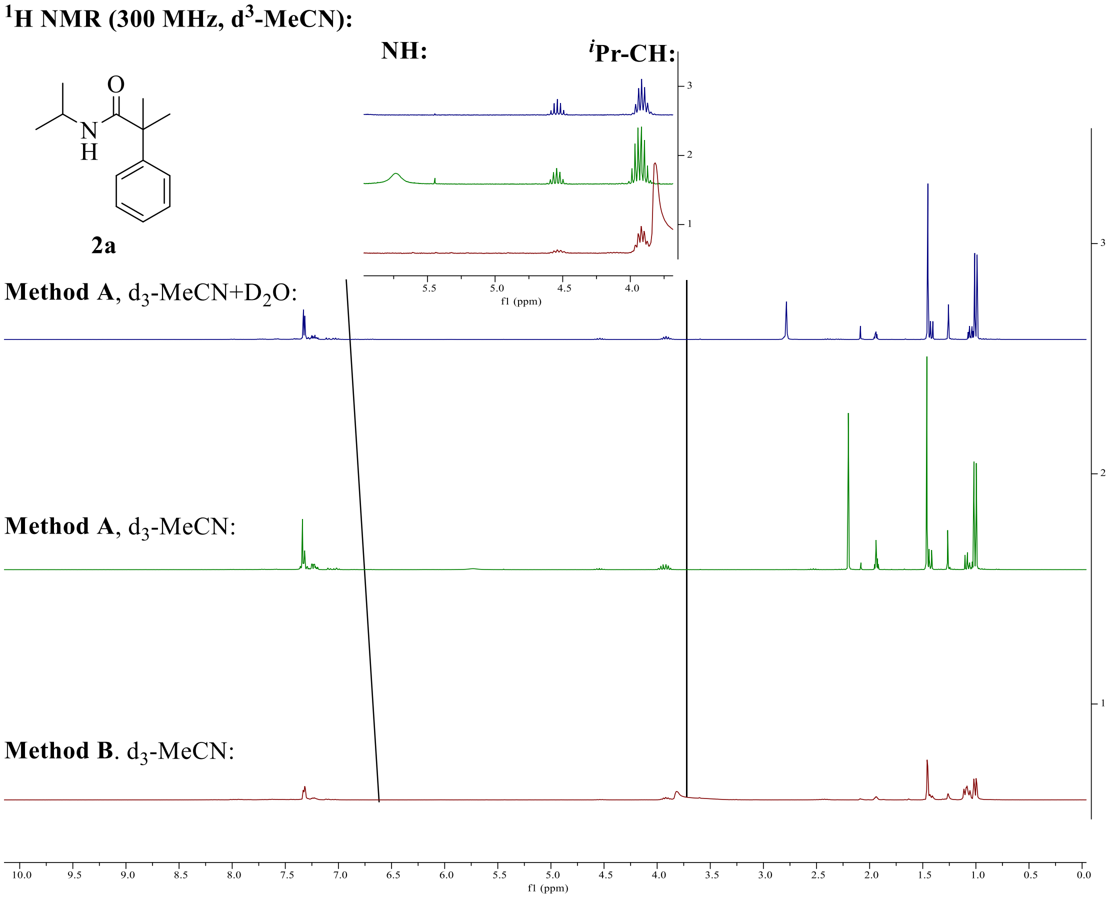

Figure 3: ^{1}H NMR analysis of the deuterium incorporation study. Top: Reaction run under conditions of **method A**. Deuterated acetonitrile was used as the solvent and deuterium oxide (20 equiv.) was used as an additive. Middle: Reaction conducted under conditions of **method A** with deuterated acetonitrile as the solvent. Bottom: Reaction under conditions of **method B**. Deuterated acetonitrile was used as the solvent.

5. General procedures

General Procedure (GP1) for the preparation of *ortho*-iodoaryl sulfonic acids (S-1 - S-7):

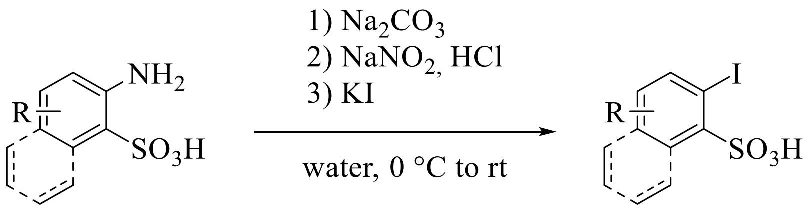

Ortho-iodoaryl sulfonic acids **S-1 - S-7** were prepared following a modified procedure by *Kice* and coworkers.[1] To a stirred solution of the corresponding *ortho*-amino sulfonic acid (1.0 equiv.) in water (0.6 M) sodium carbonate (0.5 equiv.) was added until no further gas formation was observed. The solution was cooled to 0 °C and sodium nitrite (1.1 equiv.) was added over 15 minutes. After 30 minutes of stirring at this temperature, HCl (37%, 2.0 equiv.) was added and after additional 30 minutes potassium iodide (aq., 0.72 M, 1.2 equiv.) was added dropwise. The reaction mixture was warmed to room temperature overnight. After removal of the solvent *in vacuo* the crude residue was recrystallized from water to afford the pure *ortho*-iodoaryl sulfonic acids **S-1 – S-7** as crystalline solids.

General Procedure (GP2) for the preparation of *ortho*-iodoaryl sulfonic chlorides (S-8 – S-14):

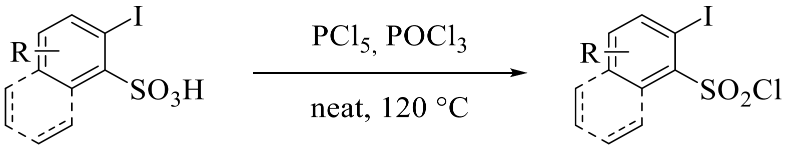

Ortho-iodoaryl sulfonic acids **S-1 – S-7** were converted to the corresponding sulfonyl chlorides **S-8 – S-14** following a literature-known procedure.[2] The neat *ortho*-iodoaryl sulfonic acids (1.0 equiv.) were treated with PCl_5 (200 w%). For some substrates additional use of $POCl_3$ (1.5 equiv.) was necessary. As the reaction mixture became homogenous it was heated to 120 °C. After one to three hours of heating the solution was poured onto ice. The aqueous phase was extracted with Et_2O (3 x), the combined organic phases were dried over $MgSO_4$ and concentrated *in vacuo*. The crude sulfonic chlorides **S-8 – S-14** were purified by flash column chromatography and obtained as crystalline solids.

General Procedure (GP3) for the preparation of *ortho*-iodoaryl sulfonamides (S-18 – S-24 and S-35 – S-37):

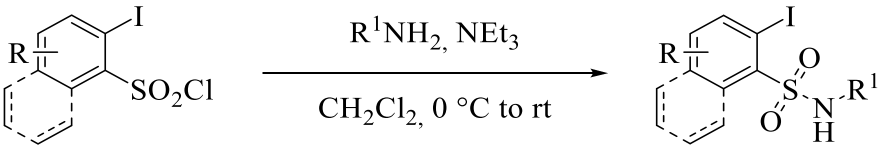

Ortho-iodoaryl sulfonamides **S-18 – S-24** and **S-35 – S-37** were prepared following a modified procedure by *Niu, Xie et al.*[3] A solution of *ortho*-iodoarylsulfonyl chloride (1.0 equiv.) and NEt_3 (2.0 equiv.) in anhydrous CH_2Cl_2 (1.0 M) was cooled to 0 °C. After adding a primary amine (1.2 equiv.) the solution was warmed to room temperature and stirred until reaction completion was indicated by TLC. The sulfonamides **S-18 – S-24** and **S-35 – S-37** were obtained by direct flash column chromatography (Pentane/EtOAc, Pentane/CH_2Cl_2 or Pentane/Et_2O) as colourless solids.

General Procedure (GP4) for the directed *ortho*-metalation-iodination (S-25 – S-30):

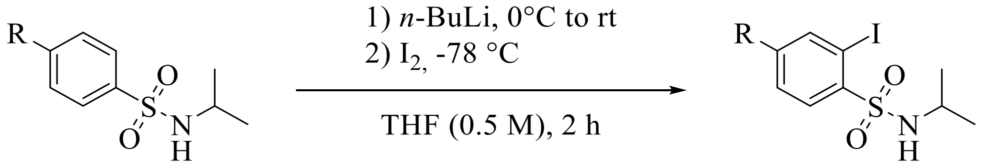

Ortho-iodoaryl sulfonamides **S-25 – S-30** were prepared following a modified procedure.[4] To a solution of sulfonamide (1.0 equiv.) in anhydrous THF (0.5 M), *n*-butyllithium (1.6 M in hexanes, 2.1 equiv.) was added dropwise at 0 °C. The reaction mixture was stirred at 0 °C for 15 minutes and then warmed to room temperature. After stirring for one hour at room temperature the solution was cooled to –78 °C and stirred for further 15 minutes. Then, a solution of iodine (1.1 equiv.) in anhydrous THF (0.73 M) was added until the brown colour persisted. The reaction mixture was stirred for one hour at –78 °C and subsequently quenched by addition of NH_4Cl (sat. aq. solution) followed by NaS_2O_5 (sat. aq. solution). The aqueous phase was extracted with EtOAc (3 x), the combined organic phases were dried over $MgSO_4$ and concentrated *in vacuo*. Separation by flash column chromatography afforded sulfonamides **S-25 – S-30** as colourless solids.

General Procedure (GP5) for the preparation of *N*-acylated *ortho*-iodoaryl sulfonamides (1a-e and 1g-aj):

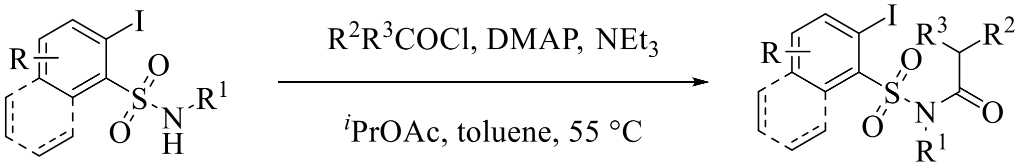

N-acylated sulfonamides **1a-e** and **1f-aj** were prepared following a procedure by *Studer* and coworkers.[5] To a solution of 4-(dimethylamino)-pyridine (DMAP, 1 mol%), NEt_3 (2.5 equiv.) and *ortho*-iodoaryl sulfonamide **S-15** and **S-18 – S-37** (1.0 equiv.) in isopropyl acetate (0.43 M) at 55 °C a solution of acyl chloride (1.1 equiv.) dissolved in anhydrous toluene (1.7 M) was added over one hour by syringe pump. The reaction mixture was stirred at this temperature overnight and subsequently quenched by HCl (ca. 2 M). The organic phase was separated and the aqueous phase was extracted by CH_2Cl_2 (3 x). The combined organic phases were dried over $MgSO_4$ and concentrated *in vacuo*. The desired *N*-acylated sulfonamides **1a-e** and **1f-aj** were obtained after flash column chromatography (Pentane/EtOAc) as colourless solids or yellowish oils.

General Procedure (GP6) for the light-mediated radical arylation reaction:

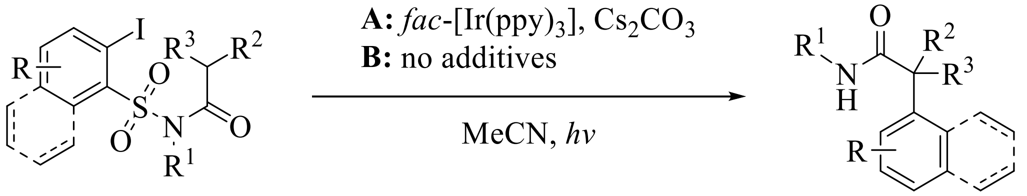

Method A: A solution of *N*-acylated sulfonamide **1a-aj** (1.0 equiv.), *fac*-Ir(ppy)$_3$ (1 mol%) and Cs_2CO_3 (3.0 equiv,) in acetonitrile (1.0 M) was degassed by three *freeze-pump-thaw*-cycles and then irradiated with blue LEDs (456 nm and 467 nm) for 16 h. The crude reaction mixture was filtered over a short plug of silica and concentrated *in vacuo*. The products **2a-aj** were obtained after reverse-phase MPLC (water/MeCN or water/MeOH, gradient from 5% to 90%) as colourless solids or yellowish oils.

Method B: A solution of *N*-acylated sulfonamide **1a-b** and **1d-e** (1.0 equiv.) in acetonitrile (1.0 M) in a *Pyrex*® quartz tube was degassed by three *freeze-pump-thaw*-cycles and then irradiated with UV-light (254 nm) for 16 h. The crude reaction mixture was filtered over a short plug of silica and sodium metabisulfite and concentrated *in vacuo*. The products **2a-b** and **2d-e**

were obtained after reverse-phase MPLC (water/MeCN or water/MeOH, gradient from 5% to 90%) as colourless solids or yellowish oils.

6. Analytical data of compounds

6.1 Synthesis 2-iodoarylsulfonic acids

2-Iodobenzenesulfonic acid (S-1):

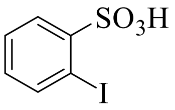

The title compound was prepared following the general procedure **GP1** using 2-aminobenzenesulfonic acid (17.3 g, 100 mmol, 1.0 equiv.), sodium carbonate (5.30 g, 50.0 mmol, 0.50 equiv.), sodium nitrite (7.59 g, 110 mmol, 1.1 equiv.), concentrated HCl (16.7 mL, 200 mmol, 2.0 equiv.) and potassium iodide (18.0 g, 120 mmol, 1.2 equiv., dissolved in 170 mL water) in water (170 mL). Recrystallisation from hot water gave acid **S-1** as orange crystals (23.3 g, 82.0 mmol, 82%).

^{1}H NMR (300 MHz, D_2O, 300 K): δ_H (ppm) = 7.94 – 7.87 (m, 2H), 7.37 – 7.32 (m, 1H), 7.03 – 6.97 (m, 1H).

^{13}C NMR (75 MHz, D_2O, 300 K): δ_C (ppm) = 150.6 (C_q), 141.2 (*C*H), 130.4 (*C*H), 128.5 (*C*H), 127.9 (*C*H), 93.7 (C_q).

HRMS (ESI) *m/z* = 282.8931 calcd. for $[C_6H_4O_3SI]^-$ $[M-H]^-$, found: 282.8939.

The analytical data are consistent with those reported in literature.[1]

2-Iodo-5-methylbenzenesulfonic acid (S-2):

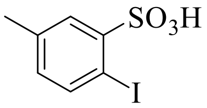

The title compound was prepared following the general procedure **GP1** using 2-amino-5-methylbenzenesulfonic acid (2.98 g, 10.0 mmol, 1.0 equiv.), sodium carbonate (530 mg, 5.00 mmol, 0.50 equiv.), sodium nitrite (759 mg, 11.0 mmol, 1.1 equiv.), concentrated HCl (2 mL) and potassium iodide (18.0 g, 120 mmol, 1.2 equiv., dissolved in 17 mL water) in water (17 mL). Recrystallisation from hot water gave acid **S-2** as a pale yellow solid (1.67 g, 5.59 mmol, 56%).

^{1}H NMR (300 MHz, D_2O, 300 K): δ_H (ppm) = 7.98 (d, *J* = 8.0 Hz, 1H), 7.86 (d, *J* = 2.2 Hz, 1H), 7.08 (dd, *J* = 8.0, 2.2 Hz, 1H), 2.35 (s, 3H).

^{13}C NMR (75 MHz, D_2O, 300 K): δ_C (ppm) = 144.6 (C_q), 141.9 (*C*H), 139.3 (C_q), 133.2 (*C*H), 129.0 (*C*H), 86.6 (C_q), 20.1 (CH_3).

HRMS (ESI) *m/z* = 296.9088 calcd. for $[C_7H_6O_3SI]^-$ $[M-H]^-$, found: 296.9078.

The analytical data are consistent with those reported in literature.[1]

5-Chloro-2-iodobenzenesulfonic acid (S-3):

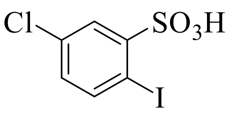

The title compound was prepared following the general procedure **GP1** using 2-amino-5-chlorobenzenesulfonic acid (2.08 g, 10.0 mmol, 1.0 equiv.), sodium carbonate (530 mg, 5.00 mmol, 0.50 equiv.), sodium nitrite (759 mg, 11.0 mmol, 1.1 equiv.), concentrated HCl (2 mL) and potassium iodide (18.0 g, 120 mmol, 1.2 equiv., dissolved in 17 mL water) in water (17 mL). Recrystallisation from hot water gave the acid **S-3** as orange crystals (1.38 g, 4.34 mmol, 43%).

^{1}H NMR (300 MHz, D_2O, 300 K): δ_H (ppm) = 8.08 – 7.03 (m, 2H), 7.27 (dd, J = 8.4, 2.5 Hz, 1H).

^{13}C NMR (75 MHz, D_2O, 300 K): δ_C (ppm) = 146.5 (C_q), 143.4 (*C*H), 134.3 (C_q), 132.2 (*C*H), 128.4 (*C*H), 88.5 (C_q).

HRMS (ESI) *m/z* = 316.8532 calcd. for $[C_6H_3O_3SClI]^-$ $[M-H]^-$, found: 316.8542.

The analytical data are consistent with those reported in literature.[1]

2-Iodo-5-methoxybenzenesulfonic acid (S-4):

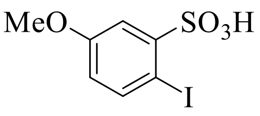

The title compound was prepared following the general procedure **GP1** using 2-amino-5-methoxybenzenesulfonic acid (2.03 g, 10.0 mmol, 1.0 equiv.), sodium carbonate (530 mg, 5.00 mmol, 0.50 equiv.), sodium nitrite (759 mg, 11.0 mmol, 1.1 equiv.), concentrated HCl (2 mL) and potassium iodide (18.0 g, 120 mmol, 1.2 equiv., dissolved in 17 mL water) in water (17 mL). Recrystallisation from hot water gave acid **S-4** as a brown solid (2.66 g, 8.47 mmol, 85%).

^{1}H NMR (300 MHz, D_2O, 300 K): δ_H (ppm) = 7.99 (d, J = 8.7 Hz, 1H), 7.62 (d, J = 3.0 Hz, 1H), 6.87 (dd, J = 8.7, 3.0 Hz, 1H), 3.87 (s, 3H).

^{13}C NMR (75 MHz, D_2O, 300 K): δ_C (ppm) = 159.2 (C_q), 146.0 (C_q), 143.0 (*C*H), 118.3 (*C*H), 114.7 (*C*H), 79.6 (C_q), 55.8 (CH_3).

HRMS (ESI) *m/z* = 312.9026 calcd. for $[C_7H_6O_4SI]^-$ $[M-H]^-$, found: 312.9037.

The analytical data are consistent with those reported in literature.[1]

4-Chloro-2-iodo-5-methylbenzenesulfonic acid (S-5):

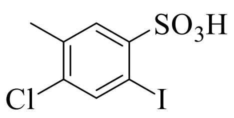

The title compound was prepared following the general procedure **GP1** using 2-amino-5-chloro-4-methylbenzenesulfonic acid (2.22 g, 10.0 mmol, 1.0 equiv.), sodium carbonate (530 mg, 5.00 mmol, 0.50 equiv.), sodium nitrite (759 mg, 11.0 mmol, 1.1 equiv.), concentrated HCl (2 mL) and potassium iodide (18.0 g, 120 mmol, 1.2 equiv., dissolved in 17 mL water) in water (17 mL). Recrystallisation from hot water gave acid **S-5** as a beige solid (2.99 g, 8.99 mmol, 90%).

^{1}H NMR (300 MHz, D_2O, 300 K): δ_H (ppm) = 8.11 (s, 1H), 7.90 (s, 1H), 2.35 (s, 3H).

^{13}C NMR (75 MHz, D_2O, 300 K): δ_C (ppm) = 143.5 (C_q), 142.4 (C_q), 141.2 (CH), 137.0 (C_q), 130.2 (CH), 87.2 (C_q), 19.0 (CH_3).

HRMS (ESI) *m/z* = 330.8687 calcd. for $[C_7H_5O_3SCClI]^-$ $[M-H]^-$, found: 330.8693.

The analytical data are consistent with those reported in literature.[1]

4,5-Dichloro-2-iodobenzenesulfonic acid (S-6):

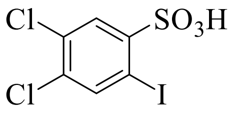

The title compound was prepared following the general procedure **GP1** using 2-amino-4,5-dichlorobenzenesulfonic acid (2.42 g, 10.0 mmol, 1.0 equiv.), sodium carbonate (530 mg, 5.00 mmol, 0.50 equiv.), sodium nitrite (759 mg, 11.0 mmol, 1.1 equiv.), concentrated HCl (2 mL) and potassium iodide (18.0 g, 120 mmol, 1.2 equiv., dissolved in 17 mL water) in water (17 mL). Recrystallisation from hot water gave acid **S-6** as a beige solid (3.08 g, 8.74 mmol, 87%).

^{1}H NMR (300 MHz, D_2O, 300 K): δ_H (ppm) = 8.27 (s, 1H), 8.11 (s, 1H).

^{13}C NMR (75 MHz, D_2O, 300 K): δ_C (ppm) = 145.1 (C_q), 142.7 (CH), 135.0 (C_q), 132.4 (C_q), 129.6 (CH), 88.7 (C_q).

HRMS (ESI) *m/z* = 350.8152 calcd. for $[C_6H_2O_3SCl_2I]^-$ $[M-H]^-$, found: 350.8148.

The analytical data are consistent with those reported in literature.[1]

2-Iodo-3,5-dimethylbenzenesulfonic acid (S-7):

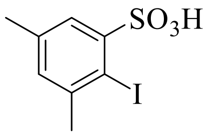

The title compound was prepared following the general procedure **GP1** using 2-amino-3,5-dimethylbenzenesulfonic acid (2.01 g, 10.0 mmol, 1.0 equiv.), sodium carbonate (530 mg, 5.00 mmol, 0.50 equiv.), sodium nitrite (759 mg, 11.0 mmol, 1.1 equiv.), concentrated HCl (2 mL) and potassium iodide (18.0 g, 120 mmol,

1.2 equiv., dissolved in 17 mL water) in water (17 mL). Recrystallisation from hot water gave acid **S-7** as pale yellow crystals (706 mg, 2.26 mmol, 23%).

^{1}H NMR (300 MHz, DMSO-d$_6$, 300 K): δ_H (ppm) = 7.60 (s, 1H), 7.08 (s, 1H), 2.38 (s, 3H), 2.22 (s, 3H).

^{13}C NMR (75 MHz, DMSO-d$_6$, 300 K): δ_C (ppm) = 151.3 (*C*q), 142.5 (*C*q), 136.7 (*C*q), 130.6 (*C*H), 127.0 (*C*H), 96.8 (*C*q), 30.3 (*C*H$_3$), 20.8 (*C*H$_3$).

HRMS (ESI) *m/z* = 310.9244 calcd. for $[C_8H_8O_3SI]^-$ $[M{-}H]^-$, found: 310.9238.

The analytical data are consistent with those reported in literature.[1]

6.2 Synthesis of 2-iodoarylsulfonyl chlorides

2-Iodobenzenesulfonyl chloride (S-8):

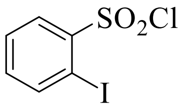

The title compound was prepared following the general procedure **GP2** using 2-iodobenzenesulfonic acid **S-1** (5.68 g, 20.0 mmol, 1.0 equiv.), PCl_5 (12.0 g, 57.6 mmol, 2.9 equiv.) and $POCl_3$ (2.9 mL, 31 mmol, 1.6 equiv.). Purification by flash column chromatography (Et_2O/Pentane, *v/v* = 1:9) gave sulfonyl chloride **S-8** as an off-white solid (4.54 g, 15.0 mmol, 75%).

^{1}H NMR (300 MHz, $CDCl_3$, 300 K): δ_H (ppm) = 8.26 – 8.21 (m, 2H), 7.63 – 7.57 (m, 1H), 7.38 – 7.33 (m, 1H).

^{13}C NMR (75 MHz, $CDCl_3$, 300 K): δ_C (ppm) = 146.4 (*C*q), 143.8 (*C*H), 135.5 (*C*H), 130.5 (*C*H), 128.9 (*C*H), 92.2 (*C*q).

The analytical data are consistent with those reported in literature.[1]

2-Iodo-5-methylbenzenesulfonyl chloride (S-9):

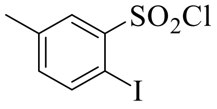

The title compound was prepared following the general procedure **GP2** using 2-iodo-5-methylbenzenesulfonic acid **S-2** (596 mg, 2.00 mmol, 1.0 equiv.) and PCl_5 (1.19 g, 200 w%). Purification by flash column chromatography (Et_2O/Pentane, *v/v* = 1:9) gave sulfonyl chloride **S-9** as an off-white solid (388 mg, 1.23 mmol, 61%).

^{1}H NMR (300 MHz, $CDCl_3$, 300 K): δ_H (ppm) = 8.08 – 8.05 (m, 2H), 7.18 – 7.15 (m, 1H), 2.43 (s, 3H).

^{13}C NMR (75 MHz, $CDCl_3$, 300 K): δ_C (ppm) = 146.0 (C_q), 143.5 (CH), 139.7 (C_q), 136.4 (CH), 131.0 (CH), 87.9 (C_q), 21.0 (CH).

The analytical data are consistent with those reported in literature.[1]

5-Chloro-2-iodobenzenesulfonyl chloride (S-10):

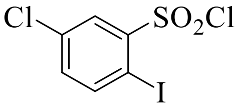

The title compound was prepared following the general procedure **GP2** using 5-chloro-2-iodobenzenesulfonic acid **S-3** (637 mg, 2.00 mmol, 1.0 equiv.), PCl_5 (1.13 g, 5.40 mmol, 2.7 equiv.) and $POCl_3$ (270 µL, 3.00 mmol, 1.5 equiv.). Purification by flash column chromatography (Et_2O/Pentane, *v/v* = 1:9) gave sulfonyl chloride **S-10** as an off-white solid (520 mg, 1.54 mmol, 77%).

^{1}H NMR (300 MHz, $CDCl_3$, 300 K): δ_H (ppm) = 8.22 (d, J = 2.5 Hz, 1H), 8.13 (d, J = 8.4 Hz, 1H), 7.34 (dd, J = 8.4, 2.5 Hz, 1H).

^{13}C NMR (75 MHz, $CDCl_3$, 300 K): δ_C (ppm) = 147.3 (C_q), 144.7 (CH), 135.6 (C_q), 135.5 (CH), 130.4 (CH), 89.3 (C_q).

The analytical data are consistent with those reported in literature.[1]

2-Iodo-5-methoxybenzenesulfonyl chloride (S-11):

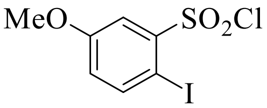

The title compound was prepared following the general procedure **GP2** using 2-iodo-5-methoxybenzensulfonic acid **S-4** (628 mg, 2.00 mmol, 1.0 equiv.), PCl_5 (1.13 g, 5.40 mmol, 2.7 equiv.) and $POCl_3$ (270 µL, 3.00 mmol, 1.5 equiv.). Purification by flash column chromatography (Et_2O/Pentane, *v/v* = 1:4) gave sulfonyl chloride **S-11** as a brown solid (388 mg, 1.17 mmol, 58%).

^{1}H NMR (300 MHz, $CDCl_3$, 300 K): δ_H (ppm) = 8.00 (d, J = 8.7 Hz, 1H), 7.62 (d, J = 3.0 Hz, 1H), 6.87 (dd, J = 8.7, 3.0 Hz, 1H), 3.89 (s, 3H).

^{13}C NMR (75 MHz, $CDCl_3$, 300 K): δ_C (ppm) = 159.9 (C_q), 146.9 (C_q), 144.2 (CH), 122.2 (CH), 115.8 (CH), 79.9 (C_q), 56.0 (CH$_3$).

The analytical data are consistent with those reported in literature.[1]

4-Chloro-2-iodo-5-methylbenzenesulfonyl chloride (S-12):

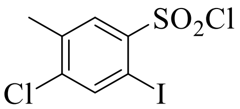

The title compound was prepared following the general procedure **GP2** using 4-chloro-2-iodo-5-methylbenzenesulfonic acid **S-5** (665 mg, 2.00 mmol, 1.0 equiv.), PCl_5 (1.13 g, 5.40 mmol, 2.7 equiv.) and $POCl_3$

(270 µL, 3.00 mmol, 1.5 equiv.). Purification by flash column chromatography (Et_2O/Pentane, *v/v* = 1:99) gave sulfonyl chloride **S-12** as a pale yellow solid (526 mg, 1.50 mmol, 75%).

^{1}H NMR (300 MHz, $CDCl_3$, 300 K): δ_H (ppm) = 8.17 (s, 1H), 8.09 (s, 1H), 2.44 (s, 3H).

^{13}C NMR (75 MHz, $CDCl_3$, 300 K): δ_C (ppm) = 144.5 (C_q), 143.1 (*C*H), 141.8 (C_q), 137.8 (C_q), 132.0 (*C*H), 88.4 (C_q), 19.9 (*C*H).

The analytical data are consistent with those reported in literature.[1]

4,5-Dichloro-2-iodo-benzenesulfonyl chloride (S-13):

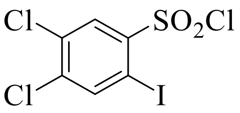

The title compound was prepared following the general procedure **GP2** using 4,5-dichloro-2-iodobenzenesulfonic acid **S-6** (706 mg, 2.00 mmol, 1.0 equiv.), PCl_5 (1.13 g, 5.40 mmol, 2.7 equiv.) and $POCl_3$ (270 µL, 3.00 mmol, 1.5 equiv.). Purification by flash column chromatography (Et_2O/Pentane, *v/v* = 1:99) gave sulfonyl chloride **S-13** as a colourless solid (583 mg, 1.57 mmol, 79%).

^{1}H NMR (300 MHz, $CDCl_3$, 300 K): δ_H (ppm) = 8.28 (s, 2H).

^{13}C NMR (75 MHz, $CDCl_3$, 300 K): δ_C (ppm) = 145.4 (C_q), 144.3 (*C*H), 140.2 (C_q), 134.0 (C_q), 131.5 (*C*H), 89.3 (C_q).

The analytical data are consistent with those reported in literature.[1]

2-Iodo-3,5-dimethylbenzenesulfonyl chloride (S-14):

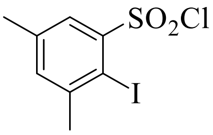

The title compound was prepared following the general procedure **GP2** using 2-iodo-3,5-dimethylbenzenesulfonic acid **S-7** (675 mg, 2.16 mmol, 1.00 equiv.), PCl_5 (1.21 g, 5.83 mmol, 2.70 equiv.) and $POCl_3$ (300 µL, 3.24 mmol, 1.50 equiv.). Purification by flash column chromatography (Et_2O/Pentane, *v/v* = 1:99) gave sulfonyl chloride **S-14** as a colourless solid (583 mg, 1.57 mmol, 79%).

^{1}H NMR (300 MHz, $CDCl_3$, 300 K): δ_H (ppm) = 7.90 (s, 1H), 7.39 (s, 1H), 2.59 (s, 3H), 2.39 (s, 3H).

^{13}C NMR (75 MHz, $CDCl_3$, 300 K): δ_C (ppm) = 146.9 (C_q), 146.4 (C_q), 138.9 (C_q), 136.1 (*C*H), 129.0 (*C*H), 94.9 (C_q), 30.2 (*C*H_3), 20.8 (*C*H_3).

The analytical data are consistent with those reported in literature.[1]

6.3 Synthesis of 2-iodoarylsulfonamides

2-Iodo-*N*-methylbenzenesulfonamide (S-15):

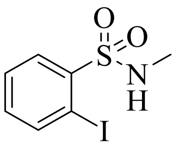

The title compound was prepared following a procedure of *Rabai*.[6] To this end, methylamine (2.0 M solution in 1,4-Dioxane, 0.70 mL, 1.4 mmol, 1.1 equiv.) and 2-iodobenzenesulfonyl chloride **S-8** (0.36 g, 1.2 mmol, 1.0 equiv.) were dissolved in 1,4-dioxane (1.2 ml) at room temperature and stirred overnight. After removal of the solvent *in vacuo* the desired sulfonamide was obtained after flash column chromatography (Et_2O/Pentane, *v/v* = 1:1) and as a colourless solid (0.20 g, 0.66 mmol, 55%).

^{1}H NMR (300 MHz, $CDCl_3$, 300 K): δ_H (ppm) = 8.15 (dd, *J* = 7.9, 1.7 Hz, 1H), 8.08 (dd, *J* = 7.9, 1.2 Hz, 1H), 7.53 (td, *J* = 7.7, 1.2 Hz, 1H), 7.24 (td, *J* = 7.7, 1.7 Hz, 1H), 5.18 (s, 1H), 2.60 (d, *J* = 5.4 Hz, 3H).

^{13}C NMR (75 MHz, $CDCl_3$, 300 K): δ_C (ppm) = 142.2 (*C*H), 140.8 (C_q), 133.5 (*C*H), 131.7 (*C*H), 128.6 (*C*H), 92.0 (C_q), 29.2 (CH_3).

HRMS (ESI) *m/z* = 319.9213 calcd. for $[C_7H_8NO_2SINa]^+$, found: 319.9209.

The analytical data are consistent with those reported in literature.[6]

2-Iodo-*N*-phenylbenzenesulfonamide (S-16):

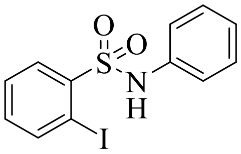

The title compound was prepared following a procedure of *Demonceau* and coworkers.[7] Aniline (0.12 mL, 1.3 mmol, 1.1 equiv.) and pyridine (0.14 mL, 1.8 mmol, 1.5 equiv.) were dissolved in anhydrous CH_2Cl_2 (2 mL) at room temperature. Subsequently, 2-iodobenzenesulfonyl chloride **S-8** (0.36 g, 1.2 mmol, 1.0 equiv.) was added and the mixture was stirred at room temperature overnight. Removal of the solvent and purification by flash column chromatography (Et_2O/Pentane, *v/v* = 1:1) lead to isolation of sulfonamide **S-3b** as a colourless solid (0.40 g, 1.1 mmol, 92%).

^{1}H NMR (300 MHz, $CDCl_3$, 300 K): δ_H (ppm) = 8.08 – 8.02 (m, 2H), 7.43 – 7.38 (m, 1H), 7.27 – 7.07 (m, 6H).

^{13}C NMR (75 MHz, $CDCl_3$, 300 K): δ_C (ppm) = 142.2 (*C*H), 141.1 (C_q), 135.7 (C_q), 133.7 (*C*H), 132.0 (*C*H), 129.3 (*C*H), 128.5 (*C*H), 125.7 (*C*H), 121.8 (*C*H), 92.2 (C_q).

HRMS (ESI) *m/z* = 381.9369 calcd. for $[C_{12}H_{10}NO_2SINa]^+$, found: 381.9368.

The analytical data are consistent with those reported in literature.[7]

2-Iodo-*N*-methoxybenzenesulfonamide (S-17):

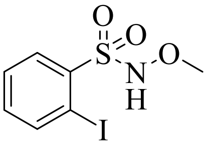

The title compound was prepared following a modified procedure by *Tabuchi, Yamamoto* and *Nakayama*.[8] *O*-Methylhydroxylammonium chloride (251 mg, 3.00 mmol, 1.14 equiv.) was dissolved in pyridine (1.1 mL) at 0 °C. After slow addition of 2-iodobenzenesulfonyl chloride **S-8** (796 mg, 2.63 mmol, 1.00 equiv.) the reaction mixture was allowed to reach room temperature overnight. The reaction was quenched by addition of water (5 mL). The aqueous phase was extracted with EtOAc (3 x 10 mL) and the combined organic phases were washed with water, HCl (ca. 2 M) and brine. The organics were dried over $MgSO_4$ and concentrated *in vacuo*. The title compound **S-3c** was obtained as a pale yellow solid (767 mg, 2.45 mmol, 93%).

MP: 101-102 °C.

FT IR (neat) υ (cm^{-1}) = 3213, 1568, 1449, 1427, 1382, 1338, 1711, 1043, 1013, 906, 761, 738, 671, 566.

^{1}H NMR (300 MHz, $CDCl_3$, 300 K): δ_H (ppm) = 8.27 – 8.24 (m, 1H), 8.13 – 8.08 (m, 1H), 7.57 (ddd, *J* = 8.0, 7.5, 1.2 Hz, 1H), 7.32 – 7.27 (m, 1H), 3.80 (s, 3H).

^{13}C NMR (75 MHz, $CDCl_3$, 300 K): δ_C (ppm) = 142.2 (*C*H), 139.2 (C_q), 134.4 (*C*H), 133.3 (*C*H), 128.7 (*C*H), 92.1 (C_q), 65.1 (CH_3).

HRMS (ESI) *m/z* = 335.9162 calcd. for $[C_7H_8NO_3SINa]^+$ $[M+Na]^+$, found: 335.9160.

2-Iodo-*N*-isopropylbenzenesulfonamide (S-18):

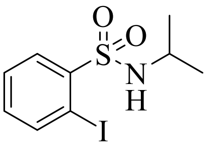

The title compound was prepared following general procedure **GP3** using isopropylamine (0.17 mL, 2.0 mmol, 1.2 equiv.), 2-iodobenzenesulfonyl chloride **S-8** (0.52 g, 1.7 mmol, 1.0 equiv.) and NEt_3 (0.46 mL, 3.4 mmol, 2.0 equiv.) in CH_2Cl_2. Purification by flash column chromatography (Et_2O/Pentane, *v/v* = 1:1) afforded sulfonamide **S-18** as a colourless solid (0.54 g, 1.7 mmol, 99%).

MP: 102-103 °C.

FT IR (neat) υ (cm^{-1}) = 3312, 2974, 2931, 1569, 1422, 1333, 1033, 1158, 1121, 1017, 760, 724, 641, 591, 570.

^{1}H NMR (300 MHz, $CDCl_3$, 300 K): δ_H (ppm) = 8.21 (dd, *J* = 7.9, 1.7 Hz, 1H), 8.08 (dd, *J* = 7.9, 1.3 Hz, 1H), 7.51 (td, *J* = 7.7, 1.2 Hz, 1H), 7.22 (td, *J* = 7.7, 1.7 Hz, 1H), 5.09 (d, *J* = 7.4 Hz, 1H), 3.52 – 3.35 (m, 1H), 1.11 (d, *J* = 6.5 Hz, 6H).

^{13}C NMR (75 MHz, $CDCl_3$, 300 K): δ_C (ppm) = 143.0 (C_q), 142.2 (*C*H), 133.2 (*C*H), 131.0 (*C*H), 128.5 (*C*H), 92.4 (C_q), 46.5 (*C*H), 23.5 (CH_3).

HRMS (ESI) *m/z* = 347.9526 calcd. for $[C_9H_{12}NO_2SINa]^+$ $[M+Na]^+$, found: 347.9543.

2-Iodo-*N*-isopropyl-5-methylbenzenesulfonamide (S-19):

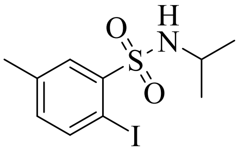

The title compound was prepared following general procedure **GP3** using 2-iodo-5-methylbenzenesulfonyl chloride **S-9** (317 mg, 1.00 mmol, 1.0 equiv.), isopropylamine (105 µL, 1.20 mmol, 1.2 equiv.) and NEt_3 (280 µL, 2.00 mmol, 2.0 equiv.) in CH_2Cl_2 (1 mL). Purification by flash column chromatography (EtOAc/pentane, *v/v* = 1:9) afforded sulfonamide **S-19** as a colourless solid (314 mg, 0.927 mmol, 93%).

MP: 121-122 °C.

FT IR (neat) υ (cm^{-1}) = 3313, 2975, 2929, 2874, 1456, 1413, 1387, 1302, 1157, 1131, 1099, 1015, 996, 893, 819, 690, 600, 578.

^{1}H NMR (300 MHz, $CDCl_3$, 300 K): δ_H (ppm) = 8.01 (d, *J* = 2.2 Hz, 1H), 7.90 (d, *J* = 8.0 Hz, 1H), 7.02 (ddd, *J* = 8.0, 2.2, 0.9 Hz, 1H), 5.12 (d, *J* = 7.5 Hz, 1H), 3.47 – 3.32 (m, 1H), 2.38 (s, 3H), 1.10 (d, *J* = 6.5 Hz, 6H).

^{13}C NMR (75 MHz, $CDCl_3$, 300 K): δ_C (ppm) = 142.5 (C_q), 142.0 (*C*H), 139.1 (C_q), 134.2 (*C*H), 131.7 (*C*H), 88.2 (C_q), 46.5 (*C*H), 23.5 (CH_3), 20.9 (CH_3).

HRMS (ESI) *m/z* = 361.9682 calcd. for $[C_{10}H_{14}NO_2SINa]^+$ $[M+Na]^+$, found: 361.9682.

5-Chloro-2-iodo-*N*-isopropylbenzenesulfonamide (S-20):

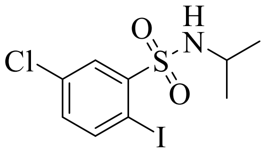

The title compound was prepared following general procedure **GP3** using 5-chloro-2-iodo-benzenesulfonyl chloride **S-10** (337 mg, 1.0 mmol, 1.0 equiv.), isopropylamine (105 µL, 1.20 mmol, 1.2 equiv.) and NEt_3 (280 µL, 2.00 mmol, 2.0 equiv.) in CH_2Cl_2 (1 mL). Purification by flash column chromatography (EtOAc/pentane, *v/v* = 1:9) afforded sulfonamide **S-20** as a colourless solid (325 mg, 0.904 mmol, 90%).

MP: 124-125 °C.

FT IR (neat) υ (cm^{-1}) = 3306, 3086, 2973, 2934, 1442, 1414, 1365, 1333, 1247, 1159, 1126, 1103, 1016, 998, 861, 821, 695, 670. 559, 579.

^{1}H NMR (300 MHz, $CDCl_3$, 300 K): δ_H (ppm) = 8.18 (d, *J* = 2.5 Hz, 1H), 7.97 (d, *J* = 8.4 Hz, 1H), 7.19 (dd, *J* = 8.4, 2.5 Hz, 1H), 5.17 (d, *J* = 7.6 Hz, 1H), 3.52 – 3.37 (m, 1H), 1.12 (d, *J* = 6.5 Hz, 6H).

^{13}C NMR (75 MHz, $CDCl_3$, 300 K): δ_C (ppm) = 144.6 (C_q), 143.2 (*C*H), 135.3 (C_q), 133.3 (*C*H), 131.0 (*C*H), 89.5 (C_q), 46.7 (*C*H), 23.5 (CH_3).

HRMS (ESI) *m/z* = 381.9136 calcd. for $[C_9H_{11}NO_2SClINa]^+$ $[M+Na]^+$, found: 381.9137.

2-Iodo-*N*-isopropyl-5-methoxybenzenesulfonamide (S-21):

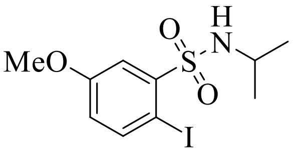

The title compound was prepared following general procedure **GP3** using 2-iodo-5-methoxybenzenesulfonyl chloride **S-11** (333 mg, 1.00 mmol, 1.0 equiv.), isopropylamine (105 µL, 1.20 mmol, 1.2 equiv.) and NEt_3 (280 µL, 2.00 mmol, 2.0 equiv.) in CH_2Cl_2 (1 mL). Purification by flash column chromatography (EtOAc/pentane, *v/v* = 1:9) afforded sulfonamide **S-21** as a colourless solid (323 mg, 0.908 mmol, 91%).

MP: 110-111 °C.

FT IR (neat) υ (cm^{-1}) = 3312, 2973, 3937, 2841, 1584, 1460, 1436, 1334, 1289, 1260, 1229, 1158, 1125, 1037, 1010, 892, 821, 691, 620, 586, 550.

^{1}H NMR (300 MHz, $CDCl_3$, 300 K): δ_H (ppm) = 7.80 (d, *J* = 8.6 Hz, 1H), 7.66 (d, *J* = 3.1 Hz, 1H), 6.68 (dd, *J* = 8.6, 3.1 Hz, 1H), 5.05 (d, *J* = 7.5 Hz, 1H), 3.76 (s, 3H), 3.40 – 3.25 (m, 1H), 1.02 (d, *J* = 6.6 Hz, 6H).

^{13}C NMR (75 MHz, $CDCl_3$, 300 K): δ_C (ppm) = 159.9 (C_q), 143.8 (C_q), 142.8 (*C*H), 119.7 (*C*H), 116.7 (*C*H), 80.3 (C_q), 55.8 (CH_3), 46.6 (*C*H), 23.5 (CH_3).

HRMS (ESI) *m/z* = 377.9631 calcd. for $[C_{10}H_{14}NO_3SINa]^+$ $[M+Na]^+$, found: 377.9631.

4-Chloro-2-iodo-*N*-isopropyl-5-methylbenzenesulfonamide (S-22):

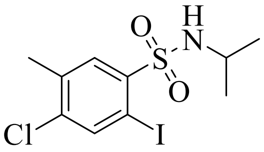

The title compound was prepared following general procedure **GP3** using 4-chloro-2-iodo-5-methylbenzenesulfonyl chloride **S-12** (351 mg, 1.00 mmol, 1.0 equiv.), isopropylamine (105 µL, 1.20 mmol, 1.2 equiv.) and NEt_3 (280 µL, 2.00 mmol, 2.0 equiv.) in CH_2Cl_2 (1 mL). Purification by flash column chromatography (EtOAc/pentane, *v/v* = 1:9) sulfonamide **S-22** as a colourless solid (348 mg, 0.931 mmol, 93%).

MP: 165-166 °C.

FT IR (neat) υ (cm^{-1}) = 3322, 3281, 2975, 2934, 1570, 1448, 1423, 1342, 1173, 1160, 1142, 1110, 1064, 1010, 908, 714, 632.

^{1}H NMR (300 MHz, $CDCl_3$, 300 K): δ_H (ppm) = 8.05 (s, 1H), 8.02 (s, 1H), 5.03 (d, J = 7.5 Hz, 1H), 3.49 – 3.33 (m, 1H), 2.40 (s, 3H), 1.13 (d, J = 6.6 Hz, 6H).

^{13}C NMR (75 MHz, $CDCl_3$, 300 K): δ_C (ppm) = 141.6 (*C*H), 141.3 (C_q), 138.9 (C_q), 137.2 (C_q), 132.8 (*C*H), 88.6 (C_q), 46.6 (C_q), 23.5 (CH_3), 19.8 (*C*H).

HRMS (ESI) *m/z* = 395.9292 calcd. for $[C_{10}H_{13}NO_2SClINa]^+$ $[M+Na]^+$, found: 395.9294.

4,5-Dichloro-2-iodo-*N*-isopropylbenzenesulfonamide (S-23):

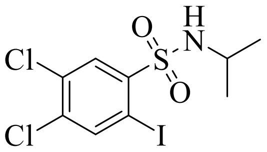

The title compound was prepared following general procedure **GP3** using 4,5-dichloro-2-iodobenzenesulfonyl chloride **S-13** (371 mg, 1.00 mmol, 1.0 equiv.), isopropylamine (105 µL, 1.20 mmol, 1.2 equiv.) and NEt_3 (280 µL, 2.00 mmol, 2.0 equiv.) in CH_2Cl_2 (1 mL). Purification by flash column chromatography (EtOAc/pentane, *v/v* = 1:9) afforded sulfonamide **S-23** as a colourless solid (462 mg, 1.00 mmol, 99%).

MP: 159-160 °C.

FT IR (neat) υ (cm^{-1}) = 3293, 3087, 2971, 1528, 1436, 1325, 1174, 1159, 1145, 1105, 1048, 1016, 902, 848, 680, 556.

^{1}H NMR (300 MHz, $CDCl_3$, 300 K): δ_H (ppm) = 8.26 (s, 1H), 8.13 (s, 1H), 5.04 (d, J = 7.6 Hz, 1H), 3.54 – 3.38 (m, 1H), 1.15 (d, J = 6.5 Hz, 6H).

^{13}C NMR (75 MHz, $CDCl_3$, 300 K): δ_C (ppm) = 142.9 (*C*H), 142.8 (C_q), 137.2 (C_q), 133.7 (C_q), 132.1 (*C*H), 89.4 (C_q), 46.8 (*C*H), 23.6 (CH_3).

HRMS (ESI) *m/z* = 415.8746 calcd. for $[C_9H_{10}NO_2SCl_2INa]^+$ $[M+Na]^+$, found: 415.8743.

2-Iodo-N-isopropyl-3,5-dimethylbenzenesulfonamide (S-24):

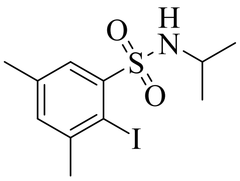

The title compound was prepared following general procedure **GP3** using 2-iodo-3,5-dimethylbenzenesulfonyl chloride **S-14** (469 mg, 1.42 mmol, 1.0 equiv.), isopropyl amine (150 µL, 1.70 mmol, 1.2 equiv.) and NEt_3 (300 µL, 2.84 mmol, 2.0 equiv.) in CH_2Cl_2 (5 mL). Purification by flash

column chromatography (EtOAc/pentane, *v/v* = 3:7) afforded sulfonamide **S-24** as a colourless solid (473 mg, 1.34 mmol, 94%).

MP: 156-157 °C.

FT IR (neat) υ (cm^{-1}) = 3277, 3078, 2966, 2921, 1451, 1421, 1384, 1314, 1301, 1134, 1120, 1025, 1004, 907, 867, 698, 608, 578.

^{1}H NMR (600 MHz, $CDCl_3$, 300 K): δ_H (ppm) = 7.88 (s, 1H), 7.24 (s, 1H), 5.23 (d, *J* = 7.2 Hz, 1H), 3.45 – 3.37 (m, 1H), 2.53 (s, 3H), 2.35 (s, 3H), 1.11 (d, *J* = 6.6 Hz, 6H).

^{13}C NMR (151 MHz, $CDCl_3$, 300 K): δ_C (ppm) = 144.6 (C_q), 143.1 (C_q), 138.4 (C_q), 134.0 (*C*H), 129.6 (*C*H), 95.1 (C_q), 46.5 (*C*H), 29.9 (CH_3), 23.5 (CH_3), 20.7 (CH_3).

HRMS (ESI) *m/z* = 375.9839 calcd. for $[C_{11}H_{16}NO_2SINa]^+$ $[M+Na]^+$, found: 375.9837.

2-Iodo-*N*-isopropyl-4-methylbenzenesulfonamide (S-25):

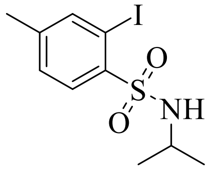

The title compound was prepared following a two-step procedure. At first, *p*-toluenesulfonyl chloride (762 mg, 4.00 mmol, 1.0 equiv.) was transformed to *N*-isopropyl-4-methylbenzenesulfonamide following general procedure **GP3** using isopropylamine (410 µL, 4.80 mmol, 1.2 equiv.) and NEt_3 (1.11 mL, 8.00 mmol, 2.0 equiv.) in CH_2Cl_2 (5 mL) and filtration over a short plug of silica (eluent: EtOAc) in quantitative yield. The intermediate was then subjected to sequential treatment with *n*-BuLi (1.6 M-solution in hexanes, 5.25 mL, 8.40 mmol, 2.1 equiv.) and iodine (1.12 g, 4.40 mmol, 1.1 equiv.) in THF (14 mL) according to general procedure **GP4**. Purification by flash column chromatography (Et_2O/Pentane, *v/v* = 1:19) afforded sulfonamide **S-25** as a colourless solid (1.49 g, 3.79 mmol, 95%).

MP: 96-97 °C.

FT IR (neat) υ (cm^{-1}) = 3302, 2973, 1587, 1459, 1410, 1387, 1332, 1302, 1263, 1157, 1138, 1103, 1028, 995, 890, 823, 734, 664, 649, 579.

^{1}H NMR (300 MHz, $CDCl_3$, 300 K): δ_H (ppm) = 8.05 (d, *J* = 8.0 Hz, 1H), 7.89 (s, 1H), 7.28 (ddd, *J* = 8.0, 1.7, 0.8 Hz, 1H), 5.07 (d, *J* = 7.4 Hz, 1H), 3.46 – 3.31 (m, 1H), 2.37 (s, 3H), 1.10 (d, *J* = 6.5 Hz, 6H).

^{13}C NMR (75 MHz, $CDCl_3$, 300 K): δ_C (ppm) = 144.3 (C_q), 142.7 (*C*H), 140.0 (C_q), 130.9 (*C*H), 129.2 (*C*H), 92.4 (C_q), 46.5 (*C*H), 23.5 (CH_3), 20.7 (CH_3).

HRMS (ESI) *m/z* = 361.9682 calcd. for $[C_{10}H_{14}NO_2SINa]^+$ $[M+Na]^+$, found: 361.9682.

2-Iodo-*N*-isopropyl-4-(trifluoromethyl)benzenesulfonamide (S-26):

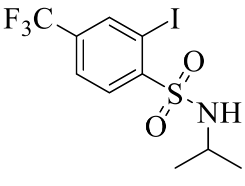

The title compound was prepared following a two-step procedure. At first, 4-(trifluoromethyl)benzenesulfonyl chloride (978 mg, 4.00 mmol, 1.0 equiv.) was transformed to *N*-isopropyl-4-(trifluoromethyl)benzene-sulfonamide following general procedure **GP3** using isopropylamine (410 µL, 4.80 mmol, 1.2 equiv.) and NEt_3 (1.11 mL, 8.00 mmol, 2.0 equiv.) in CH_2Cl_2 (5 mL) and filtration over a short plug of silica (eluent: EtOAc) in quantitative yield. The intermediate was then subjected to sequential treatment with *n*-BuLi (1.6 M-solution in hexanes, 5.25 mL, 8.40 mmol, 2.1 equiv.) and iodine (1.12 g, 4.40 mmol, 1.1 equiv.) in THF (14 mL) according to general procedure **GP4**. Purification by reverse-phase MPLC (MeCN/water, gradient from 5% to 95%) afforded sulfonamide **S-26** as a colourless solid (576 mg, 1.47 mmol, 37%).

MP: 76-77 °C.

FT IR (neat) υ (cm^{-1}) = 3303, 2976, 1380, 1316, 1173, 1132, 1105, 1072, 1031, 1002, 894, 714, 634, 608, 561.

^{1}H NMR (600 MHz, $CDCl_3$, 300 K): δ_H (ppm) = 8.32 – 8.29 (m, 2H), 7.77 (ddd, *J* = 8.2, 1.9, 0.8 Hz, 1H), 5.13 (d, *J* = 7.6 Hz, 1H), 3.52 – 3.42 (m, 1H), 1.13 (d, *J* = 6.5 Hz, 6H).

^{13}C{^{19}F} NMR (151 MHz, $CDCl_3$, 300 K): δ_C (ppm) = 146.7 (C_q), 139.1 (*C*H), 134.7 (C_q), 131.1 (*C*H), 125.6 (*C*H), 122.1 (C_q), 92.5 (C_q), 46.8 (*C*H), 23.5 (CH_3).

^{19}F{^{1}H} NMR (470 MHz, $CDCl_3$, 300 K): δ_F (ppm) = −63.1.

HRMS (ESI) *m/z* = 415.9310 calcd. for $[C_{10}H_{11}NO_2SF_3INa]^+$ $[M+Na]^+$, found: 415.9394.

2-Iodo-*N*-methyl-3,5-bis(trifluoromethyl)benzenesulfonamide (S-27):

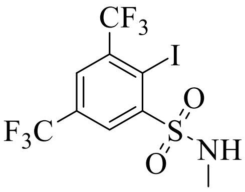

The title compound was prepared following a two-step procedure. At first, 3,5-Bis(trifluoromethyl)benzenesulfonyl chloride (531 mg, 1.70 mmol, 1.0 equiv.) was transformed to *N*-Methyl-3,5-bis(trifluoromethyl)-benzenesulfonamide following general procedure **GP3** using methylamine (2 M in THF, 1.02 mL, 2.04 mmol, 1.2 equiv.) and NEt_3 (471 µL, 3.40 mmol, 2.0 equiv.) in CH_2Cl_2 (5 mL) and filtration over a short plug of silica (eluent: EtOAc). The desired sulfonamide was isolated as a colourless solid (471 mg, 1.53 mmol, 90%). The intermediate was then subjected to sequential treatment with *n*-BuLi (1.6 M-solution in

hexanes, 1.83 mL, 2.94 mmol, 2.1 equiv.) and iodine (390 mg, 1.54 mmol, 1.1 equiv.) in THF (3.0 mL) according to general procedure **GP4**. Purification by reverse-phase MPLC (MeCN/water, gradient from 5% to 95%) afforded sulfonamide **S-27** as a colourless solid (92.3 mg, 0.200 mmol, 14%).

MP: 143-145 °C.

FT IR (neat) υ (cm^{-1}) = 3325, 2962, 1612, 1333, 1283, 1263, 1206, 1095, 904, 729, 650, 586.

^{1}H NMR (600 MHz, $CDCl_3$, 300 K): δ_H (ppm) = 8.64 (d, J = 2.2 Hz, 1H), 8.04 (d, J = 2.2 Hz, 1H), 5.38 (d, J = 5.4 Hz, 1H), 2.71 (d, J = 5.4 Hz, 3H).

^{13}C{^{19}F} NMR (151 MHz, $CDCl_3$, 300 K): δ_C (ppm) = 146.2 (C_q), 138.1 (C_q), 131.6 (C_q), 130.9 (*C*H), 127.5 (*C*H), 122.5 (C_q), 122.1 (C_q), 95.5 (C_q), 29.3 (CH_3).

^{19}F{^{1}H} NMR (470 MHz, $CDCl_3$, 300 K): δ_F (ppm) = −62.0, −63.2.

HRMS (ESI) *m/z* = 455.8960 calcd. for $[C_9H_6NO_2SF_6INa]^+$ $[M+Na]^+$, found: 455.8961.

5-(Dimethylamino)-2-iodo-*N*-isopropylnaphthalene-1-sulfonamide (S-28):

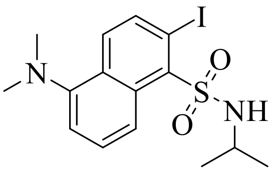

The title compound was prepared following a two-step procedure. At first, dansyl chloride (1.00 g, 3.71 mmol, 1.0 equiv.) was transformed to 5-(Dimethylamino)-*N*-isopropylnaphthalene-1-sulfonamide following general procedure **GP3** using isopropylamine (364 µL, 4.45 mmol, 1.2 equiv.) and NEt_3 (1.03 mL, 7.41 mmol, 2.0 equiv.) in CH_2Cl_2 (5 mL) and filtration over a short plug of silica (eluent: EtOAc) in quantitative yield. The intermediate was then subjected to sequential treatment with *n*-BuLi (1.6 M-solution in hexanes, 4.90 mL, 7.79 mmol, 2.1 equiv.) and iodine (1.04 g, 4.08 mmol, 1.1 equiv.) in THF (13 mL) according to general procedure **GP4**. Purification by reverse-phase MPLC (MeOH/water, gradient from 20% to 90%) afforded the title compound as a yellow oil (155 mg, 0.370 mmol, 10%).

FT IR (neat) υ (cm^{-1}) = 3325, 2973, 2938, 2835, 2791, 1575, 1454, 1398, 1339, 1265, 1139, 1046, 907, 800, 748, 703, 630, 590.

^{1}H NMR (300 MHz, $CDCl_3$, 300 K): δ_H (ppm) = 8.85 (dt, J = 9.0, 0.9 Hz, 1H), 8.16 – 8.09 (m, 2H), 7.50 (dd, J = 9.0, 7.5 Hz, 1H), 7.16 (dd, J = 7.6, 0.9 Hz, 1H), 5.42 (d, J = 7.5 Hz, 1H), 3.59 – 3.48 (m, 1H), 2.85 (s, 6H), 1.04 (d, J = 6.6 Hz, 6H).

^{13}C NMR (75 MHz, $CDCl_3$, 300 K): δ_C (ppm) = 151.7 (C_q), 139.3 (C_q), 138.3 (CH), 133.5 (C_q), 129.9 (CH), 129.5 (C_q), 128.5 (CH), 120.1 (CH), 115.3 (CH), 96.7 (C_q), 46.4 (CH), 45.5 (CH_3), 23.5 (CH_3).

HRMS (ESI) *m/z* = 441.0104 calcd. for $[C_{15}H_{19}N_2O_2SINa]^+$ $[M+Na]^+$, found: 441.0099.

3-Iodo-*N*-isopropyl-4-(*N*-isopropylsulfamoyl)benzamide (S-29):

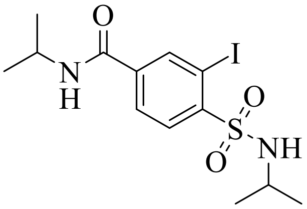

The title compound was prepared following a two-step procedure. At first, 4-(Chlorosulfonyl)benzoic acid (3.00 g, 13.6 mmol, 1.0 equiv.) was transformed to 4-(*N*-isopropylsulfamoyl)benzoic acid following general procedure **GP3** using isopropylamine (1.34 mL, 16.3 mmol, 1.2 equiv.) and NEt_3 (5.70 mL, 40.8 mmol, 2.0 equiv.) in CH_2Cl_2 (10 mL) and filtration over a short plug of silica (eluent: EtOAc). The desired sulfonamide was isolated as an orange solid (312 mg, 1.28 mmol, 9%). The intermediate was then subjected to sequential treatment with *sec*-BuLi (1.7 M-solution in heptanes, 2.34 mL, 3.98 mmol, 3.1 equiv.) and iodine (357 mg, 1.41 mmol, 1.1 equiv.) in THF (5 mL) according to general procedure **GP4**. Purification by reverse-phase MPLC (MeCN/water, gradient from 5% to 90%) afforded the title compound as a colourless solid (109 mg, 0.294 mmol, 23%).

MP: 128-131 °C.

FT IR (neat) υ (cm^{-1}) = 3392, 2976, 2217, 1641, 1454, 1329, 1164, 1140, 1095, 1036, 1008, 894, 757, 657, 606.

^{1}H NMR (600 MHz, $CDCl_3$, 300 K): δ_H (ppm) = 8.27 (d, J = 1.7 Hz, 1H), 7.78 (dd, J = 8.0, 1.7 Hz, 1H), 7.42 (d, J = 8.0 Hz, 1H), 6.00 (d, J = 8.0 Hz, 1H), 4.93 (d, J = 7.5 Hz, 1H), 4.30 – 4.24 (m, 1H), 3.47 – 3.41 (m, 1H), 1.30 (d, J = 6.6 Hz, 6H), 1.10 (d, J = 6.6 Hz, 6H).

^{13}C NMR (151 MHz, $CDCl_3$, 300 K): δ_C (ppm) = 167.5 (C_q), 146.1 (C_q), 143.2 (C_q), 137.6 (CH), 128.5 (CH), 126.5 (CH), 92.6 (C_q), 46.4 (CH), 42.5 (CH), 23.8 (CH_3), 22.5 (CH_3).

HRMS (ESI) *m/z* = 367.9448 calcd. for $[C_{10}H_{11}NO_4SI]^+$ $[M+Na]^+$, found: 367.9460.

2-Iodo-*N*-isopropylnaphthalene-1-sulfonamide (S-30):

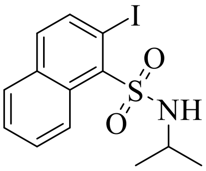

The title compound was prepared following a two-step procedure. At first, naphthalene-1-sulfonyl chloride (1.01 g, 4.45 mmol, 1.0 equiv.) was transformed to *N*-isopropylnaphthalene-1-sulfonamide following general procedure **GP3** using isopropylamine (0.460 mL, 5.34 mmol, 1.2 equiv.) and

NEt_3 (1.23 mL, 8.90 mmol, 2.0 equiv.) in CH_2Cl_2 (5 mL) and filtration over a short plug of silica (eluent: EtOAc) in quantitative yield. The intermediate was then subjected to sequential treatment with *n*-BuLi (1.6 M-solution in hexanes, 5.90 mL, 9.35 mmol, 2.1 equiv.) and iodine (1.24 g, 4.90 mmol, 1.10 equiv.) in THF (16 mL) according to general procedure **GP4**. Purification by flash column chromatography (EtOAc/pentane, *v/v* = 1:9) afforded sulfonamide **S-30** as a slightly yellow solid (1.04 g, 2.78 mmol, 62%).

MP: 132-133 °C.

FT IR (neat) υ (cm^{-1}) = 3309, 2972, 1584, 1421, 1339, 1162, 1133, 1003, 815, 771, 677, 595.

^{1}H NMR (300 MHz, $CDCl_3$, 300 K): δ_H (ppm) = 9.30 – 9.27 (m, 1H), 8.17 (d, *J* = 8.6 Hz, 1H), 7.87 – 7.84 (m, 1H), 7.67 – 7.56 (m, 3H), 5.41 (d, *J* = 7.6 Hz, 1H), 3.62 – 3.46 (m, 1H), 1.05 (d, *J* = 6.5 Hz, 6H).

^{13}C NMR (75 MHz, $CDCl_3$, 300 K): δ_C (ppm) = 139.4 (C_q), 139.2 (*C*H), 133.7 (C_q), 133.6 (*C*H), 131.9 (C_q), 128.8 (*C*H), 128.6 (*C*H), 127.1 (*C*H), 125.7 (*C*H), 96.6 (C_q), 46.5 (*C*H), 23.5 (CH_3).

HRMS (ESI) *m/z* = 397.9682 calcd. for $[C_{13}H_{14}NO_2SINa]^+$ $[M+Na]^+$, found: 397.9680.

3-Bromo-*N*-isopropylthiophene-2-sulfonamide (S-31):

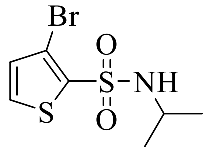

Following a procedure by *Barange, Tu et al.*[9] 3-bromothiophene (500 µL, 5.33 mmol, 1.00 equiv.) was dissolved in anhydrous CH_2Cl_2 (2 mL) at −78 °C. Chlorosulfuric acid (185 µL, 31.9 mmol, 5.98 equiv.) was added over 3 h at this temperature and stirring was continued overnight whilst the reaction mixture was allowed to slowly reach room temperature. The reaction was quenched by addition to ice and the crude 3-bromothiophene-2-sulfonyl chloride was obtained after extraction of the aqueous phase with CH_2Cl_2 (3 x 20 mL), drying over $MgSO_4$ and concentration *in vacuo*. The crude sulfonyl chloride was then treated with isopropylamine (550 µL, 6.40 mmol, 1.20 equiv.) and NEt_3 (1.50 mL, 10.7 mmol, 2.00 equiv.) in CH_2Cl_2 (5 mL) in accordance with general procedure **GP3**. Purification by flash column chromatography (EtOAc/Pentane, *v/*v = 3:7) afforded the title compound **S-31** as a colourless solid (871 mg, 3.07 mmol, 58%).

MP: 86-87 °C.

FT IR (neat) υ (cm^{-1}) = 3290, 2978, 1489, 1423, 1158, 1138, 1091, 1044, 875, 733, 677, 620, 594, 559.

^{1}H NMR (300 MHz, $CDCl_3$, 300 K): δ_H (ppm) = 7.50 (d, *J* = 5.3 Hz, 1H), 7.12 (d, *J* = 5.3 Hz, 1H), 4.86 (d, *J* = 7.5 Hz, 1H), 3.64 – 3.48 (m, 1H), 1.16 (d, *J* = 6.5 Hz, 6H).

^{13}C NMR (75 MHz, $CDCl_3$, 300 K): δ_C (ppm) = 137.2 (C_q), 132.5 (*C*H), 130.5 (*C*H), 112.8 (C_q), 46.8 (*C*H), 23.5 (*C*H).

HRMS (ESI) *m/z* = 307.9207 calcd. for $[C_7H_{10}NO_2S_2BrNa]^+$ $[M+Na]^+$, found: 307.9201.

3-Iodo-*N*-isopropylthiophene-2-sulfonamide (S-32):

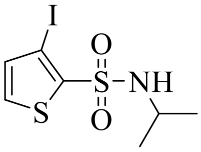

The title compound was prepared following general procedure **GP4** with 3-bromothiophene-2-sulfonamide **S-31** (568 mg, 2.00 mmol, 1.0 equiv.), *n*-BuLi (1.6 M-solution in hexanes, 2.50 mL, 4.00 mmol, 2.0 equiv.) and iodine (609 mg, 2.40 mmol, 1.2 equiv.) in THF (11 mL). Purification by reverse-phase MPLC (MeCN/water, gradient from 5% to 90%) afforded the title compound **S-32** as a colourless solid (262 mg, 0.791 mmol, 40%).

MP: 87-88 °C.

FT IR (neat) υ (cm^{-1}) = 3293, 2976, 1478, 1384, 1340, 1302, 1158, 1135, 1037, 997, 865, 730, 674, 589, 558.

^{1}H NMR (300 MHz, $CDCl_3$, 300 K): δ_H (ppm) = 7.46 (d, *J* = 5.2 Hz, 1H), 7.22 (d, *J* = 5.1 Hz, 1H), 4.94 (d, *J* = 7.6 Hz, 1H), 3.59 – 3.51 (m, 1H), 1.16 (d, *J* = 6.5 Hz, 7H).

^{13}C NMR (75 MHz, $CDCl_3$, 300 K): δ_C (ppm) = 140.8 (C_q), 138.1 (*C*H), 132.0 (*C*H), 82.6 (C_q), 46.8 (*C*H), 23.5 (CH_3).

HRMS (ESI) *m/z* = 353.9090 calcd. for $[C_7H_{10}NO_2S_2INa]^+$ $[M+Na]^+$, found: 353.9089.

2-(Benzylthio)-3-iodopyridine (S-33):

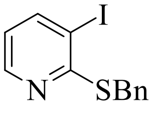

The title compound was prepared following a procedure by *Kehne et al.*[10] 2-Fluoro-3-iodopyridine (2.50 g, 11.2 mmol, 1.0 equiv.), benzylmercaptane (1.50 mL, 11.2 mmol, 1.0 equiv.) and K_2CO_3 (1.71 g, 12.3 mmol, 1.1 equiv.) were dissolved in anhydrous MeCN (15 mL) and refluxed for 18 h at 90 °C. The reaction mixture was allowed to cool to room temperature and the solvent was removed *in vacuo*. The residue was taken up in water (20 mL) and CH_2Cl_2 (20 mL). The phases were separated and the aqueous phase was extracted with CH_2Cl_2 (3 x 20 mL). The combined organic phases were washed with brine, dried over $MgSO_4$ and concentrated *in vacuo*. Purification by flash column

chromatography (Et_2O/Pentane, *v/*v = 1:9) afforded the title compound **S-3p** as a colourless solid (2.68 g, 8.21 mmol, 73%).

^{1}H NMR (300 MHz, $CDCl_3$, 300 K): δ_H (ppm) = 8.40 (dd, J = 4.7, 1.6 Hz, 1H), 7.90 (dd, J = 7.7, 1.6 Hz, 1H), 7.43 – 7.40 (m, 2H), 7.31 – 7.19 (m, 3H), 6.70 (dd, J = 7.8, 4.7 Hz, 1H), 4.39 (s, 2H).

^{13}C NMR (75 MHz, $CDCl_3$, 300 K): δ_C (ppm) = 161.6 (C_q), 148.1 (*C*H), 145.9 (*C*H), 137.4 (C_q), 129.3 (*C*H), 128.5 (*C*H), 127.2 (*C*H), 120.3 (*C*H), 93.1 (C_q), 37.2 (CH_2).

HRMS (ESI) *m/z* = 327.9651 calcd. for $[C_{12}H_{10}NSIH]^+$ $[M+H]^+$, found: 327.9652.

The analytical data are consistent with those reported in literature.[11]

3-Iodo-*N*-isopropylpyridine-2-sulfonamide (S-34):

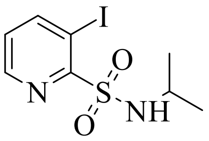

Following a procedure by *Friese et al.*[12] 2-(benzylthio)-3-iodopyridine **S-34** (982 mg, 3.00 mmol, 1.0 equiv.) was dissolved in CH_2Cl_2 (9.8 mL) and subsequently treated dropwise with a diluted aqueous NaOCl-solution (6.30 mL of aqueous 30w%-solution diluted with additional 6.30 mL water) and HCl (conc., 1.5 mL) at 0 °C. The solution was stirred for 30 minutes at 0 °C and then extracted with CH_2Cl_2 (3 x 15 mL). The combined organic phases were washed with $NHCO_3$-solution (sat. aq., 30 mL) and sat. aq. $Na_2S_2O_3$ (sat. aq., 30 mL), dried over $MgSO_4$ and concentrated *in vacuo*. The crude sulfonyl chloride was then treated with isopropylamine (310 µL, 3.60 mmol, 1.2 equiv.) and NEt_3 (830 µL, 6.00 mmol, 2.0 equiv.) in CH_2Cl_2 (5 mL) in accordance with general procedure **GP3**. Purification by flash column chromatography (EtOAc/Pentane, *v/v* = 3:7) afforded sulfonamide **S-35** as a colourless solid (232 mg, 2.13 mmol, 71%).

MP: 96-97 °C.

FT IR (neat) υ (cm^{-1}) = 3424, 2973, 1658, 1508, 1491, 1368, 1711, 1081, 1009, 906, 729, 700, 647, 578.

^{1}H NMR (600 MHz, $CDCl_3$, 300 K): δ_H (ppm) = 8.59 – 8.58 (m, 1H), 8.39 (ddd, J = 7.9, 1.4, 0.6 Hz, 1H), 7.16 (ddd, J = 7.9, 4.6, 0.6 Hz, 1H), 4.87 (d, J = 6.5 Hz, 1H), 3.67 – 3.59 (m, 1H), 1.22 (d, J = 6.5 Hz, 5H).

^{13}C NMR (151 MHz, $CDCl_3$, 300 K): δ_C (ppm) = 158.7 (C_q), 150.5 (*C*H), 147.3 (*C*H), 126.7 (*C*H), 88.3 (C_q), 47.4 (*C*H), 23.7 (CH_3).

HRMS (ESI) *m/z* = 348.9478 calcd. for $[C_8H_{11}N_2O_2SINa]^+$ $[M+Na]^+$, found: 348.9478.

2-Iodo-*N*-benzylbenzenesulfonamide (S-35):

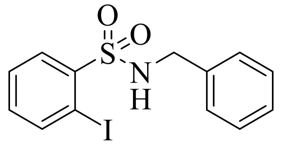

The title compound was prepared following general procedure **GP3** using benzylamine (0.23 mL, 2.1 mmol, 1.2 equiv.), 2-iodobenzenesulfonyl chloride **S-8** (0.54 g, 1.8 mmol, 1.0 equiv.) and NEt_3 (0.50 mL, 3.6 mmol, 2.0 equiv.) in CH_2Cl_2 (2 mL). Purification by flash column chromatography (EtOAc/Pentane, *v/v* = 1:4) afforded sulfonamide **S-36** as a colourless solid (505 mg, 1.35 mmol, 76%).

MP: 99-100 °C.

FT IR (neat) υ (cm^{-1}) = 334, 1569, 1496, 1421, 1331, 1161, 1014, 758, 724, 698, 604, 581.

^{1}H NMR (300 MHz, $CDCl_3$, 300 K): δ_H (ppm) = 8.18 (dd, *J* = 7.9, 1.7 Hz, 1H), 8.05 (dd, *J* = 7.9, 1.2 Hz, 1H), 7.50 (dt, *J* = 7.7, 1.2 Hz, 1H), 7.27 – 7.21 (m, 6H), 5.51 (t, *J* = 6.2 Hz, 1H), 4.09 (d, *J* = 6.2 Hz, 2H).

^{13}C NMR (75 MHz, $CDCl_3$, 300 K): δ_C (ppm) = 142.2 (*C*H), 142.0 (C_q), 135.7 (C_q), 133.4 (*C*H), 131.4 (*C*H), 128.7 (*C*H), 128.6 (*C*H), 128.1 (*C*H), 128.0 (*C*H), 92.3 (C_q), 47.5 (CH_2).

HRMS (ESI) *m/z* = 395.9526 calcd. for $[C_{13}H_{12}NO_2SINa]^+$ $[M+Na]^+$, found: 395.9525.

2-Iodo-*N*-cyclohexylbenzenesulfonamide (S-36):

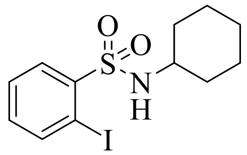

The title compound was prepared following general procedure **GP3** using cyclohexylamine (0.42 mL, 3.6 mmol, 1.2 equiv.), 2-iodobenzene-sulfonyl chloride **S-8** (908 mg, 3.00 mmol, 1.0 equiv.) and NEt_3 (0.84 mL, 6.0 mmol, 2.0 equiv.) in CH_2Cl_2 (5 mL). Purification by flash column chromatography (EtOAc/Pentane, *v/v* = 1:9) afforded sulfonamide **S-37** as a colourless solid (1.10 g, 3.00 mmol, quant.).

MP: 120-121 °C.

FT IR (neat) υ (cm^{-1}) = 3314, 2931, 1450, 1424, 1330, 1161, 1122, 1015, 759, 726, 604.

^{1}H NMR (300 MHz, $CDCl_3$, 300 K): δ_H (ppm) = 8.19 (dd, *J* = 7.9, 1.7 Hz, 1H), 8.05 (dd, *J* = 7.9, 1.2 Hz, 1H), 7.50 (td, *J* = 7.6, 1.2 Hz, 1H), 7.20 (td, *J* = 7.6, 1.7 Hz, 1H), 5.22 (d, *J* = 7.6 Hz, 1H), 3.17 – 3.06 (m, 1H), 1.77 – 1.45 (m, 5H), 1.29 – 1.10 (m, 5H).

^{13}C NMR (75 MHz, $CDCl_3$, 300 K): δ_C (ppm) = 143.2 (C_q), 142.2 (*C*H), 133.2 (*C*H), 130.8 (*C*H), 128.6 (*C*H), 92.5 (C_q), 53.0 (*C*H), 33.6 (CH_2), 25.1 (CH_2), 24.5 (CH_2).

HRMS (ESI) *m/z* = 387.9839 calcd. for $[C_{12}H_{16}NO_2SINa]^+$ $[M+Na]^+$, found: 387.9837.

(*S*)-2-Iodo-*N*-(1-phenylethyl)benzenesulfonamide (S-37):

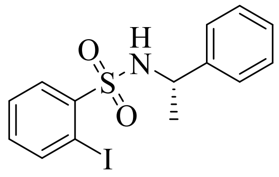

The title compound was prepared following general procedure **GP3** using (*S*)-phenylethylamine (0.39 mL, 3.1 mmol, 1.2 equiv.), 2-iodobenzenesulfonyl chloride **S-8** (771 mg, 2.55 mmol, 1.0 equiv.) and NEt_3 (0.70 mL, 5.0 mmol, 2.0 equiv.) in CH_2Cl_2 (4 mL). Purification by flash column chromatography (Pentane/CH_2Cl_2, *v/v* = 1:9) afforded sulfonamide **S-38** as a colourless solid (985 mg, 2.55 mmol, 99%).

MP: 131-132 °C.

FT IR (neat) υ (cm^{-1}) = 3303, 1570, 1447, 1426, 1333, 1164, 1117, 1084, 1014, 958, 867, 758, 728, 699, 579.

^{1}H NMR (300 MHz, $CDCl_3$, 300 K): δ_H (ppm) = 8.01 – 7.91 (m, 2H), 7.36 (td, *J* = 7.7, 1.2 Hz, 1H), 7.17 – 7.07 (m, 6H), 5.49 (d, *J* = 7.3 Hz, 1H), 4.50 – 4.41 (m, 1H), 1.48 (d, *J* = 6.9 Hz, 3H).

^{13}C NMR (75 MHz, $CDCl_3$, 300 K): δ_C (ppm) = 142.6 (C_q), 141.9 (*C*H), 141.0 (C_q), 132.9 (*C*H), 131.0 (*C*H), 128.5 (*C*H), 128.3 (*C*H), 127.7 (*C*H), 126.2 (*C*H), 92.5 (C_q), 54.3 (*C*H), 23.0 (CH_3).

HRMS (ESI) *m/z* = 409.9682 calcd. for $[C_{14}H_{14}INNaO_2S]^+$ $[M+Na]^+$, found: 409.9676.

6.4 Synthesis of *N*-acylated 2-iodoarylsulfonamides

***N*-((2-Iodophenyl)sulfonyl)-*N*-isopropylisobutyramide (1a):**

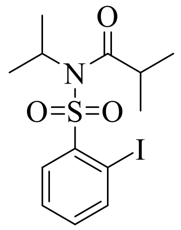

The title compound was prepared following general procedure **GP5** using sulfonamide **S-18** (0.36 g, 1.1 mmol, 1.0 equiv.), DMAP (1.4 mg, 11 µmol, 1 mol%), NEt_3 (0.39 mL, 2.8 mmol, 2.5 equiv.) and isobutyrylchloride (0.13 mL, 1.2 mmol, 1.1 equiv.) in isopropyl acetate (2.6 mL) and toluene (0.85 mL). Purification by flash column chromatography (Pentane/Et_2O, *v/v* = 1:1) afforded sulfonamide **1a** as a colourless solid (0.41 g, 1.1 mmol, 94%).

MP: 84-85 °C.

FT IR (neat) υ (cm^{-1}) = 2972, 2935, 2875, 1700, 1569, 1447, 1422, 1383, 1346, 1192, 1346, 1166, 1153, 1015, 972, 761, 729, 596, 575.

^{1}H NMR (300 MHz, $CDCl_3$, 300 K): δ_H (ppm) = 8.21 (dd, J = 8.0, 1.6 Hz, 1H), 8.14 (dd, J = 8.0, 1.2 Hz, 1H), 7.59 – 7.54 (m, 1H), 7.31 – 7.25 (m, 1H), 4.09 (hept, J = 6.8 Hz, 1H), 3.60 (hept, J = 6.7 Hz, 1H), 1.32 (d, J = 6.8 Hz, 6H), 1.18 (d, J = 6.7 Hz, 6H).

^{13}C NMR (75 MHz, $CDCl_3$, 300 K): δ_C (ppm) = 179.6 (C_q), 143.1 (CH), 141.6 (C_q), 134.1 (CH), 131.6 (CH), 128.5 (CH), 92.8 (C_q), 53.5 (CH), 36.9 (CH), 20.0 (CH_3), 19.4 (CH_3).

HRMS (ESI) *m/z* = 417.9944 calcd. for $[C_{13}H_{18}INNaO_3S]^+$ $[M+Na]^+$, found 417.9954.

***N*-((2-Iodophenyl)sulfonyl)-*N*-cyclohexylisobutyramide (1b):**

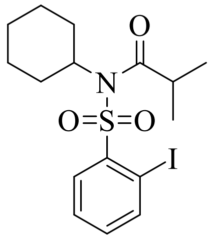

The title compound was prepared following general procedure **GP5** using sulfonamide **S-36** (219 mg, 0.600 mmol, 1.0 equiv.), DMAP (0.7 mg, 6 µmol, 1 mol%), NEt_3 (210 µL, 1.50 mmol, 2.5 equiv.) and isobutyrylchloride (69 µL, 0.66 mmol, 1.1 equiv.) in isopropyl acetate (3 mL) and toluene (1 mL). Purification by flash column chromatography (2.5% EtOAc in pentane) afforded sulfonamide **1b** as a colourless solid (235 mg, 0.540 mmol, 90%).

MP: 121-122 °C.

FT IR (neat) υ (cm^{-1}) = 2970, 2934, 2856, 1702, 1349, 1199, 1168, 1141, 1121, 1094, 1014, 982, 737, 592, 571.

^{1}H NMR (300 MHz, $CDCl_3$, 300 K): δ_H (ppm) = 8.20 (dd, J = 8.0, 1.6 Hz, 1H), 8.13 (dd, J = 7.8, 1.2 Hz, 1H), 7.56 (td, J = 7.8, 1.2 Hz, 1H), 7.30 – 7.24 (m, 1H), 3.73 – 3.62 (m, 1H), 3.60 – 3.51 (m, 1H), 2.24 – 2.12 (m, 2H), 1.73 – 1.56 (m, 6H), 1.17 (d, J = 6.7 Hz, 6H), 1.18 – 1.04 (m, 2H).

^{13}C NMR (75 MHz, $CDCl_3$, 300 K): δ_C (ppm) = 179.9 (C_q), 143.1 (CH), 141.8 (C_q), 134.1 (CH), 131.4 (CH), 128.4 (CH), 92.8 (C_q), 62.0 (CH), 37.1 (CH), 29.8 (CH_2), 26.7 (CH_2), 25.1 (CH_2), 19.5 (CH_3).

HRMS (ESI) *m/z* = 458.0257 calcd. for $[C_{16}H_{22}NO_3SINa]^+$ $[M+Na]^+$, found 458.0253.

(*S*)-*N*-((2-Iodophenyl)sulfonyl)-*N*-(1-phenylethyl)isobutyramide (1c):

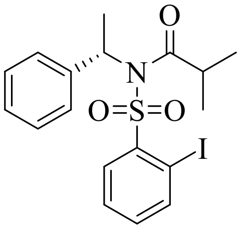

The title compound was prepared following general procedure **GP5** using sulfonamide **S-37** (0.25 g, 0.64 mmol, 1.0 equiv.), DMAP (0.8 mg, 6 µmol, 1 mol%), NEt_3 (0.22 mL, 1.6 mmol, 2.5 equiv.) and isobutyrylchloride (73 µL, 0.70 mmol, 1.1 equiv.) in isopropyl acetate (1.5 mL) and toluene (400 µL). Purification by flash column chromatography (Pentane/Et_2O, *v/v* = 7:3) afforded sulfonamide **1c** as a colourless solid (0.21 g, 0.45 mmol, 71%).

MP: 96-98 °C.

FT IR (neat) υ (cm^{-1}) = 2975, 2938, 2877, 1702, 1448, 1352, 1194, 1169, 1126, 959, 759, 726, 669, 612, 592, 560.

^{1}H NMR (300 MHz, $CDCl_3$, 300 K): δ_H (ppm) = 8.30 (dd, *J* = 8.0, 1.6 Hz, 1H), 8.14 (dd, *J* = 7.9, 1.2 Hz, 1H), 7.59 – 7.54 (m, 1H), 7.49 – 7.45 (m, 2H), 7.35 – 7.24 (m, 4H), 5.38 (q, *J* = 7.0 Hz, 1H), 3.36 (hept, *J* = 6.7 Hz, 1H), 1.67 (d, *J* = 7.0 Hz, 3H), 1.03 (d, *J* = 6.7 Hz, 3H), 0.91 (d, *J* = 6.7 Hz, 3H).

^{13}C NMR (75 MHz, $CDCl_3$, 300 K): δ_C (ppm) = 179.0 (C_q), 143.0 (*C*H), 141.7 (C_q), 139.7 (C_q), 134.2 (*C*H), 132.5 (*C*H), 128.6 (*C*H), 128.2 *C*H), 127.4 (*C*H), 127.1 (*C*H), 92.8 (C_q), 57.2 (*C*H), 36.7 (*C*H), 19.8 (CH_3), 18.2 (CH_3), 17.1 (CH_3).

HRMS (ESI) *m/z* = 480.0101 calcd. for $[C_{18}H_{20}INNaO_3S]^+$ $[M+Na]^+$, found 480.0103.

***N*-((2-Iodophenyl)sulfonyl)-*N*-methylisobutyramide (1d):**

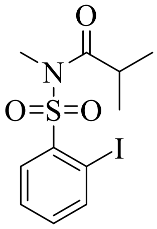

The title compound was prepared following general procedure **GP5** using sulfonamide **S-15** (0.15 g, 0.52 mmol, 1.0 equiv.), DMAP (0.6 mg, 5 µmol, 1 mol%), NEt_3 (0.18 mL, 1.3 mmol, 2.5 equiv.) and isobutyryl chloride (61 µL, 0.58 mmol, 1.1 equiv.) in isopropyl acetate (2.3 mL) and toluene (0.95 mL). Purification by flash column chromatography (Pentane/Et_2O, *v/v* = 1:1) afforded sulfonamide **1d** as a colourless solid (0.18 g, 0.50 mmol, 96%).

MP: 129-132 °C

FT IR (neat) υ (cm^{-1}) = 2974, 2939, 1701, 1569, 1449, 1389, 1345, 1236, 1170, 1058, 1014, 995, 794, 745, 617, 563.

^{1}H NMR (300 MHz, $CDCl_3$, 300 K): δ_H (ppm) = 8.32 (dd, J = 8.1, 1.6 Hz, 1H), 8.06 (dd, J = 7.9, 1.2 Hz, 1H), 7.60 – 7.54 (m, 1H), 7.28 – 7.23 (m, 1H), 3.48 (s, 3H), 3.17 – 3.03 (m, 1H), 1.12 (d, J = 6.7 Hz, 6H).

^{13}C NMR (75 MHz, $CDCl_3$, 300 K): δ_C (ppm) = 177.6 (C_q), 142.5 (CH), 142.2 (C_q), 134.1 (CH), 132.8 (CH), 128.6 (CH), 91.1 (C_q), 34.0 (CH_3), 33.6 (CH), 18.9 (CH_3).

HRMS (ESI) *m/z* = 389.9631 calcd. for $[C_{11}H_{14}INNaO_3S]^+$ $[M+Na]^+$, found 389.9639.

***N*-((2-Iodophenyl)sulfonyl)-*N*-benzylisobutyramide (1e):**

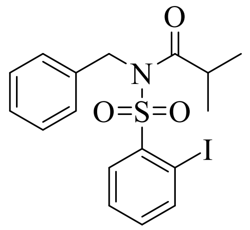

The title compound was prepared following general procedure **GP5** using sulfonamide **S-35** (187 mg, 0.500 mmol, 1.0 equiv.), DMAP (0.6 mg, 5 µmol, 1 mol%), NEt_3 (170 µL, 1.25 mmol, 2.5 equiv.) and isobutyrylchloride (58 µL, 0.55 mmol, 1.1 equiv.) in isopropyl acetate (3 mL) and toluene (1 mL). Purification by flash column chromatography (EtOAc/Pentane, *v/v* = 1:4) led to isolation of the desired *N*-acylated sulfonamide **1e** as a colourless oil (256 mg, 0.500 mmol, 99%).

FT IR (neat) υ (cm^{-1}) = 2975, 1935, 1700, 1344, 1169, 1117, 1089, 755, 590, 567.

^{1}H NMR (300 MHz, $CDCl_3$, 300 K): δ_H (ppm) = 8.25 (dd, J = 8.1, 1.6 Hz, 1H), 7.94 (dd, J = 7.9, 1.2 Hz, 1H), 7.46 (ddd, J = 7.9, 7.4, 1.2 Hz, 1H), 7.35 – 7.24 (m, 4H), 7.21 – 7.11 (m, 2H), 5.15 (s, 2H), 2.78 (hept, J = 6.7 Hz, 1H), 0.85 (d, J = 6.7 Hz, 6H).

^{13}C NMR (75 MHz, $CDCl_3$, 300 K): δ_C (ppm) = 177.8 (C_q), 142.5 (CH), 142.2 (C_q), 136.9 (C_q), 134.2 (CH), 133.2 (CH), 128.9 (CH), 128.6 (CH), 127.7 (CH), 126.8 (CH), 91.1 (C_q), 51.3 (CH_2), 34.0 (CH), 19.1 (CH_3).

HRMS (ESI) *m/z* = 465.9944 calcd. for $[C_{17}H_{18}NO_3SINa]^+$ $[M+Na]^+$, found 465.9935.

***N*-((2-Iodophenyl)sulfonyl)-*N*-phenylisobutyramide (1f):**

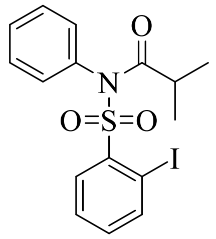

The title compound was prepared following a slightly modified procedure by *Li, Hu et al.*[15] Sulfonamide **S-16** (0.21 g, 0.59 mmol, 1.0 equiv.) and diisopropylethylamine (0.12 mL, 0.70 mmol, 1.2 equiv.) were dissolved in CH_2Cl_2 (1 mL) at room temperature. Isobutyryl chloride (74 µL, 0.70 mmol, 1.2 equiv.) was added dropwise over 3 h by syringe pump and the reaction mixture was stirred overnight. Removal of the solvent *in vacuo* and purfication by flash column

chromatography (Et_2O/Pentane, *v/v* = 3:7) afforded sulfonamide **1f** as a colourless solid (0.20 g, 0.47 mmol, 79%).

MP: 133-135 °C

FT IR (neat) υ (cm^{-1}) = 2977, 2876, 1703, 1488, 1450, 1236, 1174, 1140, 1122, 1093, 1014, 961, 824, 758, 734, 697, 582, 565.

^{1}H NMR (300 MHz, $CDCl_3$, 300 K): δ_H (ppm) = 8.38 (dd, *J* = 8.1, 1.7 Hz, 1H), 8.09 (dd, *J* = 7.9, 1.2 Hz, 1H), 7.66 – 7.63 (m, 2H), 7.58 – 7.48 (m, 4H), 7.25 – 7.22 (m, 1H), 2.59 (hept, *J* = 6.8 Hz, 1H), 1.04 (d, *J* = 6.8 Hz, 6H).

^{13}C NMR (75 MHz, $CDCl_3$, 300 K): δ_C (ppm) = 177.8 (C_q), 142.6 (*C*H), 142.3 (C_q), 135.0 (C_q), 134.1 (*C*H), 134.1 (*C*H), 130.9 (*C*H), 130.1 (*C*H), 129.6 (*C*H), 128.4 (*C*H), 91.6 (C_q), 33.9 (*C*H), 19.1 (CH_3).

HRMS (ESI) *m/z* = 451.9788 calcd. for $[C_{16}H_{16}INNaO_3S]^+$ $[M+Na]^+$, found 451.9797.

***N*-((2-Iodophenyl)sulfonyl)isobutyramide (1g):**

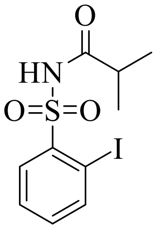

The title compound was prepared following a slightly modified procedure by *Anana et al.*[13] and *Hermann et al.*[14] To this end, 2-iodobenzenesulfonyl chloride (570 mg, 1.88 mmol, 1.0 equiv.) was suspended in aqueous ammonia (25 Vol%, 7 mL) and stirred overnight. The precipitate was filtered off, washed with water and pentane and dried *in vacuo*. The sulfonamide was obtained as a colourless crystalline solid (438 mg, 1.55 mmol, 82%), which was used for the next step without further purification. Subsequently, sodium hydride (60% dispersion in mineral oil, 56.6 mg, 1.42 mmol, 2.2 equiv.) was washed with pentane (3 x 5 mL) and then suspended in anhydrous THF (3.1 mL). After the slurry was cooled to 0 °C, a solution of 2-iodobenzenesulfonamide (182 mg, 0.64 mmol, 1.0 eq,) in anhydrous THF (0.43 mL) was added dropwise. After stirring for one hour the mixture was cooled to –78 °C and isobutryl chloride (74 µL, 0.71 mmol, 1.1 equiv.) was added slowly. The reaction mixture was warmed to room temperature overnight, quenched by addition of HCl (ca. 2 M aq., 10 mL) and extracted with CH_2Cl_2 (3 x 20 mL). The combined organic phases were dried over $MgSO_4$ and concentrated *in vacuo.* The crude residue was purifed by recrystallisation (cyclohexane/$CHCl_3$) and the title compound was obtained as a colourless crystalline solid (72 mg, 0.20 mmol, 32%).

MP: 154-155 °C.

FT IR (neat) υ (cm^{-1}) = 3250, 2975, 1722, 1701, 1428, 1339, 1188, 1172, 1085, 1015, 885, 828, 760, 726, 579, 563.

^{1}H NMR (300 MHz, $CDCl_3$, 300 K): δ_H (ppm) = 8.97 (s, 1H), 8.40 – 8.37 (m, 1H), 8.10 – 8.07 (m, 1H), 7.61 – 7.56 (m, 1H), 7.32 – 7.26 (m, 1H), 2.61 – 2.47 (m, 1H), 1.18 (d, *J* = 7.0 Hz, 6H)

^{13}C NMR (75 MHz, $CDCl_3$, 300 K): δ_C (ppm) = 174.3 (C_q), 142.4 (*C*H), 140.6 (C_q), 134.6 (*C*H), 133.3 (*C*H), 128.7 (*C*H), 92.0 (C_q), 35.9 (*C*H), 18.7 (CH_3).

HRMS (ESI) *m/z* = 375.9475 calcd. for $[C_{10}H_{12}NO_3SINa]^+$ $[M+Na]^+$, found 375.9473.

***N*-((2-Iodophenyl)sulfonyl)-*N*-methoxyisobutyramide (1h):**

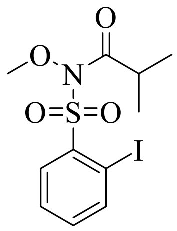

The title compound was prepared following general procedure **GP5** using sulfonamide **S-17** (626 mg, 2.00 mmol, 1.0 equiv.), DMAP (2.4 mg, 0.02 mmol, 1 mol%), NEt_3 (0.70 mL, 5.0 mmol, 2.5 equiv.) and isobutyrylchloride (230 µL, 2.20 mmol, 1.10 equiv.) in isopropyl acetate (4.7 mL) and toluene (1.3 mL). Purification by flash column chromatography (EtOAc/Pentane, *v/v* = 1:4) afforded sulfonamide **1h** as a colourless solid (600 mg, 1.57 mmol, 78%).

MP: 85-86 °C.

FT IR (neat) υ (cm^{-1}) = 2973, 2009, 1678, 1513, 1450, 1267, 1073, 1030, 771, 733, 701, 623, 583, 566.

^{1}H NMR (300 MHz, $CDCl_3$, 300 K): δ_H (ppm) = 8.26 (dd, *J* = 8.1, 1.6 Hz, 1H), 8.10 (dd, *J* = 7.9, 1.3 Hz, 1H), 7.55 (ddd, *J* = 8.1, 7.4, 1.3 Hz, 1H), 7.29 – 7.23 (m, 1H), 4.03 (s, 3H), 3.29 – 3.16 (m, 1H), 1.19 (d, *J* = 6.9 Hz, 6H).

^{13}C NMR (75 MHz, $CDCl_3$, 300 K): δ_C (ppm) = 176.6 (C_q), 142.9 (*C*H), 140.4 (C_q), 134.5 (*C*H), 133.4 (*C*H), 128.4 (*C*H), 91.6 (C_q), 66.6 (CH_3), 33.6 (*C*H), 18.8 (CH_3).

HRMS (ESI) *m/z* = 405.9580 calcd. for $[C_{11}H_{14}INO_4S]^+$ $[M+Na]^+$, found 405.9583.

***N*-((2-Iodo-5-methylphenyl)sulfonyl)-*N*-isopropylisobutyramide (1i):**

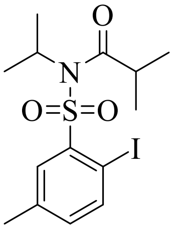

The title compound was prepared following general procedure **GP5** using sulfonamide **S-19** (0.24 g, 0.69 mmol, 1.0 equiv.), DMAP (0.9 mg, 7 µmol, 1 mol%), NEt_3 (0.24 mL, 1.8 mmol, 2.5 equiv.) and isobutyrylchloride (81 µL, 0.77 mmol, 1.1 equiv.) in isopropyl acetate (3.5 mL) and toluene (1.2 mL). Purification by flash column chromatography (Pentane/EtOAc, *v/v* = 9:1) afforded sulfonamide **S-4hb** as a colourless solid (0.25 g, 0.62 mmol, 89%).

MP: 101-102 °C.

FT IR (neat) υ (cm^{-1}) = 2976, 2940, 1700, 1559, 1448, 1420, 1437, 1191, 1160, 1105, 970, 739, 641, 598, 576.

^{1}H NMR (300 MHz, $CDCl_3$, 300 K): δ_H (ppm) = 8.03 – 7.96 (m, 2H), 7.09 (ddd, *J* = 8.0, 2.2, 0.8 Hz, 1H), 4.04 (hept, *J* = 6.8 Hz, 1H), 3.64 (hept, *J* = 6.7 Hz, 1H), 2.42 (s, 3H), 1.32 (d, *J* = 6.8 Hz, 6H), 1.19 (d, *J* = 6.7 Hz, 6H).

^{13}C NMR (75 MHz, $CDCl_3$, 300 K): δ_C (ppm) = 179.6 (C_q), 142.8 (*C*H), 141.1 (C_q), 139.2 (*C*H), 135.1 (*C*H), 132.2 (*C*H), 88.6 (C_q), 53.5 (*C*H), 36.9 (*C*H), 21.0 (CH_3), 20.0 (CH_3), 19.4 (CH_3).

HRMS (ESI) *m/z* = 432.0101 calcd. for $[C_{14}H_{20}NO_3SINa]^+$ $[M+Na]^+$, found 432.0094.

***N*-((2-Iodo-4-methylphenyl)sulfonyl)-*N*-isopropylisobutyramide (1j):**

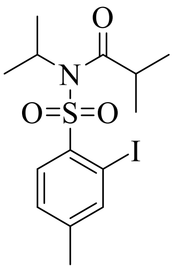

The title compound was prepared following general procedure **GP5** using sulfonamide **S-25** (0.34 g, 1.0 mmol, 1.0 equiv.), DMAP (1.2 mg, 10 µmol, 1 mol%), NEt_3 (0.35 mL, 2.5 mmol, 2.5 equiv.) and isobutyrylchloride (0.12 mL, 1.1 mmol, 1.1 equiv.) in isopropyl acetate (5.0 mL) and toluene (1.7 mL). Purification by flash column chromatography (Pentane/EtOAc, *v/v* = 19:1) afforded sulfonamide **1j** as a colourless solid (0.43 g, 0.94 mmol, 94%).

MP: 83-84 °C.

FT IR (neat) υ (cm^{-1}) = 2973, 2937, 1706, 1381, 1352, 1315, 1173, 1133, 1072, 972, 716, 610, 573.

^{1}H NMR (300 MHz, $CDCl_3$, 300 K): δ_H (ppm) = 8.07 (d, *J* = 8.2 Hz, 1H), 7.95 (s, 1H), 7.34 (ddd, *J* = 8.2, 1.8, 0.9 Hz, 1H), 4.07 (hept, *J* = 6.8 Hz, 1H), 3.62 (hept, *J* = 6.7 Hz, 1H), 2.40 (s, 3H), 1.32 (d, *J* = 6.8 Hz, 6H), 1.17 (d, *J* = 6.7 Hz, 6H).

^{13}C NMR (75 MHz, $CDCl_3$, 300 K): δ_C (ppm) = 179.6 (C_q), 145.4 (CH), 143.5 (CH), 138.7 (C_q), 131.5 (CH), 129.2 (CH), 92.8 (C_q), 53.4 (CH), 36.8 (CH), 20.8 (CH_3), 20.0 (CH_3), 19.4 (CH_3).

HRMS (ESI) *m/z* = 432.0101 calcd. for $[C_{14}H_{20}NO_3SINa]^+$ $[M+Na]^+$, found 432.0095.

***N*-((2-Iodo-5-methoxyphenyl)sulfonyl)-*N*-isopropylisobutyramide (1k):**

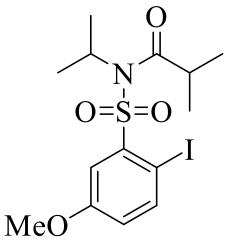

The title compound was prepared following general procedure **GP5** using sulfonamide **S-21** (0.25 g, 0.64 mmol, 1.0 equiv.), DMAP (0.9 mg, 7 µmol, 1 mol%), NEt_3 (0.22 mL, 1.6 mmol, 2.5 equiv.) and isobutyrylchloride (74 µL, 0.70 mmol, 1.1 equiv.) in isopropyl acetate (3.5 mL) and toluene (1.2 mL). Purification by flash column chromatography (Pentane/EtOAc, *v/v* = 9:1) afforded sulfonamide **1k** as a colourless solid (0.27 g, 0.63 mmol, 99%).

MP: 86-87 °C.

FT IR (neat) υ (cm^{-1}) = 2971, 2936, 1701, 1586, 164, 1436, 1292, 1232, 1195, 1165, 1152, 973, 829, 701, 618, 587, 554.

^{1}H NMR (300 MHz, $CDCl_3$, 300 K): δ_H (ppm) = 7.96 (d, *J* = 8.7 Hz, 1H), 7.75 (d, *J* = 3.0 Hz, 1H), 6.84 (dd, *J* = 8.6, 3.0 Hz, 1H), 4.12 – 3.98 (m, 1H), 3.88 (s, 3H), 3.63 (hept, *J* = 6.8 Hz, 1H), 1.33 (d, *J* = 6.8 Hz, 6H), 1.19 (d, *J* = 6.7 Hz, 6H).

^{13}C NMR (75 MHz, $CDCl_3$, 300 K): δ_C (ppm) = 179.5 (C_q), 159.9 (CH), 143.6 (C_q), 142.2 (CH), 120.4 (CH), 117.4 (CH), 80.6 (C_q), 55.9 (CH_3), 53.6 (CH), 36.9 (CH), 20.0 (CH_3), 19.4 (CH_3).

HRMS (ESI) *m/z* = 448.0050 calcd. for $[C_{14}H_{20}NO_4SINa]^+$ $[M+Na]^+$, found 448.0046.

***N*-((5-Chloro-2-iodophenyl)sulfonyl)-*N*-isopropylisobutyramide (1l):**

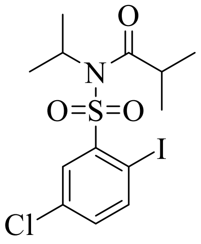

The title compound was prepared following general procedure **GP5** using sulfonamide **S-20** (0.25 g, 0.68 mmol, 1.0 equiv.), DMAP (0.9 mg, 7 µmol, 1 mol%), NEt_3 (0.24 mL, 1.7 mmol, 2.5 equiv.) and isobutyrylchloride (79 µL, 0.75 mmol, 1.1 equiv.) in isopropyl acetate (3.5 mL) and toluene (1.2 mL). Purification by flash column chromatography (Pentane/EtOAc, *v/v* = 9:1) afforded sulfonamide **1l** as a colourless solid (0.27 g, 0.64 mmol, 93%).

MP: 143-144 °C.

FT IR (neat) υ (cm^{-1}) = 2975, 2935, 1705, 1367, 1347, 1195, 1169, 1150, 1107, 1084, 1013, 973, 830, 604, 582.

^{1}H NMR (300 MHz, $CDCl_3$, 300 K): δ_H (ppm) = 8.18 (d, J = 2.5 Hz, 1H), 8.03 (d, J = 8.4 Hz, 1H), 7.28 – 7.24 (m, 1H), 4.06 (hept, J = 6.9 Hz, 1H), 3.57 (hept, J = 6.7 Hz, 1H), 1.34 (d, J = 6.9 Hz, 6H), 1.20 (d, J = 6.7 Hz, 6H).

^{13}C NMR (75 MHz, $CDCl_3$, 300 K): δ_C (ppm) = 179.4 (C_q), 144.0 (*C*H), 143.1 (C_q), 135.4 (C_q), 134.2 (*C*H), 131.5 (*C*H), 89.8 (C_q), 53.7 (*C*H), 37.1 (*C*H), 20.1 (CH_3), 19.4 (CH_3).

HRMS (ESI) *m/z* = 451.9555 calcd. for $[C_{13}H_{17}NO_3SClINa]^+$ $[M+Na]^+$, found 451.9551.

***N*-((2-Iodo-4-(trifluoromethyl)phenyl)sulfonyl)-*N*-isopropylisobutyramide (1m):**

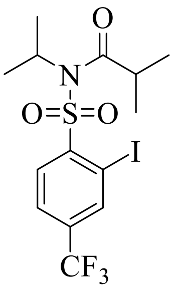

The title compound was prepared following general procedure **GP5** using sulfonamide **S-26** (0.39 g, 1.0 mmol, 1.0 equiv.), DMAP (1.2 mg, 10 µmol, 1 mol%), NEt_3 (0.35 mL, 2.5 mmol, 2.5 equiv.) and isobutyrylchloride (0.12 mL, 1.1 mmol, 1.1 equiv.) in isopropyl acetate (5.0 mL) and toluene (1.7 mL). Purification by flash column chromatography (Pentane/EtOAc, *v/v* = 19:1) afforded sulfonamide **1m** as a colourless oil (0.23 g, 0.55 mmol, 55%).

FT IR (neat) υ (cm^{-1}) = 2973, 2934, 1701, 1347, 1195, 1167, 1155, 974, 652, 590, 552.

^{1}H NMR (600 MHz, $CDCl_3$, 300 K): δ_H (ppm) = 8.34 – 8.32 (m, 1H), 8.31 (m, 1H), 7.83 – 7.81 (m, 1H), 4.13 (hept, J = 6.8 Hz, 1H), 3.54 – 3.46 (m, 1H), 1.36 (d, J = 6.8 Hz, 6H), 1.19 (d, J = 6.8 Hz, 6H).

^{13}C{^{19}F} NMR (151 MHz, $CDCl_3$, 300 K): δ_C (ppm) = 179.4 (C_q), 145.2 (C_q), 139.9 (*C*H), 135.4 (C_q), 131.8 (*C*H), 125.5 (*C*H), 121.9 (C_q), 92.8 (C_q), 53.6 (*C*H), 37.0 (*C*H), 20.3 (CH_3), 19.4 (CH_3).

^{19}F{^{1}H} NMR (470 MHz, $CDCl_3$, 300 K): δ_F (ppm) = −63.2.

HRMS (ESI) *m/z* = 485.9818 calcd. for $[C_{14}H_{17}NO_3SF_3INa]^+$ $[M+Na]^+$, found 485.9813.

***N*-((5-Chloro-2-iodo-4-methylphenyl)sulfonyl)-*N*-isopropylisobutyramide (1n):**

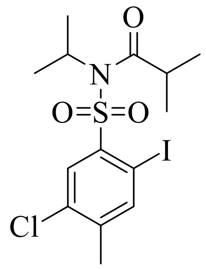

The title compound was prepared following general procedure **GP5** using sulfonamide **S-22** (0.26 g, 0.7 mmol, 1.0 equiv.), DMAP (0.9 mg, 7 µmol, 1 mol%), NEt_3 (0.24 mL, 1.8 mmol, 2.5 equiv.) and isobutyrylchloride (81 µL, 0.77 mmol, 1.1 equiv.) in isopropyl acetate (3.5 mL) and toluene (1.2 mL). Purification by flash column chromatography (Pentane/EtOAc, *v/v* = 9:1) afforded sulfonamide **1n** as a colourless solid (0.30 g, 0.68 mmol, 97%).

MP: 125-126 °C.

FT IR (neat) υ (cm^{-1}) = 2972, 2936, 1703, 1451, 1350, 1195, 1168, 1152, 1064, 975, 894, 714, 634, 614, 596, 564.

^{1}H NMR (300 MHz, $CDCl_3$, 300 K): δ_H (ppm) = 8.07 (s, 1H), 8.06 (s, 1H), 4.04 (hept, J = 6.8 Hz, 1H), 3.60 (hept, J = 6.8 Hz, 1H), 2.43 (s, 3H), 1.33 (d, J = 6.8 Hz, 6H), 1.19 (d, J = 6.8 Hz, 6H).

^{13}C NMR (75 MHz, $CDCl_3$, 300 K): δ_C (ppm) = 179.5 (C_q), 142.4 (*C*H), 140.2 (C_q), 139.7 (C_q), 137.3 (C_q), 133.4 (*C*H), 89.0 (C_q), 53.5 (*C*H), 36.9 (*C*H), 20.1 (CH_3), 19.4 (CH_3).

HRMS (ESI) *m/z* = 465.9711 calcd. for $[C_{14}H_{19}NO_3SClINa]^+$ $[M+Na]^+$, found 465.9708.

***N*-((4,5-Dichloro-2-iodophenyl)sulfonyl)-*N*-isopropylisobutyramide (1o):**

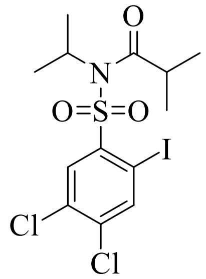

The title compound was prepared following general procedure **GP5** using sulfonamide **S-23** (0.28 g, 0.7 mmol, 1.0 equiv.), DMAP (0.9 mg, 7 µmol, 1 mol%), NEt_3 (0.24 mL, 1.8 mmol, 2.5 equiv.) and isobutyrylchloride (81 µL, 0.77 mmol, 1.1 equiv.) in isopropyl acetate (3.5 mL) and toluene (1.2 mL). Purification by flash column chromatography (Pentane/EtOAc, *v/v* = 9:1) afforded sulfonamide **1o** as a colourless solid (0.30 g, 0.65 mmol, 93%).

MP: 127-128°C.

FT IR (neat) υ (cm^{-1}) = 2973, 2937, 1706, 1434, 1351, 1168, 1146, 1046, 973, 846, 631, 617.

^{1}H NMR (300 MHz, $CDCl_3$, 300 K): δ_H (ppm) = 8.25 (s, 1H), 8.18 (s, 1H), 4.07 (hept, J = 6.8 Hz, 1H), 3.53 (hept, J = 6.7 Hz, 1H), 1.36 (d, J = 6.8 Hz, 6H), 1.20 (d, J = 6.7 Hz, 7H).

^{13}C NMR (75 MHz, $CDCl_3$, 300 K): δ_C (ppm) = 179.4 (C_q), 143.5 (*C*H), 141.4 (C_q), 138.4 (C_q), 133.7 (C_q), 132.7 (*C*H), 89.7 (C_q), 53.6 (*C*H), 37.0 (*C*H), 20.2 (CH_3), 19.4 (CH_3).

HRMS (ESI) *m/z* = 485.9165 calcd. for $[C_{13}H_{16}NO_3SCl_2INa]^+$ $[M+Na]^+$, found 485.9164.

***N*-((2-Iodo-3,5-dimethylphenyl)sulfonyl)-*N*-isopropylisobutyramide (1p):**

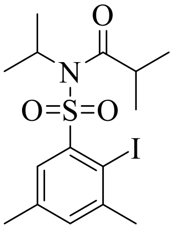

The title compound was prepared following general procedure **GP5** using sulfonamide **S-24** (0.45 g, 1.3 mmol, 1.0 equiv.), DMAP (1.6 mg, 13 µmol, 1 mol%), NEt_3 (0.44 mL, 3.2 mmol, 2.5 equiv.) and isobutyrylchloride (0.15 mL, 1.4 mmol, 1.1 equiv.) in isopropyl acetate (3.0 mL) and toluene (0.8 mL). Purification by flash column chromatography (Pentane/EtOAc, *v/v* = 9:1) afforded sulfonamide **1p** as a colourless solid (0.45 g, 1.2 mmol, 92%).

MP: 121-122 °C.

FT IR (neat) υ (cm^{-1}) = 2973, 2934, 1669, 1453, 1382, 1343, 1194, 1155, 1115, 1088, 1011, 972, 829, 700, 609, 595, 579, 557.

^{1}H NMR (300 MHz, $CDCl_3$, 300 K): δ_H (ppm) = 7.84 (s, 1H), 7.31 (s, 1H), 4.05 (hept, *J* = 6.8 Hz, 1H), 3.65 (hept, *J* = 6.7 Hz, 1H), 2.55 (s, 3H), 2.38 (s, 3H), 1.31 (d, *J* = 6.8 Hz, 6H), 1.19 (d, *J* = 6.7 Hz, 6H).

^{13}C NMR (75 MHz, $CDCl_3$, 300 K): δ_C (ppm) = 179.7 (C_q), 145.5 (C_q), 141.8 (C_q), 138.4 (C_q), 134.9 (*C*H), 129.9 (*C*H), 95.6 (C_q), 53.4 (*C*H), 36.9 (*C*H), 30.0 (CH_3), 20.8 (CH_3), 19.9 (CH_3), 19.4 (CH_3).

HRMS (ESI) *m/z* = 446.0257 calcd. for $[C_{15}H_{22}NO_3SINa]^+$ $[M+Na]^+$, found 446.0253.

***N*-((2-Iodonaphthalen-1-yl)sulfonyl)-*N*-isopropylisobutyramide (1q):**

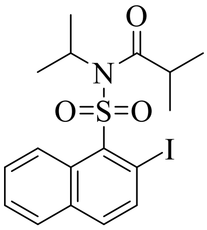

The title compound was prepared following general procedure **GP5** using sulfonamide **S-30** (0.38 g, 1.0 mmol, 1.0 equiv.), DMAP (1.2 mg, 10 µmol, 1 mol%), NEt_3 (0.35 mL, 2.5 mmol, 2.5 equiv.) and isobutyrylchloride (0.12 mL, 1.1 mmol, 1.1 equiv.) in isopropyl acetate (2.4 mL) and toluene (0.7 mL). Purification by flash column chromatography (Pentane/EtOAc, *v/v* = 9:1) afforded sulfonamide **1q** as a colourless solid (0.25 g, 0.56 mmol, 56%).

MP: 137-138 °C.

FT IR (neat) υ (cm^{-1}) = 2974, 2934, 1702, 1386, 1349, 1200, 1156, 1117, 1090, 976, 816, 770, 677, 598, 585.

^{1}H NMR (300 MHz, $CDCl_3$, 300 K): δ_H (ppm) = 9.06 – 9.03 (m, 1H), 8.25 (d, *J* = 8.6 Hz, 1H), 7.90 (dd, *J* = 7.4, 2.2 Hz, 1H), 7.71 – 7.60 (m, 3H), 4.06 (hept, *J* = 6.8 Hz, 1H), 3.71 (hept, *J* = 6.8 Hz, 1H), 1.24 (d, *J* = 6.8 Hz, 6H), 1.18 (d, *J* = 6.8 Hz, 6H).

^{13}C NMR (75 MHz, $CDCl_3$, 300 K): δ_C (ppm) = 179.2 (C_q), 139.9 (*C*H), 136.7 (C_q), 134.7 (*C*H), 133.6 (C_q), 131.9 (C_q), 129.2 (*C*H), 129.1 (*C*H), 127.4 (*C*H), 124.7 (*C*H), 98.5 (C_q), 52.7 (*C*H), 36.9 (*C*H), 19.8 (CH_3), 19.2 (CH_3).

HRMS (ESI) *m/z* = 468.0101 calcd. for $[C_{17}H_{20}NO_3SINa]^+$ $[M+Na]^+$, found 468.0099.

***N*-((5-(Dimethylamino)-2-iodonaphthalen-1-yl)sulfonyl)-*N*-isopropylisobutyramide (1r):**

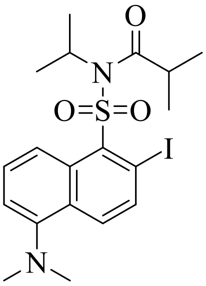

The title compound was prepared following general procedure **GP5** using sulfonamide **S-28** (171 mg, 0.409 mmol, 1.0 equiv.), DMAP (5.0 mg, 4.1 µmol, 1 mol%), NEt_3 (142 µL, 1.02 mmol, 2.5 equiv.) and isobutyrylchloride (47 µL, 0.45 mmol, 1.1 equiv.) in isopropyl acetate (1.0 mL) and toluene (0.6 mL). Purification by flash column chromatography (Pentane/EtOAc, *v/v* = 9:1) afforded sulfonamide **1r** as a yellow oil (124 mg, 0.253 mmol, 62%).

FT IR (neat) υ (cm^{-1}) = 2972, 2937, 2871, 2836, 2791, 1698, 1574, 1400, 1343, 1198, 1153, 1044, 972, 910, 797, 729, 630, 584.

^{1}H NMR (300 MHz, $CDCl_3$, 300 K): δ_H (ppm) = 8.58 (dt, *J* = 9.0, 0.9 Hz, 1H), 8.19 – 8.13 (m, 2H), 7.53 (dd, *J* = 9.0, 7.6 Hz, 1H), 7.20 – 7.17 (m, 1H), 4.09 – 4.00 (m, 1H), 3.67 (hept, *J* = 6.7 Hz, 1H), 2.86 (s, 6H), 1.24 (d, *J* = 6.7 Hz, 6H), 1.14 (d, *J* = 6.7 Hz, 6H).

^{13}C NMR (75 MHz, $CDCl_3$, 300 K): δ_C (ppm) = 179.2 (C_q), 152.0 (C_q), 138.8 (*C*H), 136.4 (C_q), 133.4 (C_q), 131.2 (*C*H), 129.4 (C_q), 129.1 (*C*H), 118.9 (*C*H), 115.6 (*C*H), 98.6 (C_q), 52.7 (*C*H), 45.4 (CH_3), 36.7 (*C*H), 19.8 (CH_3), 19.2 (CH_3).

HRMS (ESI) *m/z* = 511.0523 calcd. for $[C_{19}H_{25}N_2O_3SINa]^+$ $[M+Na]^+$, found 511.0518.

***N*-((2-Iodo-3,5-bis(trifluoromethyl)phenyl)sulfonyl)-*N*-methylisobutyramide (1s):**

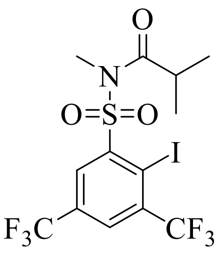

The title compound was prepared following general procedure **GP5** using sulfonamide **S-27** (68.4g, 0.148 mmol, 1.0 equiv.), DMAP (0.2 mg, 1.5 µmol, 1 mol%), NEt_3 (51 µL, 0.37 mmol, 2.5 equiv.) and isobutyrylchloride (17 µL, 0.16 mmol, 1.1 equiv.) in isopropyl acetate (0.4 mL) and toluene (0.2 mL). Purification by flash column

chromatography (Pentane/EtOAc, *v/v* = 9:1) afforded sulfonamide **1s** as a colourless oil (0.23 g, 0.55 mmol, 55%).

FT IR (neat) υ (cm^{-1}) = 2979, 1704, 1612, 1536, 1336, 1282, 1265, 1205, 1169, 1140, 1093, 1057, 626, 593.

^{1}H NMR (600 MHz, $CDCl_3$, 300 K): δ_H (ppm) = 8.78 (d, *J* = 2.2 Hz, 1H), 8.04 (d, *J* = 2.2 Hz, 1H), 3.63 (s, 3H), 2.93 (hept, *J* = 6.8 Hz, 1H), 1.14 (d, *J* = 6.8 Hz, 6H).

^{13}C{^{19}F} NMR (151 MHz, $CDCl_3$, 300 K): δ_C (ppm) = 177.4 (C_q), 147.6, 137.7, 132.7 (*C*H), 131.7, 127.9 (*C*H), 122.4, 122.0, 93.8, 33.5 (*C*H), 33.4 (CH_3), 18.6 (CH_3).

^{19}F{^{1}H} NMR (470 MHz, $CDCl_3$, 300 K): δ_F (ppm) = −61.9, −63.2.

HRMS (ESI) *m/z* = 525.9379 calcd. for $[C_{13}H_{12}NO_3SF_6INa]^+$ $[M+Na]^+$, found 525.9376.

***N*-((3-Iodopyridin-2-yl)sulfonyl)-*N*-isopropylisobutyramide (1t):**

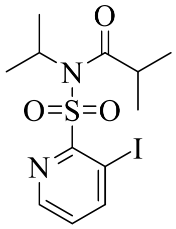

The title compound was prepared following general procedure **GP5** using sulfonamide **S-34** (0.33 g, 1.0 mmol, 1.0 equiv.), DMAP (1.2 mg, 10 µmol, 1 mol%), NEt_3 (0.35 mL, 2.5 mmol, 2.5 equiv.) and isobutyrylchloride (0.12 mL, 1.1 mmol, 1.1 equiv.) in isopropyl acetate (2.4 mL) and toluene (0.7 mL). Purification by flash column chromatography (Pentane/EtOAc, *v/v* = 4:1) afforded sulfonamide **1t** as a colourless solid (0.37 g, 0.94 mmol, 94%).

MP: 110-111 °C.

FT IR (neat) υ (cm^{-1}) = 3424, 2973, 1658, 1508, 1491, 1081, 1009, 906, 729, 700, 647.

^{1}H NMR (300 MHz, $CDCl_3$, 300 K): δ_H (ppm) = 8.56 (dd, *J* = 4.5, 1.5 Hz, 1H), 8.45 (dd, *J* = 7.9, 1.5 Hz, 1H), 7.22 (dd, *J* = 7.9, 4.5 Hz, 1H), 4.59 (hept, *J* = 6.8 Hz, 1H), 3.00 – 2.87 (m, 1H), 1.48 (d, *J* = 6.8 Hz, 6H), 1.11 (d, *J* = 6.6 Hz, 6H).

^{13}C NMR (75 MHz, $CDCl_3$, 300 K): δ_C (ppm) = 179.9 (C_q), 158.2 (C_q), 151.0 (*C*H), 146.9 (*C*H), 127.4 (*C*H), 88.0 (C_q), 53.6 (*C*H), 36.9 (*C*H), 20.7 (CH_3), 19.8 (CH_3).

HRMS (ESI) *m/z* = 418.9897 calcd. for $[C_{12}H_{17}N_2O_3SINa]^+$ $[M+Na]^+$, found 418.9890.

***N*-((3-Iodothiophen-2-yl)sulfonyl)-*N*-isopropylisobutyramide (1u):**

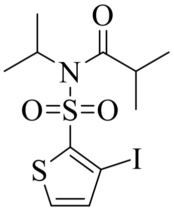

The title compound was prepared following general procedure **GP5** using sulfonamide **S-32** (0.17 g, 0.5 mmol, 1.0 equiv.), DMAP (0.6 mg, 5.0 µmol, 1 mol%), NEt_3 (0.17 mL, 1.3 mmol, 2.5 equiv.) and isobutyrylchloride (58 µL, 0.55 mmol, 1.1 equiv.) in isopropyl acetate (1.2 mL) and toluene (0.4 mL). Purification by flash column chromatography (Pentane/EtOAc, *v/v* = 9:1) afforded sulfonamide **1u** as a colourless oil (0.20 g, 0.49 mmol, 98%).

FT IR (neat) υ (cm^{-1}) = 3103, 2973, 2937, 1703, 1469, 1349, 1185, 1149, 1120, 1086, 1034, 971, 866, 735, 674, 613, 590, 570.

^{1}H NMR (300 MHz, $CDCl_3$, 300 K): δ_H (ppm) = 7.55 (d, *J* = 5.2 Hz, 1H), 7.24 (d, *J* = 5.2 Hz, 1H), 4.49 – 4.35 (m, 1H), 3.57 (hept, *J* = 6.7 Hz, 1H), 1.40 (d, *J* = 6.8 Hz, 6H), 1.16 (d, *J* = 6.7 Hz, 6H).

^{13}C NMR (75 MHz, $CDCl_3$, 300 K): δ_C (ppm) = 179.1 (C_q), 140.0 (C_q), 138.8 (*C*H), 133.3 (*C*H), 84.8 (C_q), 54.1 (*C*H), 36.7 (*C*H), 20.6 (CH_3), 19.6 (CH_3).

HRMS (ESI) *m/z* = 423.9509 calcd. for $[C_{11}H_{16}NO_3S_2INa]^+$ $[M+Na]^+$, found 423.9501.

4-Iodo-*N*-isobutyryl-3-(*N*-isobutyryl-*N*-isopropylsulfamoyl)-*N*-isopropylbenzamide (1v):

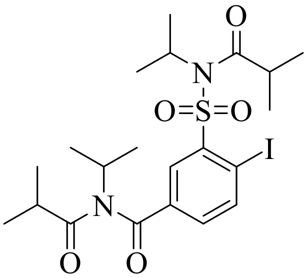

The title compound was prepared following general procedure **GP5** using sulfonamide **S-29** (51.9 mg, 0.127 mmol, 1.0 equiv.), DMAP (0.1 mg, 1 µmol, 1 mol%), NEt_3 (44 µL, 0.32 mmol, 2.5 equiv.) and isobutyrylchloride (29 µL, 0.14 mmol, 2.1 equiv.) in isopropyl acetate (0.35 mL) and toluene (0.17 mL). Purification by flash column chromatography (Pentane/EtOAc, *v/v* = 9:1) afforded sulfonamide **1v** as a colourless oil (54.6 mg, 0.099 mmol, 78%).

FT IR (neat) υ (cm^{-1}) = 2973, 2937, 2875, 1706, 0665, 1366, 1351, 1317, 1168, 1155, 1075, 1032, 973, 913, 774, 729, 654, 627, 611.

^{1}H NMR (300 MHz, $CDCl_3$, 300 K): δ_H (ppm) = 8.38 (d, *J* = 1.8 Hz, 1H), 7.93 (dd, *J* = 8.1, 1.8 Hz, 1H), 7.36 (d, *J* = 8.1 Hz, 1H), 4.46 (hept, *J* = 6.9 Hz, 1H), 4.16 – 4.02 (m, 1H), 3.13 – 2.92 (m, 2H), 1.50 (d, *J* = 6.9 Hz, 6H), 1.43 (d, *J* = 6.8 Hz, 6H), 1.11 (d, *J* = 6.7 Hz, 6H), 1.07 (d, *J* = 6.7 Hz, 6H).

^{13}C NMR (75 MHz, $CDCl_3$, 300 K): δ_C (ppm) = 182.7 (C_q), 178.6 (C_q), 171.1 (C_q), 147.2 (C_q), 142.5 (C_q), 138.4 (*C*H), 127.6 (*C*H), 127.1 (*C*H), 92.1 (C_q), 53.1 (*C*H), 51.4 (*C*H), 37.3 (*C*H), 35.7 (*C*H), 21.4 (CH_3), 20.6 (CH_3), 19.5 (2 x CH_3).

HRMS (ESI) *m/z* = 573.0891 calcd. for $[C_{21}H_{31}N_2O_5SINa]^+$ $[M+Na]^+$, found 573.0887.

***N*-((2-Iodophenyl)sulfonyl)-*N*-isopropyl-2-methylpentanamide (1w):**

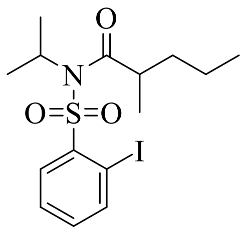

Following a procedure by *Hu, Lan et al.*[16] diisopropylamine (845 µL, 6.00 mmol, 3.0 equiv.) was dissolved in anhydrous THF (5 mL) at 0 °C. After slow addition of *n*-BuLi (1.6 M solution in hexanes, 3.13 mL, 5.00 mmol, 2.5 equiv.) the reaction mixture was warmed to room temperature and stirred for further 30 minutes. The temperature was decreased to −15 °C and pentanoic acid (220 µL, 2.00 mmol, 1.0 equiv.) was added dropwise. After stirring for 1 h methyl iodide (275 µL, 4.40 mmol, 2.2 equiv.) was added. The solution was warmed to room temperature overnight and then adjusted to pH = 1 with concentrated HCl. The aqueous phase was extracted with EtOAc (3 x 10 mL). The combined organic phases were dried over Na_2SO_4 and concentrated *in vacuo* to afford crude 2-methylpentanoic acid as a colourless liquid (104 mg, 0.896 mmol, 45%). The crude acid was dissolved in anhydrous CH_2Cl_2 (2.0 mL) with two drops of DMF. The mixture was cooled to 0 °C and treated dropwise with oxalyl chloride (310 µL, 3.60 mmol, 4.0 equiv. with respect to the acid). The solution was warmed to room temperature overnight. After removal of the solvent and excess oxalyl chloride *in vacuo*, the crude acyl chloride was dissolved in anhydrous toluene (0.6 mL) and directly reacted to sulfonamide **1w** following general procedure **GP5** using sulfonamide **S-18** (163 mg, 0.500 mmol, 1.0 equiv.), DMAP (0.6 mg, 5 µmol, 1 mol%) and NEt_3 (170 µL, 1.25 mmol, 2.5 equiv.) in isopropyl acetate (1.2 mL). Purification by reverse-phase-MPLC ($MeOH/H_2O$-gradient from 5% to 90%) afforded the title compound **1w** as a colourless oil (54.0 mg, 0.128 mmol, 26% with respect to sulfonamide **S-18**).

FT IR (neat) υ (cm^{-1}) = 2964, 2935, 1642, 1450, 1350, 1189, 1163, 1090, 1015, 970, 760, 731, 598, 577.

^{1}H NMR (300 MHz, $CDCl_3$, 300 K): δ_H (ppm) = 8.21 (dd, *J* = 8.0, 1.6 Hz, 1H), 8.13 (dd, *J* = 7.8, 1.2 Hz, 1H), 7.56 (ddd, *J* = 8.0, 7.4, 1.3 Hz, 1H), 7.27 (td, *J* = 7.7, 1.6 Hz, 1H), 4.21 (hept, *J* = 6.8 Hz, 1H), 3.43 – 3.32 (m, 1H), 1.72 – 1.69 (m, 1H), 1.39 – 1.24 (m, 9H), 1.13 (d, *J* = 6.7 Hz, 3H), 0.85 (t, *J* = 7.1 Hz, 3H).

^{13}C NMR (75 MHz, $CDCl_3$, 300 K): δ_C (ppm) = 179.1 (C_q), 143.1 (*C*H), 142.0 (C_q), 134.1 (*C*H), 131.6 (*C*H), 128.5 (*C*H), 92.7 (C_q), 53.7 (*C*H), 41.7 (*C*H), 36.4 (CH_2), 20.3 (CH_3), 20.2 (CH_2), 17.1 (CH_3), 14.1 (CH_3).

HRMS (ESI) *m/z* = 446.0257 calcd. for $[C_{15}H_{22}NO_3SINa]^+$ $[M+Na]^+$, found 446.0253.

N-((2-Iodophenyl)sulfonyl)-N-isopropyl-2,3-dimethylbutanamide (1x):

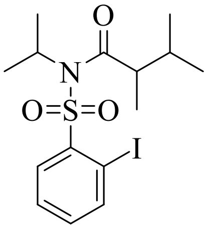

The title compound was prepared analogously to *N*-acylated sulfonamide **1w** using 3-methylbutanoic acid (220 µL, 2.00 mmol, 1.0 equiv.), *n*-BuLi (1.6 M solution in hexanes, 3.13 mL, 5.00 mmol, 2.5 equiv.), diisopropylamine (845 µL, 6.00 mmol, 3.0 equiv.) and methyl iodide (275 µL, 4.40 mmol, 2.2 equiv.). Crude 2,3-dimethylbutanoic acid was isolated as a colourless oil (96.9 mg, 0.834 mmol, 42%). Whereas, without further purification the crude acid was reacted to the title compound using oxalyl chloride (310 µL, 3.60 mmol, 4.6 equiv. with respect to the acid), sulfonamide **S-18** (163 mg, 0.500 mmol, 1.0 equiv.), DMAP (0.6 mg, 5 µmol, 1 mol%) and NEt_3 (170 µL, 1.25 mmol, 2.5 equiv.) in THF (5 mL), CH_2Cl_2 (2 mL), isopropyl acetate (1.2 mL) and toluene (0.6 mL). Purification by reverse-phase-MPLC (MeOH/water, gradient from 5% to 90%) afforded the title compound **1x** as a colourless oil (58.9 mg, 0.139 mmol, 28% with respect to sulfonamide **S-18**).

FT IR (neat) υ (cm^{-1}) = 2968, 2938, 1709, 1691, 1349, 1185, 1164, 1148, 1015, 966, 727, 599, 576.

^{1}H NMR (300 MHz, $CDCl_3$, 300 K): δ_H (ppm) = 8.24 (dd, *J* = 8.0, 1.6 Hz, 1H), 8.13 (dd, *J* = 7.9, 1.3 Hz, 1H), 7.56 (ddd, *J* = 7.9, 7.4, 1.3 Hz, 1H), 7.30 – 7.24 (m, 1H), 4.30 (hept, *J* = 6.8 Hz, 1H), 3.21 – 3.12 (m, 1H), 2.02 – 1.91 (m, 1H), 1.45 (d, *J* = 6.8 Hz, 3H), 1.35 (d, *J* = 6.8 Hz, 3H), 1.08 (d, *J* = 6.8 Hz, 3H), 0.84 – 0.81 (m, 6H).

^{13}C NMR (75 MHz, $CDCl_3$, 300 K): δ_C (ppm) = 178.5 (C_q), 143.1 (*C*H), 142.2 (C_q), 134.1 (*C*H), 131.9 (*C*H), 128.5 (*C*H), 92.7 (C_q), 53.8 (*C*H), 47.5 (*C*H), 31.4 (*C*H), 21.2 (CH_3), 20.5 (CH_3), 20.4 (CH_3), 18.2 (CH_3), 13.1 (CH_3).

HRMS (ESI) *m/z* = 446.0257 calcd. for $[C_{15}H_{22}NO_3SINa]^+$ $[M+Na]^+$, found 446.0253.

N-((2-Iodophenyl)sulfonyl)-N-isopropylcyclohexanecarboxamide (1y):

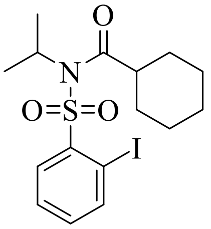

The title compound was prepared following general procedure **GP5** using sulfonamide **S-18** (0.20 g, 0.60 mmol, 1.0 equiv.), DMAP (0.7 mg, 6 µmol, 1 mol%), NEt_3 (0.21 mL, 1.5 mmol, 2.5 equiv.) and cyclohexylcarbonyl chloride (88 µL, 0.66 mmol, 1.1 equiv.) in isopropyl acetate (3 mL) and toluene (1 mL). Purification by flash column chromatography (EtOAc/Pentane, *v/v* = 1:1) afforded sulfonamide **1y** as a colourless solid (0.24 g, 0.56 mmol, 93%).

MP: 83-84 °C.

FT IR (neat) υ (cm^{-1}) = 2971, 2932, 2854, 1700, 1448, 1350, 1179, 1156, 1016, 974, 729, 602, 577.

^{1}H NMR (300 MHz, $CDCl_3$, 300 K): δ_H (ppm) = 8.13 (dd, *J* = 8.0, 1.6 Hz, 1H), 8.03 (dd, *J* = 7.9, 1.2 Hz, 1H), 7.50 – 7.44 (m, 1H), 7.21 – 7.15 (m, 1H), 4.05 (hept, *J* = 6.8 Hz, 1H), 3.20 – 3.12 (m, 1H), 1.81 – 1.77 (m, 2H), 1.68 – 1.34 (m, 6H), 1.34 (d, *J* = 6.8 Hz, 6H), 1.25 – 1.11 (m, 2H).

^{13}C NMR (75 MHz, $CDCl_3$, 300 K): δ_C (ppm) = 178.4 C_q), 143.1 (*C*H), 141.9 (C_q), 134.0 (*C*H), 131.6 (*C*H), 128.5 (*C*H), 92.7 (C_q), 53.5 (*C*H), 46.7 (*C*H), 29.6 (CH_2), 25.8 (CH_2), 25.6 (CH_2), 20.2 (CH_3).

HRMS (ESI) *m/z* = 458.0257, calcd. for $[C_{16}H_{22}INNaO_3S]^+$ $[M+Na]^+$, found 458.0252.

2-(4-Fluorophenyl)-N-((2-iodophenyl)sulfonyl)-*N*-isopropylpropanamide (1z):

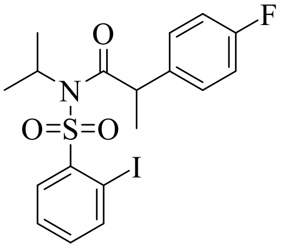

The title compound was prepared analogously to *N*-acylated sulfonamide **1w** using (4-fluorophenyl)acetic acid (308 mg, 2.00 mmol, 1.0 equiv.), *n*-BuLi (1.6 M solution in hexanes, 3.13 mL, 5.00 mmol, 2.5 equiv.), diisopropylamine (845 µL, 6.00 mmol, 3.0 equiv.) and methyl iodide (275 µL, 4.40 mmol, 2.2 equiv.). Crude 2-(4-fluorophenyl)propanoic acid was isolated as a colourless oil (163 mg, 0.969 mmol, 48%). Whereas, without further purification the crude acid was reacted to the title compound using oxalyl chloride (310 µL, 3.60 mmol, 3.7 equiv. with respect to the acid), sulfonamide **S-18** (163 mg, 0.500 mmol, 1.0 equiv.), DMAP (0.6 mg, 5 µmol, 1 mol%) and NEt_3 (170 µL, 1.25 mmol, 2.5 equiv.) in THF (5 mL), CH_2Cl_2 (2 mL), isopropyl acetate (1.2 mL) and toluene (0.6 mL). Purification by reverse-phase-MPLC (MeOH/water, gradient from 5% to 90%) afforded the title compound **1z** as a colourless solid (71.2 mg, 0.150 mmol, 30% with respect to sulfonamide **S-18**).

MP: 128-129 °C.

FT IR (neat) υ (cm^{-1}) = 2975, 2936, 1709, 1691, 1350, 1334, 1184, 1153, 1015, 969, 908, 838, 726, 597, 574.

^{1}H NMR (600 MHz, $CDCl_3$, 300 K): δ_H (ppm) = 8.13 (ddd, *J* = 7.9, 5.2, 1.4 Hz, 2H), 7.55 – 7.51 (m, 1H), 7.30 – 7.23 (m, 3H), 7.01 – 6.97 (m, 2H), 4.89 (q, *J* = 6.9 Hz, 1H), 3.99 – 3.93 (m, 1H), 1.49 (d, *J* = 6.9 Hz, 3H), 1.25 (d, *J* = 6.8 Hz, 3H), 1.19 (d, *J* = 6.8 Hz, 3H).

^{13}C{^{19}F} NMR (151 MHz, $CDCl_3$, 300 K): δ_C (ppm) = 176.2 (C_q), 162.0 (C_q), 143.1 (*C*H), 141.1 (C_q), 135.9 (C_q), 134.2 (*C*H), 131.7 (*C*H), 129.8 (*C*H), 128.6 (*C*H), 115.4 (*C*H), 93.1 (C_q), 54.0 (*C*H), 47.1 (*C*H), 19.9 (2 x CH_3), 19.5 (CH_3).

^{19}F NMR{^{1}H} (471 MHz, $CDCl_3$, 300 K): δ_F (ppm) = −115.6.

HRMS (ESI) *m/z* = 498.0007 calcd. for $[C_{18}H_{19}NO_3SFINa]^+$ $[M+Na]^+$, found 498.0005.

2-(4-Chlorophenyl)-N-((2-iodophenyl)sulfonyl)-*N*-isopropylpropanamide (1aa):

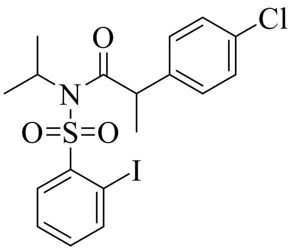

The title compound was prepared analogously to *N*-acylated sulfonamide **1w** using (4-chlorophenyl)acetic acid (341 mg, 2.00 mmol, 1.0 equiv.), *n*-BuLi (1.6 M solution in hexanes, 3.13 mL, 5.00 mmol, 2.5 equiv.), diisopropylamine (845 µL, 6.00 mmol, 3.0 equiv.) and methyl iodide (275 µL, 4.40 mmol, 2.2 equiv.). Crude 2-(4-chlorophenyl)propanoic acid was isolated as a colourless oil (157 mg, 0.850 mmol, 43%). Whereas, without further purification the crude acid was reacted to the title compound using oxalyl chloride (310 µL, 3.60 mmol, 4.2 equiv. with respect to the acid), sulfonamide **S-18** (163 mg, 0.500 mmol, 1.0 equiv.), DMAP (0.6 mg, 5 µmol, 1 mol%) and NEt_3 (170 µL, 1.25 mmol, 2.5 equiv.) in THF (5 mL), CH_2Cl_2 (2 mL), isopropyl acetate (1.2 mL) and toluene (0.6 mL). Purification by reverse-phase-MPLC (MeOH/water, gradient from 5% to 90%) afforded the title compound **1aa** as a colourless oil (48.5 mg, 98.6 µmol, 20% with respect to sulfonamide **S-18**).

FT IR (neat) υ (cm^{-1}) = 2974, 2935, 1691, 1491, 1351, 1334, 1183, 1154, 1092, 1014, 969, 729, 596, 574.

^{1}H NMR (300 MHz, $CDCl_3$, 300 K): δ_H (ppm) = 8.15 – 8.12 (m, 2H), 7.57 – 7.51 (m, 1H), 7.32 – 7.21 (m, 5H), 4.90 (q, *J* = 6.9 Hz, 1H), 3.96 (hept, *J* = 6.8 Hz, 1H), 1.49 (d, *J* = 6.9 Hz, 3H), 1.27 – 1.20 (m, 6H).

^{13}C NMR (75 MHz, $CDCl_3$, 300 K): δ_C (ppm) = 175.9 (C_q), 143.1 (*C*H), 141.2 (C_q), 138.8 (C_q), 134.3 (*C*H), 133.0 (C_q), 131.7 (*C*H), 129.6 (*C*H), 128.7 (*C*H), 127.0 (*C*H), 93.0 (C_q), 54.0 (*C*H), 47.3 (*C*H), 25.5 (CH_3), 19.9 (CH_3), 19.8 (CH_3), 19.5 (CH_3).

HRMS (ESI) *m/z* = 513.9711 calcd. for $[C_{18}H_{19}NO_3SClINa]^+$ $[M+Na]^+$, found 513.9712.

2-(4-Bromophenyl)-N-((2-iodophenyl)sulfonyl)-*N*-isopropylpropanamide (1ab):

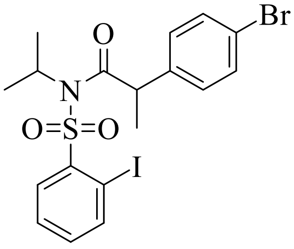

The title compound was prepared analogously to *N*-acylated sulfonamide **1w** using (4-bromophenyl)acetic acid (430 mg, 2.00 mmol, 1.0 equiv.), *n*-BuLi (1.6 M solution in hexanes, 3.13 mL, 5.00 mmol, 2.5 equiv.), diisopropylamine (845 µL, 6.00 mmol, 3.0 equiv.) and methyl iodide (275 µL, 4.40 mmol, 2.2 equiv.). Crude 2-(4-bromophenyl)propanoic acid was isolated as a colourless oil (201 mg, 0.877 mmol, 44%). Whereas, without further purification the crude acid was reacted to the title compound using oxalyl chloride (310 µL, 3.60 mmol, 4.1 equiv. with respect to the acid), sulfonamide **S-18** (163 mg, 0.500 mmol, 1.0 equiv.), DMAP (0.6 mg, 5 µmol, 1 mol%) and NEt_3 (170 µL, 1.25 mmol, 2.5 equiv.) in THF (5 mL), CH_2Cl_2 (2 mL), isopropyl acetate (1.2 mL) and toluene (0.6 mL). Purification by reverse-phase-MPLC (MeOH/water, gradient from 5% to 90%) afforded the title compound **1ab** as a colourless solid (166 mg, 0.310 mmol, 62% with respect to sulfonamide **S-18**).

MP: 121-122 °C.

FT IR (neat) υ (cm^{-1}) = 2974, 2935, 1702, 1354, 1332, 1184, 1156, 1013, 970, 760, 730, 600, 575.

^{1}H NMR (300 MHz, $CDCl_3$, 300 K): δ_H (ppm) = 8.12 (dd, *J* = 7.9, 1.4 Hz, 2H), 7.56 – 7.50 (m, 1H), 7.45 – 7.40 (m, 2H), 7.31 – 7.25 (m, 1H), 7.18 – 7.14 (m, 2H), 4.88 (q, *J* = 6.9 Hz, 1H), 3.95 (hept, *J* = 6.8 Hz, 1H), 1.49 (d, *J* = 6.9 Hz, 3H), 1.25 (d, *J* = 6.9 Hz, 3H), 1.20 (d, *J* = 6.8 Hz, 3H).

^{13}C NMR (75 MHz, $CDCl_3$, 300 K): δ_C (ppm) = 175.8 (C_q), 143.1 (*C*H), 141.2 (C_q), 139.3 (C_q), 134.2 (*C*H), 131.7 (*C*H), 130.0 (*C*H), 128.6 (*C*H), 121.2 (C_q), 93.0 (C_q), 54.0 (*C*H), 47.3 (*C*H), 19.9 (CH_3), 19.8 (CH_3), 19.5 (CH_3).

HRMS (ESI) *m/z* = 557.9206 calcd. for $[C_{18}H_{19}NO_3SBrINa]^+$ $[M+Na]^+$, found 557.9211.

***N*-((2-Iodophenyl)sulfonyl)-*N*-isopropyl-2-(*p*-tolyl)propanamide (1ac):**

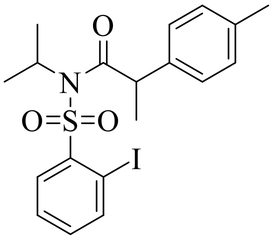

The title compound was prepared analogously to *N*-acylated sulfonamide **1w** using (4-tolyl)acetic acid (300 mg, 2.00 mmol, 1.0 equiv.), *n*-BuLi (1.6 M-solution in hexanes, 3.13 mL, 5.00 mmol, 2.5 equiv.), diisopropylamine (845 µL, 6.00 mmol, 3.0 equiv.) and methyl iodide (275 µL, 4.40 mmol, 2.2 equiv.). Crude

2-(4-tolyl)propanoic acid was isolated as a colourless oil (140 mg, 0.853 mmol, 43%). Whereas, without further purification the crude acid was reacted to the title compound using oxalyl chloride (310 µL, 3.60 mmol, 4.2 equiv. with respect to the acid), sulfonamide **S-18** (163 mg, 0.500 mmol, 1.0 equiv.), DMAP (0.6 mg, 5 µmol, 1 mol%) and NEt_3 (170 µL, 1.25 mmol, 2.5 equiv.) in THF (5 mL), CH_2Cl_2 (2 mL), isopropyl acetate (1.2 mL) and toluene (0.6 mL). Purification by reverse-phase-MPLC (MeOH/water, gradient from 5% to 90%) afforded the title compound **1ac** as a colourless solid (42.2 mg, 89.5 µmol, 18% with respect to sulfonamide **S-18**).

MP: 101-102 °C.

FT IR (neat) υ (cm^{-1}) = 2976, 2934, 1700, 1353, 1186, 1156, 1015, 969, 726, 599, 576.

^{1}H NMR (600 MHz, $CDCl_3$, 300 K): δ_H (ppm) = 8.12 – 8.09 (m, 2H), 7.51 (ddd, *J* = 8.0, 7.4, 1.2 Hz, 1H), 7.28 – 7.24 (m, 1H), 7.14 – 7.09 (m, 4H), 4.80 (q, *J* = 6.9 Hz, 1H), 4.00 (hept, *J* = 6.8 Hz, 1H), 2.32 (s, 3H), 1.48 (d, *J* = 6.9 Hz, 3H), 1.28 (d, *J* = 6.8 Hz, 3H), 1.21 (d, *J* = 6.8 Hz, 3H).

^{13}C NMR (151 MHz, $CDCl_3$, 300 K): δ_C (ppm) = 176.4 (C_q), 143.0 (*C*H), 141.5 (C_q), 137.1 (C_q), 136.7 (C_q), 134.1 (*C*H), 131.6 (*C*H), 129.2 (*C*H), 128.5 (*C*H), 128.0 (*C*H), 93.0 (C_q), 53.9 (*C*H), 47.5 (*C*H), 21.1 (CH_3), 20.1 (CH_3), 19.8 (CH_3), 19.6 (CH_3).

HRMS (ESI) *m/z* = 494.0257 calcd. for $[C_{19}H_{22}NO_3SINa]^+$ $[M+Na]^+$, found 494.0255.

***N*-((2-Iodophenyl)sulfonyl)-2-(4-isobutylphenyl)-*N*-isopropylpropanamide (1ad):**

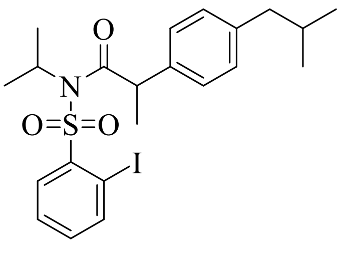

Following a slightly modified procedure by *Knaus* and coworkers[17] *Ibuprofene*® (0.21 g, 1.0 mmol, 1.0 equiv.) was dissolved in anhydrous CH_2Cl_2 (1.0 mL) and two drops of DMF at 0 °C. Oxalyl chloride (0.22 mL, 2.5 mmol, 2.5 equiv.) was added slowly at this temperature and the reaction mixture was then allowed to reach room temperature. After three hours the solvent and excess oxalyl chloride was removed *in vacuo*. The crude acyl chloride was dissolved in anhydrous toluene (1.0 mL) and directly reacted to the *N*-acylated sulfonamide **1ae** following general procedure **GP5** using sulfonamide **S-3d** (0.20 g, 0.60 mmol, 1.0 equiv.), DMAP (0.7 mg, 6 µmol, 1 mol%) and NEt_3 (0.21 mL, 1.5 mmol, 2.5 equiv.) in isopropyl acetate (3.0 mL). Purification by flash column chromatography (EtOAc/Pentane, *v/v* = 19:1) afforded the title compound **1ad** as a colourless solid (0.31 g, 0.60 mmol, 99%).

MP: 153-154 °C.

FT IR (neat) υ (cm^{-1}) = 2953, 2931, 2868, 1759, 1730, 1451, 1368, 1353, 1171, 1116, 1067, 1017, 601.

^{1}H NMR (300 MHz, $CDCl_3$, 300 K): δ_H (ppm) = 8.11 (ddd, *J* = 7.9, 4.7, 1.4 Hz, 2H), 7.50 (td, *J* = 7.7, 1.3 Hz, 1H), 7.28 – 7.22 (m, 1H), 7.13 – 7.04 (m, 4H), 4.76 (q, *J* = 6.9 Hz, 1H), 4.08 – 3.99 (m, 1H), 2.44 (d, *J* = 7.2 Hz, 2H), 1.92 – 1.78 (m, 1H), 1.49 (d, *J* = 6.8 Hz, 3H), 1.30 (d, *J* = 6.8 Hz, 3H), 1.19 (d, *J* = 6.7 Hz, 3H), 0.90 (d, *J* = 6.6 Hz, 6H).

^{13}C NMR (75 MHz, $CDCl_3$, 300 K): δ_C (ppm) = 176.5 (C_q), 143.1 (*C*H), 141.7 (C_q), 140.5 (C_q), 137.3 (C_q), 134.0 (*C*H), 131.6 (*C*H), 129.2 (*C*H), 128.5 (*C*H), 127.8 (*C*H), 92.9 (C_q), 53.9 (*C*H), 47.5 (*C*H), 45.1 (CH_2), 30.2 (*C*H), 22.4 (CH_3), 22.4 (CH_3), 20.3 (CH_3), 19.8 (CH_3), 19.6 (CH_3).

HRMS (ESI) *m/z* = 536.0727 calcd. for $[C_{22}H_{28}NO_3SINa]^+$ $[M+Na]^+$, found 536.0725.

***N*-((2-Iodophenyl)sulfonyl)-*N*-isopropyl-2-methoxy-2-phenylacetamide (1ae):**

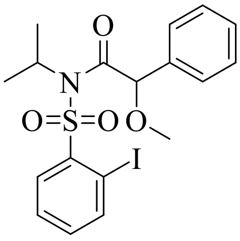

The title compound was prepared analogously to the *N*-acylated sulfonamide **1ad** using mandelic acid (166 mg, 0.800 mmol, 1.0 equiv.), oxalyl chloride (310 µL, 3.60 mmol, 4.0 equiv.), sulfonamide **S-18** (163 mg, 0.500 mmol, 1.0 equiv.), DMAP (0.6 mg, 5 µmol, 1 mol%) and NEt_3 (170 µL, 1.25 mmol, 2.5 equiv.) in CH_2Cl_2 (2 mL), isopropyl acetate (1.2 mL) and toluene (0.6 mL). Purification by reverse-phase-MPLC (MeOH/water, gradient from 5% to 90%) afforded the title compound as a colourless oil (157 mg, 0.332 mmol, 66%).

FT IR (neat) υ (cm^{-1}) = 2978, 1709, 1446, 1351, 1184, 1159, 1090, 1015, 975, 909, 726, 699, 597, 573.

^{1}H NMR (300 MHz, $CDCl_3$, 300 K): δ_H (ppm) = 8.12 (dd, *J* = 8.0, 1.4 Hz, 2H), 7.55 – 7.24 (m, 7H), 5.90 (s, 1H), 3.82 (hept, *J* = 6.8 Hz, 1H), 3.41 (s, 3H), 1.19 (d, *J* = 6.8 Hz, 3H), 1.11 (d, *J* = 6.8 Hz, 3H).

^{13}C NMR (75 MHz, $CDCl_3$, 300 K): δ_C (ppm) = 172.7 (C_q), 143.1 (*C*H), 140.7 (C_q), 135.6 (C_q), 134.4 (*C*H), 131.9 (*C*H), 128.9 (*C*H), 128.7 (*C*H), 128.6 (*C*H), 128.5 (*C*H), 93.3 (C_q), 84.4 (*C*H), 57.5 (*C*H), 54.4 (CH_3), 19.7 (CH_3), 19.1 (CH_3).

HRMS (ESI) *m/z* = 496.0050 calcd. for $[C_{18}H_{20}NO_4SINa]^+$ $[M+Na]^+$, found 496.0047.

***N*-((2-Iodophenyl)sulfonyl)-*N*-isopropyl-2-phenoxyacetamide (1af):**

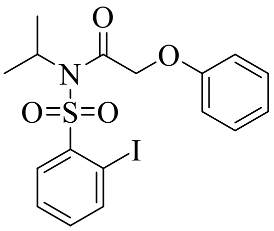

The title compound was prepared following general procedure **GP5** using sulfonamide **S-18** (0.12 g, 0.37 mmol, 1.0 equiv.), DMAP (0.5 mg, 4 µmol, 1 mol%), NEt_3 (0.13 mL, 0.93 mmol, 2.5 equiv.) and 2-phenoxyacetyl chloride (56 µL, 0.41 mmol, 1.1 equiv.) in isopropyl acetate (1.2 mL) and toluene (0.35 mL). Purification by flash column chromatography (Et_2O/Pentane, *v/v* = 1:4) afforded sulfonamide **1af** as a colourless solid (0.13 g, 0.29 mmol, 78%).

MP: 96-97 °C.

FT IR (neat) υ (cm^{-1}) = 2978, 1716, 1600, 1495, 1424, 1355, 1198, 1166, 1085, 1016, 987, 752, 727, 690, 603, 585.

^{1}H NMR (600 MHz, $CDCl_3$, 300 K): δ_H (ppm) = 8.31 (dd, J = 8.0, 1.6 Hz, 1H), 8.17 (dd, J = 7.9, 1.2 Hz, 1H), 7.61 (ddd, J = 7.9, 7.4, 1.2 Hz, 1H), 7.36 – 7.29 (m, 3H), 7.02 – 6.96 (m, 3H), 5.29 (s, 2H), 3.81 – 3.78 (m, 1H), 1.32 (d, J = 6.8 Hz, 6H).

^{13}C NMR (151 MHz, $CDCl_3$, 300 K): δ_C (ppm) = 169.7 (C_q), 158.0 (C_q), 143.2 (C_q), 139.8 (*C*H), 134.8 (*C*H), 132.6 (*C*H), 129.5 (*C*H), 128.8 (*C*H), 121.5 (*C*H), 114.9 (*C*H), 93.4 (C_q), 70.1 (CH_2), 54.3 (*C*H), 19.3 (CH_3).

HRMS (ESI) *m/z* = 481.9893 calcd. for $[C_{17}H_{18}NO_4SINa]^+$ $[M+Na]^+$, found 481.9889.

***N*-((2-Iodophenyl)sulfonyl)-*N*-isopropylacetamide (1ag):**

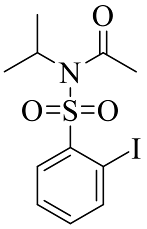

The title compound was prepared following general procedure **GP5** using sulfonamide **S-18** (98 mg, 0.30 mmol, 1.0 equiv.), DMAP (0.4 mg, 3 µmol, 1 mol%), NEt_3 (0.10 mL, 0.75 mmol, 2.5 equiv.) and acetyl chloride (24 µL, 0.33 mmol, 1.1 equiv.) in isopropyl acetate (0.7 mL) and toluene (0.3 mL). Purification by flash column chromatography (EtOAc/Pentane, *v/v* = 1:9) afforded sulfonamide **1ag** as a colourless solid (90 mg, 0.24 mmol, 81%).

MP: 102-103 °C.

FT IR (neat) υ (cm^{-1}) = 2975, 2936, 1700, 1458, 1382, 1344, 1193, 1153, 1086, 1013, 973, 829, 702, 603, 580.

^{1}H NMR (300 MHz, $CDCl_3$, 300 K): δ_H (ppm) = 8.24 (dd, J = 8.0, 1.7 Hz, 1H), 8.15 (dd, J = 8.0, 1.2 Hz, 1H), 7.57 (ddd, J = 8.0, 7.4, 1.2 Hz, 1H), 7.32 – 7.26 (m, 1H), 3.96 (hept, J = 6.8 Hz, 1H), 2.58 (s, 3H), 1.33 (d, J = 6.8 Hz, 6H).

^{13}C NMR (75 MHz, $CDCl_3$, 300 K): δ_C (ppm) = 171.3 (C_q), 143.2 (*C*H), 140.8 (C_q), 134.3 (*C*H), 132.1 (*C*H), 128.6 (*C*H), 93.0 (C_q), 53.5 (*C*H), 28.7 (CH_3), 19.7 (CH_3).

HRMS (ESI) *m/z* = 389.9631 calcd. for $[C_{11}H_{14}NO_3SINa]^+$ $[M+Na]^+$, found 389.9630.

***N*-((2-Iodophenyl)sulfonyl)-*N*-isopropylpropionamide (1ah):**

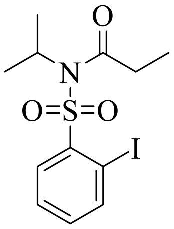

The title compound was prepared following general procedure **GP5** using sulfonamide **S-18** (98 mg, 0.30 mmol, 1.0 equiv.), DMAP (0.4 mg, 3 µmol, 1 mol%), NEt_3 (0.10 mL, 0.75 mmol, 2.5 equiv.) and propionyl chloride (29 µL, 0.33 mmol, 1.1 equiv.) in isopropyl acetate (0.7 mL) and toluene (0.3 mL). Purification by flash column chromatography (EtOAc/Pentane, *v/v* = 1:9) afforded sulfonamide **1ah** as a colourless solid (70 mg, 0.18 mmol, 61%).

MP: 113-114 °C.

FT IR (neat) υ (cm^{-1}) = 2976, 2940, 1700, 1347, 1191, 1160, 970, 739, 598, 576.

^{1}H NMR (300 MHz, $CDCl_3$, 300 K): δ_H (ppm) = 8.22 (dd, J = 8.0, 1.6 Hz, 1H), 8.14 (dd, J = 7.8, 1.2 Hz, 1H), 7.57 (ddd, J = 8.0, 7.4, 1.2 Hz, 1H), 7.31 – 7.25 (m, 1H), 4.00 (hept, J = 6.8 Hz, 1H), 2.95 (q, J = 7.3 Hz, 2H), 1.34 (d, J = 6.8 Hz, 6H), 1.16 (t, J = 7.3 Hz, 3H).

^{13}C NMR (75 MHz, $CDCl_3$, 300 K): δ_C (ppm) = 175.3 C_q), 143.1 *C*H), 141.3 (C_q), 134.2 (*C*H), 131.9 (*C*H), 128.5 (*C*H), 92.9 (C_q), 53.5 (*C*H), 33.3 (CH_2), 19.9 (CH_3), 9.0 (CH_3).

HRMS (ESI) *m/z* = 403.9788 calcd. for $[C_{12}H_{16}NO_3SINa]^+$ $[M+Na]^+$, found 403.9785.

***N*-((2-Iodophenyl)sulfonyl)-*N*-isopropyl-2,2-diphenylacetamide (1ai):**

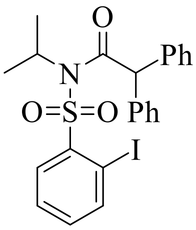

The title compound was prepared following general procedure **GP5** using sulfonamide **S-18** (163 mg, 0.500 mmol, 1.0 equiv.), DMAP (0.5 mg, 4 µmol, 1 mol%), NEt_3 (175 µL, 1.25 mmol, 2.5 equiv.) and 2,2-diphenylacetyl chloride (127 mg, 0.550 mmol, 1.1 equiv.) in isopropyl acetate (1.2 mL) and toluene (0.35 mL). Purification by flash column chromatography (Et_2O/Pentane, *v/v* = 1:4) afforded sulfonamide **1ai** as a colourless solid (160 mg, 0.308 mmol, 62%).

MP: 96-97 °C.

FT IR (neat) υ (cm^{-1}) = 3062, 3030, 2977, 1698, 1452, 1350, 1185, 1152, 1091, 1015, 977, 739, 728, 701, 597, 578.

^{1}H NMR (300 MHz, $CDCl_3$, 300 K): δ_H (ppm) = 8.12 (dd, J = 8.0, 1.6 Hz, 1H), 8.06 (dd, J = 7.8, 1.2 Hz, 1H), 7.51 – 7.45 (m, 1H), 7.32 – 7.21 (m, 11H), 6.10 (s, 1H), 4.18 (hept, J = 6.9 Hz, 1H), 1.39 (d, J = 6.9 Hz, 6H).

^{13}C NMR (75 MHz, $CDCl_3$, 300 K): δ_C (ppm) = 173.6 (C_q), 143.1 (*C*H), 141.7 (C_q), 138.3 (C_q), 134.1 (*C*H), 131.5 (*C*H), 129.2 (*C*H), 128.5 (*C*H), 128.4 (*C*H), 127.2 (*C*H), 92.9 (C_q), 58.7 (*C*H), 54.4 (*C*H), 20.1 (CH_3).

HRMS (ESI) *m/z* = 542.0257 calcd. for $[C_{23}H_{22}NO_3SINa]^+$ $[M+Na]^+$, found 542.0257.

6.5 Synthesis of α-arylated amides

***N*-Isopropyl-2-methyl-2-phenylpropanamide (2a):**

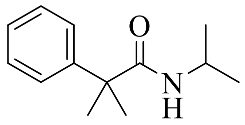

The title compound was prepared following method B of general procedure **GP6** using *N*-acylated sulfonamide **1a** (39.5 mg, 100 µmol, 1.0 equiv.) in MeCN (1.0 mL). Purification by preparative TLC (Et_2O/Pentane, *v/v* = 3:7) afforded the desired amide **2a** as a colourless oil (16.9 mg, 0.082 mmol, 82%).

FT IR (neat) υ (cm^{-1}) = 3343, 2971, 2933, 2874, 1639, 1521, 1497, 1457, 1365, 1182, 1131, 1102, 765, 698, 561.

^{1}H NMR (600 MHz, $CDCl_3$, 300 K): δ_H (ppm) = 7.36 – 7.29 (m, 4H), 7.28 – 7.26 (m, 1H), 4.90 (s, 1H), 4.08 – 4.00 (m, 1H), 1.56 (s, 6H), 1.02 (d, J = 6.5 Hz, 6H).

^{13}C NMR (151 MHz, $CDCl_3$, 300 K): δ_C (ppm) = 176.5 (C_q), 145.4 (C_q), 128.6 (*C*H), 126.9 (*C*H), 126.4 (*C*H), 46.9 (C_q), 41.4 (*C*H), 27.1 (CH_3), 22.5 (CH_3).

HRMS (ESI) *m/z* = 228.1359 calcd. for $[C_{13}H_{19}NONa]^+$ $[M+Na]^+$, found 228.1381.

***N*-Cyclohexyl-2-methyl-2-phenylpropanamide (2b):**

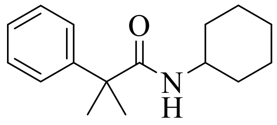

The title compound was prepared following method A of general procedure **GP6** using *N*-acylated sulfonamide **1b** (43.5 mg, 0.100 mmol, 1.0 equiv.), *fac*-$[Ir(ppy)_3]$ (0.8 mg, 1 µmol, 1 mol%) and Cs_2CO_3 (97.7 mg, 0.300 mmol, 3.0 equiv.) in MeCN (1.0 mL). Purification by reverse-phase-MPLC (MeOH/water, gradient from 5% to 90%) afforded the desired amide **2b** as a colourless solid (20.9 mg, 0.085 mmol, 85%).

MP: 90-91 °C.

FT IR (neat) υ (cm^{-1}) = 3298, 2928, 2852, 1637, 1531, 1495, 1447, 763, 697.

^{1}H NMR (300 MHz, $CDCl_3$, 300 K): δ_H (ppm) = 7.37 – 7.30 (m, 4H), 7.29 – 7.25 (m, 1H), 4.96 (s, 1H), 3.79 – 3.67 (m, 1H), 1.81 – 1.76 (m, 2H), 1.61 – 1.52 (m, 8H), 1.39 – 1.24 (m, 2H), 1.18 – 0.88 (m, 4H).

^{13}C NMR (75 MHz, $CDCl_3$, 300 K): δ_C (ppm) = 176.5 (C_q), 145.5 (C_q), 128.6 (*C*H), 126.9 (*C*H), 126.4 (*C*H), 48.1 (*C*H), 47.0 (C_q), 32.8 (CH_2), 27.1 (CH_2), 25.5 (CH_2), 24.7 (CH_3).

HRMS (ESI) *m/z* = 268.1672 calcd. for $[C_{16}H_{23}NONa]^+$ $[M+Na]^+$, found 268.1669.

(*S*)-2-Methyl-2-phenyl-*N*-(1-phenylethyl)propanamide (2c):

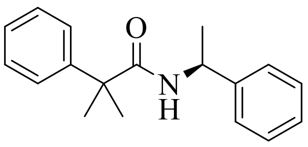

The title compound was prepared following method A of the general procedure **GP6** using *N*-acylated sulfonamide **1c** (55.1 mg, 0.120 mmol, 1.0 equiv.), *fac*-$[Ir(ppy)_3]$ (0.8 mg, 1.2 µmol, 1 mol%) and Cs_2CO_3 (118 mg, 0.360 mmol, 3.0 equiv.) in MeCN (1.2 mL). Purification by reverse-phase-MPLC (MeOH/water, gradient from 5% to 90%) afforded the desired amide **2c** as a colourless solid (24.1 mg, 0.090 mmol, 75%).

^{1}H NMR (300 MHz, $CDCl_3$, 300 K): δ_H (ppm) = 7.37 – 7.31 (m, 4H), 7.29 – 7.22 (m, 4H), 7.15 – 7.12 (m, 2H), 5.32 (d, *J* = 7.8 Hz, 1H), 5.13 – 5.04 (m, 1H), 1.60 (s, 3H), 1.56 (s, 3H), 1.34 (d, *J* = 6.9 Hz, 3H).

^{13}C NMR (75 MHz, $CDCl_3$, 300 K): δ_C (ppm) = 176.4 (C_q), 145.2 (C_q), 143.4 (C_q), 128.7 (*C*H), 128.5 (*C*H), 127.1 (*C*H), 127.0 (*C*H), 126.4 (*C*H), 125.9 (*C*H), 48.7 (*C*H), 47.0 (C_q), 27.1 (CH_3), 27.0 (CH_3), 21.6 (CH_3).

HRMS (ESI) *m/z* = 290.1515 calcd. for $[C_{18}H_{21}NONa]^+$ $[M+Na]^+$, found 290.1511.

The analytical data are consistent with those reported in literature.[21]

***N*,2-Dimethyl-2-phenylpropanamide (2d):**

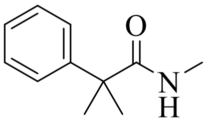

The title compound was prepared following method A of the general procedure **GP6** using *N*-acylated sulfonamide **1d** (73.4 mg, 0.200 mmol, 1.0 equiv.), *fac*-$[Ir(ppy)_3]$ (1.3 mg, 2.0 µmol, 1 mol%) and Cs_2CO_3 (196 mg, 0.600 mmol, 3.0 equiv.) in MeCN (2 mL). Separation by reverse-phase-MPLC (MeCN/water,

gradient from 5% to 90%) afforded the desired amide **2d** as a colourless solid (27.3 mg, 0.154 mmol, 77%).

^{1}H NMR (600 MHz, $CDCl_3$, 300 K): δ_H (ppm) = 7.39 – 7.35 (m, 4H), 7.29 – 7.26 (m, 1H), 5.16 (s, 1H), 2.72 (d, *J* = 4.9 Hz, 3H), 1.58 (s, 6H).

^{13}C NMR (151 MHz, $CDCl_3$, 300 K): δ_C (ppm) = 178.0 (C_q), 145.2 (C_q), 128.7 (*C*H), 126.9 (*C*H), 126.5 (*C*H), 46.9 (C_q), 27.1 (CH_3), 26.6 (CH_3).

HRMS (ESI) *m/z* = 200.1046 calcd. for $[C_{11}H_{15}NONa]^+$ $[M+Na]^+$, found 200.1045.

The analytical data are consistent with those reported in literature.[18]

***N*-Benzyl-2-methyl-2-phenylpropanamide (2e):**

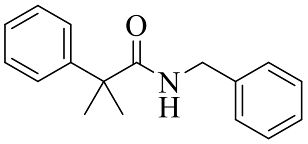

The title compound was prepared following method B of the general procedure **GP6** using *N*-acylated sulfonamide **1e** (35.4 mg, 80.0 µmol, 1.0 equiv.) in MeCN (0.8 mL). Purification by reverse-phase-MPLC (MeCN/water, gradient from 5% to 90%) afforded the desired amide **2e** as a colourless solid (11.7 mg, 0.046 mmol, 58%).

^{1}H NMR (600 MHz, $CDCl_3$, 300 K): δ_H (ppm) = 7.40 – 7.33 (m, 4H), 7.30 – 7.22 (m, 4H), 7.14 – 7.12 (m, 2H), 5.46 (s, 1H), 4.39 (d, *J* = 5.8 Hz, 2H), 1.62 (s, 6H).

^{13}C NMR (151 MHz, $CDCl_3$, 300 K): δ_C (ppm) = 177.3 (C_q), 145.0 (C_q), 138.5 (C_q), 128.7 (*C*H), 128.5 (*C*H), 127.3 (*C*H), 127.2 (*C*H), 127.0 (*C*H), 126.4 (*C*H), 47.0 (C_q), 43.6 (CH_2), 27.1 (CH_3).

HRMS (ESI) *m/z* = 276.1359 calcd. for $[C_{17}H_{19}NONa]^+$ $[M+Na]^+$, found 276.1357.

The analytical data are consistent with those reported in literature.[19]

2-Methyl-*N*,2-diphenylpropanamide (2f):

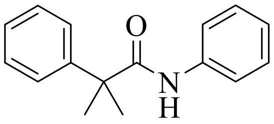

The title compound was prepared following method A of the general procedure **GP6** using *N*-acylated sulfonamide **1f** (64.4 mg, 0.15 mmol, 1.0 equiv.), *fac*-[Ir(ppy)$_3$] (1.0 mg, 1.5 µmol, 1 mol%) and Cs_2CO_3 (147 mg, 0.45 mmol, 3.0 equiv.) in MeCN (1.6 mL). Purification by reverse-phase-MPLC (MeCN/water, gradient from 5% to 90%) afforded the desired amide **2f** as a colourless solid (11.8 mg, 0.049 mmol, 33%).

^{1}H NMR (600 MHz, $CDCl_3$, 300 K): δ_H (ppm) = 7.47 – 7.40 (m, 4H), 7.37 – 7.31 (m, 3H), 7.28 – 7.25 (m, 2H), 7.08 – 7.05 (m, 1H), 6.78 (s, 1H), 1.68 (s, 6H).

^{13}C NMR (151 MHz, $CDCl_3$, 300 K): δ_C (ppm) = 176.3 (C_q), 144.6 (C_q), 138.0 (C_q), 129.0 (*C*H), 128.9 (*C*H), 127.4 (*C*H), 126.5 (*C*H), 124.1 (*C*H), 119.6 (*C*H), 48.1 (C_q) 27.0 (CH_3).

HRMS (ESI) *m/z* = 262.1202 calcd. for $[C_{16}H_{17}NONa]^+$ $[M+Na]^+$, found 262.1199.

The analytical data are consistent with those reported in literature.[20]

***N*-Isopropyl-2-methyl-2-(m-tolyl)propanamide (2i):**

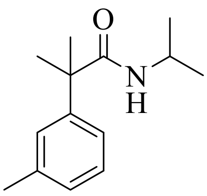

The title compound was prepared following method A of the general procedure **GP6** using *N*-acylated sulfonamide **1i** (102 mg, 0.250 mmol, 1.0 equiv.), *fac*-[Ir(ppy)$_3$] (1.6 mg, 2.5 µmol, 1 mol%) and Cs_2CO_3 (244 mg, 0.750 mmol, 3.0 equiv.) in MeCN (2.5 mL). Purification by reverse-phase-MPLC (MeCN/water, gradient from 5% to 90%) afforded the desired amide **2i** as a colourless solid (32.4 mg, 0.148 mmol, 59%).

MP: 69-70 °C.

FT IR (neat) υ (cm^{-1}) = 3338, 2975, 1653, 1507, 1457, 1180, 908, 731, 646.

^{1}H NMR (300 MHz, $CDCl_3$, 300 K): δ_H (ppm) = 7.25 (d, J = 8.2 Hz, 1H), 7.22 – 7.13 (m, 2H), 7.10 –7.07 (m, 1H), 4.92 (s, 1H), 4.12 – 3.96 (m, 1H), 2.36 (s, 3H), 1.54 (s, 6H), 1.02 (d, *J* = 6.6 Hz, 6H).

^{13}C NMR (75 MHz, $CDCl_3$, 300 K): δ_C (ppm) = 176.7 (C_q), 145.5 (C_q), 138.1 (C_q), 128.5 (*C*H), 127.6 (*C*H), 127.1 (*C*H), 123.4 (*C*H), 46.8 (C_q), 41.4 (*C*H), 27.2 (CH_3), 22.5 (CH_3), 21.6 (CH_3).

HRMS (ESI) *m/z* = 242.1526 calcd. for $[C_{14}H_{21}NONa]^+$ $[M+Na]^+$, found 242.1514.

***N*-Isopropyl-2-methyl-2-(p-tolyl)propenamide (2j):**

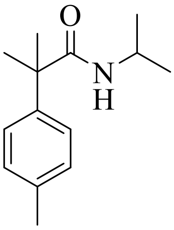

The title compound was prepared following method A of general procedure **GP6** using *N*-acylated sulfonamide **1j** (81.9 mg, 0.200 mmol, 1.0 equiv.), *fac*-[Ir(ppy)$_3$] (1.4 mg, 2.0 µmol, 1 mol%) and Cs_2CO_3 (196 mg, 0.600 mmol, 3.0 equiv.) in MeCN (2.0 mL). Purification by reverse-phase-MPLC (MeCN/water, gradient from 5% to 90%) afforded the desired amide **2j** as a colourless solid (27.6 mg, 0.101 mmol, 50%).

MP: 71°C.

FT IR (neat) υ (cm^{-1}) = 3339, 2971, 2930, 1639, 1513, 1455, 1365, 1181, 1098, 816, 614, 597, 578, 553.

^{1}H NMR (300 MHz, $CDCl_3$, 300 K): δ_H (ppm) = 7.24 (d, J = 8.3 Hz, 2H), 7.16 (d, J = 8.0 Hz, 2H), 4.92 (s, 1H), 4.08 – 3.95 (m, 1H), 2.35 (s, 3H), 1.54 (s, 6H), 1.02 (d, J = 6.6 Hz, 6H).

^{13}C NMR (75 MHz, $CDCl_3$, 300 K): δ_C (ppm) = 176.7 (C_q), 142.4 (C_q), 136.5 (C_q), 129.3 (CH), 126.3 (CH), 46.6 (C_q), 41.3 (CH), 27.2 (CH$_3$), 22.5 (CH$_3$), 20.9 (CH$_3$).

HRMS (ESI) *m/z* = 242.1515 calcd. for $[C_{14}H_{21}NONa]^+$ $[M+Na]^+$, found 242.1514.

***N*-Isopropyl-2-(3-methoxyphenyl)-2-methylpropanamide (2k):**

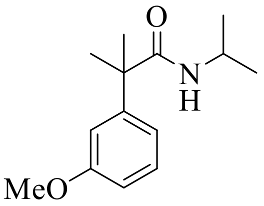

The title compound was prepared following method A of general procedure **GP6** using *N*-acylated sulfonamide **1k** (106 mg, 0.250 mmol, 1.0 equiv.), *fac*-[Ir(ppy)$_3$] (1.6 mg, 2.5 μmol, 1 mol%) and Cs_2CO_3 (244 mg, 0.750 mmol, 3.0 equiv.) in MeCN (2.5 mL). The reaction time was extended to 36 h. Separation by reverse-phase-MPLC (MeCN/water, gradient from 5% to 90%) afforded the desired amide **2k** as a colourless solid (34.2 mg, 0.145 mmol, 66%).

MP: 47-48 °C.

FT IR (neat) υ (cm^{-1}) = 3299, 2973, 2938, 1642, 1607, 1583, 1531, 1047, 781, 702.

^{1}H NMR (300 MHz, $CDCl_3$, 300 K): δ_H (ppm) = 7.30 – 7.25 (m, 1H), 6.95 – 6.89 (m, 2H), 6.83 – 6.79 (m, 1H), 4.93 (s, 1H), 4.09 – 3.98 (m, 1H), 3.81 (s, 3H), 1.54 (s, 6H), 1.02 (d, J = 6.6 Hz, 6H).

^{13}C NMR (75 MHz, $CDCl_3$, 300 K): δ_C (ppm) = 176.4 (C_q), 159.7 (C_q), 147.2 (C_q), 129.6 (CH), 118.8 (CH), 112.7 (CH), 111.8 (CH), 55.2 (CH$_3$), 47.0 (C_q), 41.4 (CH), 27.1 (CH$_3$), 22.5 (CH$_3$).

HRMS (ESI) *m/z* = 258.1465 calcd. for $[C_{14}H_{21}NO_2Na]^+$ $[M+Na]^+$, found 258.1462.

2-(3-Chlorophenyl)-*N*-isopropyl-2-methylpropanamide (2l):

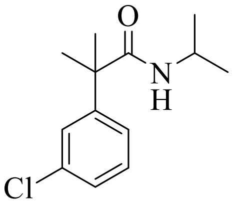

The title compound was prepared following method A of general procedure **GP6** using *N*-acylated sulfonamide **1l** (107 mg, 0.250 mmol, 1.0 equiv.), *fac*-[Ir(ppy)$_3$] (1.6 mg, 2.5 μmol, 1 mol%) and Cs_2CO_3 (244 mg, 0.750 mmol, 3.0 equiv.) in MeCN (2.5 mL). The reaction time was extended to 36 h. Separation by reverse-phase-MPLC (MeCN/water, gradient from 5% to 90%) afforded the desired amide **2l** as a colourless solid (47.5 mg, 0.198 mmol, 79%).

MP: 46-47 °C.

FT IR (neat) υ (cm^{-1}) = 3338, 2973, 2935, 1639, 1596, 1527, 1467, 1366, 1181, 786, 699.

^{1}H NMR (300 MHz, $CDCl_3$, 300 K): δ_H (ppm) = 7.35 – 7.21 (m, 4H), 4.91 (s, 1H), 4.12 – 3.97 (m, 1H), 1.54 (s, 6H), 1.05 (d, *J* = 6.5 Hz, 6H).

^{13}C NMR (75 MHz, $CDCl_3$, 300 K): δ_C (ppm) = 175.6 (C_q), 147.7 (C_q), 134.5 (C_q), 129.9 (*C*H), 127.1 (*C*H), 126.5 (*C*H), 124.7 (*C*H), 46.9 (C_q), 41.5 (*C*H), 27.0 (CH_3), 22.5 (CH_3).

HRMS (ESI) *m/z* = 262.0969 calcd. for $[C_{13}H_{18}NOClNa]^+$ $[M+Na]^+$, found 262.0969.

***N*-Isopropyl-2-methyl-2-(4-(trifluoromethyl)phenyl)propenamide (2m):**

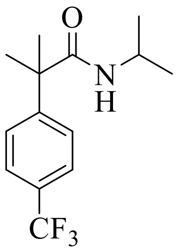

The title compound was prepared following method A of the general procedure **GP6** using *N*-acylated sulfonamide **1m** (92.7 mg, 0.200 mmol, 1.0 equiv.), *fac*-$[Ir(ppy)_3]$ (1.4 mg, 2.0 µmol, 1 mol%) and Cs_2CO_3 (196 mg, 0.600 mmol, 3.0 equiv.) in MeCN (2.0 mL). Purification by reverse-phase-MPLC (MeCN/water, gradient from 5% to 90%) afforded the desired amide **2m** as a colourless solid (25.9 mg, 0.095 mmol, 47%).

MP: 64-65 °C.

FT IR (neat) υ (cm^{-1}) = 3334, 2973, 2932, 1639, 1528, 1515, 1325, 1164, 1122, 1101, 1067, 1017, 840, 659, 608.

^{1}H NMR (600 MHz, $CDCl_3$, 300 K): δ_H (ppm) = 7.62 – 7.61 (m, 2H), 7.49 – 7.47 (m, 2H), 4.91 (s, 1H), 4.11 – 4.01 (m, 1H), 1.57 (s, 5H), 1.06 (d, *J* = 6.5 Hz, 5H).

^{13}C{^{19}F} NMR (151 MHz, $CDCl_3$, 300 K): δ_C (ppm) = 175.3 (C_q), 149.6 (C_q), 129.2 (C_q), 126.7 (*C*H), 125.6 (*C*H), 124.1 (C_q), 47.0 (C_q), 41.6 (*C*H), 27.0 (CH_3), 22.5 (CH_3).

^{19}F{^{1}H} NMR (470 MHz, $CDCl_3$, 300 K): δ_F (ppm) = −62.6.

HRMS (ESI) *m/z* = 296.1233 calcd. for $[C_{14}H_{18}NOF_3Na]^+$ $[M+Na]^+$, found 296.1230.

2-(3-Chloro-4-methylphenyl)-*N*-isopropyl-2-methylpropanamide (2n):

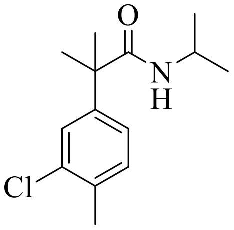

The title compound was prepared following method A of the general procedure **GP6** using *N*-acylated sulfonamide **1n** (111 mg, 0.250 mmol, 1.0 equiv.), *fac*-$[Ir(ppy)_3]$ (1.6 mg, 2.5 µmol, 1 mol%) and Cs_2CO_3 (244 mg, 0.750 mmol, 3.0 equiv.) in MeCN (2.5 mL). Purification by

reverse-phase-MPLC (MeCN/water, gradient from 5% to 90%) afforded the desired amide **2n** as a colourless solid (40.8 mg, 0.161 mmol, 64%).

MP: 60-61 °C.

FT IR (neat) υ (cm^{-1}) = 3353, 2972, 2934, 1639, 1507, 1481, 1386, 1365, 1180, 1047, 906, 819, 728.

^{1}H NMR (300 MHz, $CDCl_3$, 300 K): δ_H (ppm) = 7.32 – 7.29 (m, 1H), 7.20 (d, *J* = 2.4 Hz, 1H), 7.10 (dd, *J* = 8.3, 2.4 Hz, 1H), 4.91 (s, 1H), 4.09 – 3.96 (m, 1H), 2.38 (s, 3H), 1.52 (s, 6H), 1.04 (d, *J* = 6.6 Hz, 6H).

^{13}C NMR (75 MHz, $CDCl_3$, 300 K): δ_C (ppm) = 176.0 (C_q), 144.1 (C_q), 136.1 (C_q), 132.9 (C_q), 129.1 (*C*H), 129.0 (*C*H), 125.1 (*C*H), 46.5 (C_q), 41.5 (*C*H), 27.1 (CH_3), 22.5 (CH_3), 20.2 (CH_3).

HRMS (ESI) *m/z* = 276.1137 calcd. for $[C_{14}H_{20}NOClNa]^+$ $[M+Na]^+$, found 276.1125.

2-(3,4-Dichlorophenyl)-*N*-isopropyl-2-methylpropanamide (2o):

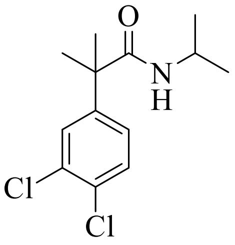

The title compound was prepared following method A of the general procedure **GP6** using *N*-acylated sulfonamide **1o** (116 mg, 0.250 mmol, 1.0 equiv.), *fac*-$[Ir(ppy)_3]$ (1.6 mg, 2.5 µmol, 1 mol%) and Cs_2CO_3 (244 mg, 0.750 mmol, 3.0 equiv.) in MeCN (2.5 mL). Purification by reverse-phase-MPLC (MeCN/water, gradient from 5% to 90%) afforded the desired amide **2o** as a colourless oil (49.6 mg, 0.181 mmol, 72%).

FT IR (neat) υ (cm^{-1}) = 3338, 2973, 2933, 1640, 1526, 1508, 1471, 1180, 1141, 1030, 908, 811, 731, 710.

^{1}H NMR (300 MHz, $CDCl_3$, 300 K): δ_H (ppm) = 7.44 – 7.39 (m, 2H), 7.17 (dd, *J* = 8.5, 2.3 Hz, 1H), 4.96 (s, 1H), 4.12 – 3.96 (m, 1H), 1.52 (s, 6H), 1.06 (d, *J* = 6.6 Hz, 6H).

^{13}C NMR (75 MHz, $CDCl_3$, 300 K): δ_C (ppm) = 175.0 (C_q), 146.0 (C_q), 132.7 (C_q), 131.0 (C_q), 130.5 (*C*H), 128.3 (*C*H), 126.0 (*C*H), 46.5 (C_q), 41.7 (*C*H), 27.0 (CH_3), 22.5 (CH_3).

HRMS (ESI) *m/z* = 296.0590 calcd. for $[C_{13}H_{17}NOCl_2Na]^+$ $[M+Na]^+$, found 296.0580.

2-(3,5-Dimethylphenyl)-*N*-isopropyl-2-methylpropanamide (2p):

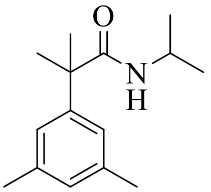

The title compound was prepared following method A of the general procedure **GP6** using *N*-acylated sulfonamide **1p** (84.7 mg, 0.200 mmol, 1.0 equiv.), *fac*-[Ir(ppy)$_3$] (1.4 mg, 2.0 µmol, 1 mol%) and Cs_2CO_3 (196 mg, 0.600 mmol, 3.0 equiv.) in MeCN (2.0 mL). Purification by reverse-phase-MPLC (MeCN/water, gradient from 5% to 90%) afforded the desired amide **2p** as a colourless solid (28.8 mg, 0.123 mmol, 62%).

MP: 88-89 °C.

FT IR (neat) υ (cm^{-1}) = 3298, 2971, 2929, 1643, 1602, 1508, 1457, 1386, 1363, 1265, 1181, 848, 734, 707, 653.

^{1}H NMR (300 MHz, $CDCl_3$, 300 K): δ_H (ppm) = 6.95 – 6.94 (m, 2H), 6.90 (s, 1H), 4.94 (s, 1H), 4.12 – 3.96 (m, 1H), 2.32 (s, 6H), 1.52 (s, 6H), 1.03 (d, *J* = 6.6 Hz, 6H).

^{13}C NMR (75 MHz, $CDCl_3$, 300 K): δ_C (ppm) = 176.8 (C_q), 145.5 (C_q), 138.0 (C_q), 128.4 (*C*H), 124.2 (*C*H), 46.7 (C_q), 41.3 (*C*H), 27.2 (CH_3), 22.5 (CH_3), 21.4 (CH_3).

HRMS (ESI) *m/z* = 256.1672 calcd. for $[C_{15}H_{23}NONa]^+$ $[M+Na]^+$, found 256.1670.

***N*-Isopropyl-2-methyl-2-(naphthalen-1-yl)propenamide (2q):**

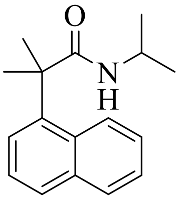

The title compound was prepared following method A of general procedure **GP6** using *N*-acylated sulfonamide **1q** (89.1 mg, 0.200 mmol, 1.0 equiv.), *fac*-[Ir(ppy)$_3$] (1.4 mg, 2.0 µmol, 1 mol%) and Cs_2CO_3 (196 mg, 0.600 mmol, 3.0 equiv.) in MeCN (2.0 mL). Purification by reverse-phase-MPLC (MeCN/water, gradient from 5% to 90%) afforded the desired amide **2q** as a colourless oil (37.5 mg, 0.147 mmol, 73%).

FT IR (neat) υ (cm^{-1}) = 3419, 2973, 1656, 1059, 1458, 1178, 905, 805, 780, 724, 646.

^{1}H NMR (600 MHz, $CDCl_3$, 300 K): δ_H (ppm) = 7.95 – 7.92 (m, 1H), 7.88 – 7.85 (m, 1H), 7.82 – 7.80 (m, 1H), 7.60 (dd, *J* = 7.3, 1.2 Hz, 1H), 7.48 – 7.45 (m, 3H), 4.85 (d, *J* = 8.1 Hz, 1H), 4.07 – 4.01 (m, 1H), 1.73 (s, 6H), 0.84 (d, *J* = 6.6 Hz, 6H).

^{13}C NMR (151 MHz, $CDCl_3$, 300 K): δ_C (ppm) = 177.5 (C_q), 140.8 (C_q), 134.5 (C_q), 131.0 (C_q), 129.0 (*C*H), 128.6 (*C*H), 125.8 (*C*H), 125.6 (*C*H), 125.6 (*C*H), 125.1 (*C*H), 123.9 (*C*H), 46.9 (C_q), 41.3 (*C*H), 27.9 (CH_3), 22.2 (CH_3).

HRMS (ESI) *m/z* = 278.1515 calcd. for $[C_{17}H_{21}NONa]^+$ $[M+Na]^+$, found 278.1512.

2-(5-(Dimethylamino)naphthalen-1-yl)-*N*-isopropyl-2-methylpropanamide (2r):

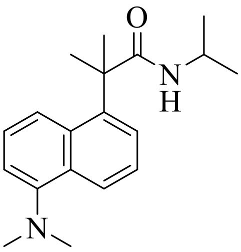

The title compound was prepared following method A of the general procedure **GP6** using *N*-acylated sulfonamide **1r** (117 mg, 0.240 mmol, 1.0 equiv.), *fac*-$[Ir(ppy)_3]$ (1.6 mg, 2.4 µmol, 1 mol%) and Cs_2CO_3 (234 mg, 0.719 mmol, 3.0 equiv.) in MeCN (2.4 mL). Purification by reverse-phase-MPLC (MeOH/water, gradient from 5% to 90%) afforded the desired amide **2r** as a yellow oil (48.6 mg, 0.163 mmol, 68%).

FT IR (neat) υ (cm^{-1}) = 3418, 3322, 2972, 2939, 2871, 2828, 2787, 1653, 1593, 1508, 1454, 1409, 1384, 1306, 1177, 1145, 1049, 954, 923, 909, 787, 729, 645, 593.

^{1}H NMR (300 MHz, $CDCl_3$, 300 K): δ_H (ppm) = 8.31 – 8.28 (m, 1H), 7.61 – 7.57 (m, 2H), 7.49 – 7.44 (m, 1H), 7.39 – 7.33 (m, 1H), 7.09 – 7.07 (m, 1H), 4.87 (d, *J* = 8.2 Hz, 1H), 4.11 – 3.97 (m, 1H), 2.89 (s, 6H), 1.71 (s, 6H), 0.85 (d, J = 6.6 Hz, 6H).

^{13}C NMR (75 MHz, $CDCl_3$, 300 K): δ_C (ppm) = 177.7 (C_q), 155.0 (C_q), 141.0 (C_q), 132.4 (C_q), 130.0 (C_q), 125.6 (*C*H), 124.5 (*C*H), 124.4 (*C*H), 123.9 (*C*H), 120.7 (*C*H), 114.0 (*C*H), 47.1 (C_q), 45.5 (CH_3), 41.4 (*C*H), 28.1 (CH_3), 22.2 (CH_3).

HRMS (ESI) *m/z* = 321.1937 calcd. for $[C_{19}H_{26}N_2ONa]^+$ $[M+Na]^+$, found 321.1935.

2-(3,5-Bis(trifluoromethyl)phenyl)-*N*,2-dimethylpropanamide (2s):

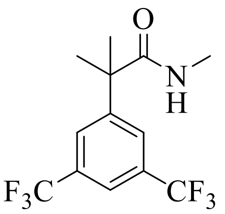

The title compound was prepared following method A of the general procedure **GP6** using *N*-acylated sulfonamide **1s** (43.0 mg, 0.078 mmol, 1.0 equiv.), *fac*-$[Ir(ppy)_3]$ (0.5 mg, 0.8 µmol, 1 mol%) and Cs_2CO_3 (76.4 mg, 0.234 mmol, 3.0 equiv.) in MeCN (0.8 mL). Purification by reverse-phase-MPLC (MeOH/water, gradient from 5% to 90%) afforded the desired amide **2s** as a colourless oil (13.7 mg, 0.044 mmol, 56%).

FT IR (neat) υ (cm^{-1}) = 3334, 2924, 1709, 1650, 1540, 1372, 1276, 1232, 1180, 1132, 897, 845, 726, 706, 683.

^{1}H NMR (600 MHz, $CDCl_3$, 300 K): δ_H (ppm) = 7.82 (d, *J* = 1.6 Hz, 2H), 7.80 (t, *J* = 1.6 Hz, 1H), 5.38 – 5.23 (m, 1H), 2.81 (d, *J* = 4.7 Hz, 3H), 1.64 (s, 6H).

^{13}C{^{19}F} NMR (151 MHz, $CDCl_3$, 300 K): δ_C (ppm) = 175.7 (C_q), 148.1 (C_q), 131.9 (C_q), 126.5 (*C*H), 123.2 (*C*H), 121.1 (C_q), 47.0 (C_q), 26.9 (CH_3), 26.9 (CH_3).

^{19}F{^{1}H} NMR (470 MHz, $CDCl_3$, 300 K): δ_F (ppm) = −62.8.

HRMS (ESI) *m/z* = 336.0794 calcd. for $[C_{13}H_{13}NOF_6Na]^+$ $[M+Na]^+$, found 333.0793.

***N*-Isobutyryl-*N*,2-diisopropyl-4,4-dimethyl-3-oxo-3,4-dihydro-2H-benzo[e][1,2]thiazine-7-carboxamide 1,1-dioxide (2’v):**

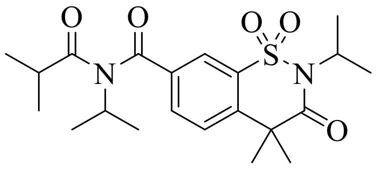

The title compound was prepared following method A of the general procedure **GP6** using *N*-acylated sulfonamide **1v** (43.0 mg, 0.078 mmol, 1.0 equiv.), *fac*-[Ir(ppy)$_3$] (0.5 mg, 0.8 µmol, 1 mol%) and Cs_2CO_3 (76.4 mg, 0.234 mmol, 3.0 equiv.) in MeCN (0.8 mL). Purification by reverse-phase-MPLC (MeCN/water, gradient from 5% to 90%) afforded the desired cyclic sulfonamide **2’v** as a colourless oil (25.0 mg, 59.2 µmol, 76%).

FT IR (neat) υ (cm^{-1}) = 2978, 2938, 2976, 2255, 1729, 1700, 1386, 1354, 1314, 1224, 1155, 1087, 977, 906, 728, 691, 639.

^{1}H NMR (600 MHz, $CDCl_3$, 300 K): δ_H (ppm) = 8.05 (dd, *J* = 8.0, 0.8 Hz, 1H), 8.02 – 7.99 (m, 2H), 4.58 – 4.50 (m, 1H), 3.98 (hept, *J* = 6.8 Hz, 1H), 3.17 – 3.08 (m, 1H), 1.79 (s, 6H), 1.51 (d, *J* = 6.9 Hz, 6H), 1.23 (d, *J* = 6.8 Hz, 6H), 1.04 (d, *J* = 6.7 Hz, 6H).

^{13}C NMR (151 MHz, $CDCl_3$, 300 K): δ_C (ppm) = 178.9 (C_q), 178.6 (C_q), 165.6 (C_q), 153.7 (C_q), 146.1 (C_q), 132.9 (C_q), 127.2 (*C*H), 126.2 (*C*H), 120.9 (*C*H), 65.6 (C_q), 53.1 (*C*H), 35.8 (*C*H), 34.9 (*C*H), 26.0 (CH_3), 21.3 (CH_3), 19.4 (CH_3), 18.8 (CH_3).

HRMS (ESI) *m/z* = 445.1768 calcd. for $[C_{21}H_{30}N_2O_5SNa]^+$ $[M+Na]^+$, found 445.1763.

***N*-Isopropyl-2-methyl-2-phenylpentanamide (2w):**

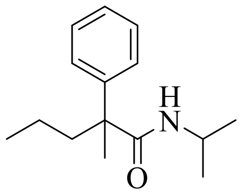

The title compound was prepared following method A of the general procedure **GP6** using *N*-acylated sulfonamide **1w** (63.5 mg, 0.150 mmol, 1.0 equiv.), *fac*-[Ir(ppy)$_3$] (1.0 mg, 1.5 µmol, 1 mol%) and Cs_2CO_3 (147 mg, 0.450 mmol, 1.20 equiv.) in MeCN (1.5 mL). Purification by reverse-phase-MPLC (MeCN/water, gradient from 5% to 90%) afforded the desired amide **2w** as a colourless solid (28.2 mg, 0.121 mmol, 81%).

MP: 78-79 °C.

FT IR (neat) υ (cm^{-1}) = 3339, 2959, 2928, 1643, 1522, 1458, 1176, 699.

^{1}H NMR (600 MHz, $CDCl_3$, 300 K): δ_H (ppm) = 7.38 – 7.30 (m, 4H), 7.29 – 7.22 (m, 1H), 4.90 (s, 1H), 4.11 – 3.99 (m, 1H), 2.03 – 1.86 (m, 2H), 1.51 (s, 3H), 1.29 – 1.21 (m, 2H), 1.03 – 1.01 (m, 6H), 0.90 (t, *J* = 7.3 Hz, 3H).

^{13}C NMR (151 MHz, $CDCl_3$, 300 K): δ_C (ppm) = 175.4 (C_q), 144.6 (C_q), 128.5 (*C*H), 126.8 (*C*H), 126.8 (*C*H), 50.5 (C_q), 41.3 (*C*H), 24.0 (CH_2), 22.5 (CH_3), 22.5 (CH_3), 17.7 (CH_2), 14.6 (CH_3).

HRMS (ESI) *m/z* = 256.1672 calcd. for $[C_{15}H_{23}NONa]^+$ $[M+Na]^+$, found 256.1670.

***N*-Isopropyl-2,3-dimethyl-2-phenylbutanamide (2x):**

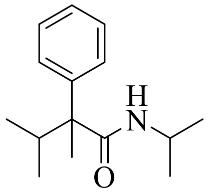

The title compound was prepared following method A of the general procedure **GP6** using *N*-acylated sulfonamide **1x** (58.7 mg, 0.139 mmol, 1.0 equiv.), *fac*-[Ir(ppy)$_3$] (0.9 mg, 1.4 µmol, 1 mol%) and Cs_2CO_3 (136 mg, 0.417 mmol, 3.0 equiv.) in MeCN (1.4 mL). Purification by reverse-phase-MPLC (MeCN/water, gradient from 5% to 90%) afforded the desired amide **2x** as a colourless oil (21.9 mg, 0.094 mmol, 72%).

FT IR (neat) υ (cm^{-1}) = 3374, 2970, 2931, 1638, 1524, 1455, 1367, 1263, 1172, 1130, 732, 699, 623, 566.

^{1}H NMR (600 MHz, $CDCl_3$, 300 K): δ_H (ppm) = 7.40 – 7.38 (m, 2H), 7.34 – 7.31 (m, 2H), 7.25 – 7.23 (m, 1H), 5.09 (s, 1H), 4.07 – 4.01 (m, 1H), 2.68 – 2.63 (m, 1H), 1.46 (s, 3H), 1.03 (dd, *J* = 11.4, 6.5 Hz, 6H), 0.95 (d, *J* = 6.7 Hz, 3H), 0.73 (d, *J* = 6.8 Hz, 3H).

^{13}C NMR (151 MHz, $CDCl_3$, 300 K): δ_C (ppm) = 175.1 (C_q), 143.5 (C_q), 128.2 (*C*H), 127.0 (*C*H), 126.5 (*C*H), 53.4 (C_q), 41.3 (*C*H), 33.9 (*C*H), 22.5 (CH_3), 22.4 (CH_3), 18.7 (CH_3), 18.0 (CH_3), 17.9 (CH_3).

HRMS (ESI) *m/z* = 256.1672 calcd. for $[C_{15}H_{23}NONa]^+$ $[M+Na]^+$, found 256.1667.

***N*-Isopropyl-1-phenylcyclohexane-1-carboxamide (2y):**

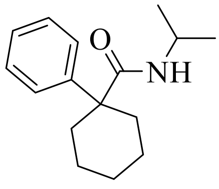

The title compound was prepared following method A of the general procedure **GP6** using *N*-acylated sulfonamide **1y** (65.3 mg, 0.150 mmol, 1.0 equiv.), *fac*-[Ir(ppy)$_3$] (1.0 mg, 1.5 µmol, 1 mol%) and Cs_2CO_3 (147 mg, 0.450 mmol, 3.0 equiv.) in MeCN (1.5 mL). Purification by reverse-phase-MPLC (MeCN/water, gradient from 5% to 90%) afforded the desired amide **2y** as a colourless solid (26.3 mg, 0.107 mmol, 71%).

MP: 111-112 °C.

FT IR (neat) υ (cm^{-1}) = 3329, 2970, 2930, 2861, 1626, 1531, 1498, 1456, 1446, 1366, 1247, 1157, 1131, 910, 731, 695, 627.

^{1}H NMR (300 MHz, $CDCl_3$, 300 K): δ_H (ppm) = 7.42 – 7.33 (m, 4H), 7.28 – 7.23 (m, 1H), 4.98 (s, 1H), 4.04 – 3.97 (m, 1H), 2.29 – 2.21 (m, 2H), 2.01 – 1.92 (m, 2H), 1.73 – 1.50 (m, 6H), 0.99 (d, *J* = 6.6 Hz, 6H).

^{13}C NMR (151 MHz, $CDCl_3$, 300 K): δ_C (ppm) = 175.0 (C_q), 143.9 (C_q), 128.7 (*C*H), 126.7 (*C*H), 126.6 (*C*H), 50.8 (C_q), 41.2 (*C*H), 34.6 (*C*H_2), 25.8 (*C*H_3), 22.9 (*C*H_2), 22.5 (*C*H_2).

HRMS (ESI) *m/z* = 268.1672 calcd. for $[C_{16}H_{23}NONa]^+$ $[M+Na]^+$, found 268.1670.

2-(4-Fluorophenyl)-*N*-isopropyl-2-phenylpropanamide (2z):

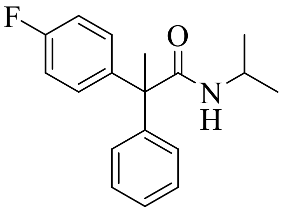

The title compound was prepared following method A of the general procedure **GP6** using *N*-acylated sulfonamide **1z** (69.5 mg, 0.141 mmol, 1.0 equiv.), *fac*-[Ir(ppy)$_3$] (0.9 mg, 1.4 µmol, 1 mol%) and Cs_2CO_3 (138 mg, 0.424 mmol, 3.0 equiv.) in MeCN (1.4 mL). Purification by reverse-phase MPLC (MeCN/water, gradient from 5% to 90%) afforded the desired amide **2z** as a colourless oil (17.4 mg, 61.0 µmol, 43%).

FT IR (neat) υ (cm^{-1}) = 3350, 2975, 2935, 1655, 1508, 1229, 1165, 905, 834, 813, 731, 700, 647, 572.

^{1}H NMR (600 MHz, $CDCl_3$, 300 K): δ_H (ppm) = 7.36 – 7.32 (m, 2H), 7.30 – 7.26 (m, 1H), 7.22 – 7.19 (m, 4H), 7.03 – 7.00 (m, 2H), 5.19 (s, 1H), 4.15 – 4.09 (m, 1H), 1.97 (s, 3H), 1.08 – 1.06 (m, 6H).

^{13}C{^{19}F} NMR (151 MHz, $CDCl_3$, 300 K): δ_C (ppm) = 173.9 (C_q), 161.6 (C_q), 144.9 (C_q), 140.9 (C_q), 129.7 (*C*H), 128.6 (*C*H), 128.0 (*C*H), 127.1 (*C*H), 115.2 (*C*H), 56.3 (C_q), 41.8 (*C*H), 27.2 (*C*H_3), 22.5 (2 x *C*H_3).

^{19}F{^{1}H} NMR (470 MHz, $CDCl_3$, 300 K): δ_C (ppm) = −116.0.

HRMS (ESI) *m/z* = 308.1421 calcd. for $[C_{18}H_{20}NOFNa]^+$ $[M+Na]^+$, found 308.1417.

2-(4-Chlorophenyl)-*N*-isopropyl-2-phenylpropanamide (2aa):

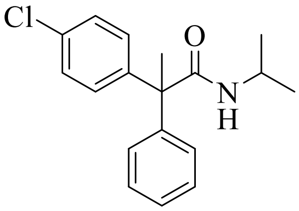

The title compound was prepared following method A of the general procedure **GP6** using *N*-acylated sulfonamide **1aa** (57.1 mg, 0.116 mmol, 1.0 equiv.), *fac*-[Ir(ppy)$_3$] (0.8 mg, 1.2 µmol, 1 mol%) and Cs_2CO_3 (113 mg, 0.347 mmol, 3.0 equiv.) in MeCN (1.2 mL). Purification by reverse-phase MPLC (MeCN/water, gradient from 5% to 90%) afforded the desired amide **2aa** as a colourless oil (21.6 mg, 72.0 µmol, 48%).

FT IR (neat) υ (cm^{-1}) = 3424, 3346, 2973, 2931, 1649, 1492, 1367, 1170, 1095, 1013, 826, 758, 700.

^{1}H NMR (300 MHz, $CDCl_3$, 300 K): δ_H (ppm) = 7.37 – 7.28 (m, 5H), 7.22 – 7.15 (m, 4H), 5.20 (d, *J* = 7.8 Hz, 1H), 4.17 – 4.06 (m, 1H), 1.96 (s, 3H), 1.08 –1.05 (m, 6H).

^{13}C NMR (75 MHz, $CDCl_3$, 300 K): δ_C (ppm) = 173.6 (C_q), 144.6 (C_q), 143.7 (C_q), 132.8 (C_q), 129.5 (*C*H), 128.6 (*C*H), 128.5 (*C*H), 128.0 (*C*H), 127.2 (*C*H), 56.4 (C_q), 41.9 (*C*H), 27.1 (CH_3), 22.5 (2 x CH_3).

HRMS (ESI) *m/z* = 324.1126 calcd. for $[C_{18}H_{20}NOClNa]^+$ $[M+Na]^+$, found 324.1124.

2-(4-Bromophenyl)-*N*-isopropyl-2-phenylpropanamide (2ab):

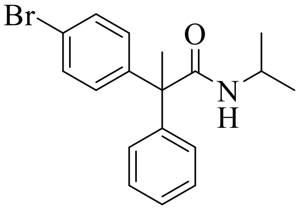

The title compound was prepared following method A of the general procedure **GP6** using *N*-acylated sulfonamide **1ab** (80.4 mg, 0.150 mmol, 1.0 equiv.), *fac*-[Ir(ppy)$_3$] (1.0 mg, 1.5 µmol, 1 mol%) and Cs_2CO_3 (147 mg, 0.450 mmol, 3.0 equiv.) in MeCN (1.5 mL). Purification by reverse-phase MPLC (MeCN/water, gradient from 5% to 90%) afforded the desired amide **2ab** as a colourless oil (32.2 mg, 93.2 µmol, 62%).

FT IR (neat) υ (cm^{-1}) = 3424, 2973, 1658, 1508, 1491, 1386, 1171, 1081, 1009, 906, 821, 729, 700, 647.

^{1}H NMR (300 MHz, $CDCl_3$, 300 K): δ_H (ppm) = 7.47 – 7.43 (m, 2H), 7.37 – 7.30 (m, 3H), 7.22 – 7.19 (m, 2H), 7.14 – 7.10 (m, 2H), 5.20 (s, 1H), 4.19 – 4.04 (m, 1H), 1.96 (s, 3H), 1.08 – 1.05 (m, 6H).

^{13}C NMR (75 MHz, $CDCl_3$, 300 K): δ_C (ppm) = 173.6 (C_q), 144.5 (C_q), 144.3 (C_q), 131.5 (*C*H), 129.9 (*C*H), 128.6 (*C*H), 128.0 (*C*H), 127.2 (*C*H), 120.9 (C_q), 56.5 (C_q), 41.9 (*C*H), 27.0 (CH_3), 22.5 (2 x CH_3).

HRMS (ESI) *m/z* = 368.0621 calcd. for $[C_{18}H_{20}NOBrNa]^+$ $[M+Na]^+$, found 368.0619.

***N*-Isopropyl-2-phenyl-2-(*p*-tolyl)propanamide (2ac):**

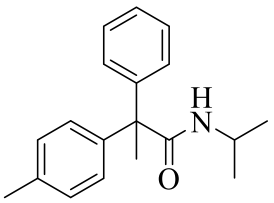

The title compound was prepared following method A of the general procedure **GP6** using *N*-acylated sulfonamide **1ac** (47.1 mg, 0.100 mmol, 1.00 equiv.), *fac*-$[Ir(ppy)_3]$ (0.8 mg, 1 µmol, 1 mol%) and Cs_2CO_3 (97.7 mg, 0.300 mmol, 3.0 equiv.) in MeCN (1.0 mL). Purification by reverse-phase MPLC (MeCN/water, gradient from 5% to 90%) afforded the desired amide **2ac** as a colourless oil (18.0 mg, 64.4 µmol, 64%).

FT IR (neat) υ (cm^{-1}) = 3424, 2973, 2931, 1658, 1511, 1455, 1367, 1171, 1130, 908, 817, 773, 700, 578.

^{1}H NMR (600 MHz, $CDCl_3$, 300 K): δ_H (ppm) = 7.34 – 7.30 (m, 2H), 7.28 – 7.23 (m, 3H), 7.14 – 7.10 (m, 4H), 5.24 (s, 1H), 4.17 – 4.07 (m, 1H), 2.35 (s, 3H), 1.97 (s, 3H), 1.07 – 1.05 (m, 6H).

^{13}C NMR (151 MHz, $CDCl_3$, 300 K): δ_C (ppm) = 174.2 (C_q), 145.3 (C_q), 142.1 (C_q), 136.5 (C_q), 129.1 (*C*H), 128.4 (*C*H), 128.0 (*C*H), 128.0 (*C*H), 126.8 (*C*H), 56.5 (C_q), 41.7 (*C*H), 27.1 (CH_3), 22.5 (CH_3), 22.5 (CH_3), 20.9 (CH_3).

HRMS (ESI) *m/z* = 304.2672 calcd. for $[C_{19}H_{23}NONa]^+$ $[M+Na]^+$, found 304.1670.

2-(4-Isobutylphenyl)-*N*-isopropyl-2-phenylpropanamide (2ad):

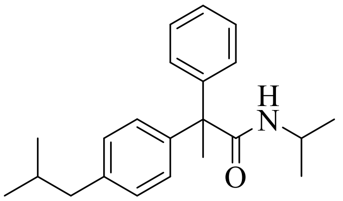

The title compound was prepared following method A of the general procedure **GP6** using *N*-acylated sulfonamide **1ad** (77.0 mg, 0.150 mmol, 1.0 equiv.), *fac*-$[Ir(ppy)_3]$ (1.0 mg, 1.5 µmol, 1 mol%) and Cs_2CO_3 (147 mg, 0.450 mmol, 3.0 equiv.) in MeCN (1.5 mL). Purification by reverse-phase MPLC (MeCN/water, gradient from 5% to 90%) afforded the desired amide **2ad** as a colourless oil (27.2 mg, 97.1 µmol, 64%).

FT IR (neat) υ (cm^{-1}) = 3424, 2973, 1658, 1508, 1491, 1171, 1009, 906, 729, 700, 647, 578.

^{1}H NMR (300 MHz, $CDCl_3$, 300 K): δ_H (ppm) = 7.38 – 7.21 (m, 6H), 7.16 – 7.04 (m, 3H), 5.21 (d, *J* = 7.8 Hz, 1H), 4.10 (dhept, *J* = 7.9, 6.6 Hz, 1H), 2.47 (d, *J* = 7.2 Hz, 2H), 1.97 (s, 3H), 1.95 – 1.78 (m, 1H), 1.08 – 1.02 (m, 6H), 0.91 (d, *J* = 6.6 Hz, 6H).

^{13}C NMR (75 MHz, $CDCl_3$, 300 K): δ_C (ppm) = 174.4 (C_q), 145.4 (C_q), 142.3 (C_q), 140.3 (C_q), 129.2 (*C*H), 128.4 (*C*H), 128.1 (*C*H), 127.8 (*C*H), 126.8 (*C*H), 56.5 (C_q), 44.9 (CH_2), 41.7 (*C*H), 30.1 (CH_3), 27.0 (*C*H), 22.5 (CH_3), 22.5 (CH_3), 22.4 (CH_3).

HRMS (ESI) *m/z* = 346.2141 calcd. for $[C_{22}H_{29}NONa]^+$ $[M+Na]^+$, found 346.2140.

***N*-Isopropyl-2-methoxy-2,2-diphenylacetamide (2ae):**

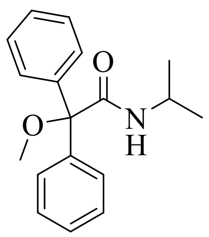

The title compound was prepared following method A of the general procedure **GP6** using *N*-acylated sulfonamide **1ae** (71.0 mg, 0.150 mmol, 1.0 equiv.), *fac*-[Ir(ppy)$_3$] (1.0 mg, 1.5 µmol, 1 mol%) and Cs_2CO_3 (147 mg, 0.450 mmol, 3.0 equiv.) in MeCN (1.5 mL). Purification by reverse-phase MPLC (MeCN/water, gradient from 5% to 90%) afforded the desired amide **2ae** as a colourless oil. (26.5 mg, 94.0 µmol, 63%).

FT IR (neat) υ (cm^{-1}) = 3416, 2970, 2931, 1673, 1511, 1449, 1367, 1175, 1092, 1075, 908, 773, 728, 700, 647, 602, 578.

^{1}H NMR (600 MHz, $CDCl_3$, 300 K): δ_H (ppm) = 7.47 – 7.43 (m, 4H), 7.39 – 7.31 (m, 6H), 7.02 (d, *J* = 8.4 Hz, 1H), 4.14 – 4.03 (m, 1H), 3.03 (s, 3H), 1.18 (d, *J* = 6.6 Hz, 7H).

^{13}C NMR (151 MHz, $CDCl_3$, 300 K): δ_C (ppm) = 171.2 (C_q), 139.1 (C_q), 129.0 (*C*H), 127.9 (*C*H), 127.8 (*C*H), 115.0 (C_q), 52.6 (CH_3), 41.2 (*C*H), 22.7 (2 x CH_3).

HRMS (ESI) *m/z* = 306.1465 calcd. for $[C_{18}H_{21}NO_2Na]^+$ $[M+Na]^+$, found 306.1463.

***N*-Isopropyl-2-phenoxy-2-phenylacetamide (2af):**

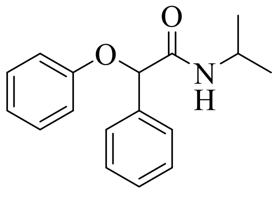

The title compound was prepared following method A of the general procedure **GP6** using *N*-acylated sulfonamide **1af** (58.2 mg, 0.131 mmol, 1.0 equiv.), *fac*-[Ir(ppy)$_3$] (0.9 mg, 1.3 µmol, 1 mol%) and Cs_2CO_3 (128 mg, 0.393 mmol, 3.0 equiv.) in MeCN (1.3 mL). Purification by reverse-phase MPLC (MeCN/water, gradient from 5% to 90%) afforded the desired amide **2af** as a colourless solid. (16.8 mg, 62.4 µmol, 48%).

MP: 103-104 °C.

FT IR (neat) υ (cm^{-1}) = 3295, 2973, 1657, 1598, 1529, 1494, 1454, 1229, 1172, 1052, 753, 729, 692.

^{1}H NMR (300 MHz, $CDCl_3$, 300 K): δ_H (ppm) = 7.55 – 7.50 (m, 2H), 7.41 – 7.26 (m, 5H), 7.03 – 6.93 (m, 3H), 6.50 (d, J = 8.2 Hz, 1H), 5.51 (s, 1H), 4.17 – 4.05 (m, 1H), 1.16 (d, J = 6.5 Hz, 6H).

^{13}C NMR (75 MHz, $CDCl_3$, 300 K): δ_C (ppm) = 168.7 (C_q), 156.9 (C_q), 136.6 (C_q), 129.7 (CH), 128.6 (CH), 128.5 (CH), 126.6 (CH), 122.2 (CH), 115.9 (CH), 80.4 (CH), 41.3 (CH), 22.6 (2 x CH_3).

HRMS (ESI) *m/z* = 292.1308 calcd. for $[C_{17}H_{19}NO_2Na]^+$ $[M+Na]^+$, found 292.1305.

***N*-Isopropyl-2-phenylpropanamide (2ag):**

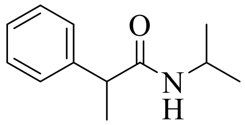

The title compound was prepared following method A of the general procedure **GP6** using *N*-acylated sulfonamide **1ag** (57.2 mg, 0.150 mmol, 1.0 equiv.), *fac*-[Ir(ppy)$_3$] (1.0 mg, 1.5 µmol, 1 mol%) and Cs_2CO_3 (147 mg, 0.450 mmol, 3.0 equiv.) in MeCN (1.5 mL). Purification by reverse-phase-MPLC (MeCN/water, gradient from 5% to 90%) afforded the desired amide **2ag** as a colourless solid (26.8 mg, 0.140 mmol, 93%).

^{1}H NMR (300 MHz, $CDCl_3$, 300 K): δ_H (ppm) = 7.38 – 7.24 (m, 5H), 5.15 (s, 1H), 4.12 – 3.96 (m, 1H), 3.51 (q, J = 7.2 Hz, 1H), 1.51 (d, J = 7.2 Hz, 3H), 1.08 (d, J = 6.5 Hz, 3H), 1.03 (d, J = 6.5 Hz, 3H).

^{13}C NMR (75 MHz, $CDCl_3$, 300 K): δ_C (ppm) = 173.3 (C_q), 141.6 (C_q), 128.9 (CH), 127.6 (CH), 127.2 (CH), 47.2 (CH), 41.4 (CH), 22.6 (CH_3), 22.5 (CH_3), 18.7 (CH_3).

HRMS (ESI) *m/z* = 214.1202 calcd. for $[C_{12}H_{17}NONa]^+$ $[M+Na]^+$, found 214.1200.

The analytical data are consistent with those reported in literature.[23]

***N*-Isopropyl-2-phenylacetamide (2ah):**

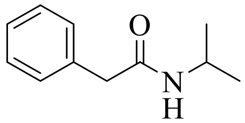

The title compound was prepared following method A of the general procedure **GP6** using *N*-acylated sulfonamide **1ah** (55.1 mg, 0.150 mmol, 1.0 equiv.), *fac*-[Ir(ppy)$_3$] (1.0 mg, 1.5 µmol, 1 mol%) and Cs_2CO_3 (147 mg, 0.450 mmol, 3.0 equiv.) in MeCN (1.5 mL). Purification by reverse-phase-MPLC (MeCN/water, gradient from 5% to 90%) afforded the desired amide **2ah** as a colourless solid (25.3 mg, 0.143 mmol, 95%).

^{1}H NMR (300 MHz, $CDCl_3$, 300 K): δ_H (ppm) = 7.42 – 7.26 (m, 5H), 5.25 (s, 1H), 4.17 – 4.02 (m, 1H), 3.57 (s, 2H), 1.10 (d, J = 6.6 Hz, 6H).

^{13}C NMR (75 MHz, $CDCl_3$, 300 K): δ_C (ppm) = 170.1 (C_q), 135.1 (C_q), 129.4 (*C*H), 129.0 (*C*H), 127.3 (*C*H), 44.0 (CH_2), 41.5 (*C*H), 22.6 (CH_3).

HRMS (ESI) *m/z* = 200.1046 calcd. for $[C_{11}H_{15}NONa]^+$ $[M+Na]^+$, found 200.1044.

The analytical data are consistent with those reported in literature.[22]

7. NMR data of compounds

^{1}H NMR (300 MHz, DMSO-d^{6}):

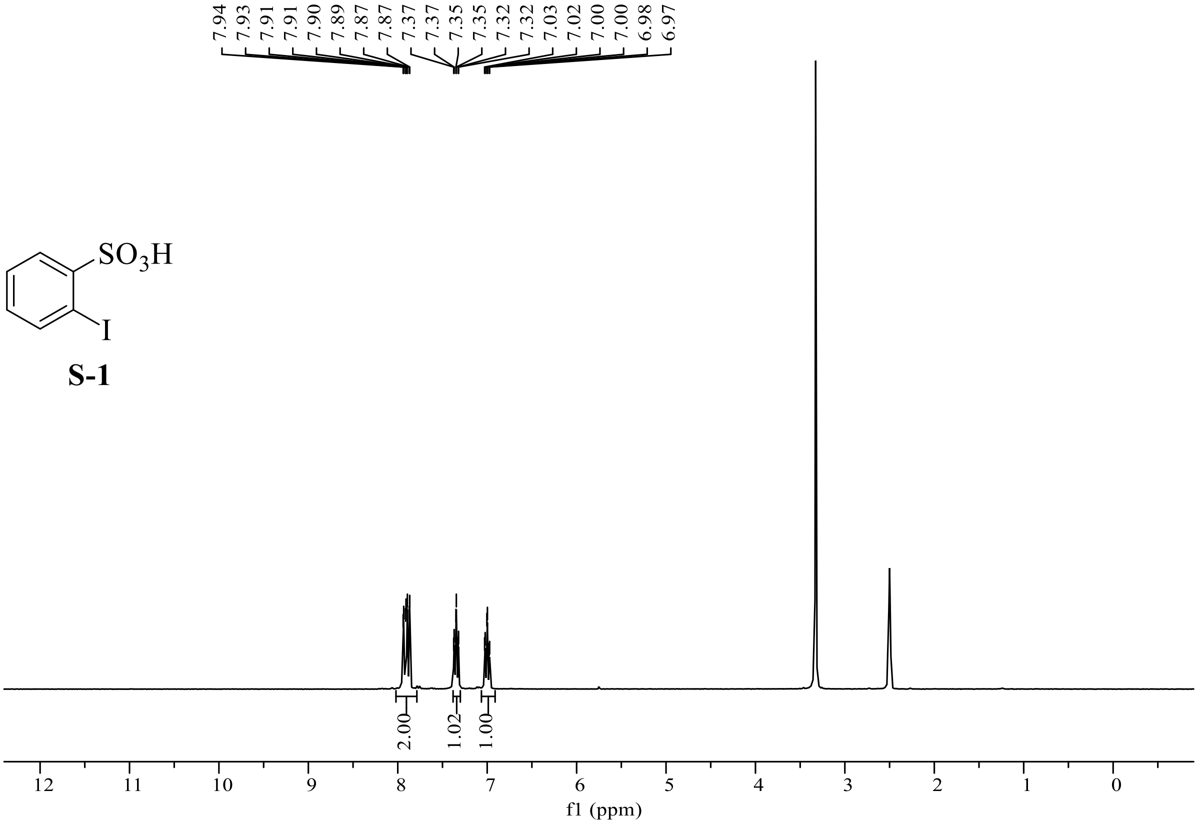

^{13}C NMR (75 MHz, DMSO-d^{6}):

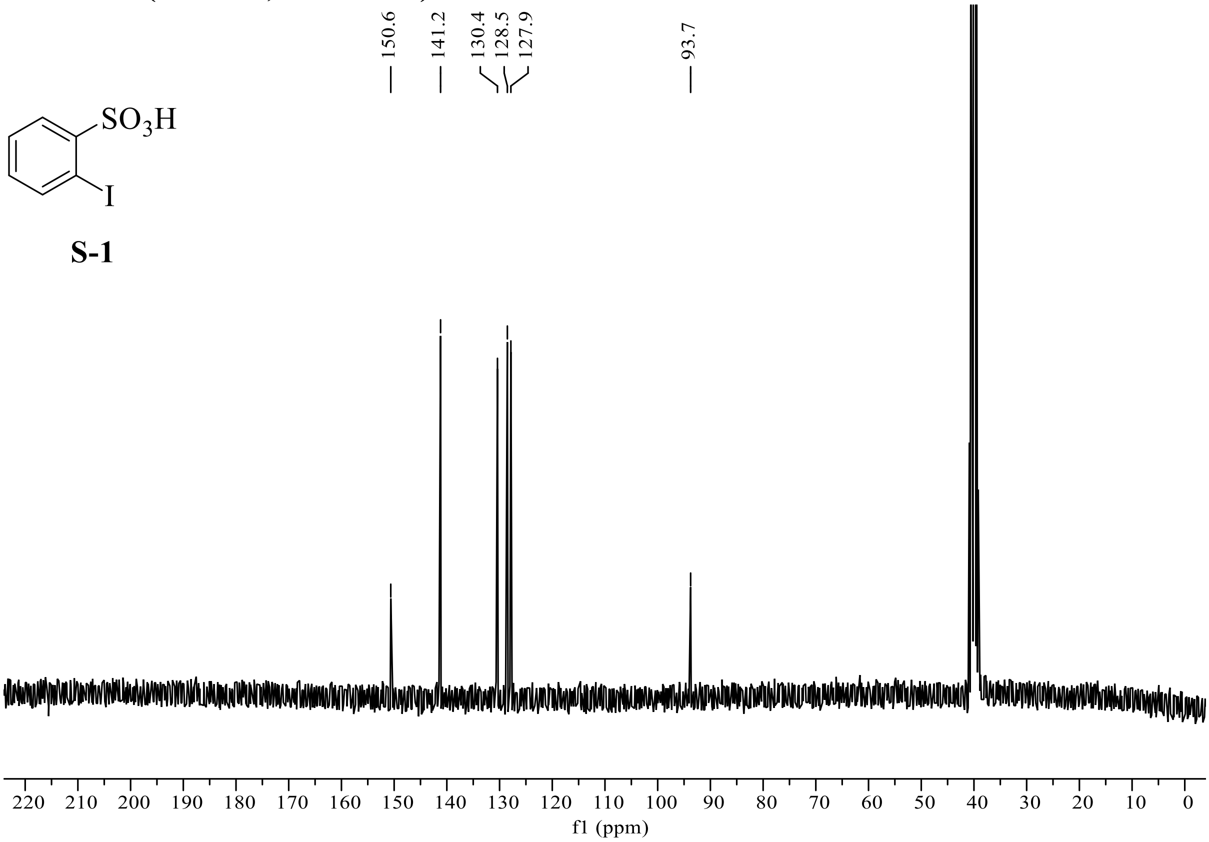

^{1}H NMR (300 MHz, D_2O):

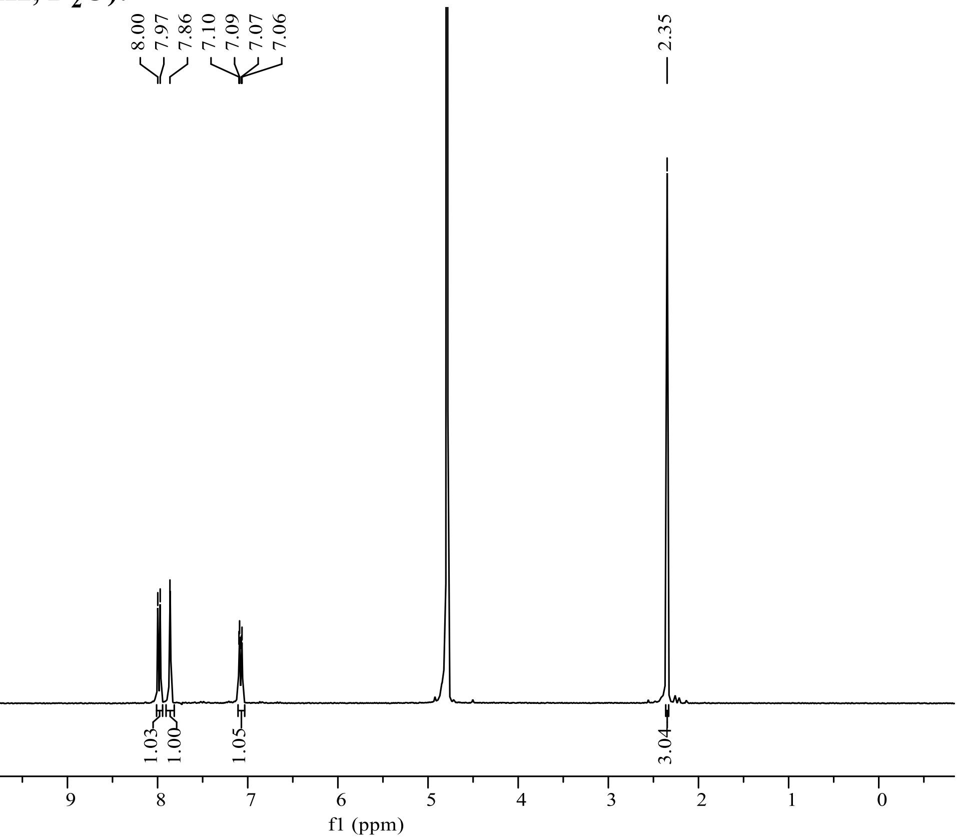

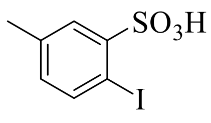

S-2

^{13}C NMR (75 MHz, D_2O):

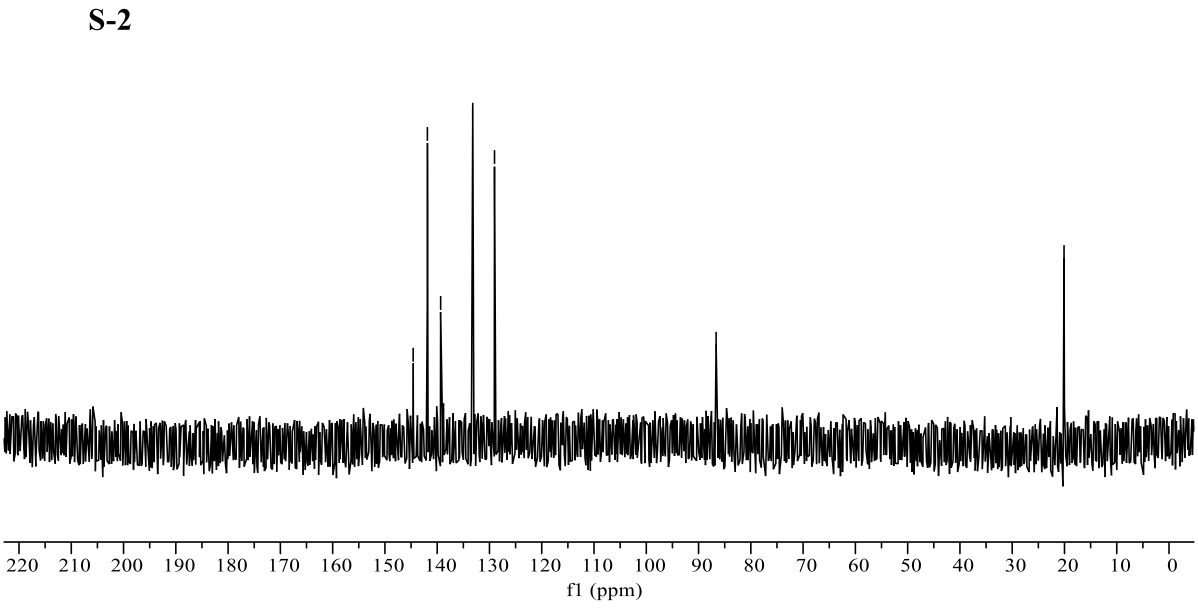

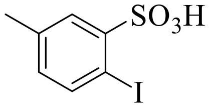

S-2

^{1}H NMR (300 MHz, D_2O):

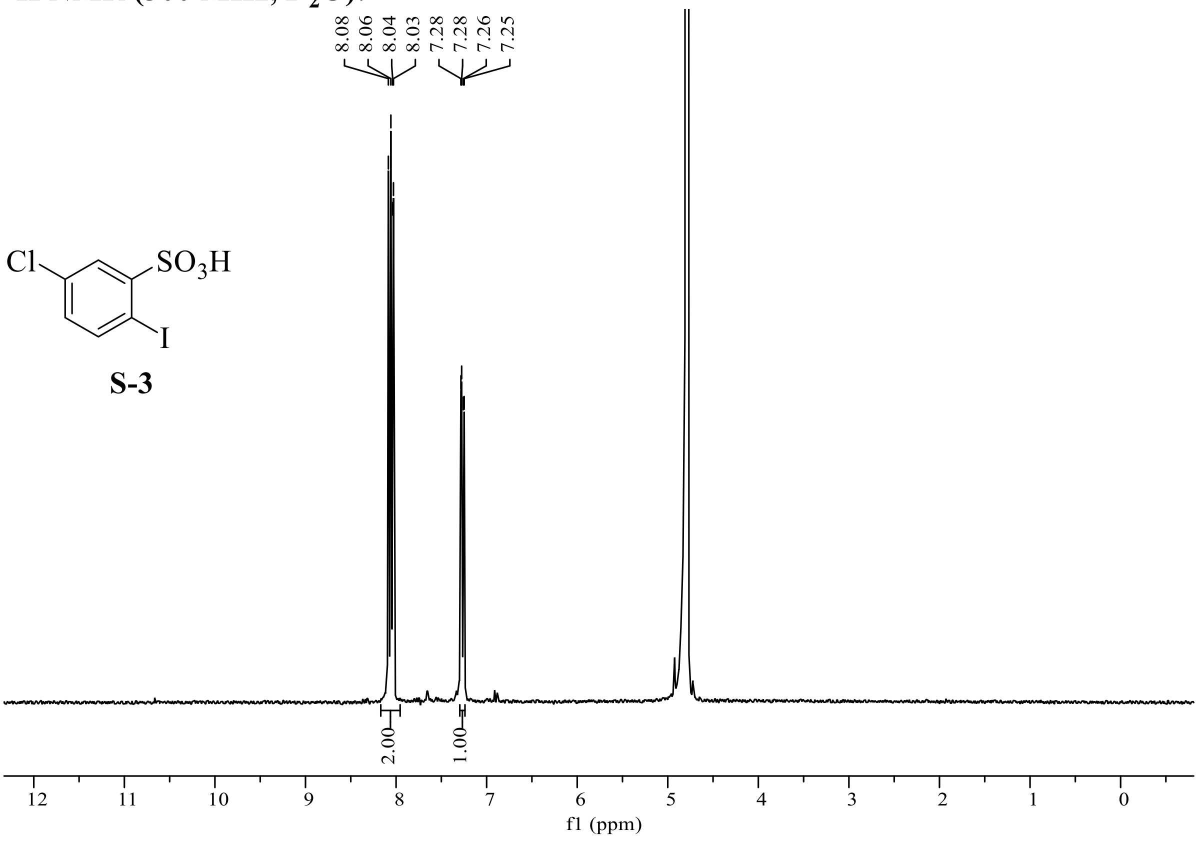

^{13}C NMR (75 MHz, D_2O):

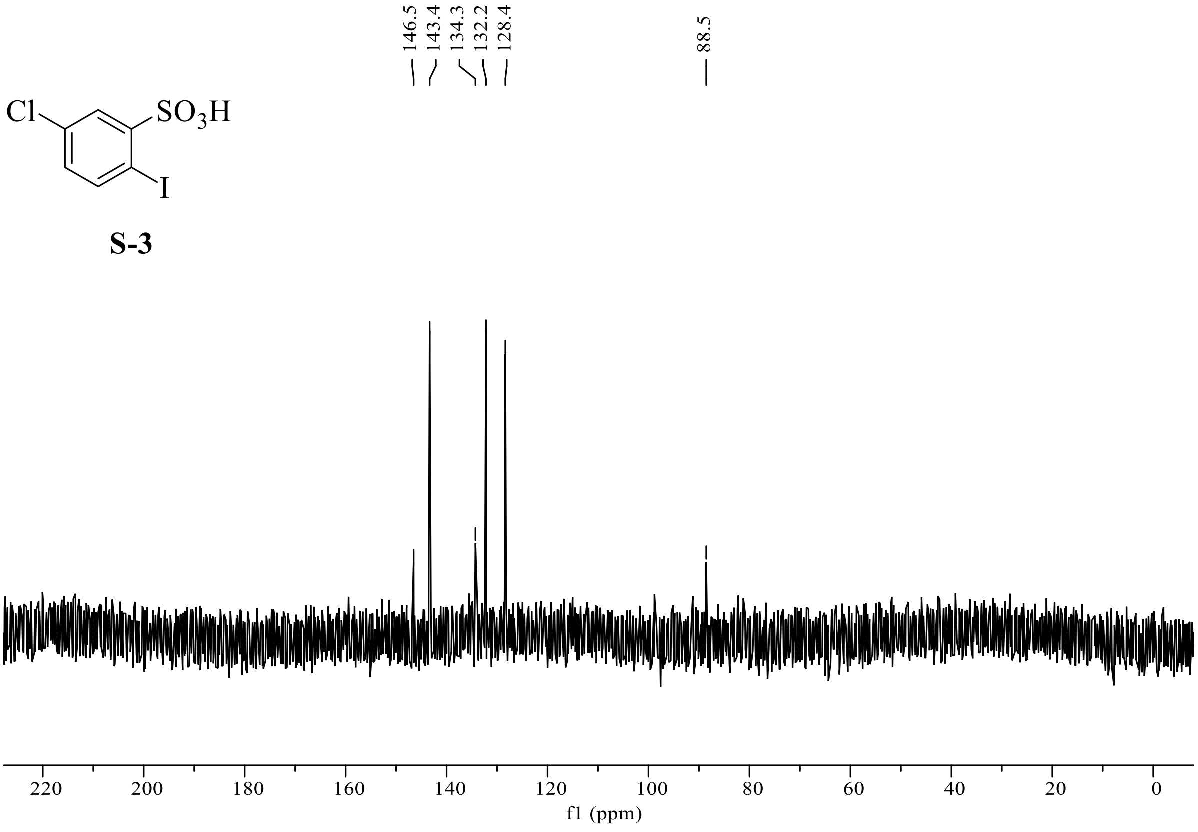

^{1}H NMR (300 MHz, D_2O):

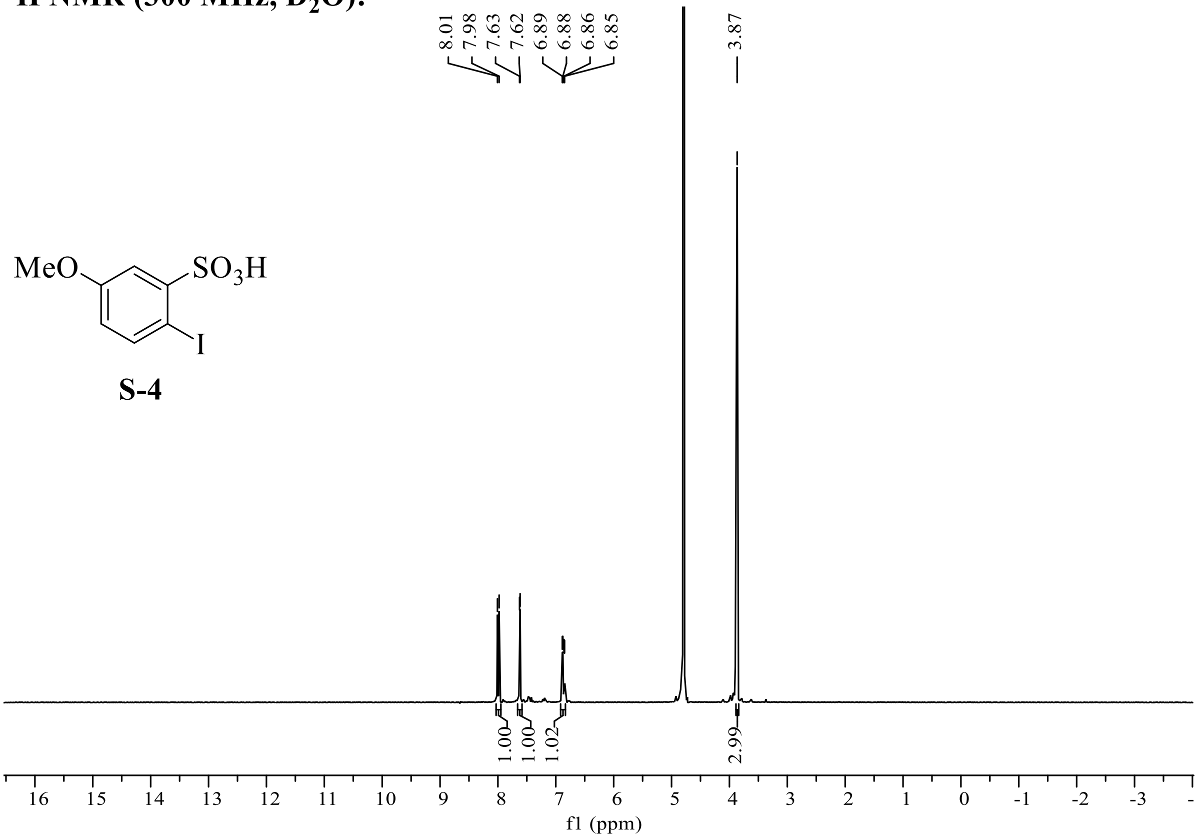

^{13}C NMR (75 MHz, D_2O):

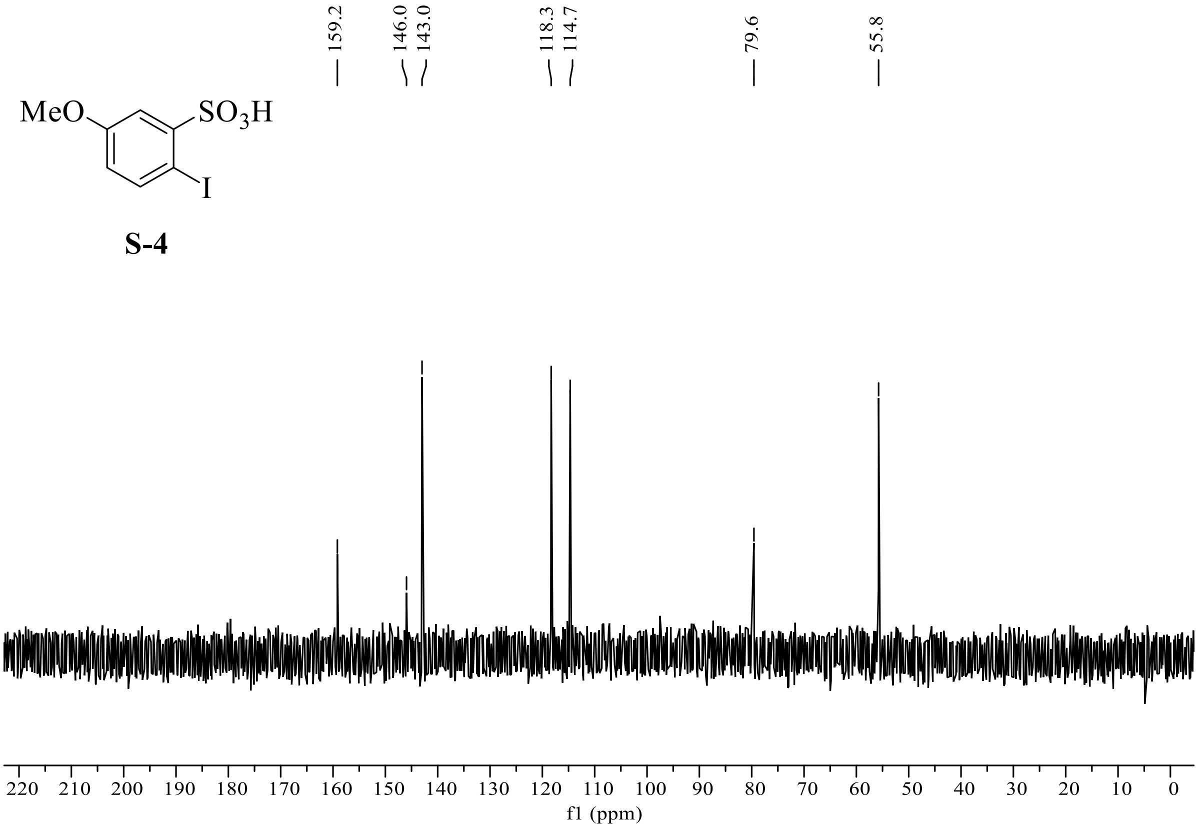

^{1}H NMR (300 MHz, D_2O):

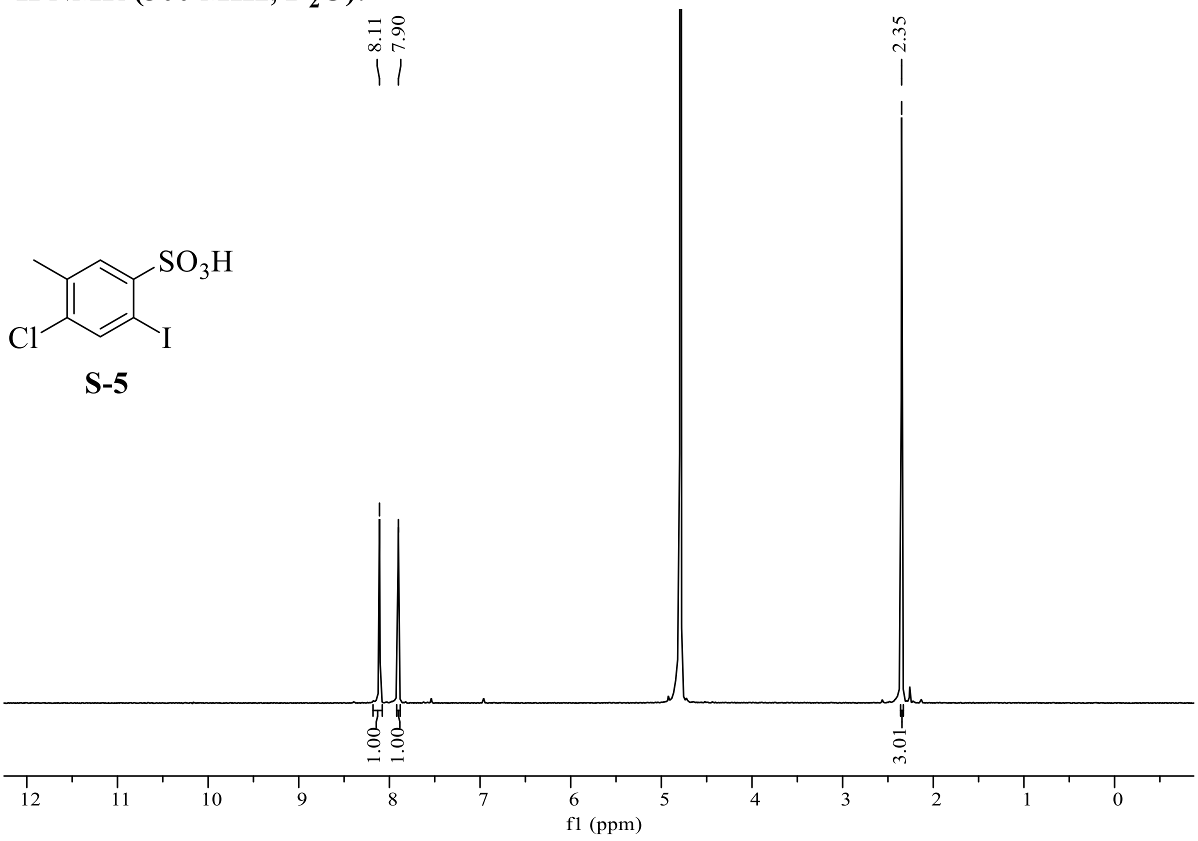

^{13}C NMR (75 MHz, D_2O):

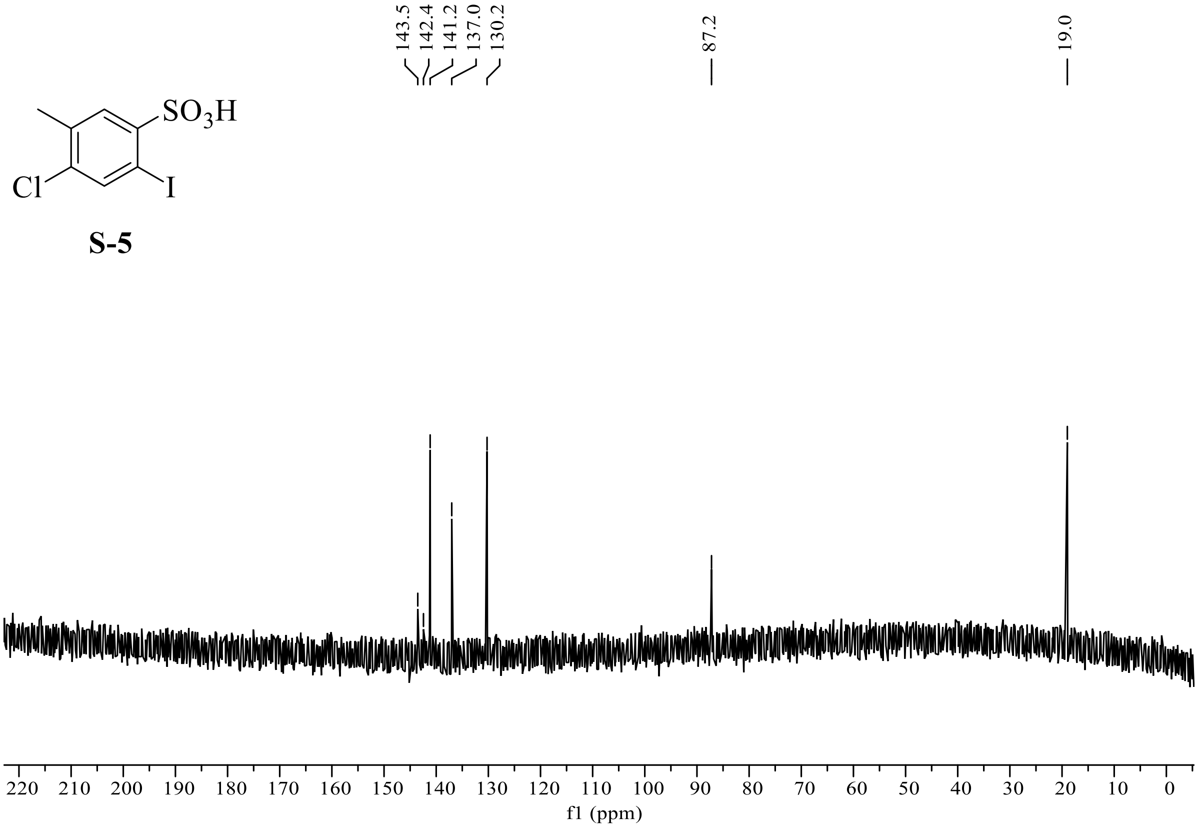

^{1}H NMR (300 MHz, D_2O):

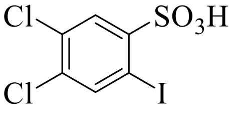

S-6

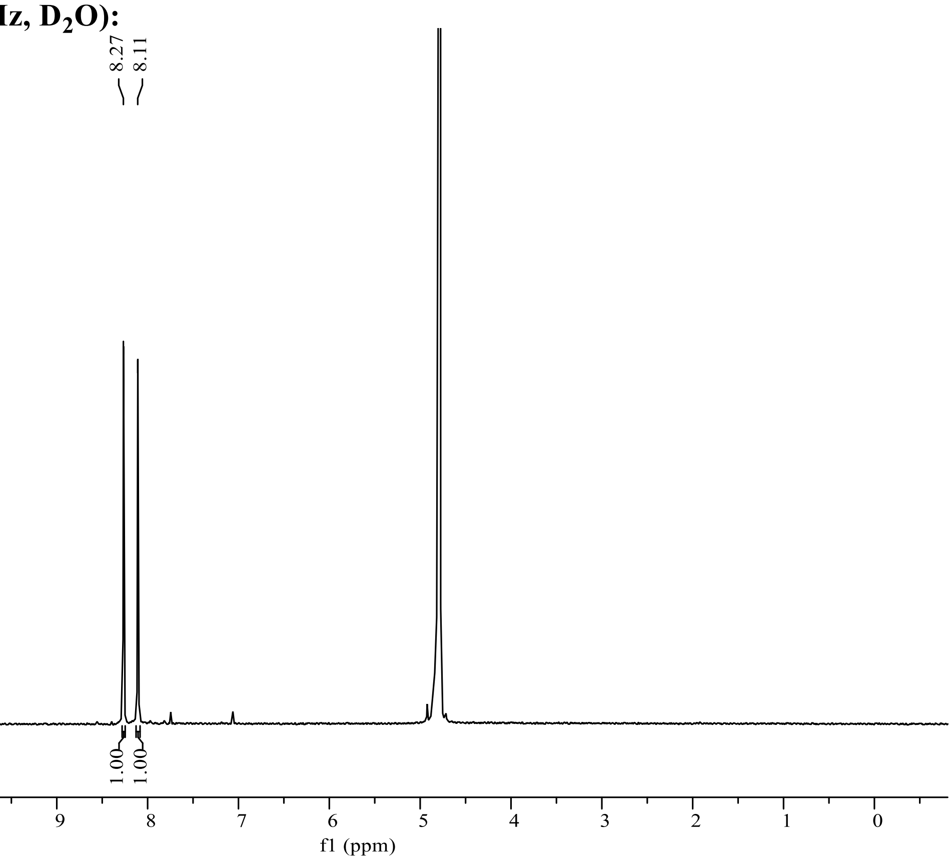

^{13}C NMR (75 MHz, D_2O):

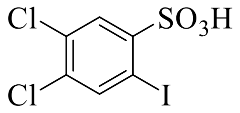

S-6

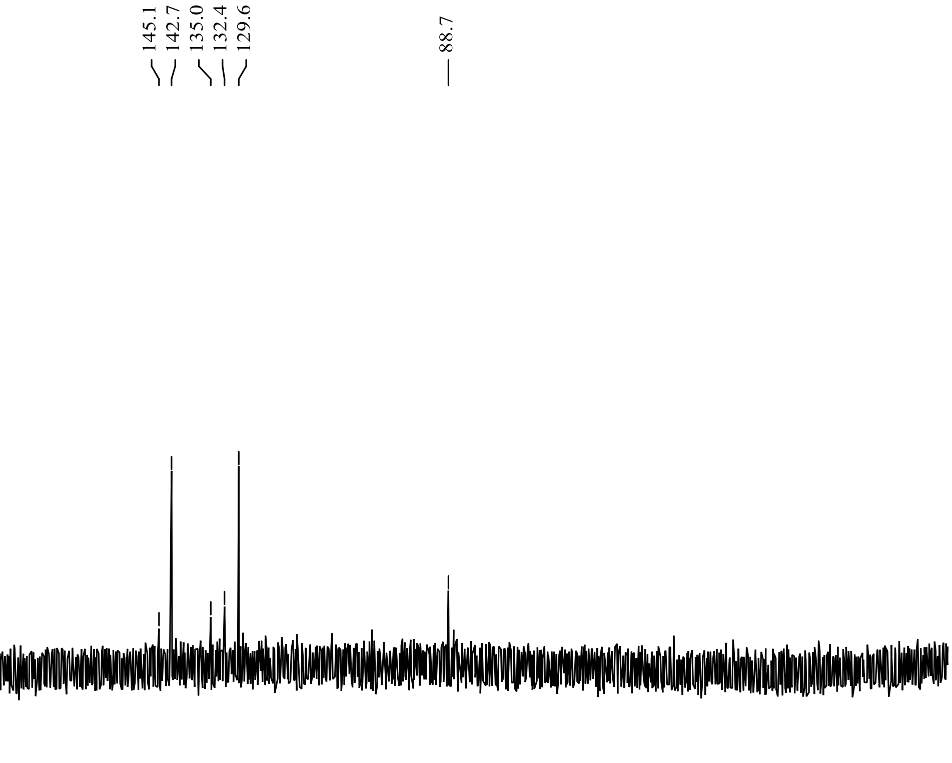

^{1}H NMR (300 MHz, DMSO-d^6):

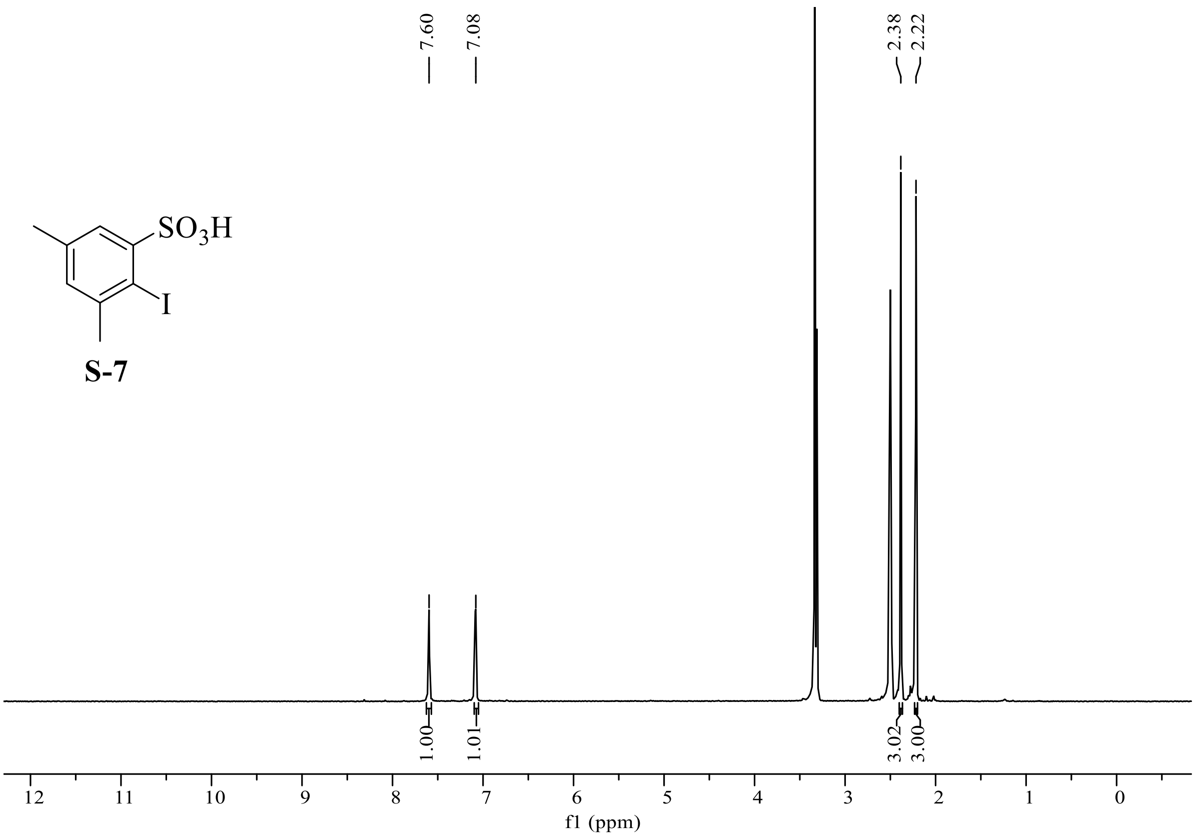

S-7

^{13}C NMR (75 MHz, DMSO-d^6):

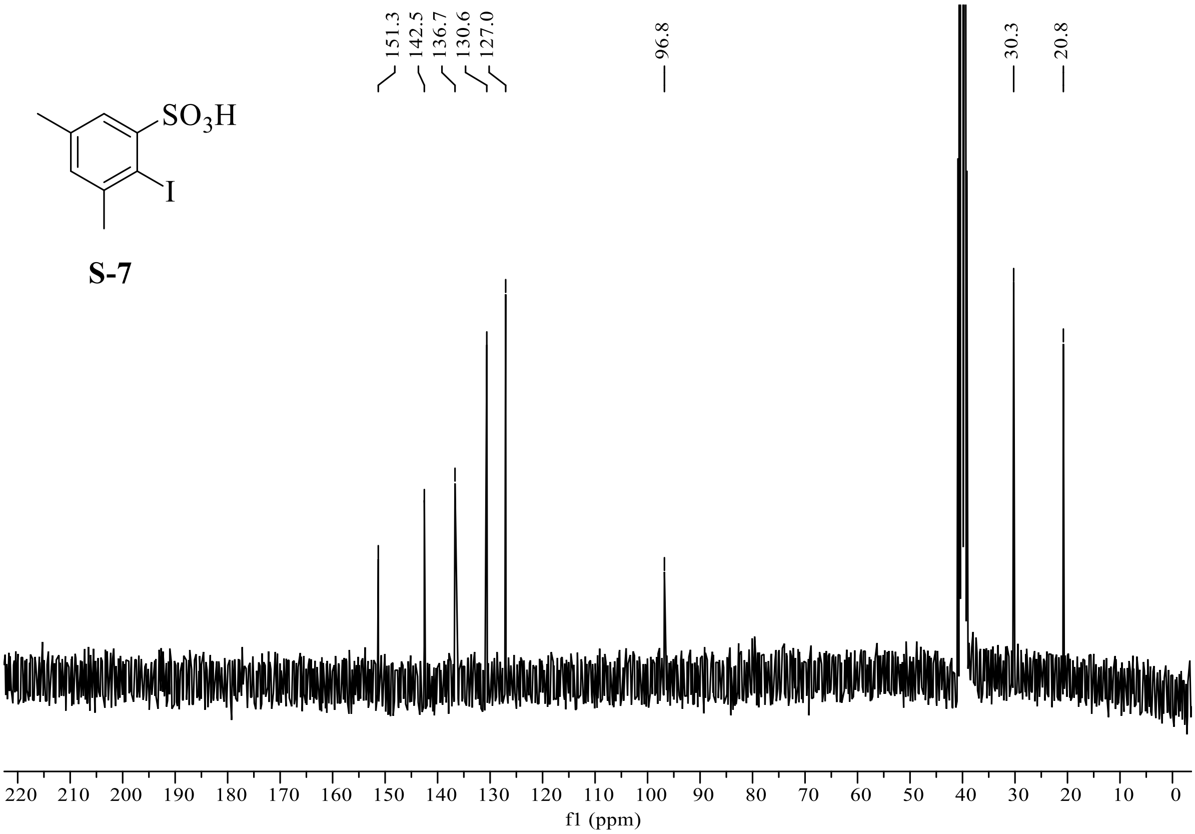

S-7

^{1}H NMR (300 MHz, $CDCl_3$):

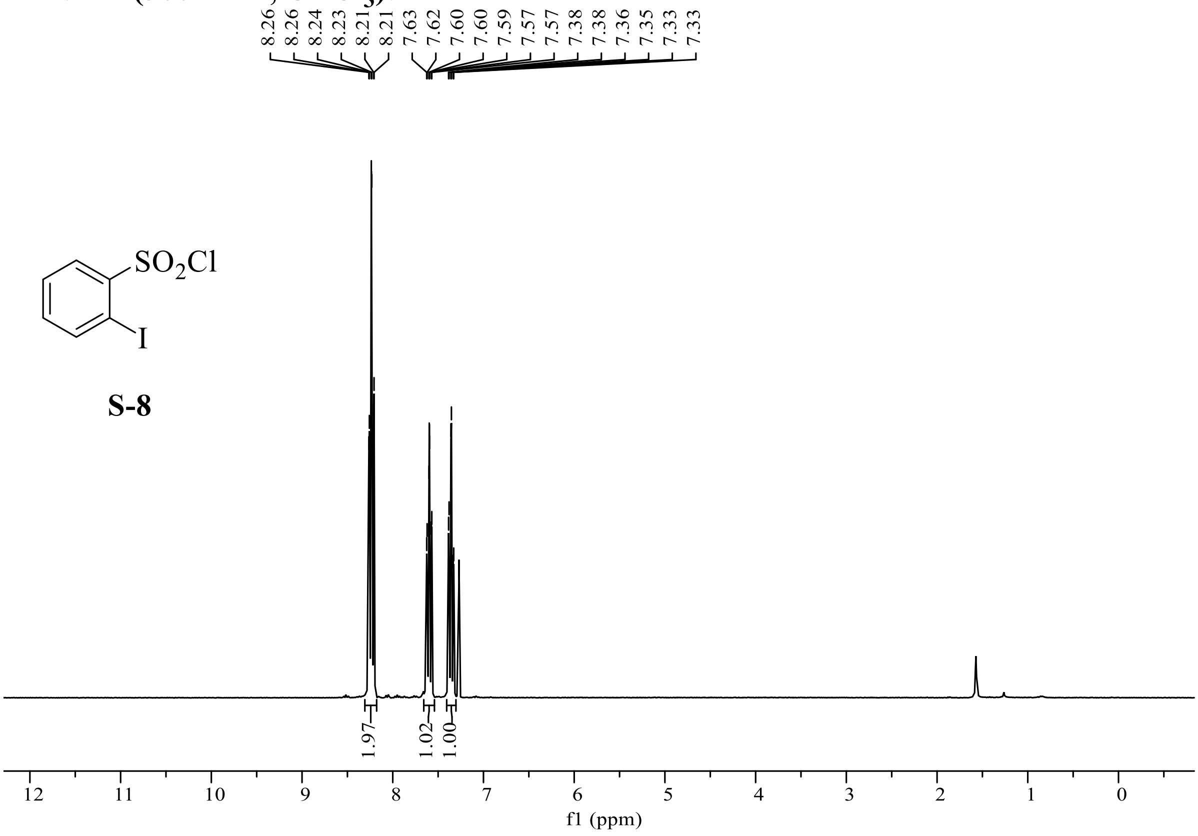

^{13}C NMR (75 MHz, $CDCl_3$):

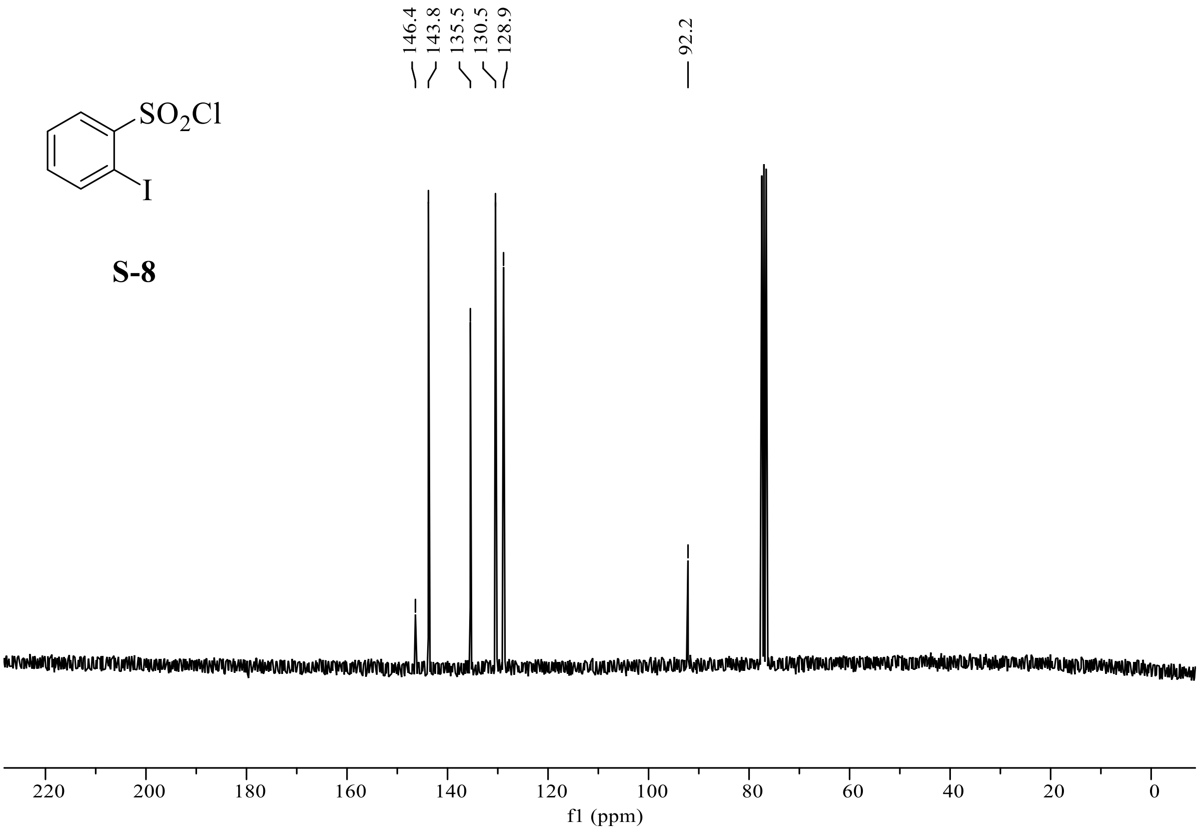

^{1}H NMR (300 MHz, $CDCl_3$):

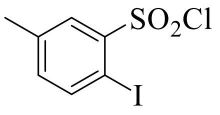

S-9

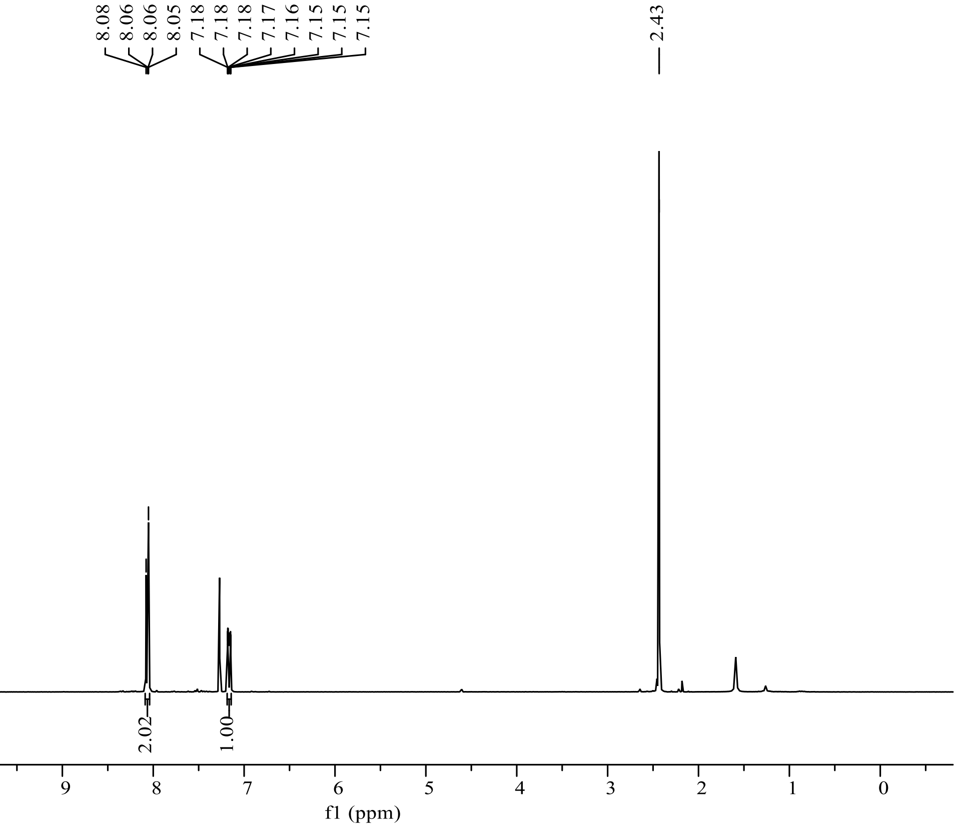

^{13}C NMR (75 MHz, $CDCl_3$):

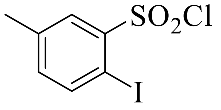

S-9

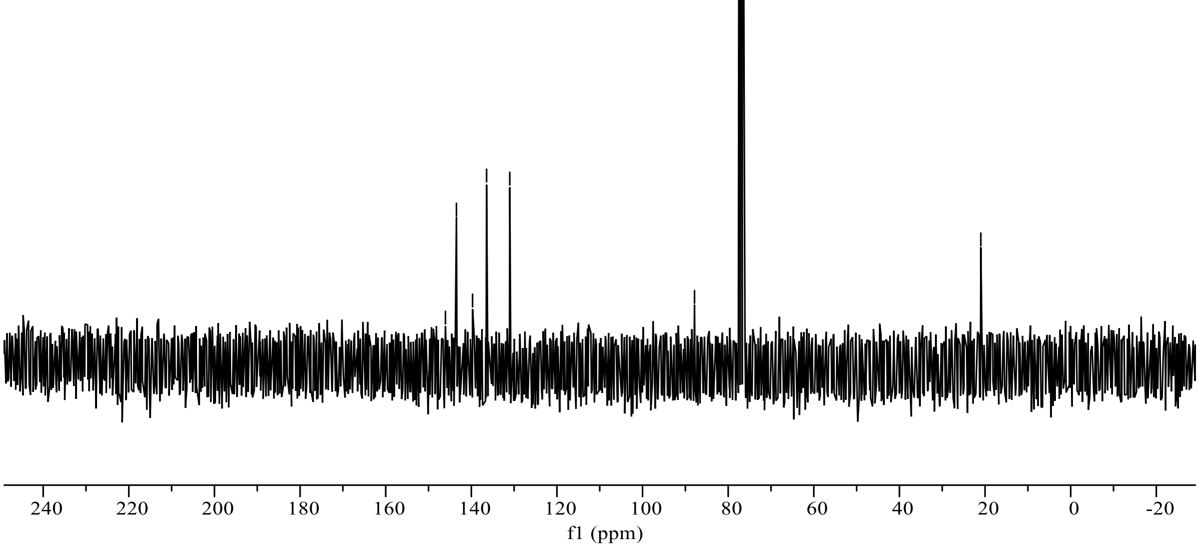

^{1}H NMR (300 MHz, $CDCl_3$):

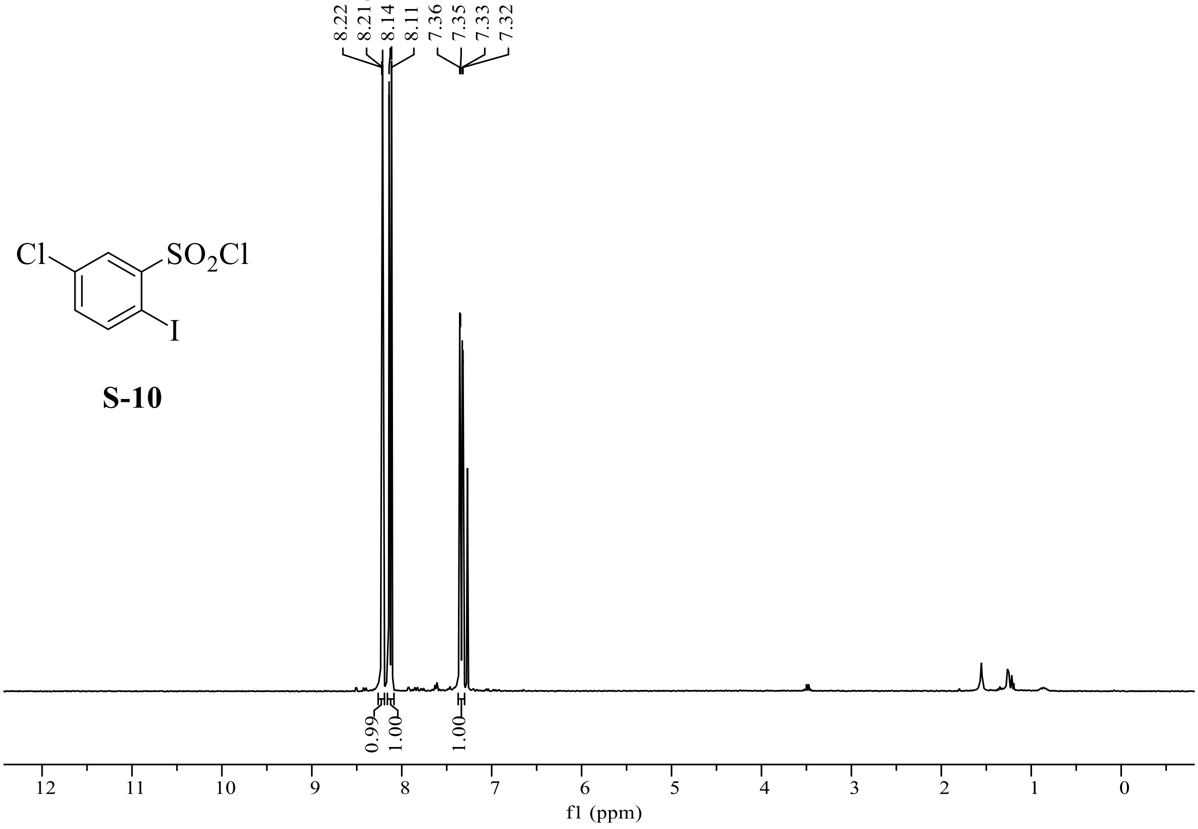

S-10

^{13}C NMR (75 MHz, $CDCl_3$):

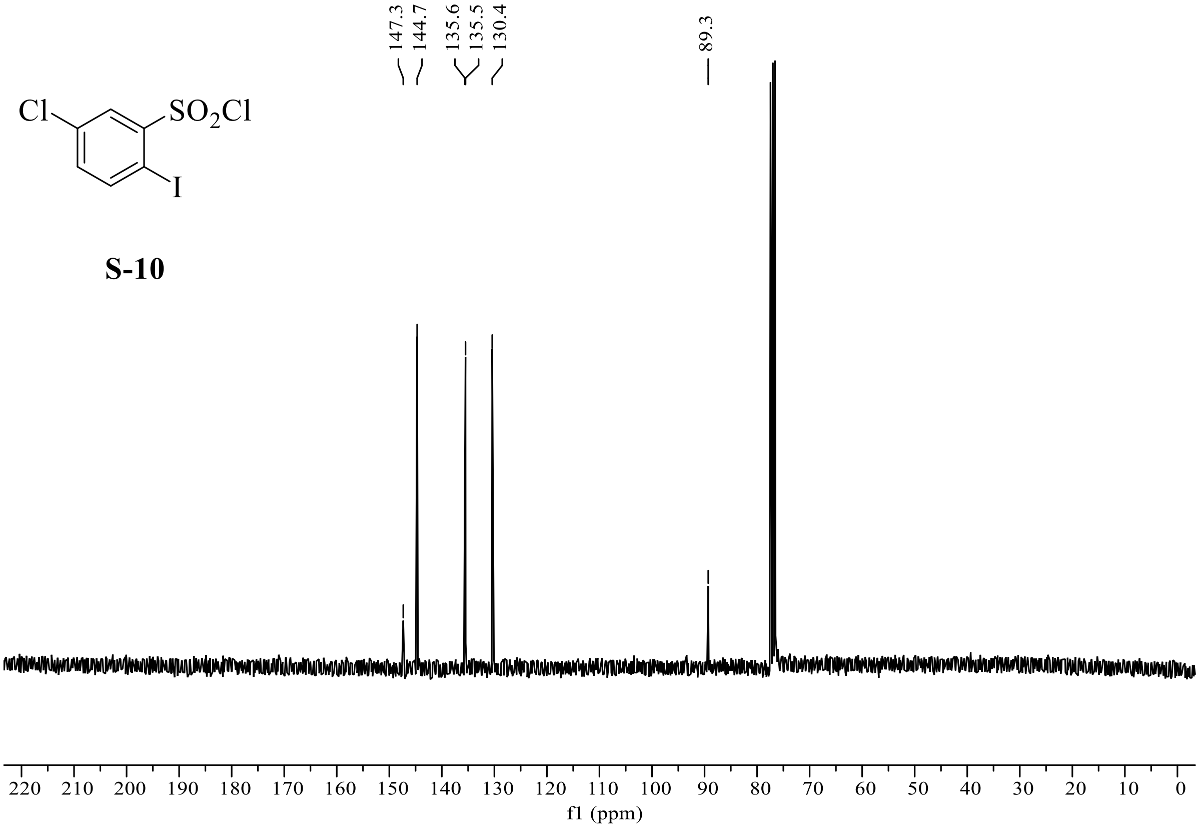

S-10

^{1}H NMR (300 MHz, $CDCl_3$):

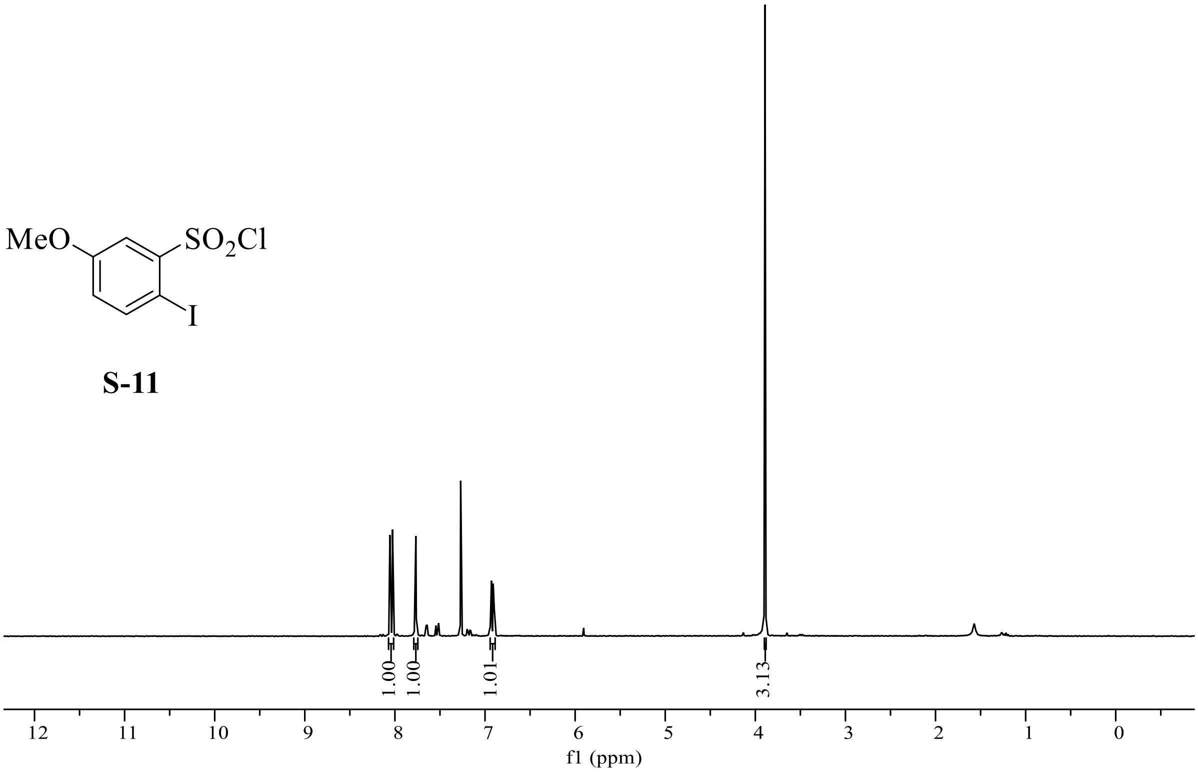

^{13}C NMR (75 MHz, $CDCl_3$):

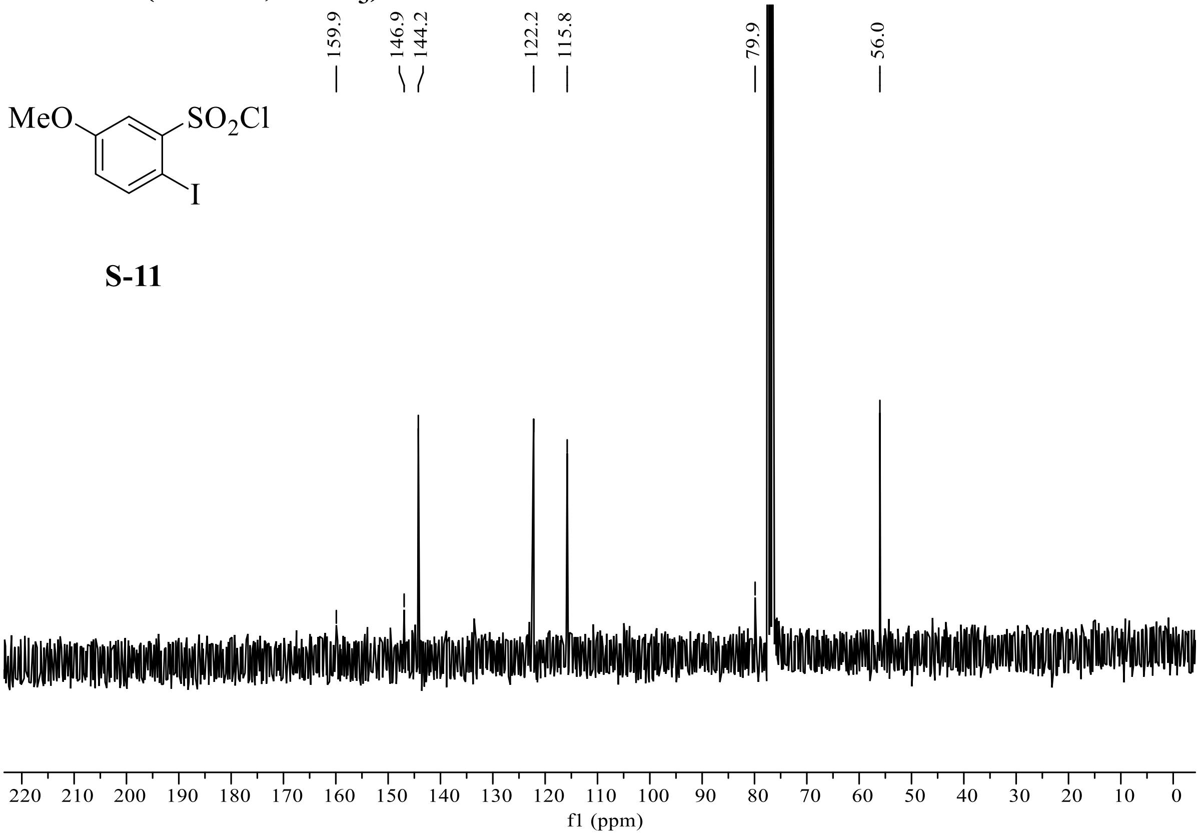

^{1}H NMR (300 MHz, $CDCl_3$):

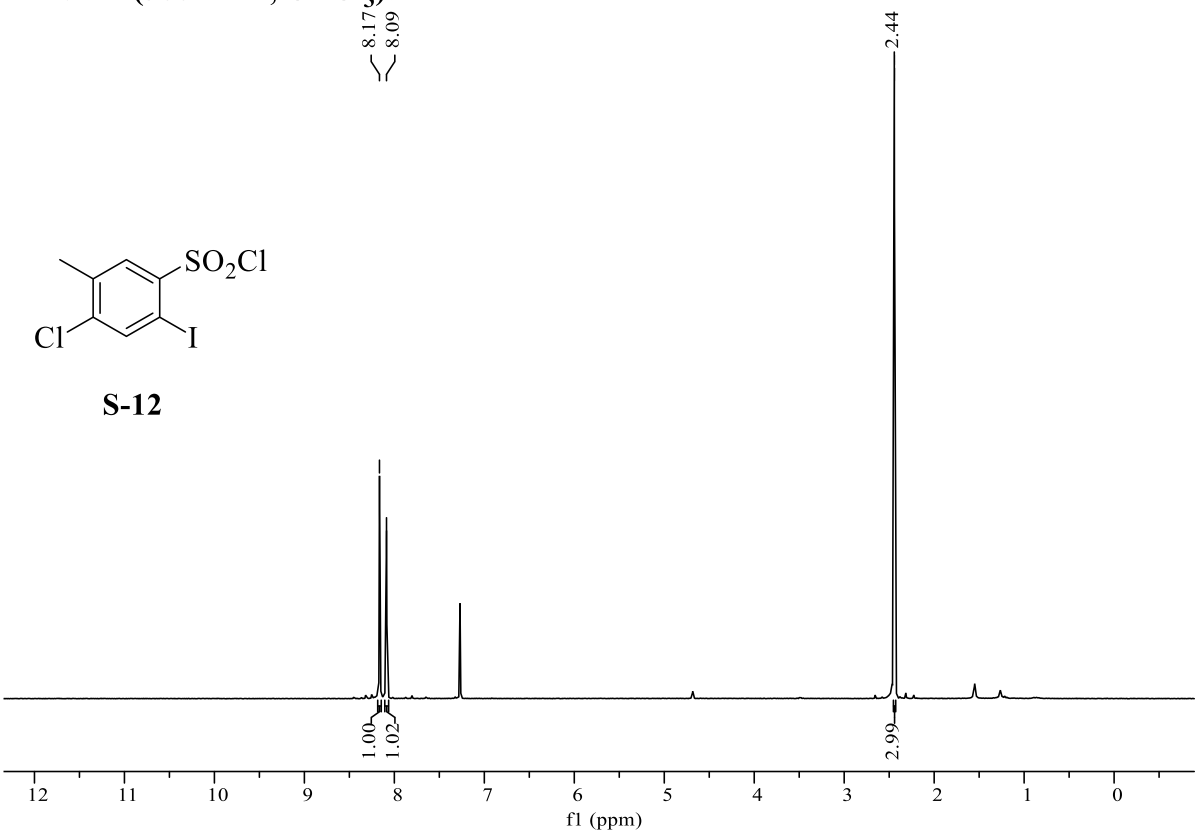

^{13}C NMR (75 MHz, $CDCl_3$):

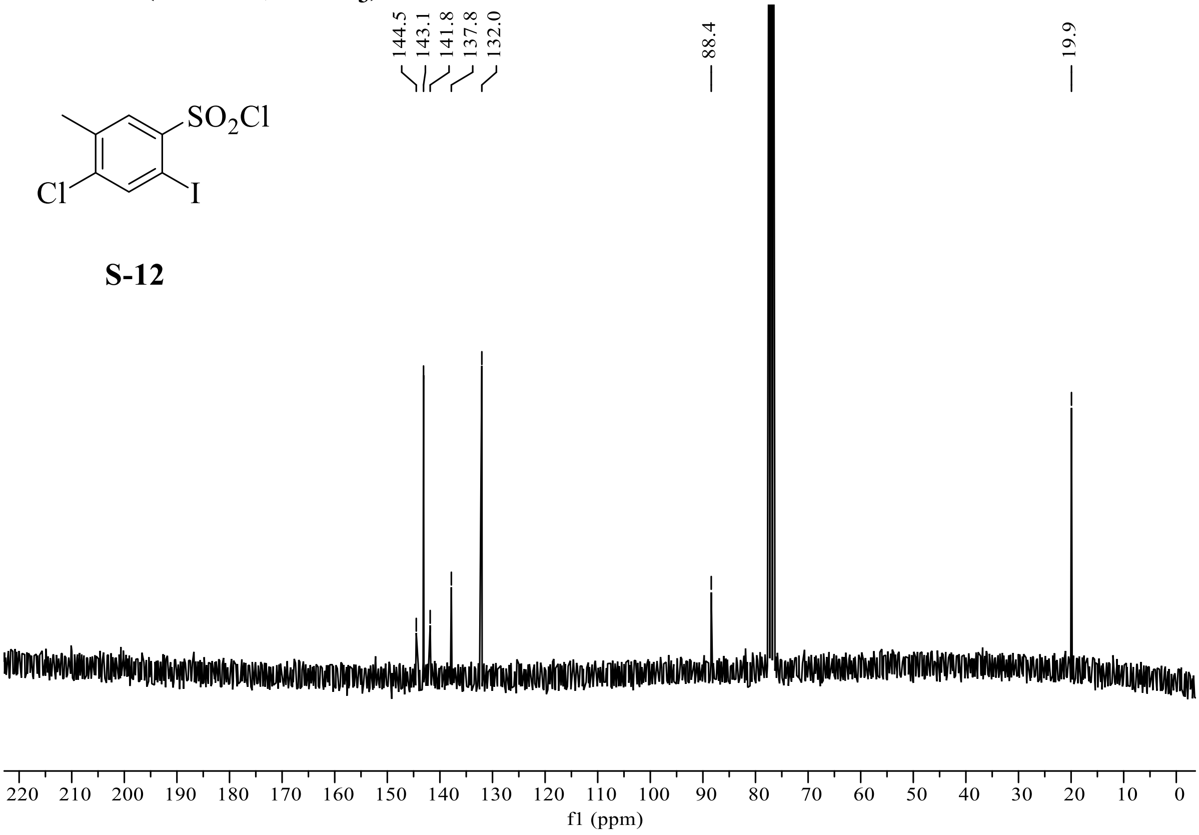

^{1}H NMR (300 MHz, $CDCl_3$):

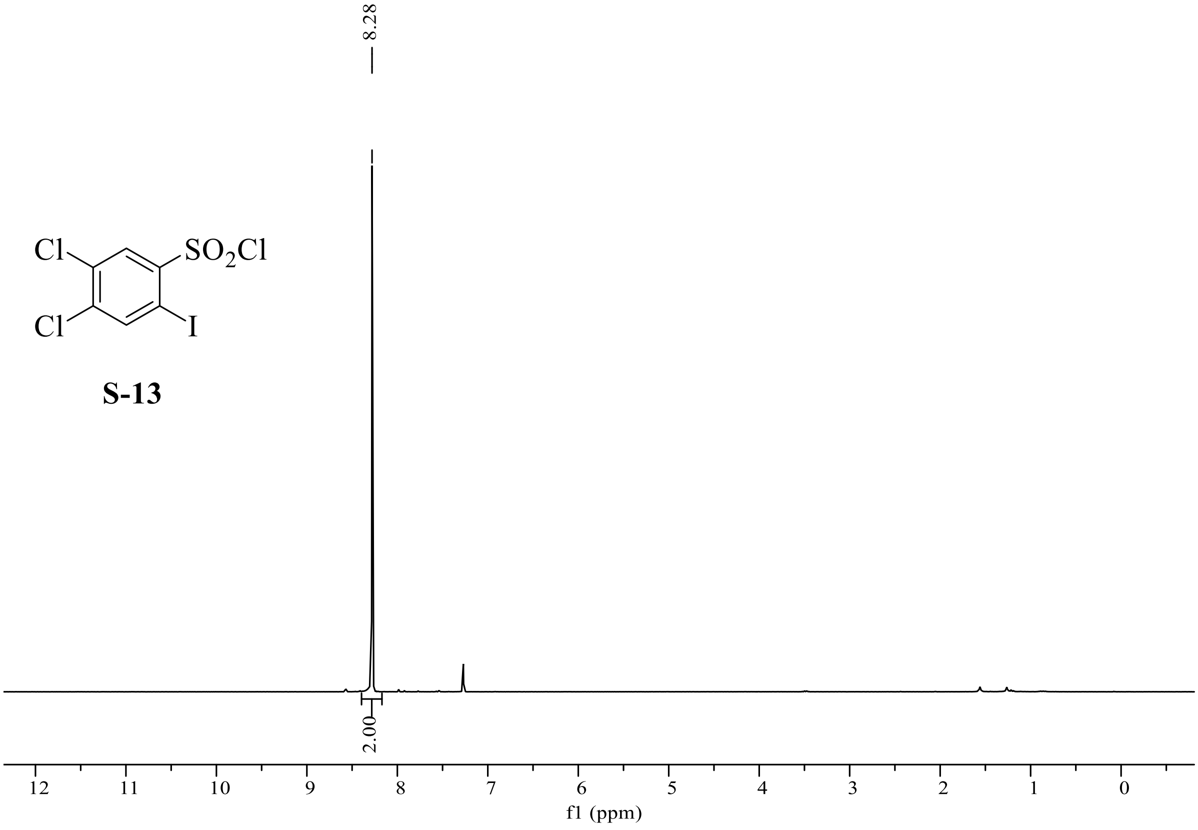

^{13}C NMR (75 MHz, $CDCl_3$):

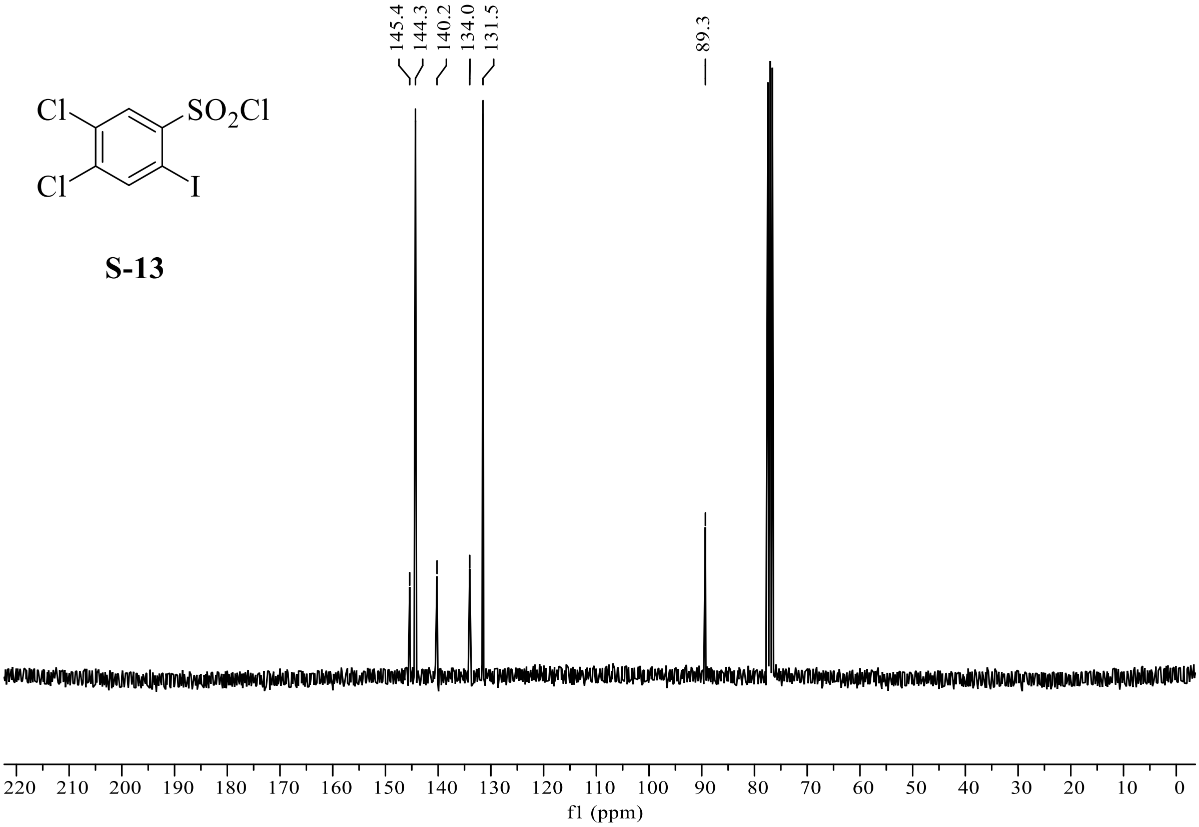

^{1}H NMR (300 MHz, $CDCl_3$):

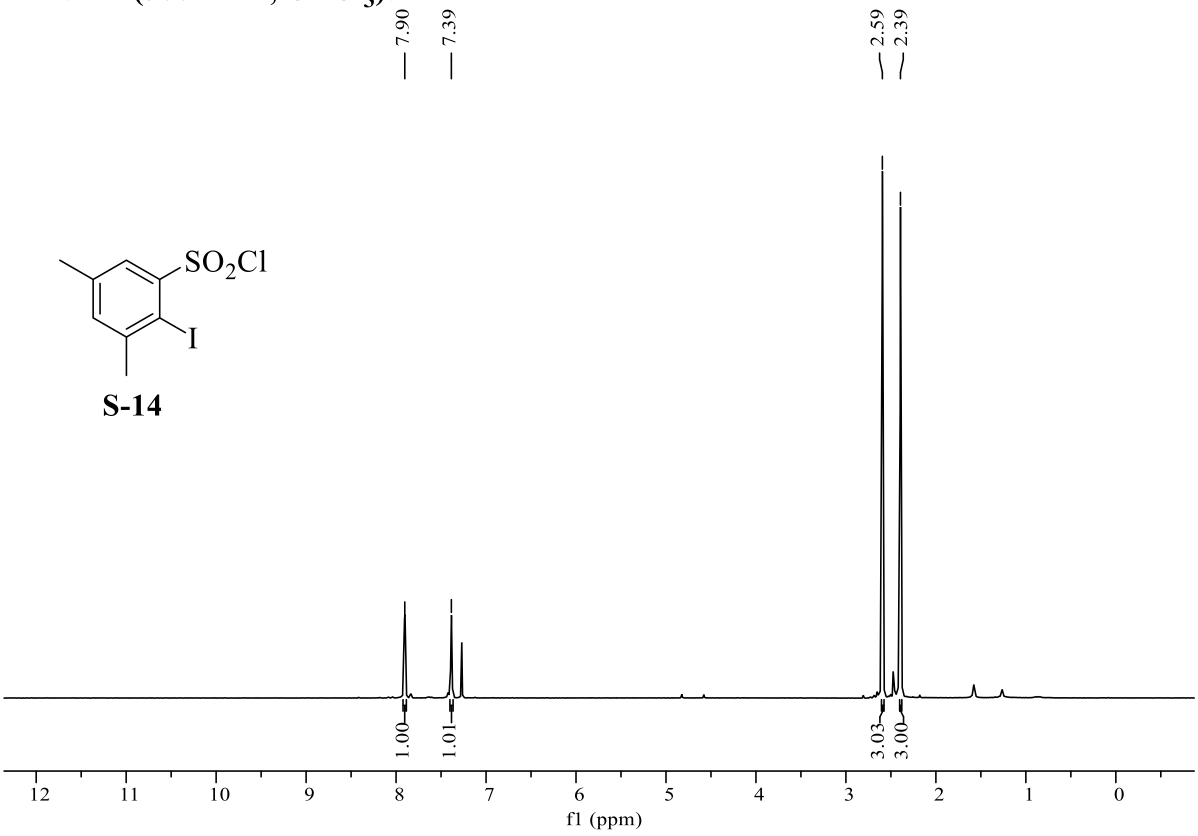

S-14

^{13}C NMR (75 MHz, $CDCl_3$):

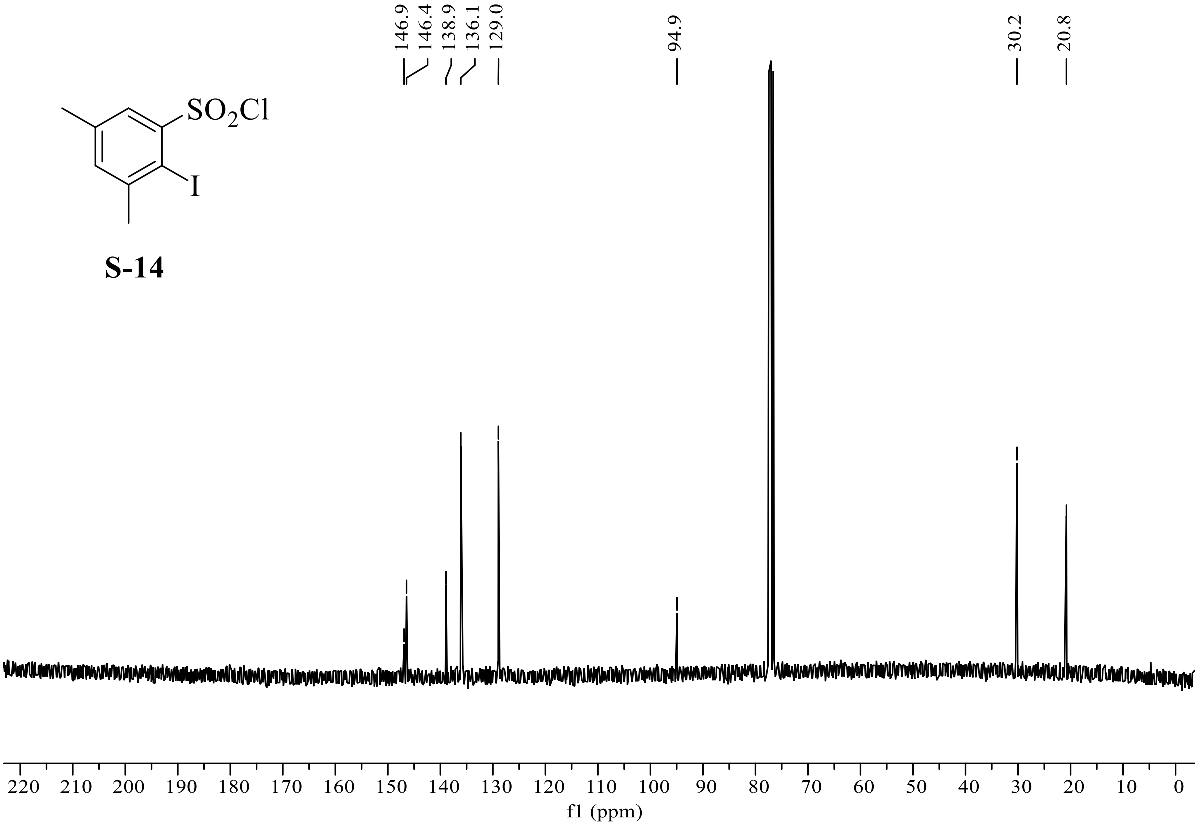

S-14

^{1}H NMR (300 MHz, $CDCl_3$):

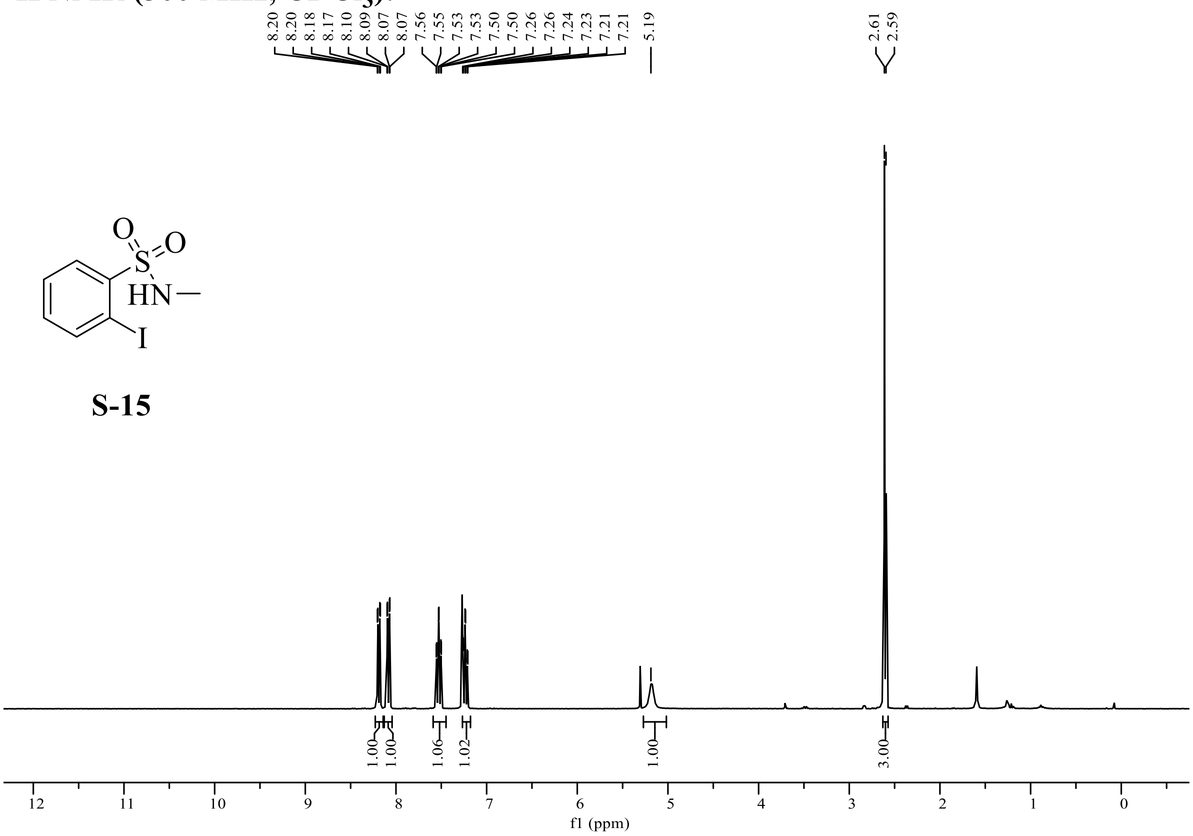

S-15

^{13}C NMR (75 MHz, $CDCl_3$):

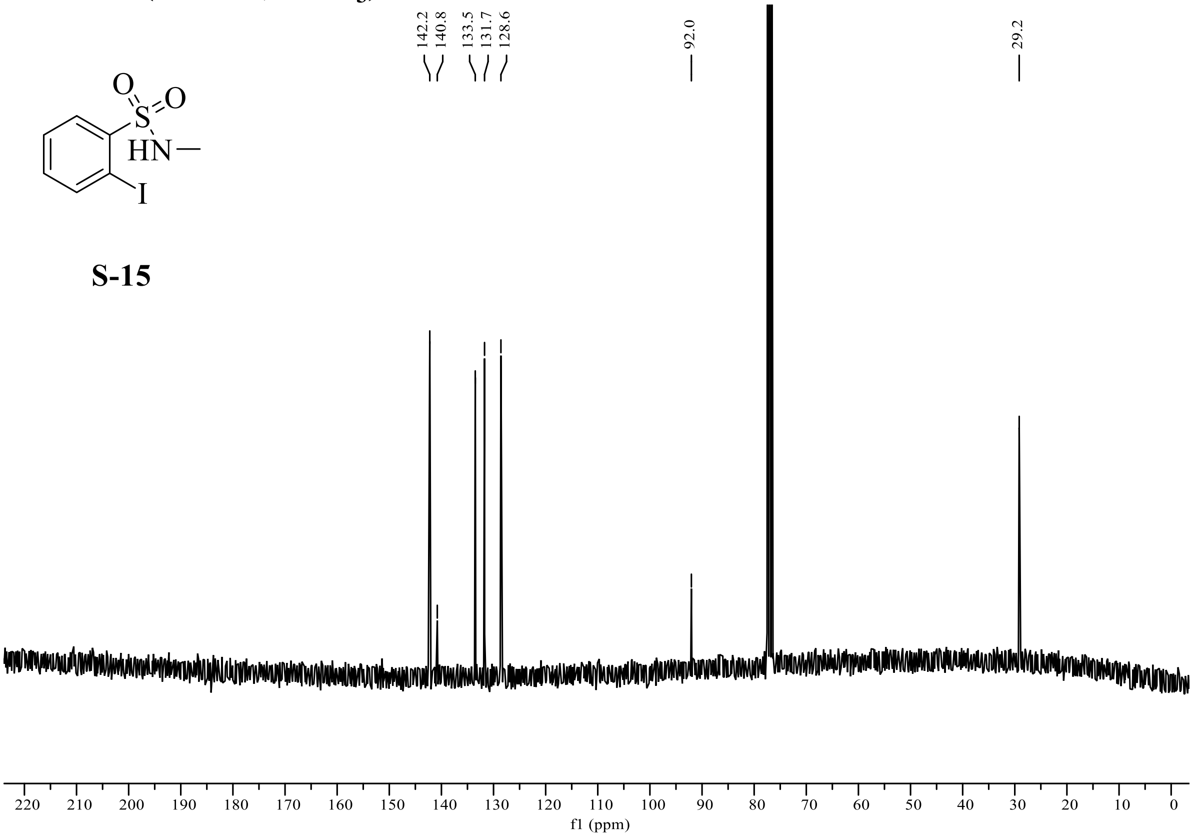

S-15

^{1}H NMR (300 MHz, $CDCl_3$):

S-16

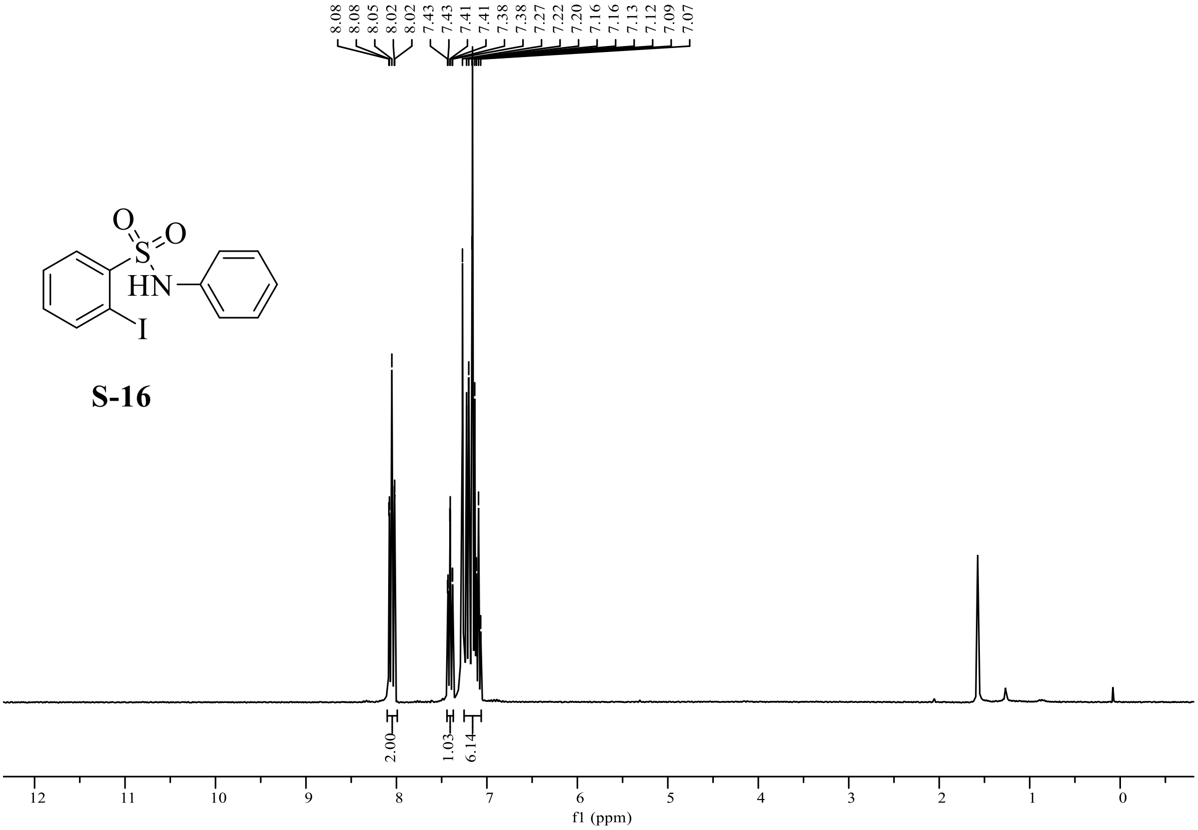

^{13}C NMR (75 MHz, $CDCl_3$):

S-16

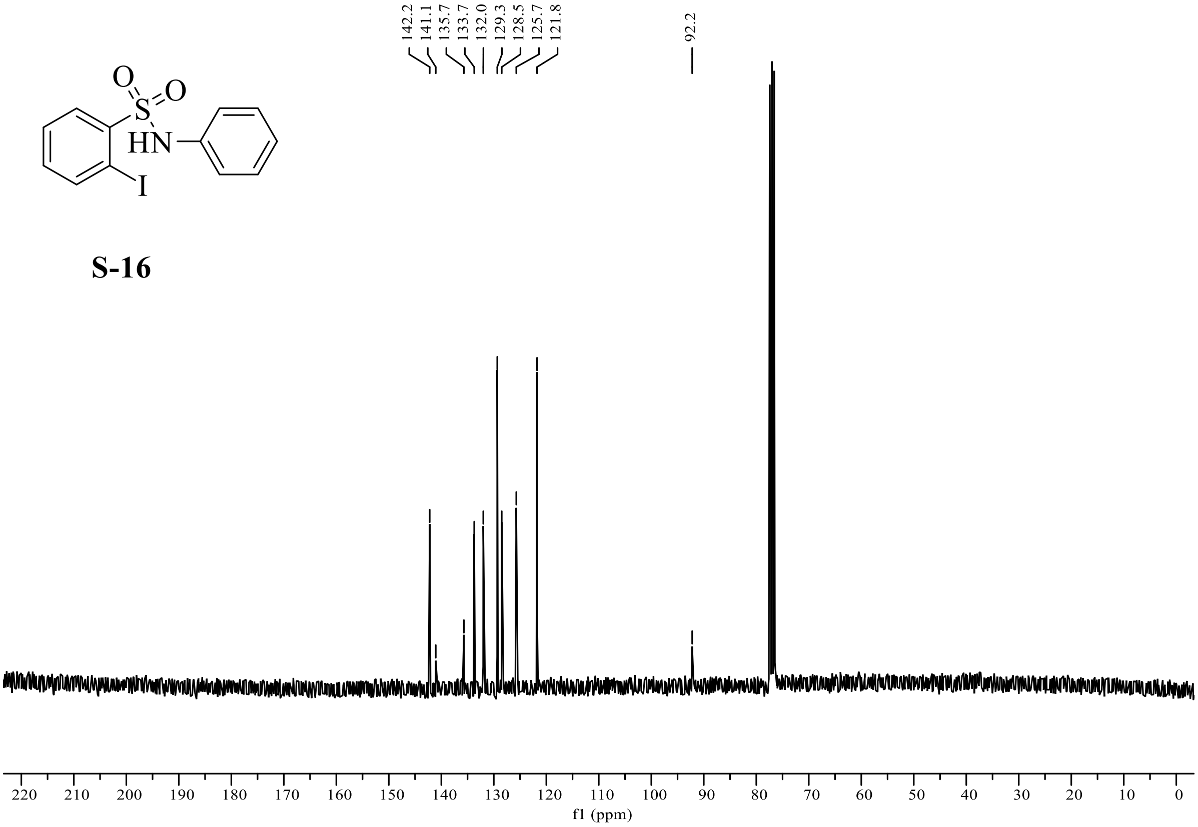

^{1}H NMR (300 MHz, $CDCl_3$):

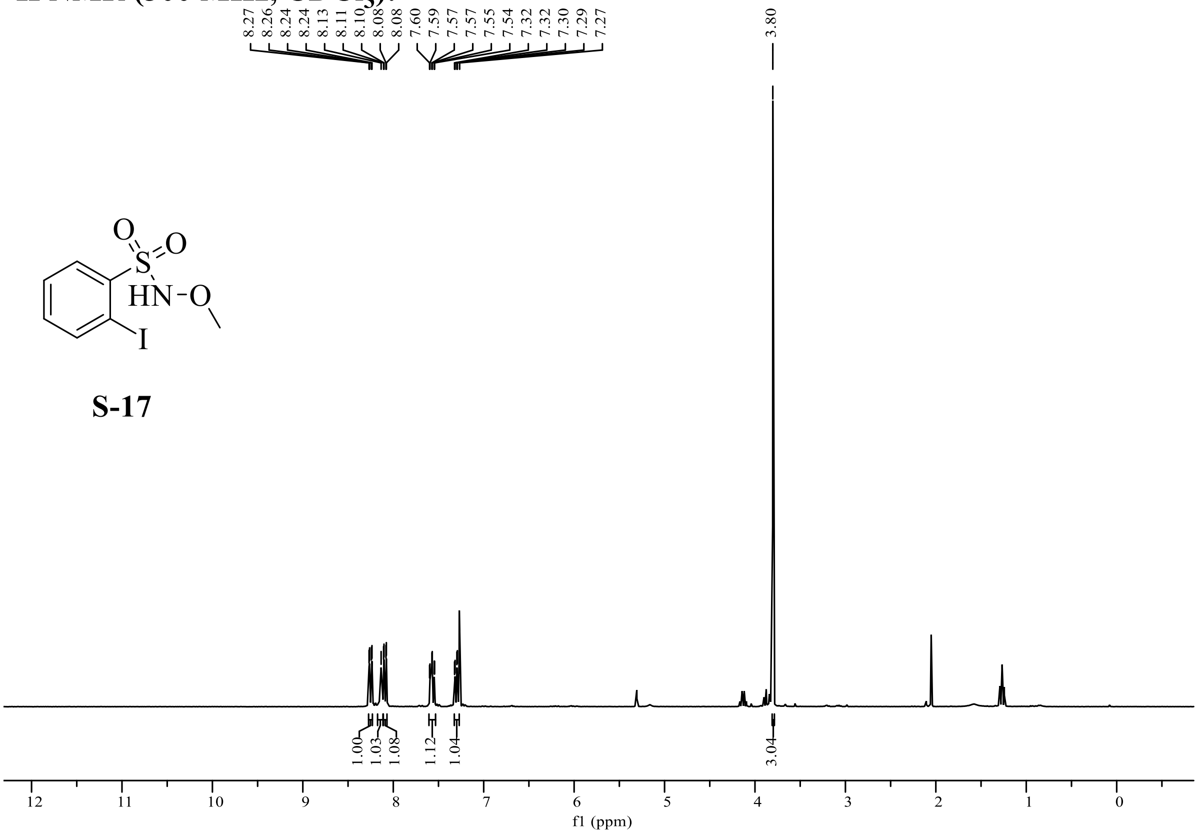

S-17

^{13}C NMR (75 MHz, $CDCl_3$):

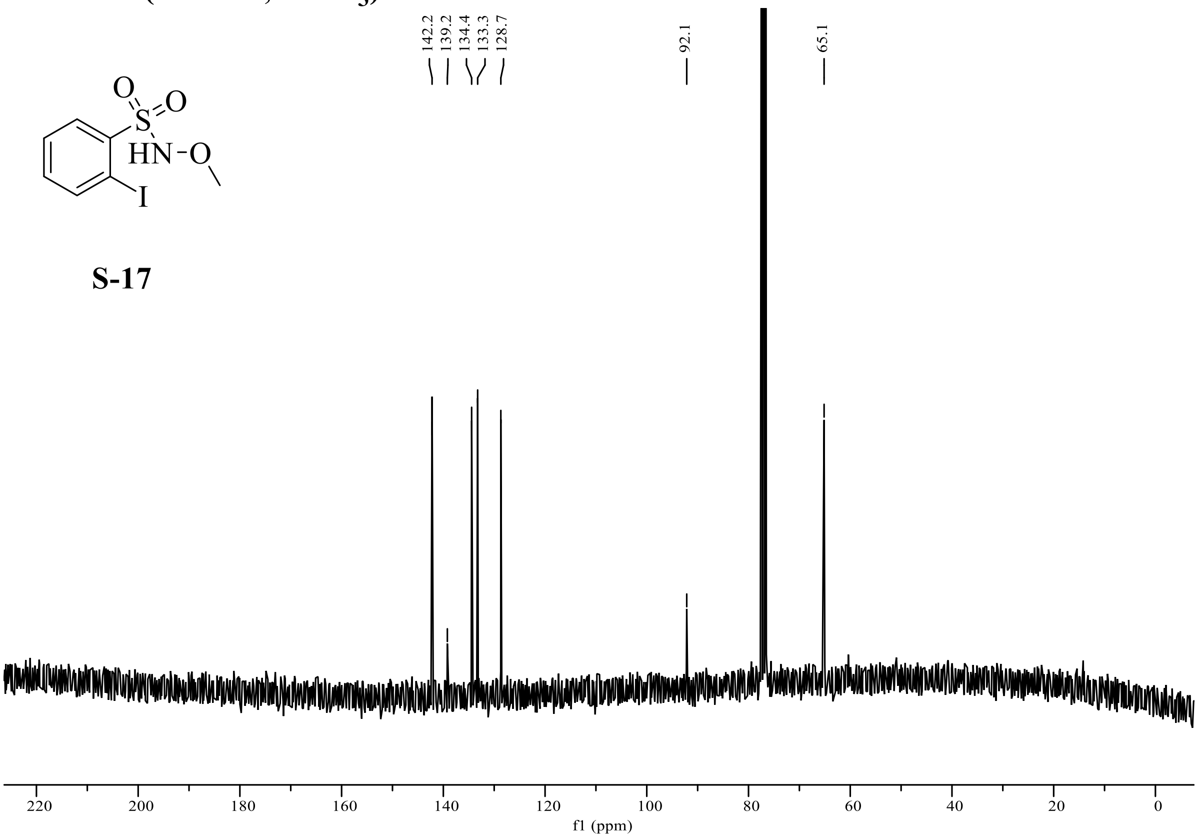

S-17

^{1}H NMR (300 MHz, $CDCl_3$):

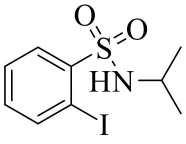

S-18

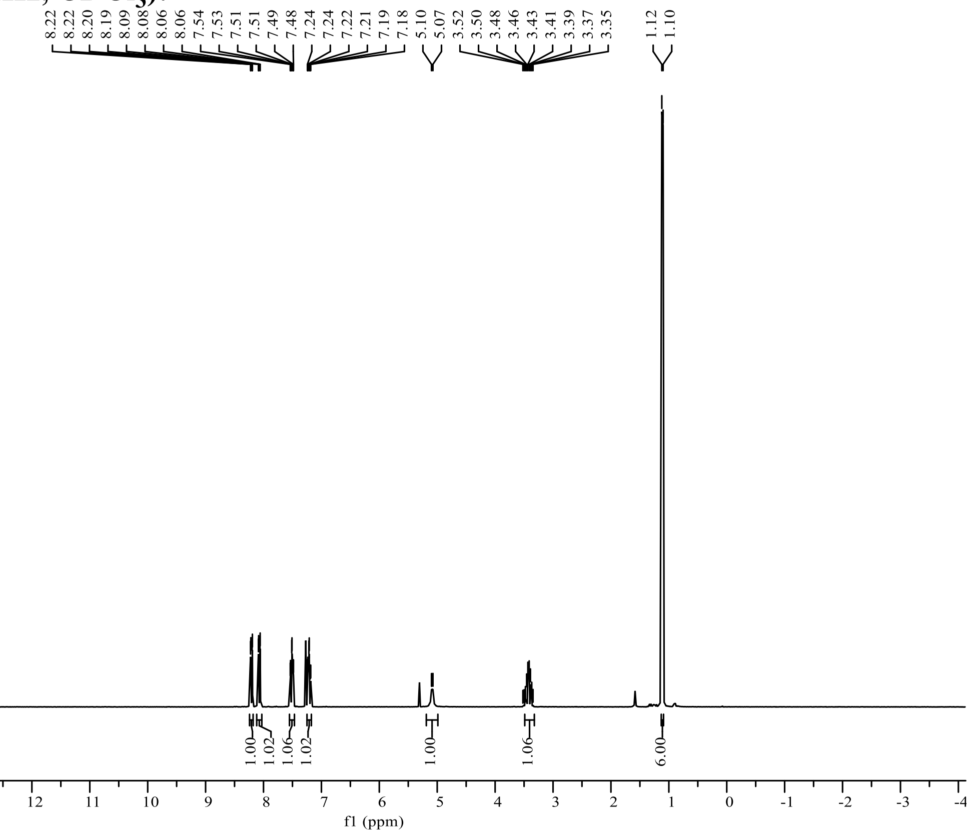

^{13}C NMR (75 MHz, $CDCl_3$):

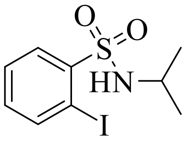

S-18

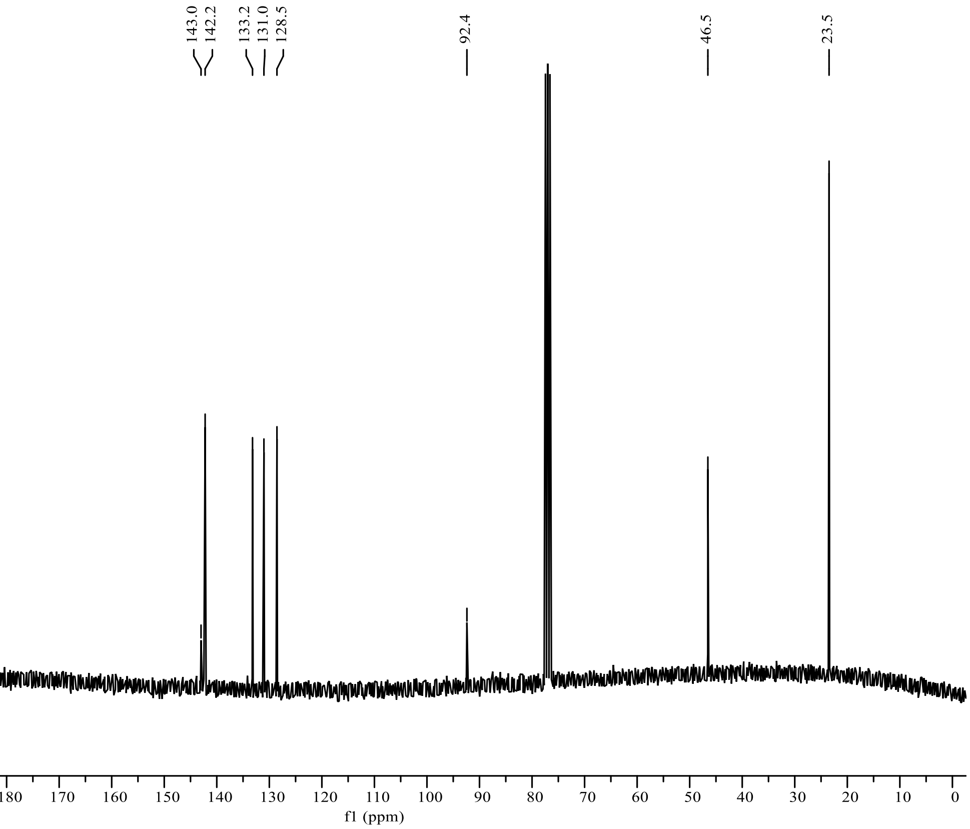

^{1}H NMR (300 MHz, $CDCl_3$):

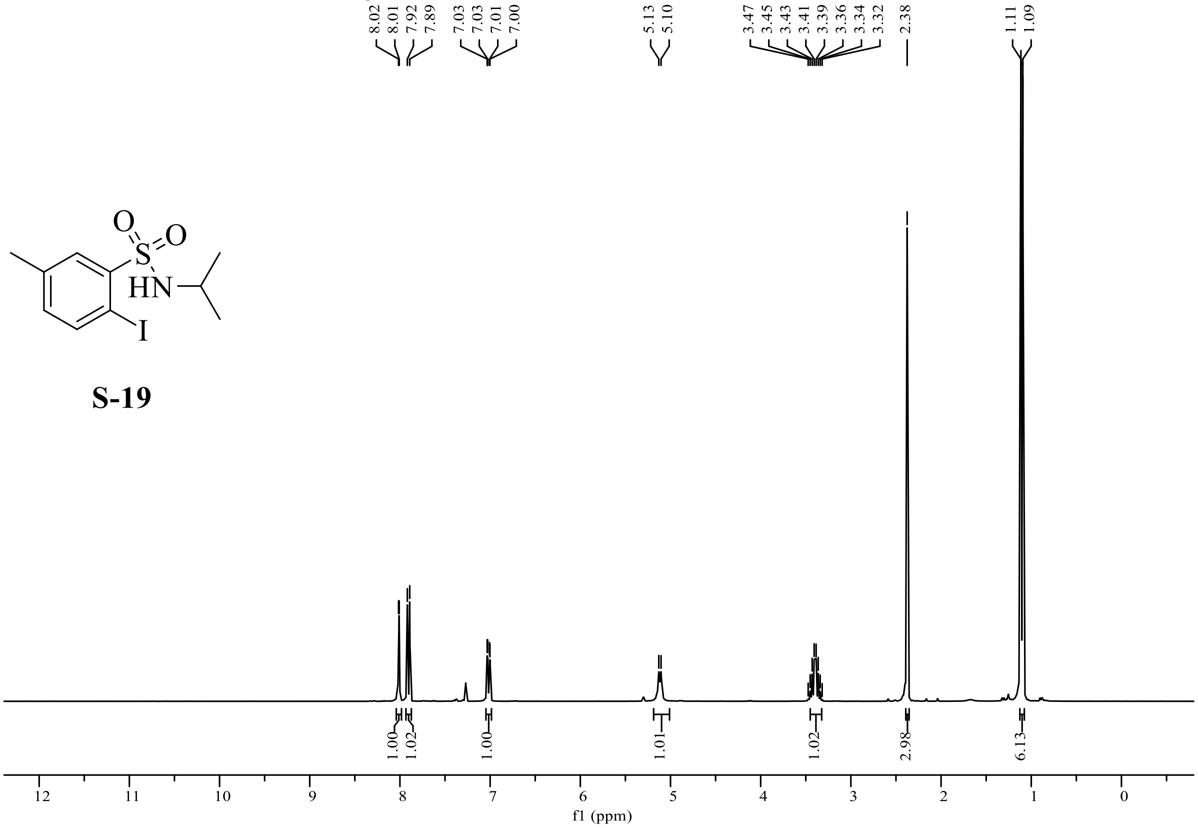

S-19

^{13}C NMR (75 MHz, $CDCl_3$):

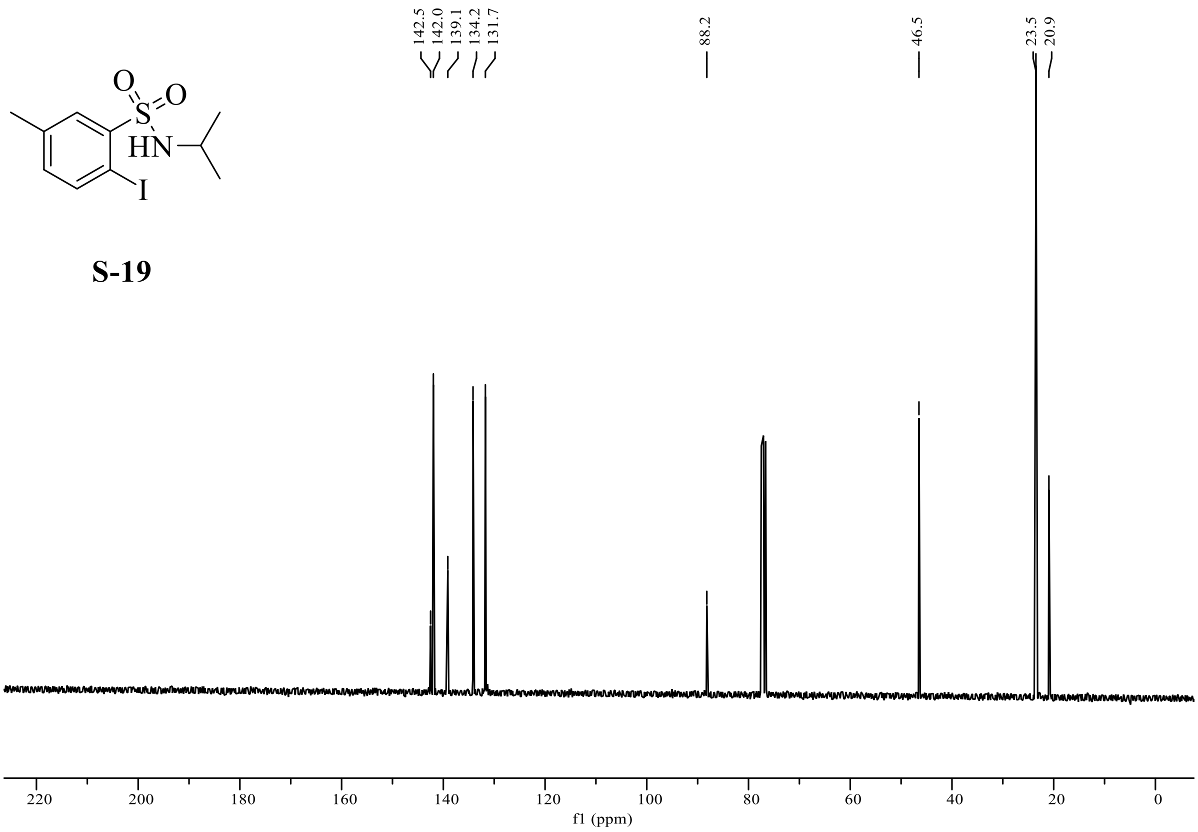

S-19

^{1}H NMR (300 MHz, $CDCl_3$):

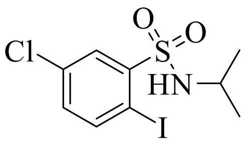

S-20

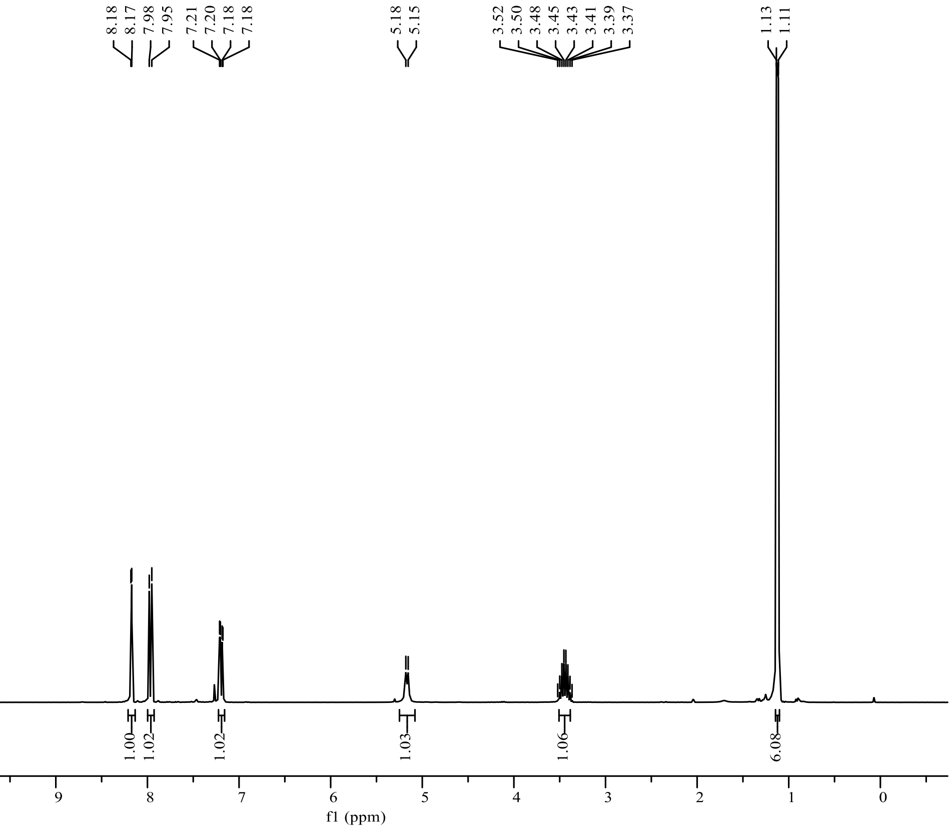

^{13}C NMR (75 MHz, $CDCl_3$):

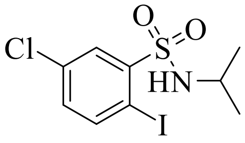

S-20

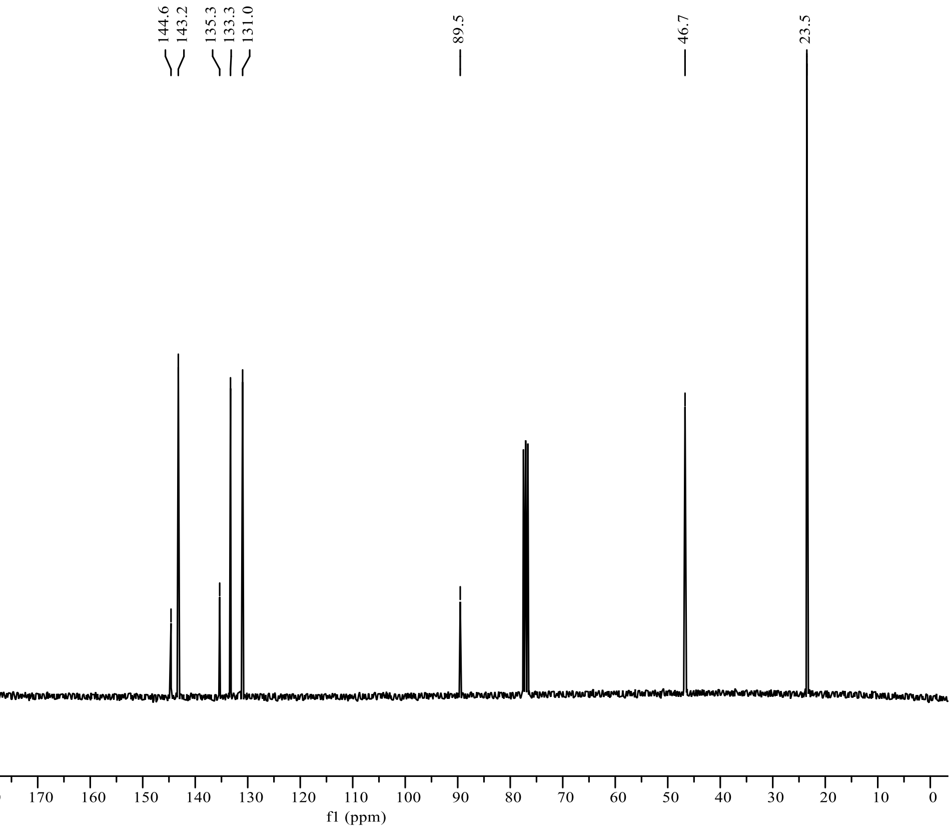

^{1}H NMR (300 MHz, $CDCl_3$):

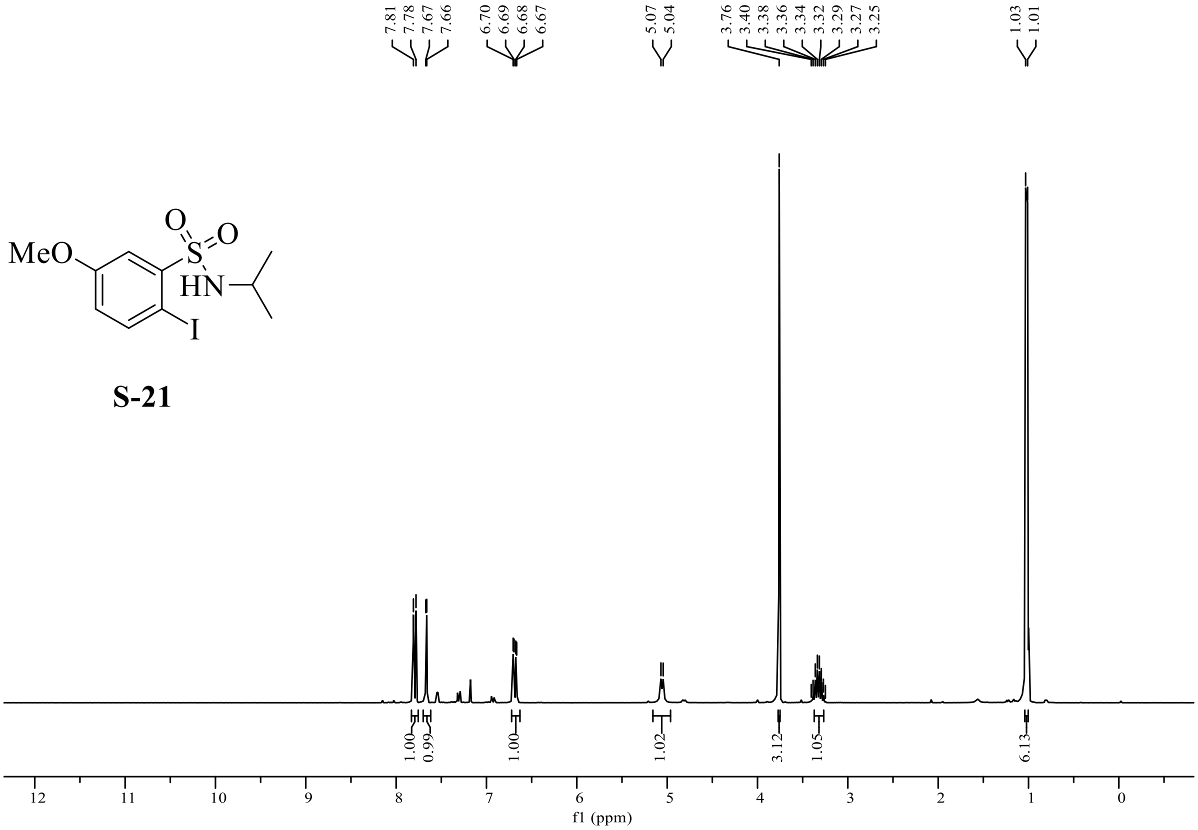

^{13}C NMR (75 MHz, $CDCl_3$):

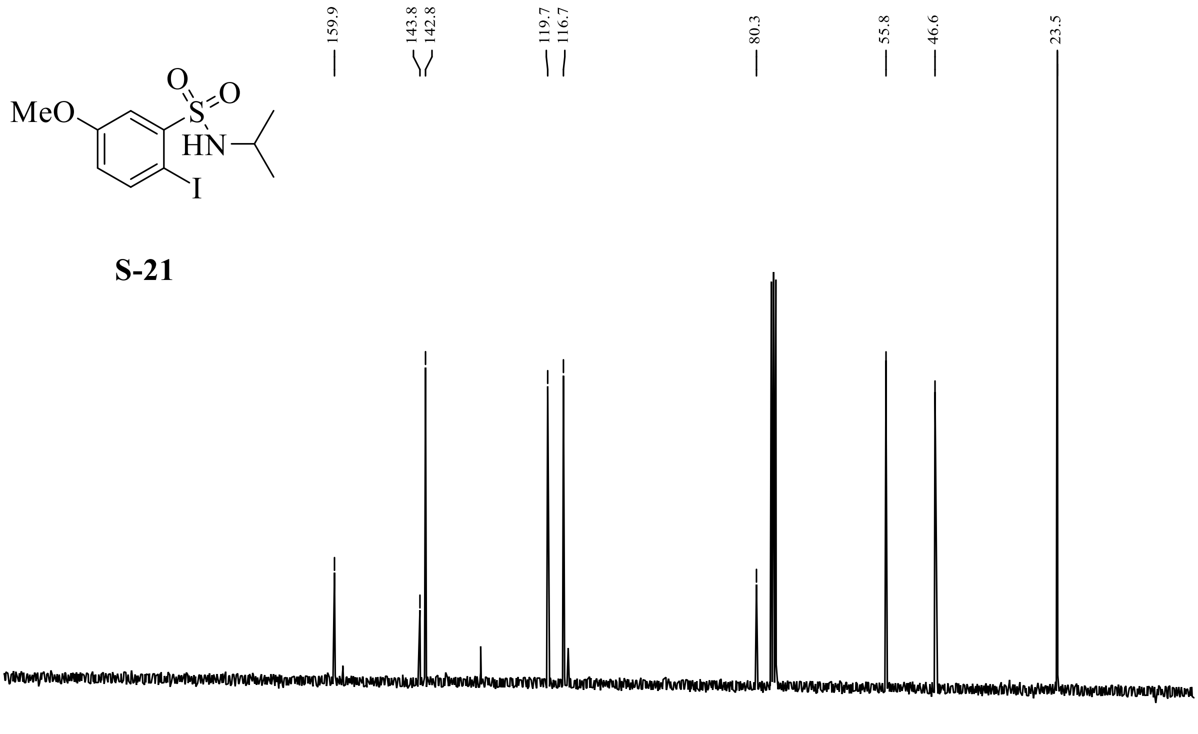

^{1}H NMR (300 MHz, $CDCl_3$):

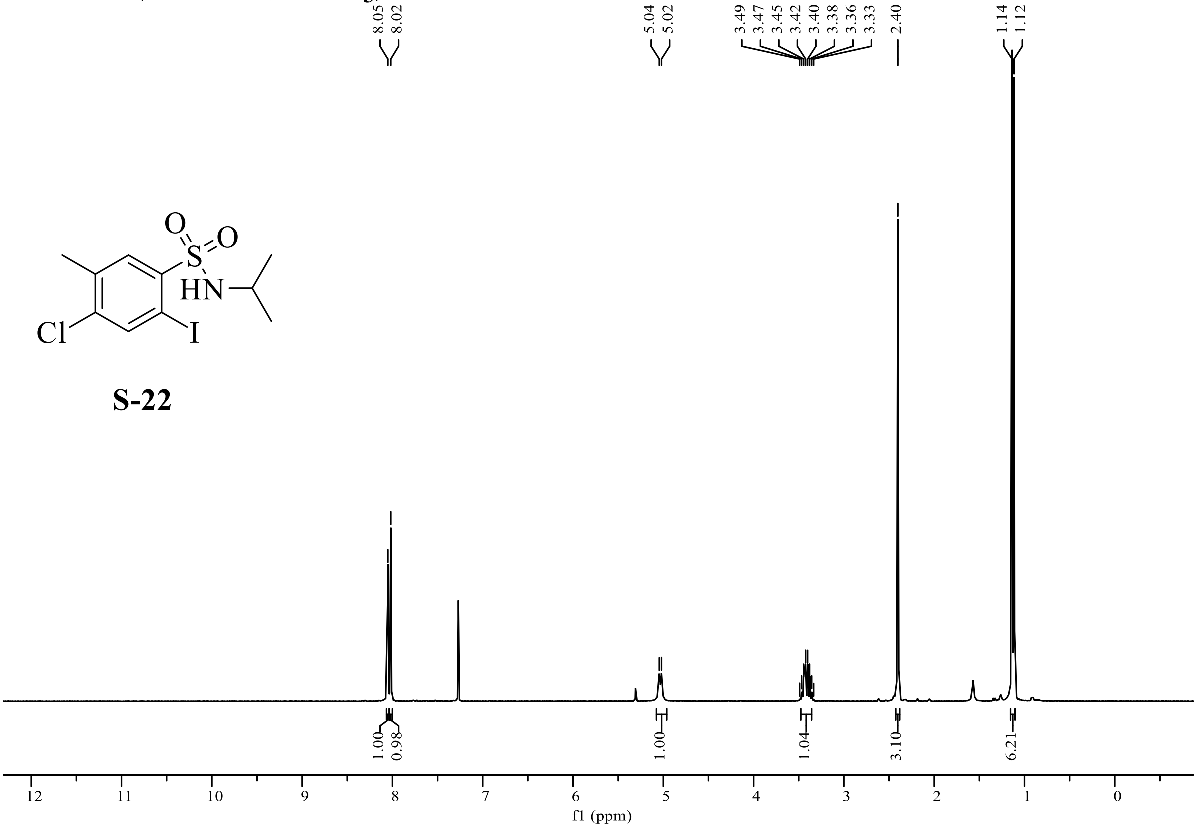

^{13}C NMR (75 MHz, $CDCl_3$):

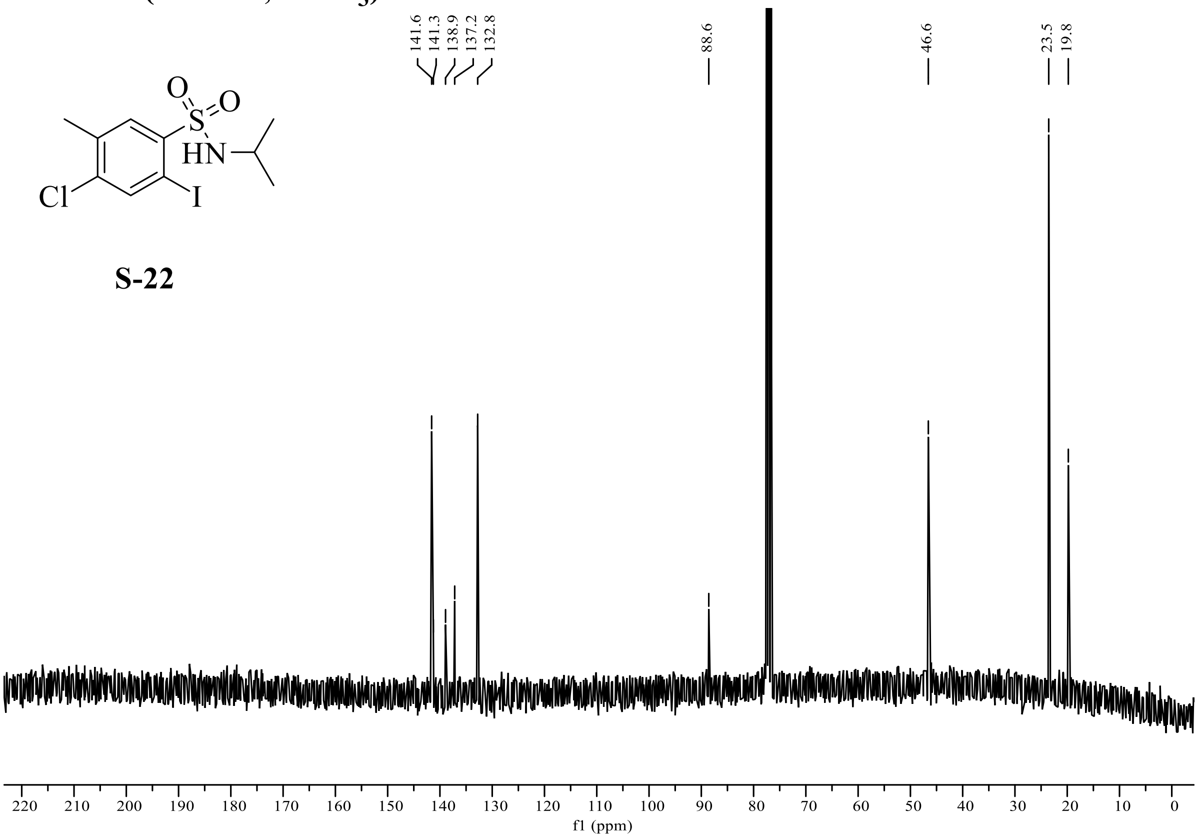

^{1}H NMR (300 MHz, $CDCl_3$):

S-23

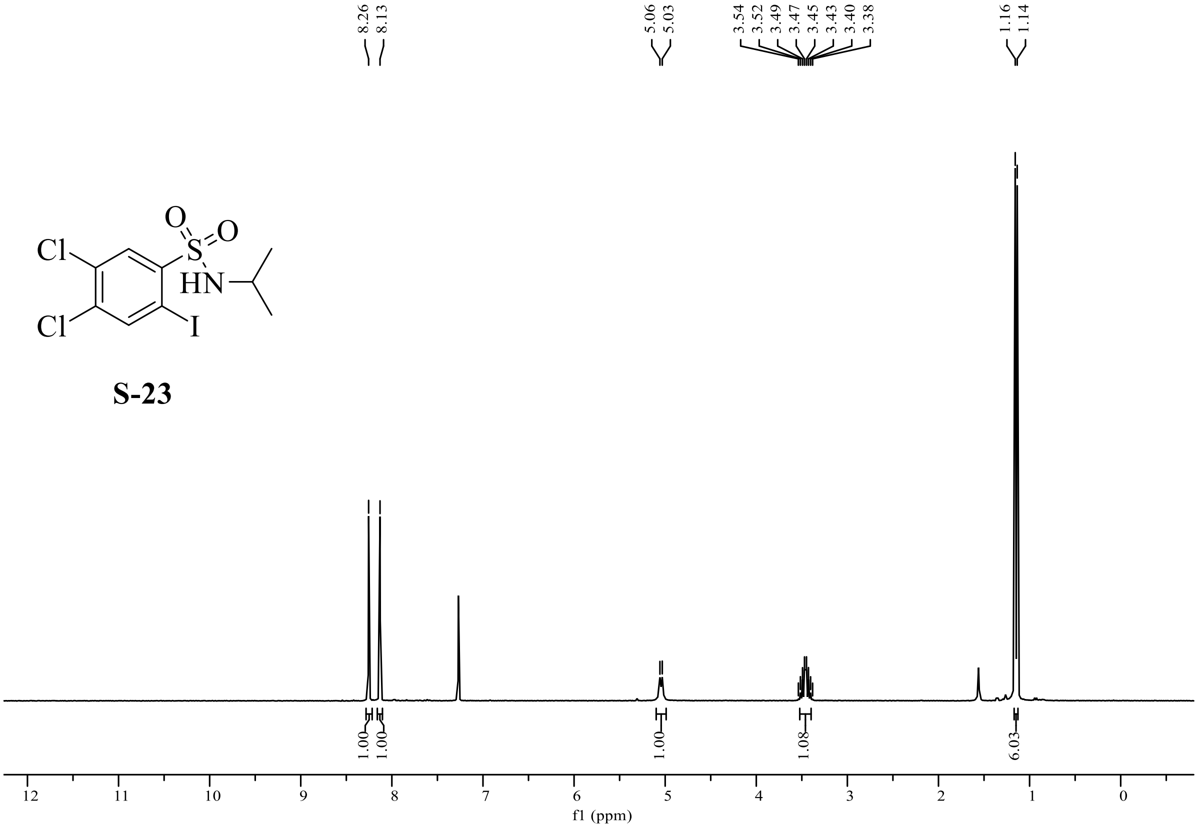

^{13}C NMR (75 MHz, $CDCl_3$):

S-23

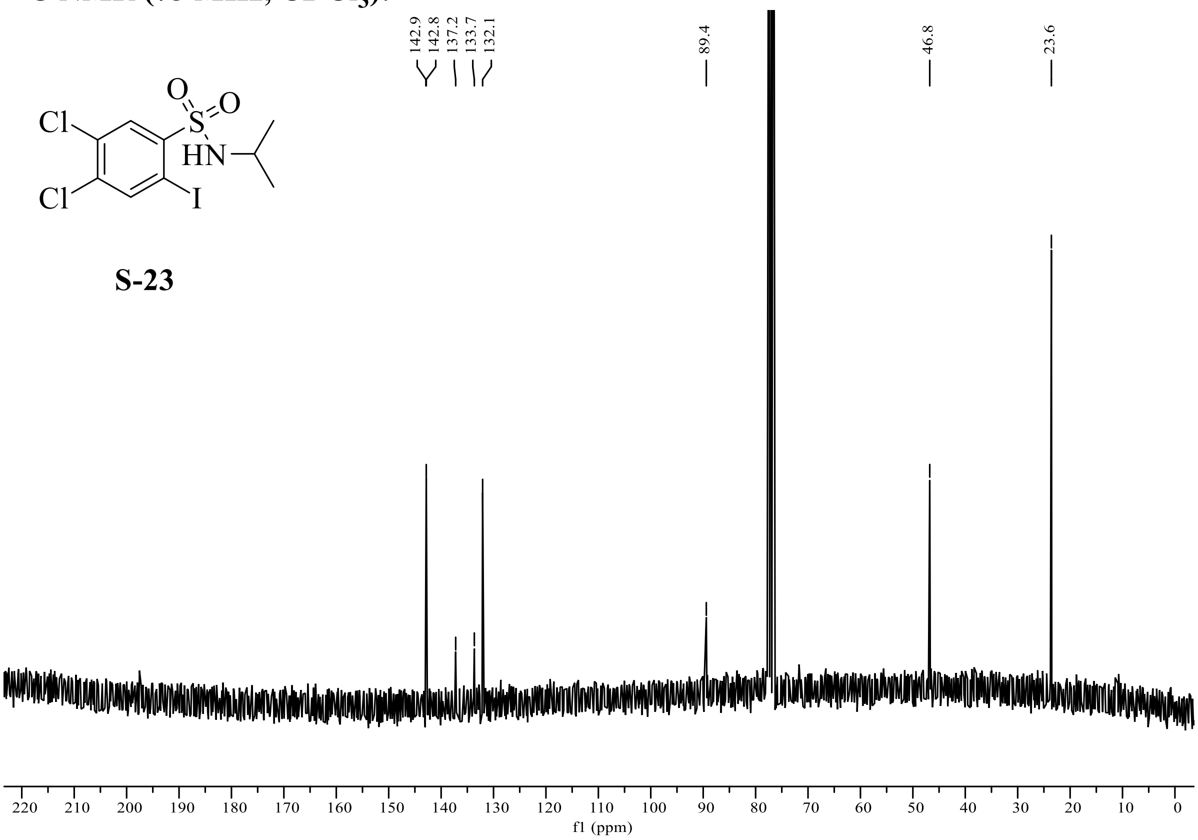

^{1}H NMR (600 MHz, CDCl$_3$):

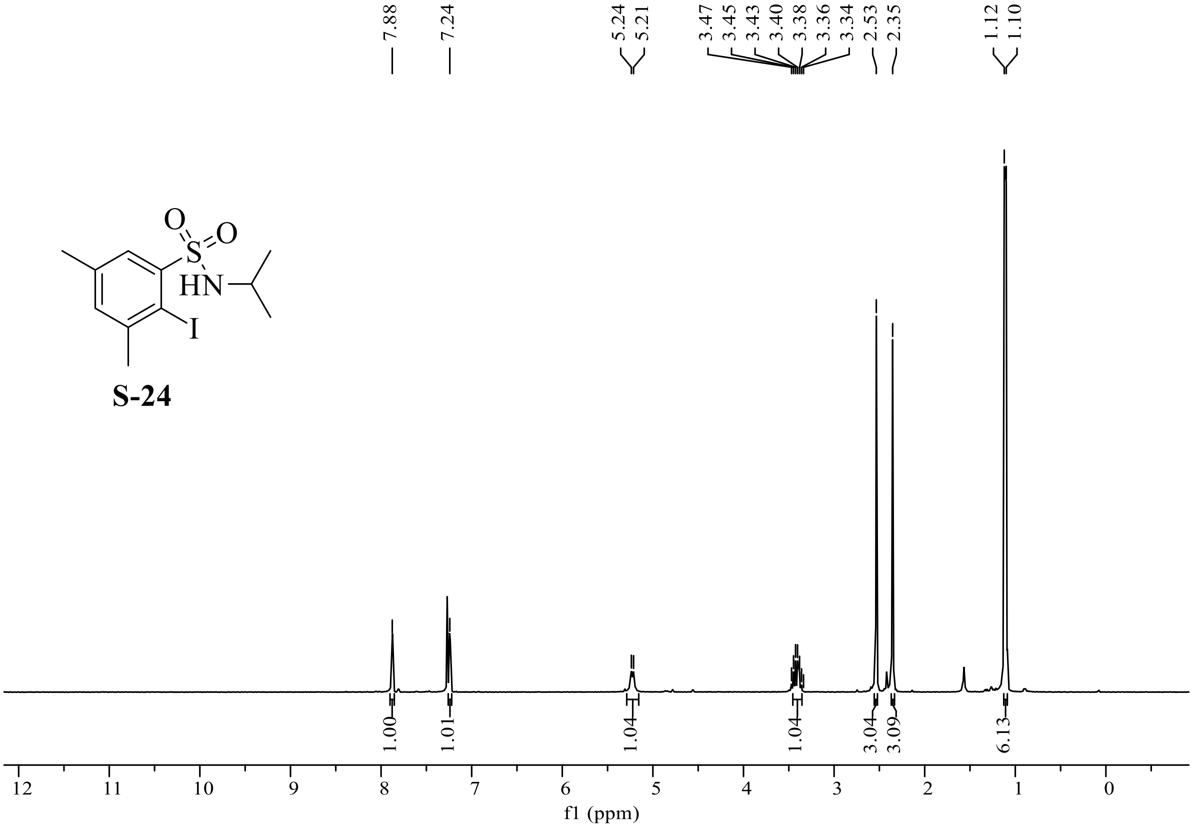

S-24

^{13}C NMR (151 MHz, CDCl$_3$):

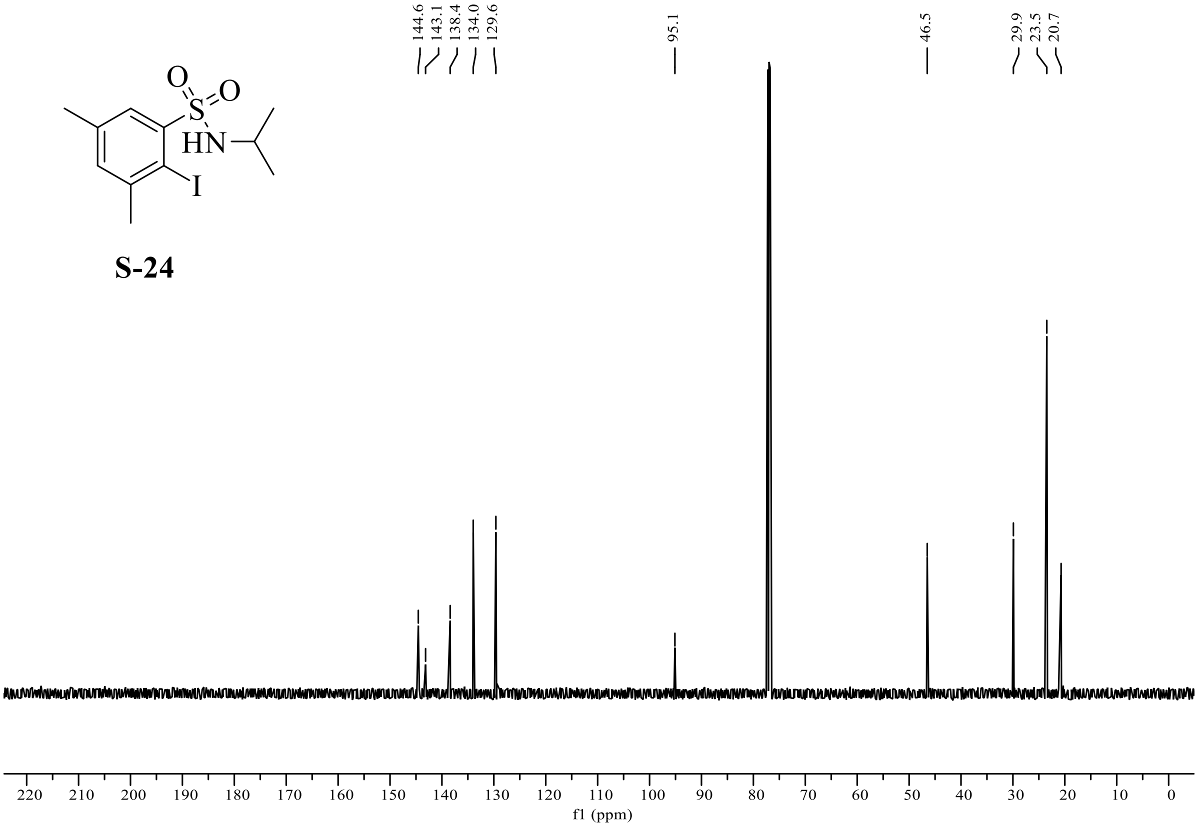

S-24

^{1}H NMR (300 MHz, $CDCl_3$):

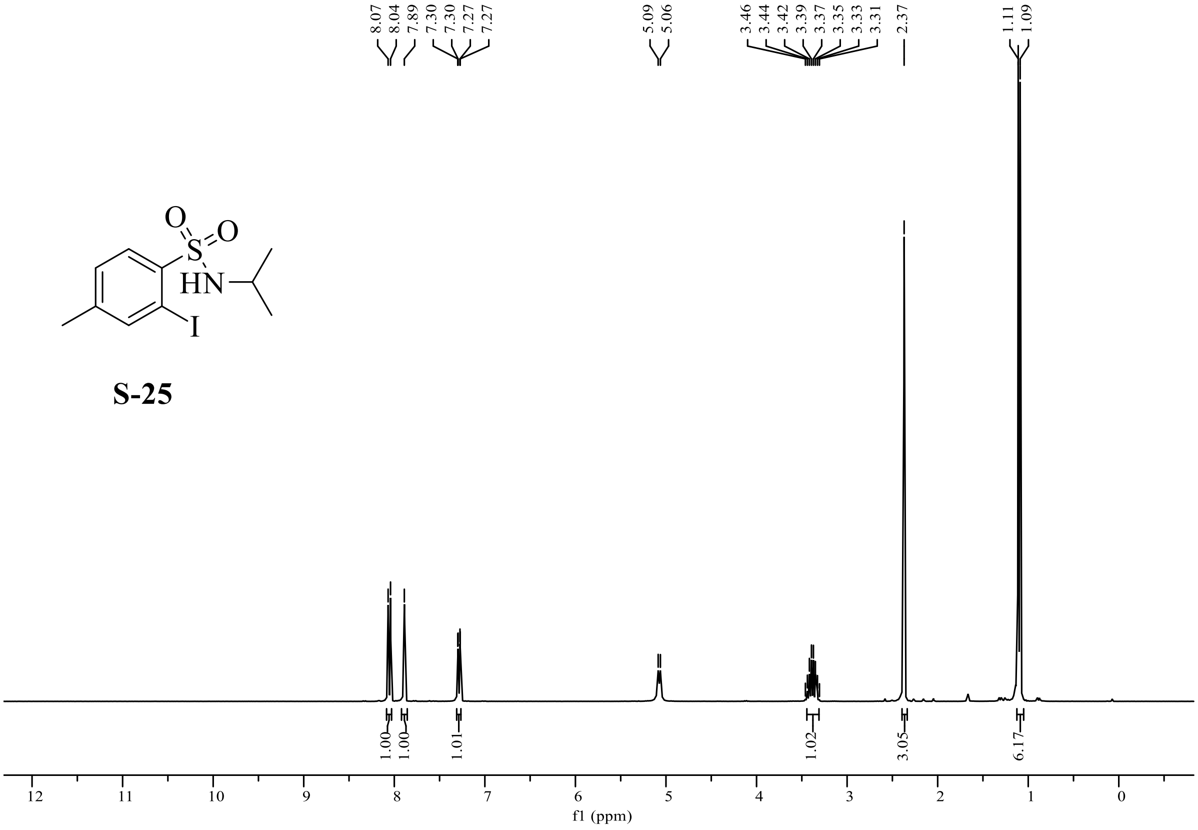

S-25

^{13}C NMR (75 MHz, $CDCl_3$):

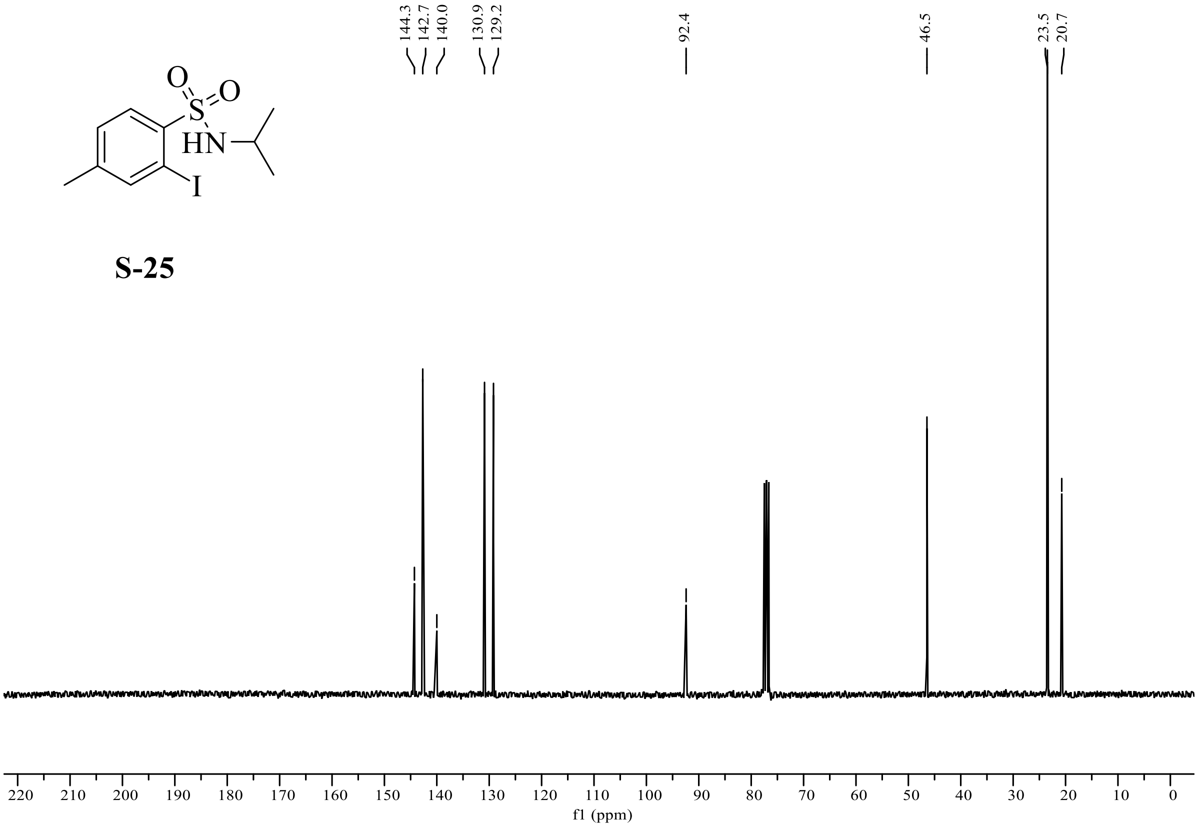

S-25

^{1}H NMR (600 MHz, CDCl$_3$):

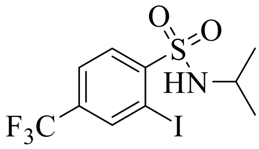

S-26

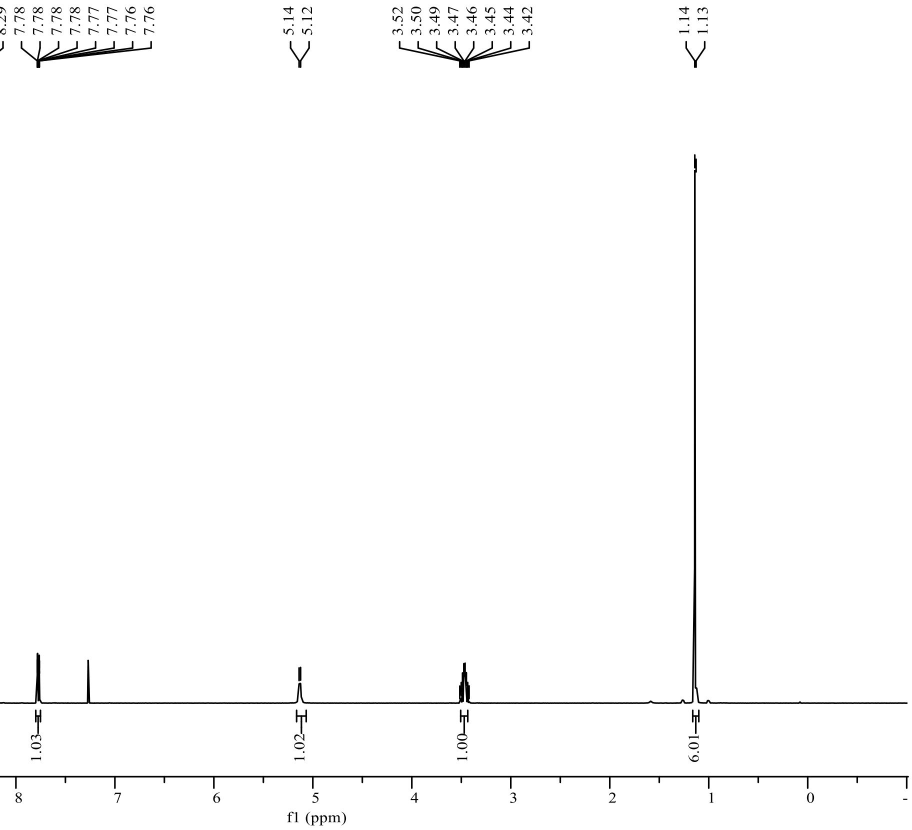

^{13}C{^{19}F} NMR (151 MHz, CDCl$_3$):

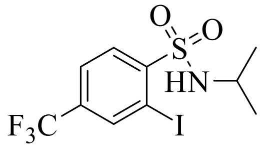

S-26

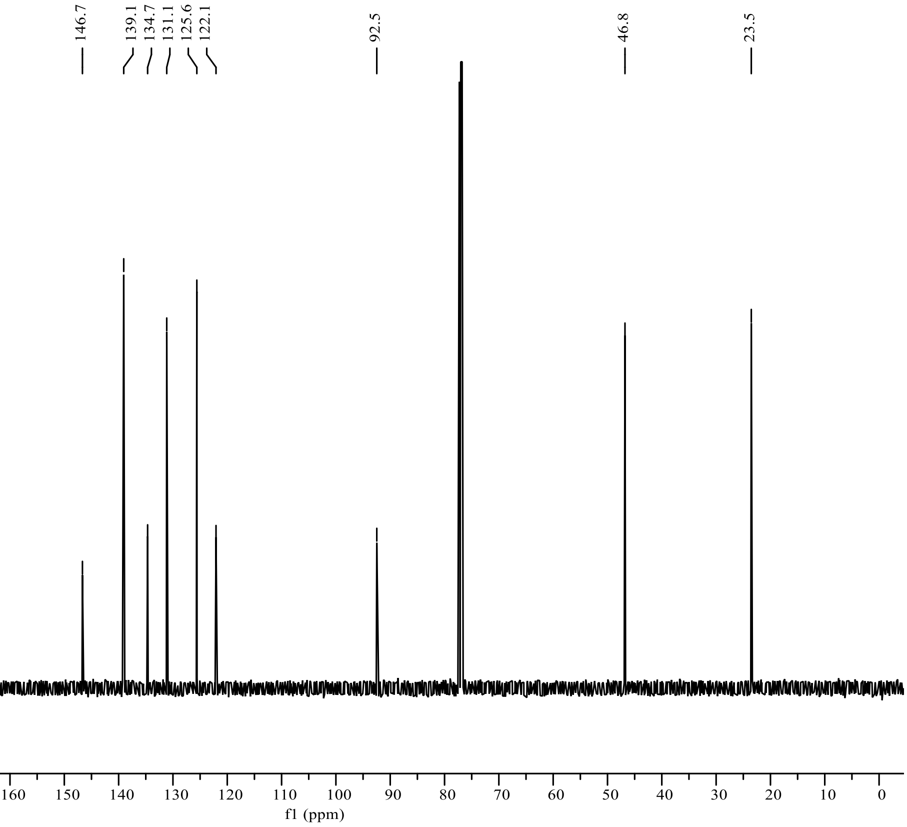

$^{19}F\{^{1}H\}$ NMR (470 MHz, $CDCl_3$):

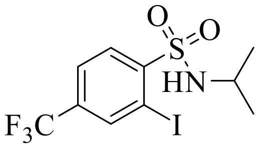

S-26

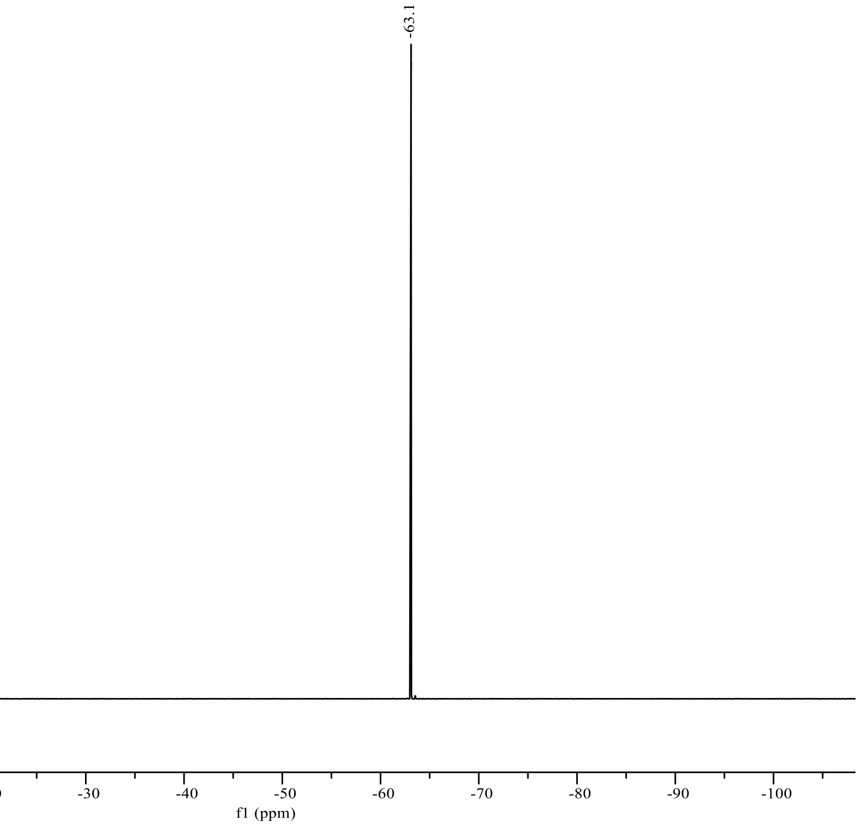

^{1}H NMR (600 MHz, $CDCl_3$):

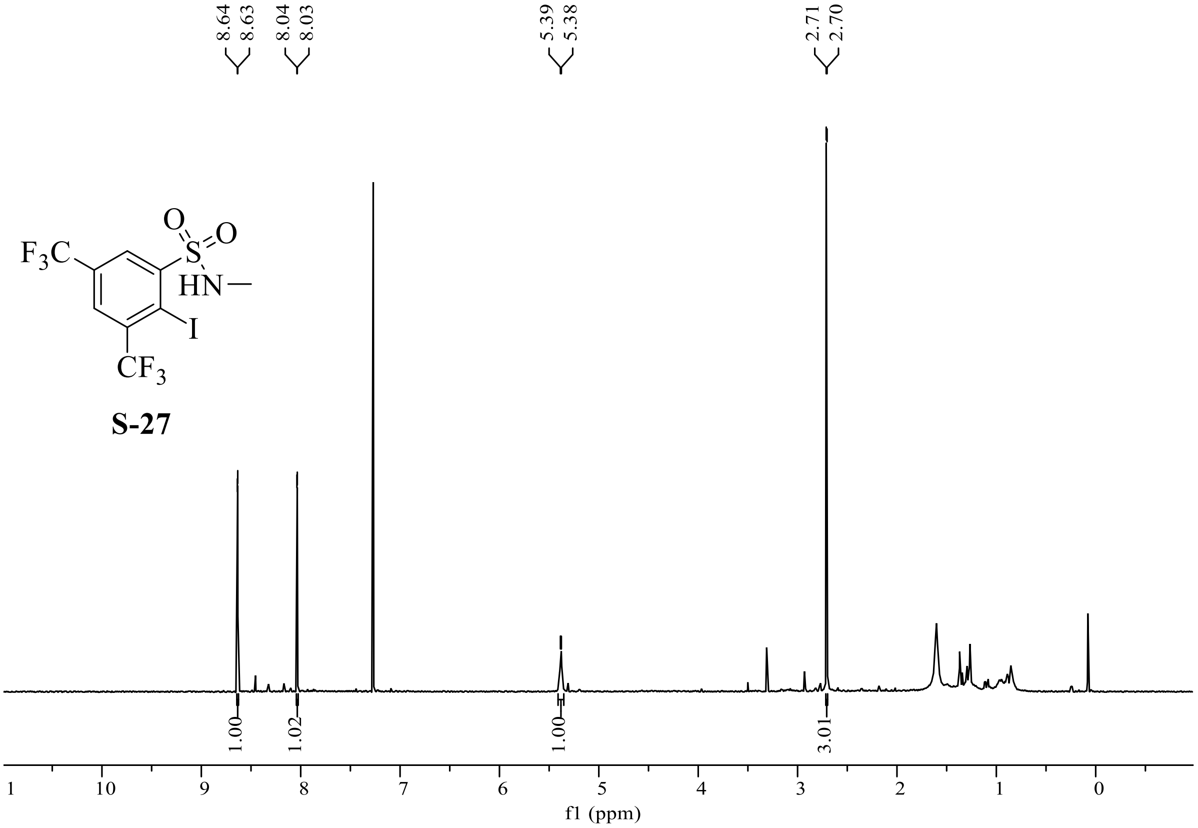

$^{13}C\{^{19}F\}$ NMR (151 MHz, $CDCl_3$):

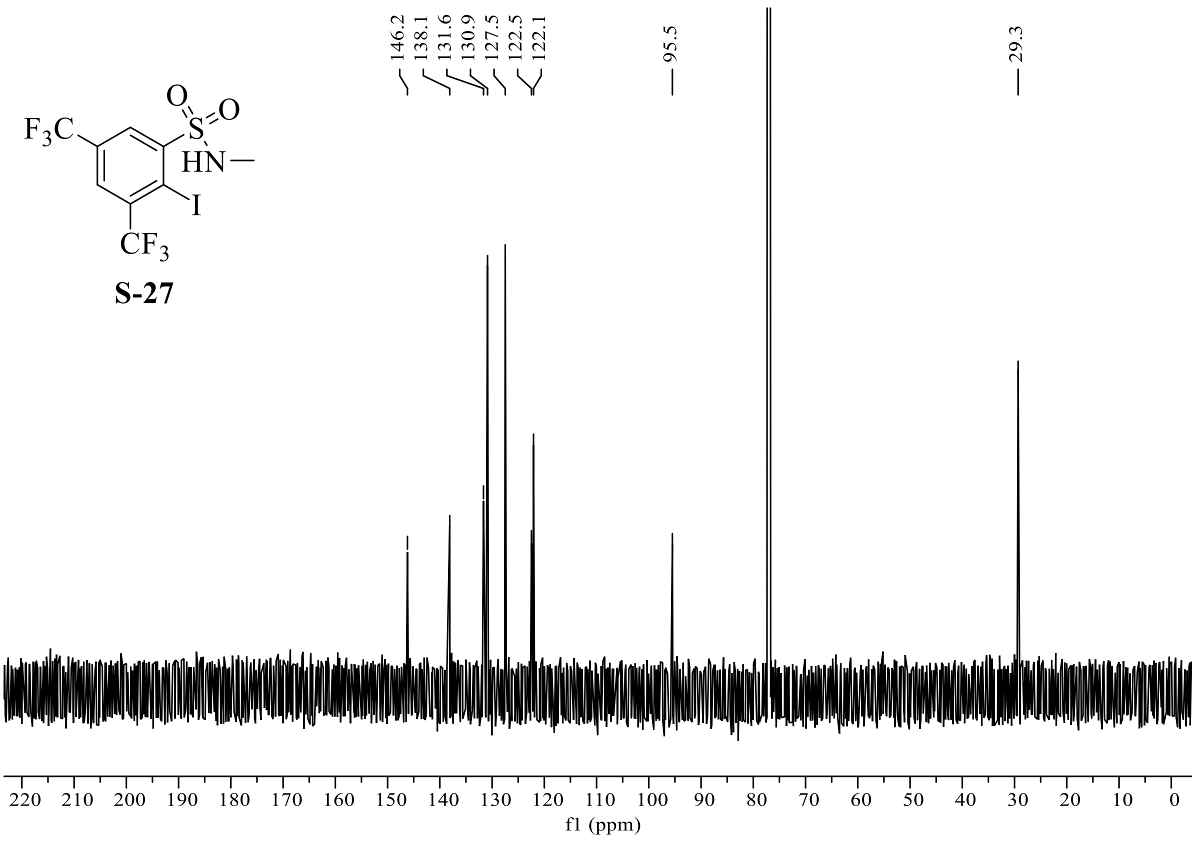

$^{19}F\{^{1}H\}$ NMR (470 MHz, $CDCl_3$):

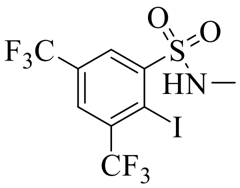

S-27

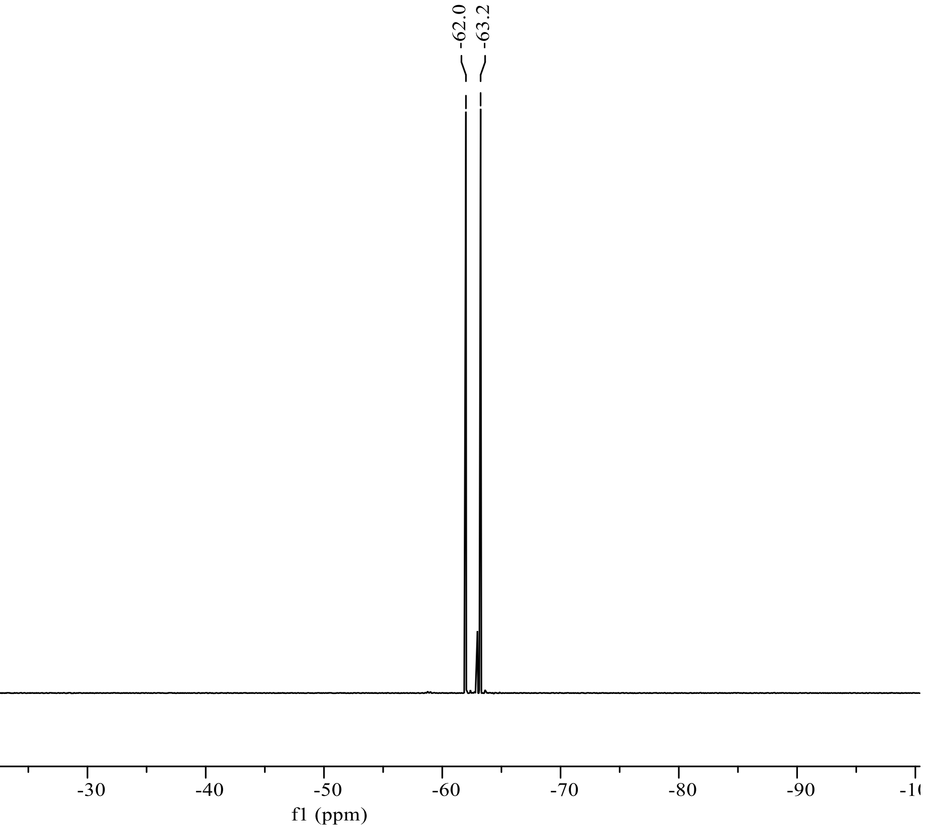

^{1}H NMR (300 MHz, $CDCl_3$):

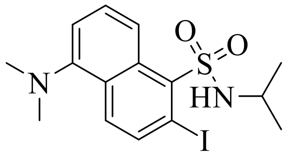

S-28

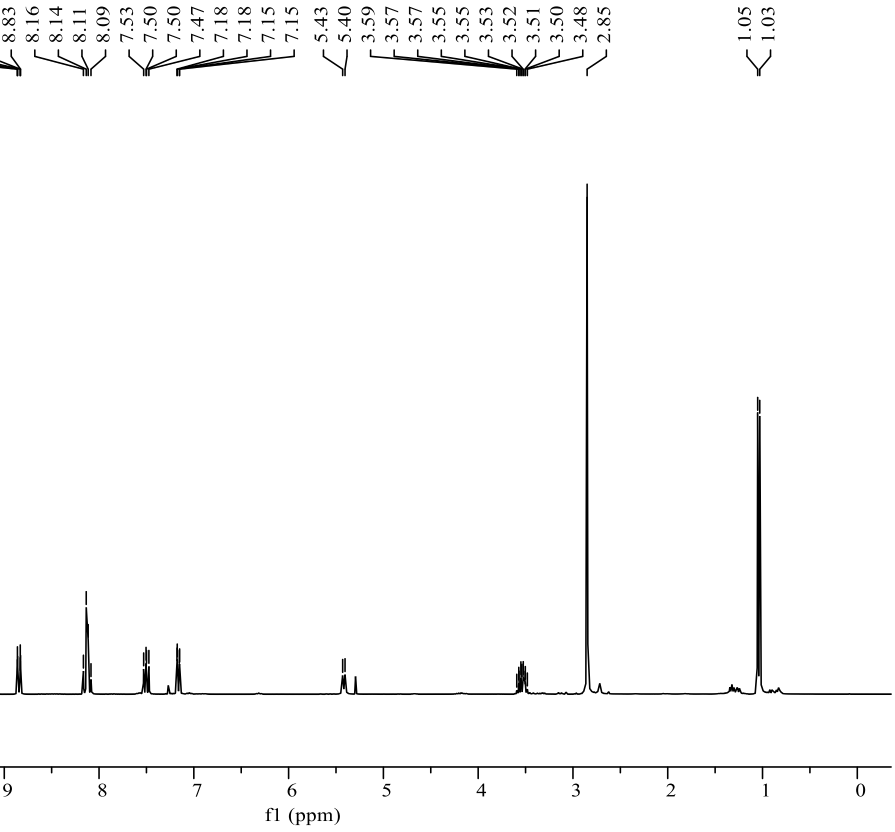

^{13}C NMR (75 MHz, $CDCl_3$):

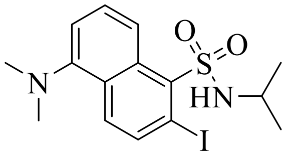

S-28

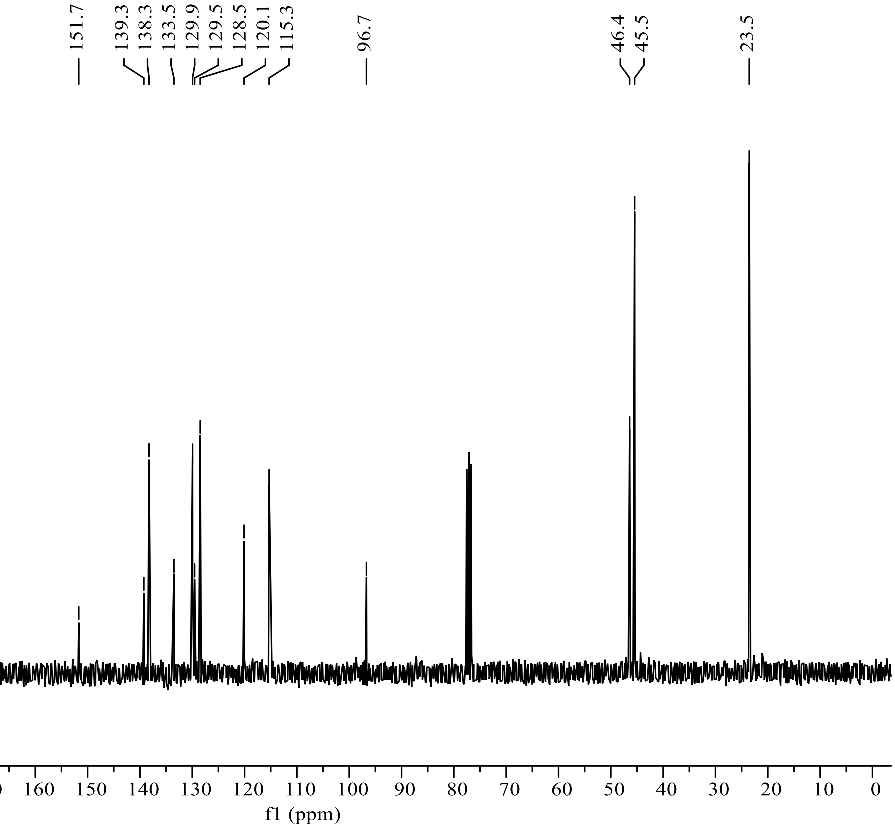

^{1}H NMR (600 MHz, $CDCl_3$):

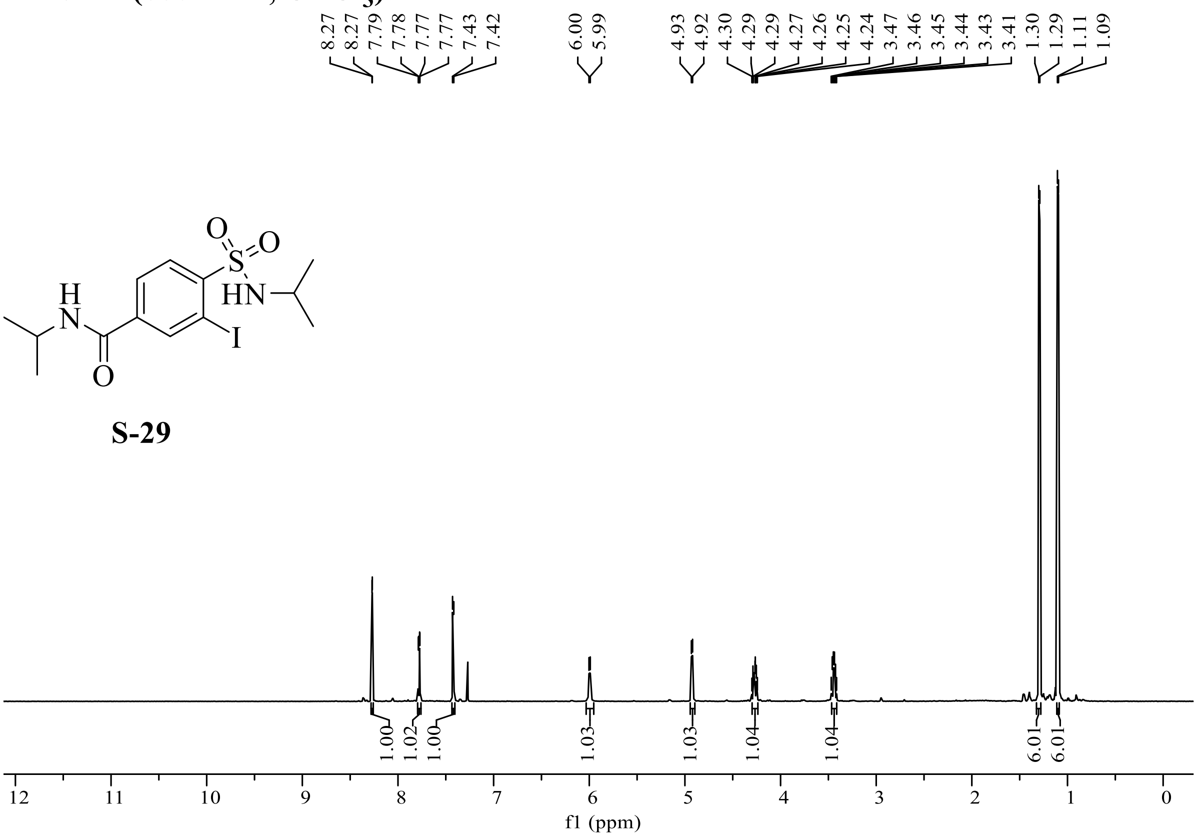

S-29

^{13}C NMR (151 MHz, $CDCl_3$):

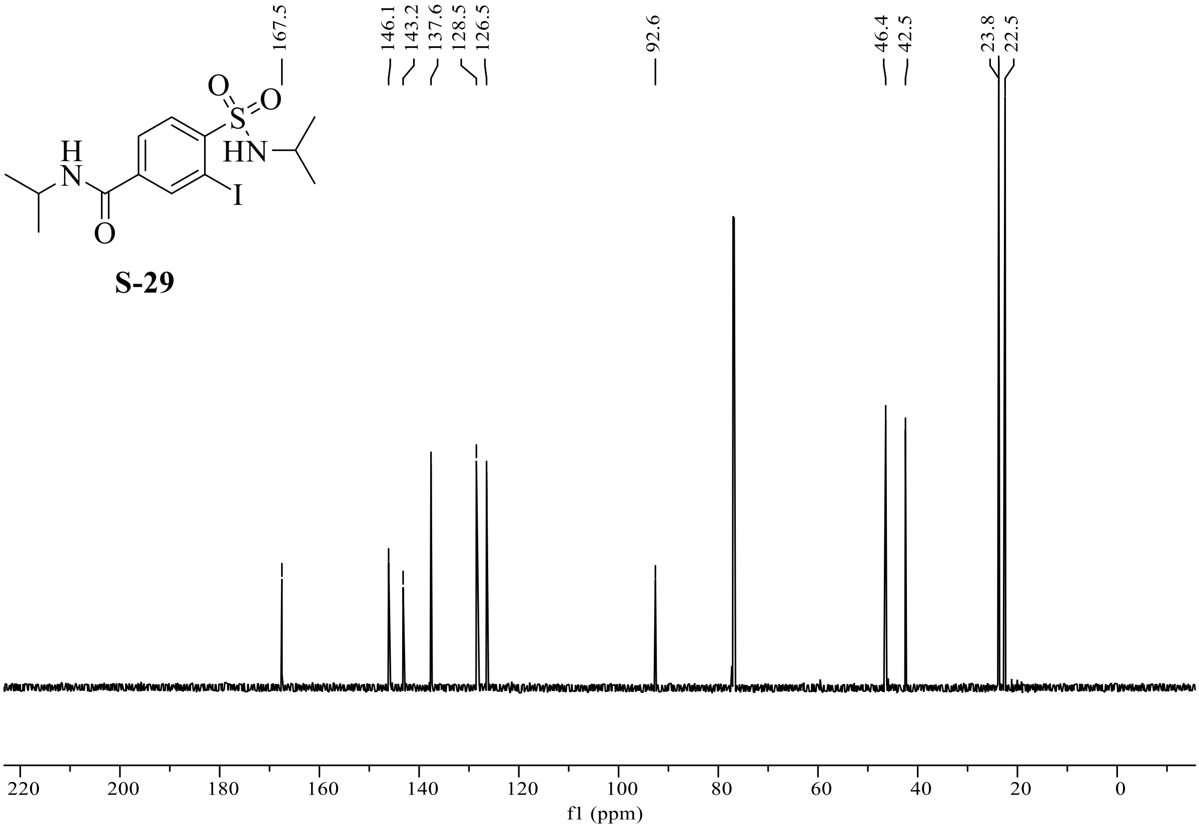

S-29

^{1}H NMR (300 MHz, $CDCl_3$):

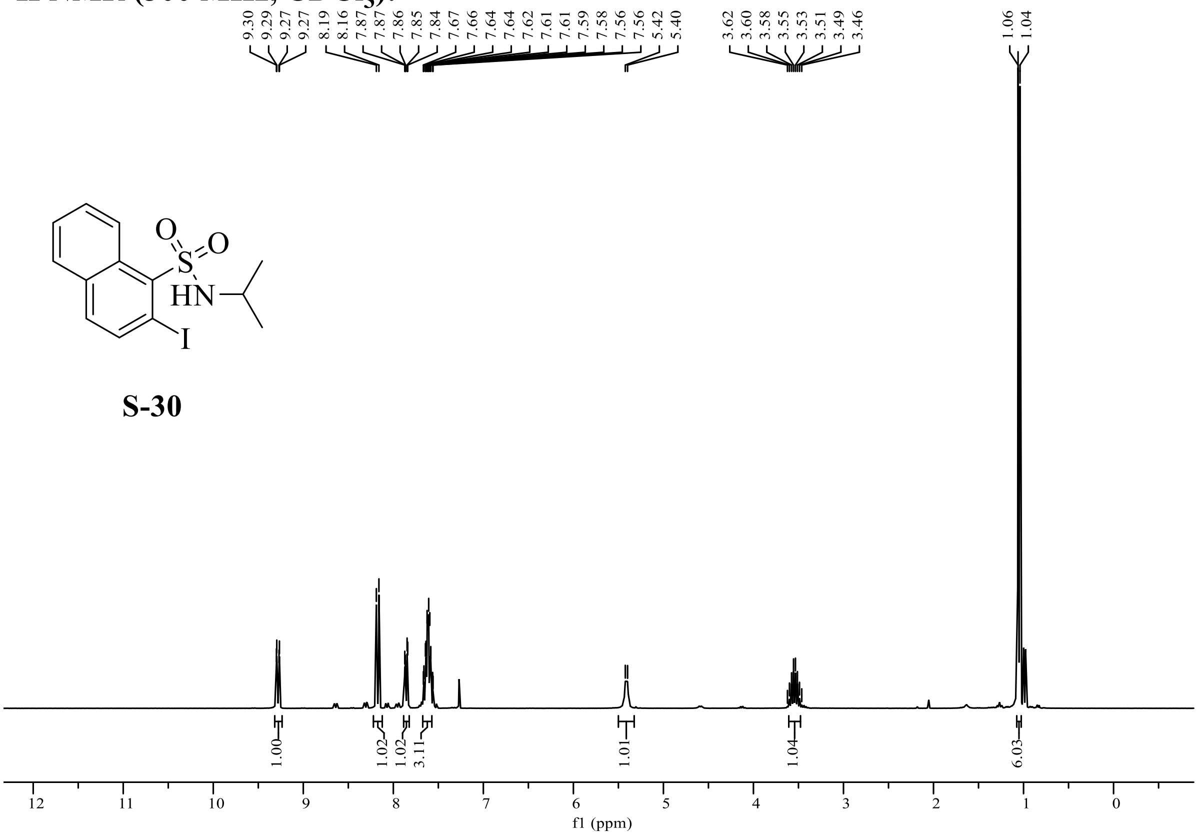

S-30

^{13}C NMR (75 MHz, $CDCl_3$):

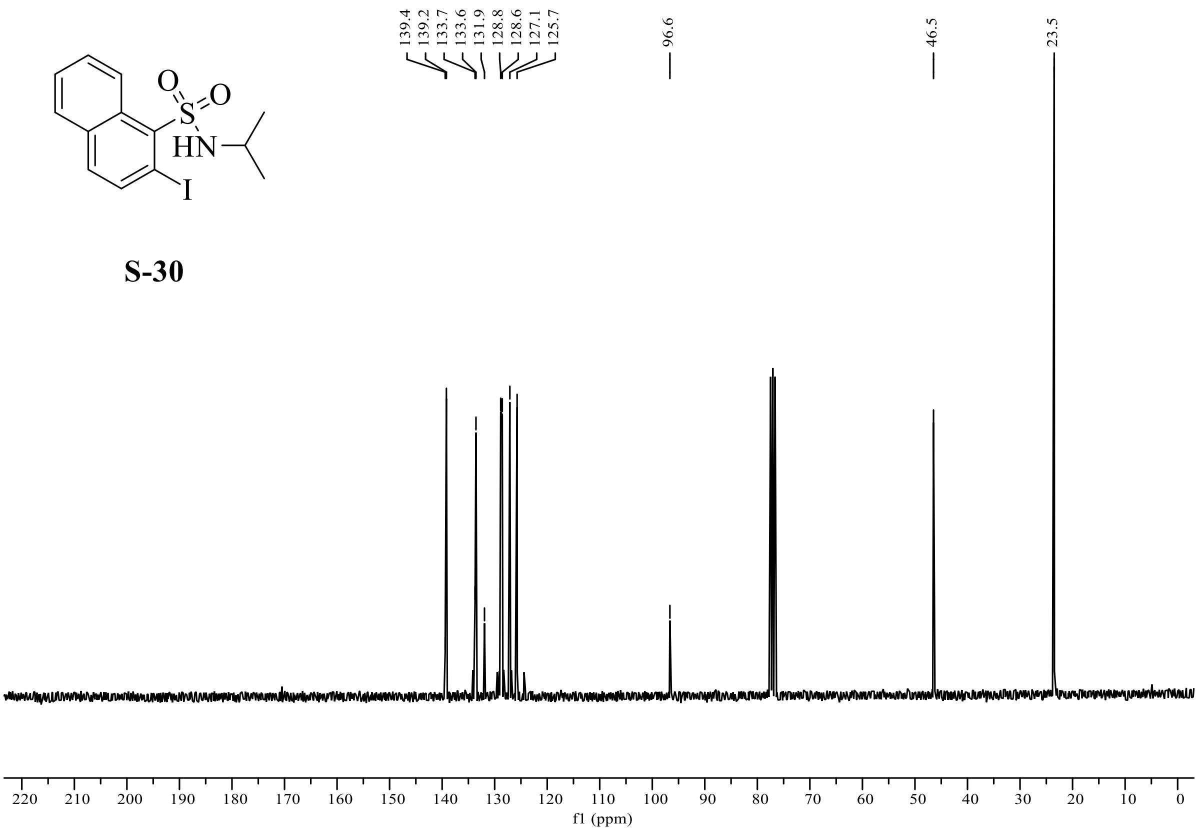

S-30

^{1}H NMR (300 MHz, $CDCl_3$):

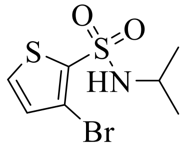

S-31

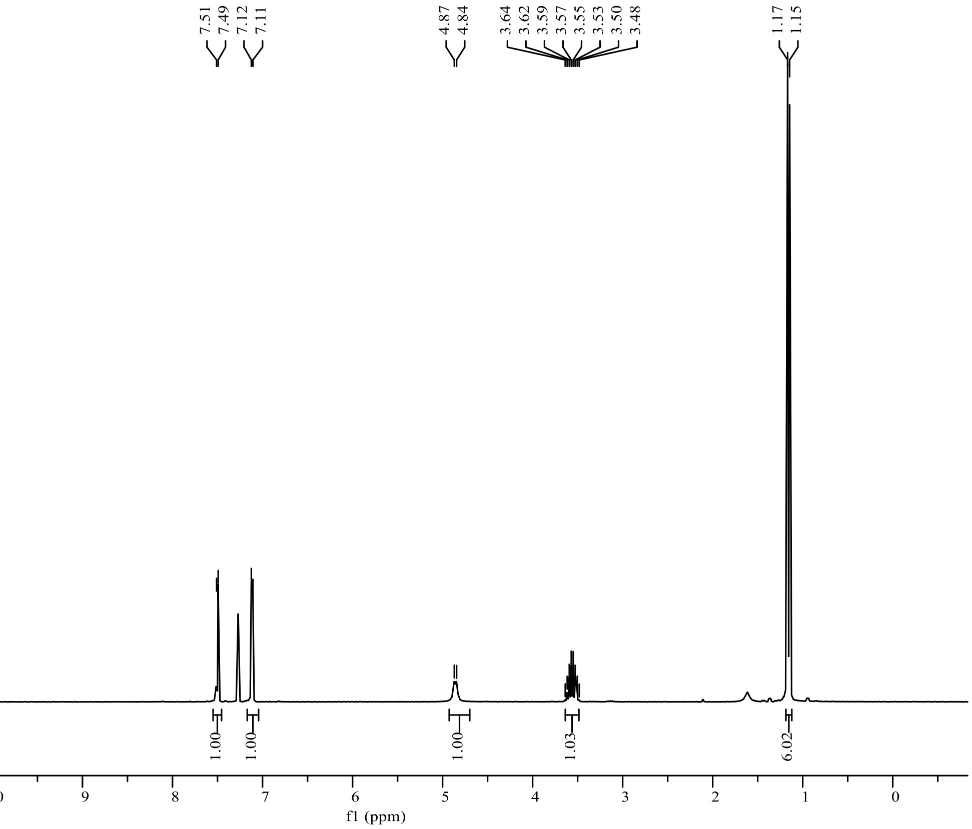

^{13}C NMR (75 MHz, $CDCl_3$):

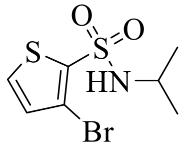

S-31

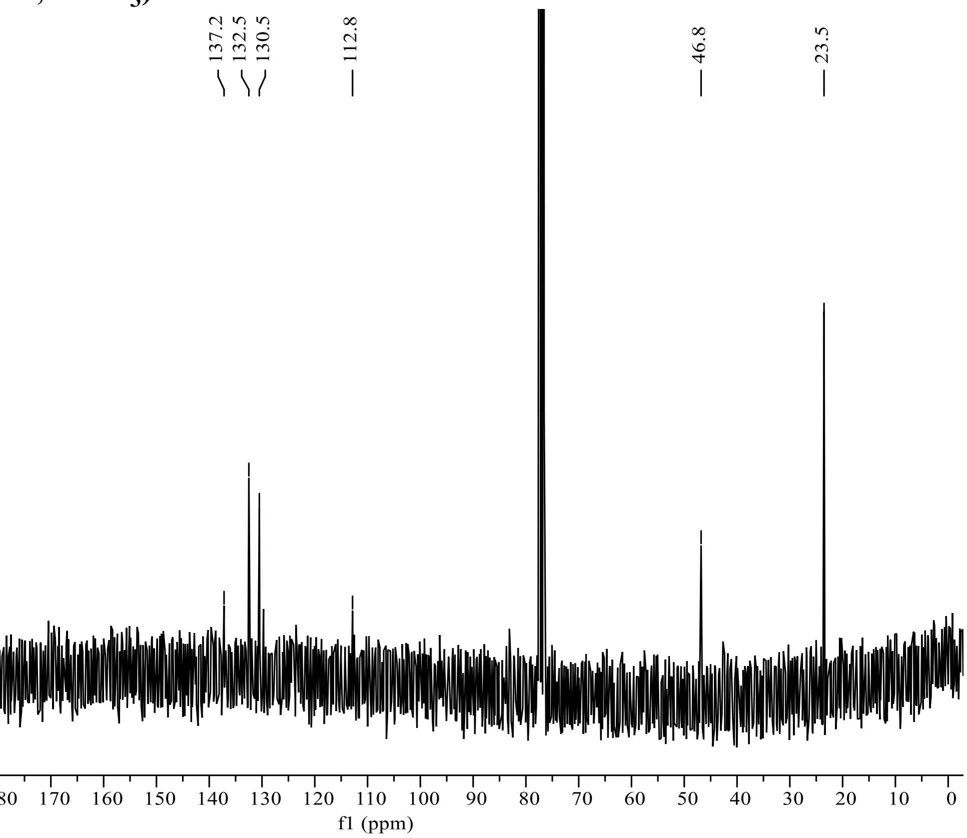

^{1}H NMR (600 MHz, $CDCl_3$):

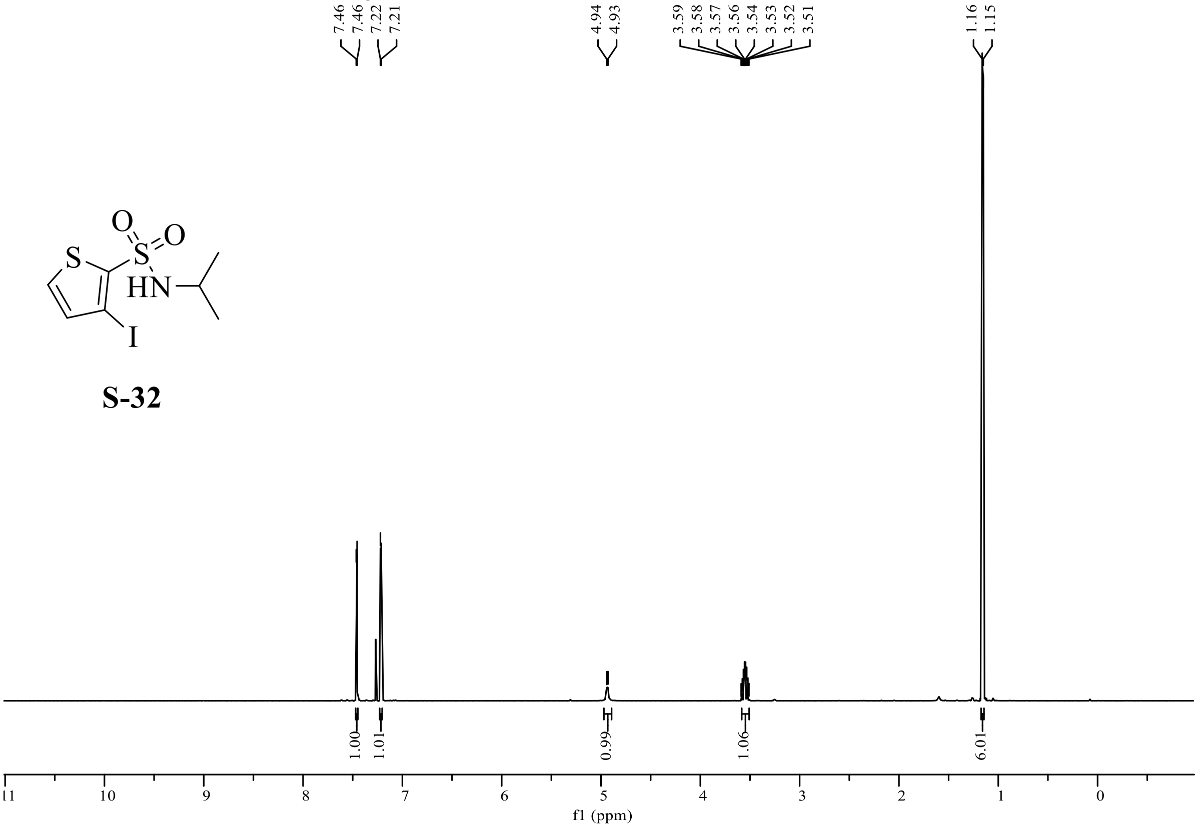

S-32

^{13}C NMR (151 MHz, $CDCl_3$):

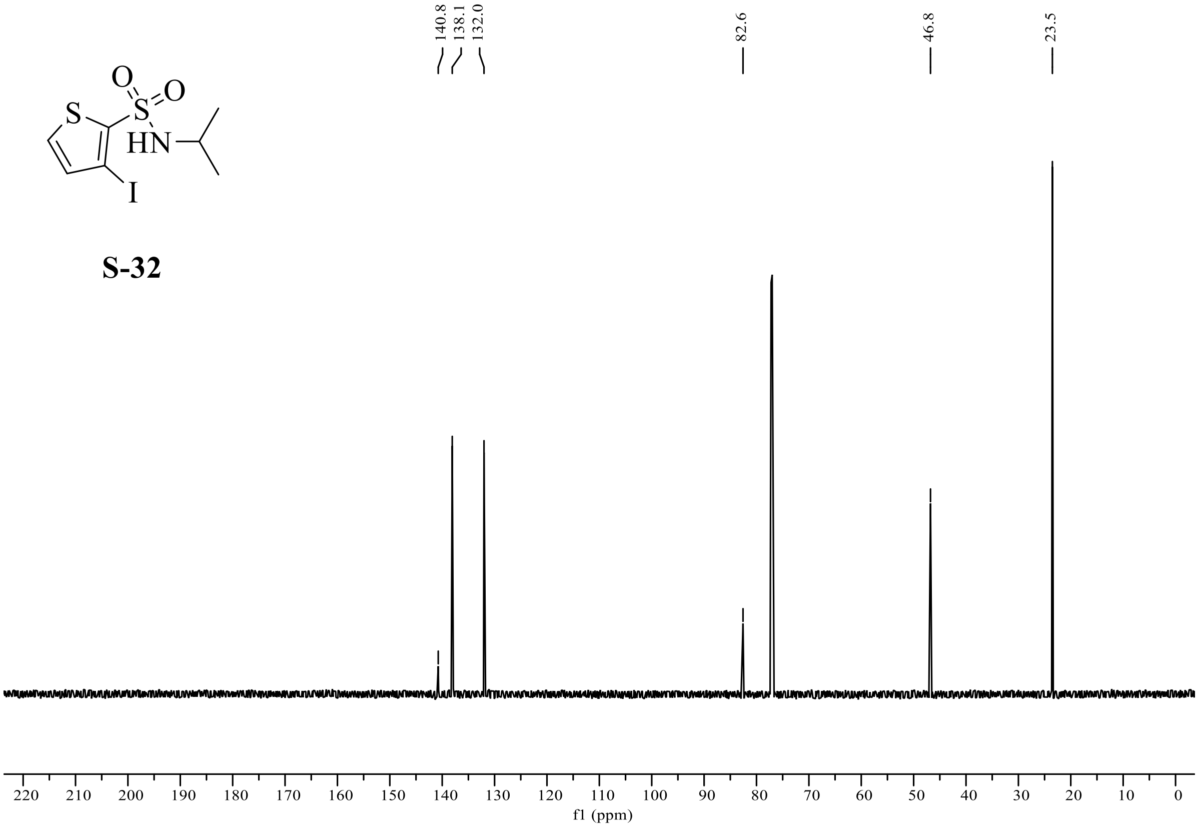

S-32

^{1}H NMR (300 MHz, $CDCl_3$):

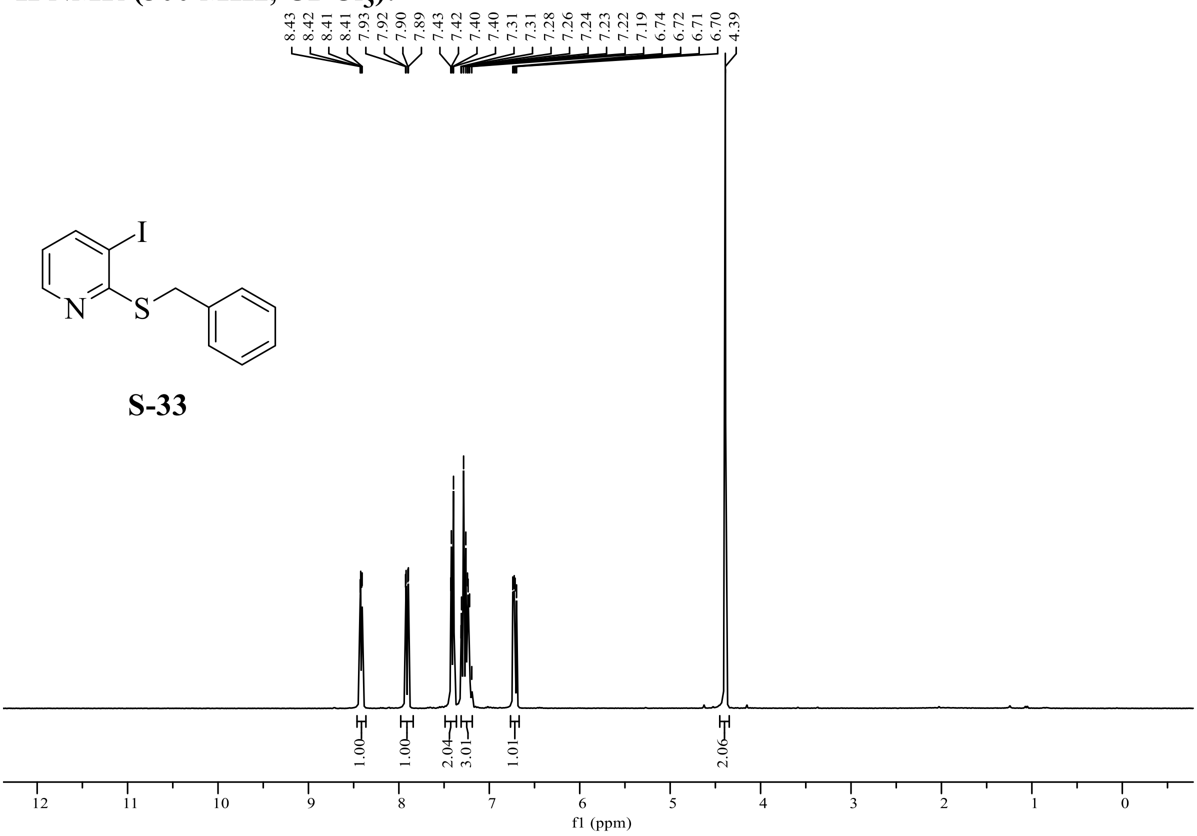

S-33

^{13}C NMR (75 MHz, $CDCl_3$):

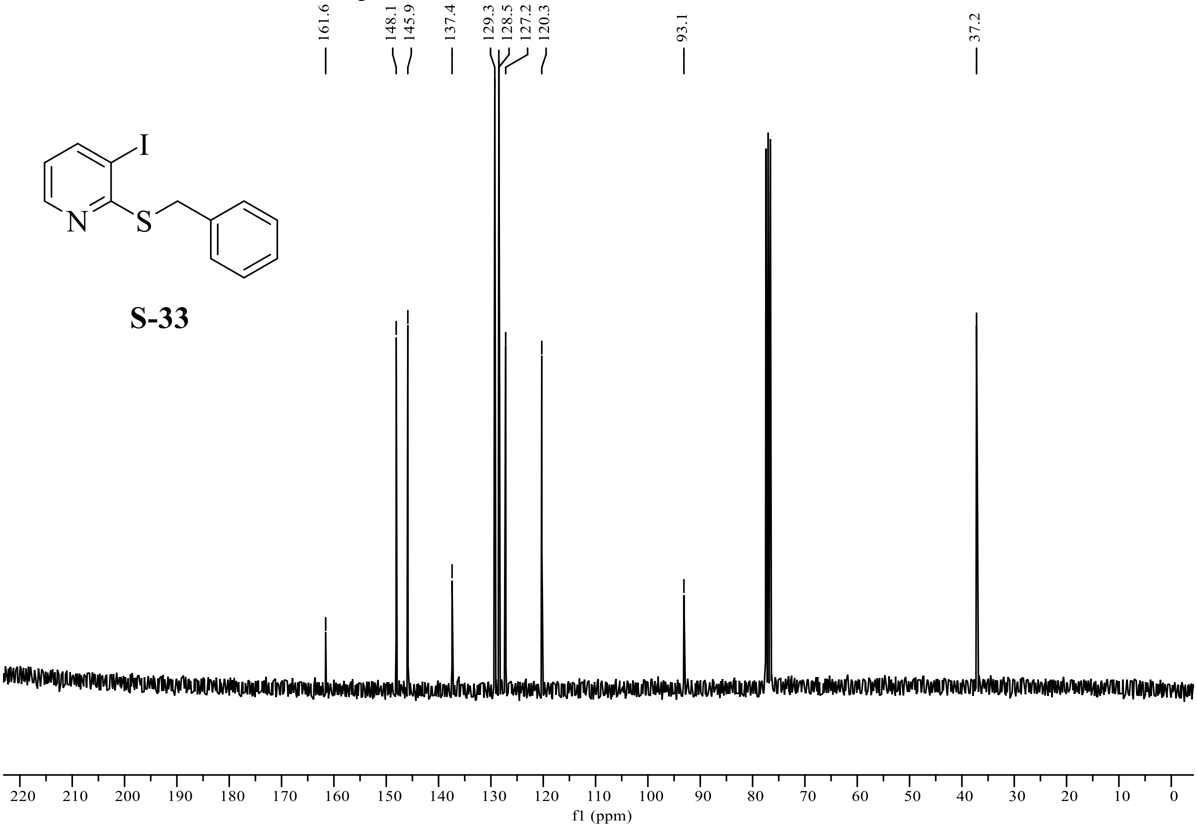

S-33

^{1}H NMR (600 MHz, $CDCl_3$):

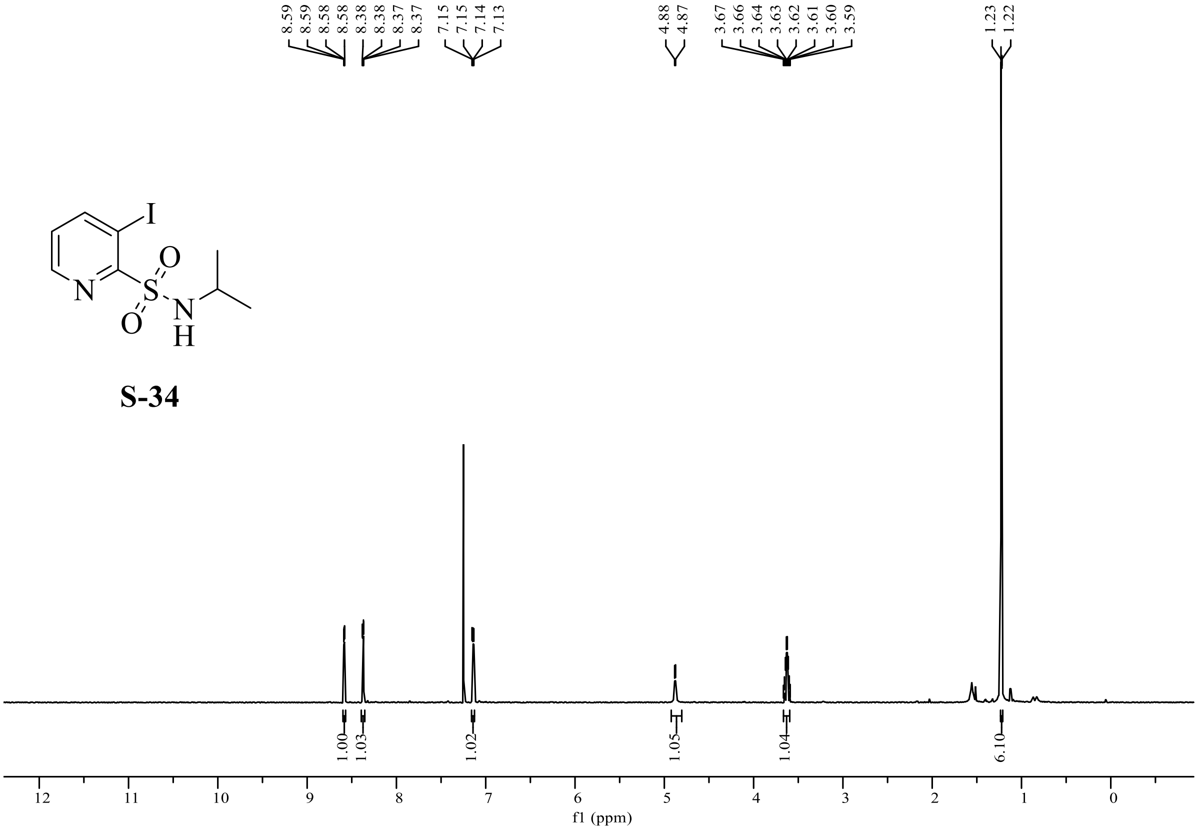

^{13}C NMR (151 MHz, $CDCl_3$):

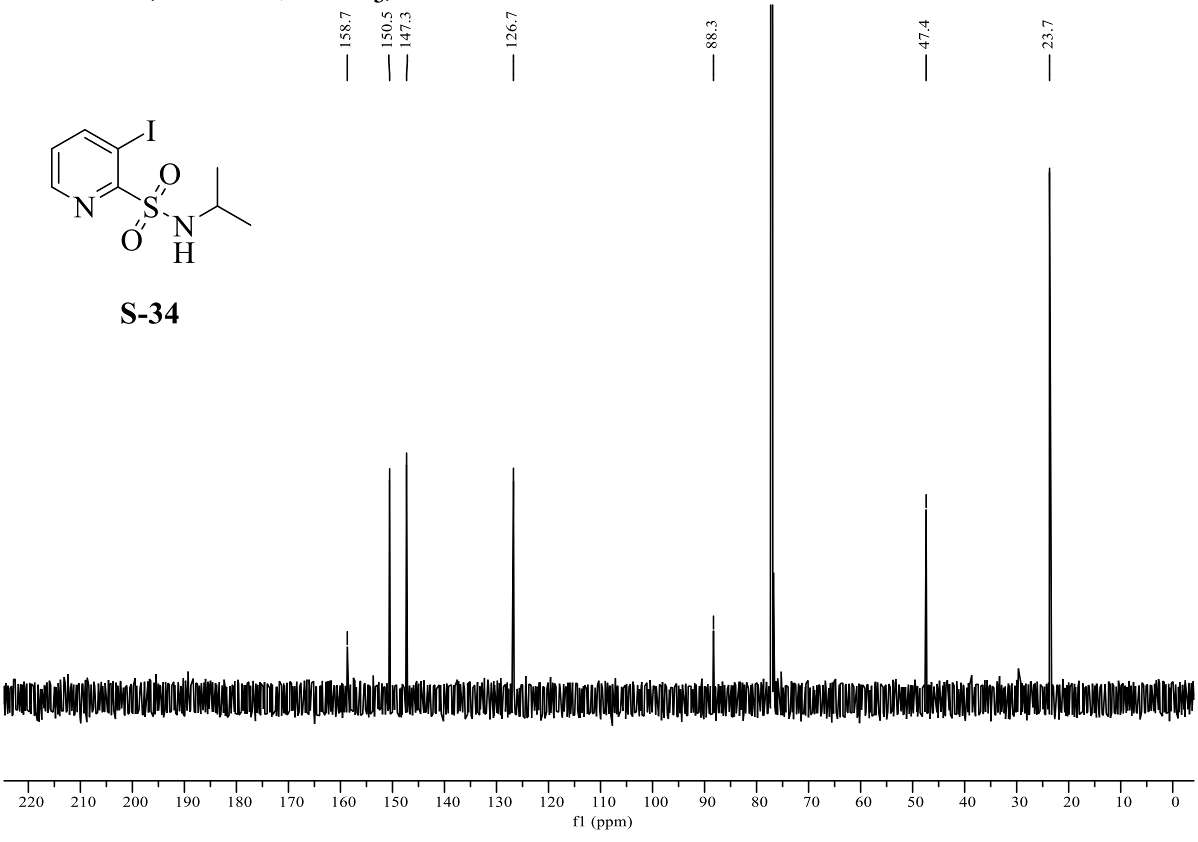

^{1}H NMR (300 MHz, $CDCl_3$):

S-35

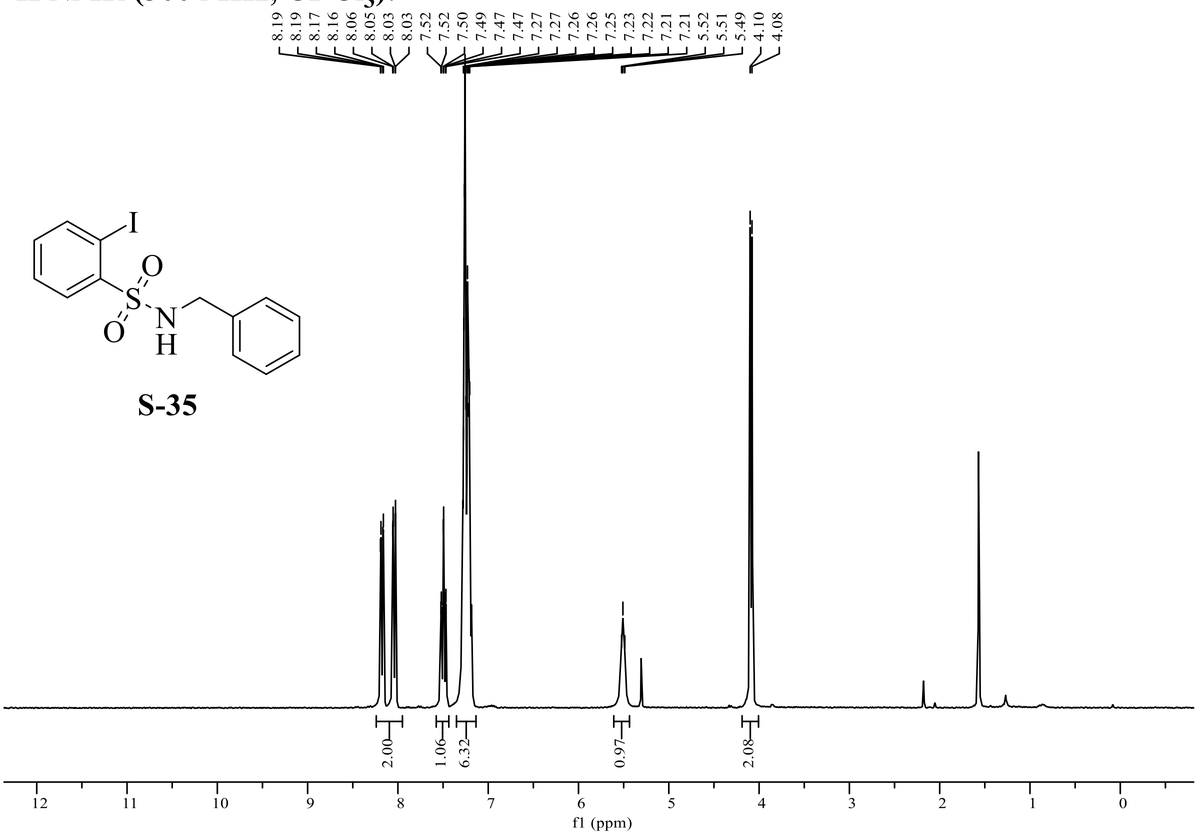

^{13}C NMR (75 MHz, $CDCl_3$):

S-35

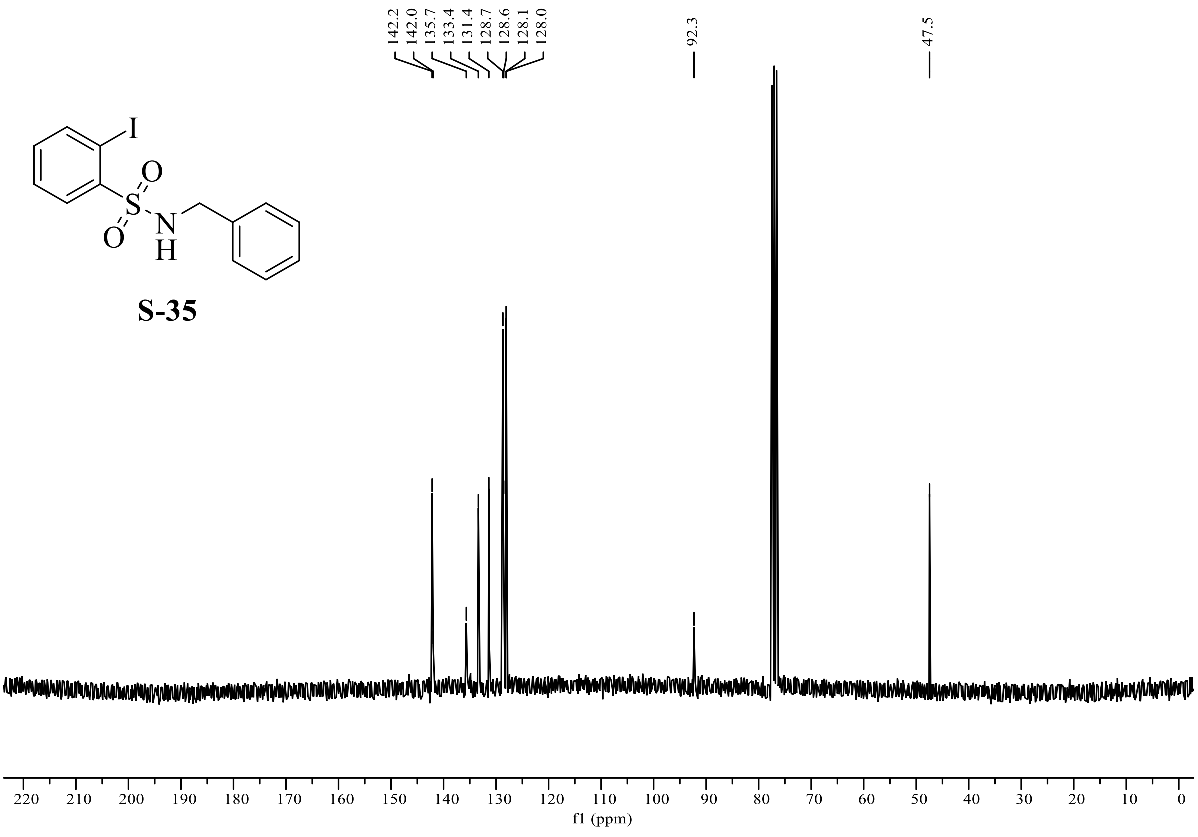

^{1}H NMR (300 MHz, $CDCl_3$):

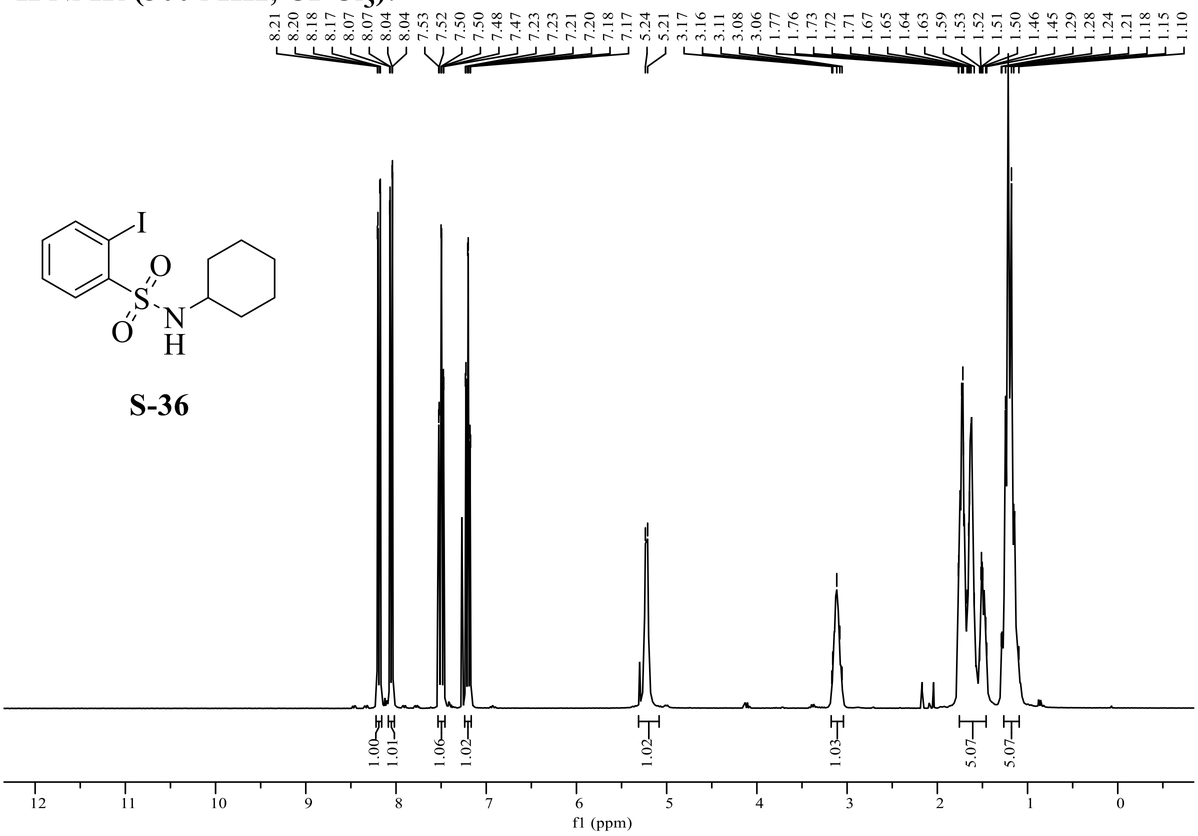

S-36

^{13}C NMR (75 MHz, $CDCl_3$):

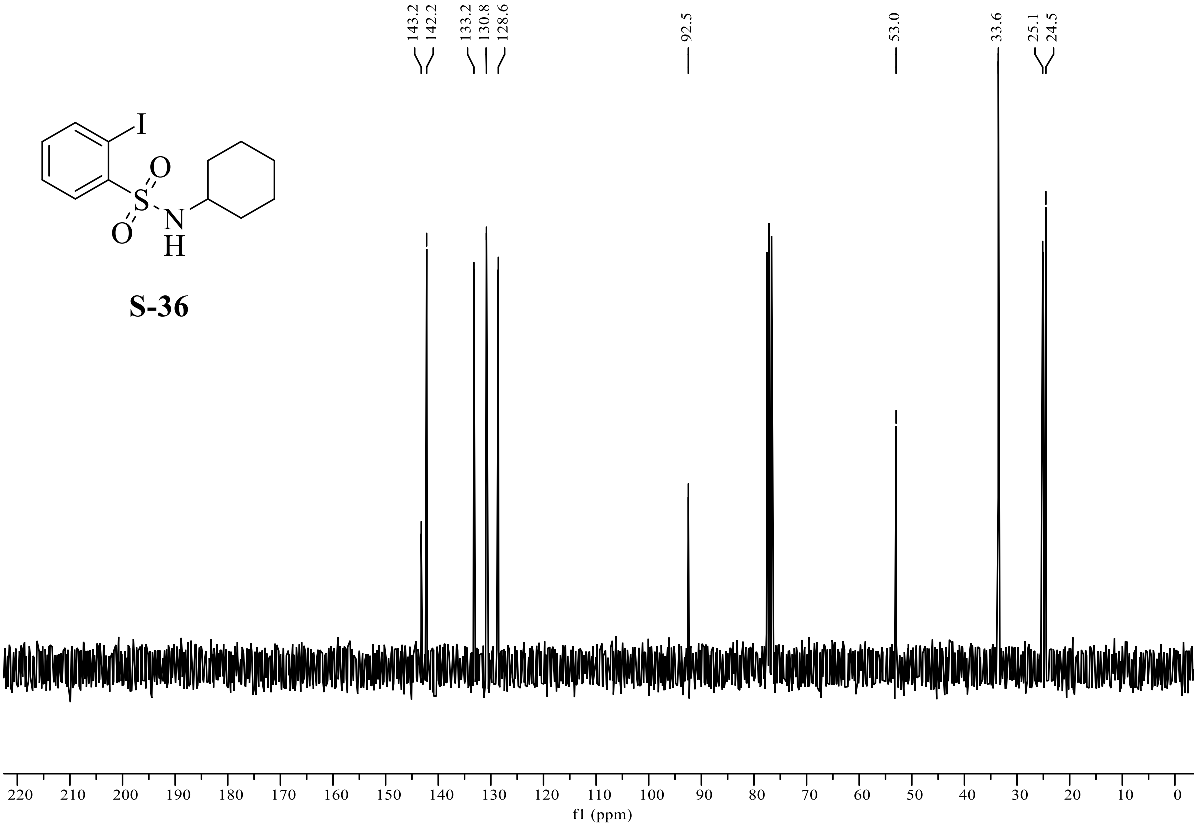

S-36

^{1}H NMR (300 MHz, $CDCl_3$):

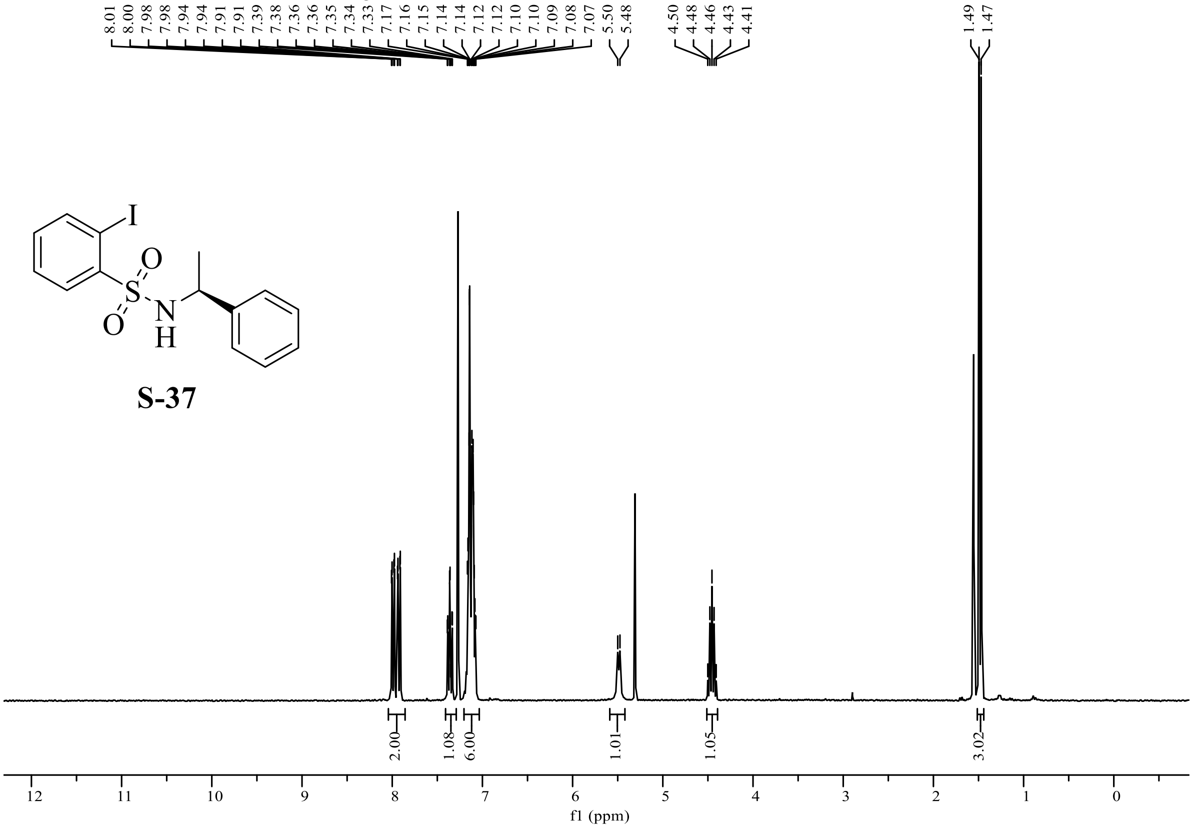

^{13}C NMR (75 MHz, $CDCl_3$):

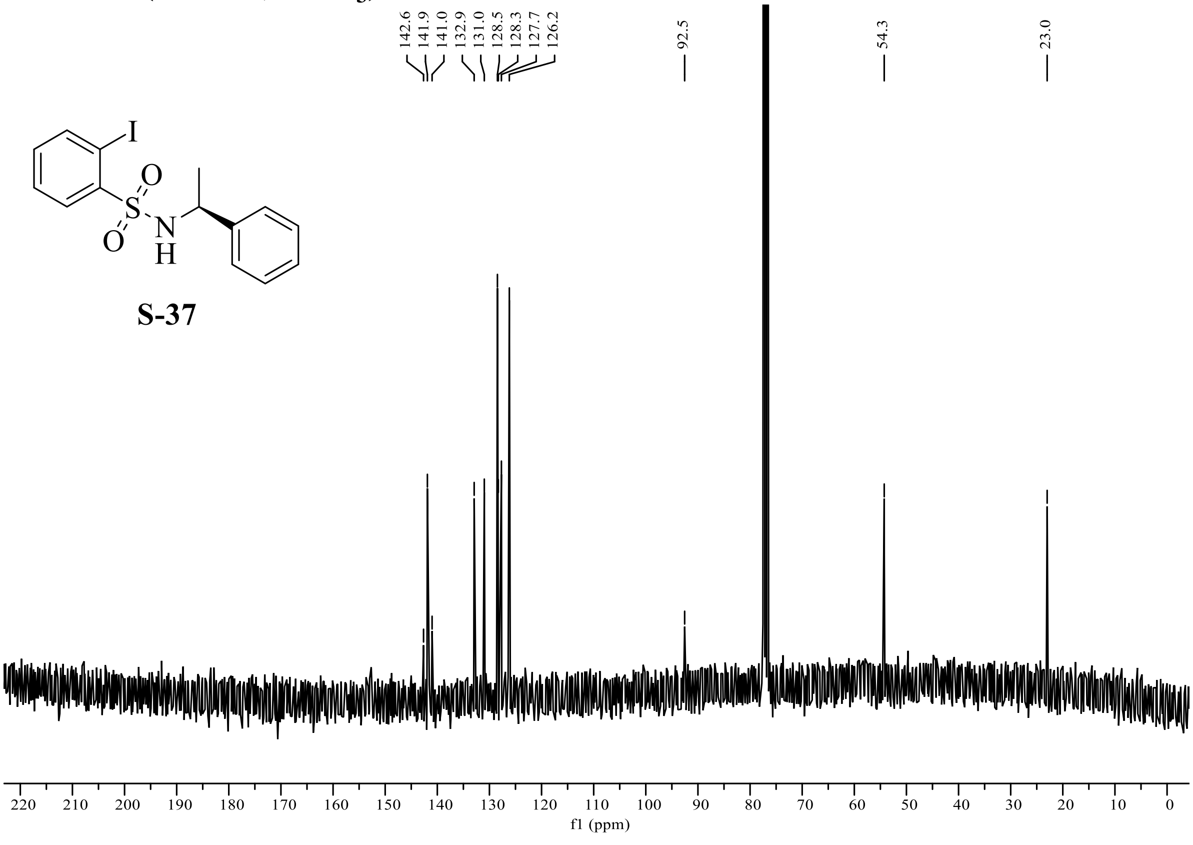

^{1}H NMR (300 MHz, $CDCl_3$):

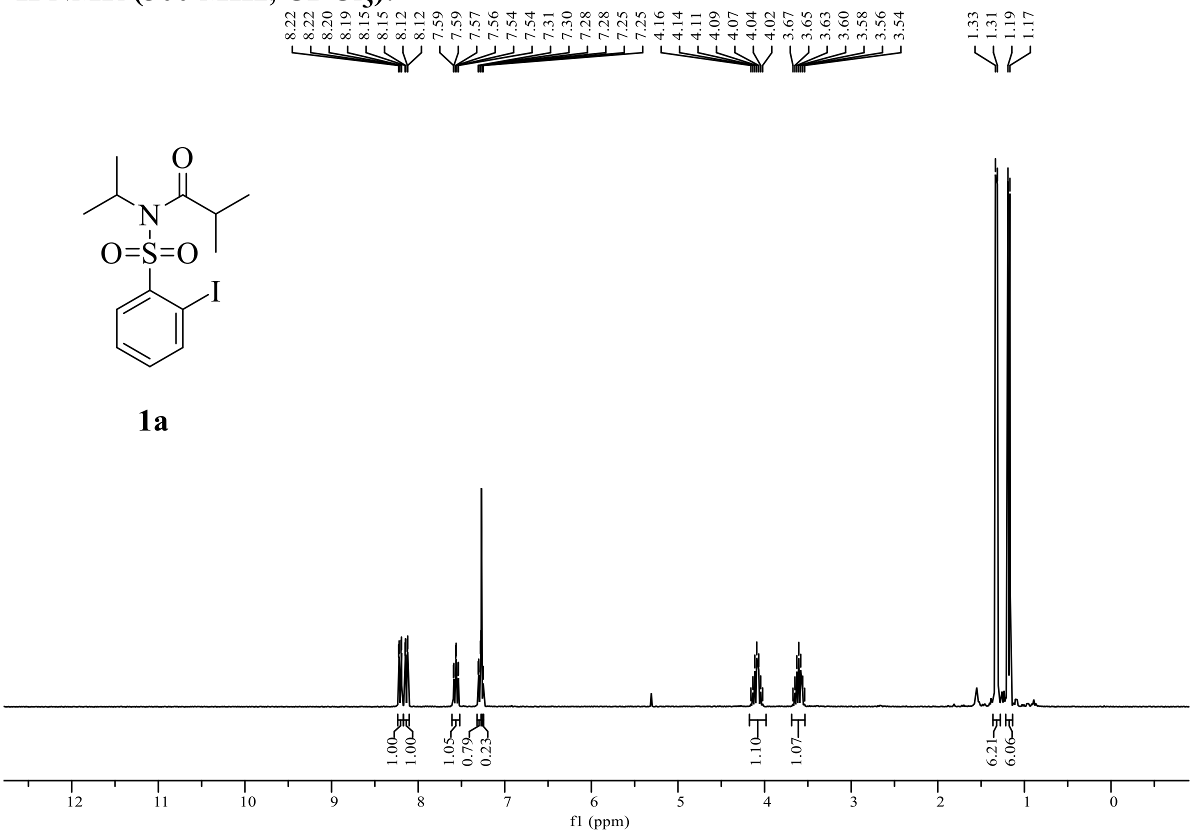

1a

^{13}C NMR (75 MHz, $CDCl_3$):

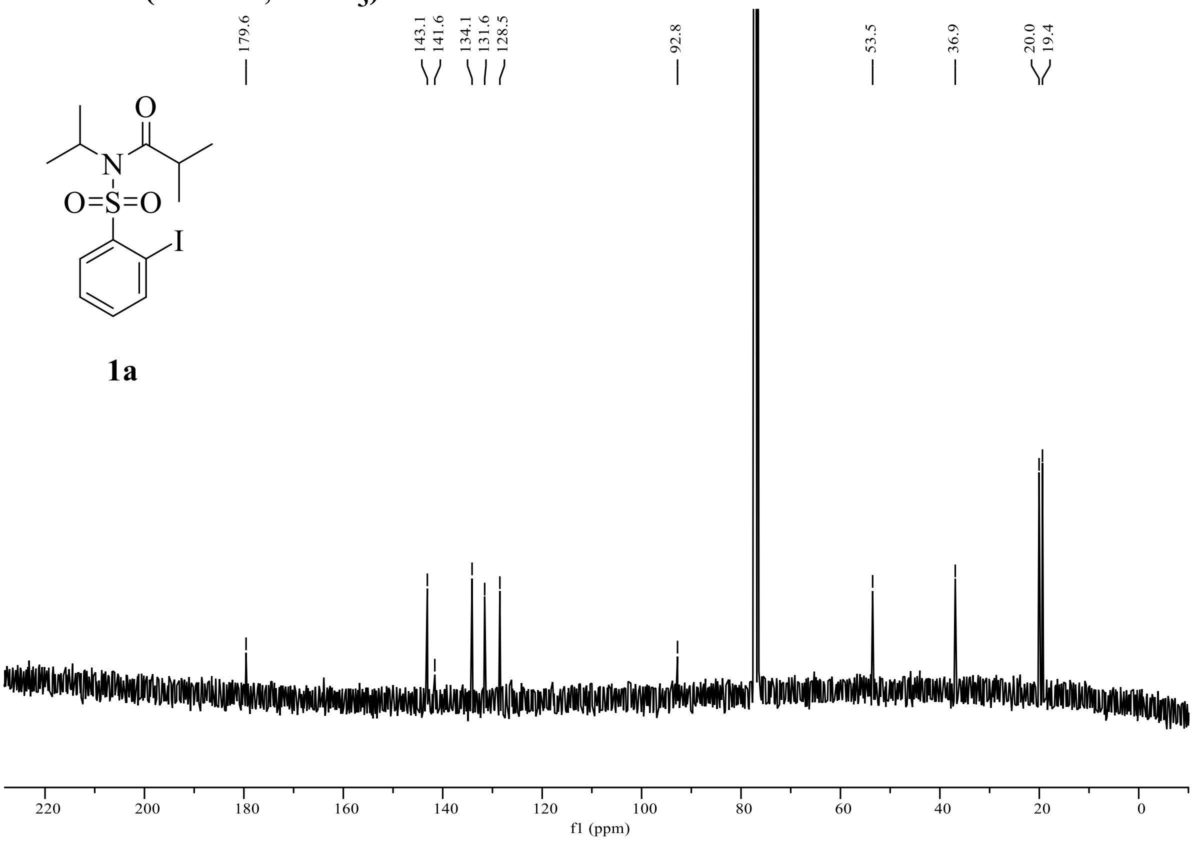

1a

^{1}H NMR (300 MHz, $CDCl_3$):

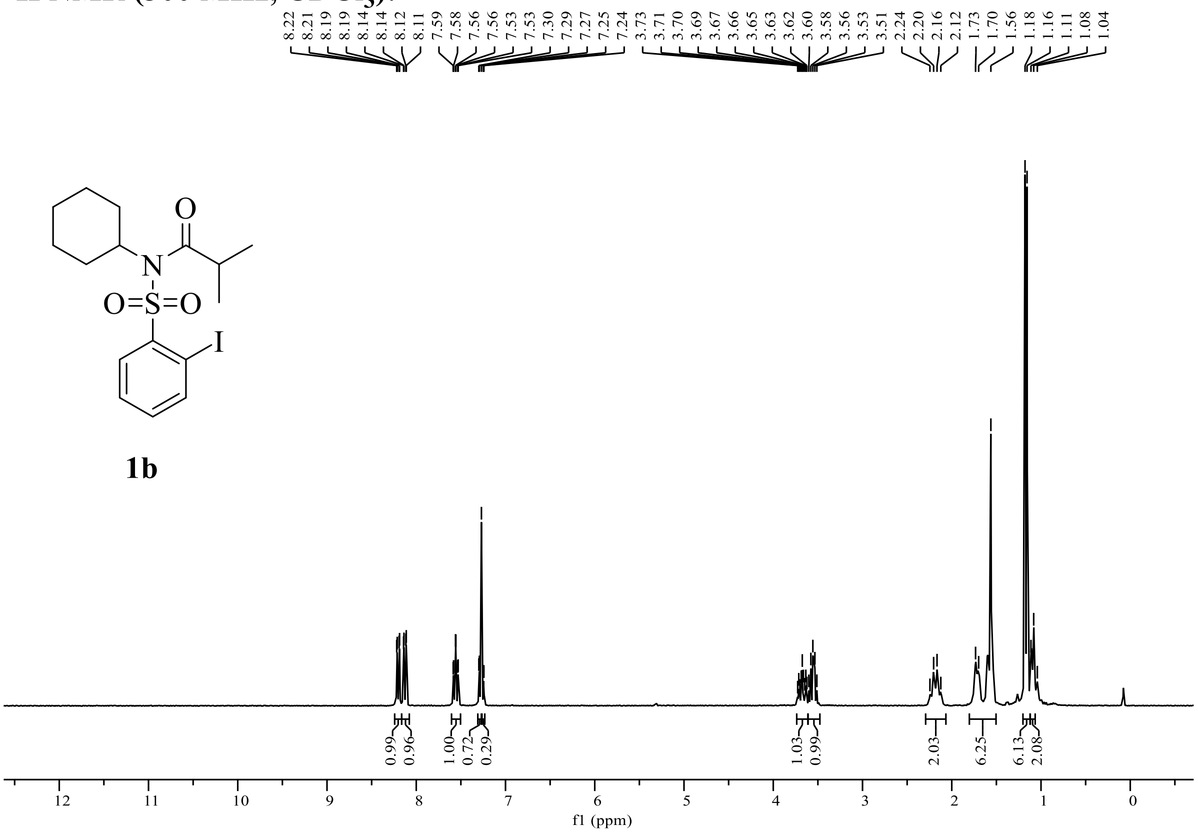

^{13}C NMR (75 MHz, $CDCl_3$):

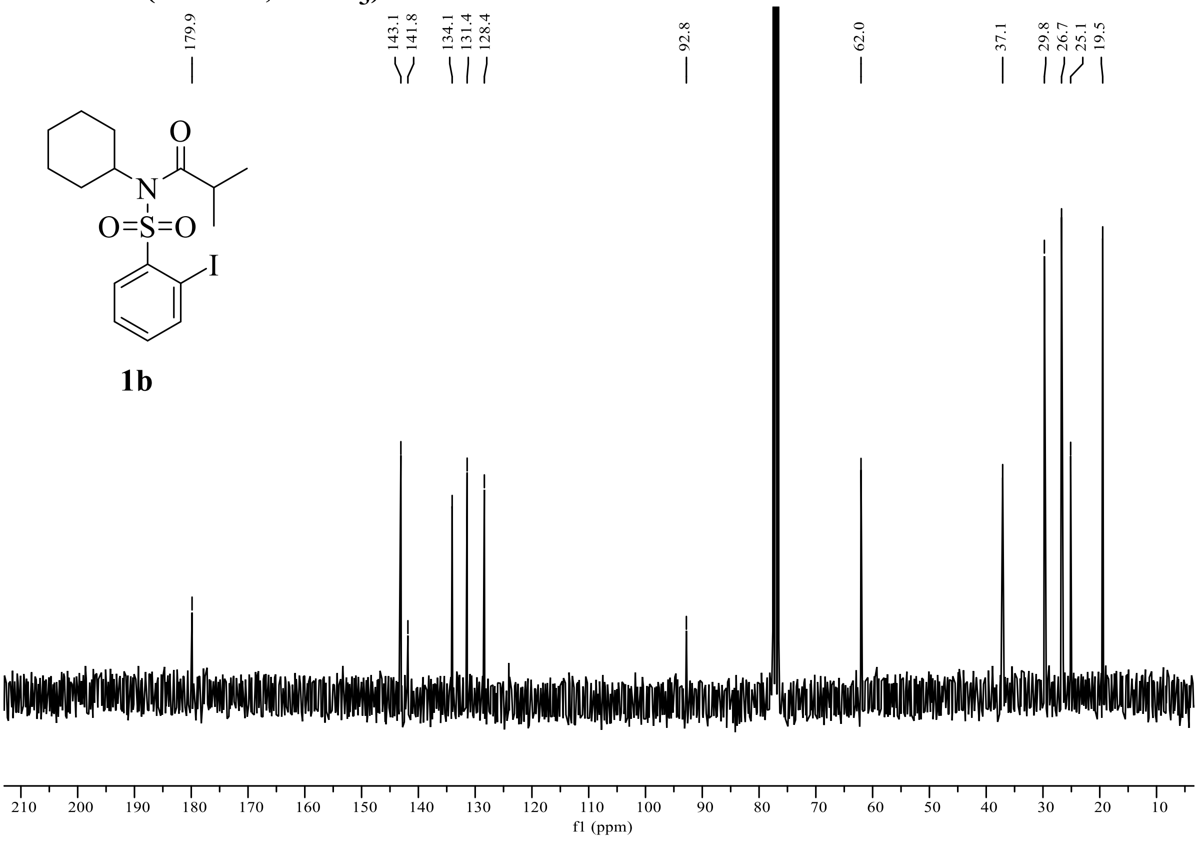

^{1}H NMR (300 MHz, $CDCl_3$):

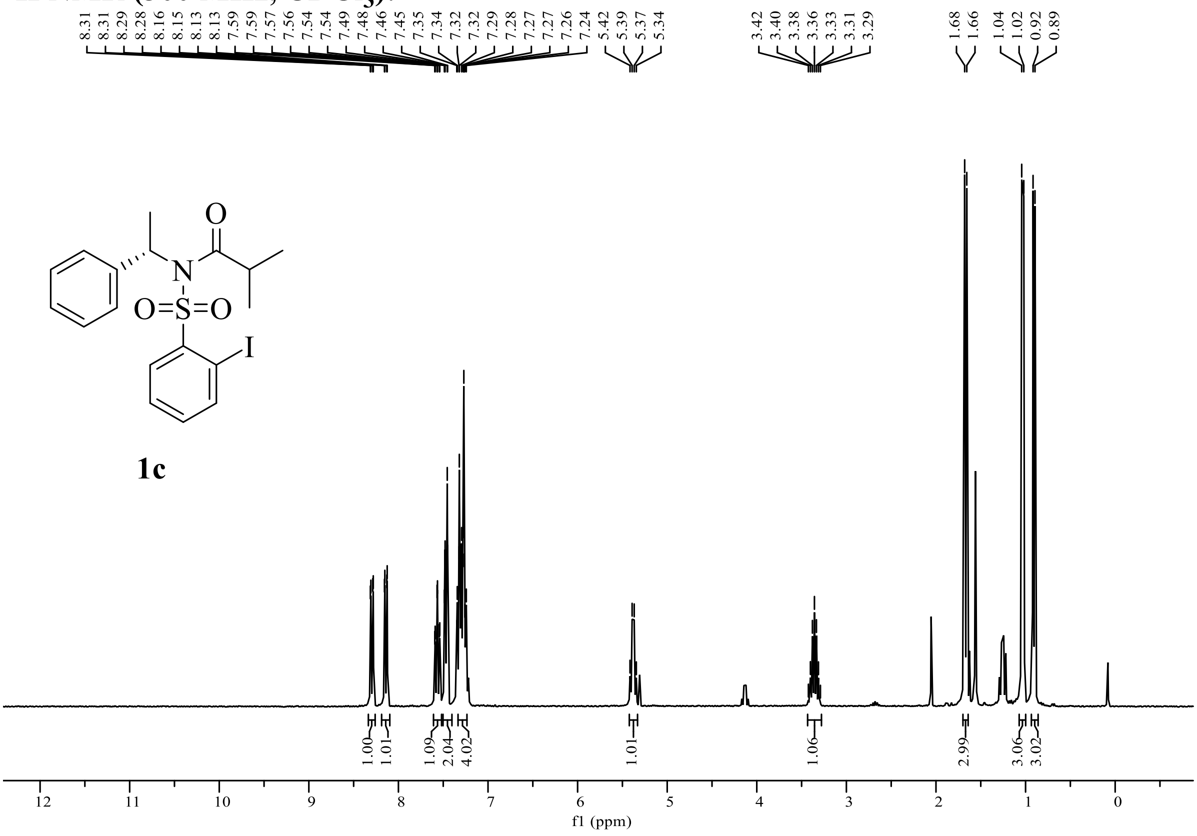

1c

^{13}C NMR (75 MHz, $CDCl_3$):

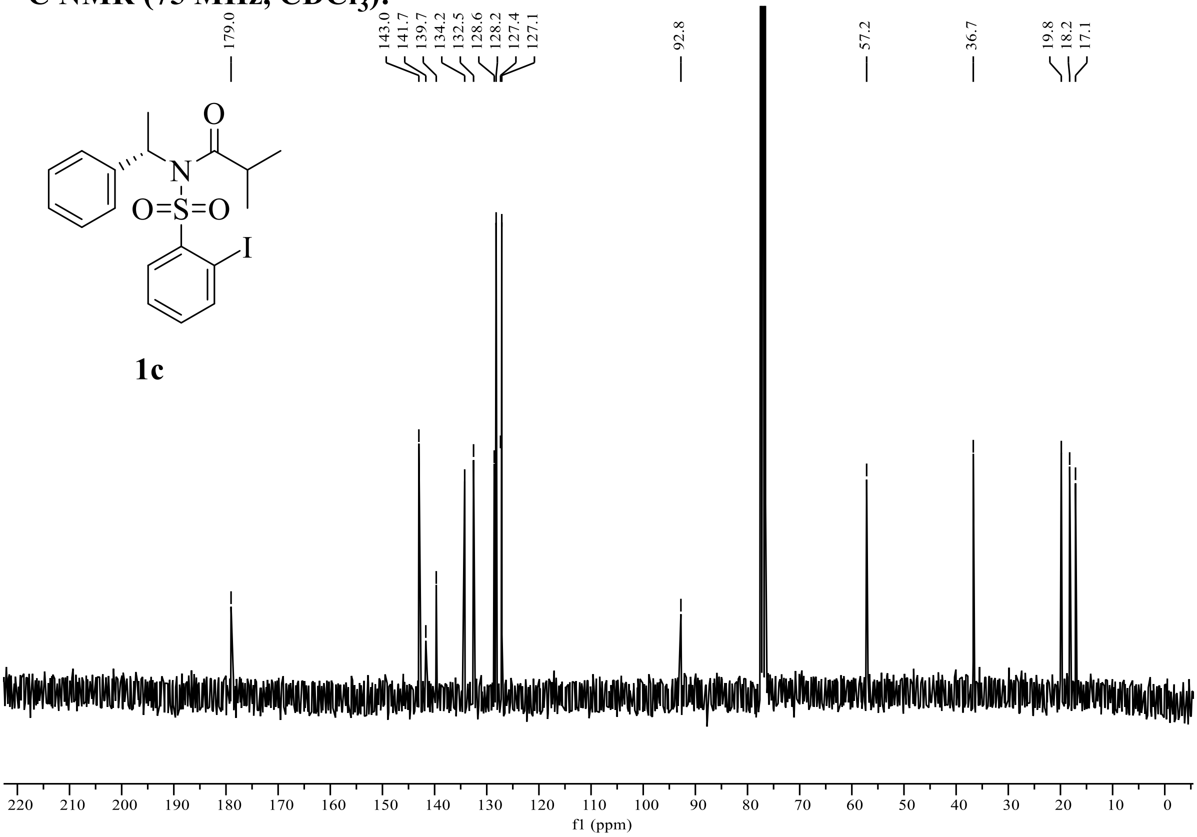

1c

^{1}H NMR (300 MHz, $CDCl_3$):

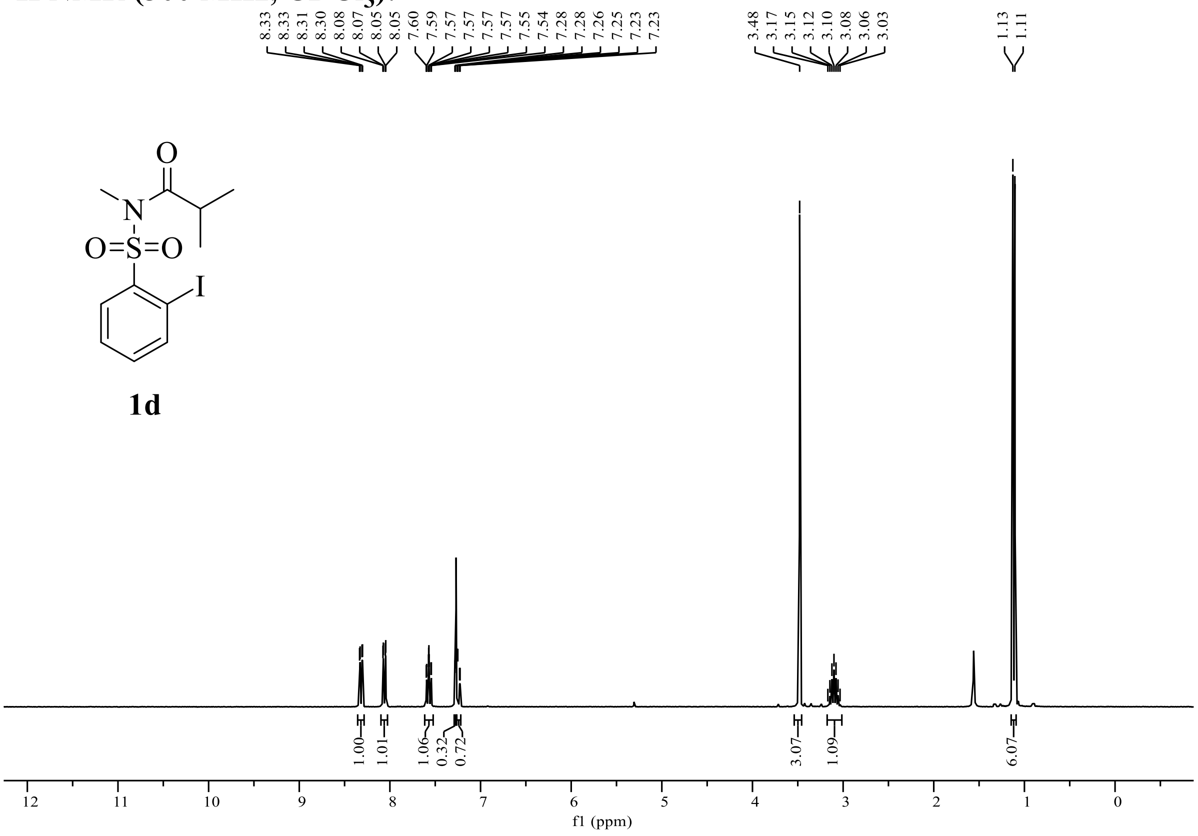

^{13}C NMR (75 MHz, $CDCl_3$):

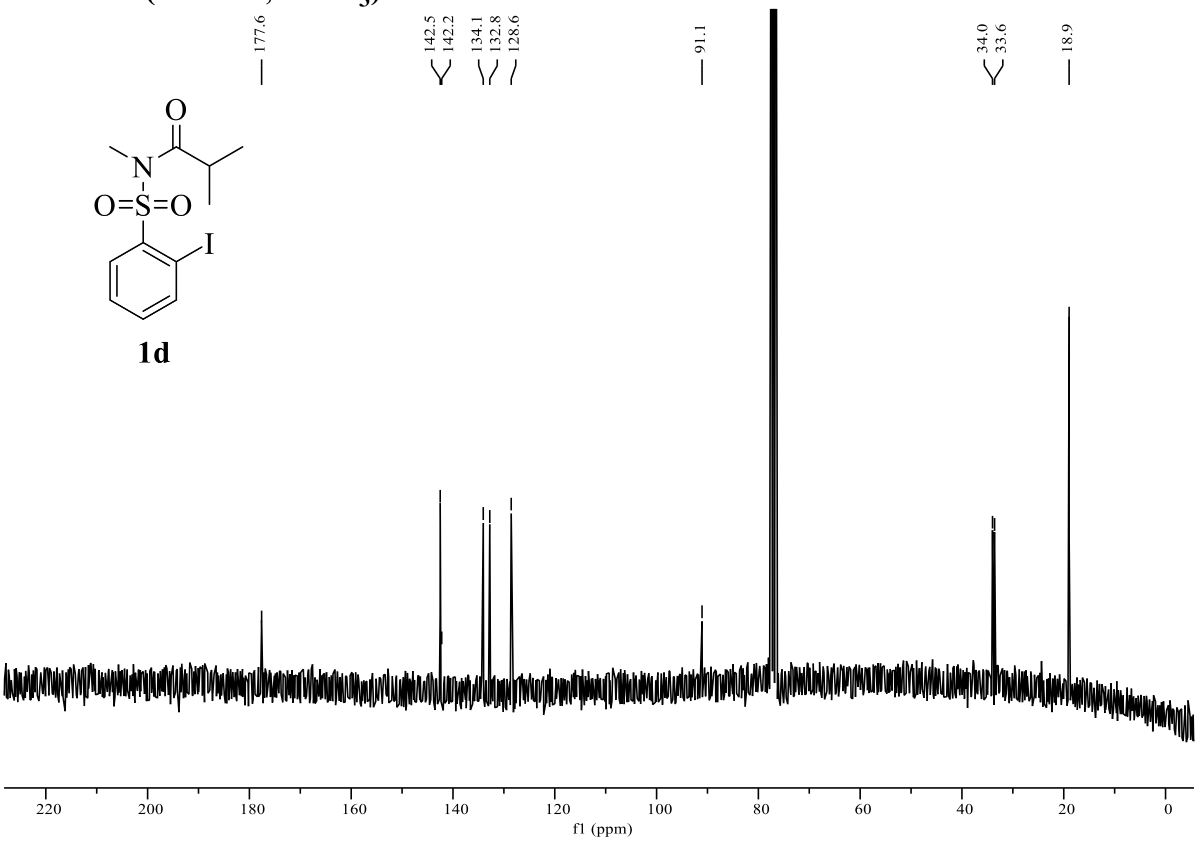

^{1}H NMR (300 MHz, $CDCl_3$):

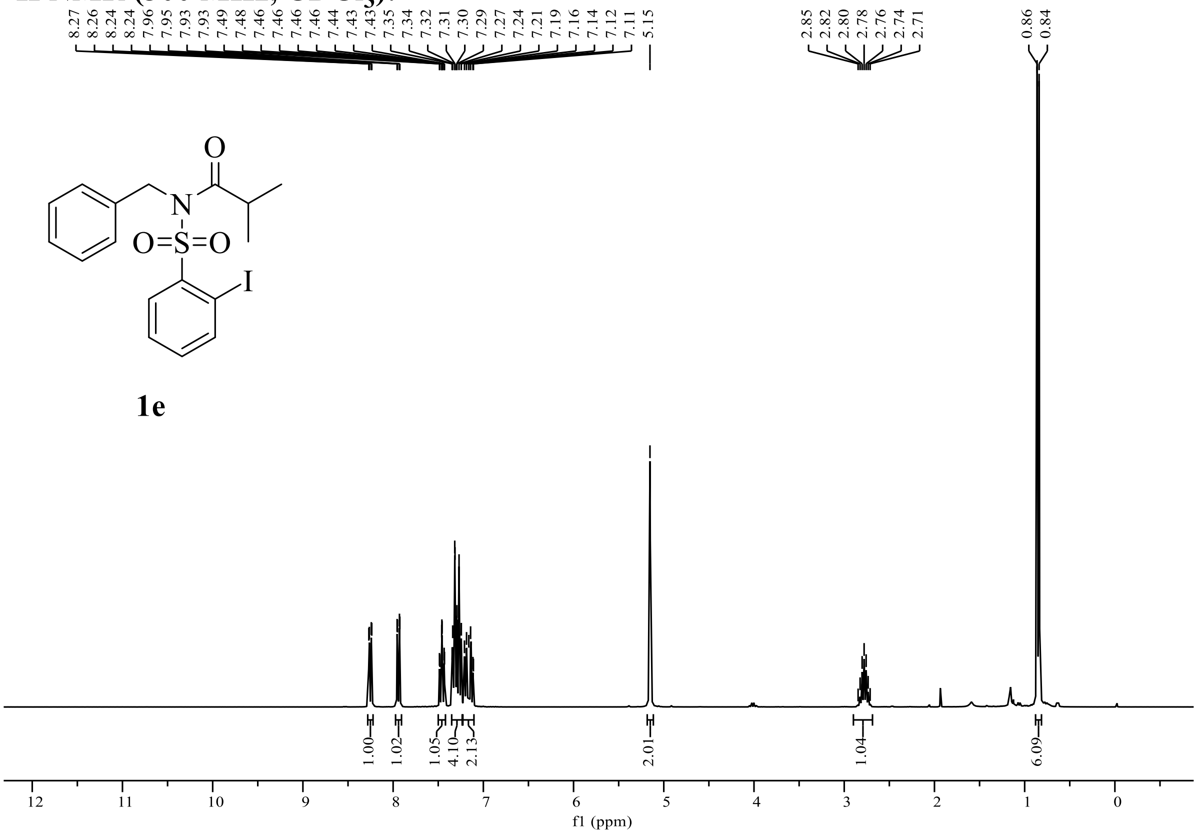

1e

^{13}C NMR (75 MHz, $CDCl_3$):

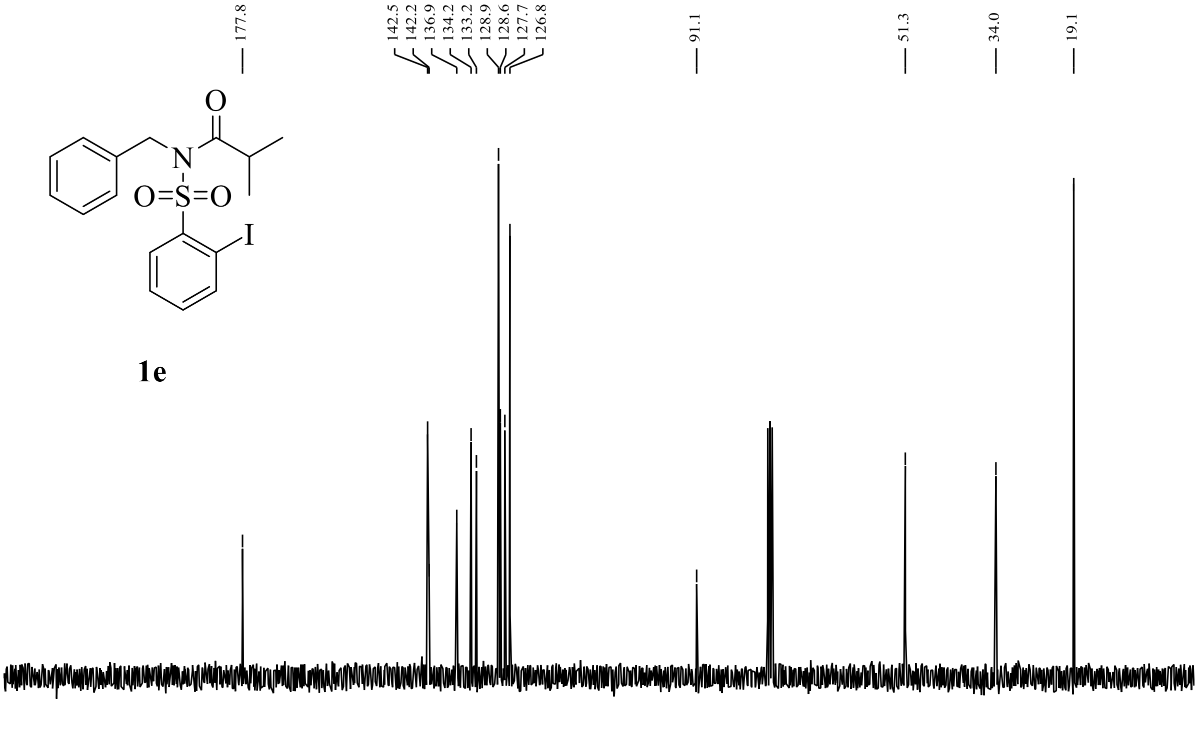

1e

^{1}H NMR (300 MHz, $CDCl_3$):

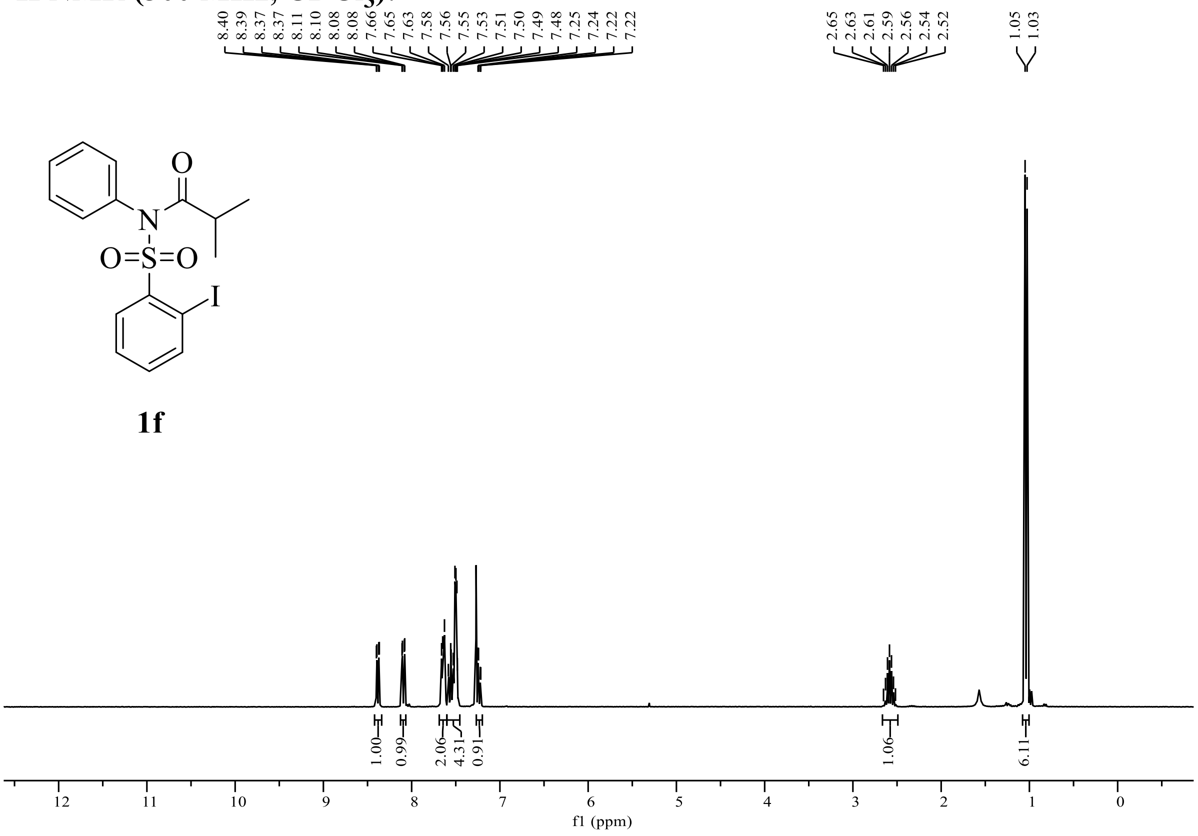

^{13}C NMR (75 MHz, $CDCl_3$):

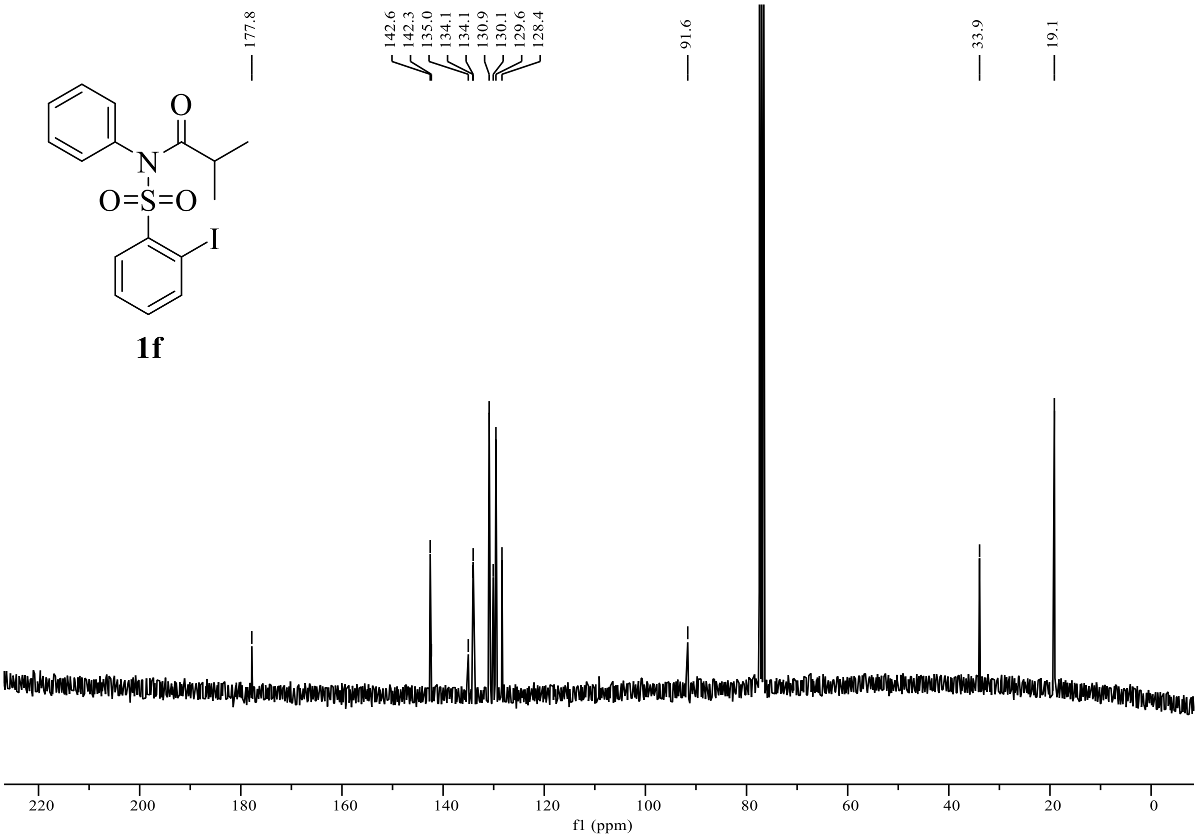

^{1}H NMR (300 MHz, $CDCl_3$):

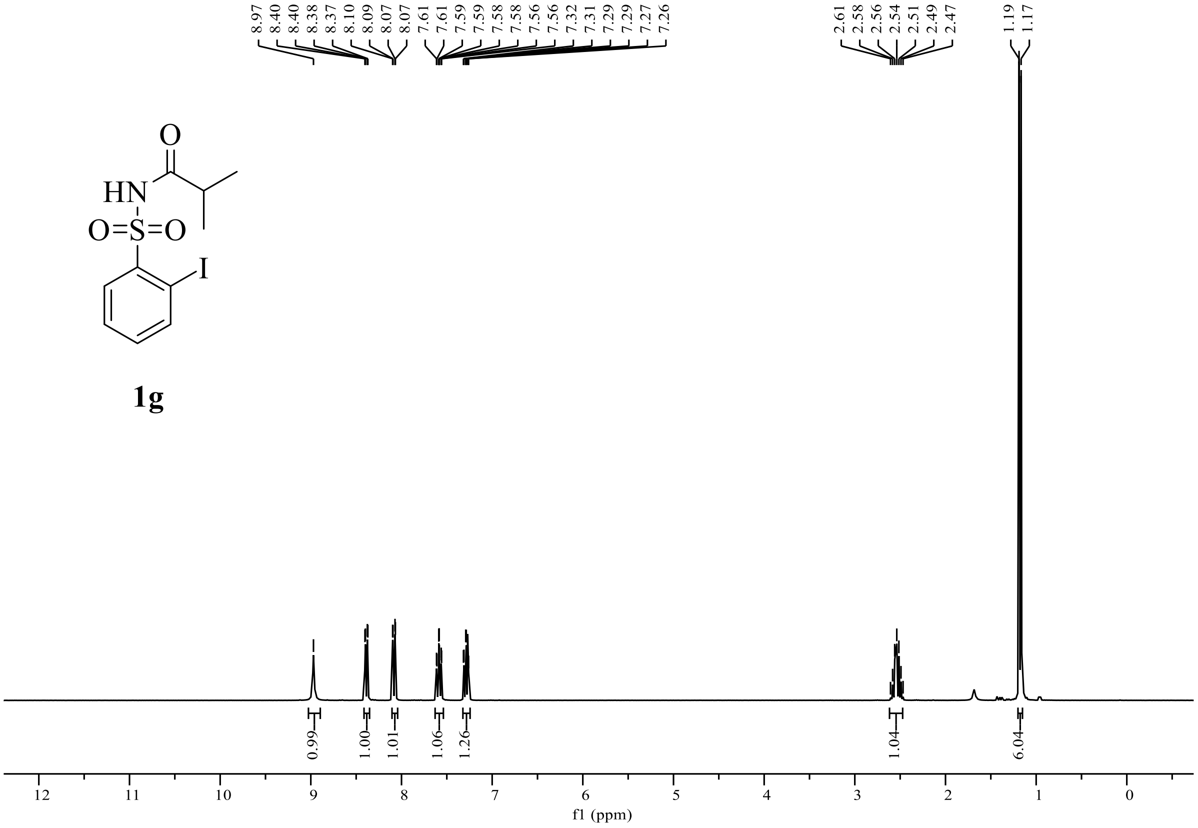

1g

^{13}C NMR (75 MHz, $CDCl_3$):

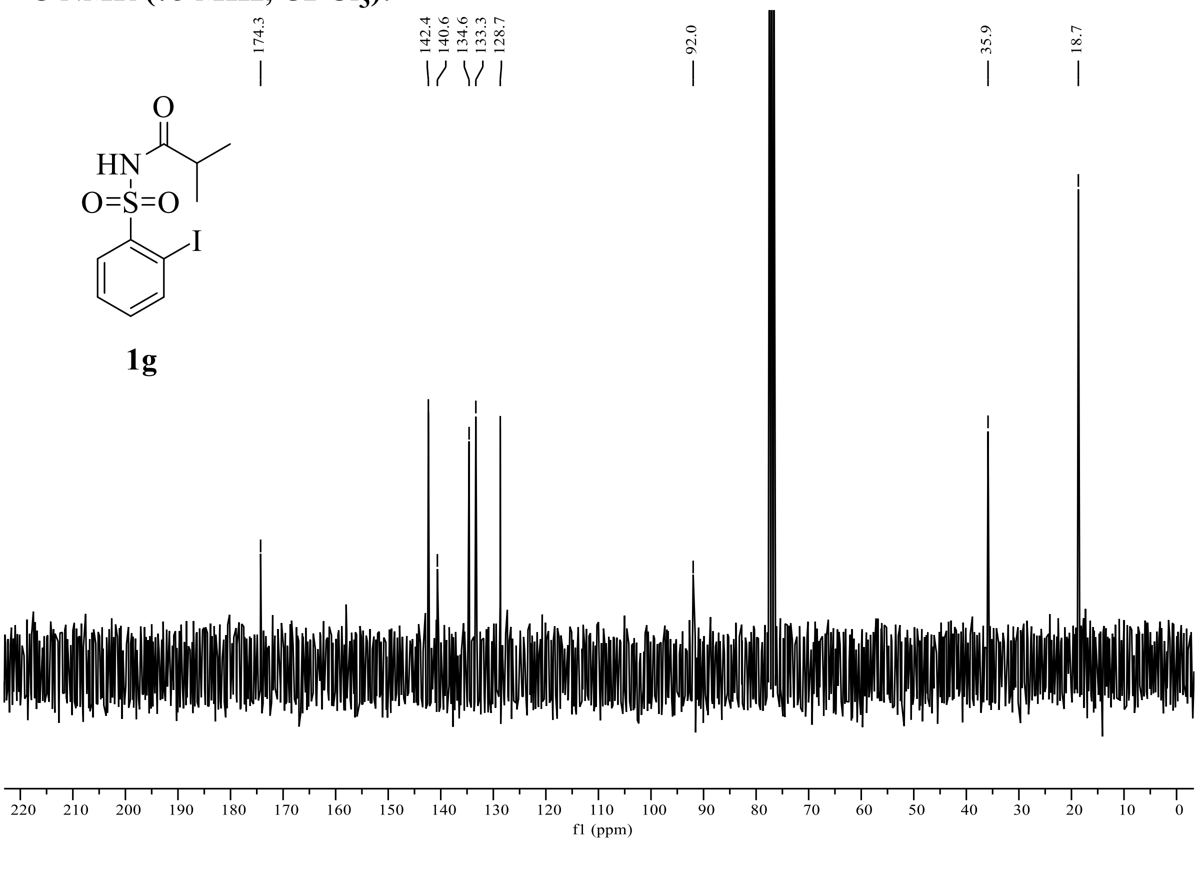

1g

^{1}H NMR (300 MHz, $CDCl_3$):

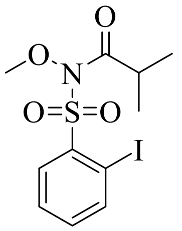

1h

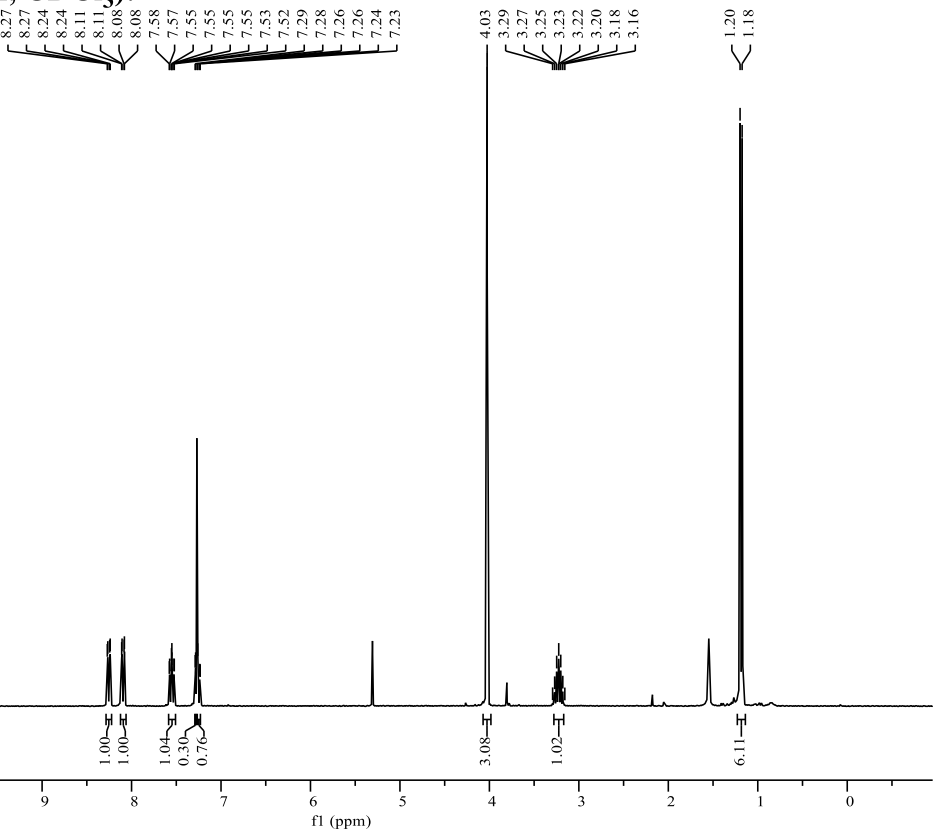

^{13}C NMR (75 MHz, $CDCl_3$):

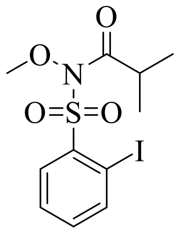

1h

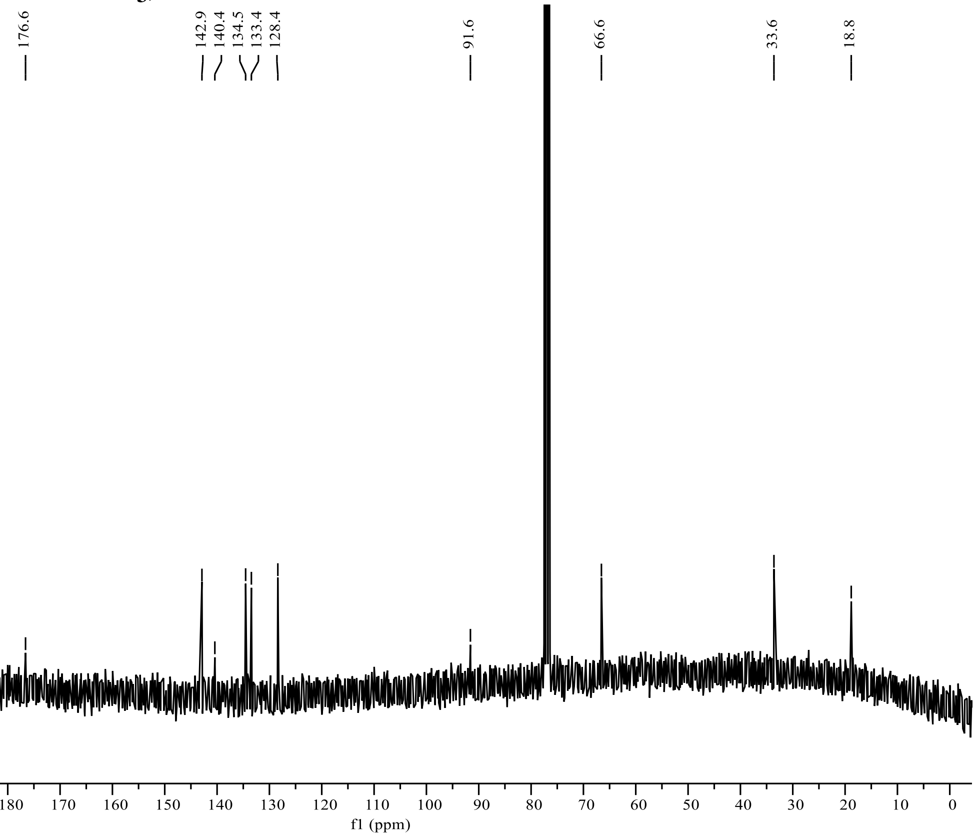

^{1}H NMR (300 MHz, $CDCl_3$):

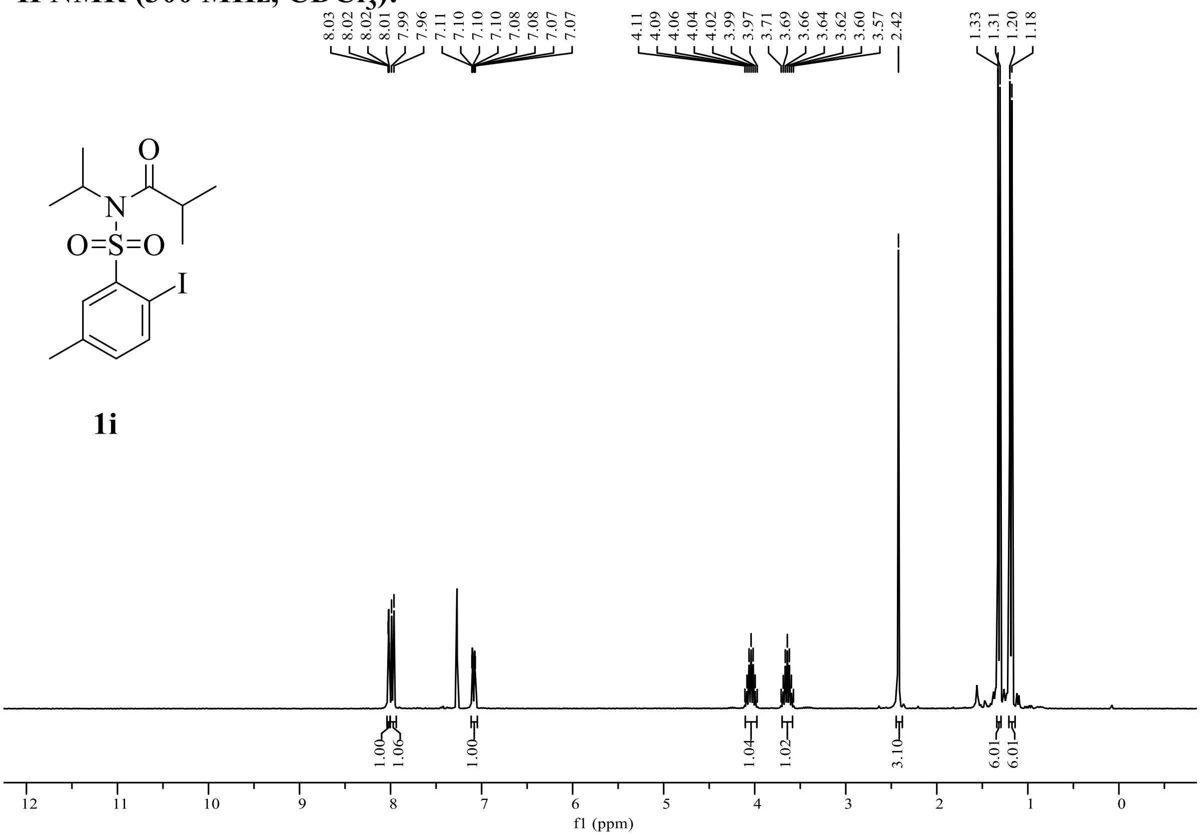

1i

^{13}C NMR (75 MHz, $CDCl_3$):

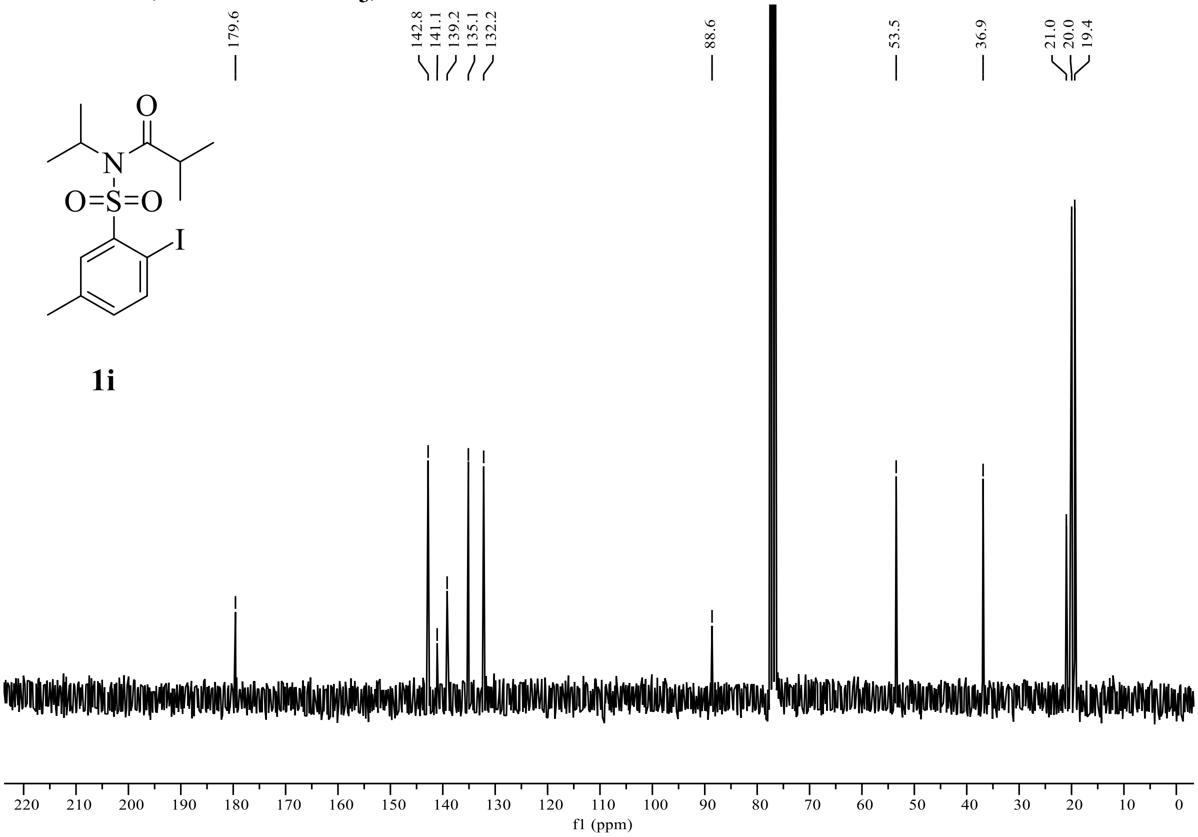

1i

^{1}H NMR (300 MHz, $CDCl_3$):

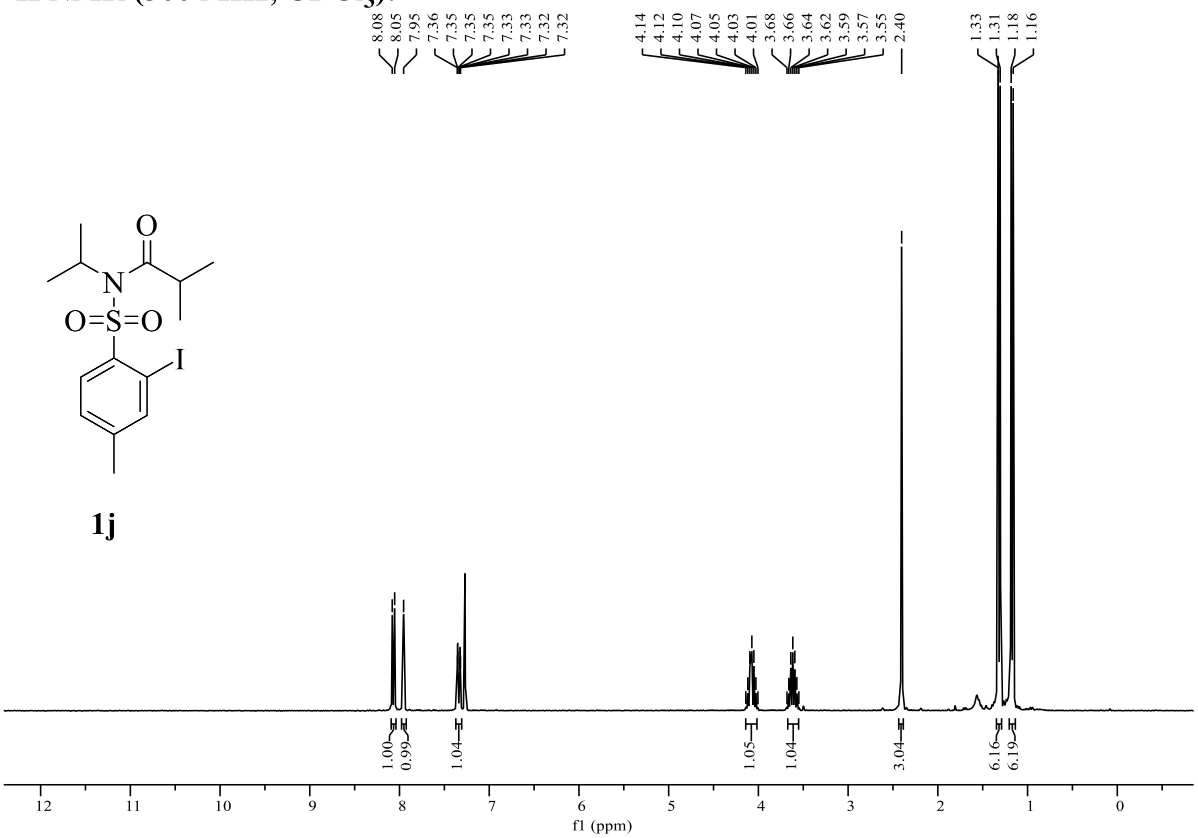

^{13}C NMR (75 MHz, $CDCl_3$):

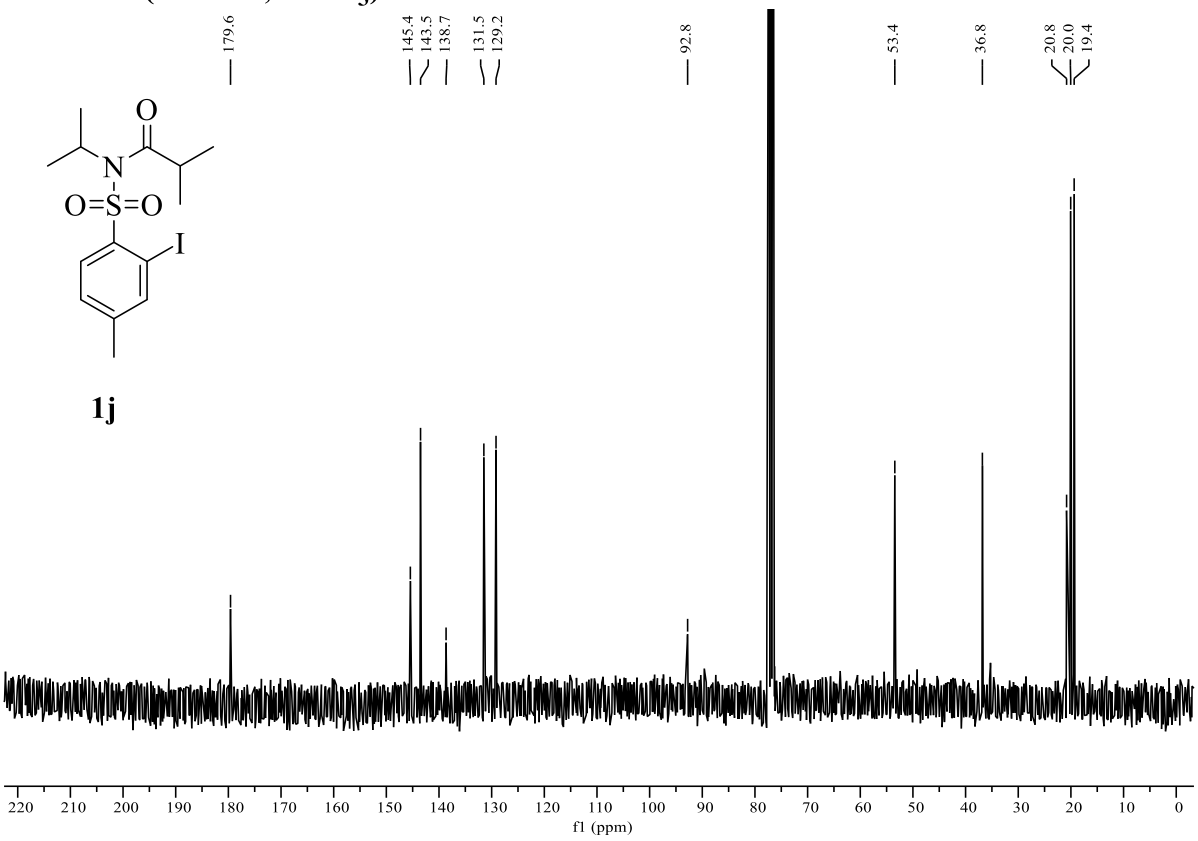

^{1}H NMR (300 MHz, $CDCl_3$):

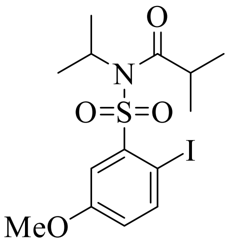

1k

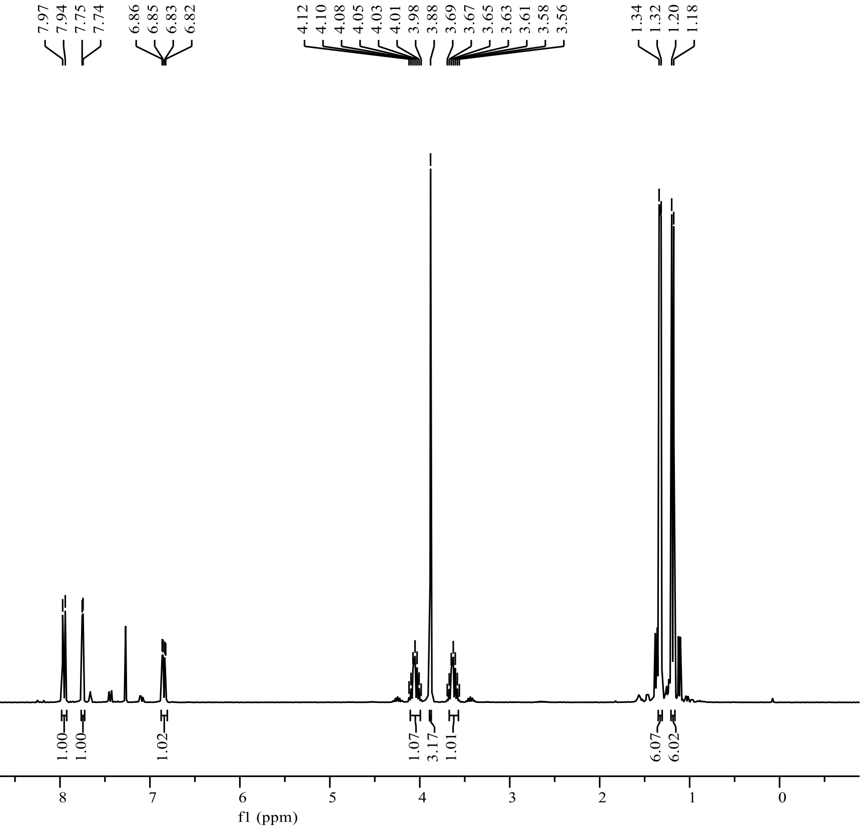

^{13}C NMR (75 MHz, $CDCl_3$):

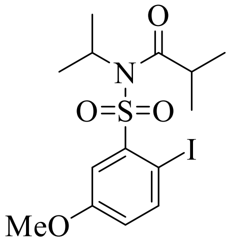

1k

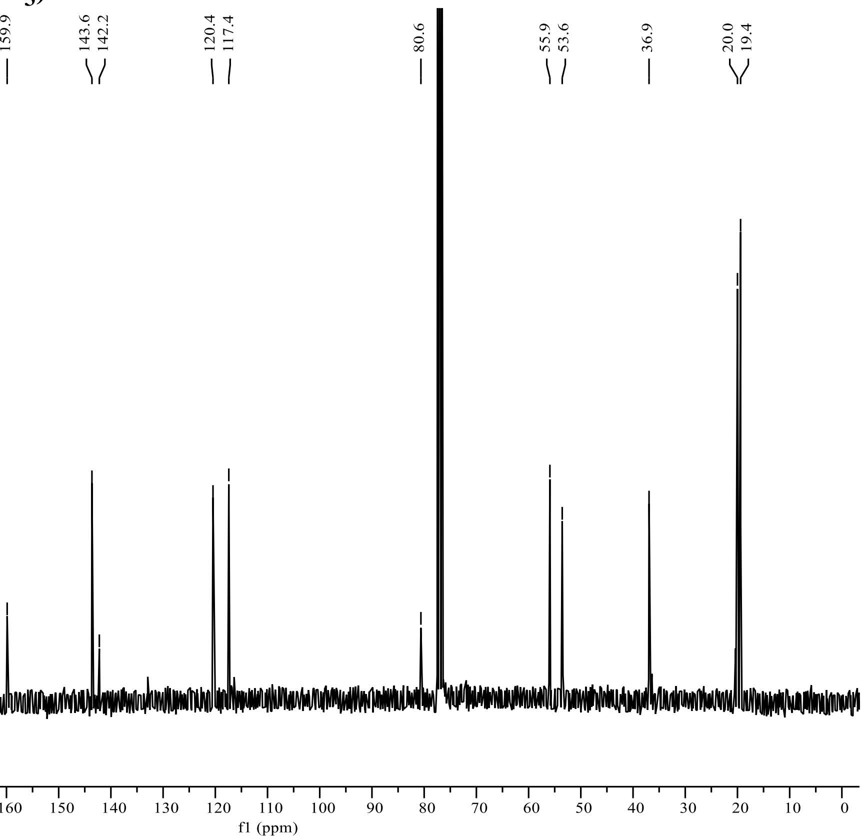

^{1}H NMR (300 MHz, $CDCl_3$):

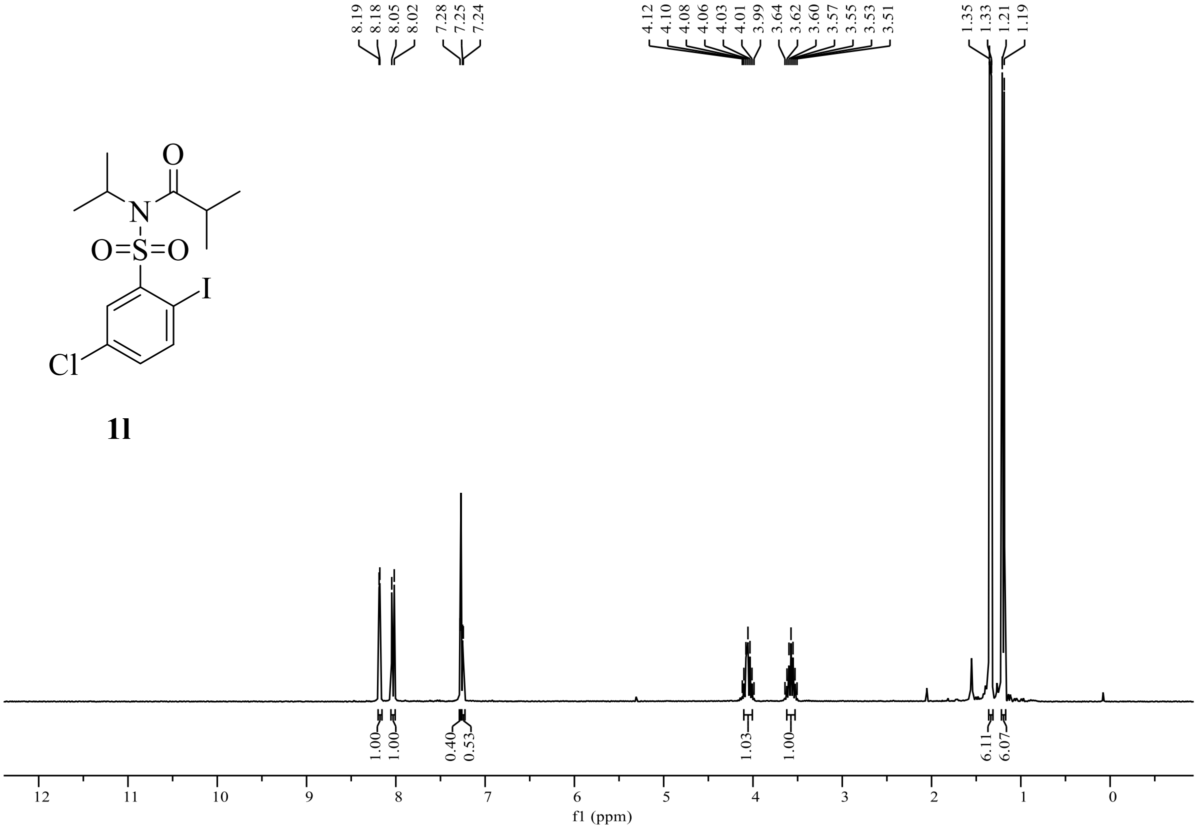

1l

^{13}C NMR (75 MHz, $CDCl_3$):

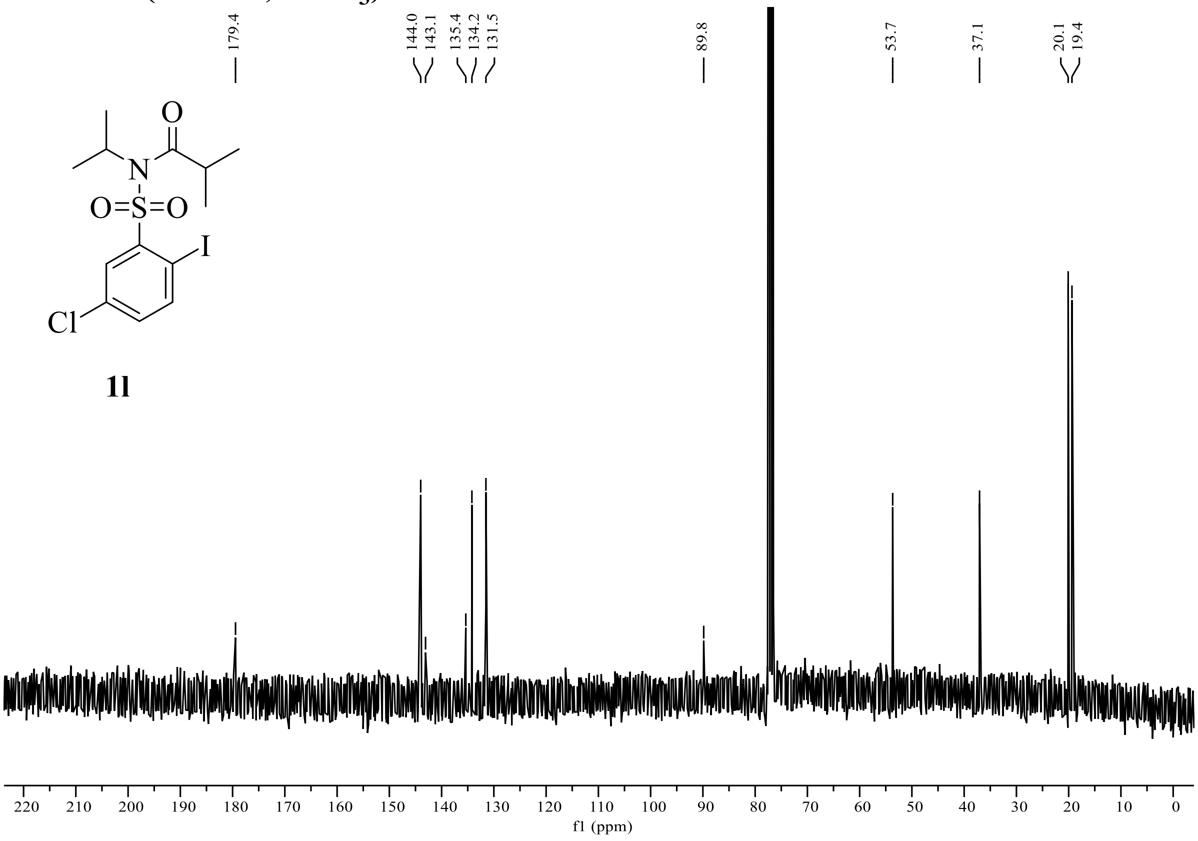

1l

^{1}H NMR (600 MHz, $CDCl_3$):

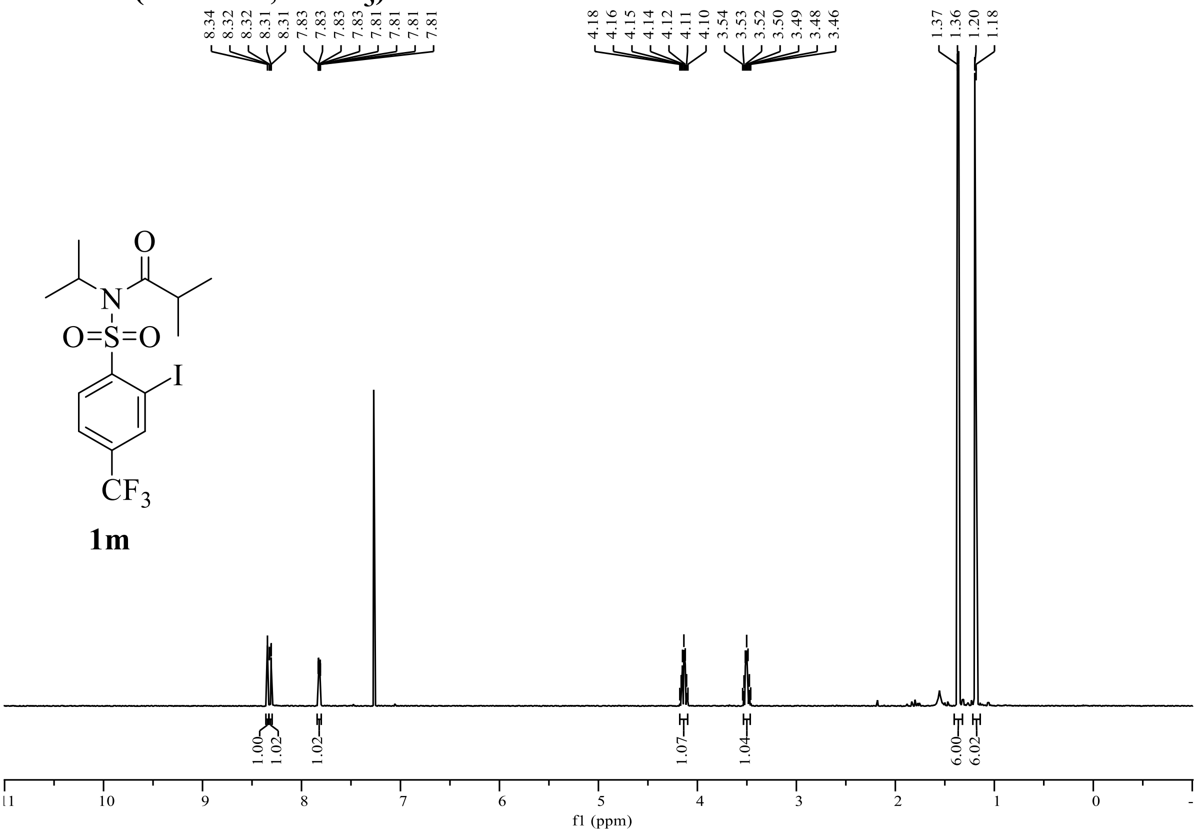

1m

$^{13}C\{^{19}F\}$ NMR (151 MHz, $CDCl_3$):

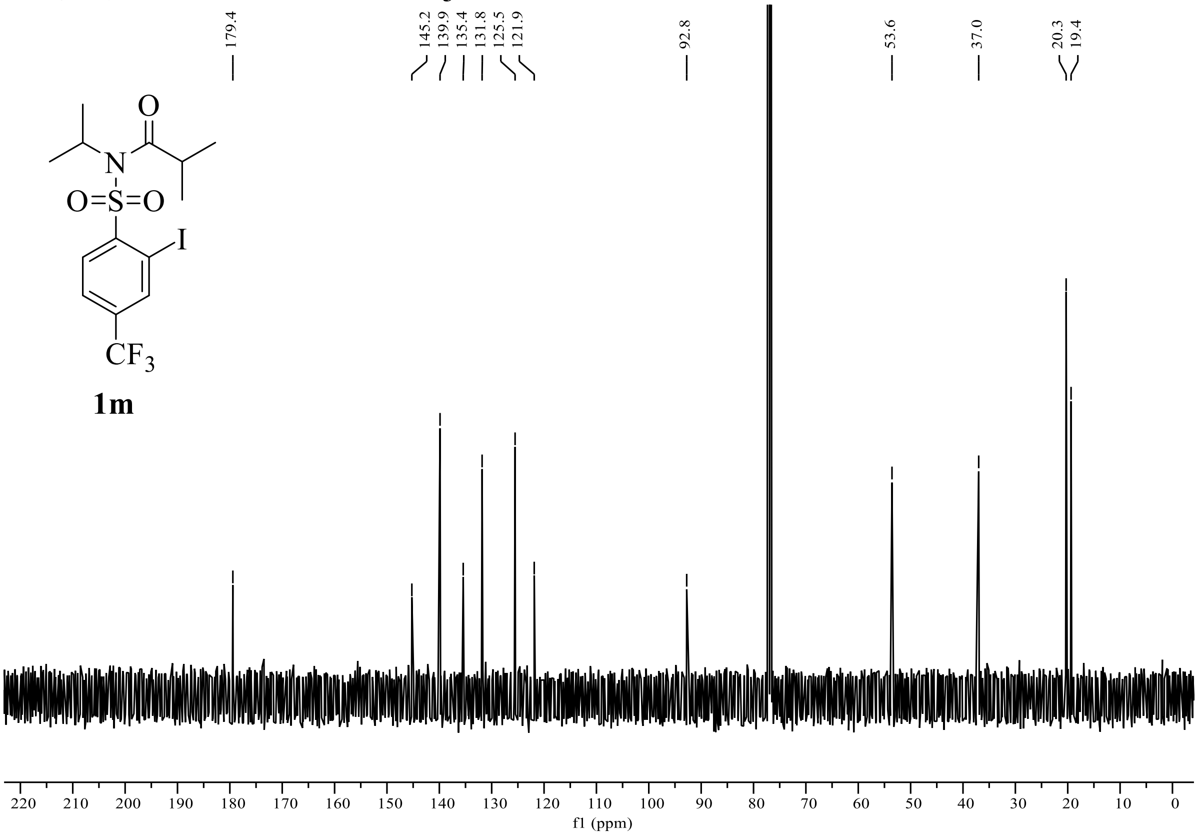

1m

$^{19}F\{^1H\}$ NMR (470 MHz, $CDCl_3$):

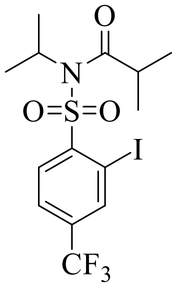

1m

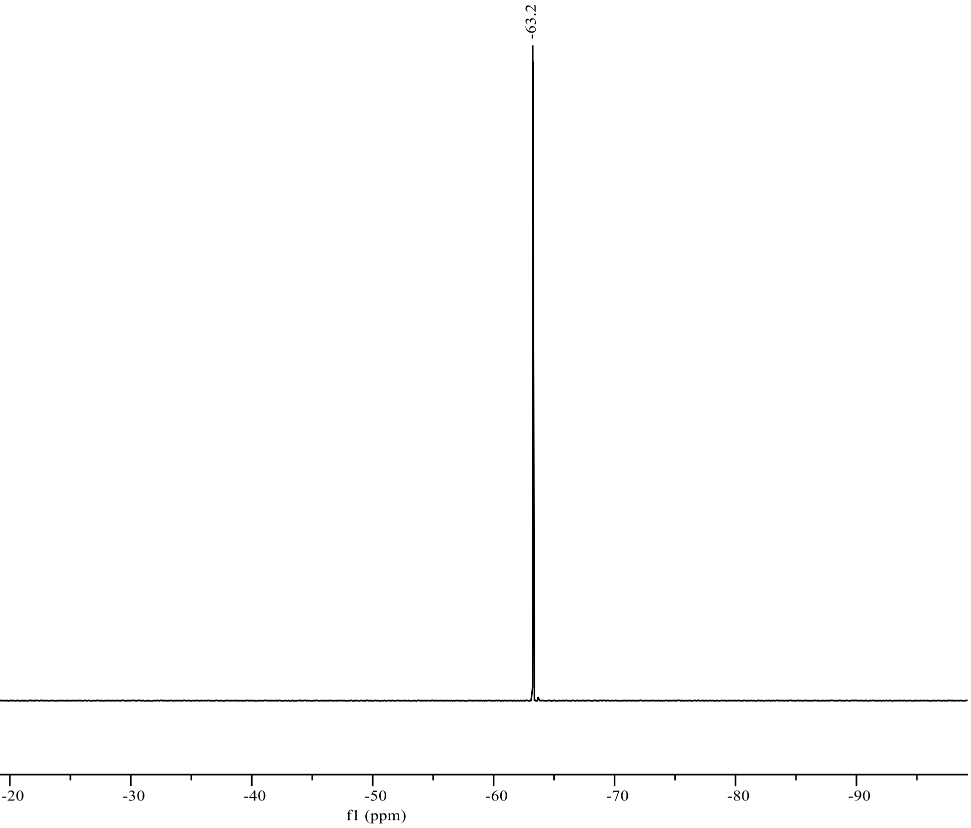

^{1}H NMR (300 MHz, CDCl$_3$):

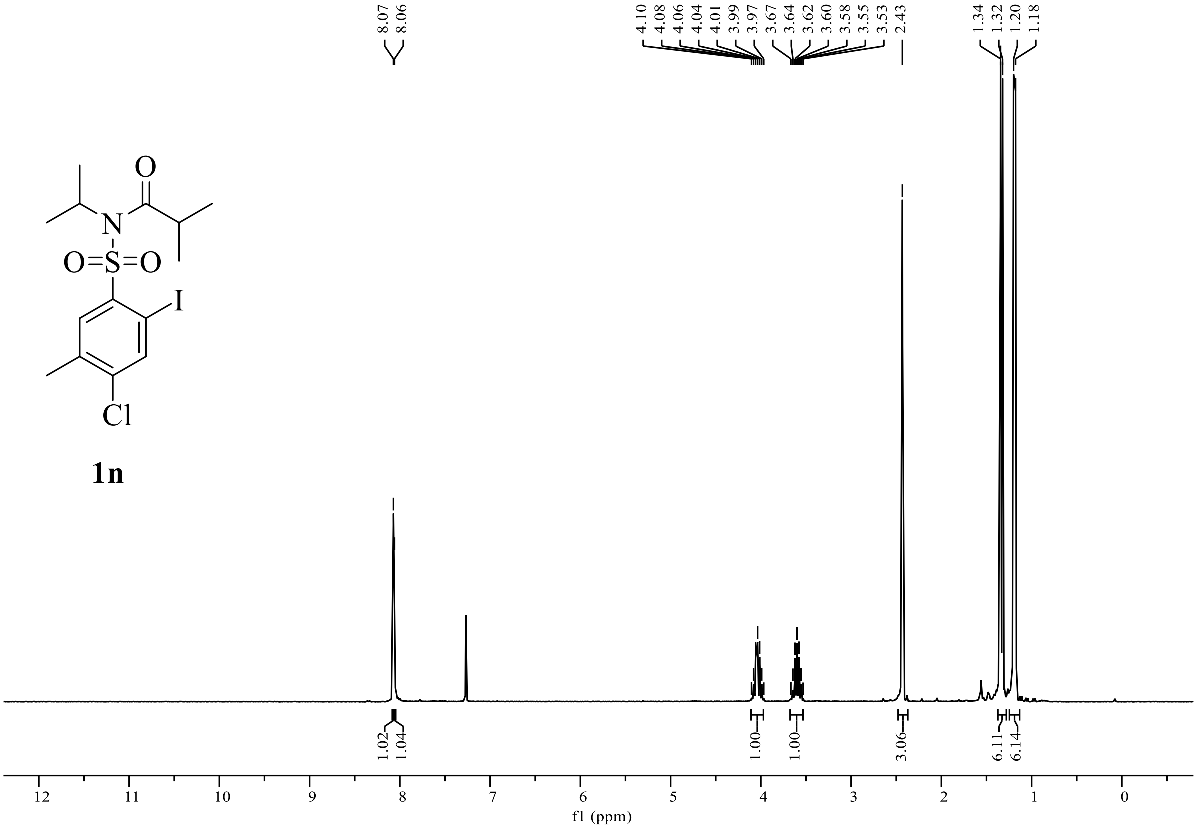

1n

^{13}C NMR (75 MHz, CDCl$_3$):

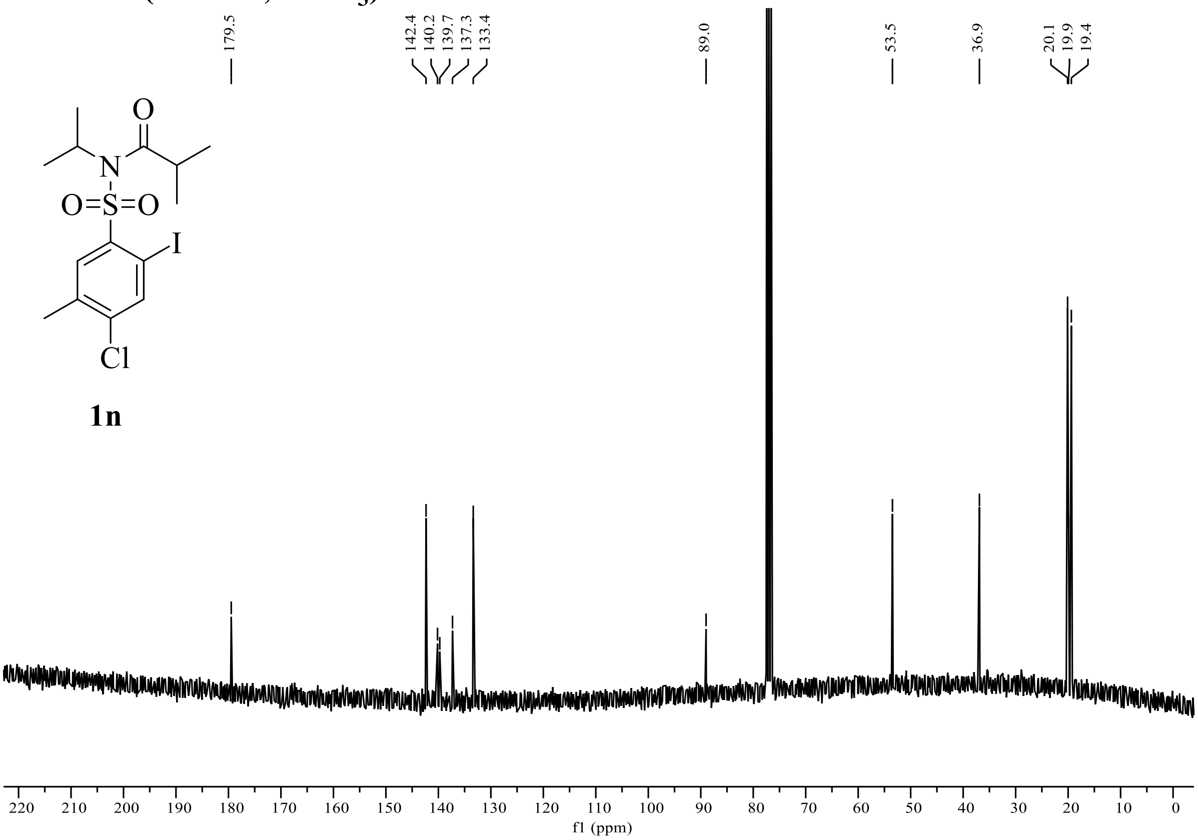

1n

^{1}H NMR (300 MHz, $CDCl_3$):

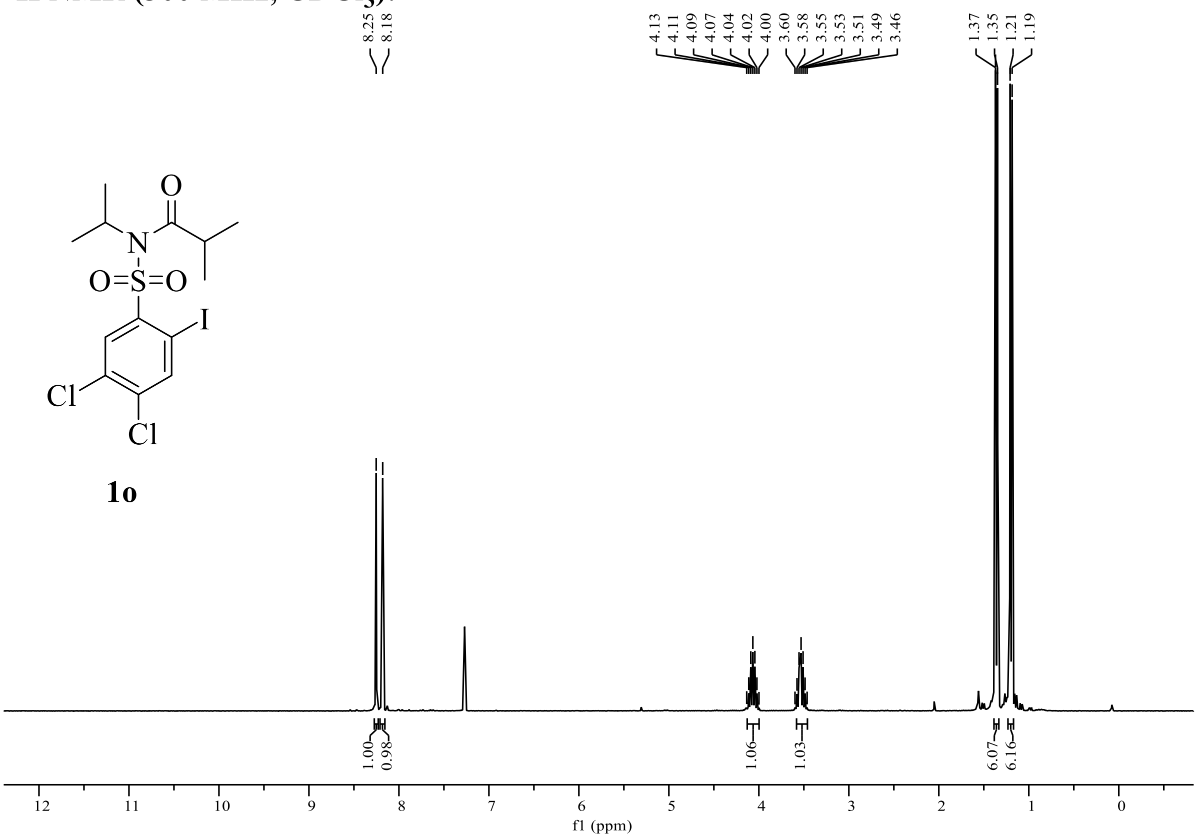

^{13}C NMR (75 MHz, $CDCl_3$):

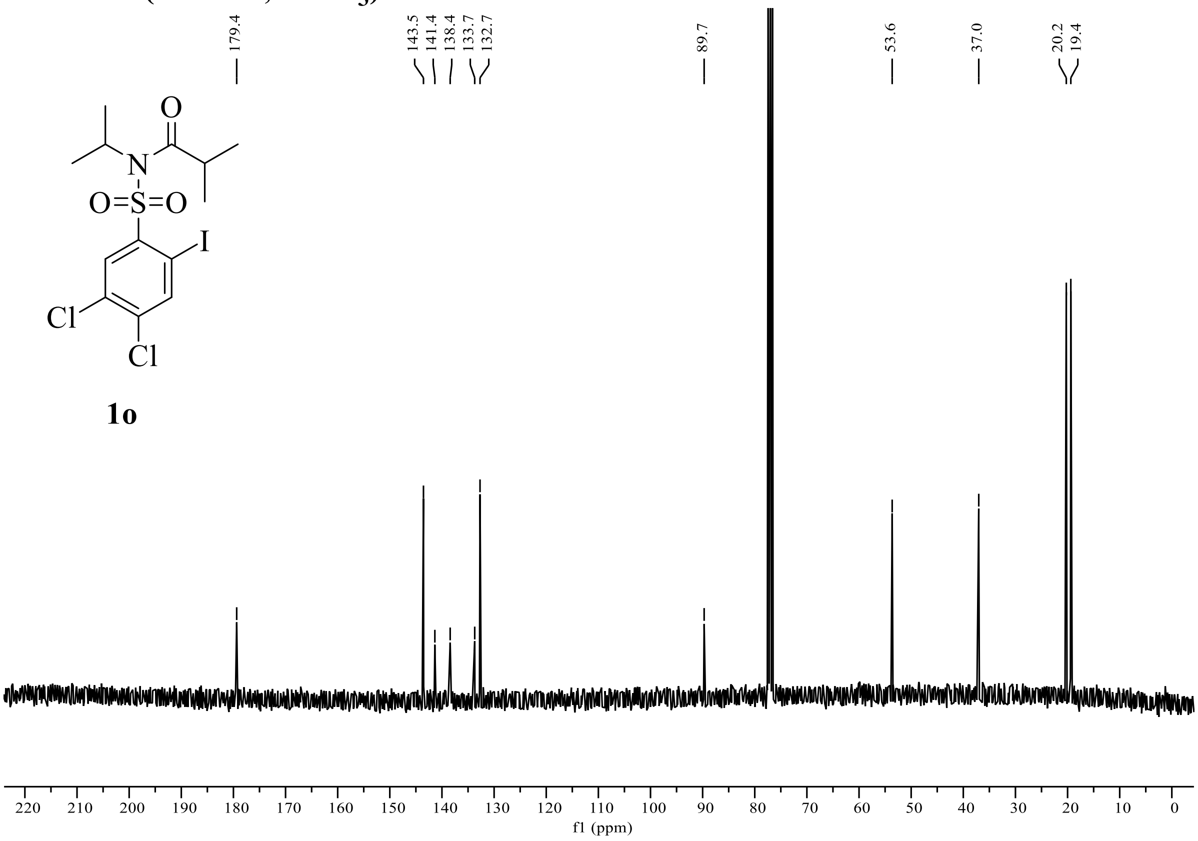

^{1}H NMR (300 MHz, $CDCl_3$):

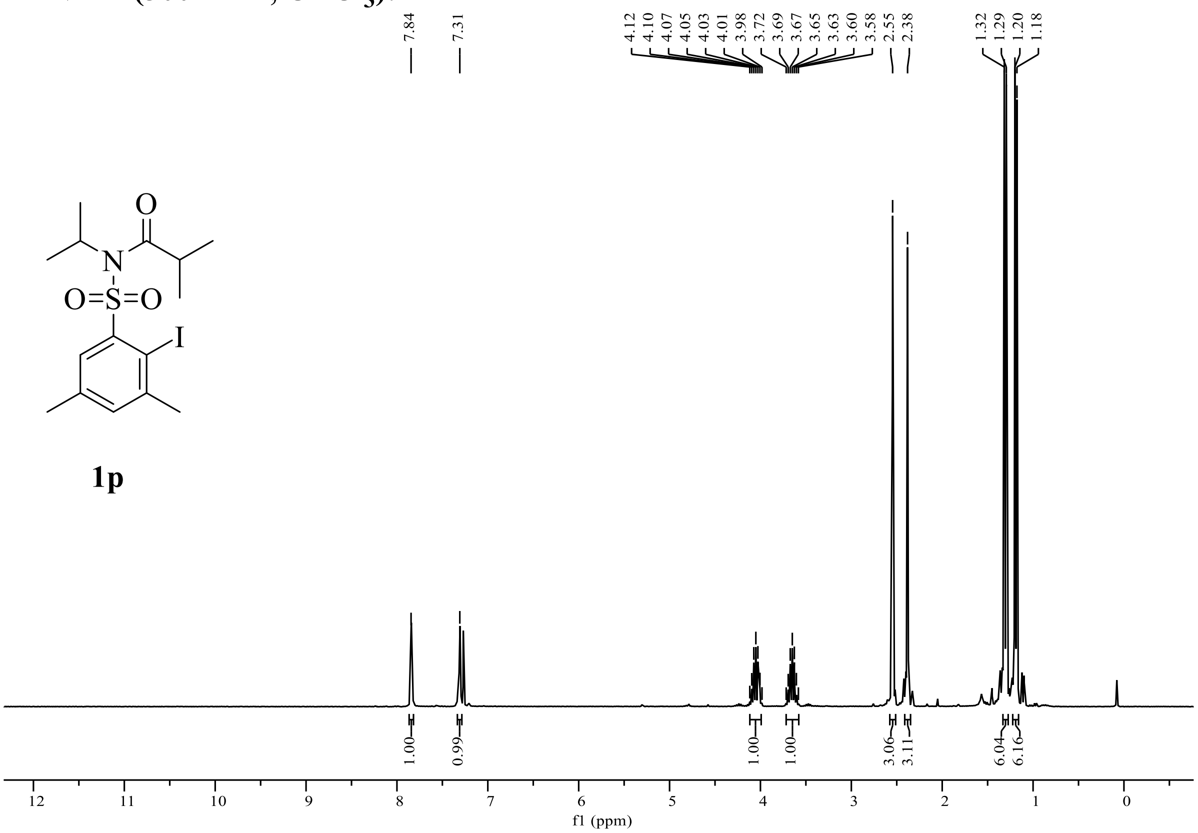

^{13}C NMR (75 MHz, $CDCl_3$):

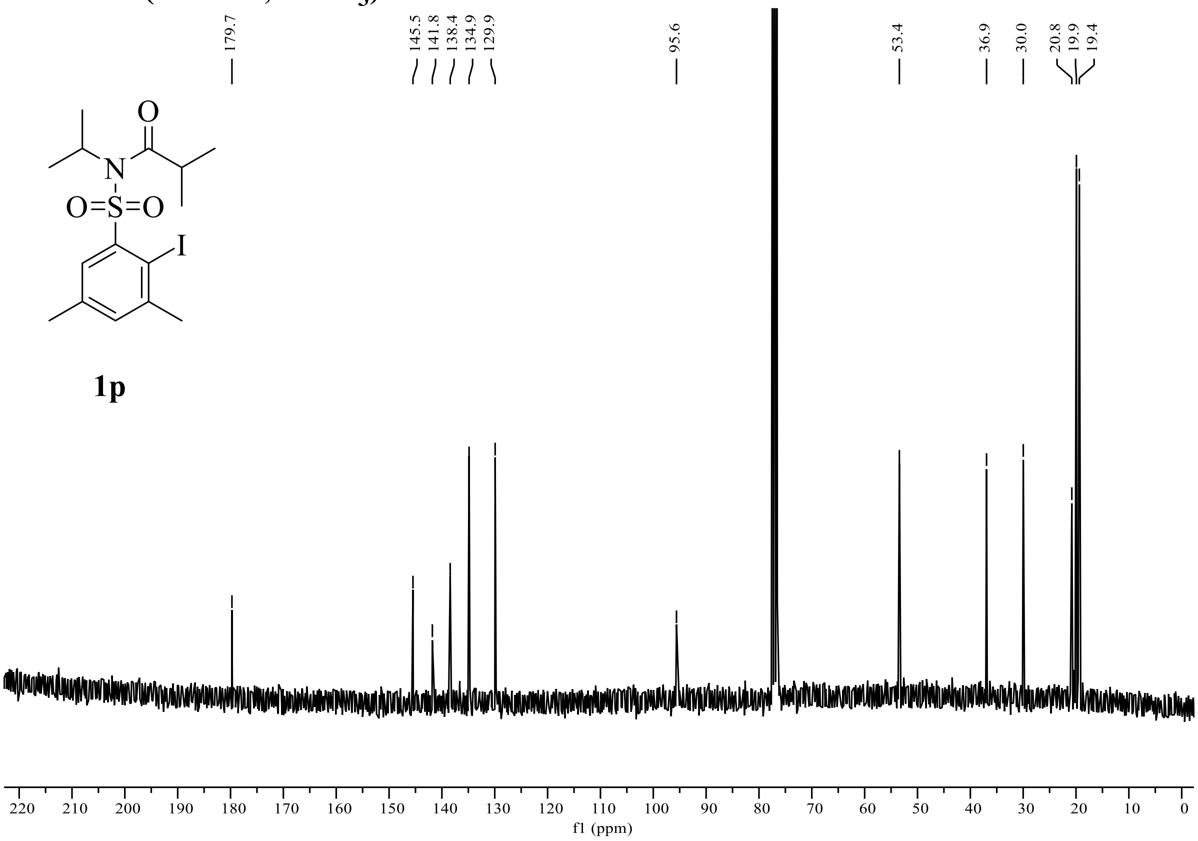

^{1}H NMR (300 MHz, $CDCl_3$):

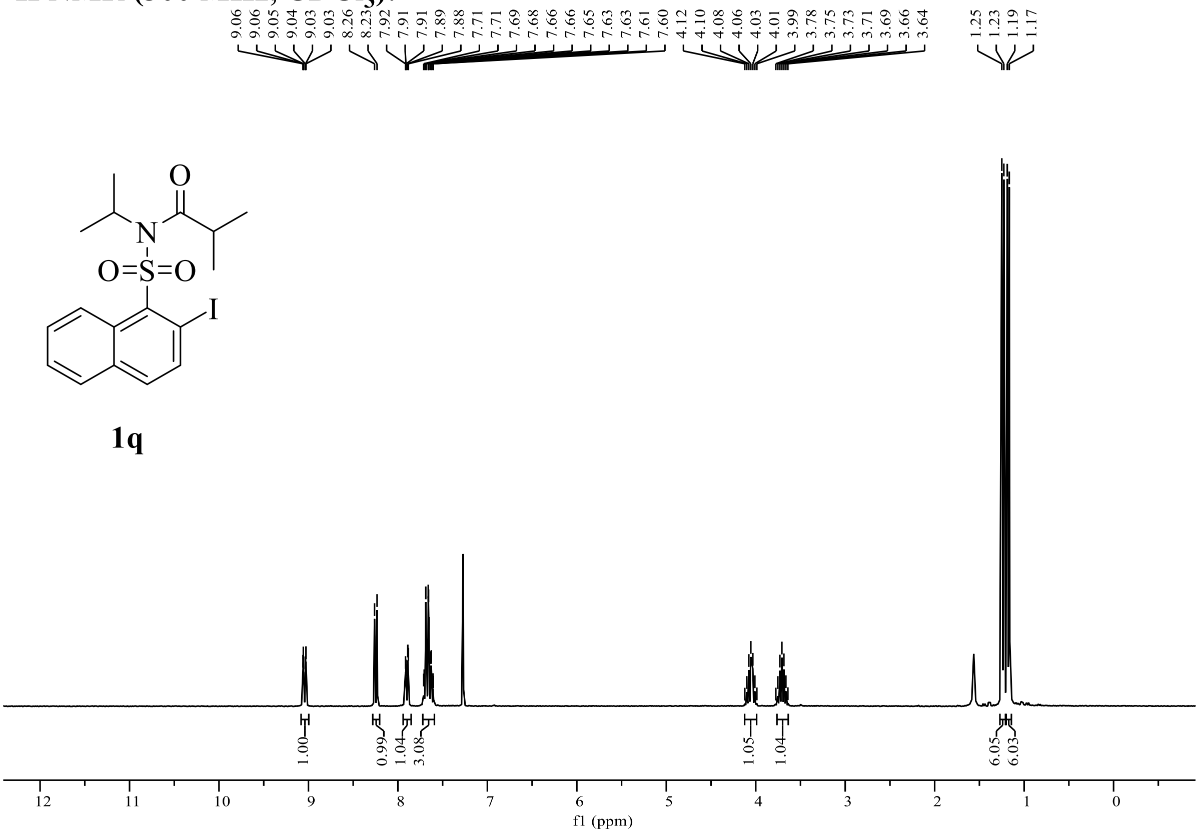

1q

^{13}C NMR (75 MHz, $CDCl_3$):

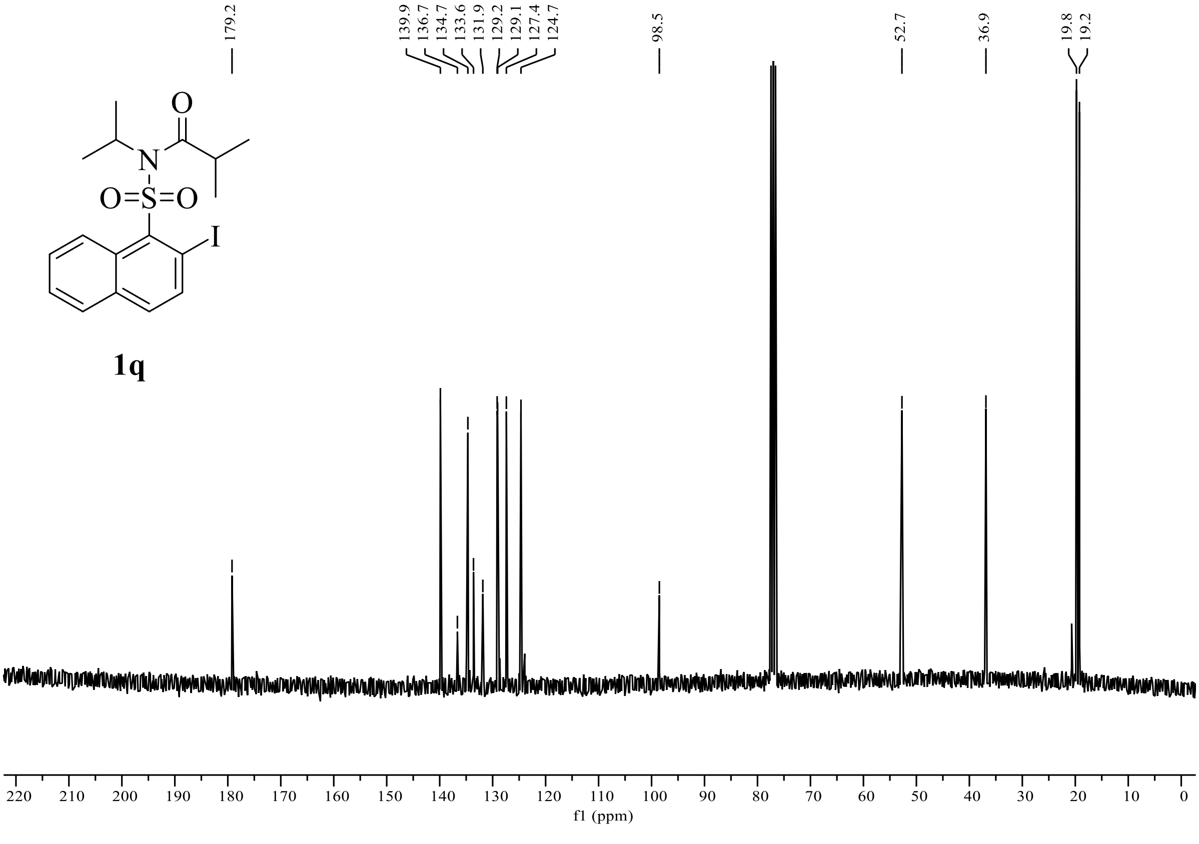

1q

^{1}H NMR (300 MHz, $CDCl_3$):

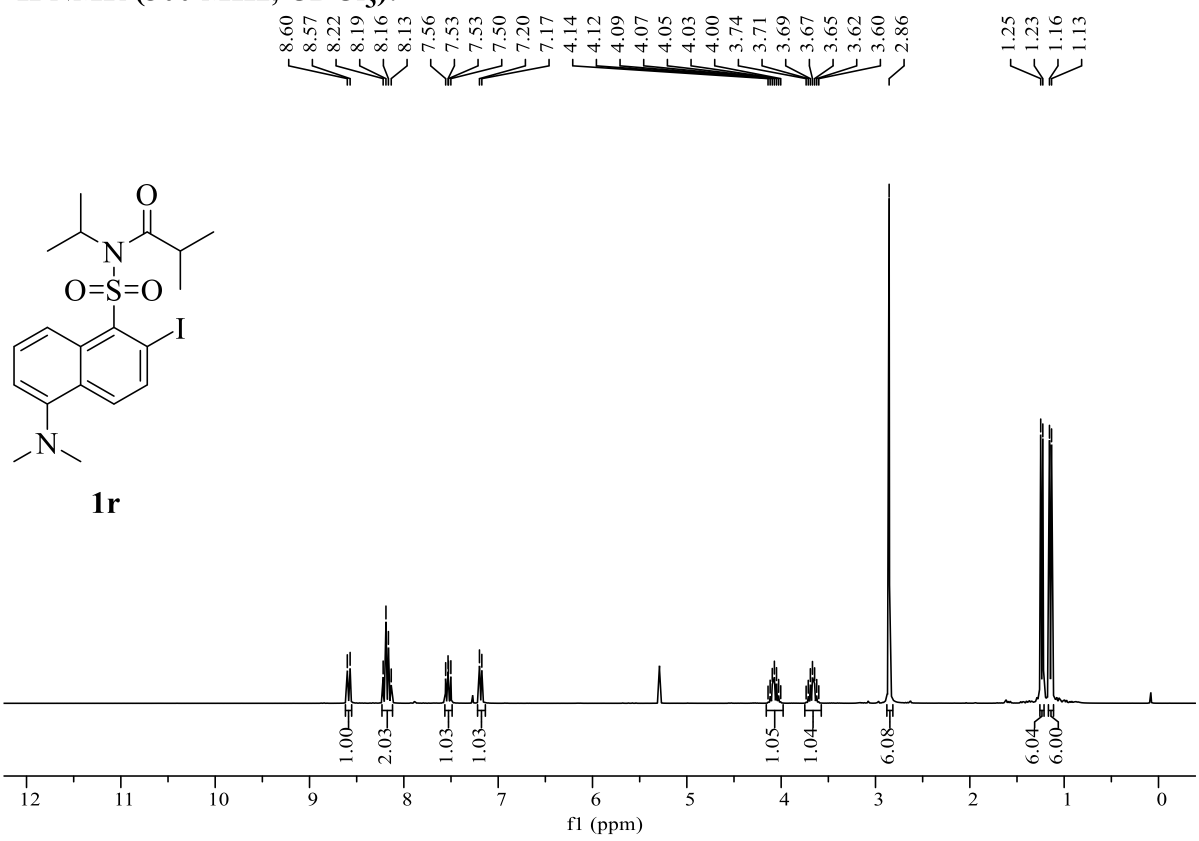

^{13}C NMR (75 MHz, $CDCl_3$):

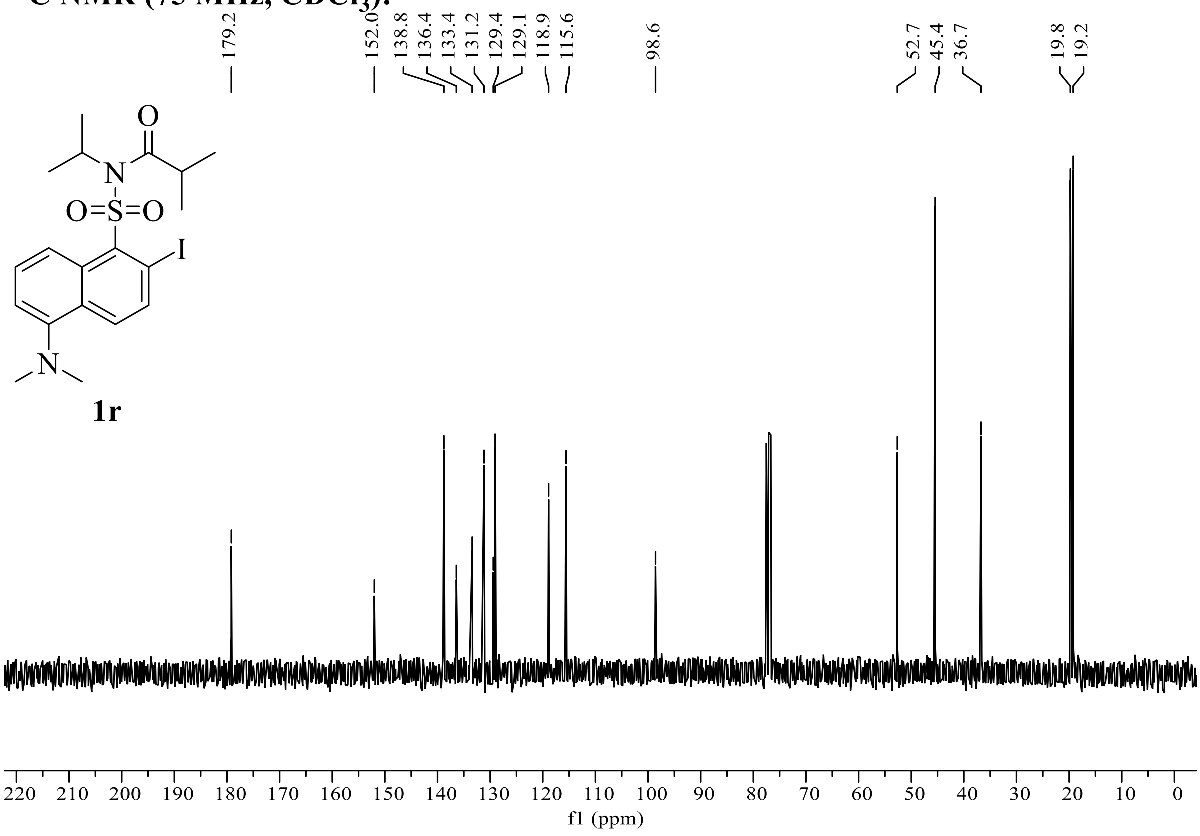

^{1}H NMR (600 MHz, $CDCl_3$):

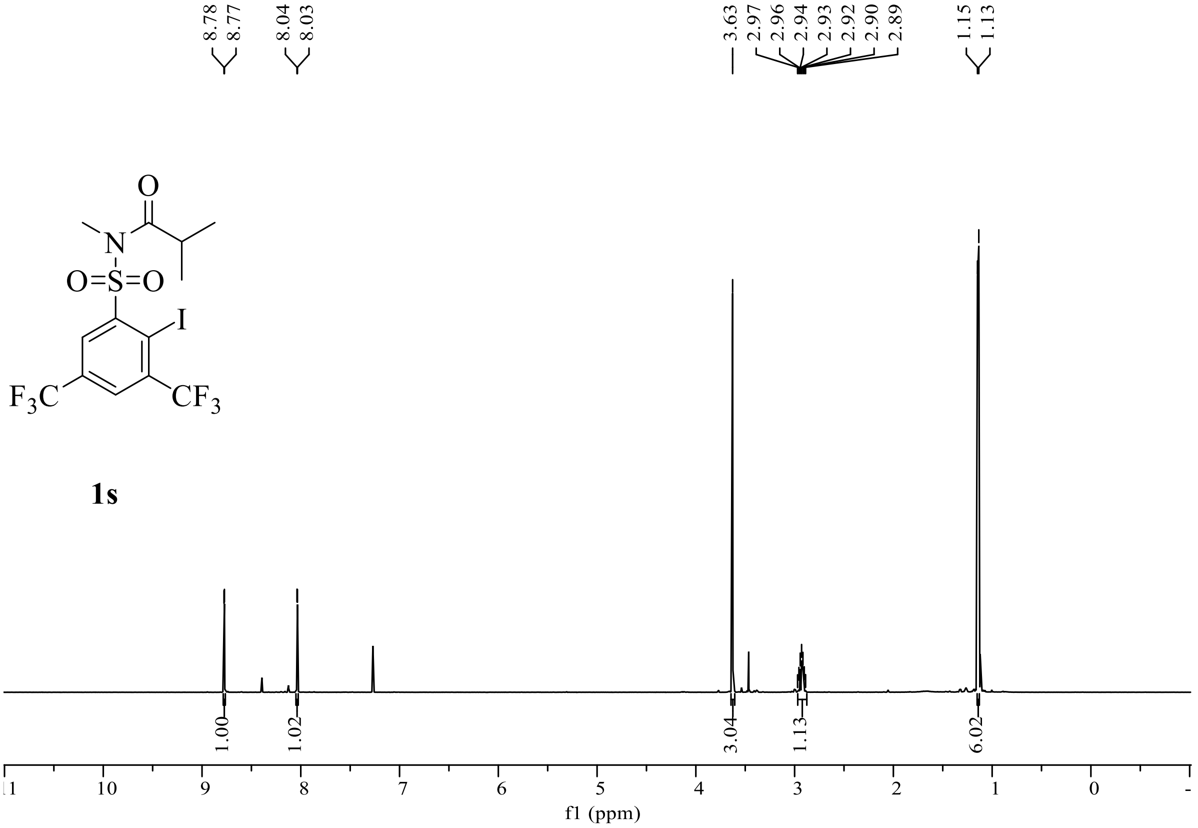

1s

$^{13}C\{^{19}F\}$ NMR (151 MHz, $CDCl_3$):

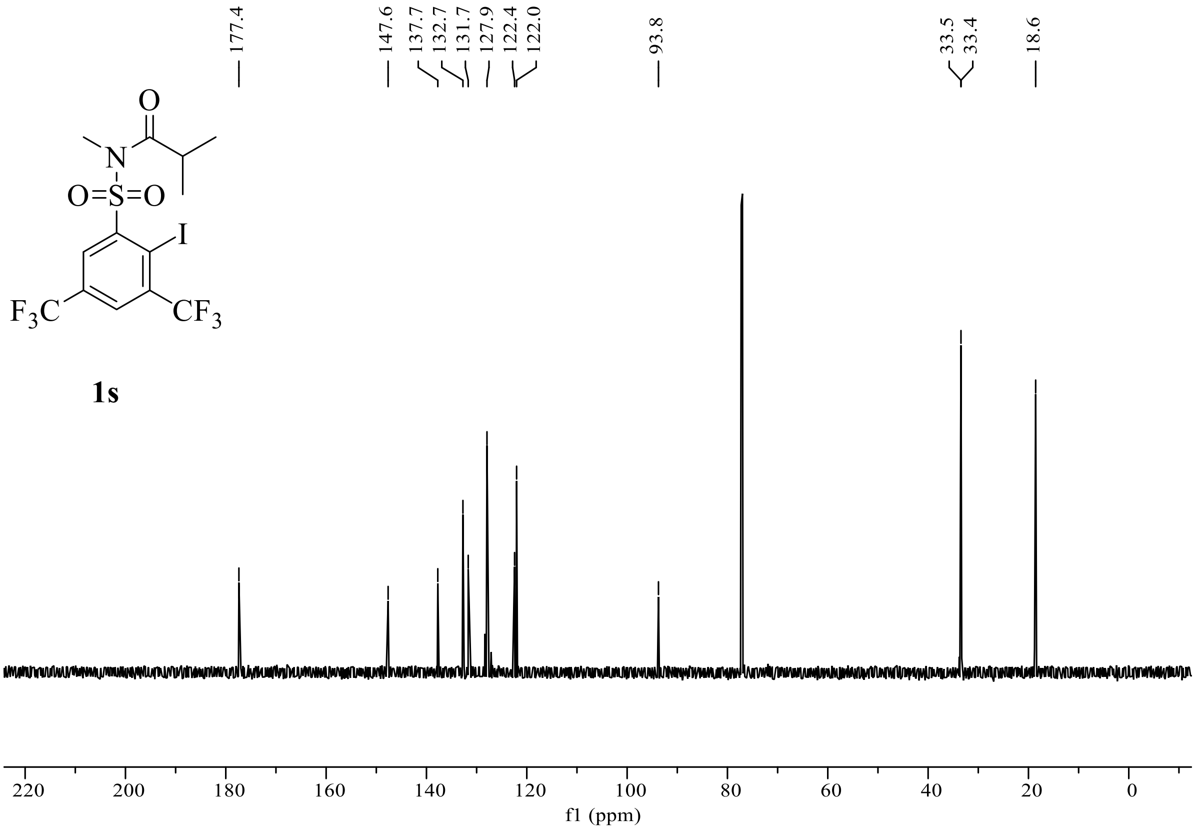

1s

$^{19}F\{^{1}H\}$ NMR (470 MHz, $CDCl_3$):

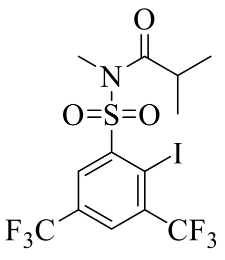

1s

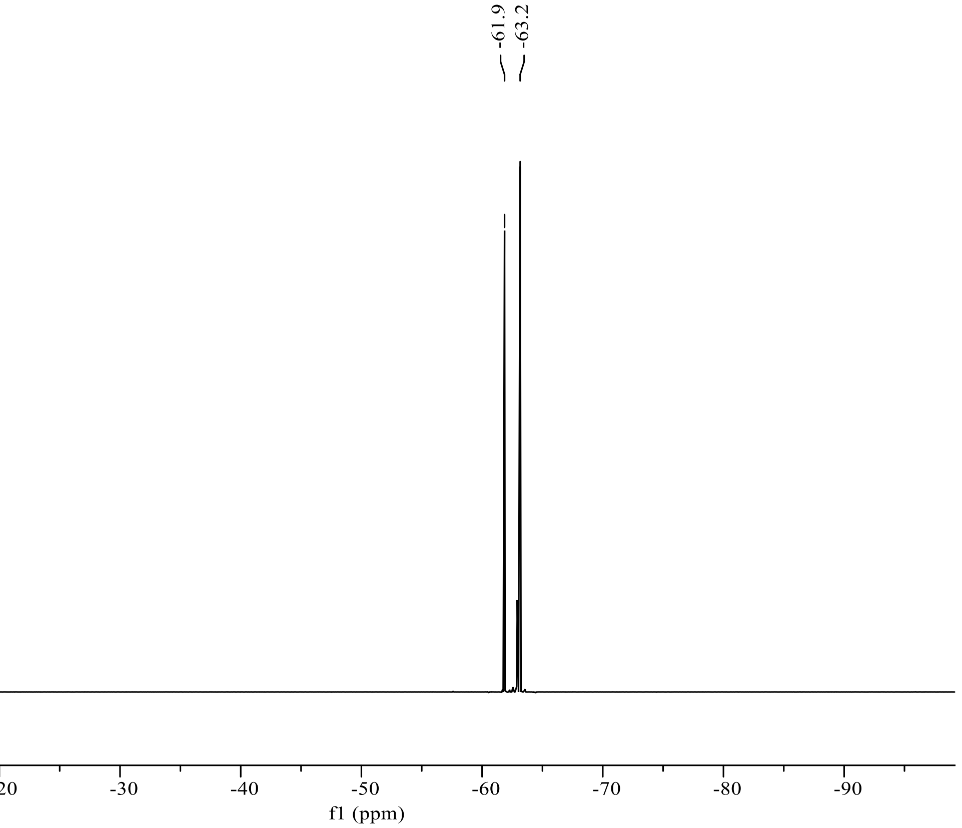

^{1}H NMR (300 MHz, $CDCl_3$):

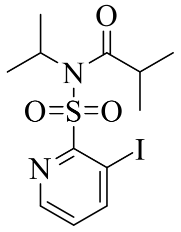

1t

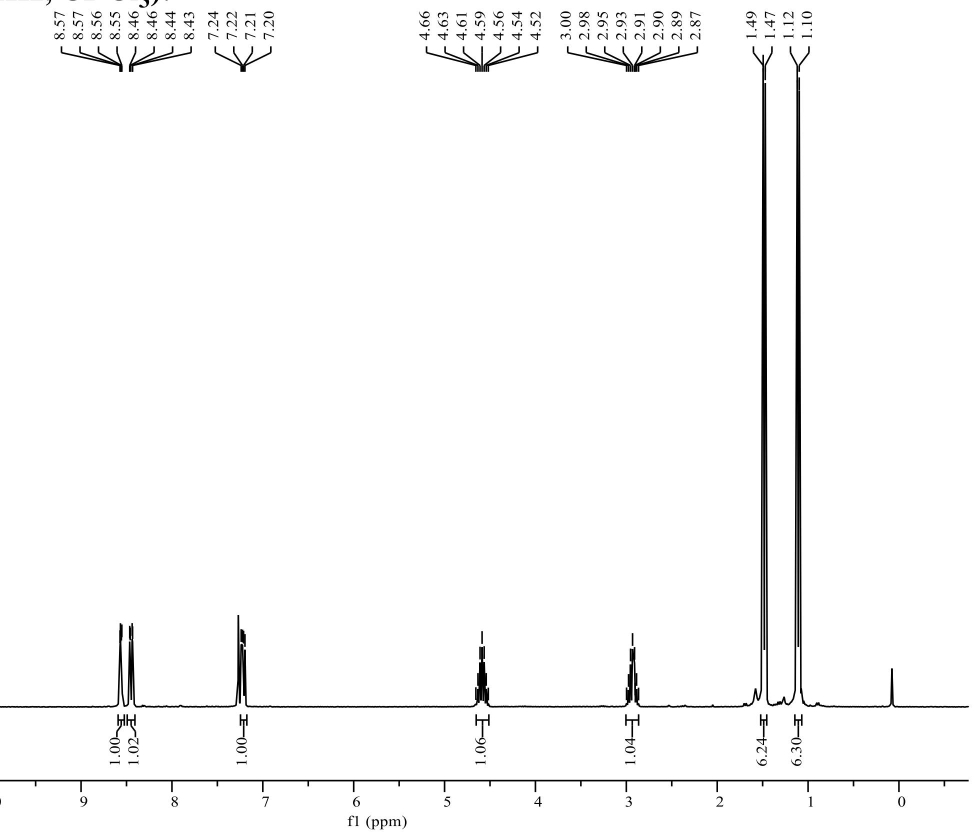

^{13}C NMR (75 MHz, $CDCl_3$):

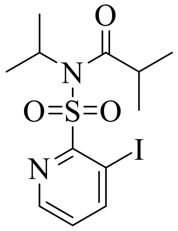

1t

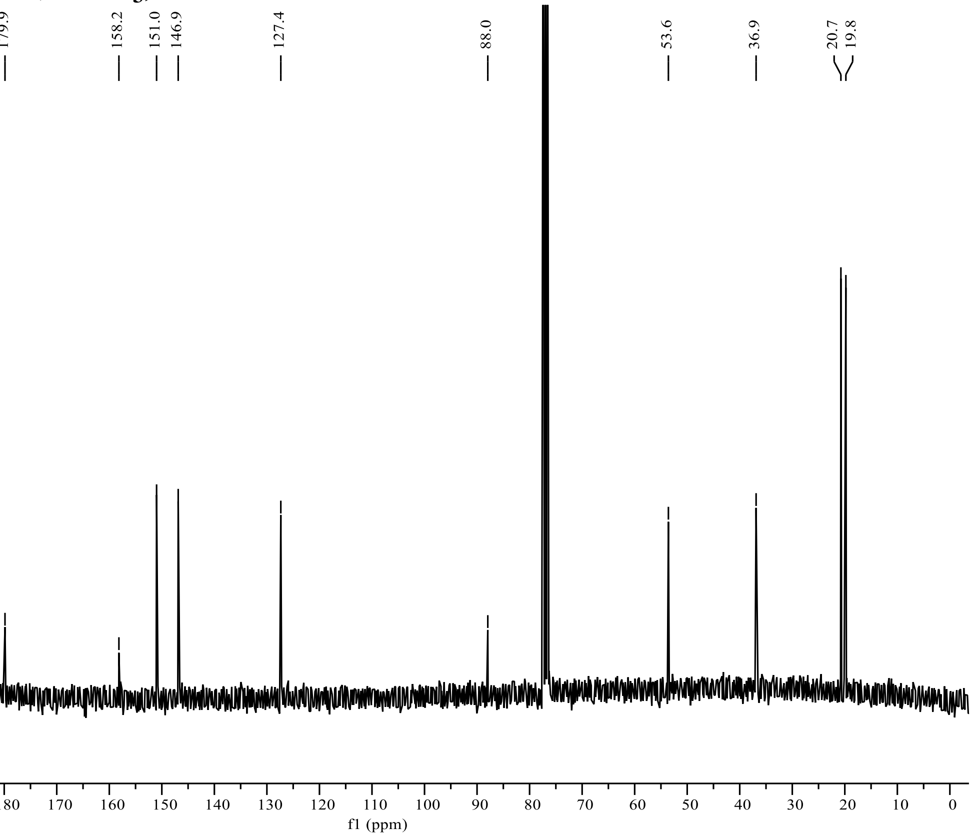

^{1}H NMR (300 MHz, $CDCl_3$):

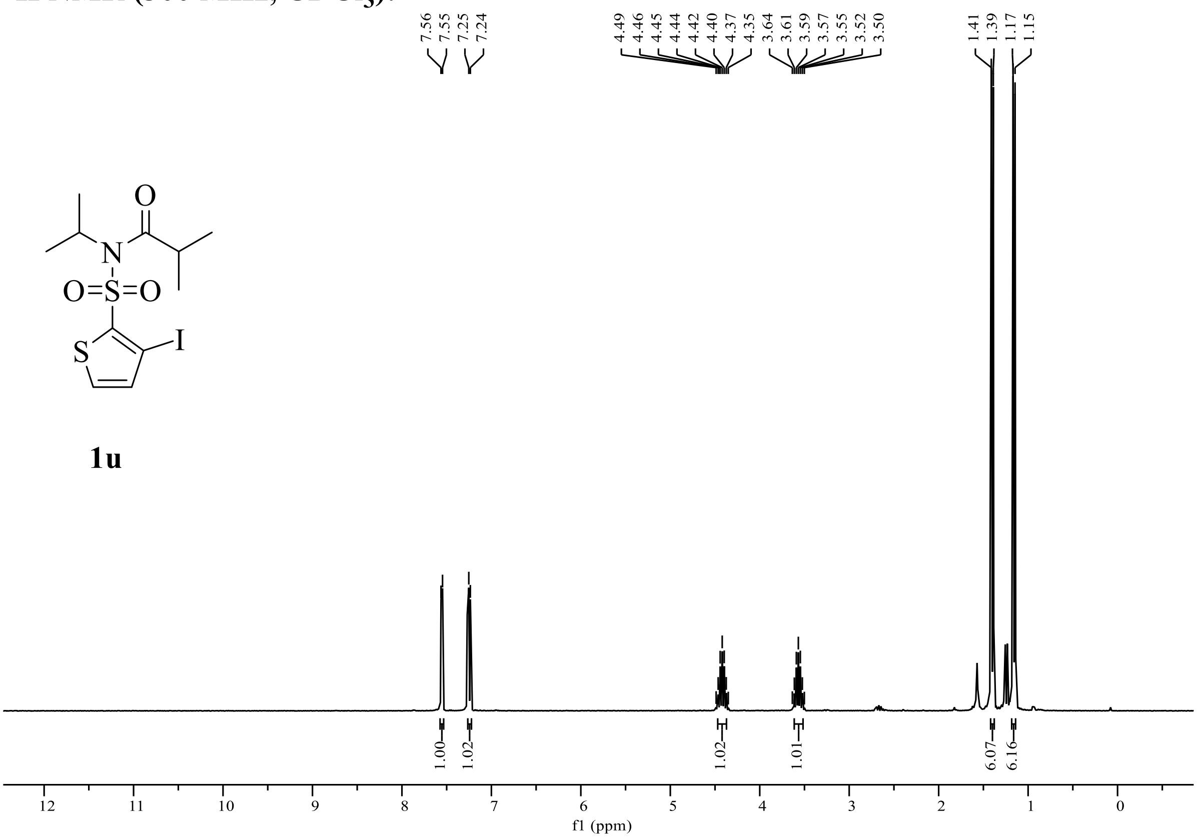

1u

^{13}C NMR (75 MHz, $CDCl_3$):

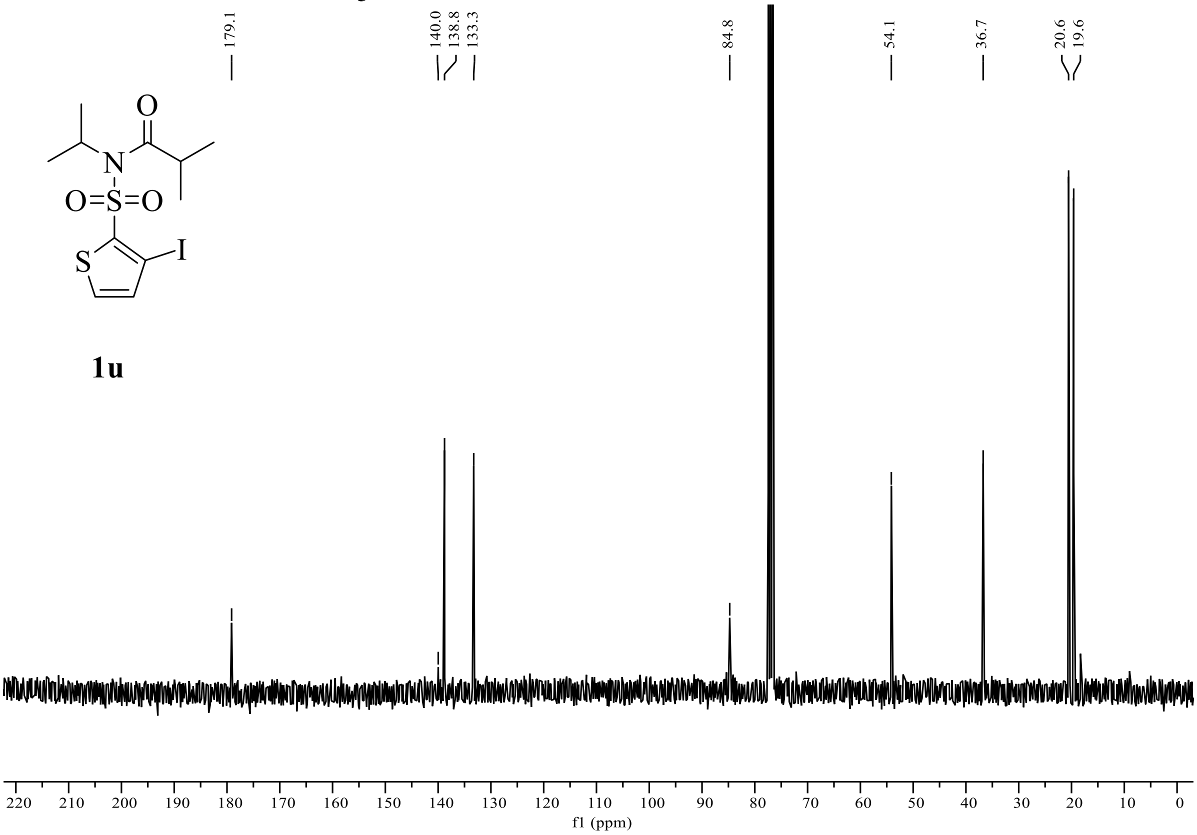

1u

^{1}H NMR (300 MHz, $CDCl_3$):

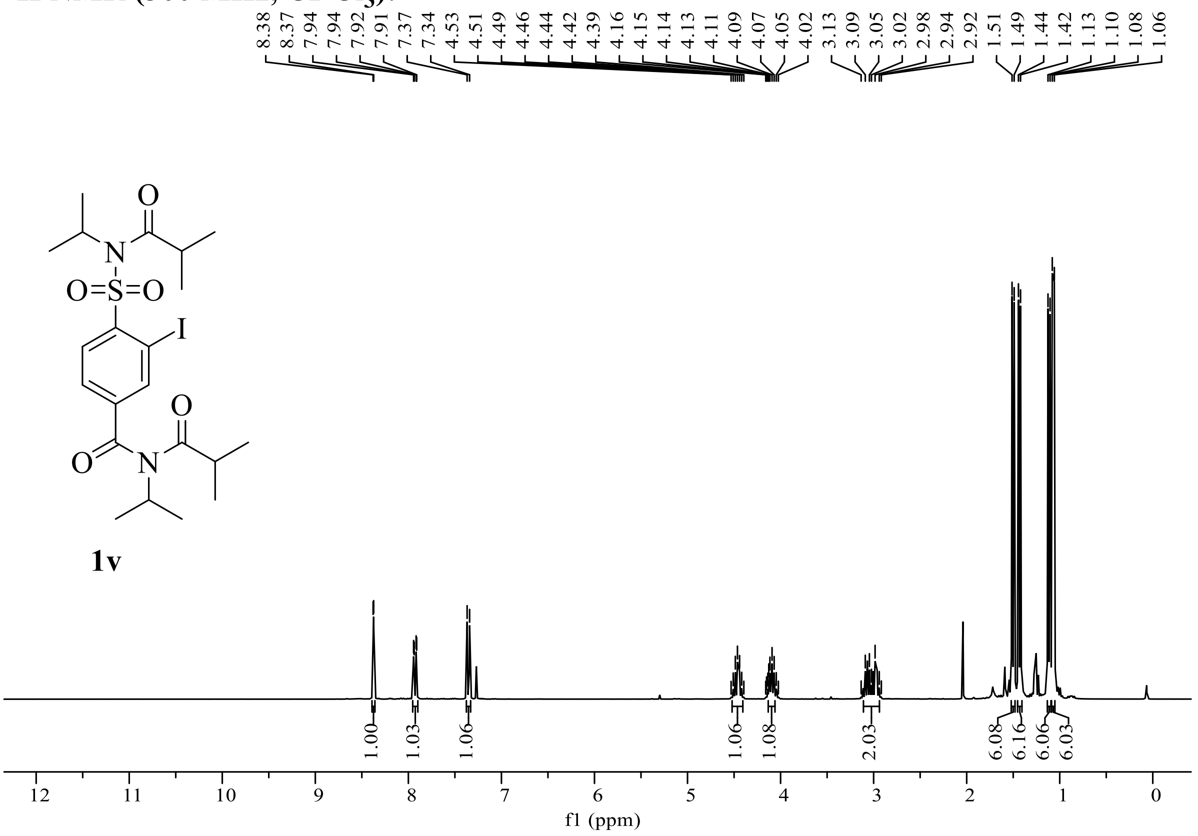

^{13}C NMR (75 MHz, $CDCl_3$):

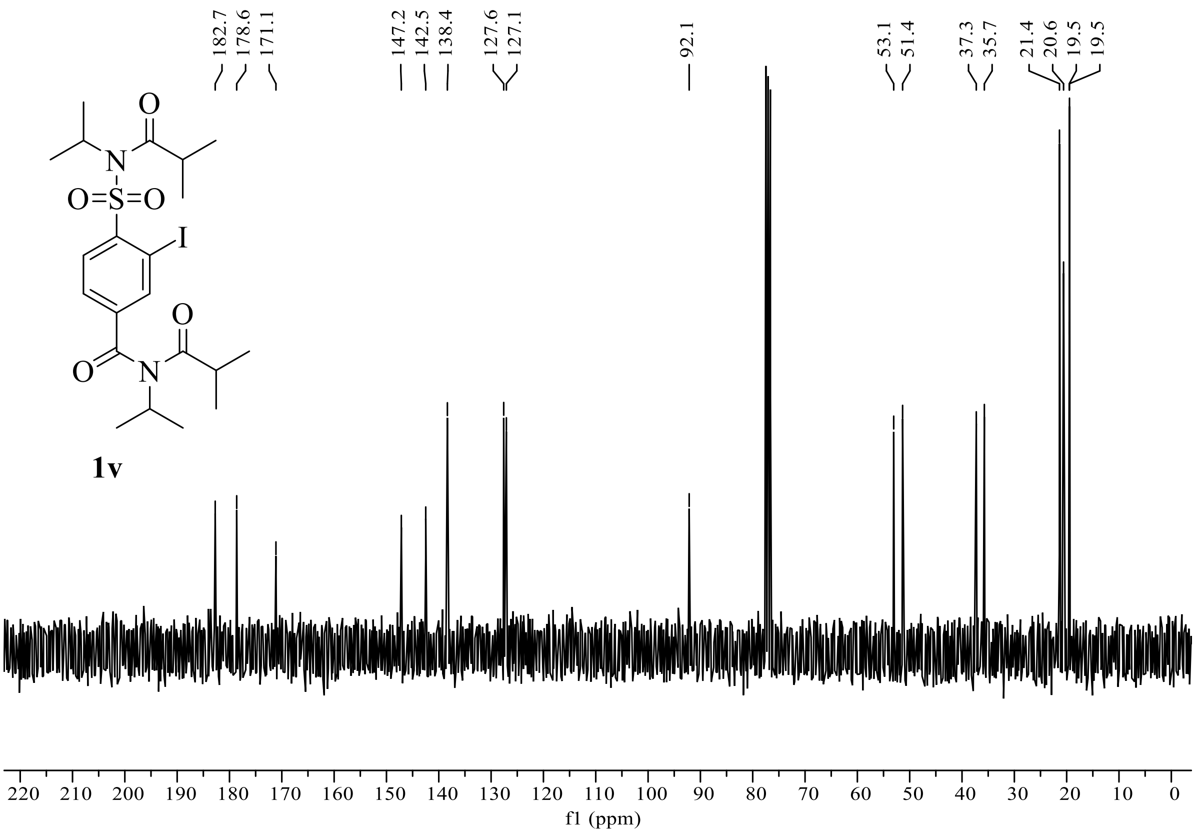

^{1}H NMR (300 MHz, $CDCl_3$):

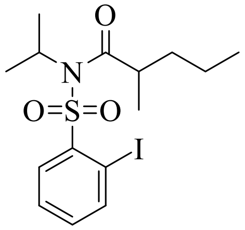

1w

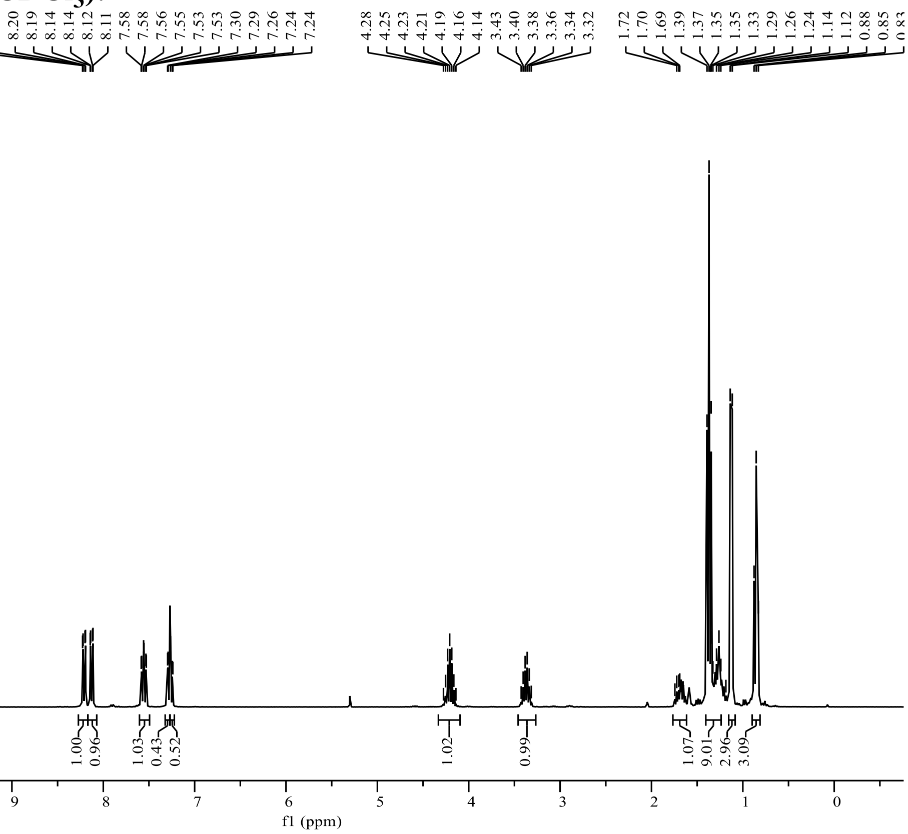

^{13}C NMR (75 MHz, $CDCl_3$):

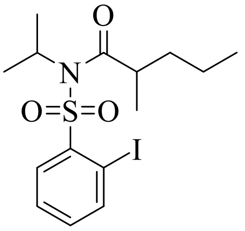

1w

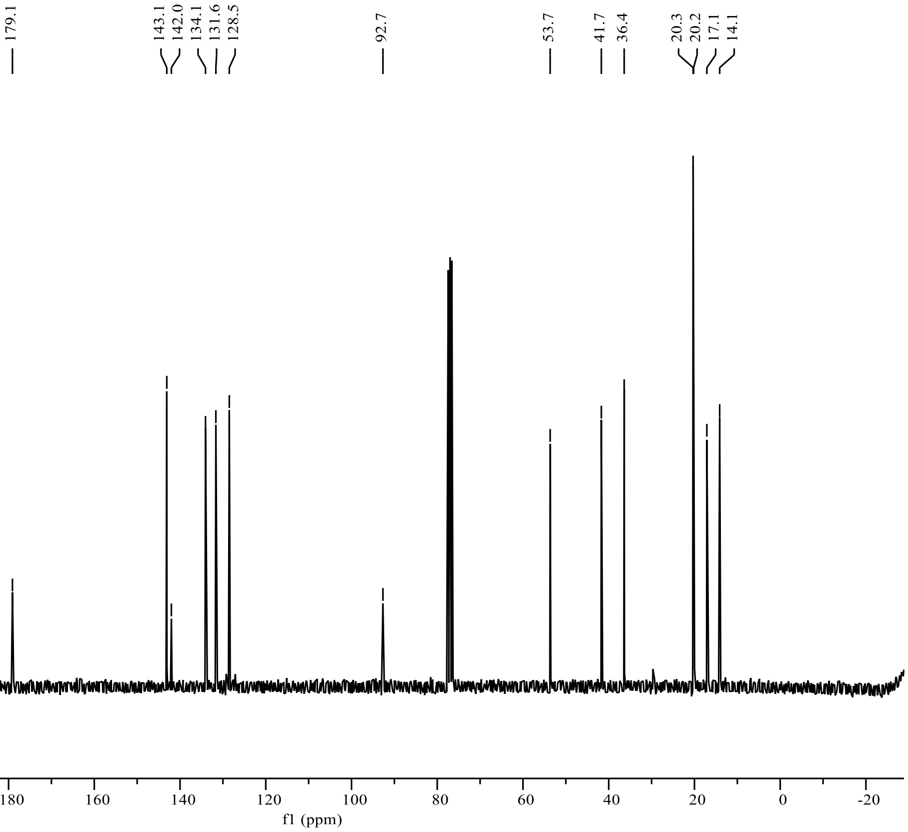

^{1}H NMR (300 MHz, $CDCl_3$):

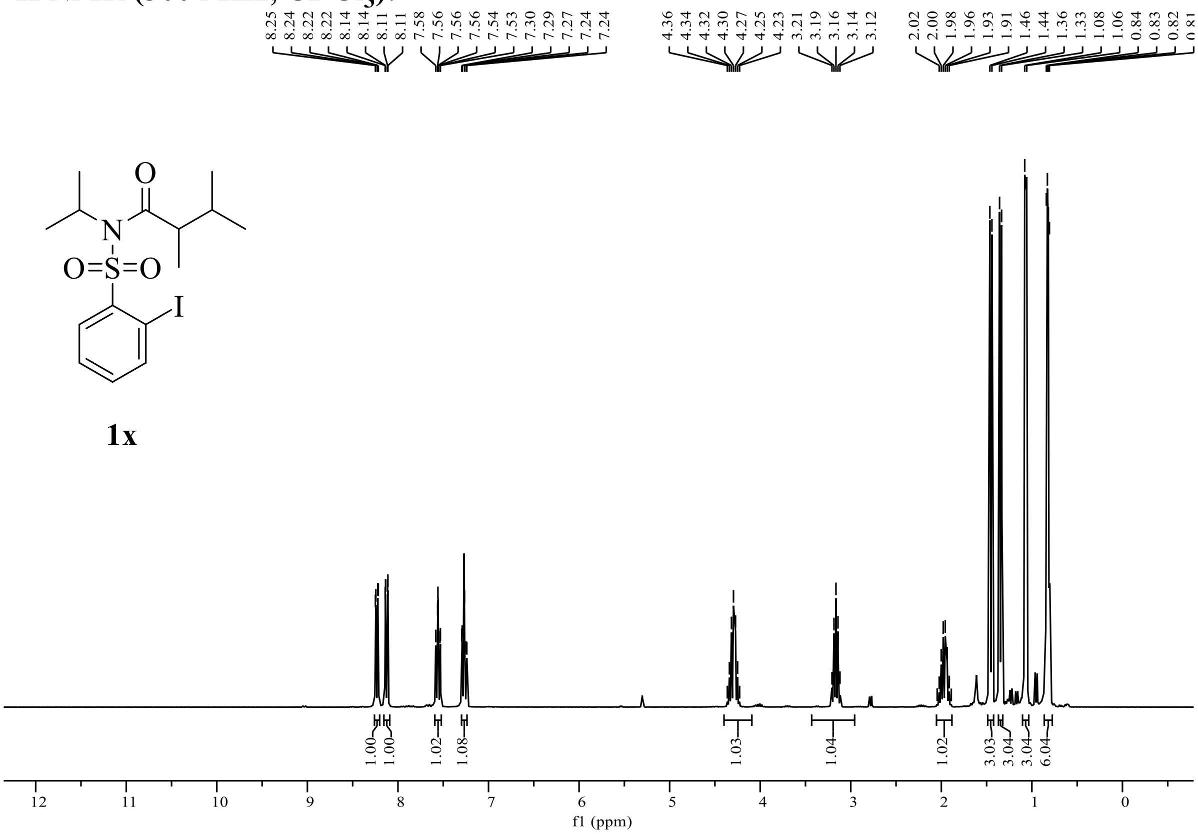

^{13}C NMR (75 MHz, $CDCl_3$):

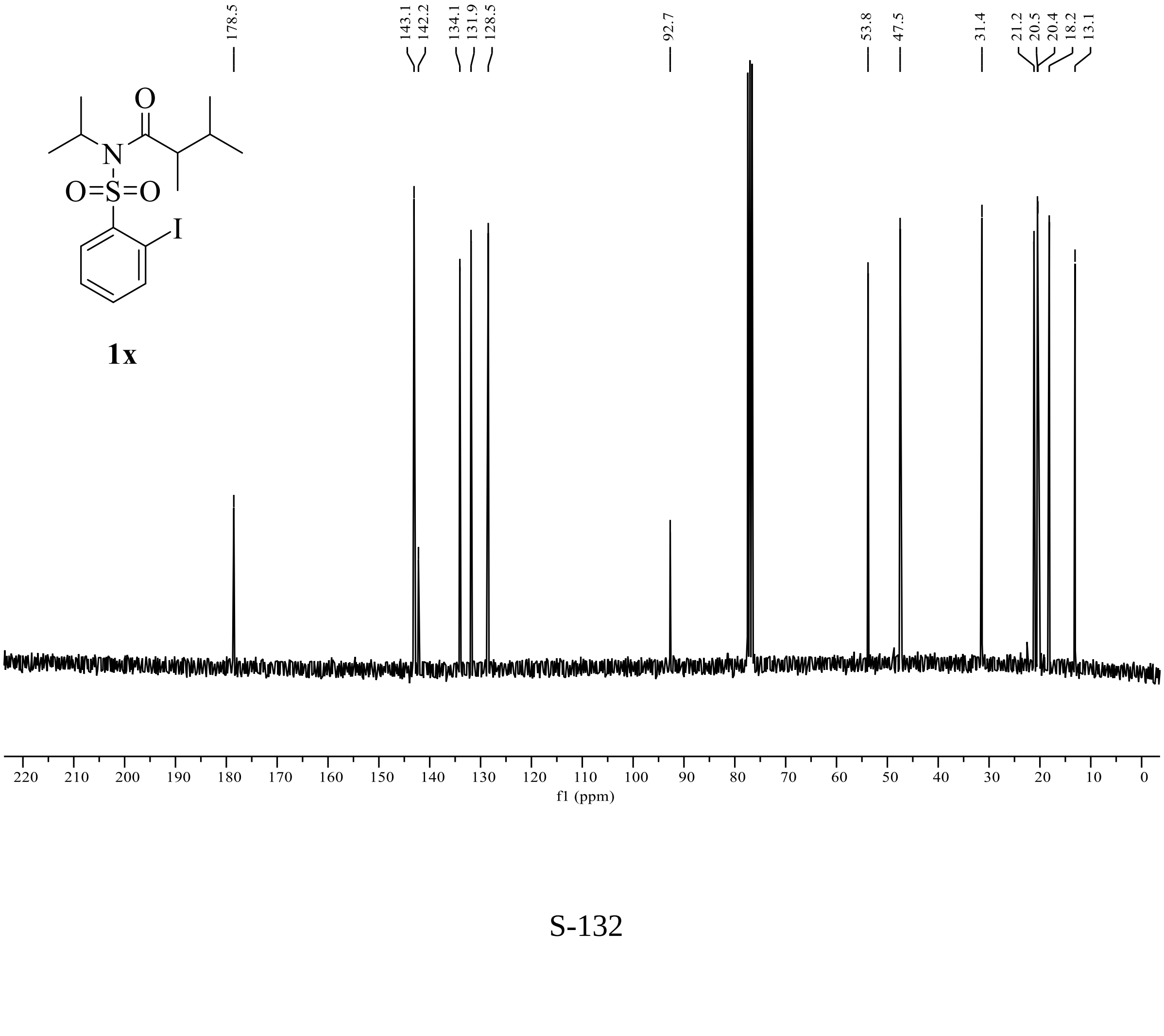

^{1}H NMR (300 MHz, $CDCl_3$):

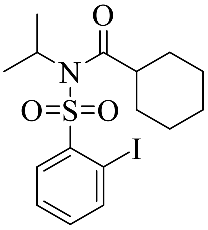

1y

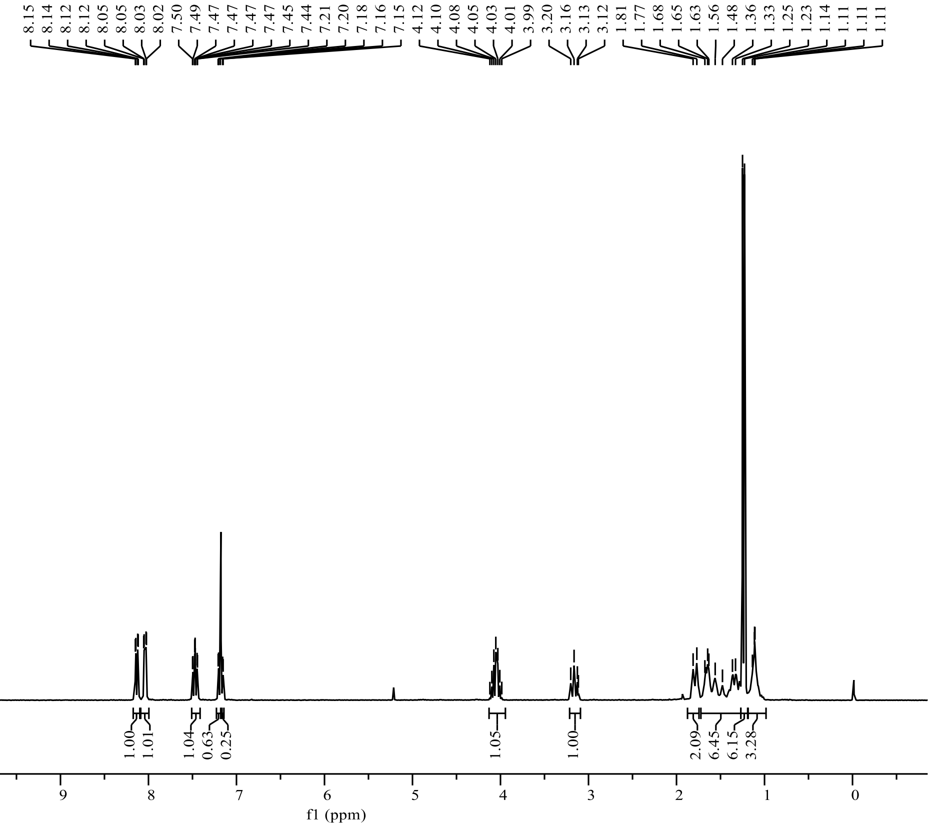

^{13}C NMR (75 MHz, $CDCl_3$):

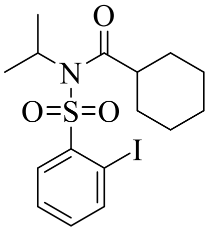

1y

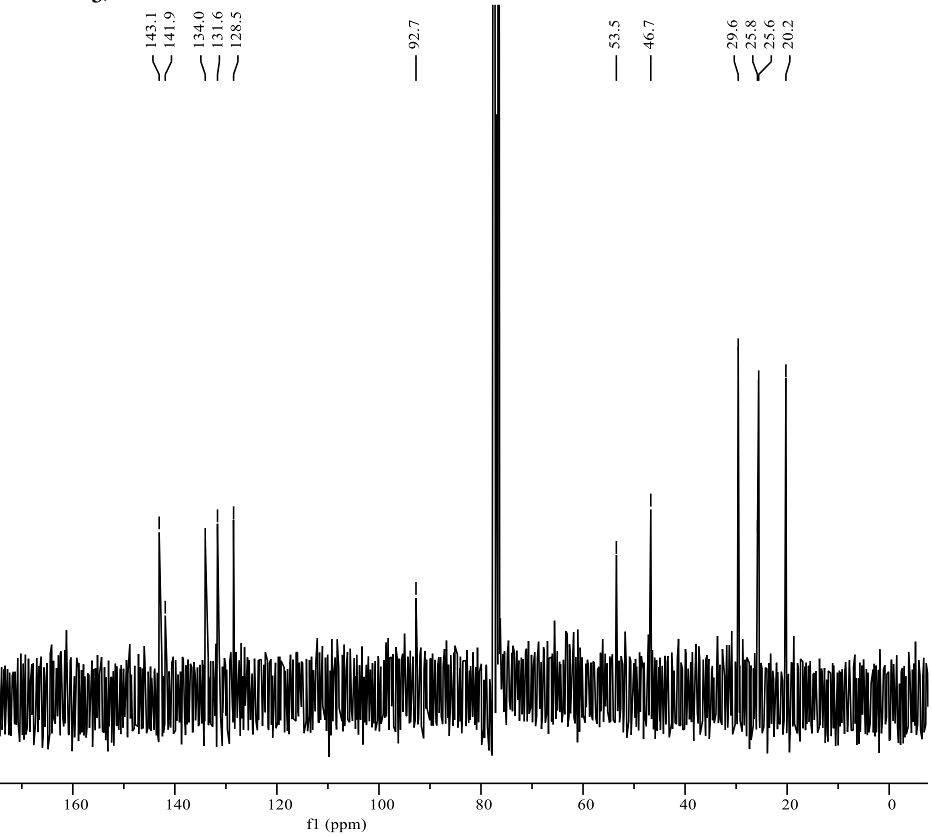

^{1}H NMR (600 MHz, $CDCl_3$):

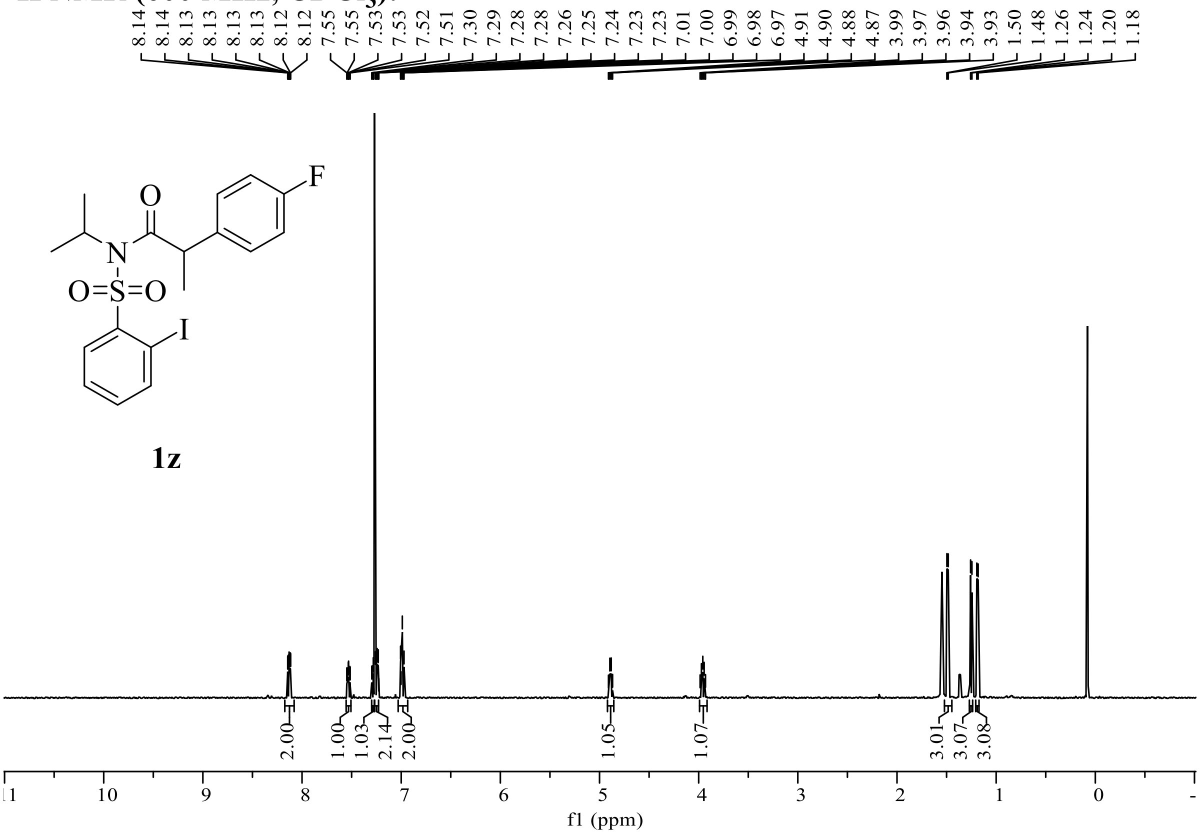

1z

$^{13}C\{^{19}F\}$ NMR (151 MHz, $CDCl_3$):

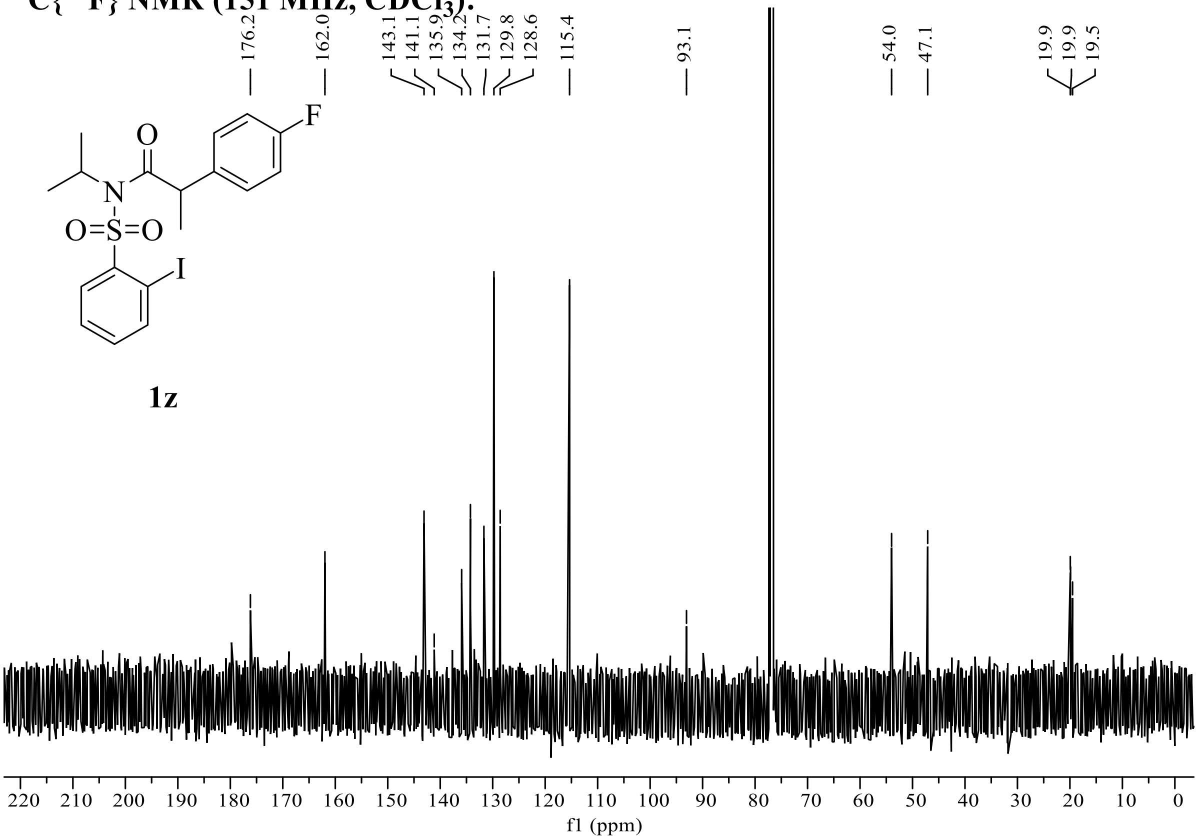

1z

$^{19}F\{^{1}H\}$ NMR (470 MHz, $CDCl_3$):

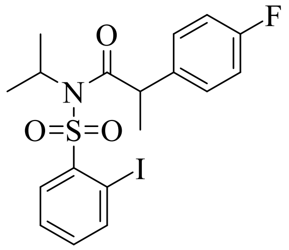

1z

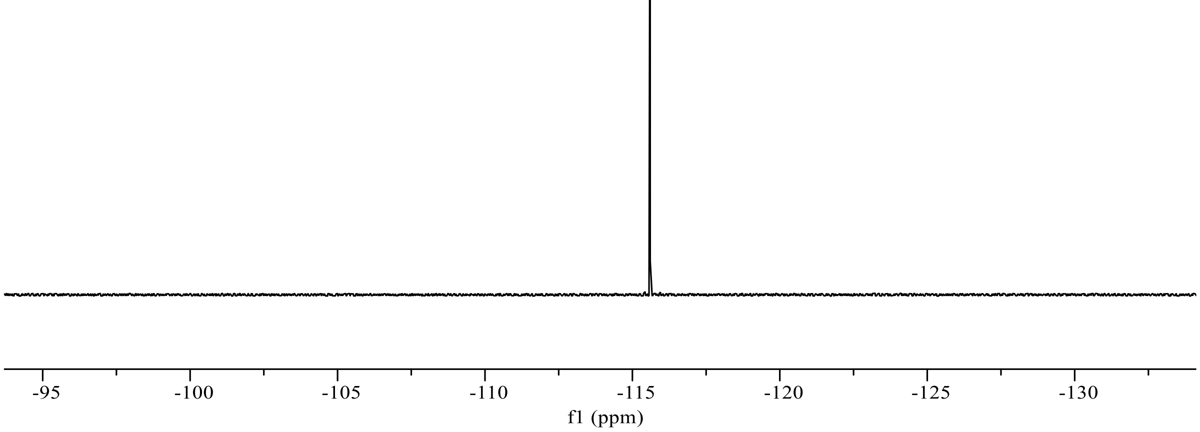

^{1}H NMR (300 MHz, $CDCl_3$):

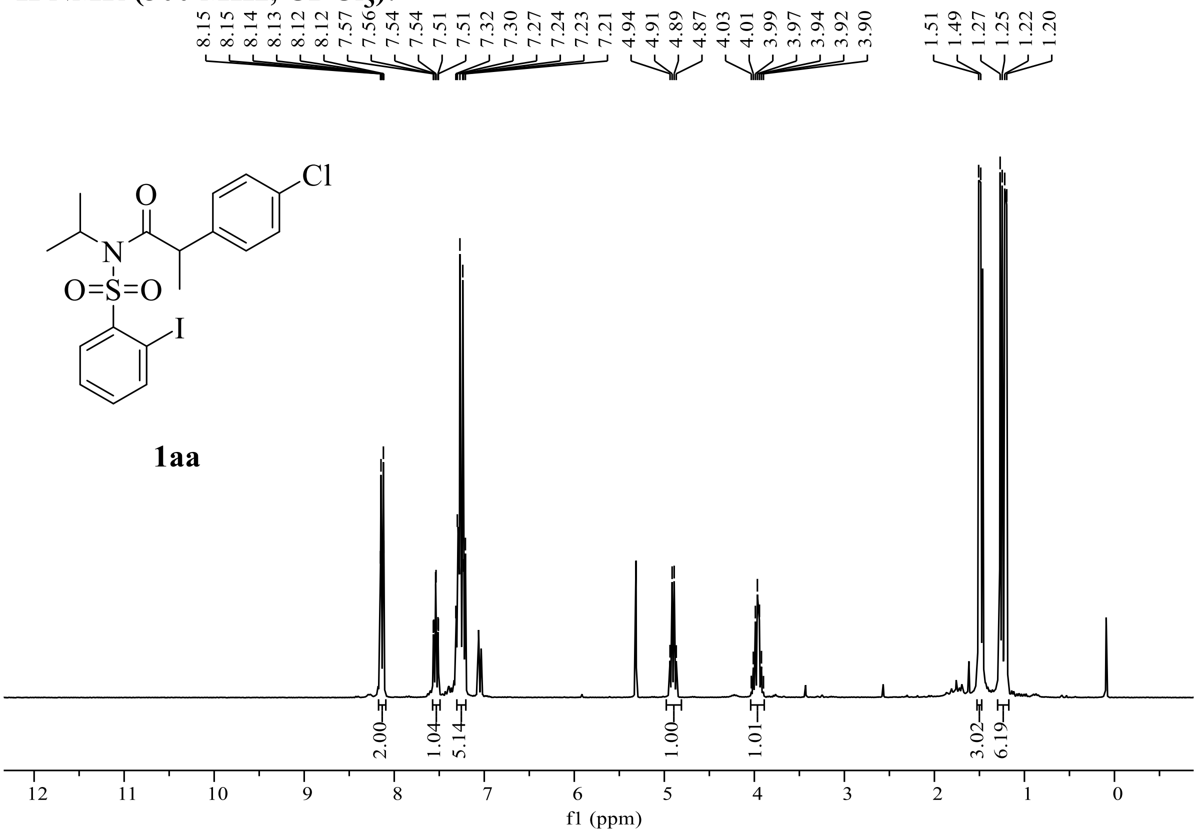

^{13}C NMR (75 MHz, $CDCl_3$):

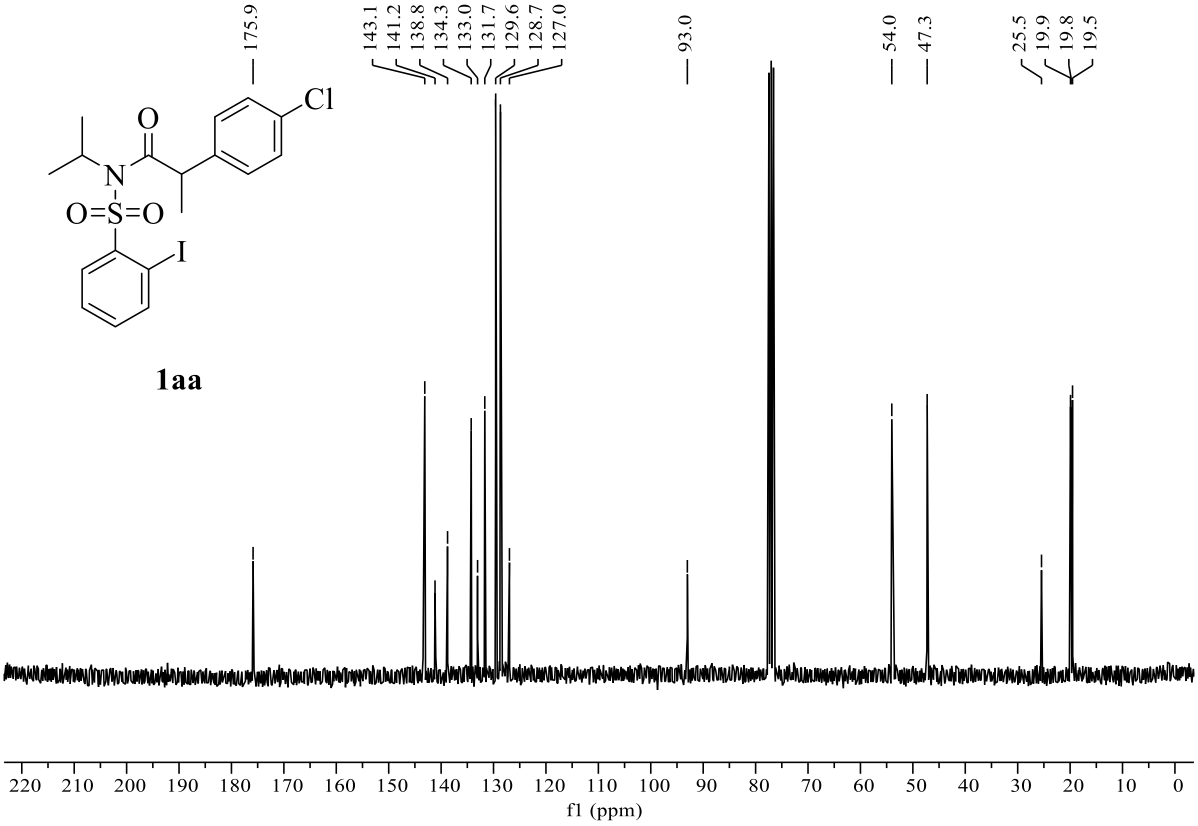

^{1}H NMR (300 MHz, $CDCl_3$):

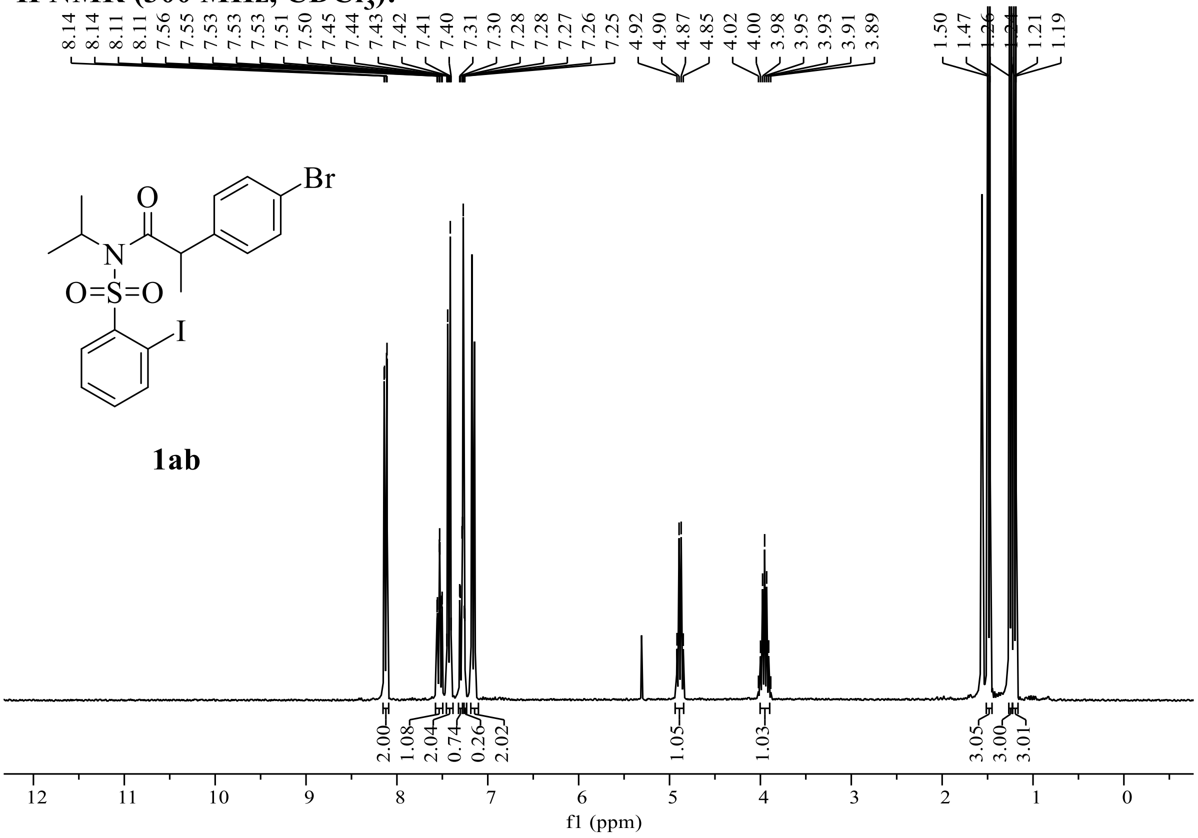

^{13}C NMR (75 MHz, $CDCl_3$):

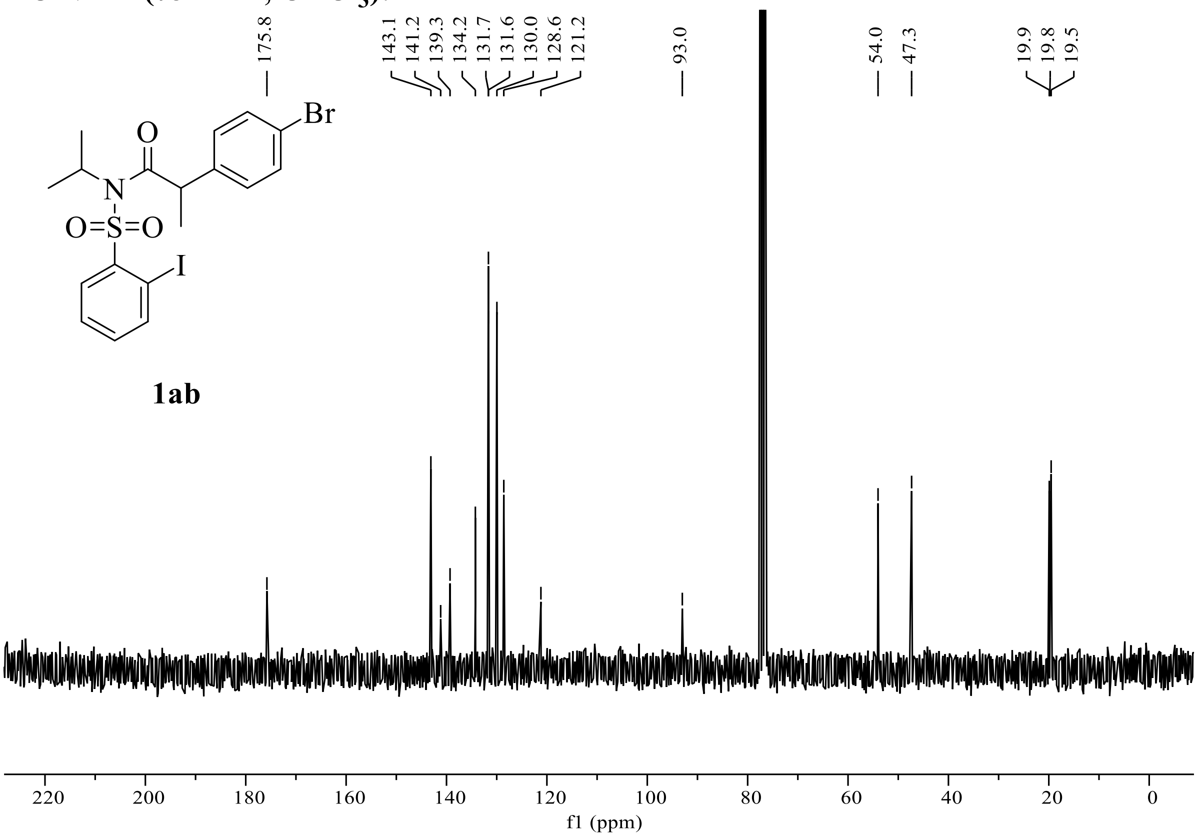

^{1}H NMR (600 MHz, $CDCl_3$):

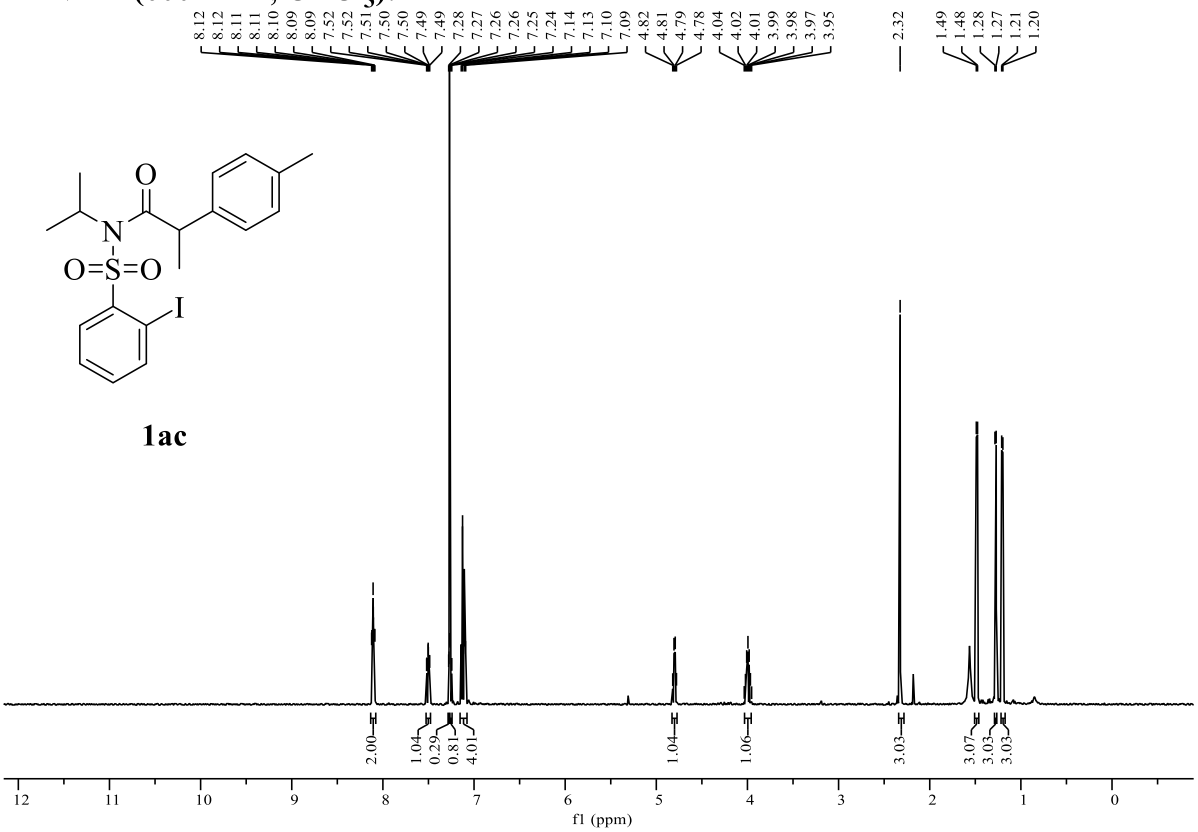

^{13}C NMR (151 MHz, $CDCl_3$):

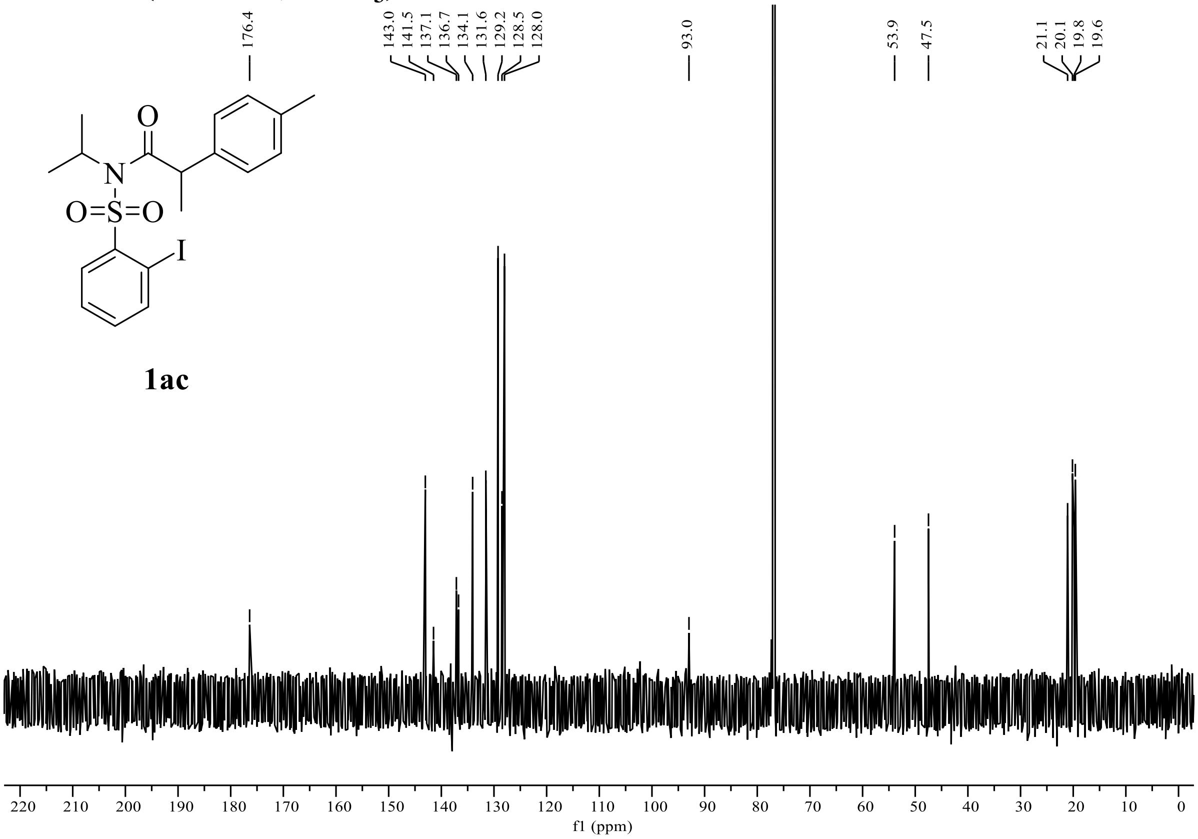

^{1}H NMR (300 MHz, $CDCl_3$):

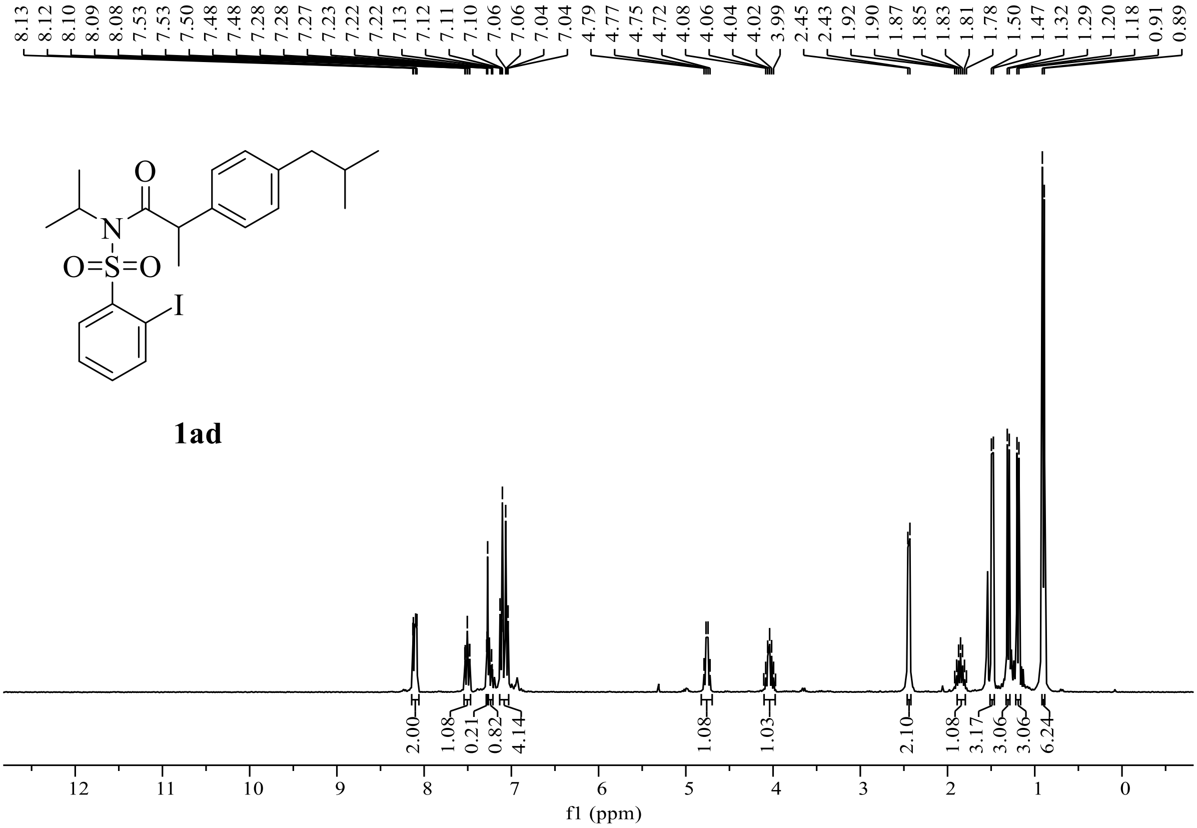

^{13}C NMR (75 MHz, $CDCl_3$):

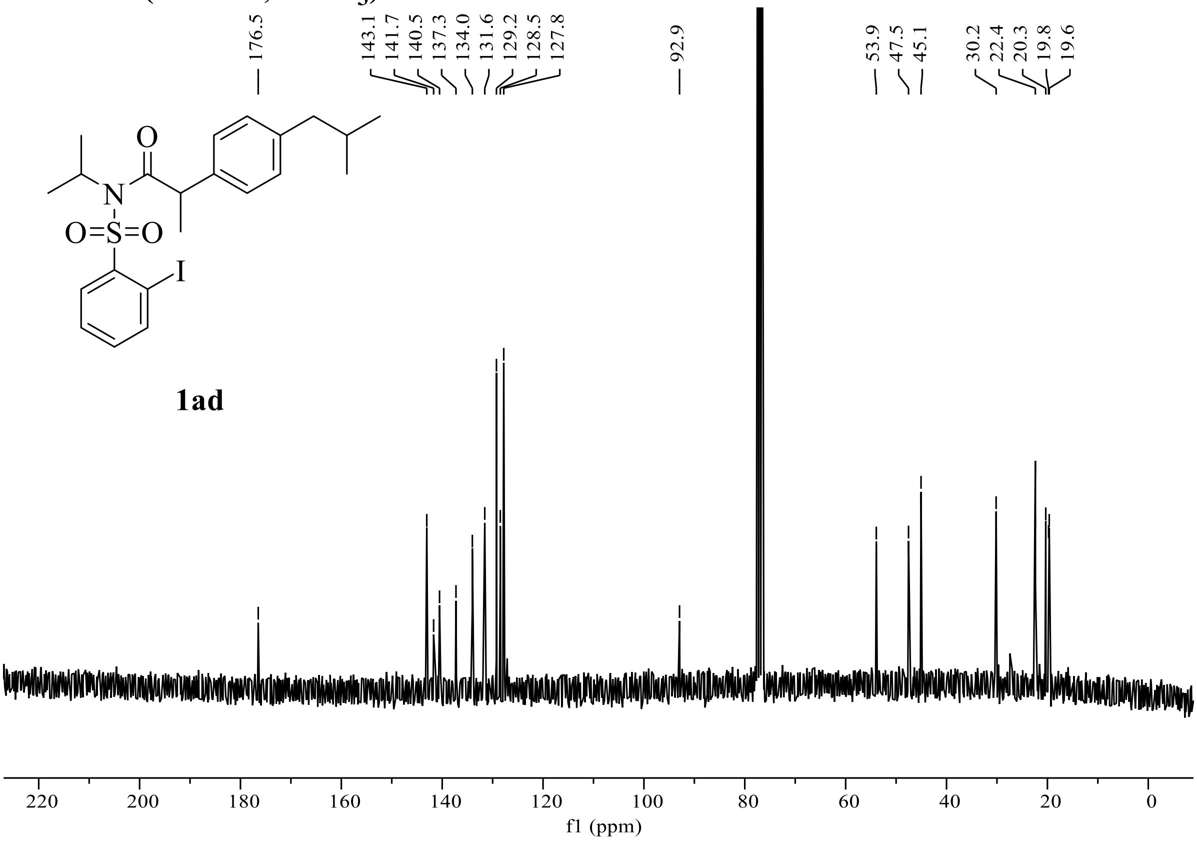

^{1}H NMR (300 MHz, $CDCl_3$):

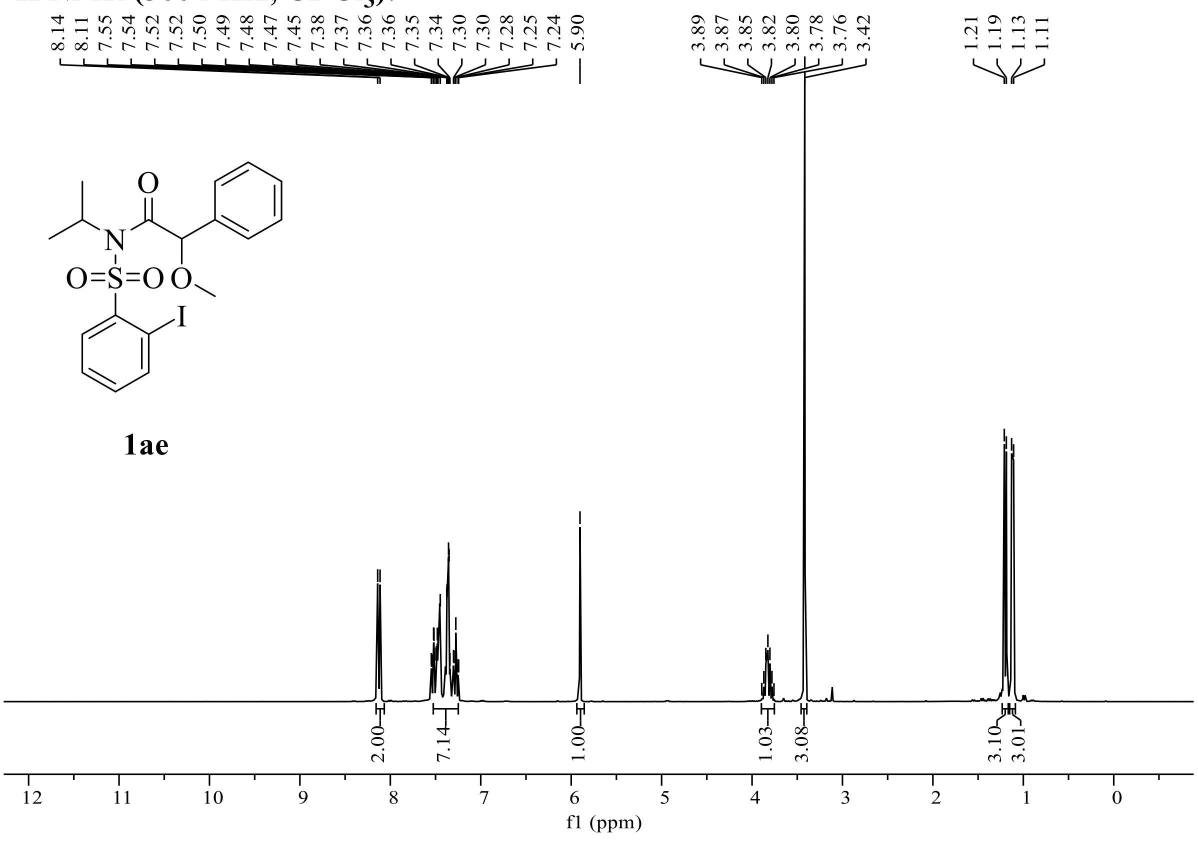

^{13}C NMR (75 MHz, $CDCl_3$):

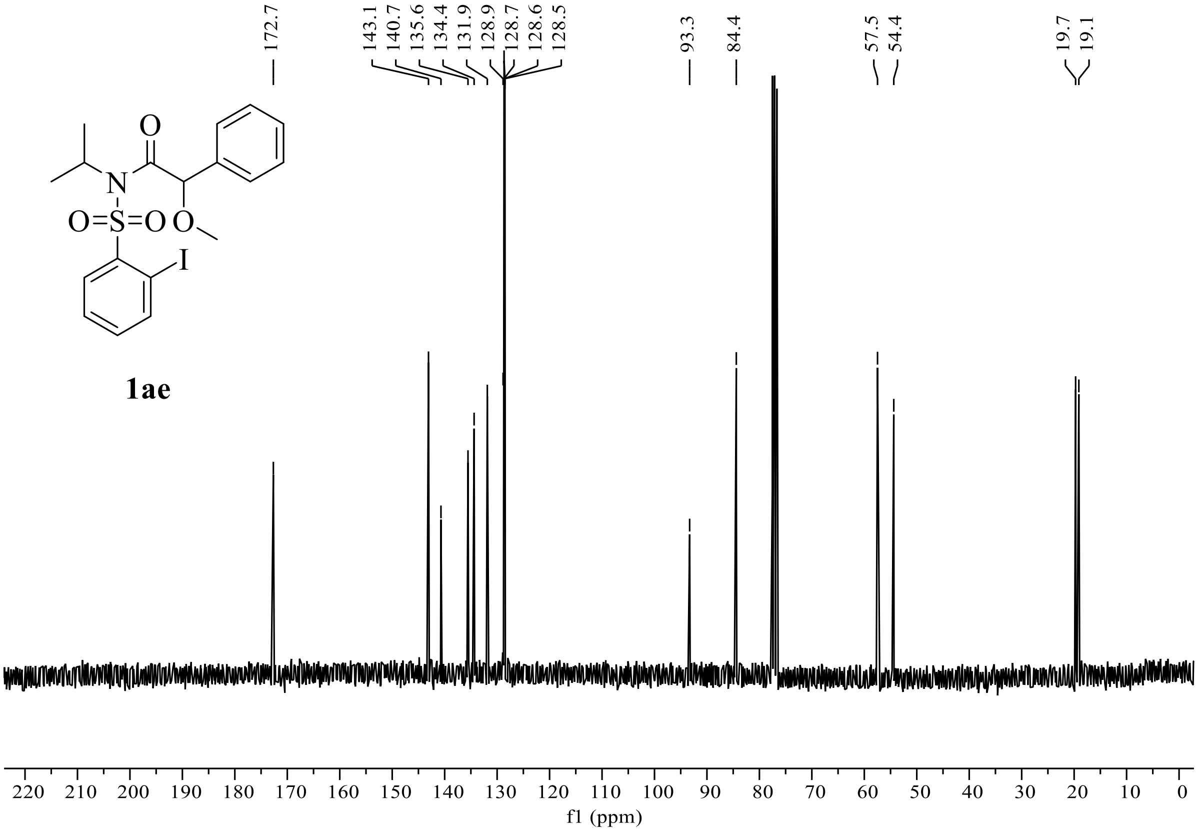

^{1}H NMR (600 MHz, $CDCl_3$):

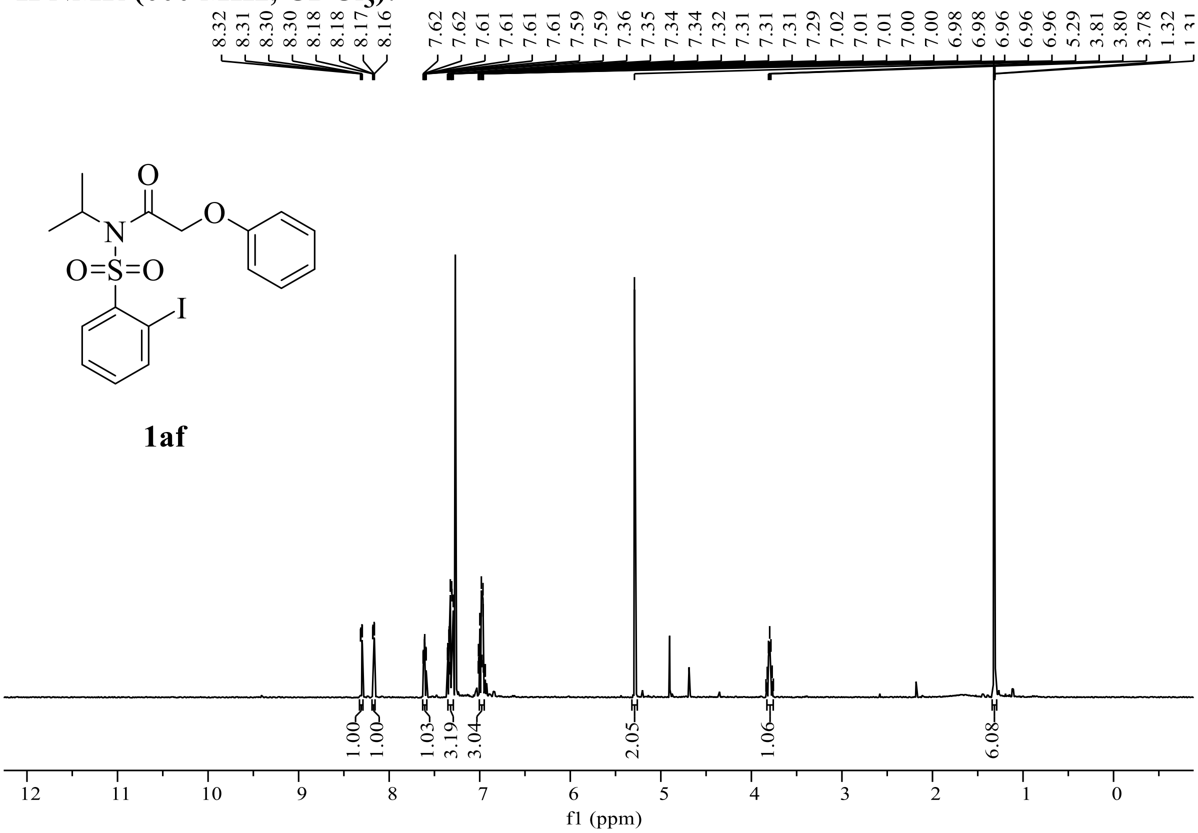

1af

^{13}C NMR (151 MHz, $CDCl_3$):

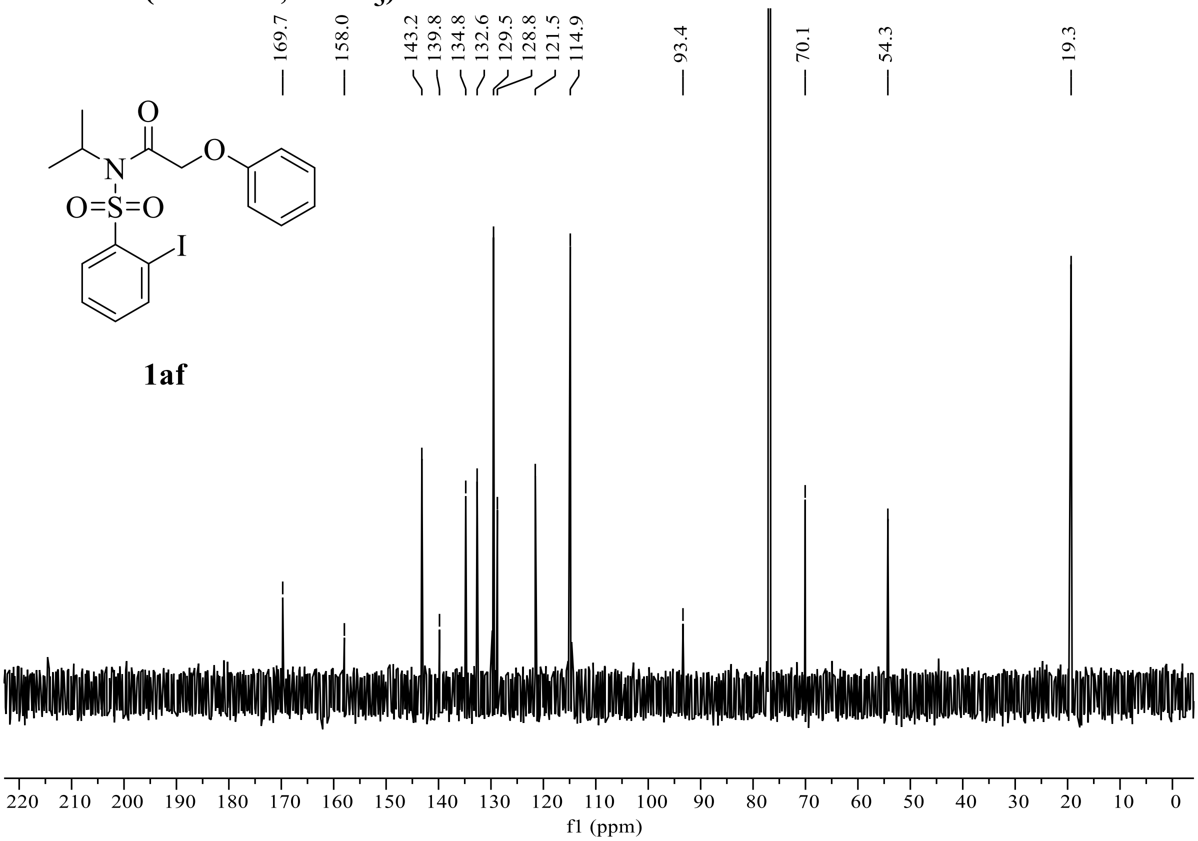

1af

^{1}H NMR (300 MHz, $CDCl_3$):

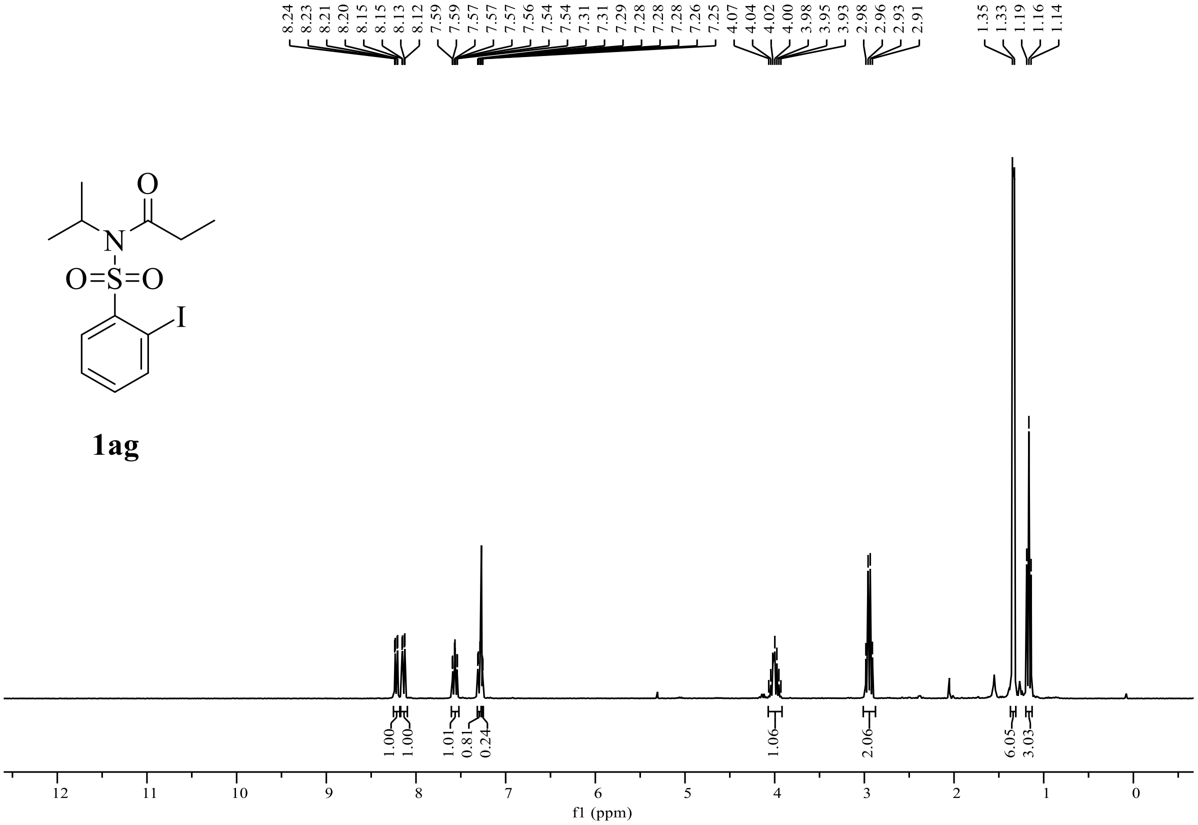

1ag

^{13}C NMR (75 MHz, $CDCl_3$):

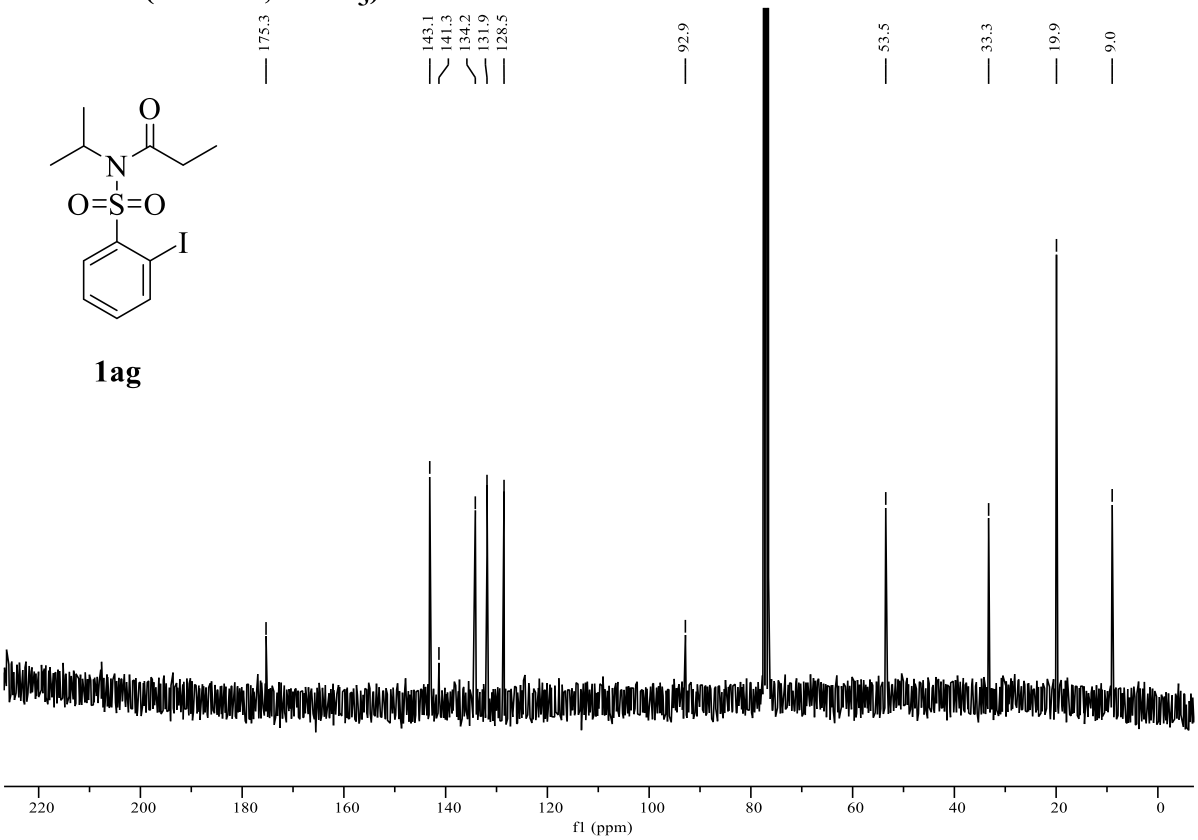

1ag

^{1}H NMR (300 MHz, $CDCl_3$):

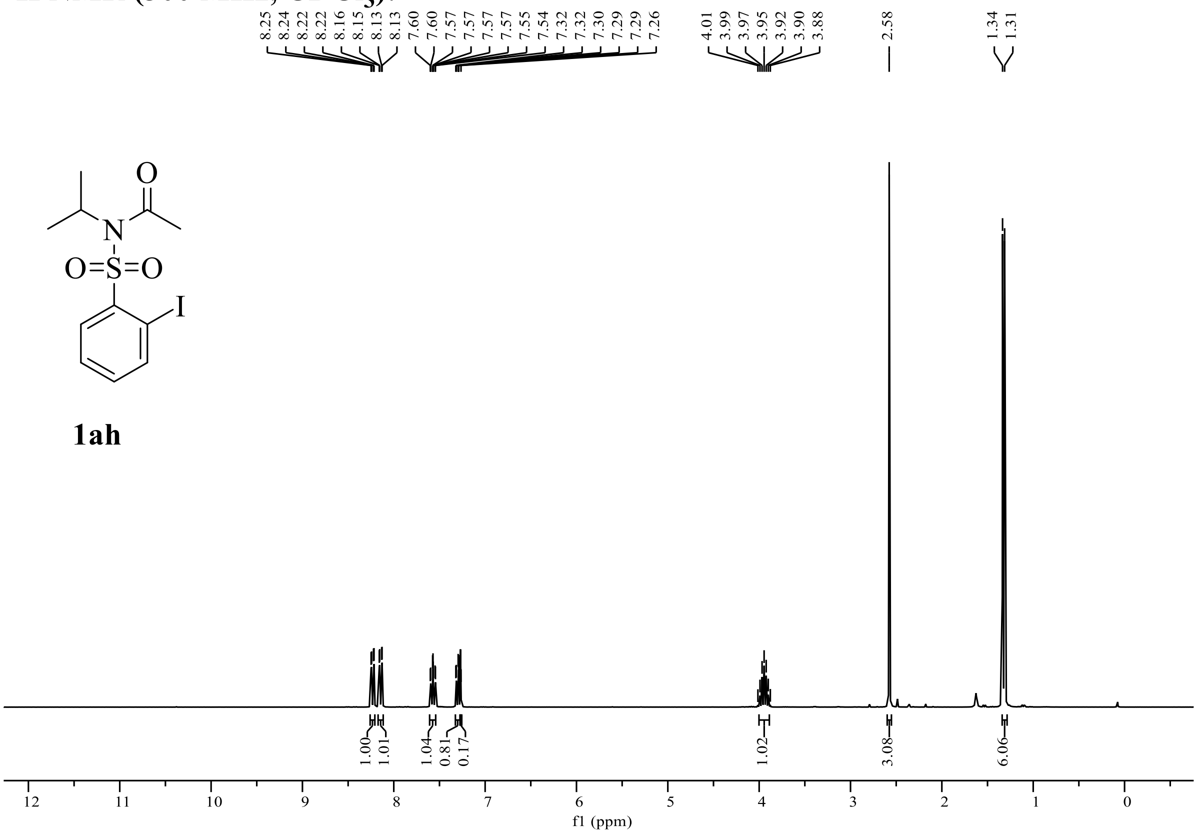

^{13}C NMR (75 MHz, $CDCl_3$):

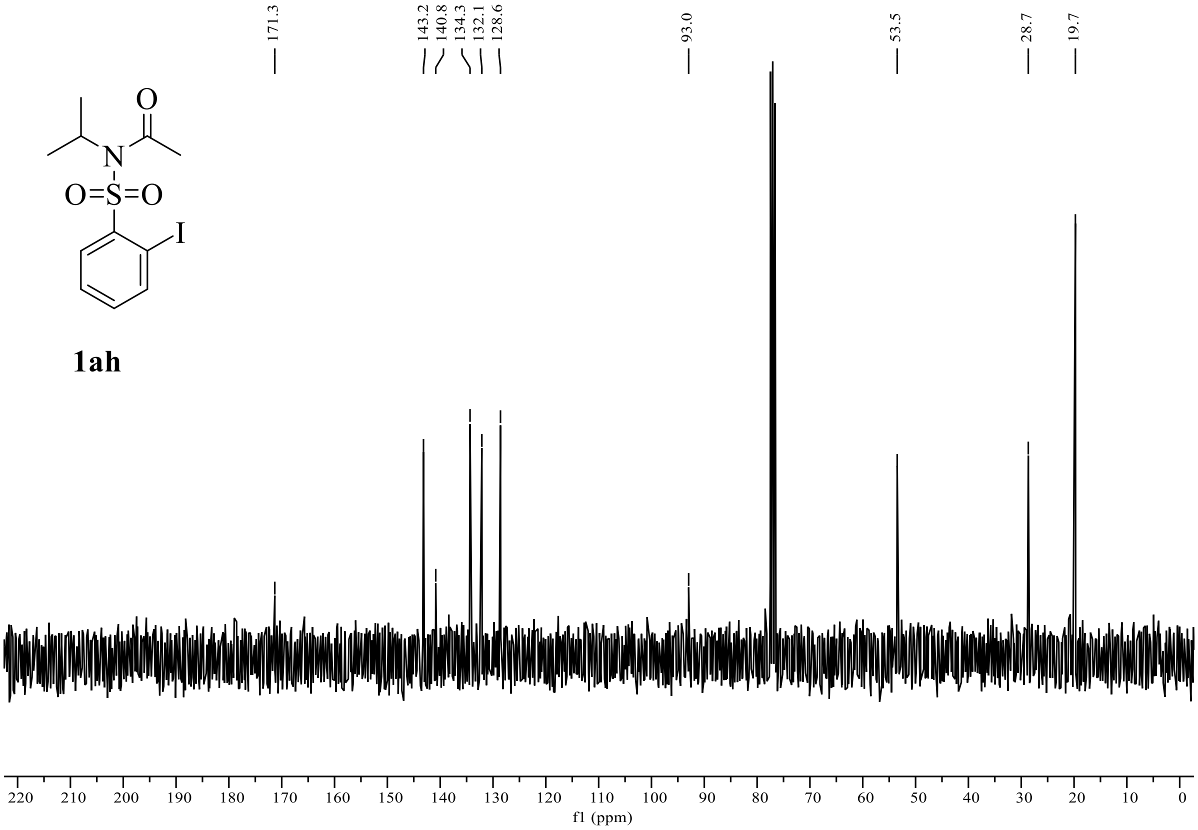

^{1}H NMR (300 MHz, $CDCl_3$):

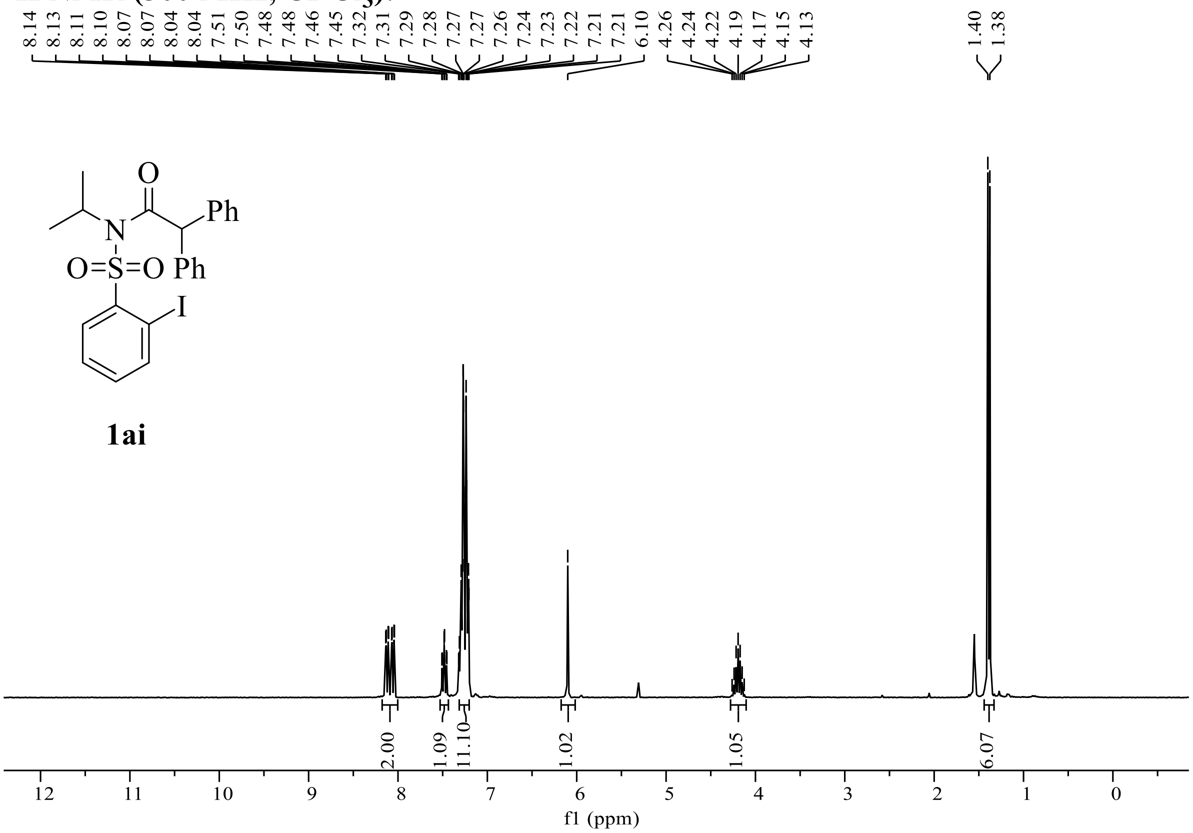

^{13}C NMR (75 MHz, $CDCl_3$):

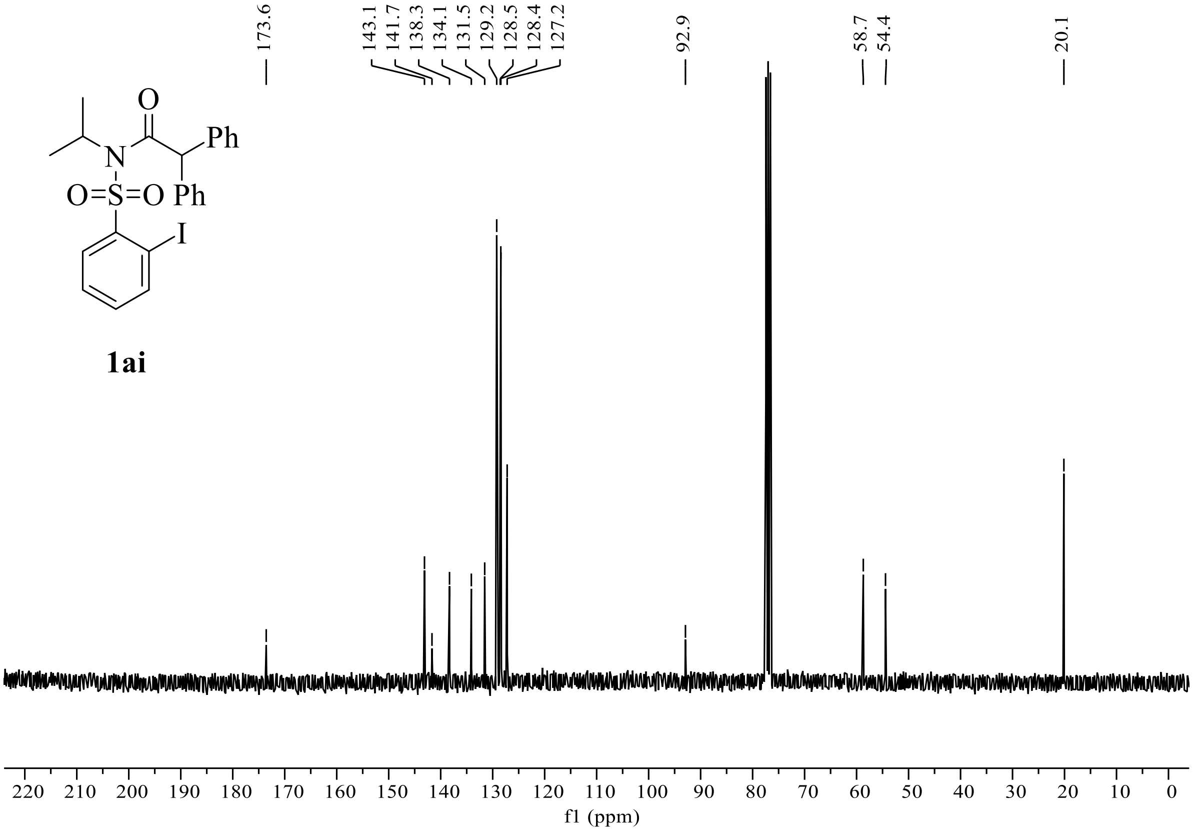

^{1}H NMR (600 MHz, $CDCl_3$):

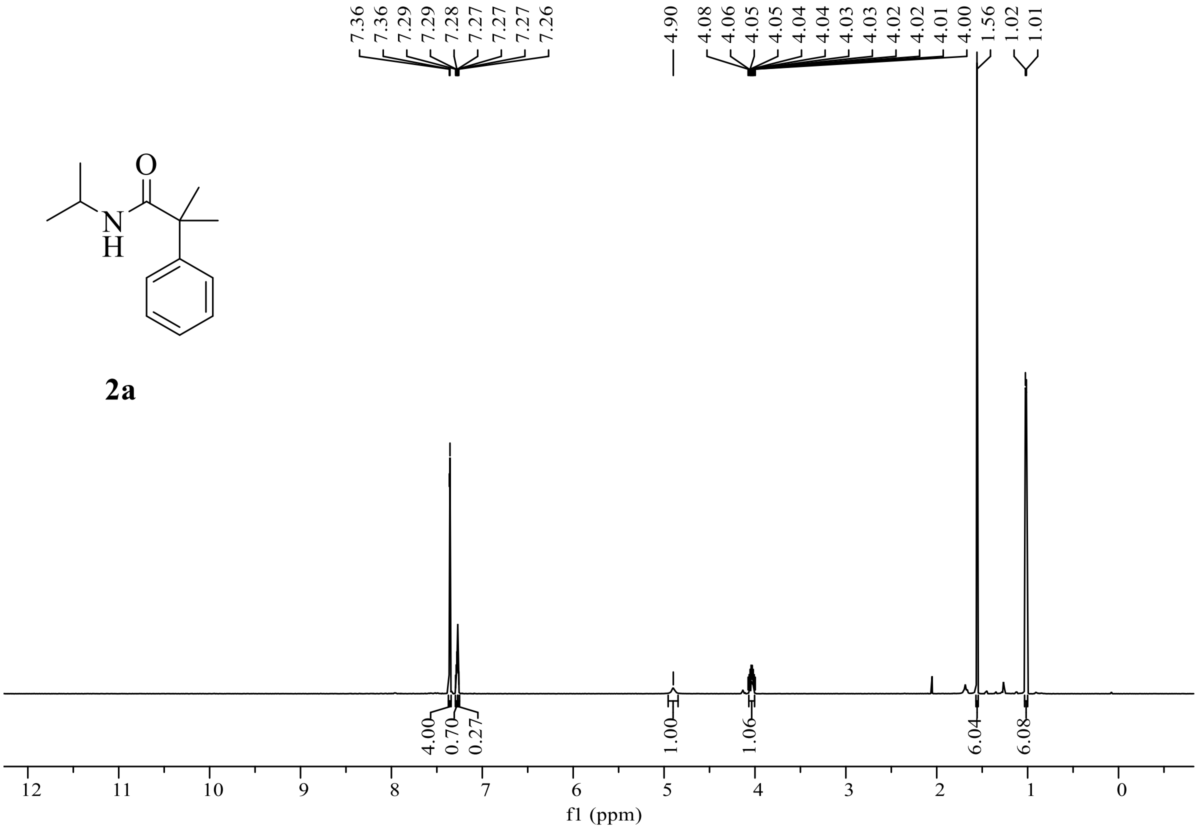

^{13}C NMR (151 MHz, $CDCl_3$):

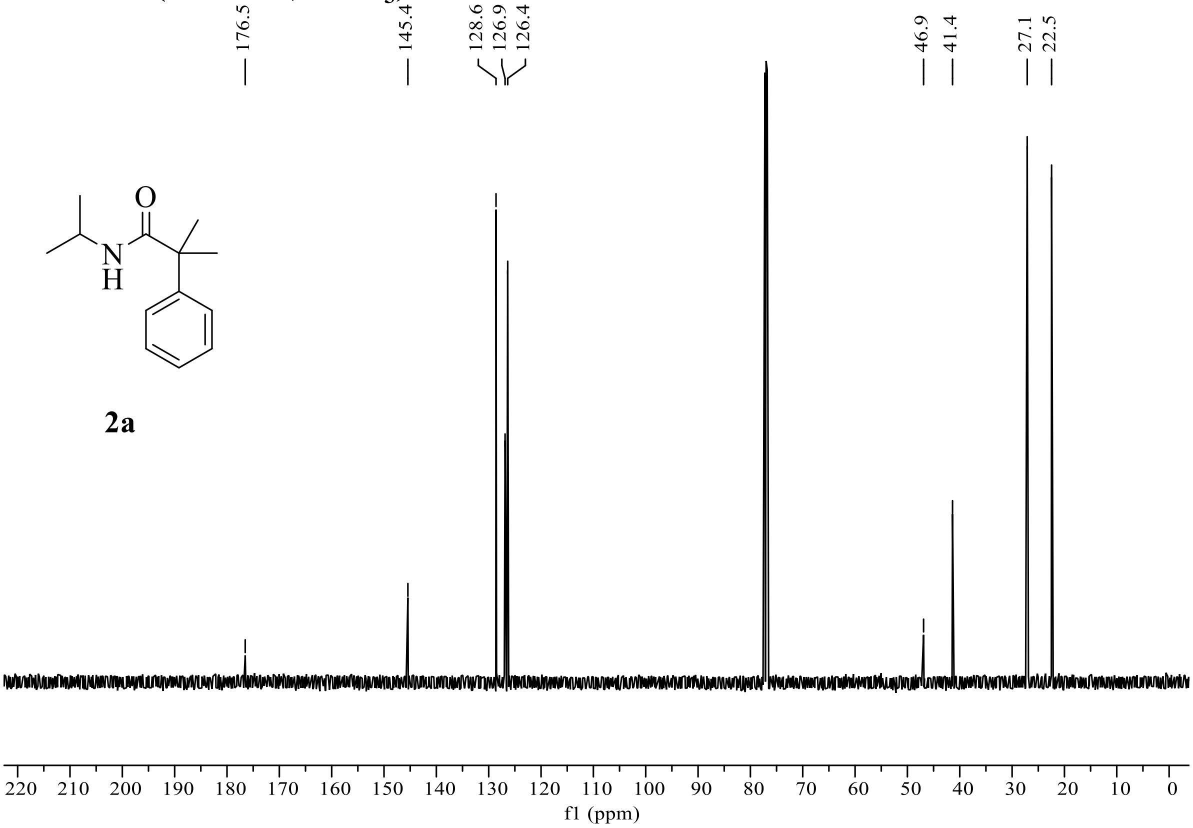

^{1}H NMR (300 MHz, $CDCl_3$):

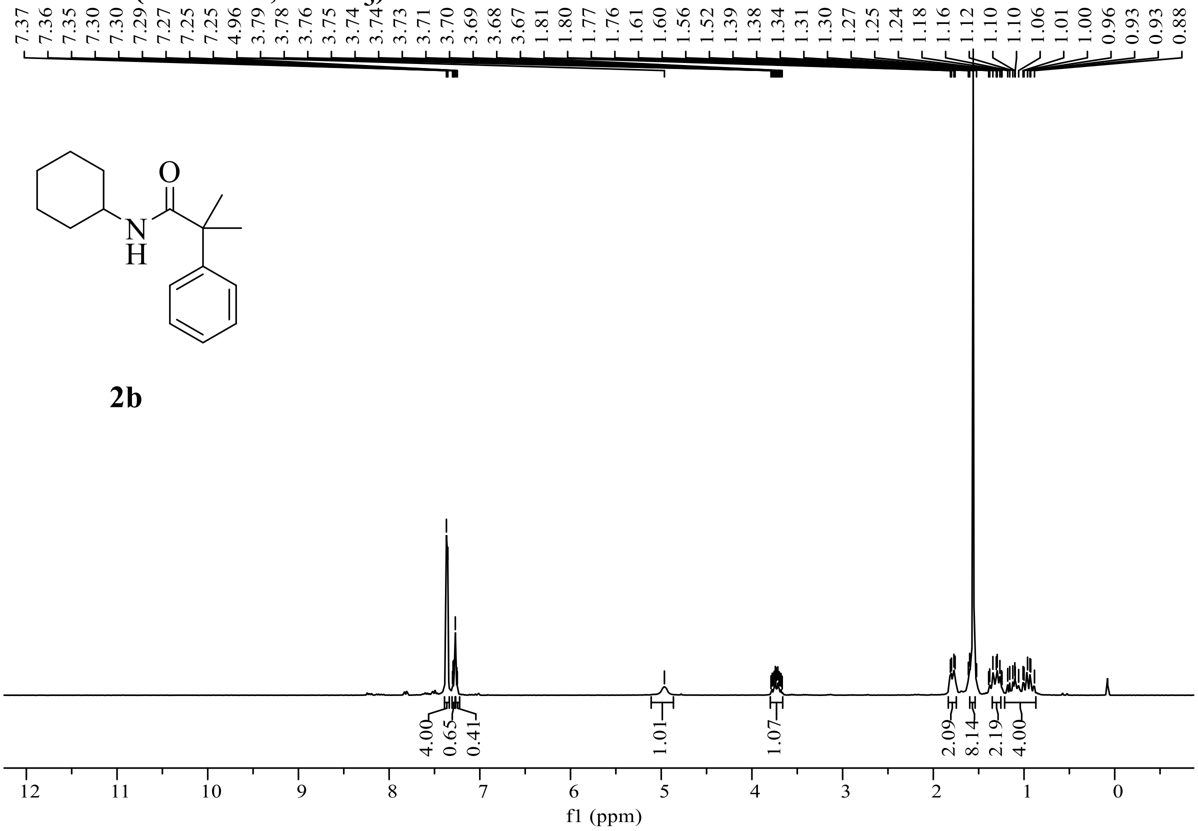

^{13}C NMR (75 MHz, $CDCl_3$):

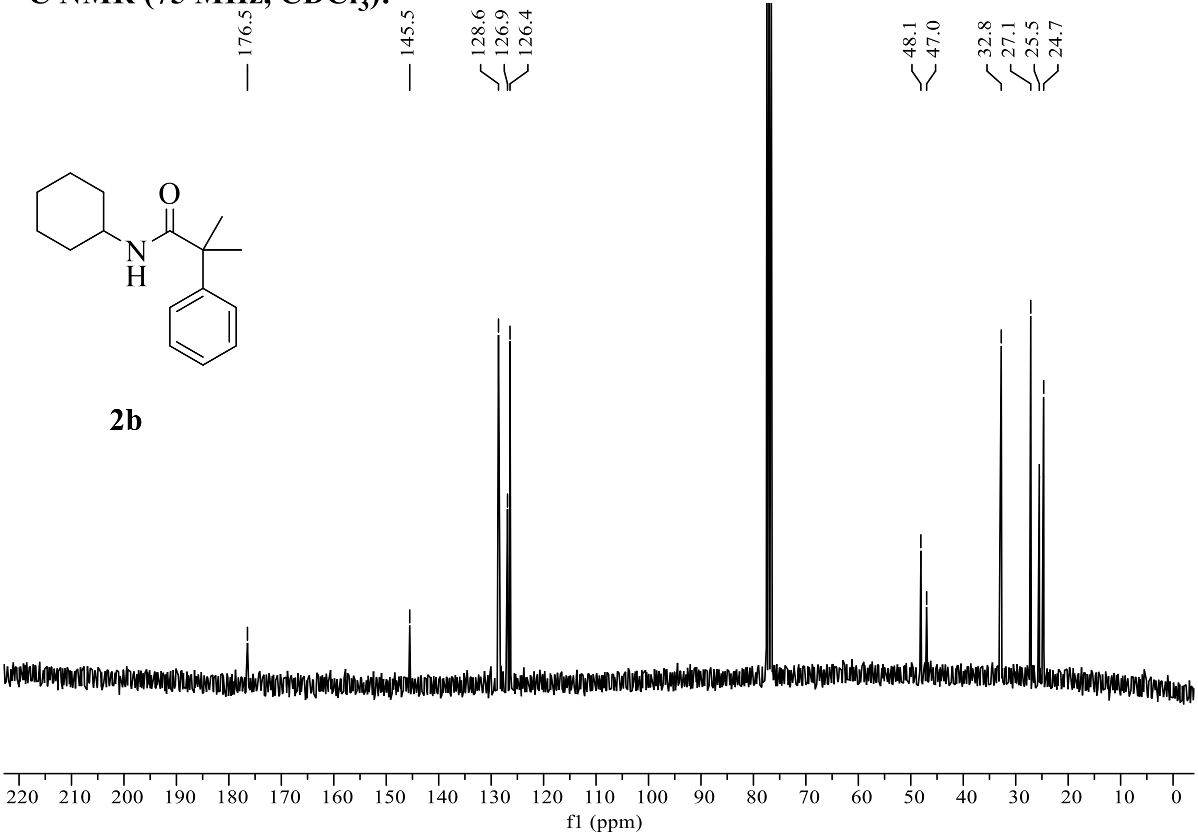

^{1}H NMR (300 MHz, $CDCl_3$):

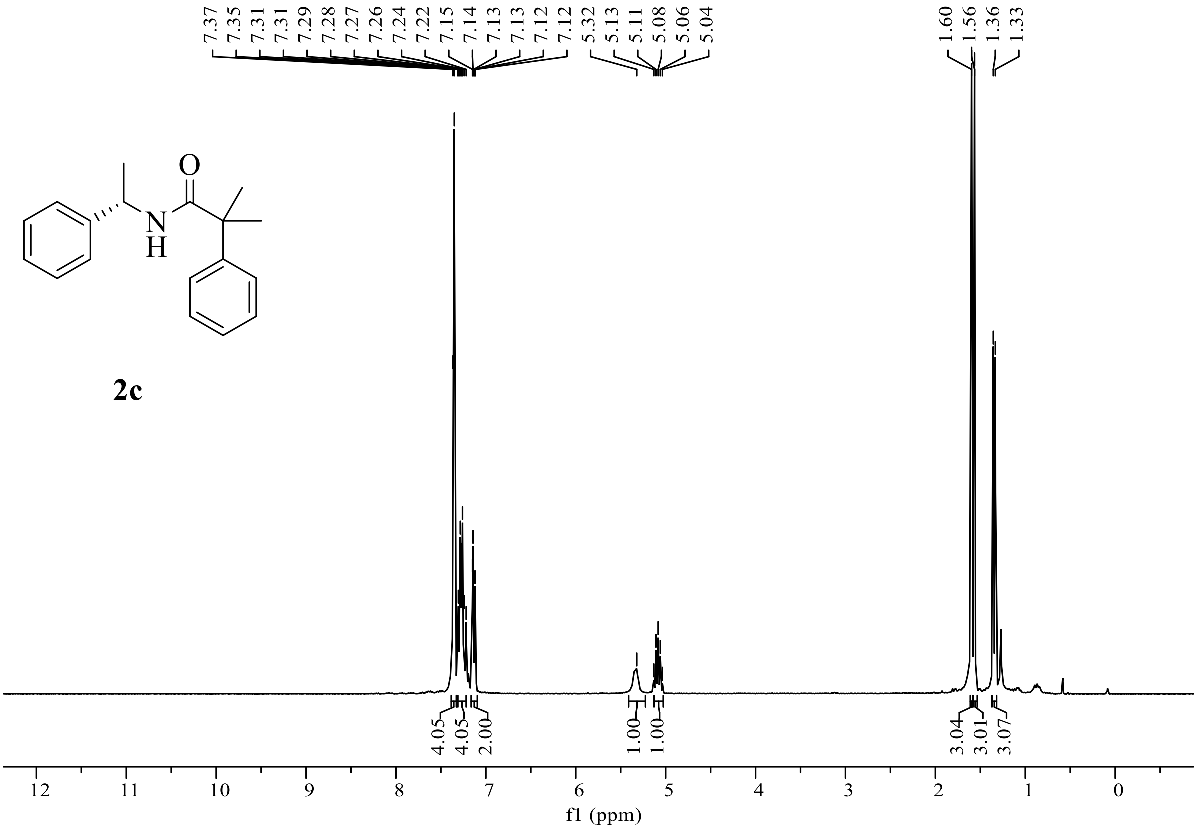

^{13}C NMR (75 MHz, $CDCl_3$):

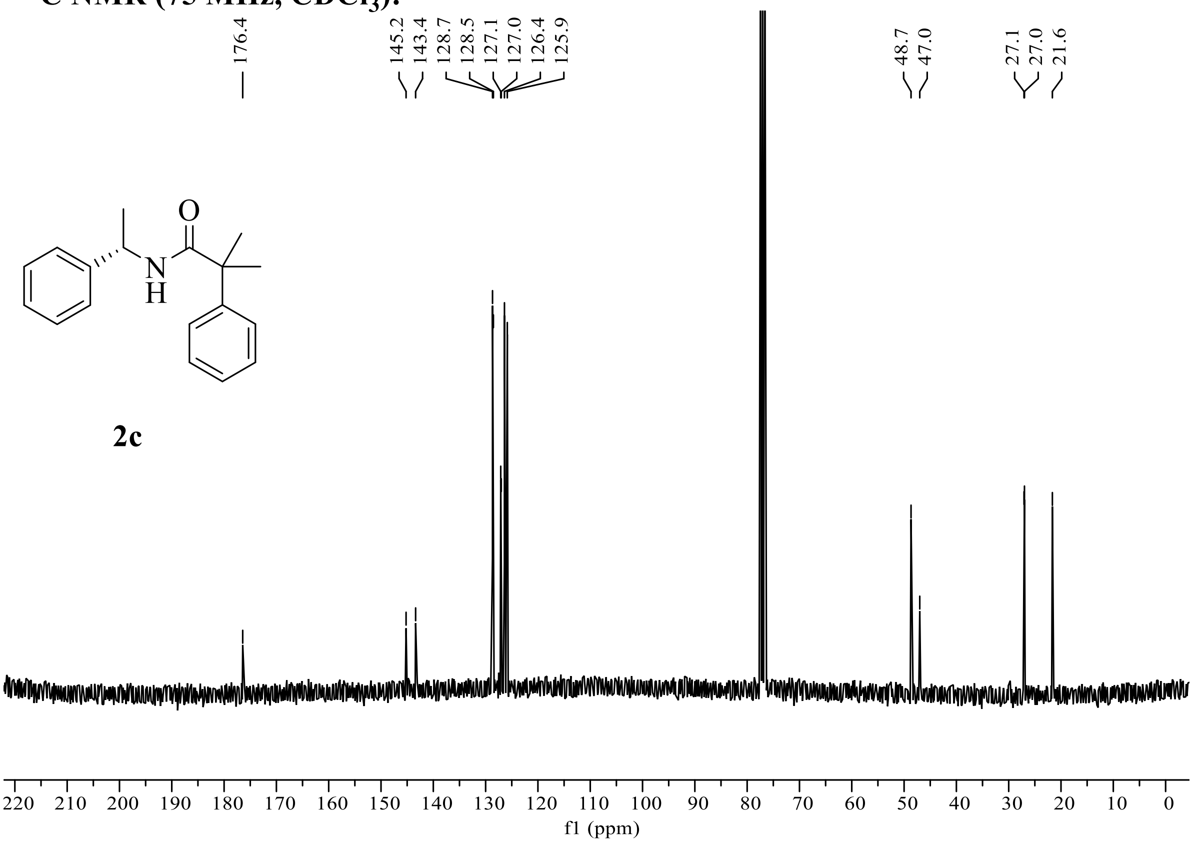

^{1}H NMR (600 MHz, $CDCl_3$):

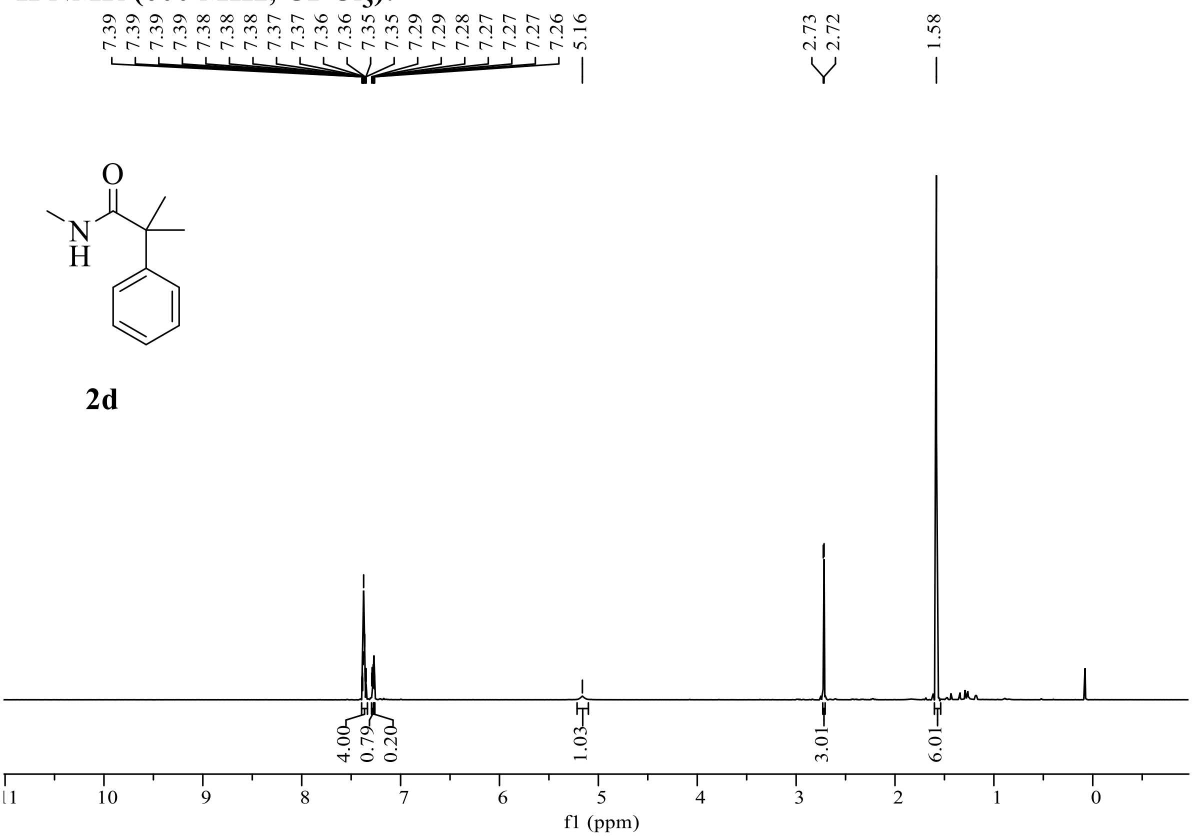

^{13}C NMR (151 MHz, $CDCl_3$):

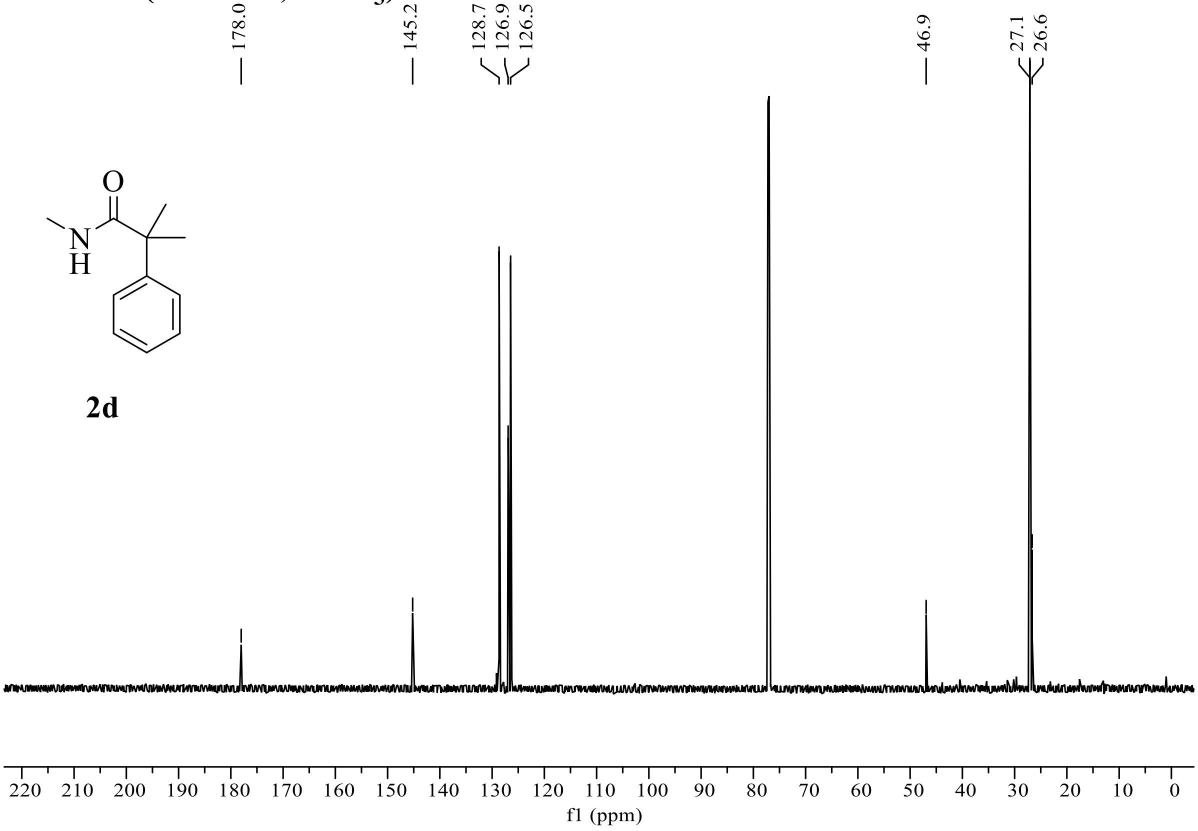

^{1}H NMR (600 MHz, $CDCl_3$):

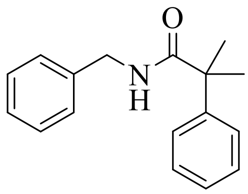

2e

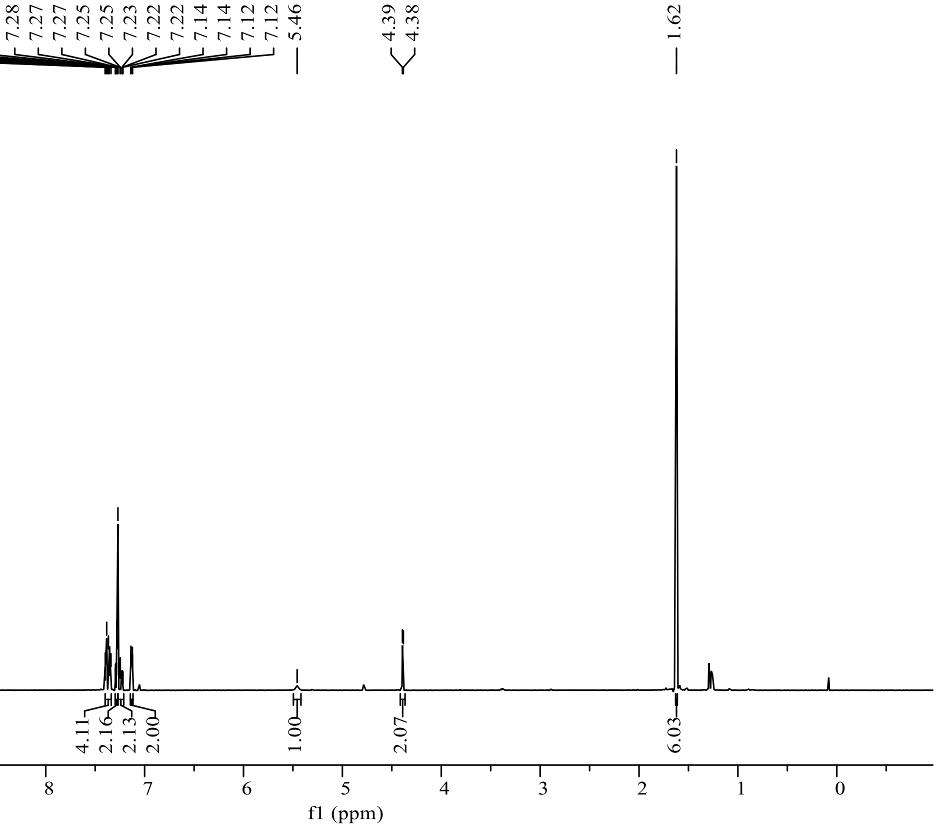

^{13}C NMR (151 MHz, $CDCl_3$):

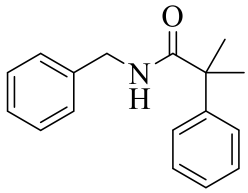

2e

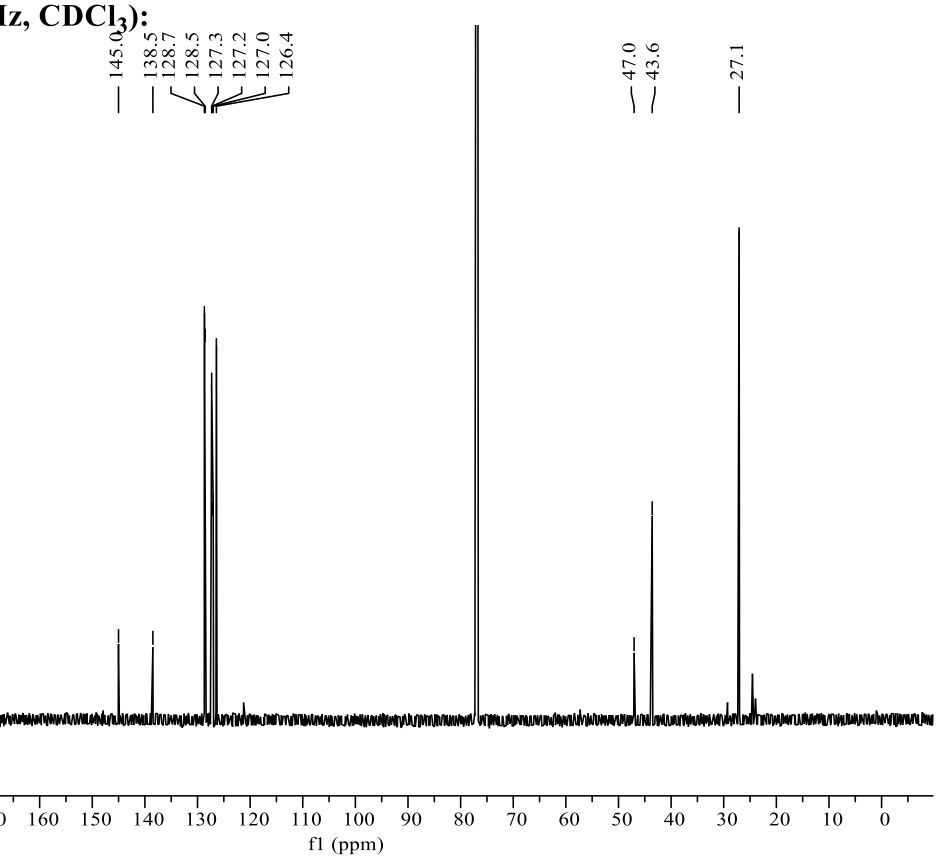

^{1}H NMR (600 MHz, $CDCl_3$):

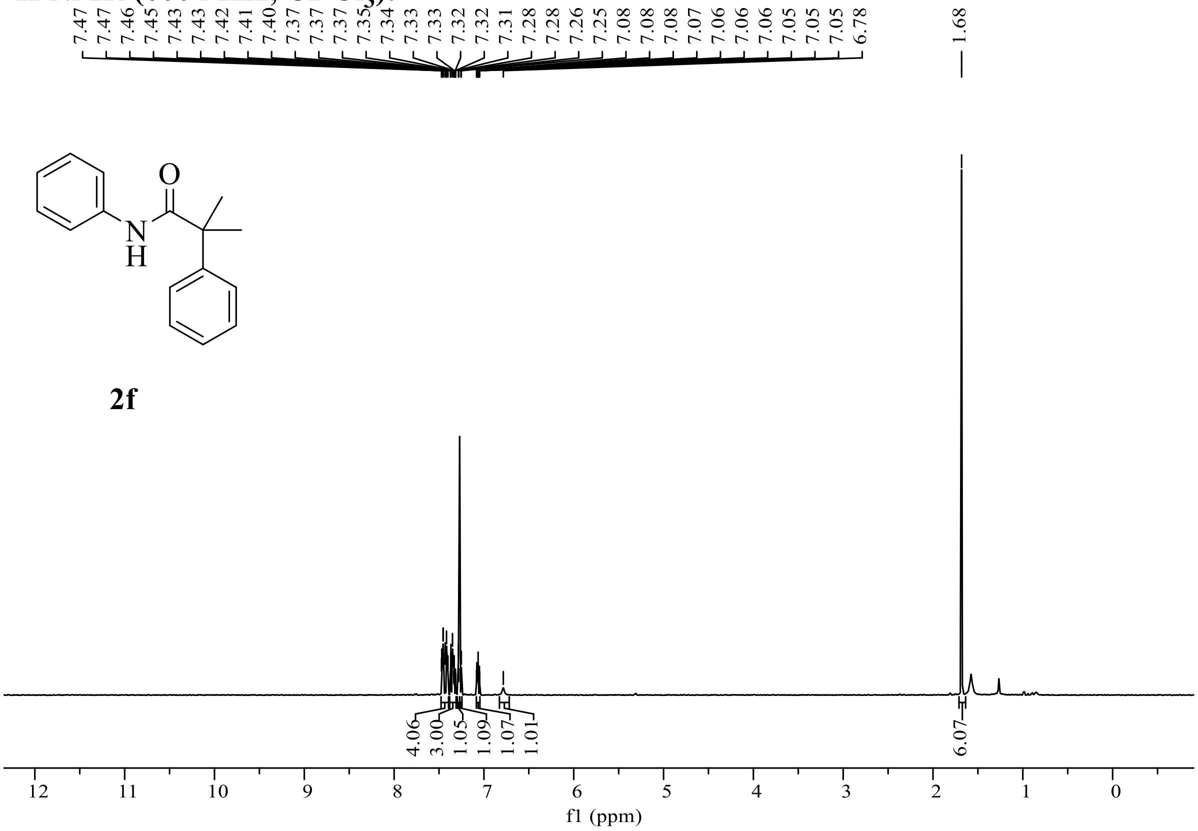

2f

^{13}C NMR (151 MHz, $CDCl_3$):

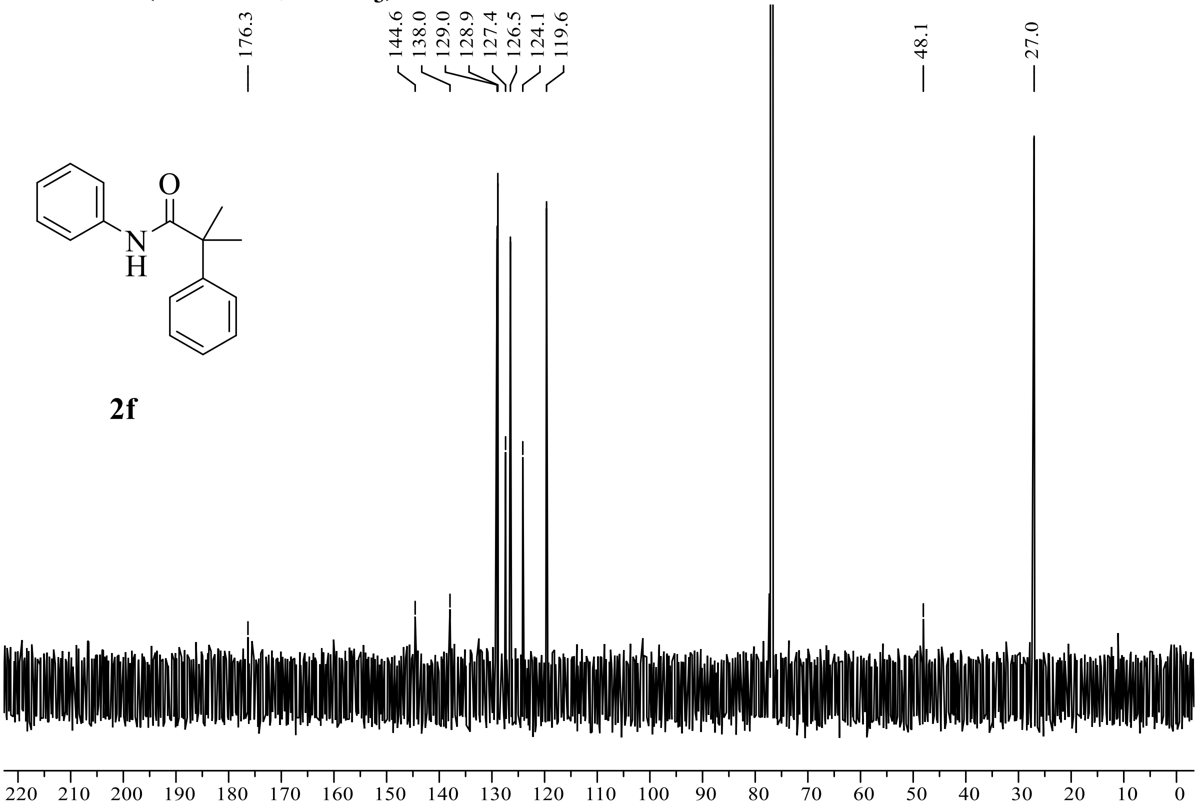

2f

^{1}H NMR (300 MHz, $CDCl_3$):

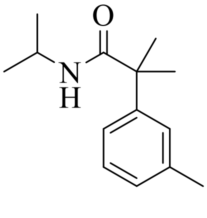

2i

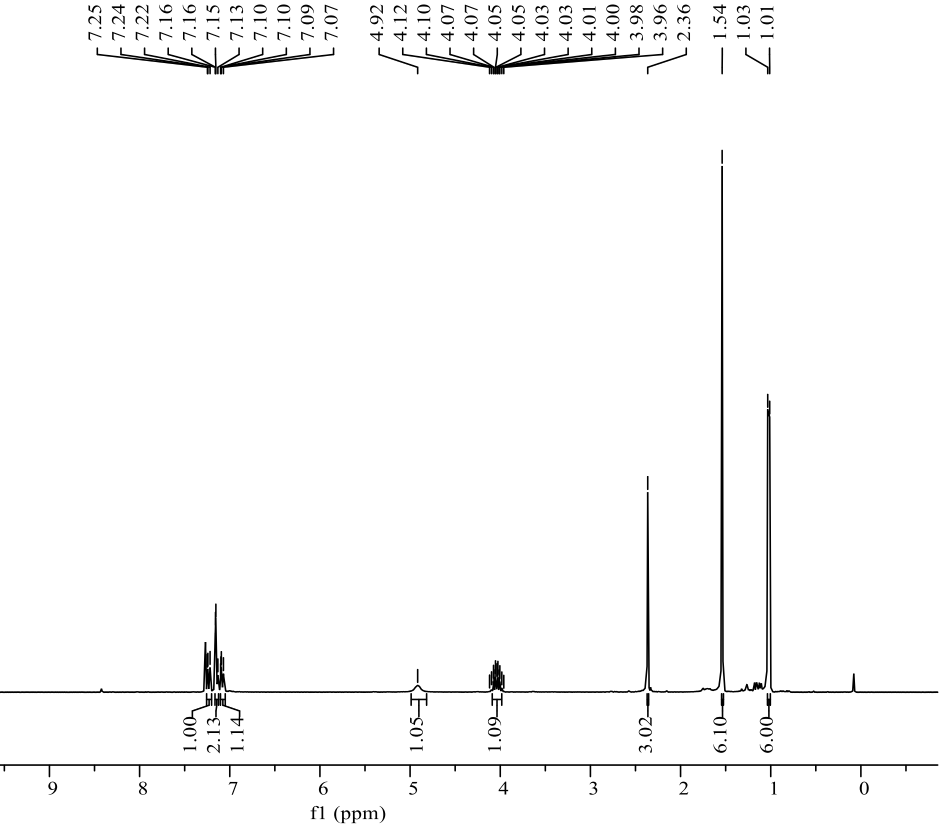

^{13}C NMR (75 MHz, $CDCl_3$):

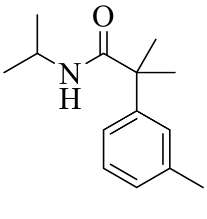

2i

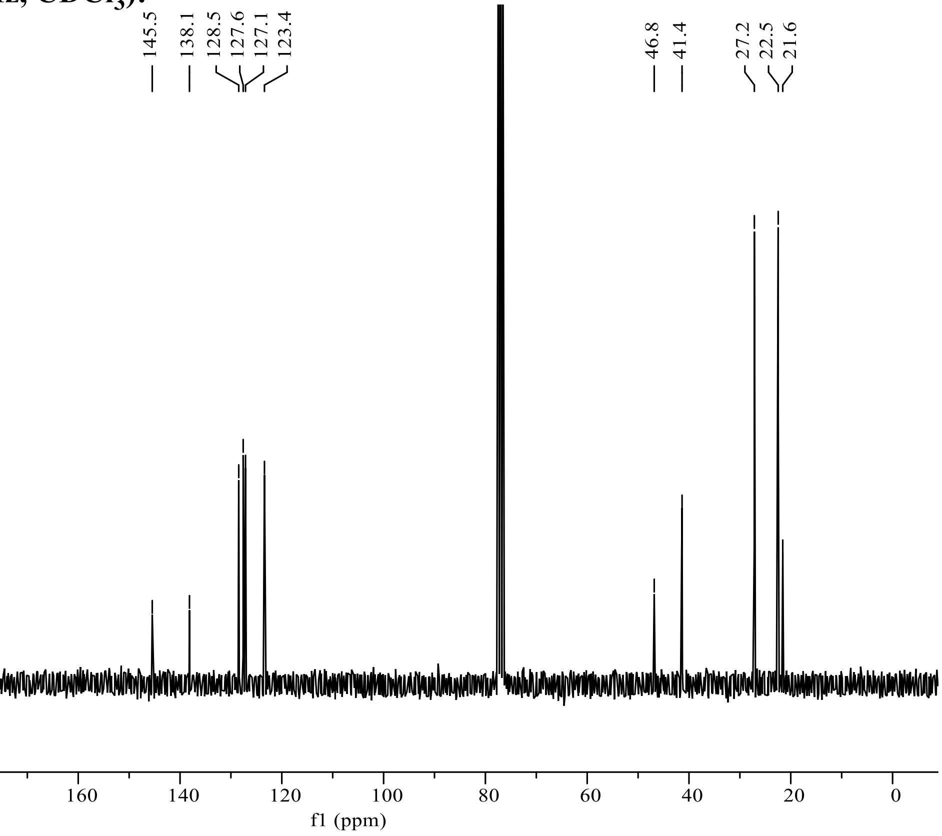

^{1}H NMR (300 MHz, $CDCl_3$):

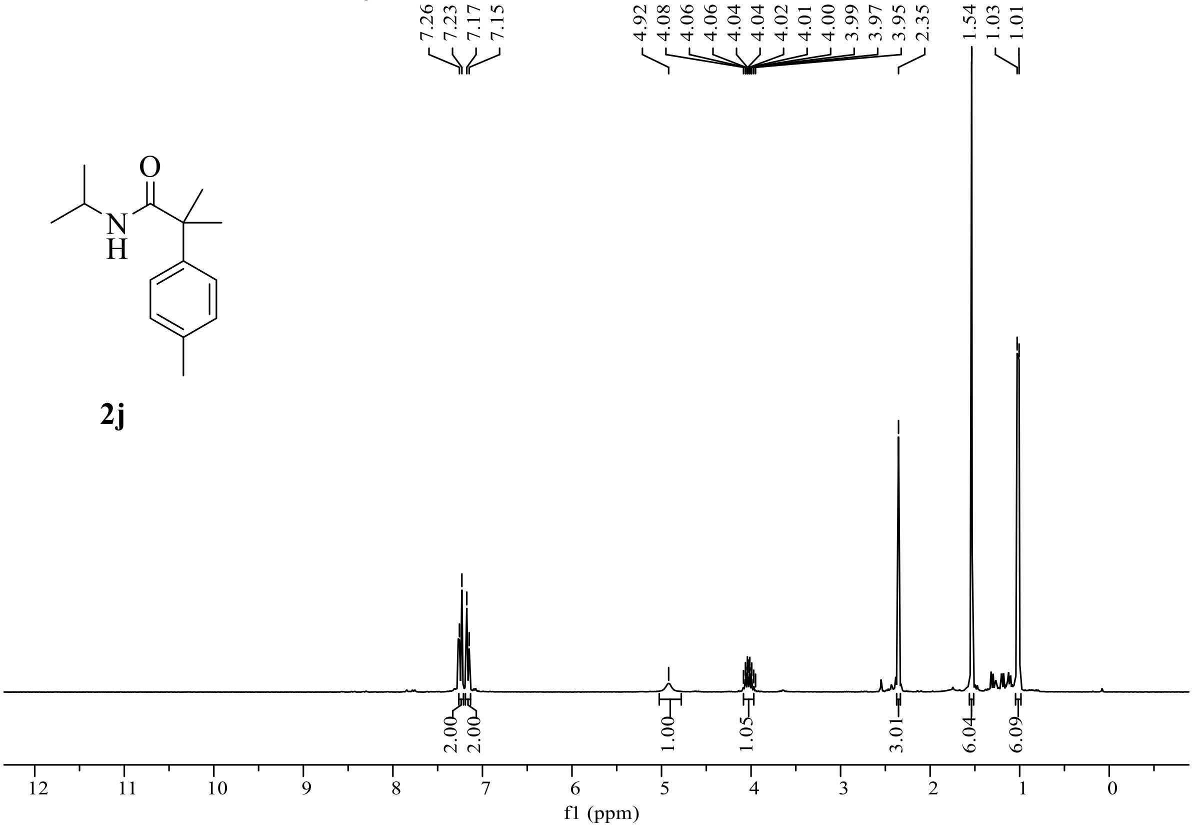

^{13}C NMR (75 MHz, $CDCl_3$):

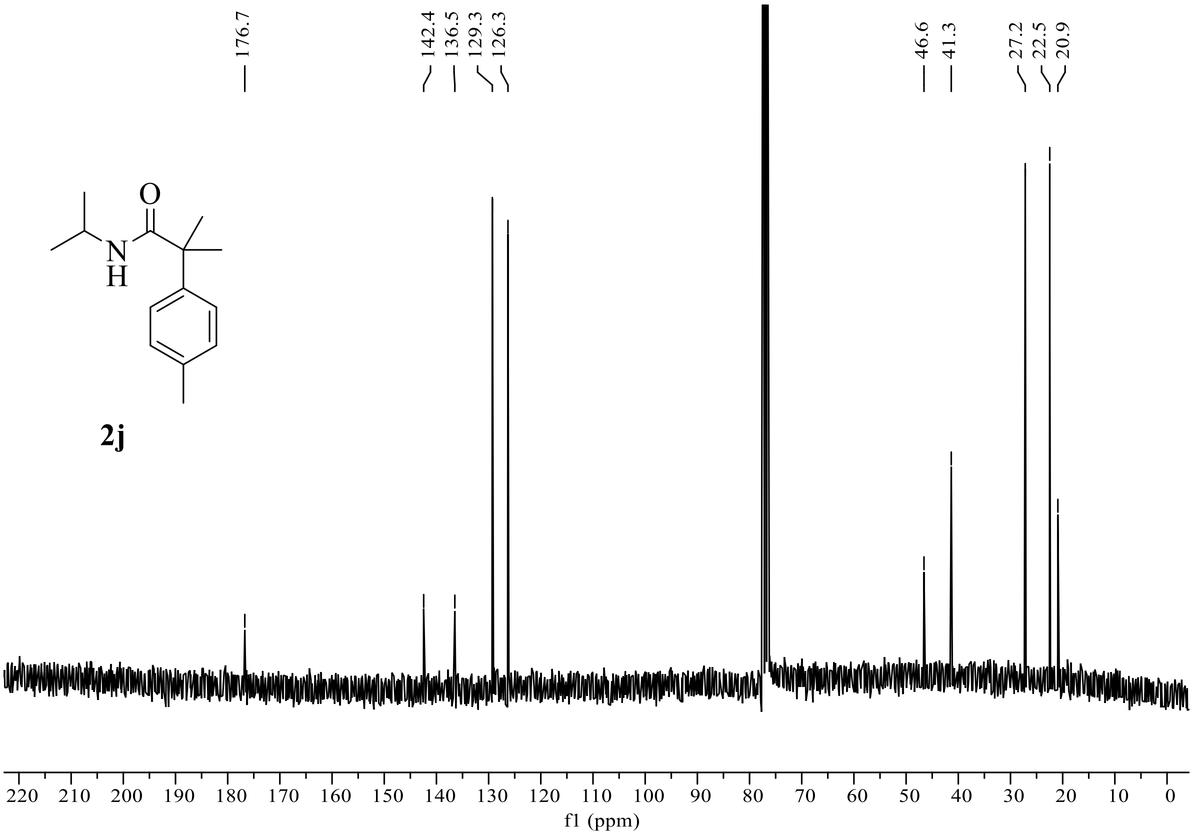

^{1}H NMR (300 MHz, $CDCl_3$):

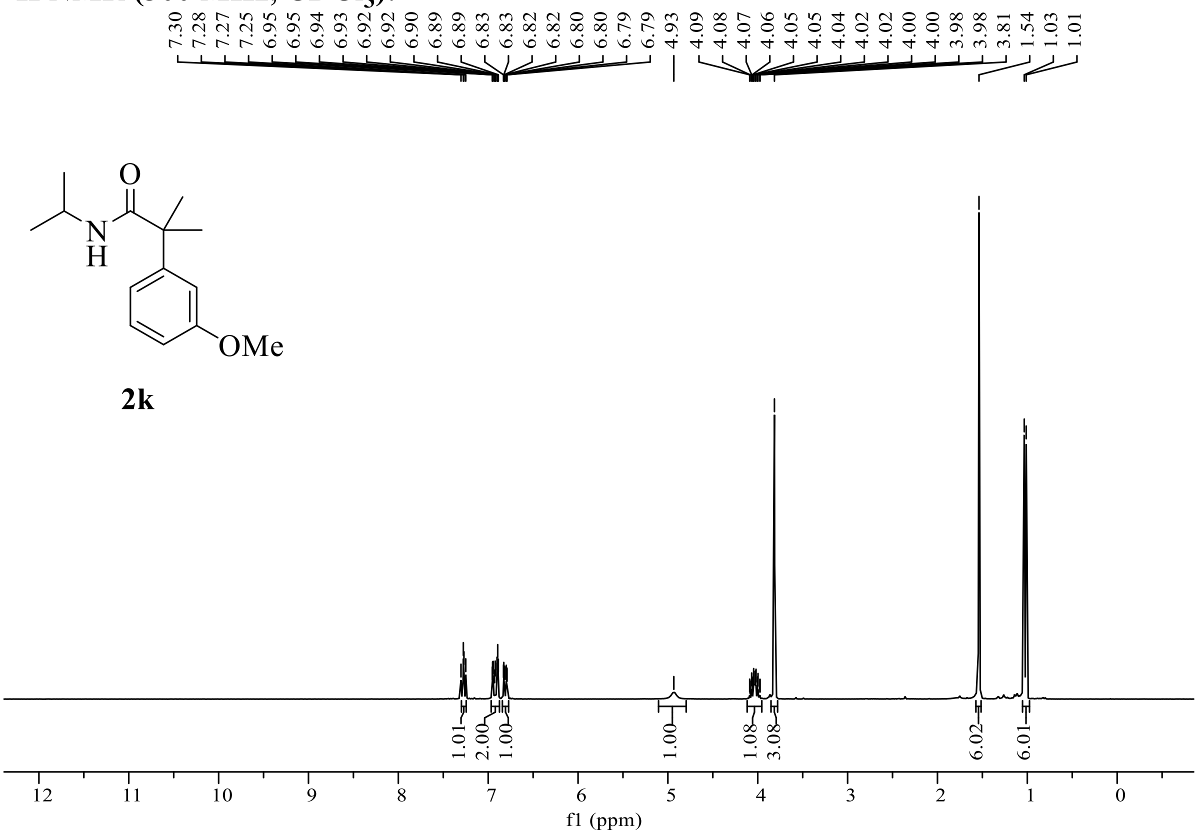

^{13}C NMR (75 MHz, $CDCl_3$):

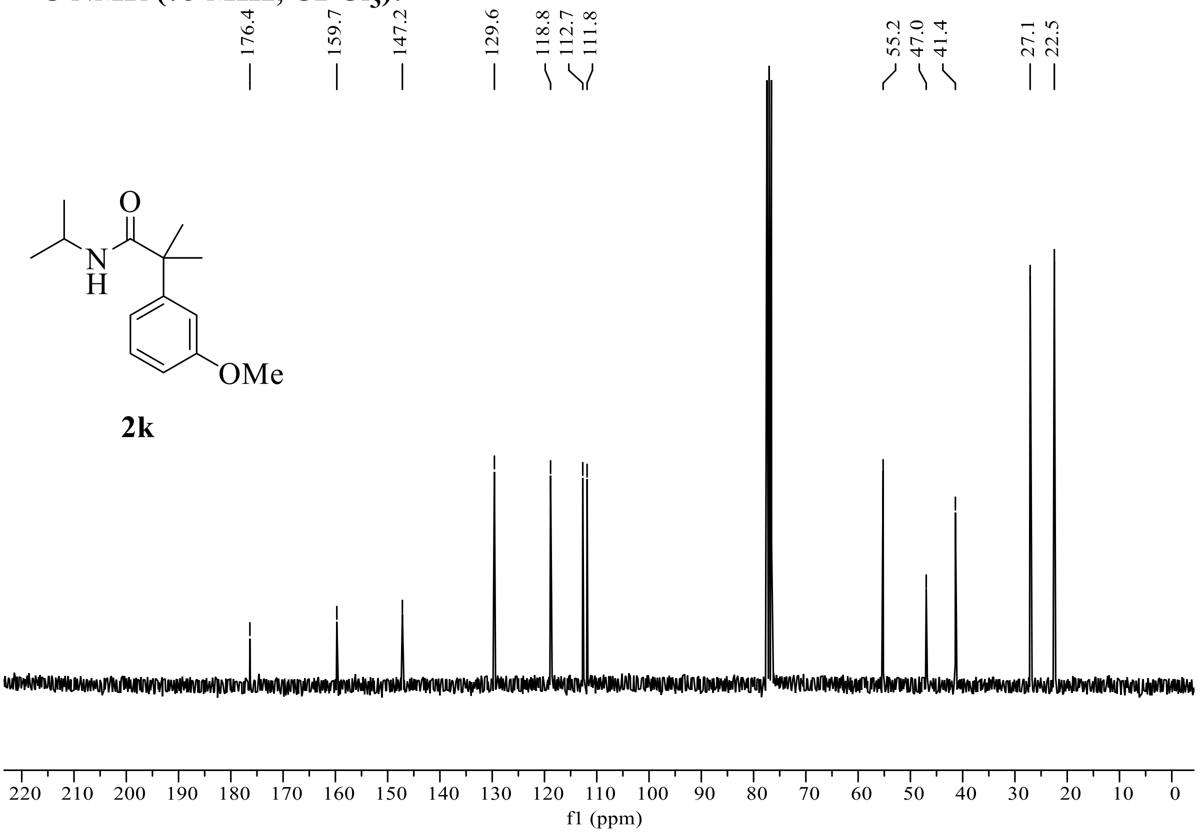

^{1}H NMR (300 MHz, $CDCl_3$):

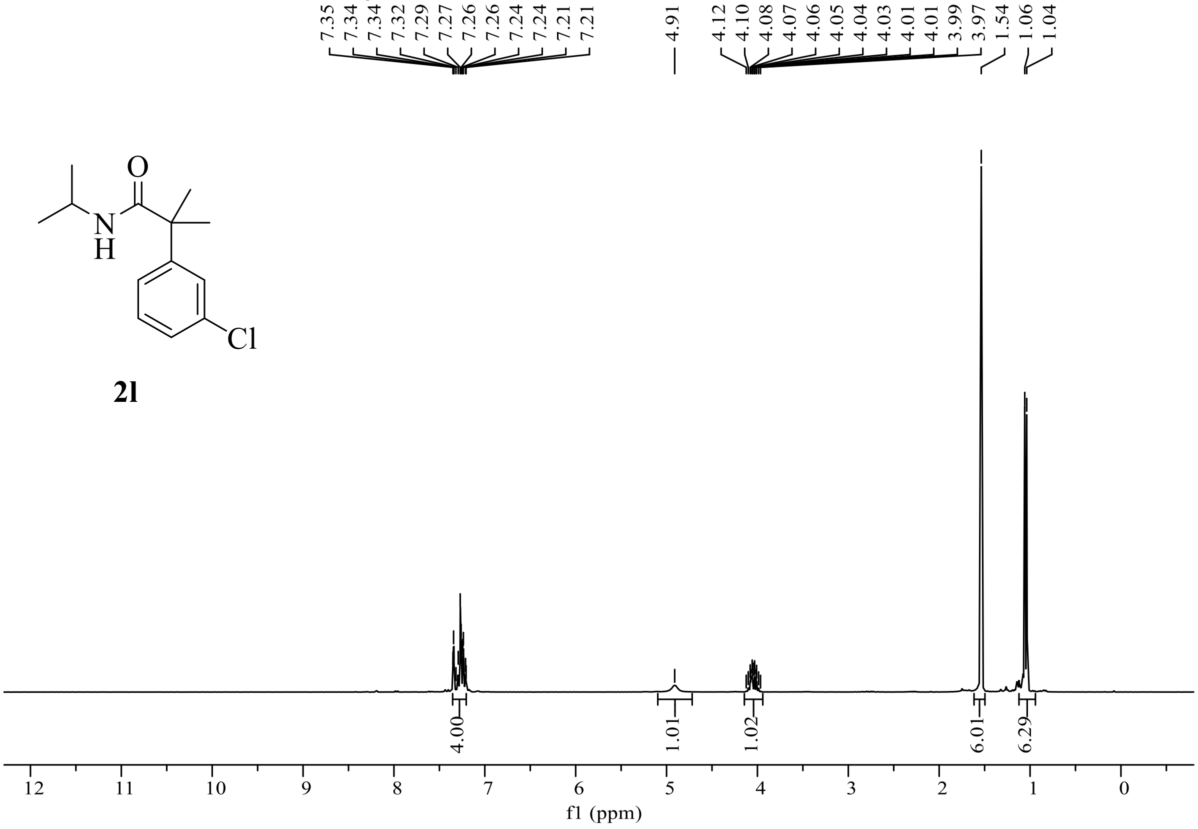

^{13}C NMR (75 MHz, $CDCl_3$):

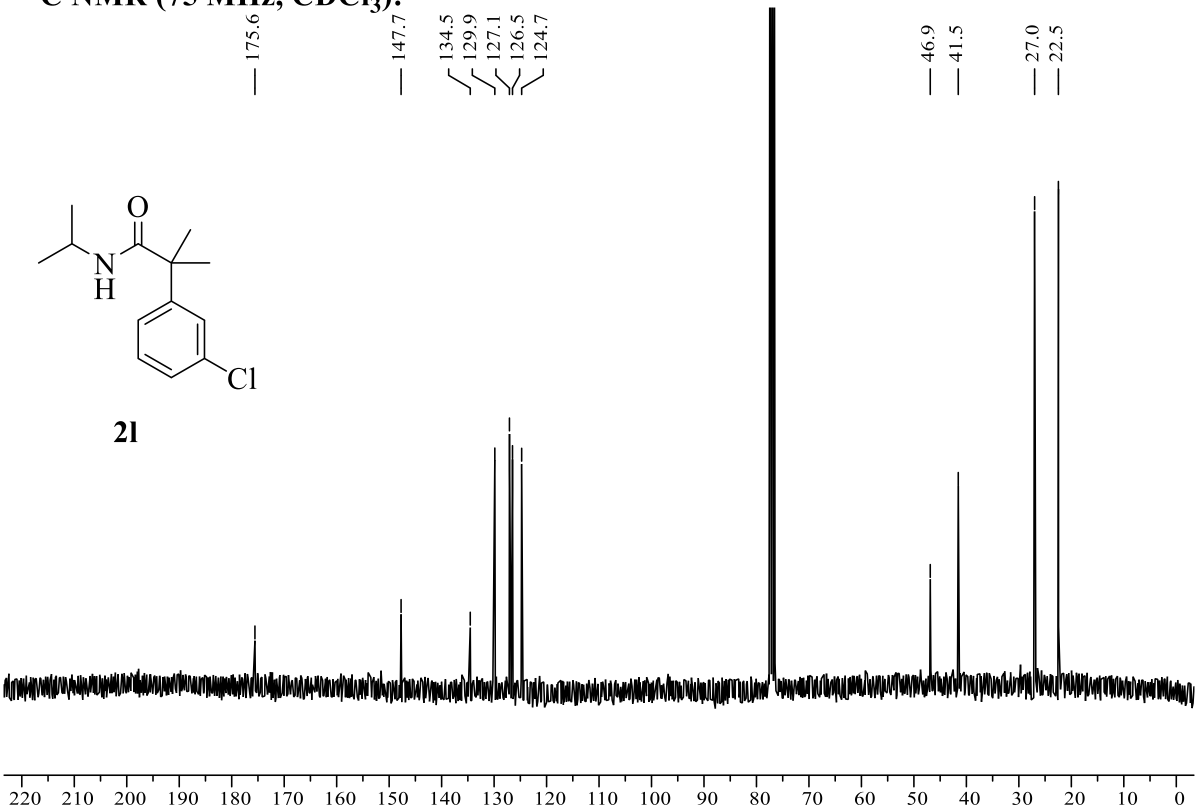

^{1}H NMR (600 MHz, CDCl$_3$):

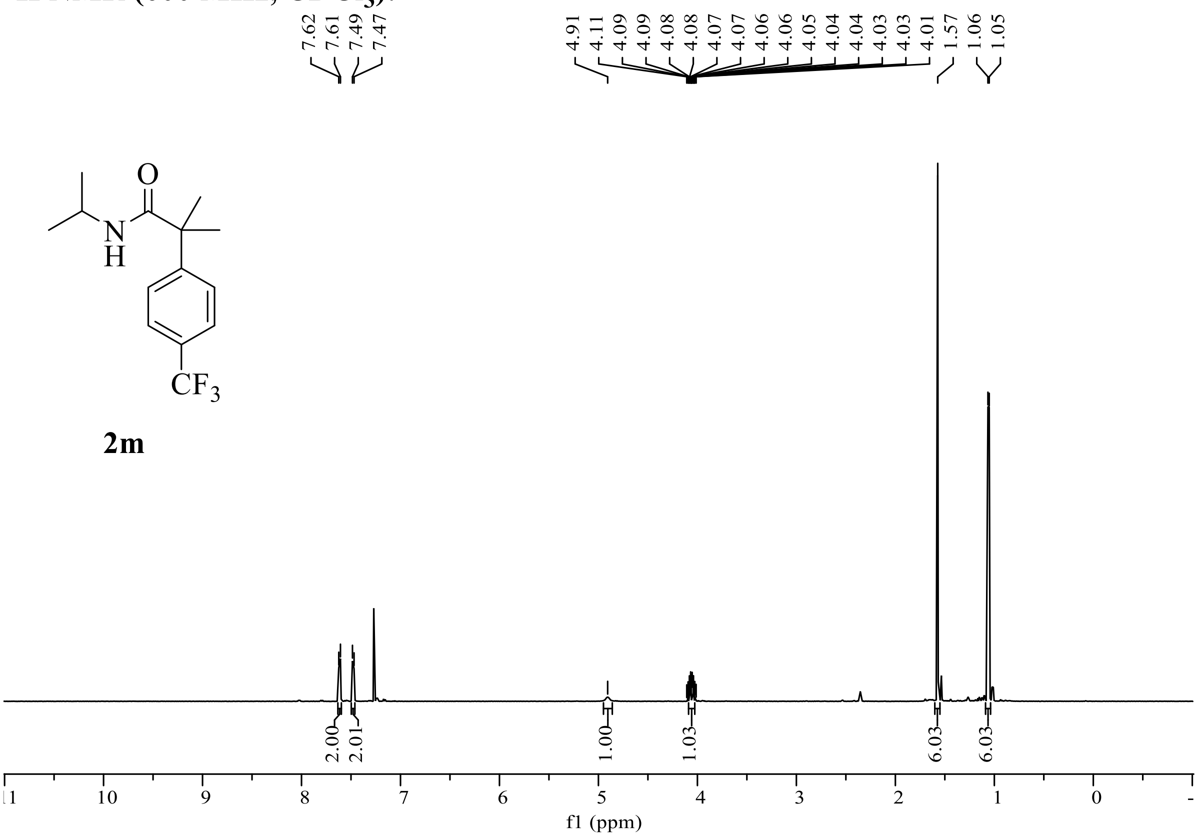

^{13}C{^{19}F} NMR (151 MHz, CDCl$_3$):

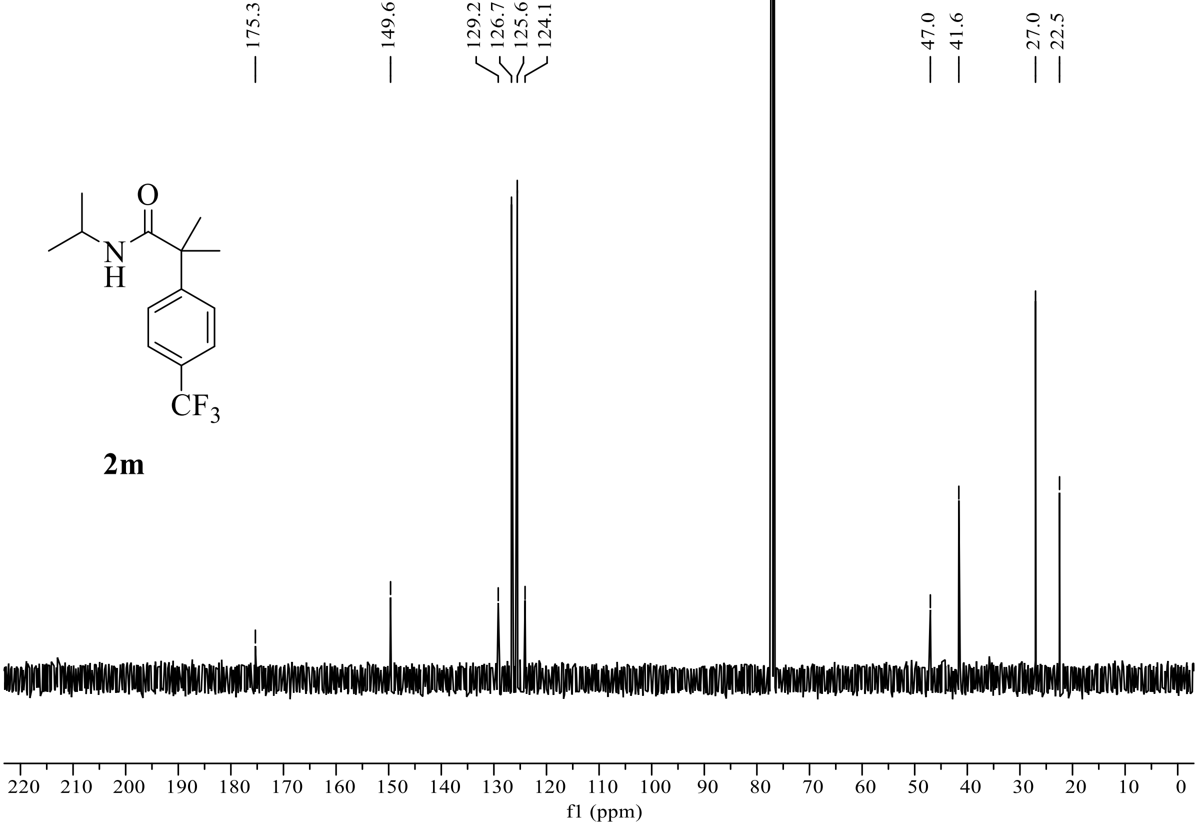

$^{19}F\{^{1}H\}$ NMR (470 MHz, $CDCl_3$):

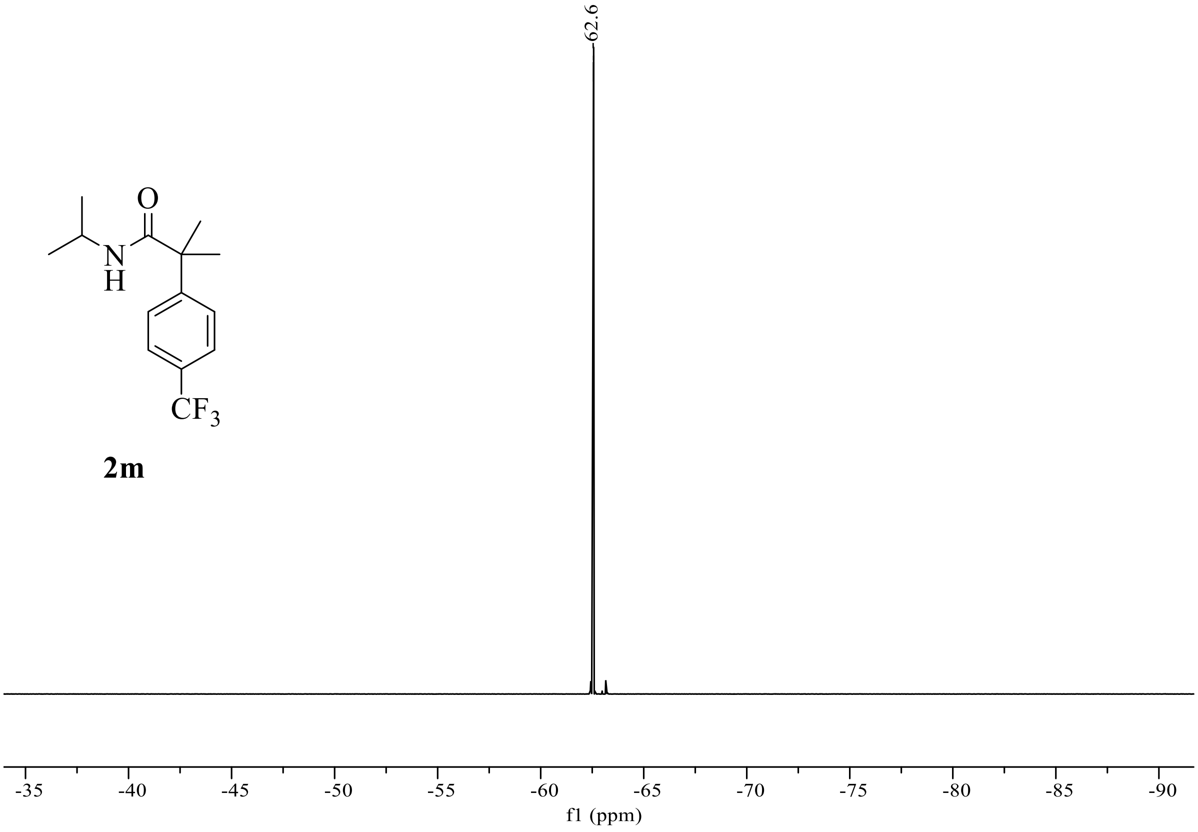

2m

^{1}H NMR (300 MHz, $CDCl_3$):

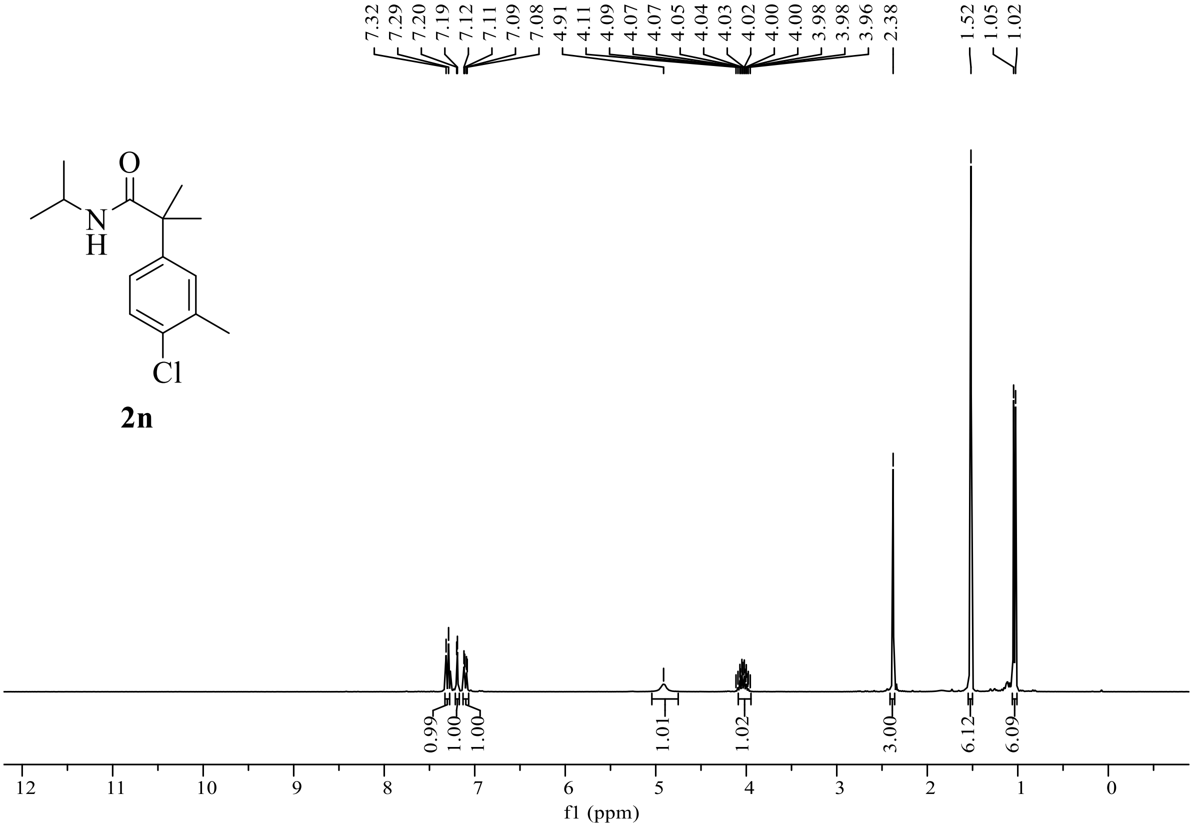

^{13}C NMR (75 MHz, $CDCl_3$):

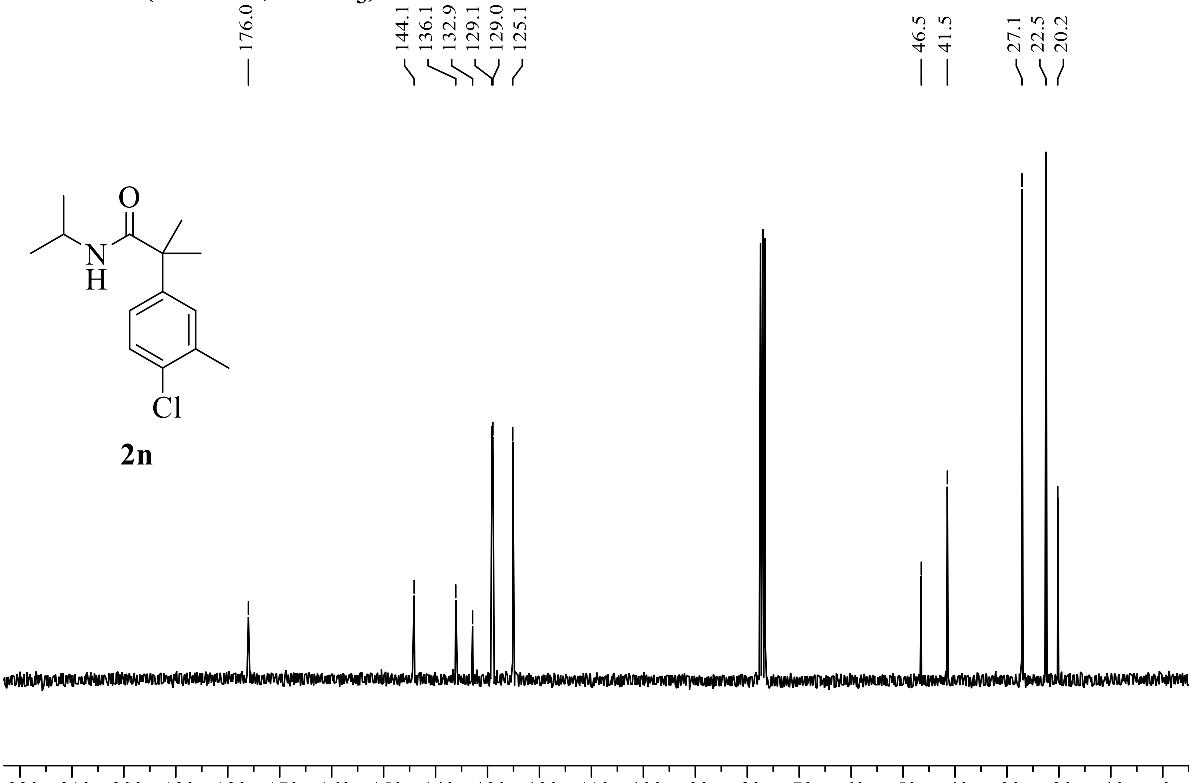

^{1}H NMR (300 MHz, $CDCl_3$):

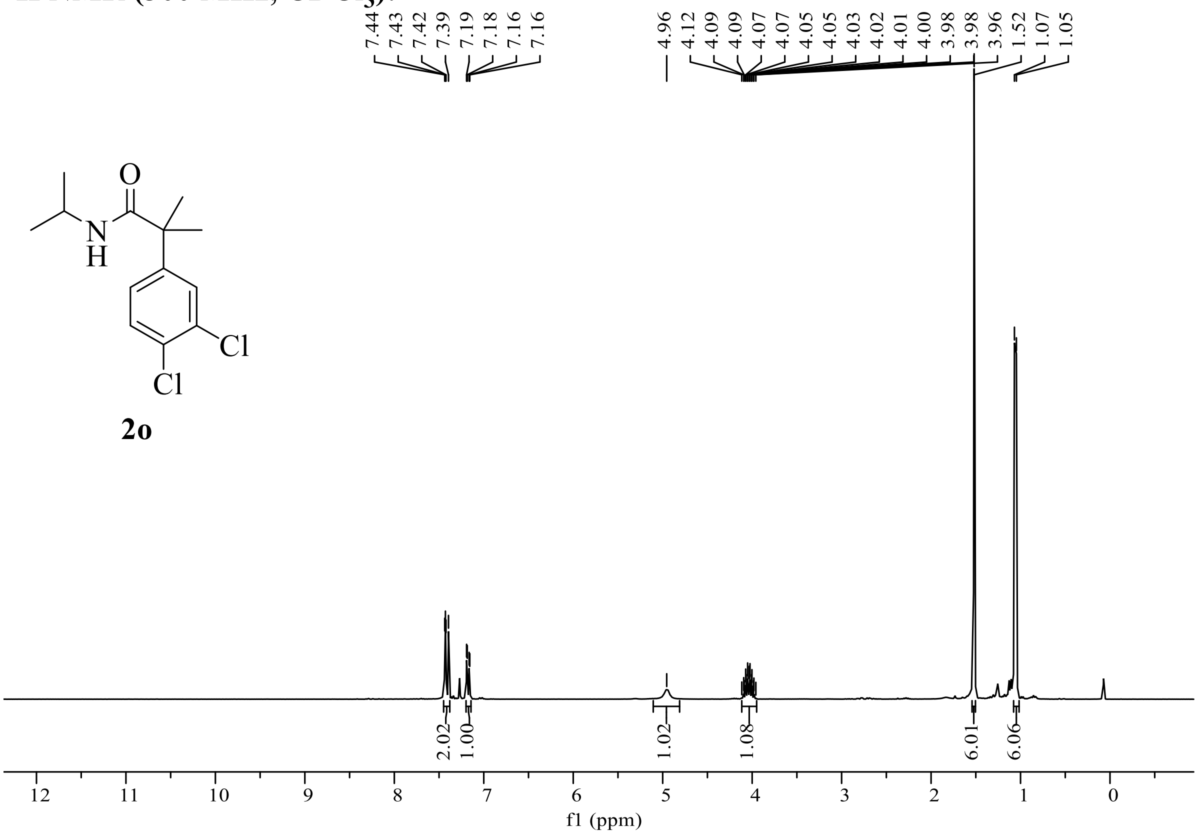

^{13}C NMR (75 MHz, $CDCl_3$):

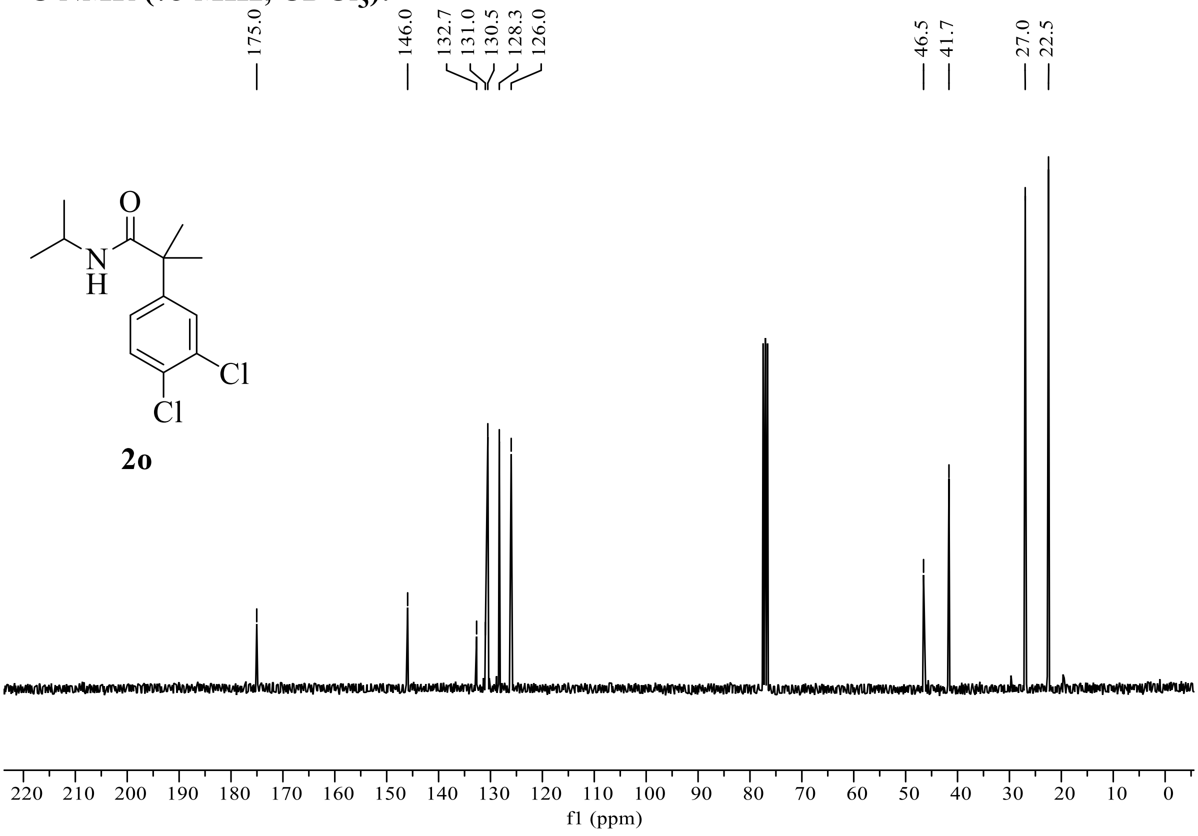

^{1}H NMR (300 MHz, $CDCl_3$):

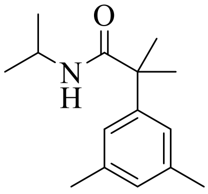

2p

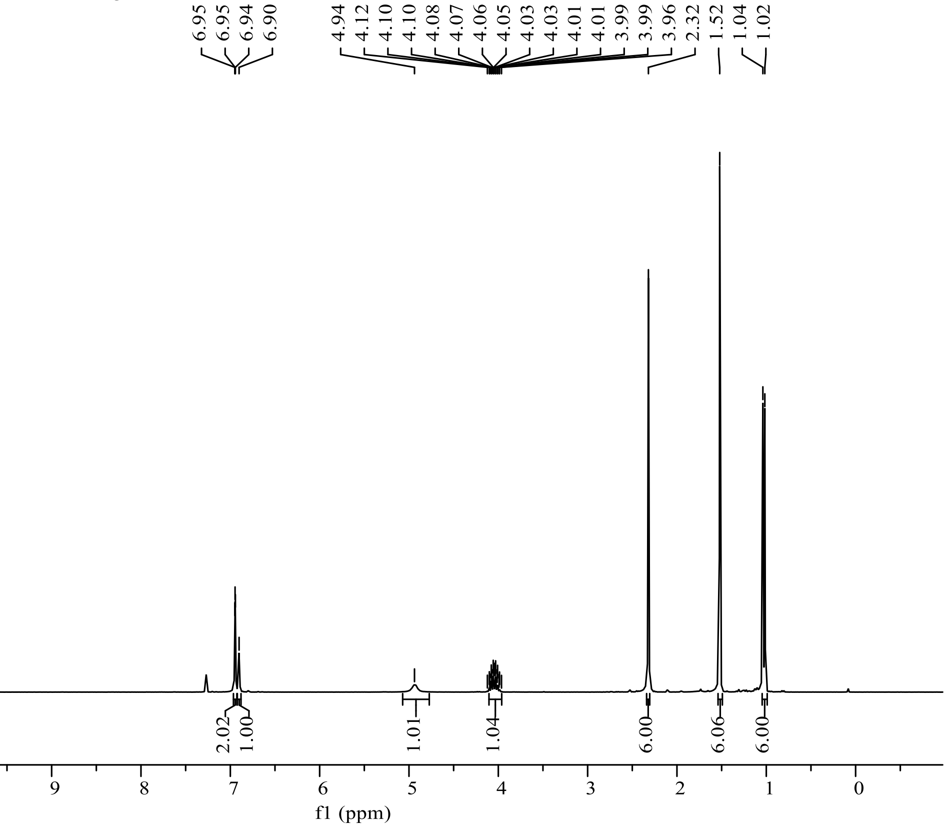

^{13}C NMR (75 MHz, $CDCl_3$):

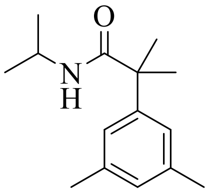

2p

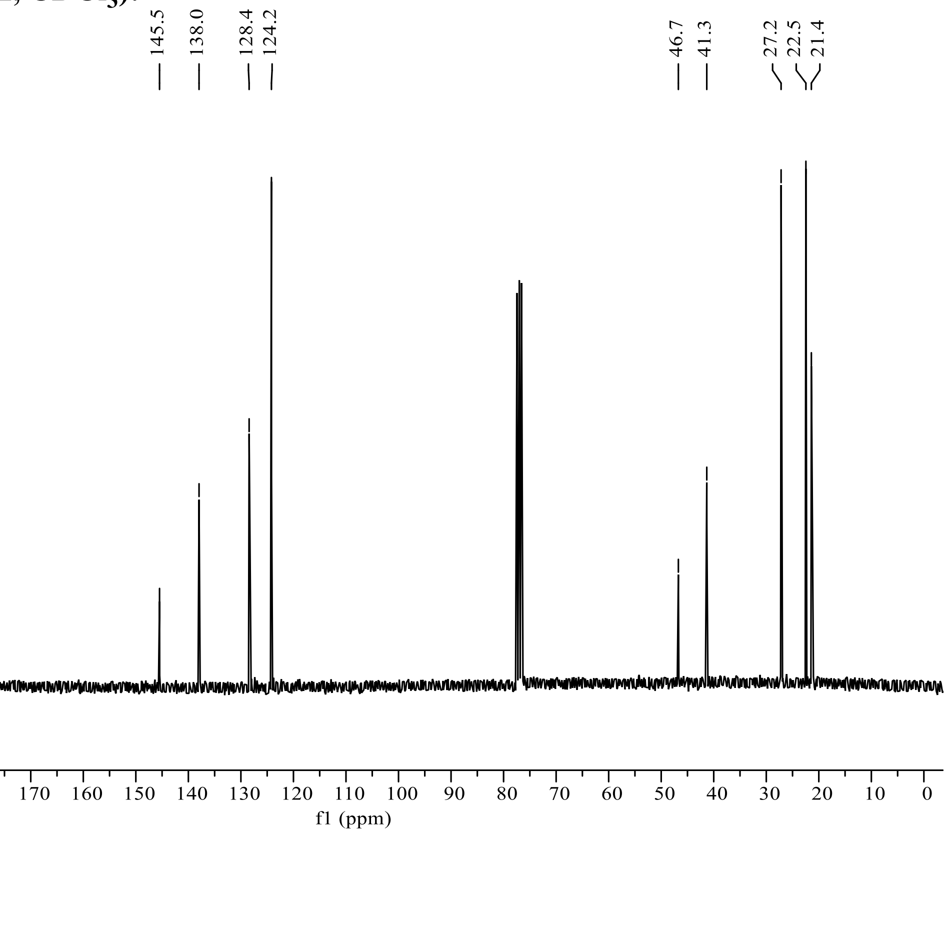

^{1}H NMR (600 MHz, $CDCl_3$):

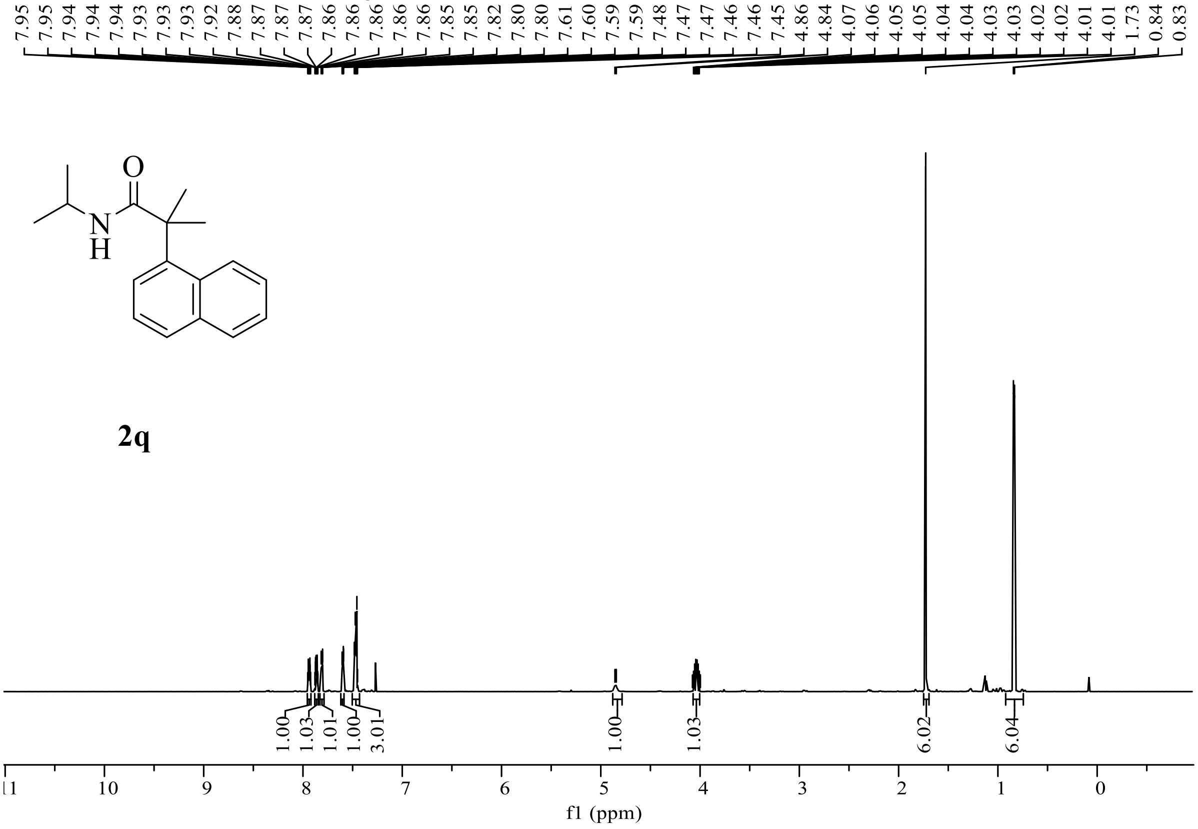

2q

^{13}C NMR (151 MHz, $CDCl_3$):

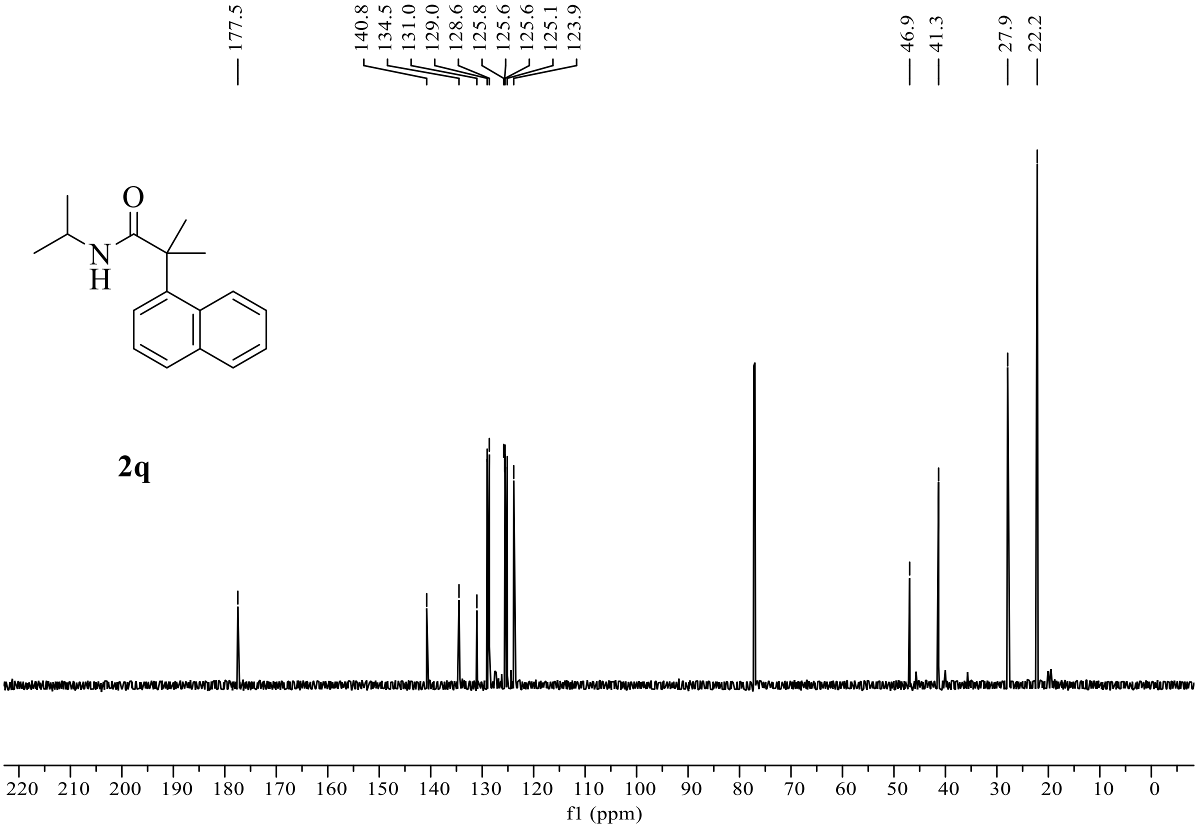

2q

^{1}H NMR (300 MHz, $CDCl_3$):

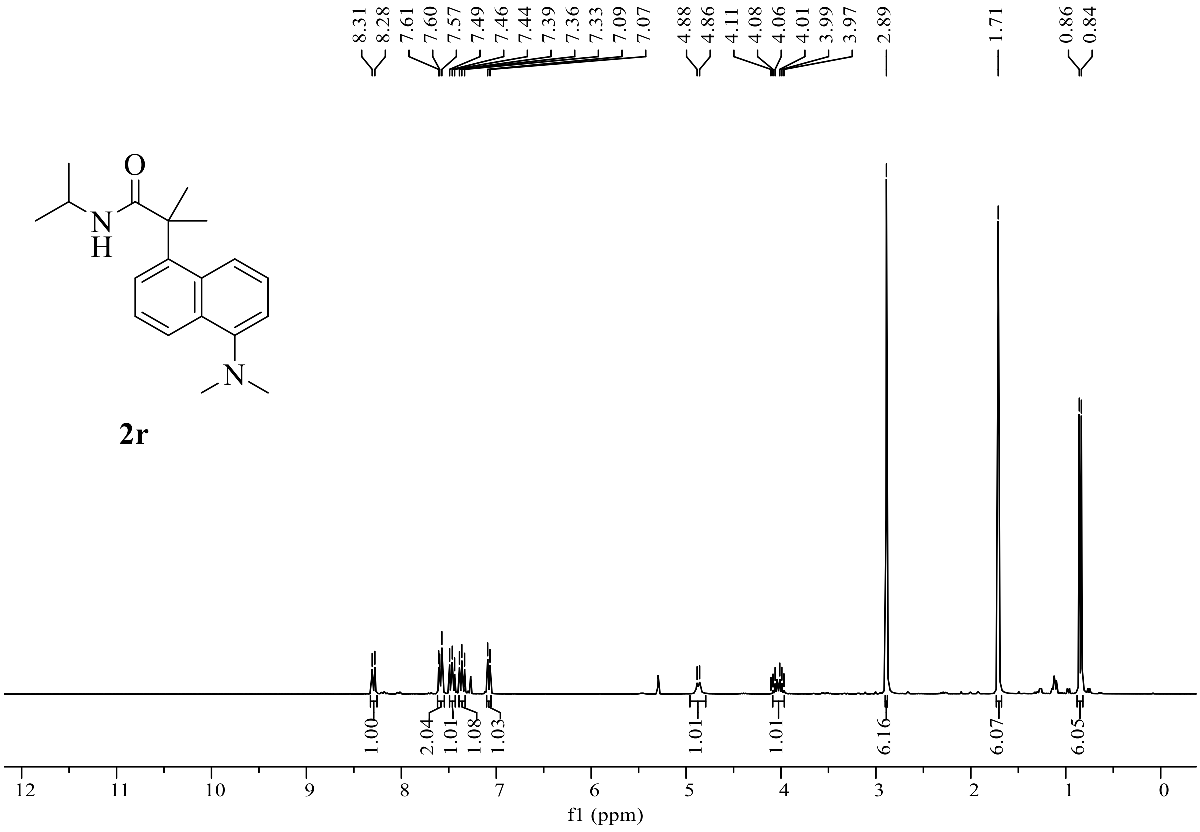

^{13}C NMR (75 MHz, $CDCl_3$):

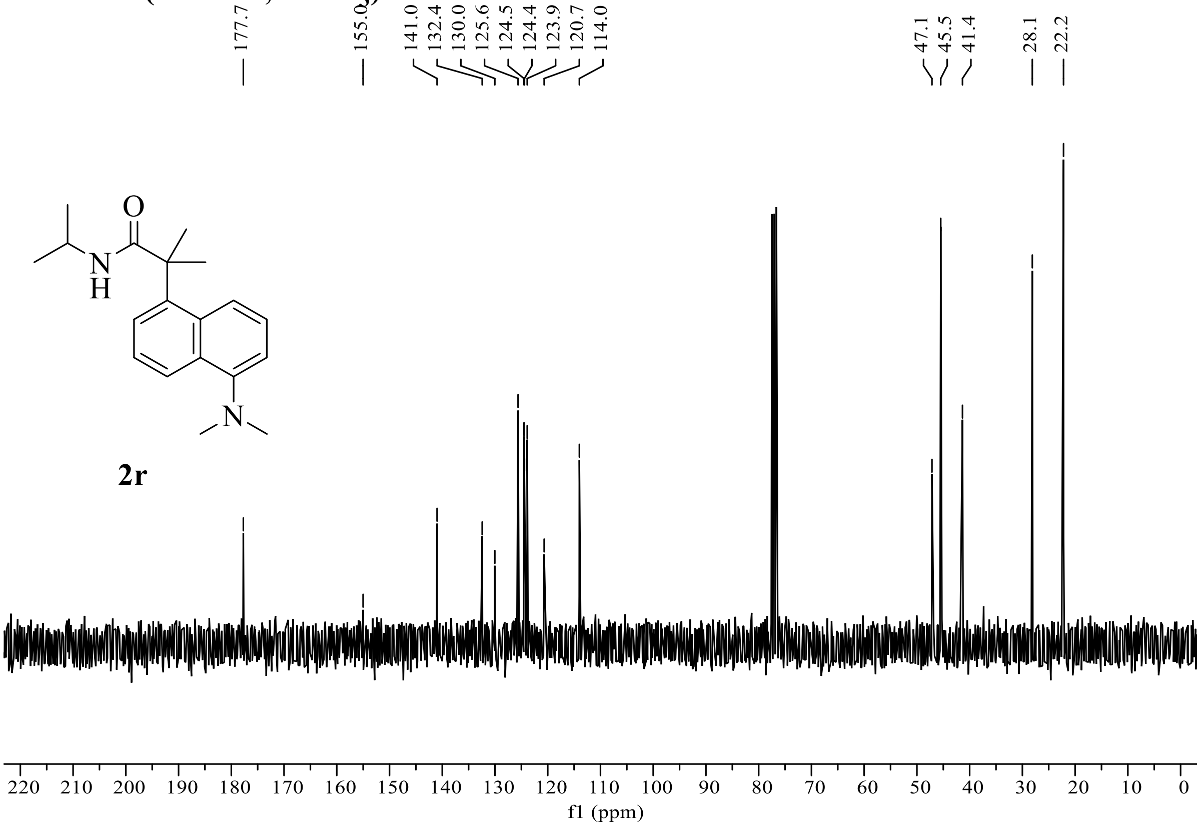

^{1}H NMR (600 MHz, $CDCl_3$):

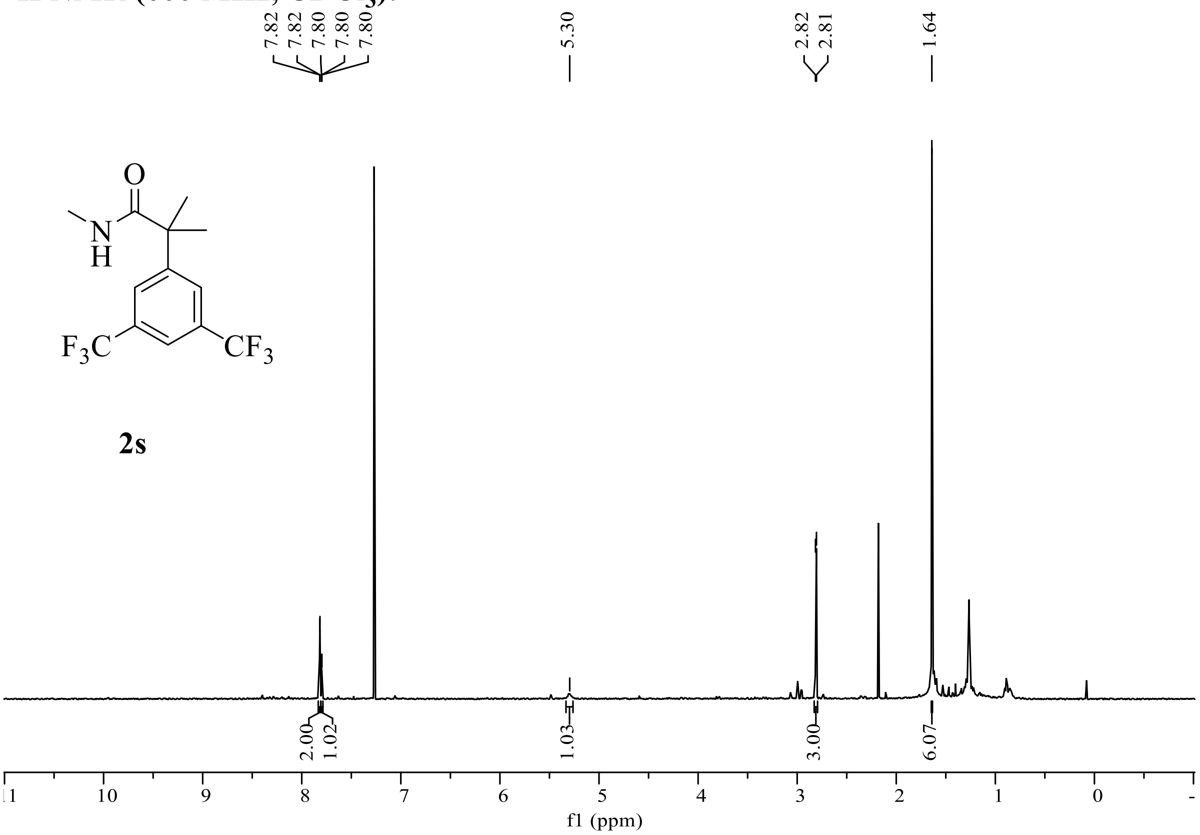

2s

^{13}C{^{19}F} NMR (151 MHz, $CDCl_3$):

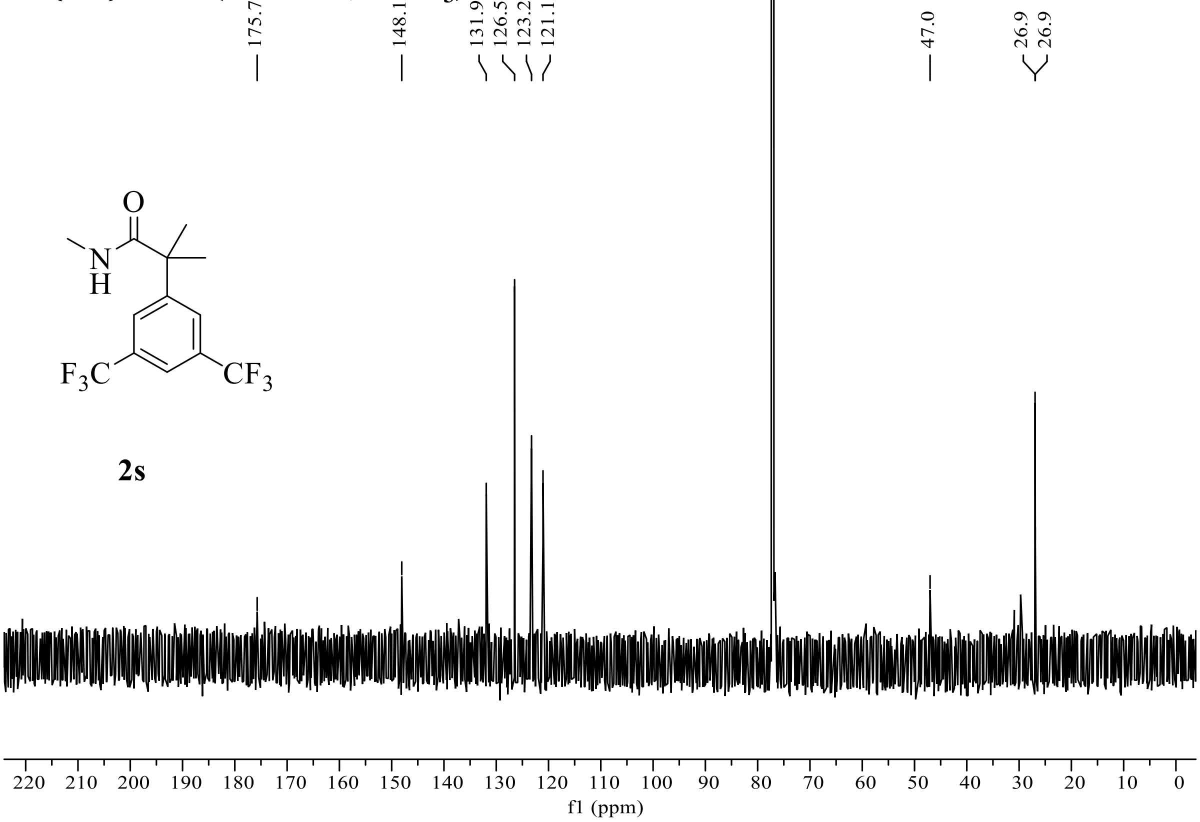

2s

$^{19}F\{^{1}H\}$ NMR (470 MHz, $CDCl_3$):

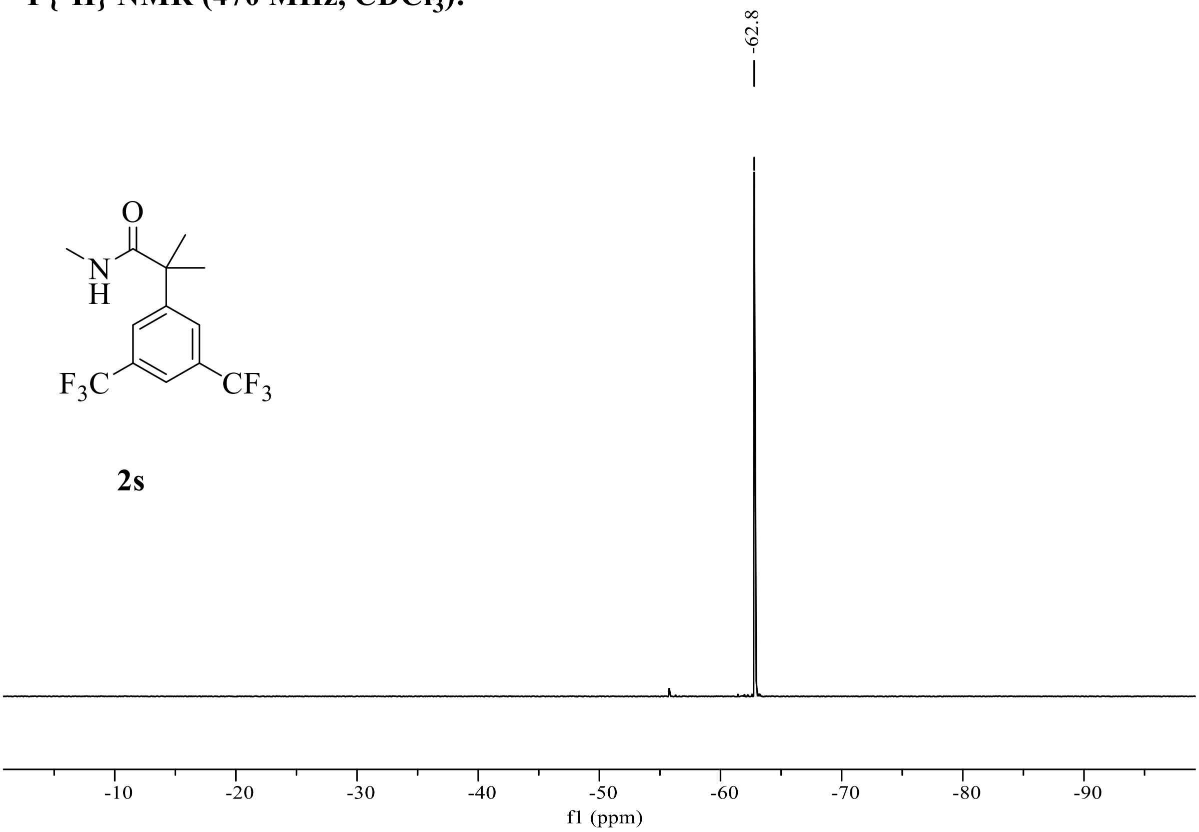

^{1}H NMR (600 MHz, $CDCl_3$):

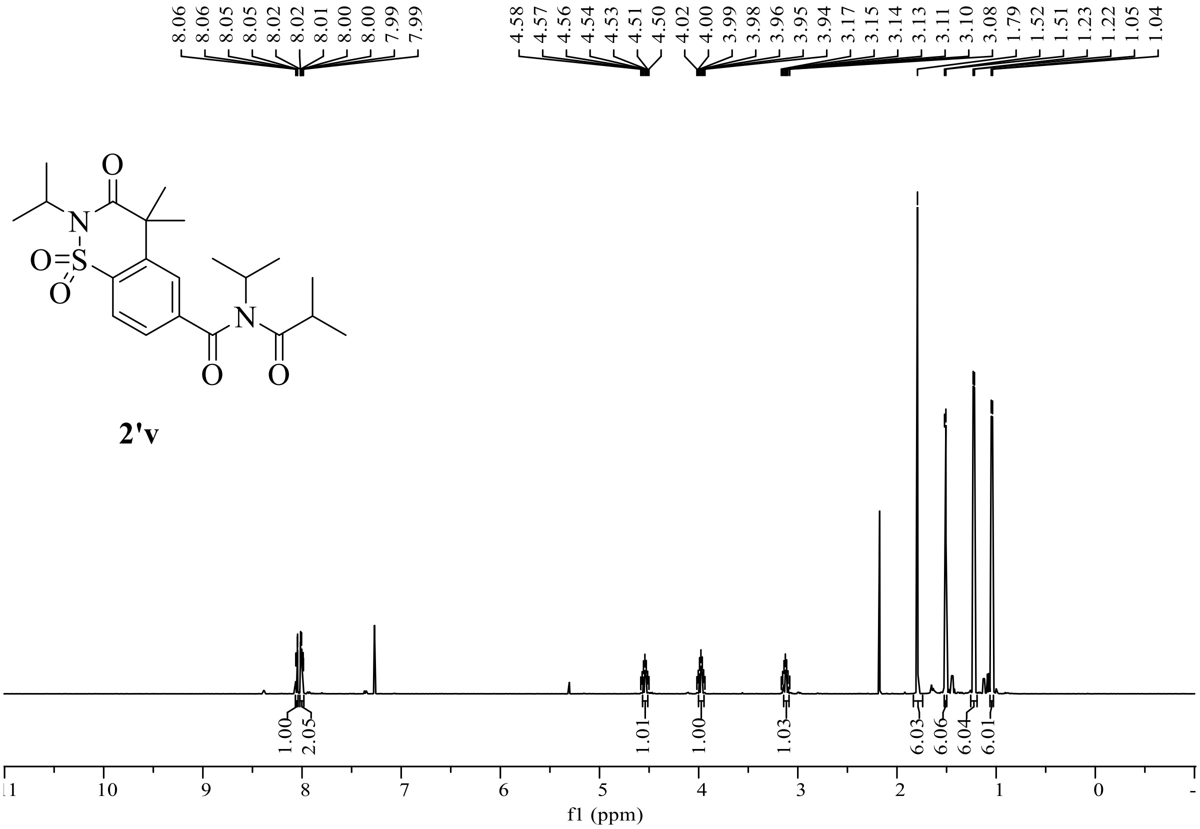

2'v

^{13}C NMR (151 MHz, $CDCl_3$):

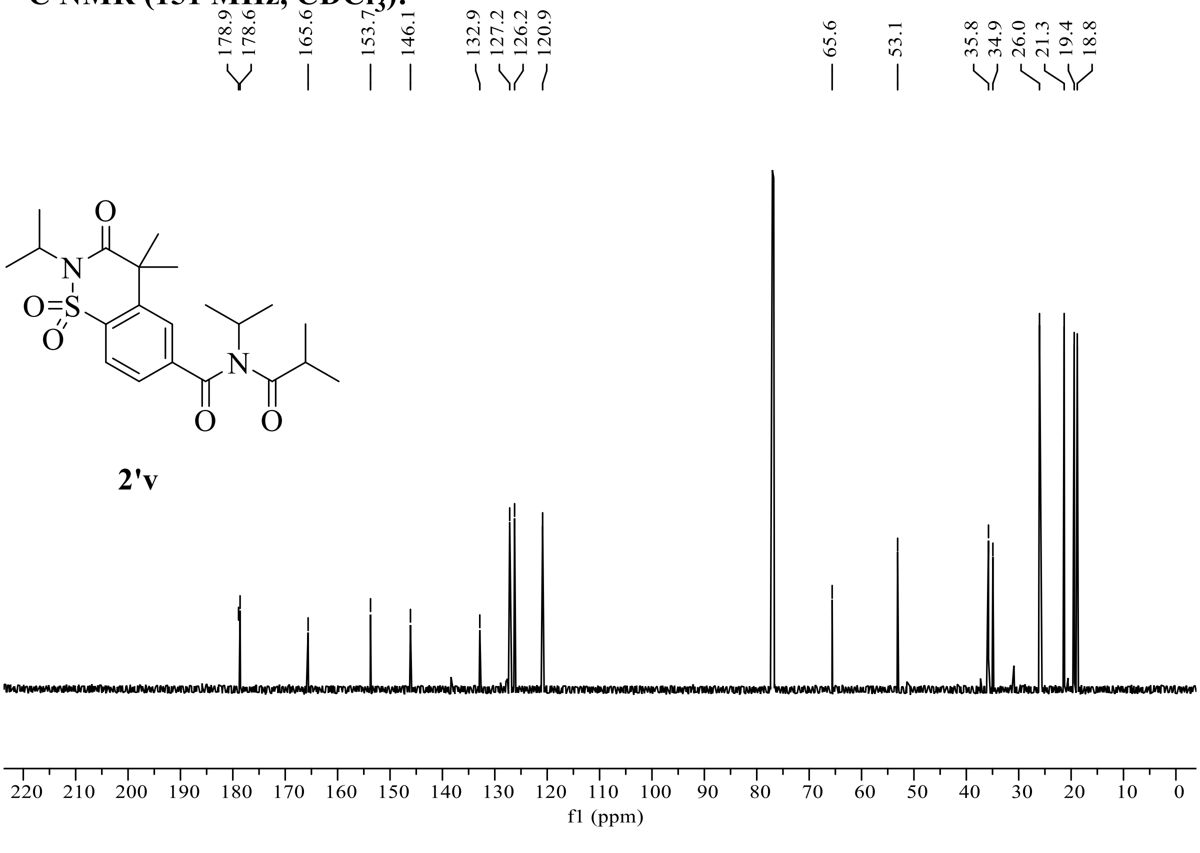

2'v

^{1}H NMR (300 MHz, $CDCl_3$):

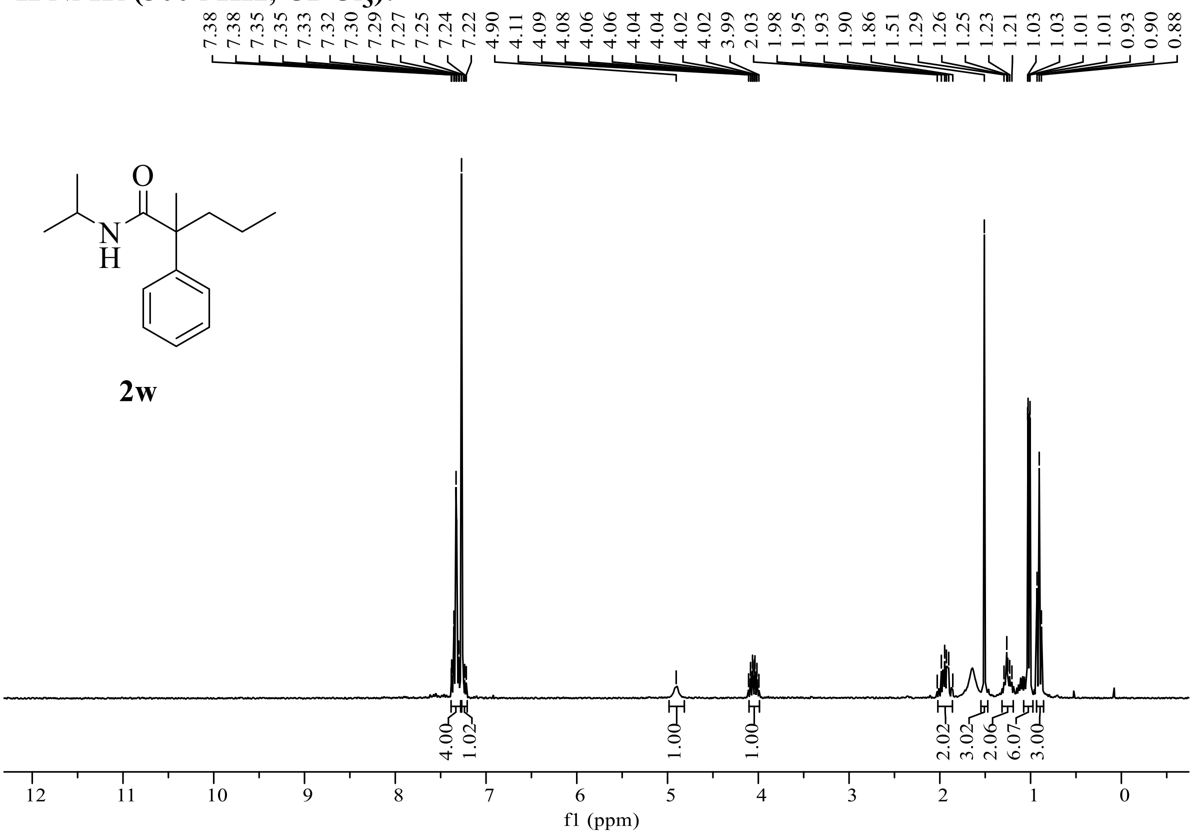

2w

^{13}C NMR (75 MHz, $CDCl_3$):

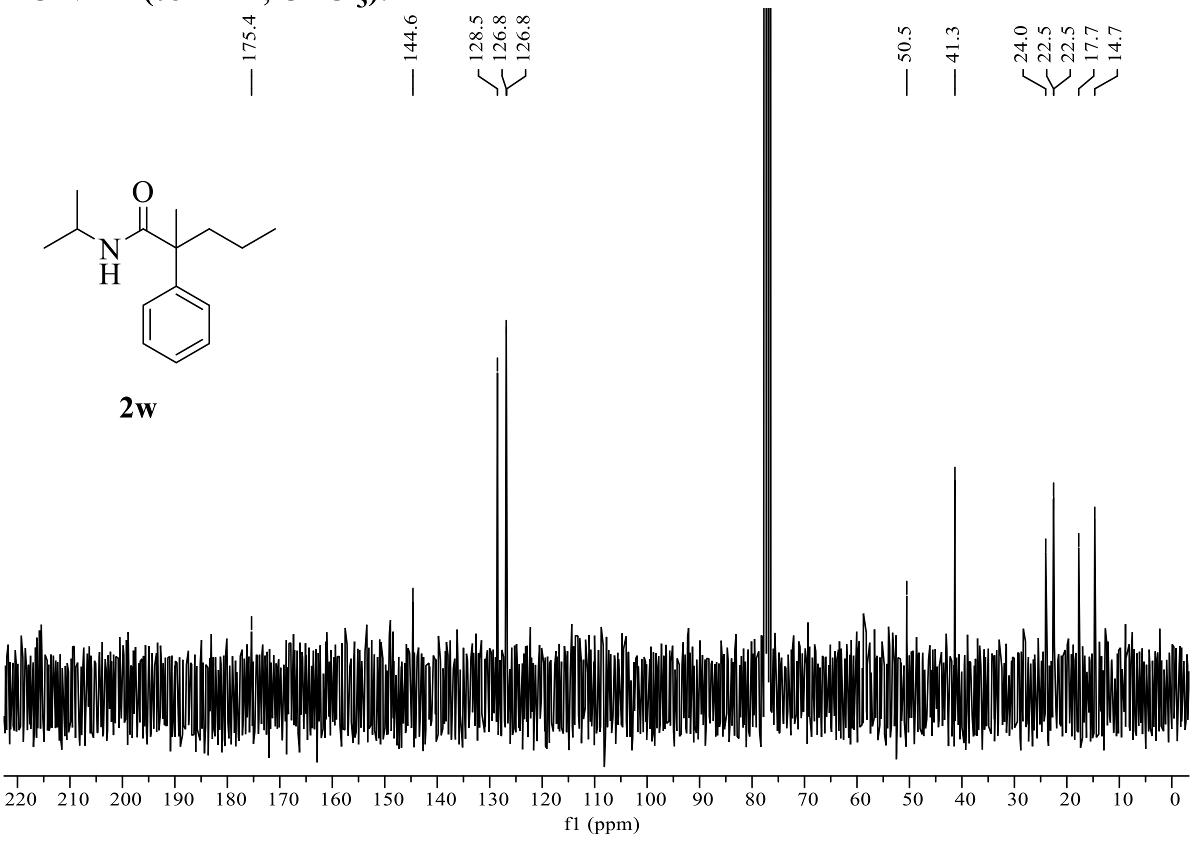

2w

^{1}H NMR (600 MHz, $CDCl_3$):

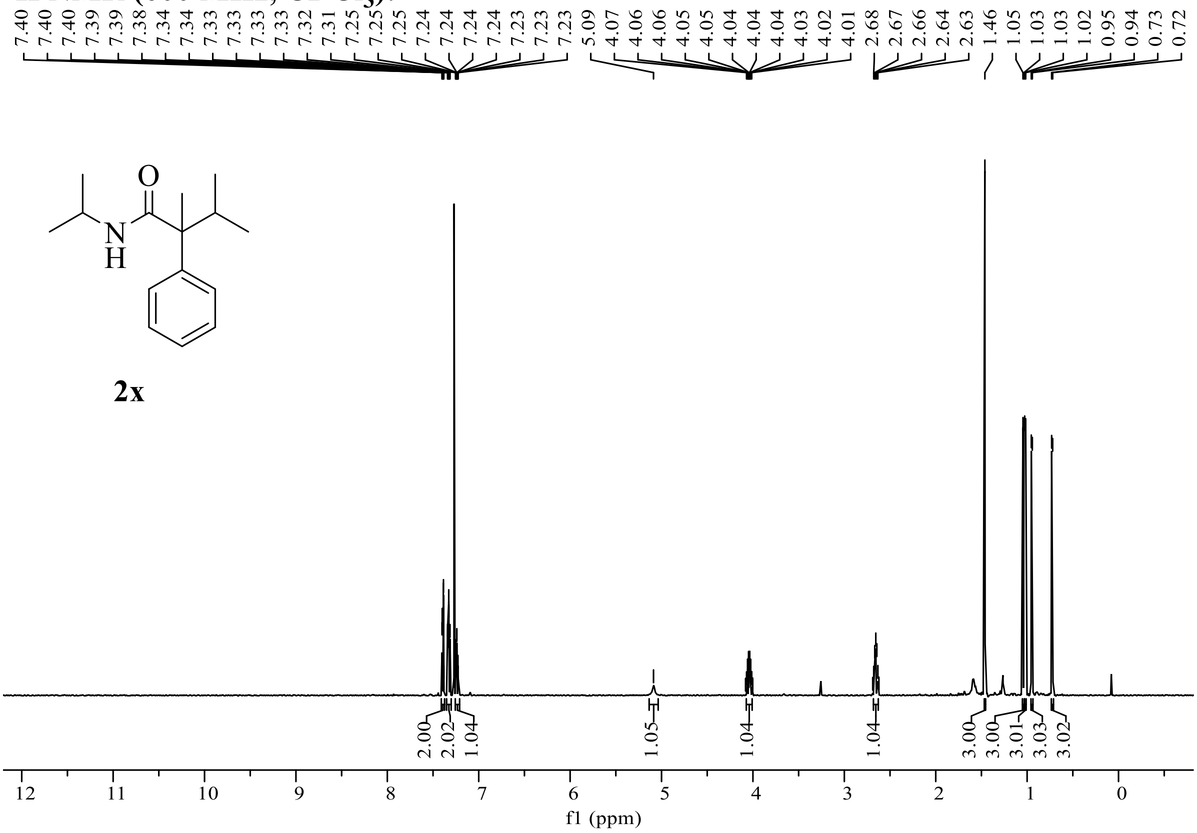

^{13}C NMR (151 MHz, $CDCl_3$):

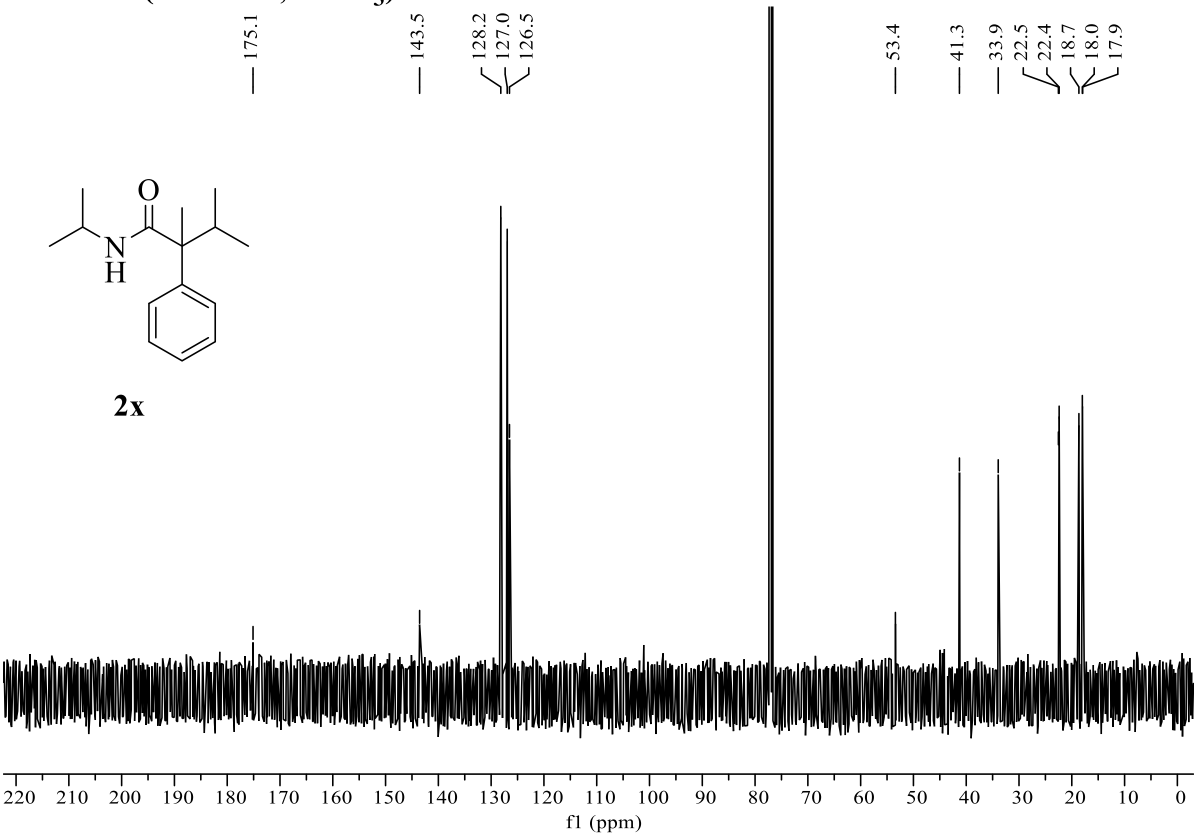

^{1}H NMR (300 MHz, $CDCl_3$):

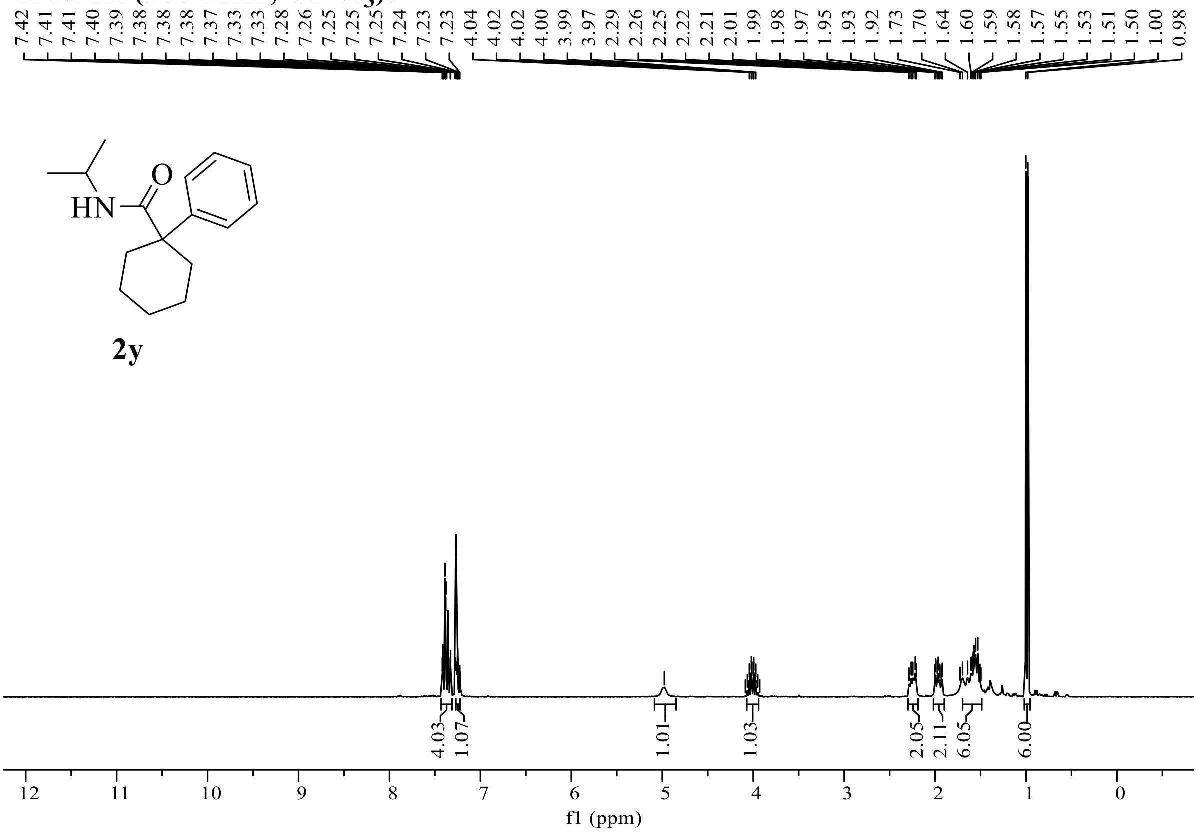

^{13}C NMR (75 MHz, $CDCl_3$):

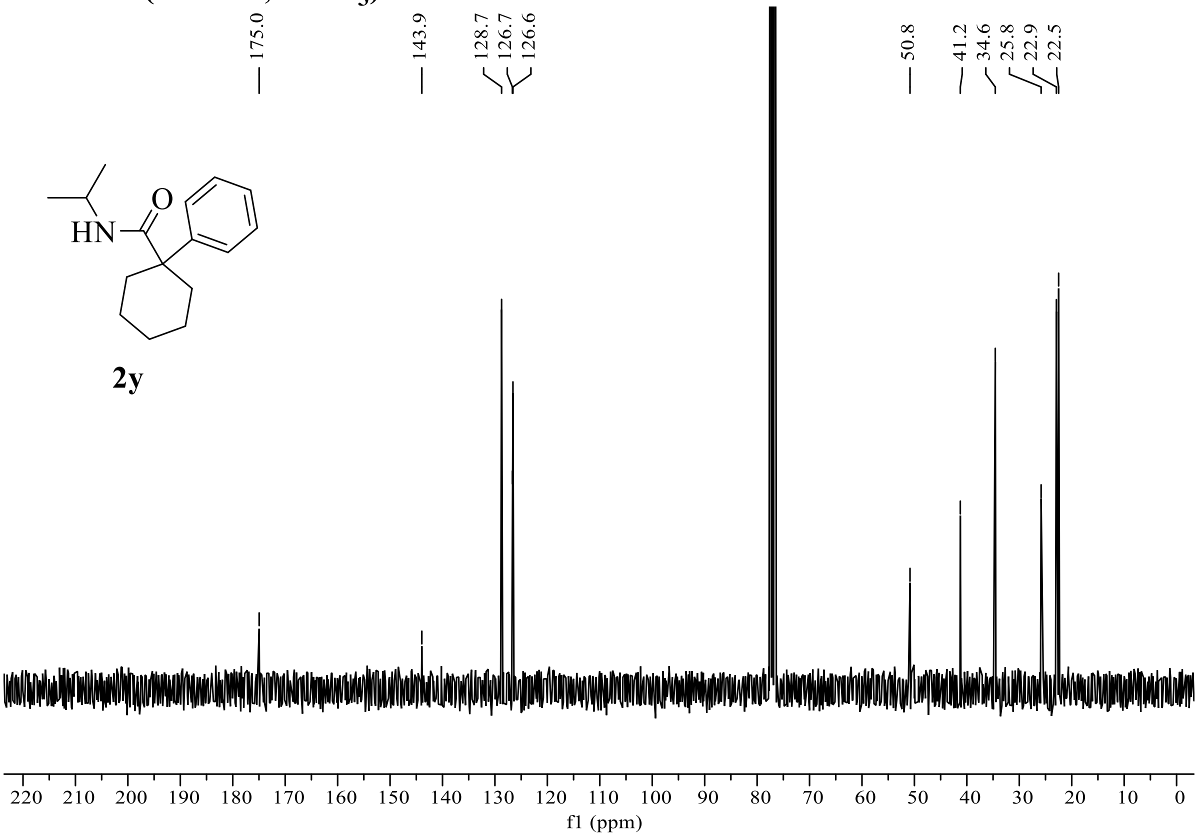

^{1}H NMR (600 MHz, $CDCl_3$):

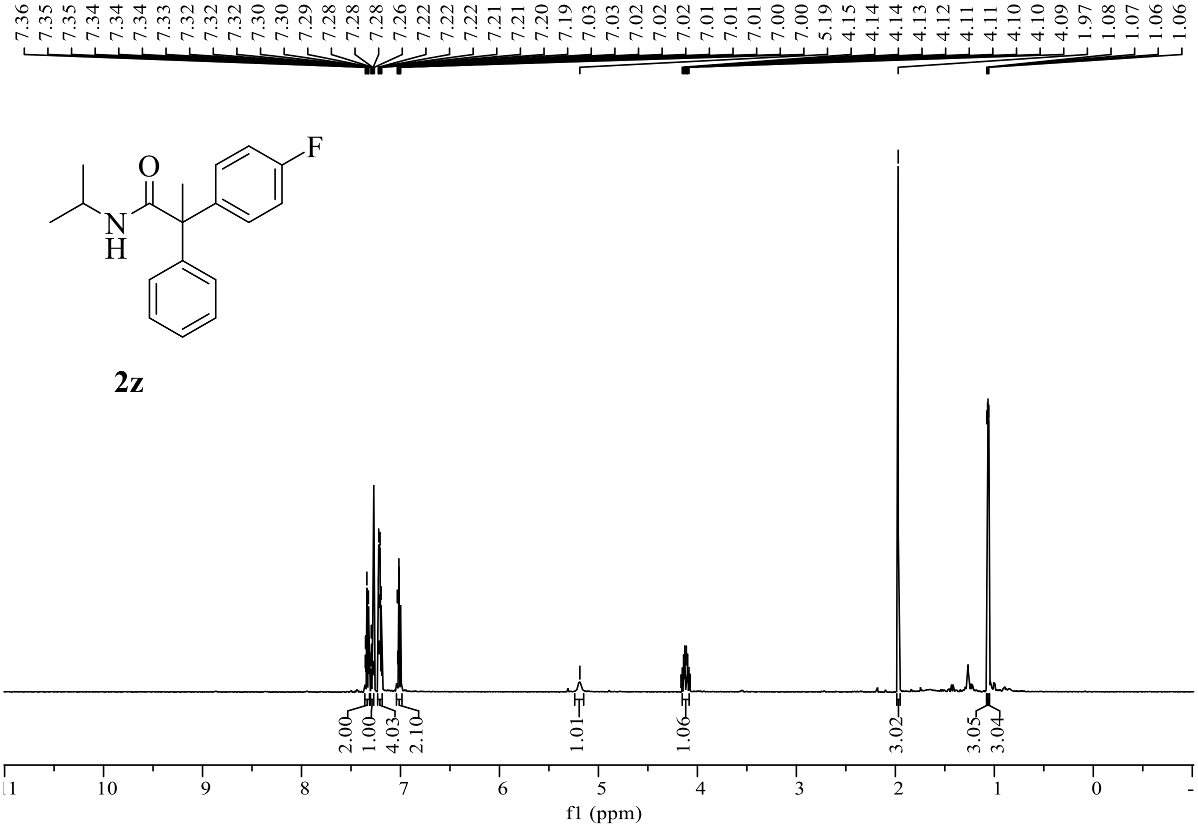

2z

^{13}C{^{19}F} NMR (151 MHz, $CDCl_3$):

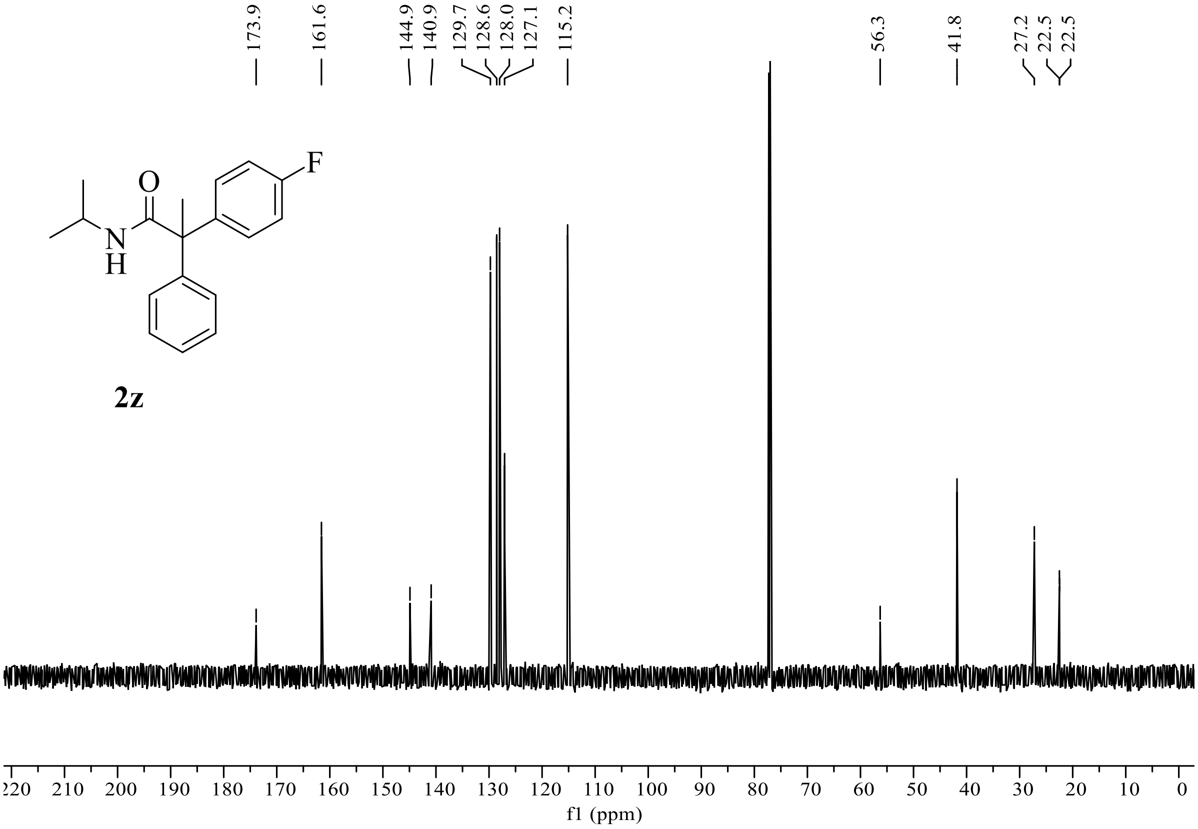

2z

$^{19}F\{^{1}H\}$ NMR (470 MHz, $CDCl_3$):

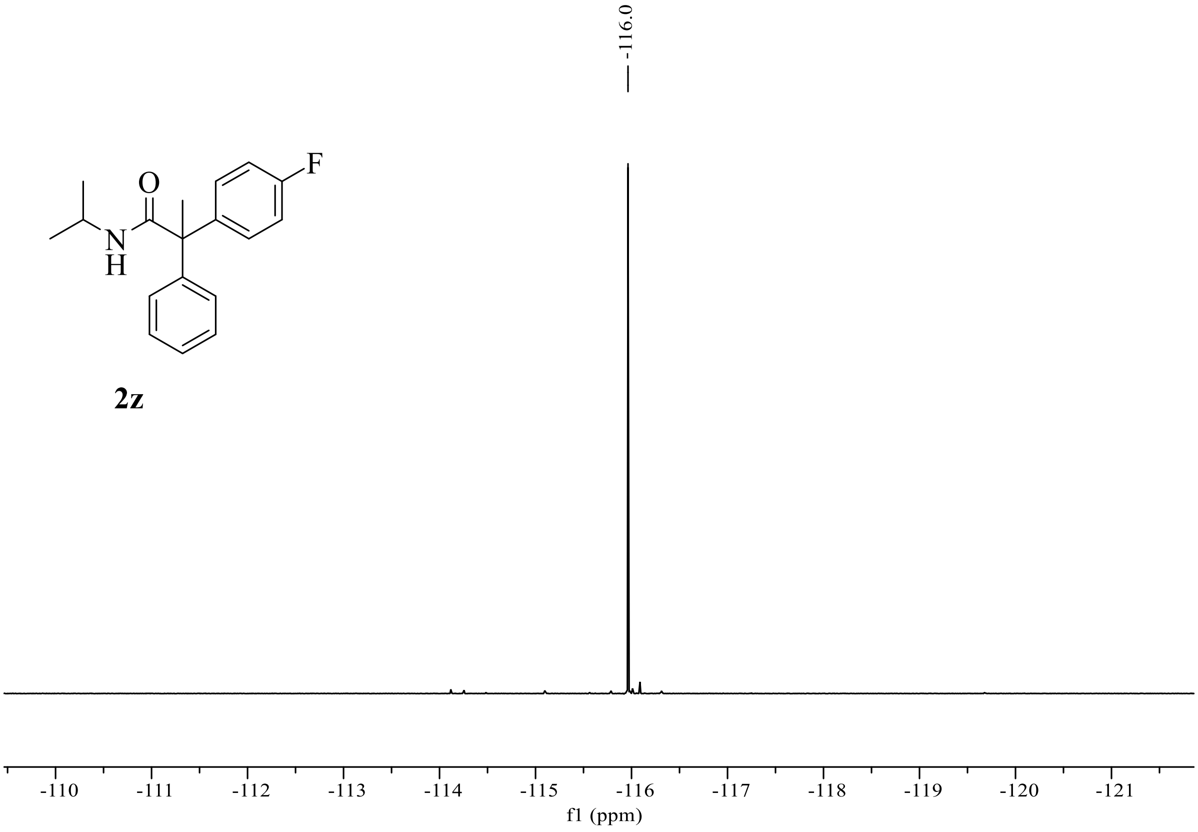

2z

^{1}H NMR (300 MHz, $CDCl_3$):

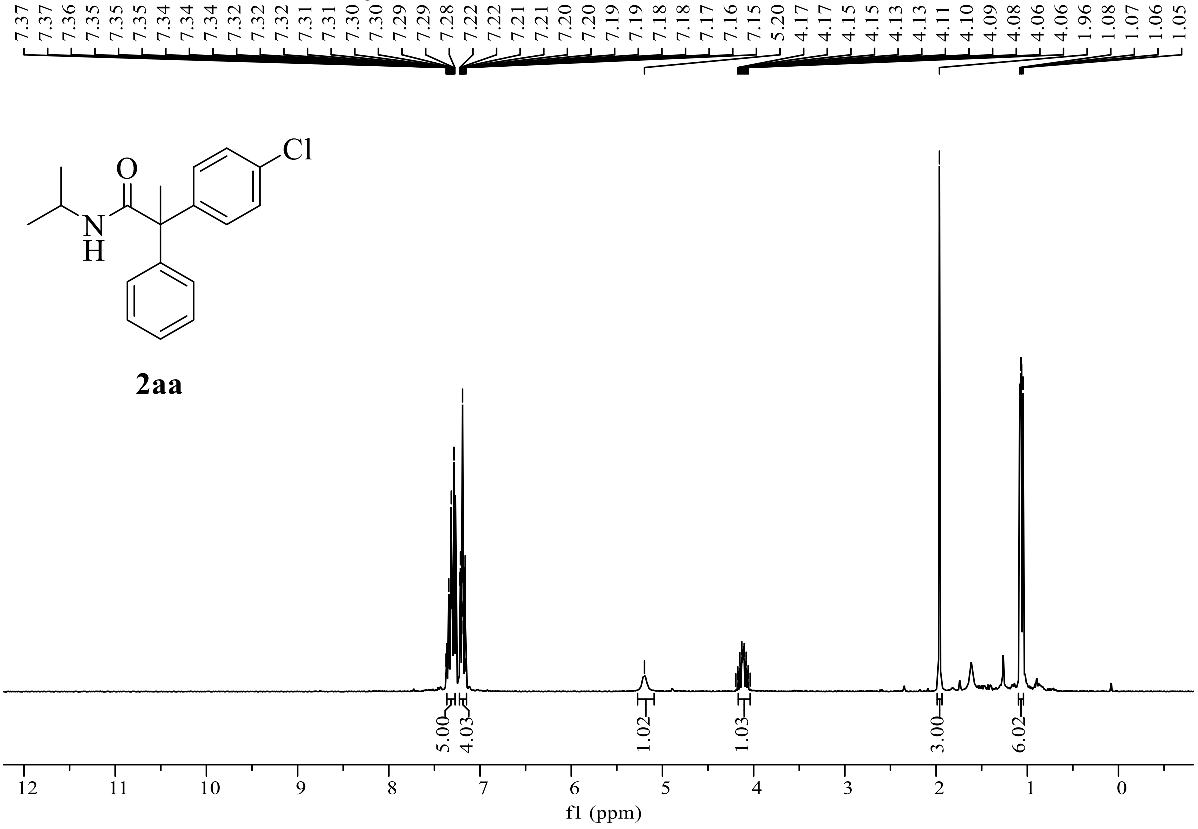

^{13}C NMR (75 MHz, $CDCl_3$):

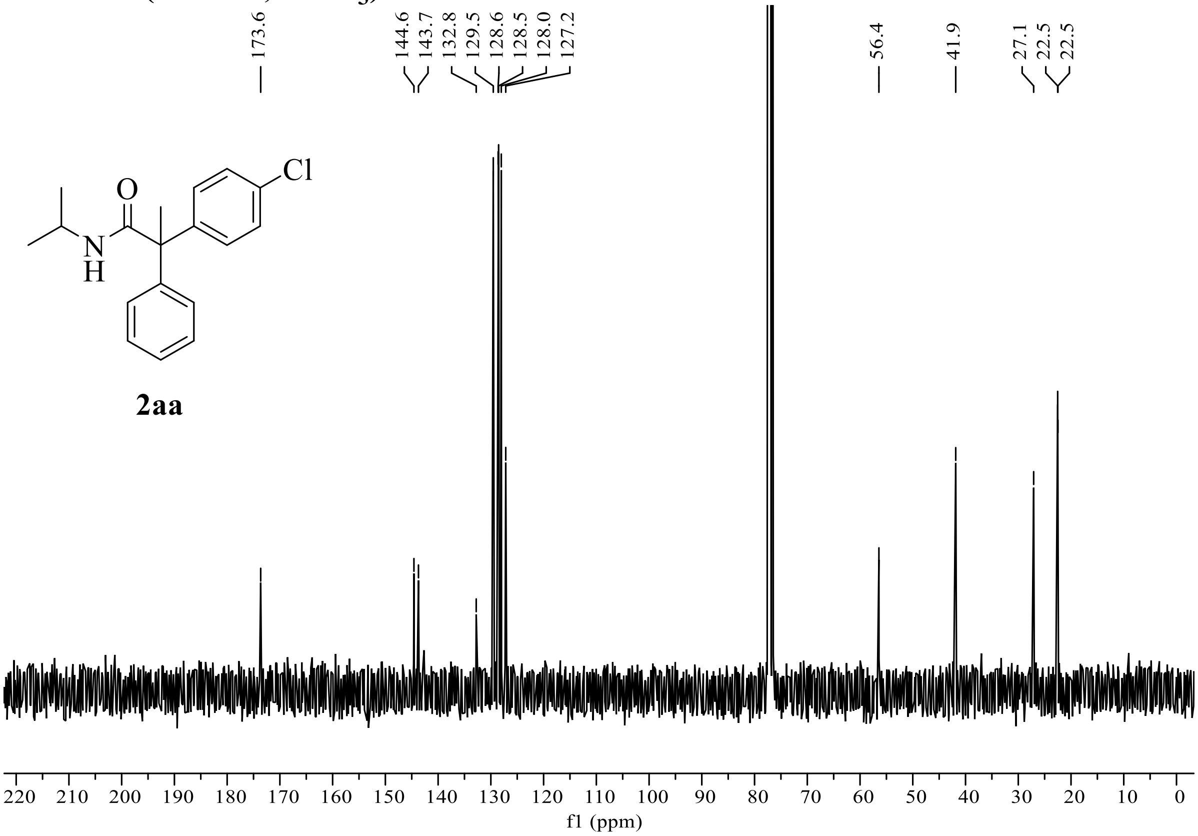

^{1}H NMR (300 MHz, $CDCl_3$):

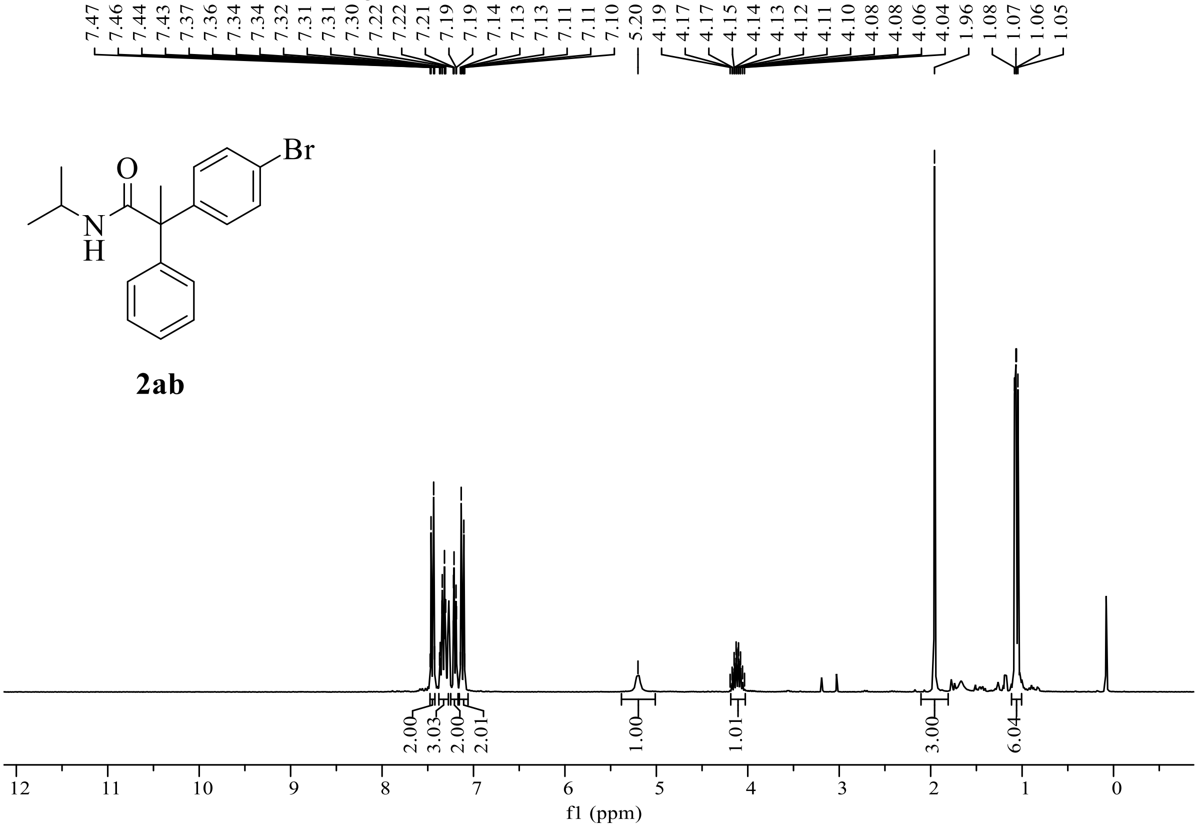

^{13}C NMR (75 MHz, $CDCl_3$):

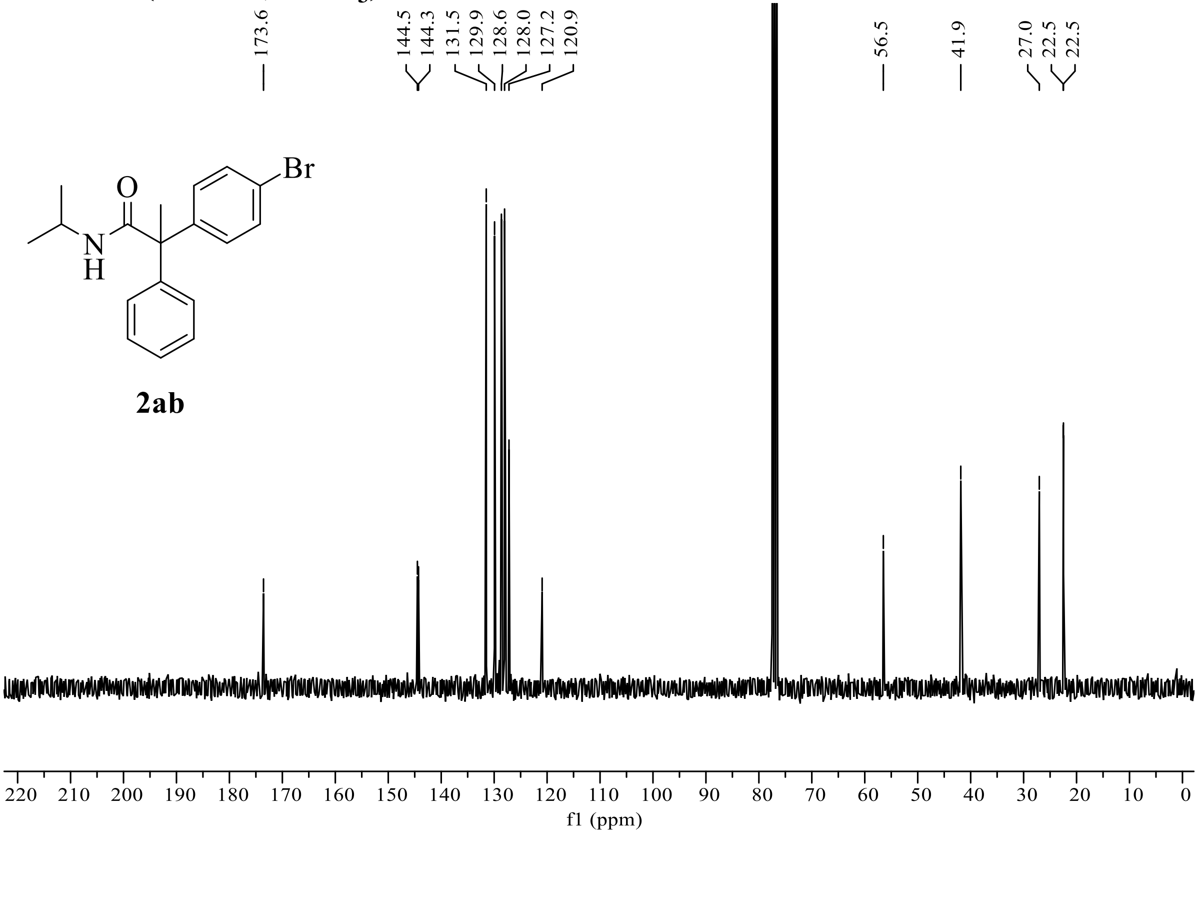

^{1}H NMR (600 MHz, $CDCl_3$):

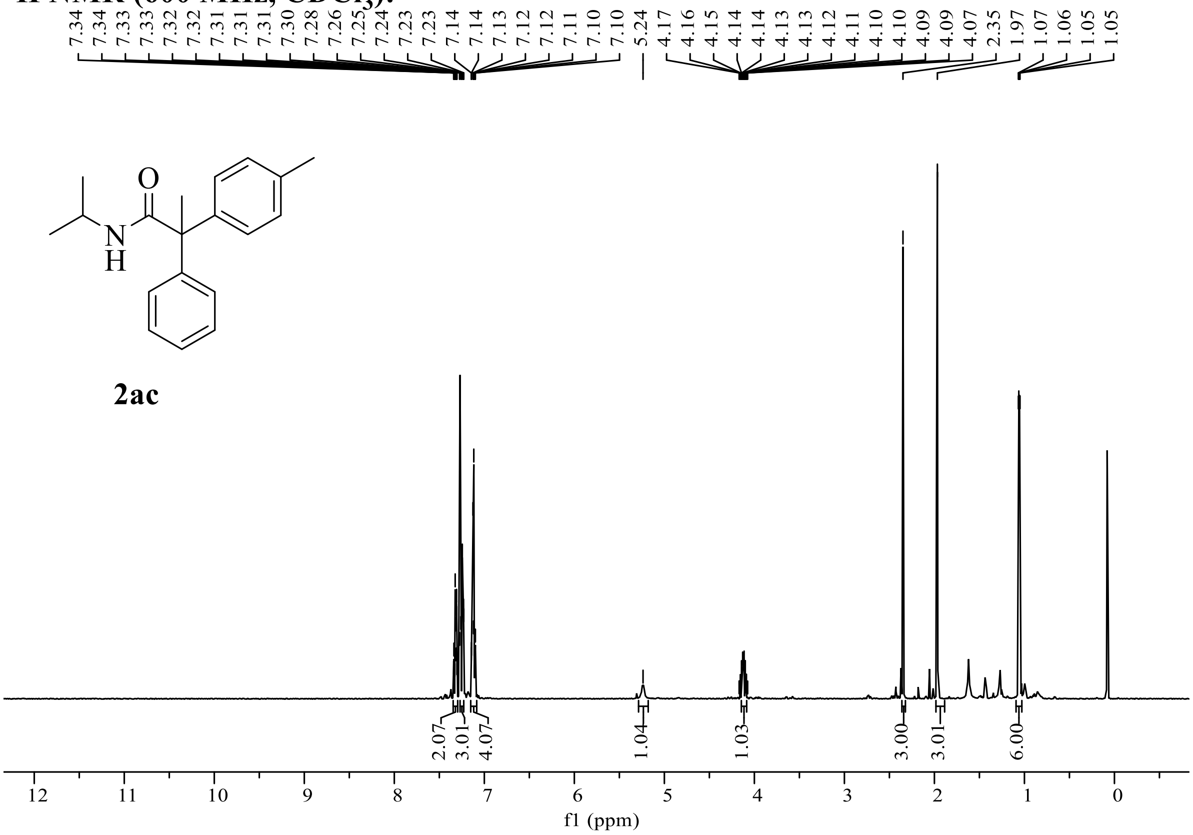

^{13}C NMR (151 MHz, $CDCl_3$):

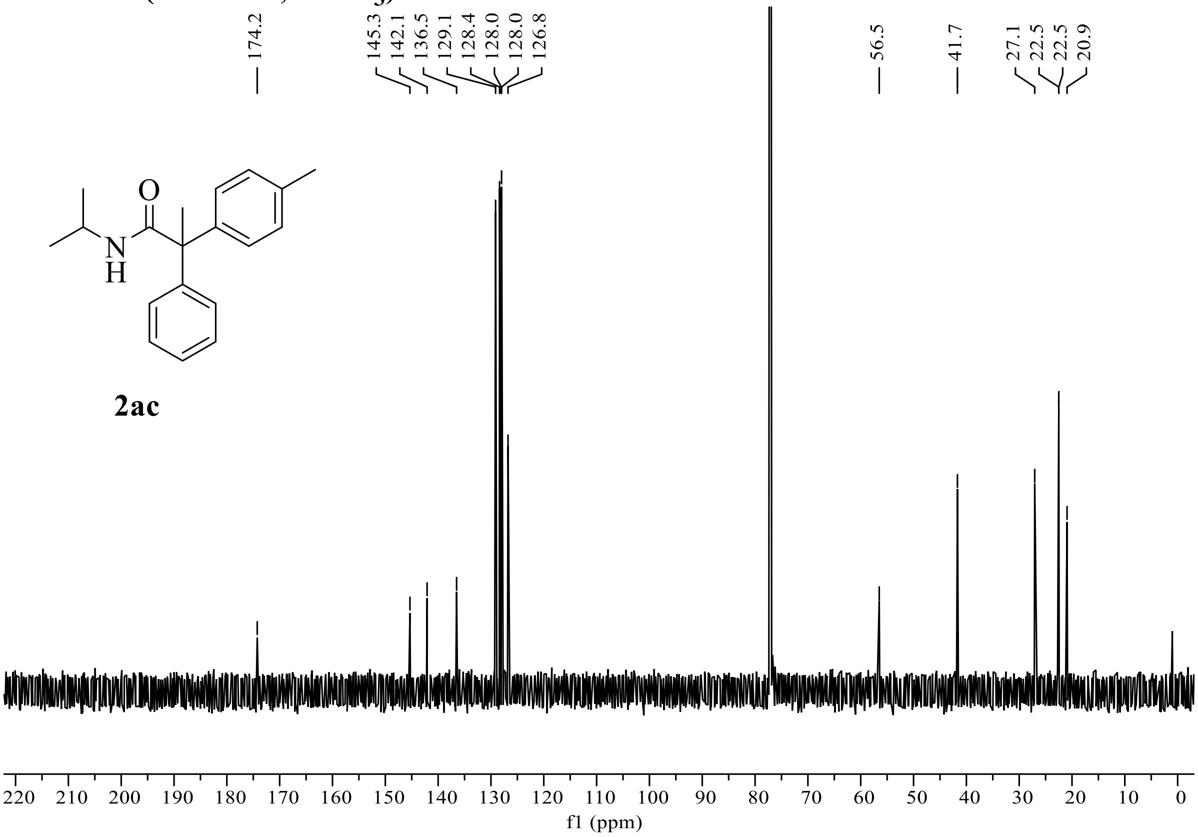

^{1}H NMR (300 MHz, $CDCl_3$):

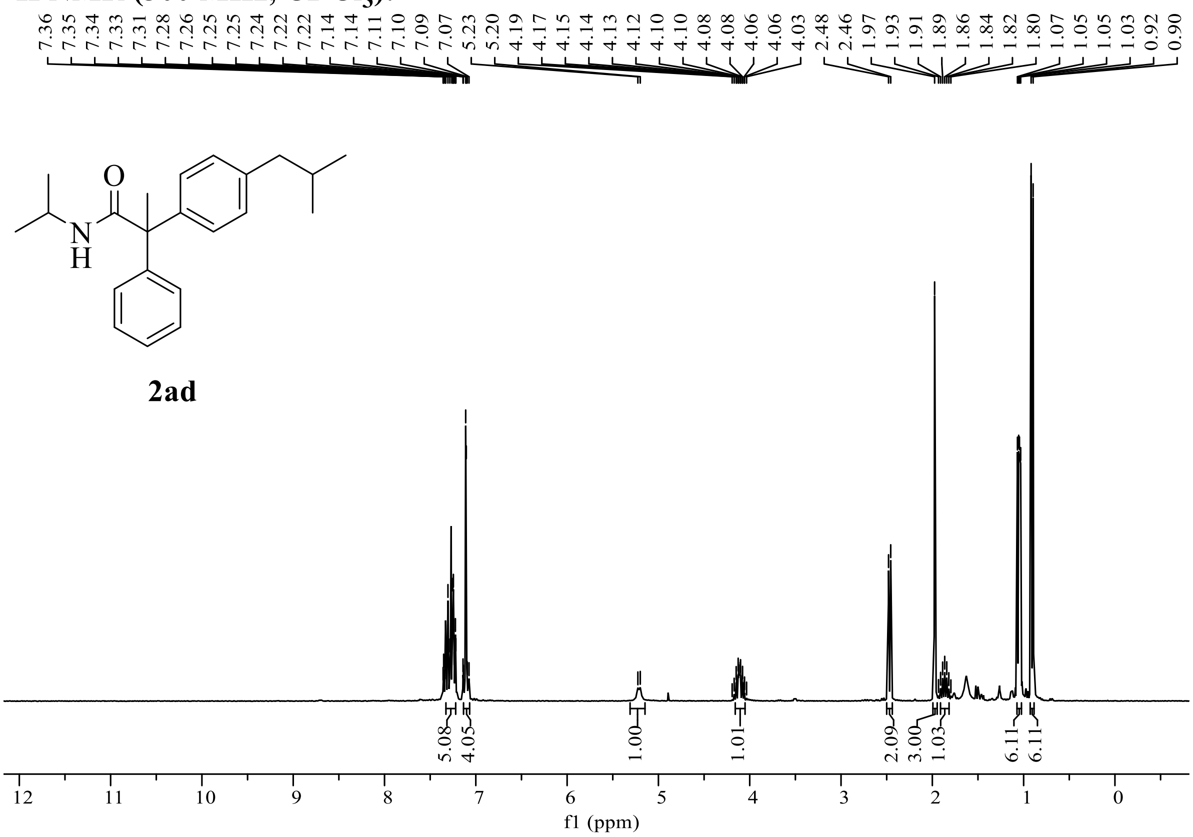

^{13}C NMR (75 MHz, $CDCl_3$):

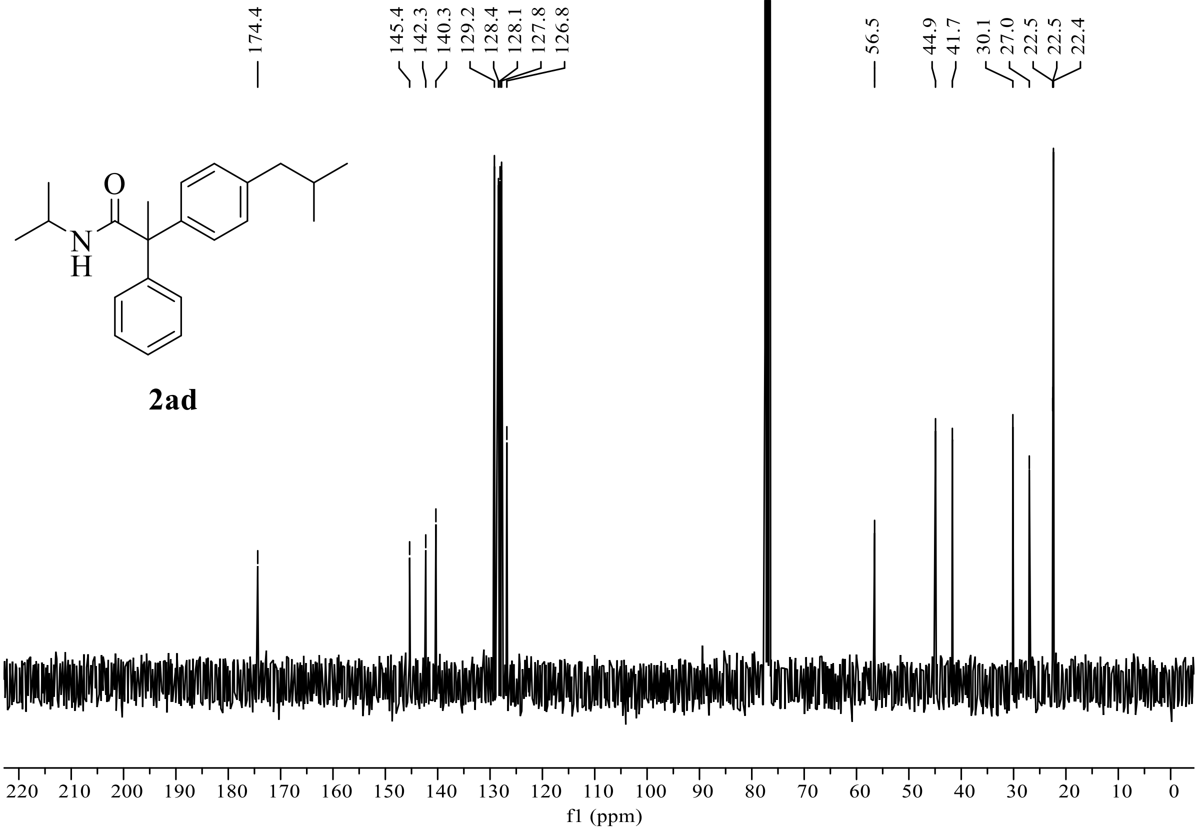

^{1}H NMR (600 MHz, $CDCl_3$):

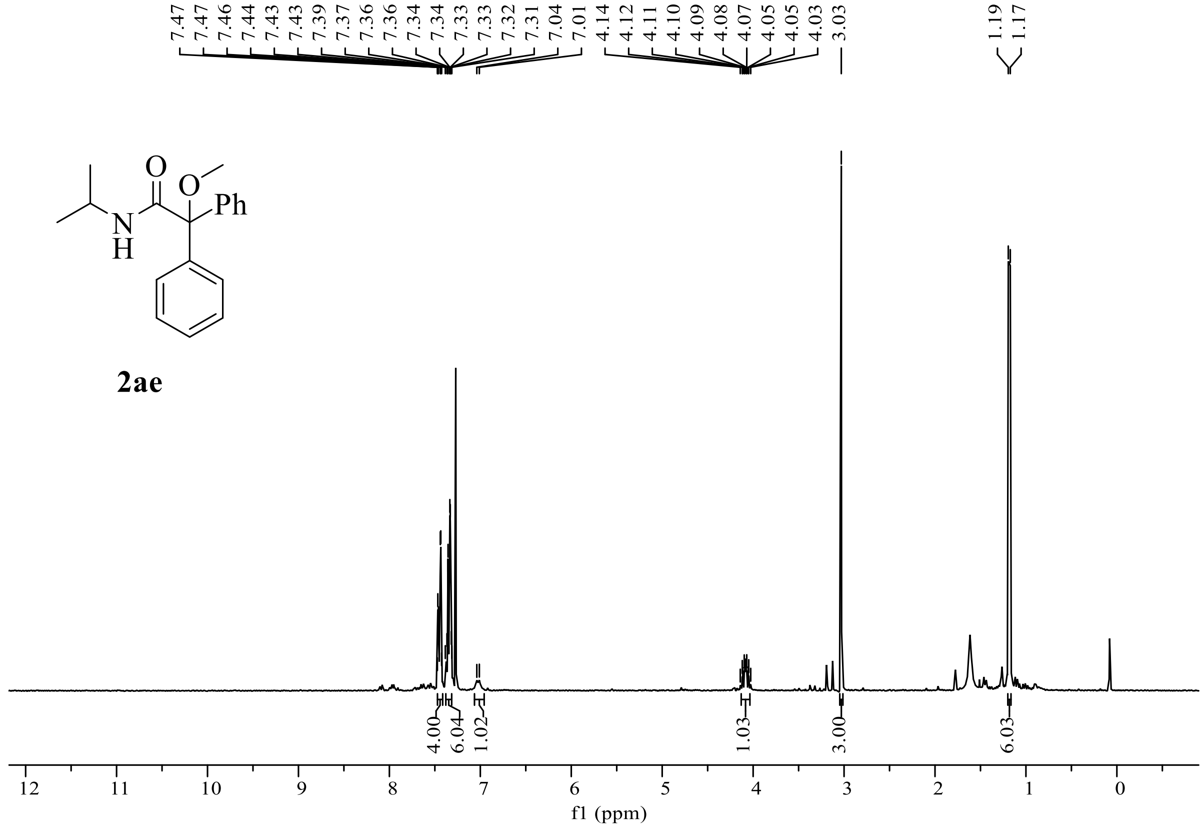

^{13}C NMR (151 MHz, $CDCl_3$):

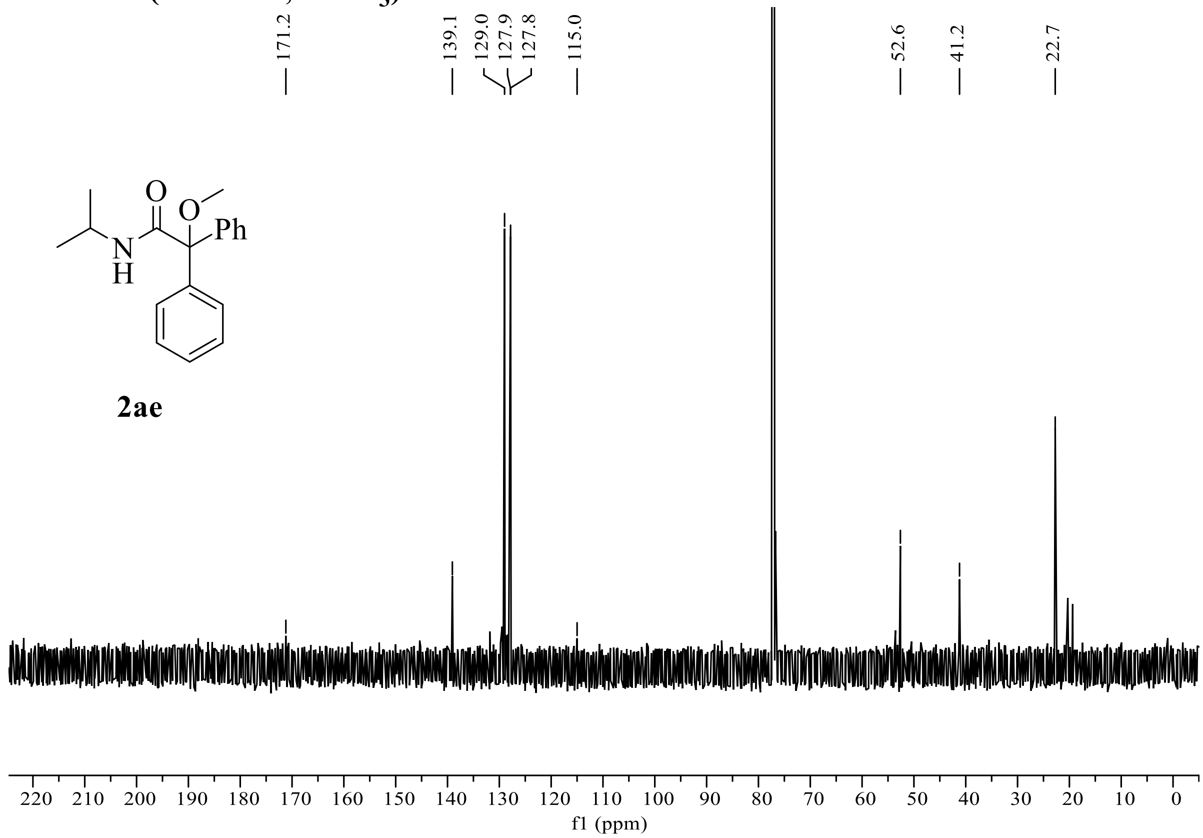

^{1}H NMR (300 MHz, $CDCl_3$):

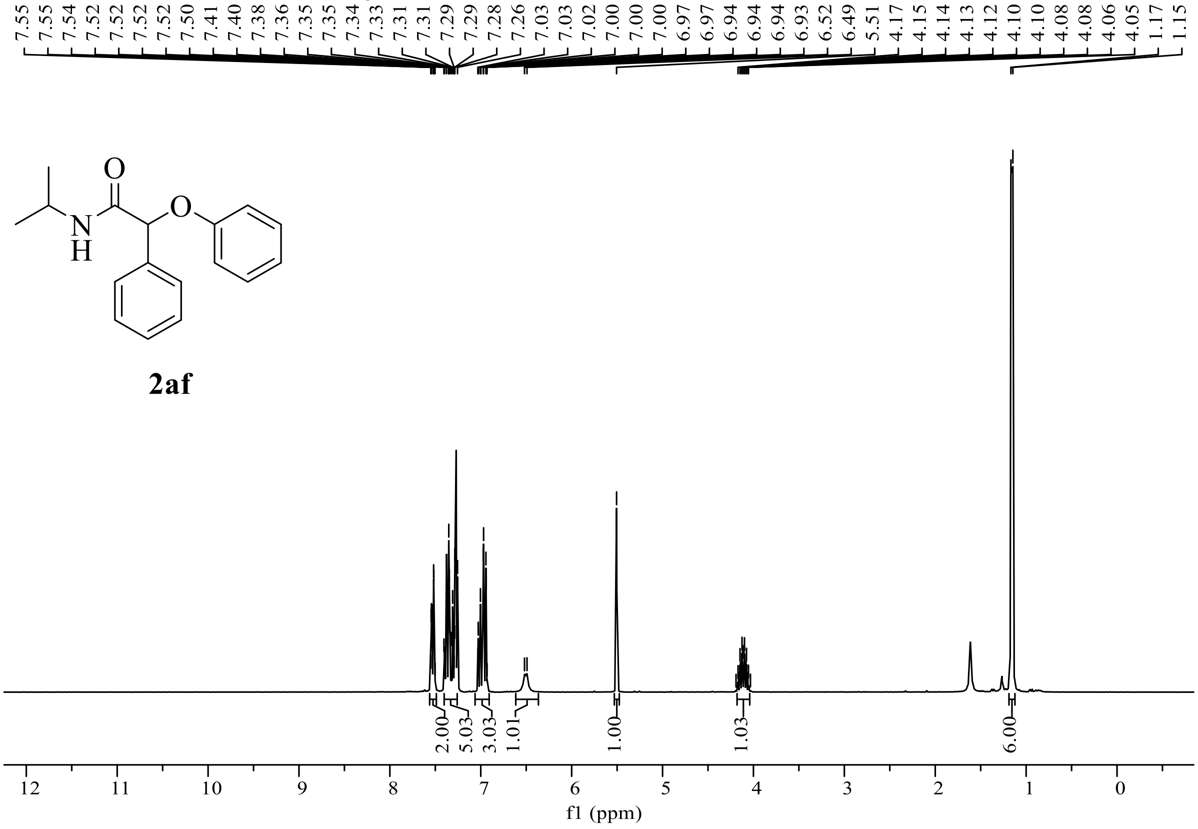

^{13}C NMR (75 MHz, $CDCl_3$):

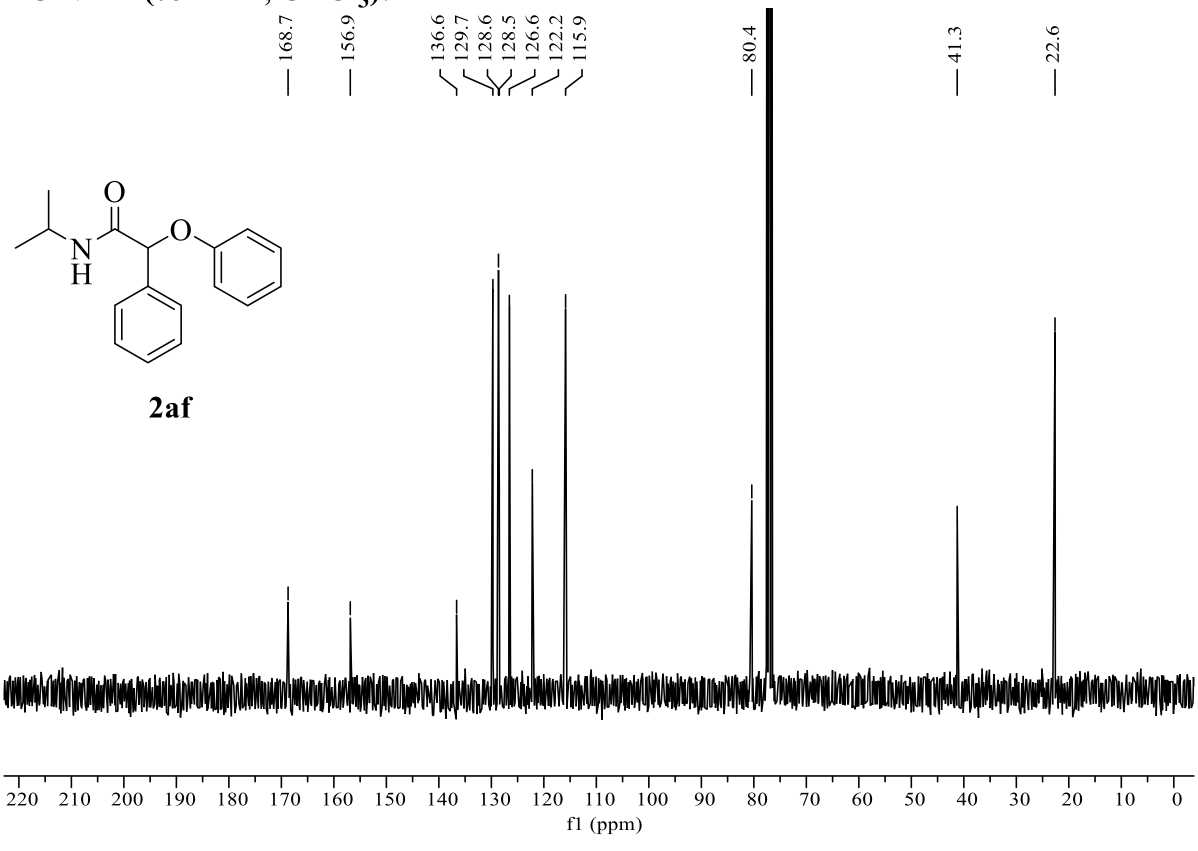

^{1}H NMR (300 MHz, $CDCl_3$):

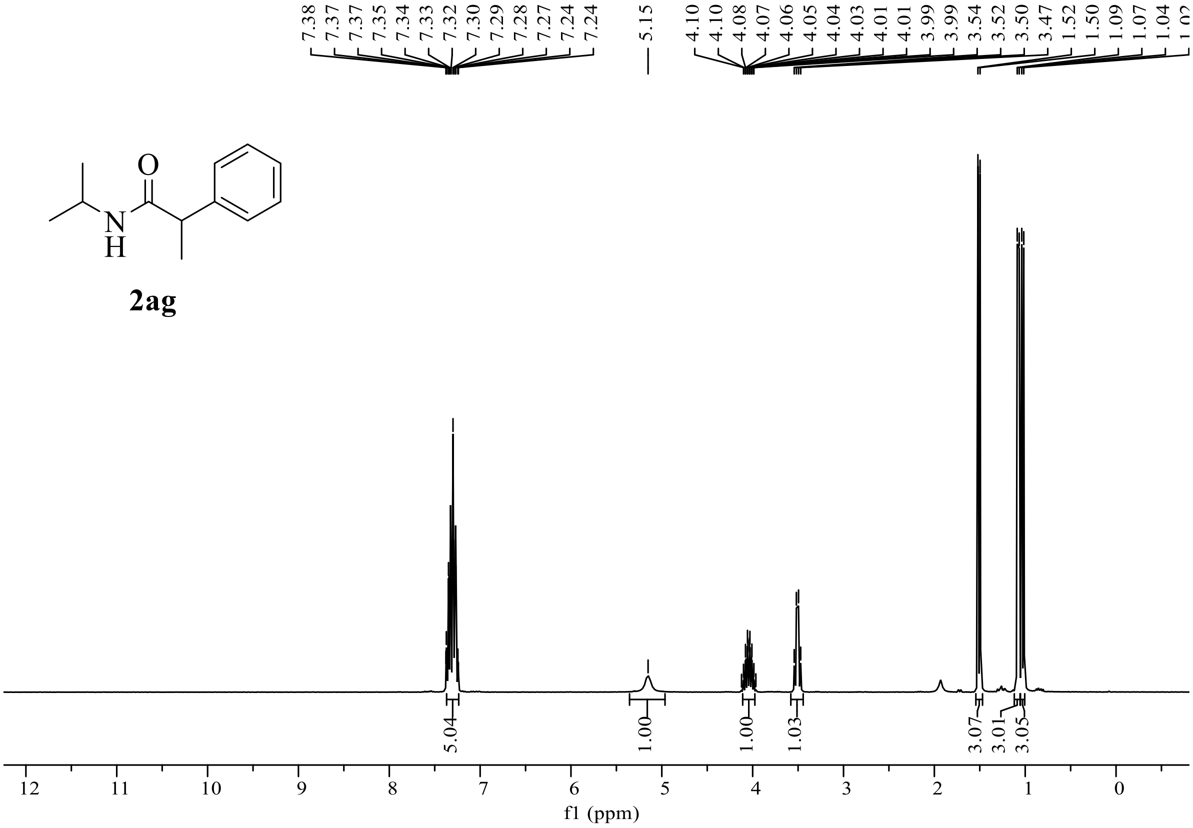

^{13}C NMR (75 MHz, $CDCl_3$):

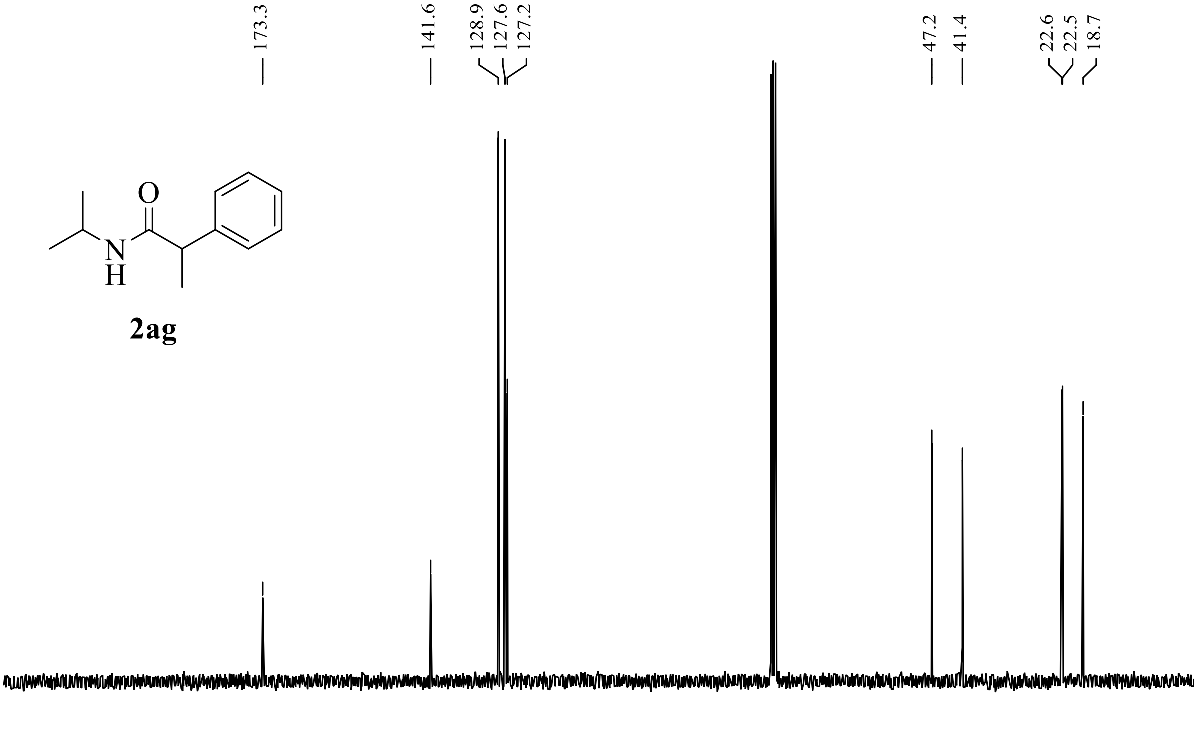

^{1}H NMR (300 MHz, $CDCl_3$):

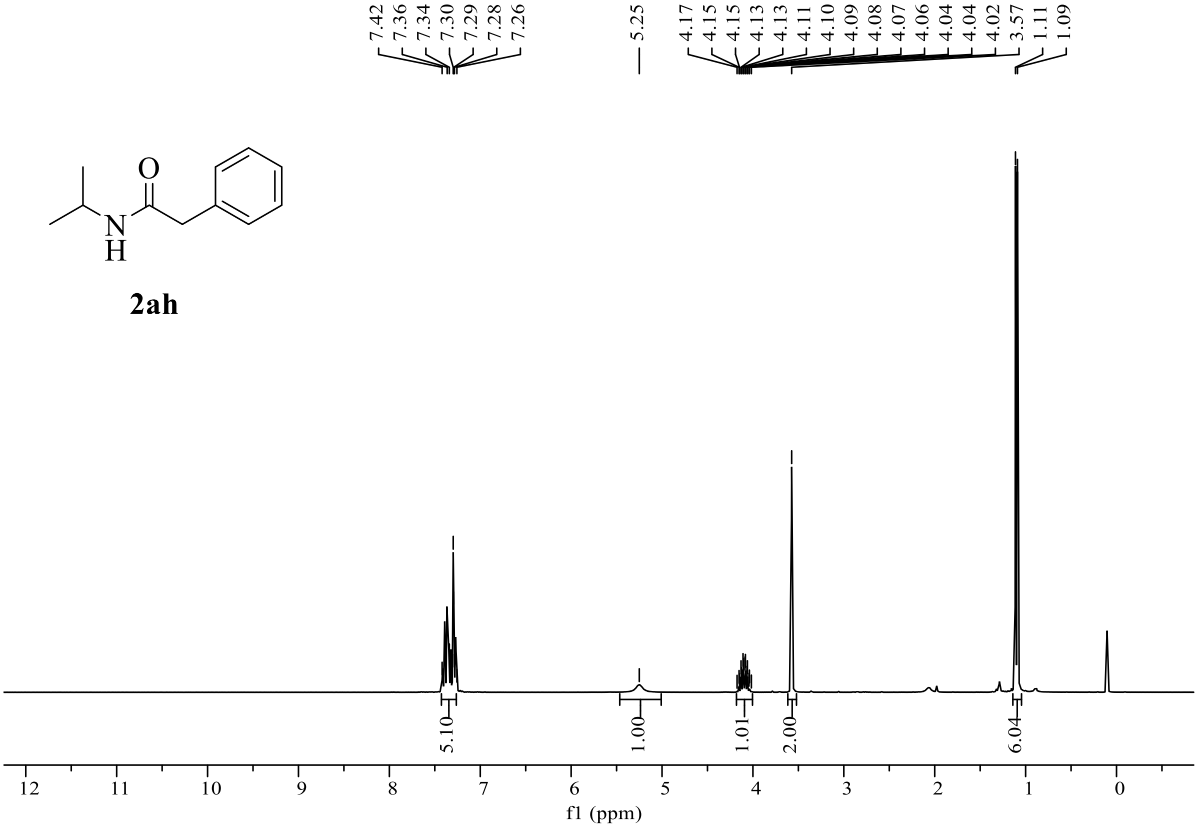

^{13}C NMR (75 MHz, $CDCl_3$):

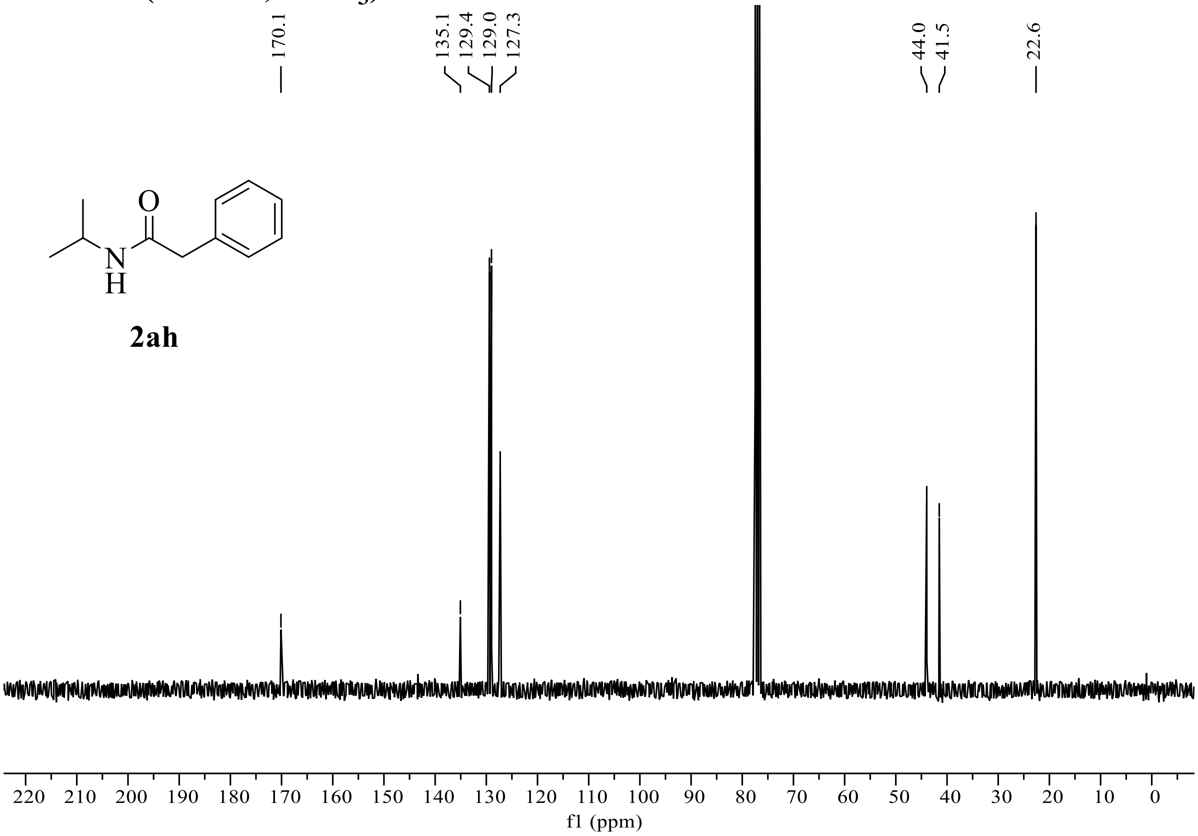

8. References

[1] M. M. Chau, J. L. Kice, *J. Org. Chem* **1977**, *42*, 3265 – 3270.

[2] H. G. O. Becker, *Organikum: Organisch-chemisches Grundpraktikum,* Wiley-VCH, Weinheim, **1999**.

[3] B. Niu, P. Xie, Z. Bian, W. Zhao, M. Zhang, Y. Zhou, L. Feng, C. Pittman, A. Zhou, *Synlett* **2015**, *26*, 635 – 638.

[4] Bayer CropScience AG, US20100285964A1, **2010**.

[5] Y. Xia, L. Wang, A. Studer, *Angew. Chem. Int. Ed.* **2018**, *57*, 12940 – 12944; *Angew. Chem.* **2018**, *130*, 13122 – 13126.

[6] J. Rabai, *Synthesis* **1989**, 523 – 525.

[7] K. S. Etsè, B. Dassonneville, G. Zaragoza, A. Demonceau, *Tetrahedron Lett.* **2017**, *58*, 789 – 793.

[8] T. Tabuchi, T. Yamamoto, M. Nakayama, EP1174028, **2002**.

[9] D. K. Barange, Y.-C. Tu, V. Kavala, C.-W. Kuo, C.-F. Yao, *Adv. Synth. Catal.* **2011**, *353*, 41 – 48.

[10] Kehne, H., Willins, L., Ort, O., Bauer, K., Bieringer, K., US5529976, **1996**.

[11] Y. Li, J. Pu, X. Jiang, *Org. Lett.* **2014**, *16*, 2692 – 2695.

[12] F. W. Friese, C. Mück-Lichtenfeld, A. Studer, *Nat. Commun.* **2018**, *9*, 2808 – 2814.

[13] R. Anana, P. N. P. Rao, Q.-H. Chen, E. E. Knaus, *J. Bioorg. Med. Chem.* **2006**, *14*, 5259 – 5265.

[14] C. K. F. Hermann, J. A. Campbell, T. D. Greenwood, J. A. Lewis, J. F. Wolfe, *J. Org. Chem* **1992**, *57*, 5328 – 5334.

[15] Y. Li, B. Hu, W. Dong, X. Xie, J. Wan, Z. Zhang, *J. Org. Chem.* **2016**, *81*, 7036 – 7041.

[16] J. Hu, T. Lan, Y. Sun, H. Chen, J. Yao, Y. Rao, *ChemComm* **2015**, *51*, 14929 – 14932.

[17] K. R. A. Abdellatif, M. A. Chowdhury, Y. Dong, D. Das, G. Yu, C. A. Velázquez, M. R. Suresh, E. E. Knaus, *Bioorg. Med. Chem. Lett.* **2009**, *19*, 3014 – 3018.

[18] C. E. Olsen, M. Elander, L. Manhem, G. Borch, S. Liaaen-Jensen, *Acta Chem. Scand.* **1975**, *29b*, 953 – 962.

[19] Y. Kawagoe, K. Moriyama, H. Togo, *Tetrahedron* **2013**, *69*, 3971 – 3977.

[20] L. Ling, C. Chen, M. Luo, X. Zeng, *Org. Lett.* **2019**, *21*, 1912 – 1916.

[21] C. R. Opie, H. Noda, M. Shibasaki, N. Kumagai, *Chem. Eur. J.* **2019**, *25*, 4648 – 4653.

[22] A. Charvieux, L. Le Moigne, L. G. Borrego, N. Duguet, E. Métay, *Chem. Eur. J.* **2019**, *40*, 6842 – 6846.

[23] D. G. McCarthy, A. F. Hegarty, *J. Chem. Soc., Perkin Trans. 2* **1980**, 579 – 591.
